# Health system resilience during the COVID‐19 pandemic: A comparative analysis of disruptions in care from 32 countries

**DOI:** 10.1111/1475-6773.14382

**Published:** 2024-09-18

**Authors:** Jorge R. Ledesma, Stavroula A. Chrysanthopoulou, Mark N. Lurie, Jennifer B. Nuzzo, Irene Papanicolas

**Affiliations:** ^1^ Department of Epidemiology Brown University School of Public Health Providence Rhode Island USA; ^2^ Department of Biostatistics Brown University School of Public Health Providence Rhode Island USA; ^3^ International Health Institute Brown University School of Public Health Providence Rhode Island USA; ^4^ Population Studies and Training Center Brown University Providence Rhode Island USA; ^5^ Pandemic Center Brown University School of Public Health Providence Rhode Island USA; ^6^ Department of Health Services, Policy & Practice Brown University School of Public Health Providence Rhode Island USA

**Keywords:** comparative analysis, COVID‐19 pandemic, disruptions, essential health services, health system resilience, hospitalizations

## Abstract

**Objective:**

To quantify disruptions in hospitalization and ambulatory care throughout the coronavirus disease 2019 (COVID‐19) pandemic for 32 countries, and examine associations of health system characteristics and COVID‐19 response strategies on disruptions.

**Data Sources:**

We utilized aggregated inpatient hospitalization and surgical procedure data from the Organization for Economic Co‐operation and Development Health Database from 2010 to 2021. Covariate data were extracted from the Organization for Economic Co‐operation and Development Health Database, World Health Organization, and Oxford COVID‐19 Government Response Tracker.

**Study Design:**

This is a descriptive study using time‐series analyses to quantify the annual effect of the COVID‐19 pandemic on non‐COVID‐19 hospitalizations for 20 diagnostic categories and 15 surgical procedures. We compared expected hospitalizations had the pandemic never occurred in 2020–2021, estimated using autoregressive integrated moving average modeling with data from 2010 to 2019, with observed hospitalizations. Observed‐to‐expected ratios and missed hospitalizations were computed as measures of COVID‐19 impact. Mixed linear models were employed to examine associations between hospitalization observed‐to‐expected ratios and covariates.

**Principal Findings:**

The COVID‐19 pandemic was associated with 16,300,000 (95% uncertainty interval 14,700,000–17,900,000; 18.0% [16.5%–19.4%]) missed hospitalizations in 2020. Diseases of the respiratory (−2,030,000 [−2,300,000 to −1,780,000]), circulatory (−1,680,000 [−1,960,000 to −1,410,000]), and musculoskeletal (−1,480,000 [−1,720,000 to −1,260,000]) systems contributed most to the declines. In 2021, there were an additional 14,700,000 (95% uncertainty interval 13,100,000–16,400,000; 16.3% [14.9%–17.9%]) missed hospitalizations. Total healthcare workers per capita (*β* = 1.02 [95% CI 1.00, 1.04]) and insurance coverage (*β* = 1.05 [1.02, 1.09]) were associated with fewer missed hospitalizations. Stringency index (*β* = 0.98 [0.98, 0.99]) and excess all‐cause deaths (*β* = 0.98 [0.96, 0.99]) were associated with more missed hospitalizations.

**Conclusions:**

There was marked cross‐country variability in disruptions to hospitalizations and ambulatory care. Certain health system characteristics appeared to be more protective, such as insurance coverage, and number of inputs including healthcare workforce and beds.

**What is known on this topic:**

- Substantial disruptions in health services associated with the coronavirus disease 2019 pandemic have placed a renewed interest in health system resilience.
- While there is a growing body of evidence documenting disruptions in services, there are limited comparative assessments across diverse countries with different health system designs, preparedness levels, and public health responses.
- Learning and adapting from health system‐specific gaps and challenges highlighted by the pandemic will be critical for improving resilience.

**What this study adds:**

- All countries experienced disruptions to hospitalizations and surgical procedures with a combined total of 30 million missed hospitalizations and 4 million missed surgical procedures in 2020–2021, but there was marked cross‐country heterogeneity in disruptions.
- Countries with greater baseline healthcare workers, insurance coverage, and hospital beds had disproportionately lower disruptions in care.
- National health planning discussions may need to balance health system resiliency and efficiency to avert preventable morbidity and mortality.

## 1 INTRODUCTION

The coronavirus disease 2019 (COVID‐19) pandemic placed health systems across the globe under extreme stress. In an effort to protect healthcare systems from unanticipated surges in care resulting from COVID‐19, countries implemented unprecedented public health measures^1^ and postponed elective surgical procedures.^2^ These responses combined with the widespread nature of the pandemic likely contributed to the over 90% of all countries regularly reporting disruptions to essential healthcare services throughout the pandemic.^3^, ^4^ While some early country‐specific analyses showcased substantial disruptions to hospital‐based care,^5^, ^6^, ^7^ the complete scale, and duration, of these disruptions across countries with heterogeneous health systems and varied national COVID‐19 response strategies remains unknown.

Equally important, disruptions in health services associated with the COVID‐19 pandemic have highlighted an urgent need to strengthen health system resilience across countries. Although a shared definition of resilience has been difficult to establish, several commonalities exist across those most widely used. Health system resilience captures the ability to prepare, mitigate, adapt to, and recover from shocks and stressors while maintaining core functions and serving ongoing care needs.^8^, ^9^ Other definitions add that a core component of resilience also involves learning lessons from previous experiences and shocks to improve performance and augment preparedness.^10^ Comprehensively describing how COVID‐19 interrupted health service across different contexts is therefore critical for learning from health system‐specific gaps and challenges highlighted by the pandemic.

The primary manner resilience has been studied throughout the pandemic is by measuring changes in health system outputs before, during, and after shocks.^11^ The most common examples are from several studies investigating changes in hospital volume due to the COVID‐19 pandemic,^12^, ^13^, ^14^ but many of these studies are limited to a single country, specific conditions, or were conducted early in the pandemic. Comparative population‐based assessments across diverse countries, with different health system designs, pandemic preparedness and public health responses are limited, yet comparative approaches provide the unique opportunity to learn how these differences may have contributed to health system resilience and pandemic recovery across settings.^11^ For example, countries with stronger performance and preparedness coming into the pandemic may have experienced fewer disruptions in care. However, it is also possible that the features that make a health system resilient also differ from those that make a system “high performing,” often defined as a health system maximizing its key objectives such as health attainment considering existing inputs.^15^ For example, the past decades have seen many countries seeking to improve their performance by making their systems more efficient, and in taking measures that reduce excess capacity, such as removing unused hospital beds.^16^ However, during the COVID‐19 pandemic a short supply of beds made the health system less equipped to deal with the sudden surge in demand.^17^, ^18^ More work is urgently needed to better understand the relationship between health system factors, health system performance, and resilience as countries recover from the pandemic.

To address these gaps, we draw on population‐based time‐series data from 32 countries on inpatient hospitalizations for 20 diagnostic categories, five avoidable hospital admissions, and on 15 surgical procedures to examine cross‐country changes in hospitalizations and ambulatory care utilization associated with the COVID‐19 pandemic. The 32 included countries vary in their health system designs, socio‐demographic characteristics, COVID‐19 severity levels, and in their national COVID‐19 response strategy. We leverage these variabilities by subsequently examining country‐specific factors that may have promoted resilience or worsened disruptions. We therefore set out to answer the following three questions:

1. How were hospitalization and ambulatory care services interrupted throughout the COVID‐19 pandemic?
2. Which health system‐specific characteristics were associated with greater resilience?
3. Which national COVID‐19 response strategies were associated with worsened disruptions?

These analyses provide new insights in cross‐country variability in health service disruptions for improving health policy aiming to strengthen resilience and preparedness.

## 2 METHODS

### 2.1 Study design and data

We conducted a descriptive study of disruptions to health services using time‐series analyses to quantify the annual effect of the COVID‐19 pandemic on non‐COVID‐19 hospitalizations for 20 diagnostic categories, the corresponding average length of hospital stays for each diagnostic category, five avoidable hospital admissions, and 15 surgical procedures across countries in the Organization for Economic Co‐operation and Development (OECD). The data on these four outcomes were extracted from the OECD Health Database^15^ (downloaded on January 30, 2024), which provided yearly data from 2010 to 2021 for OECD countries. For all four outcomes, we only included those OECD countries that reported data in 2020, had at least five consecutive years of data leading to the pandemic, and did not have major changes in data coverage during the time series.

For data on non‐COVID‐19 hospitalizations disaggregated by 20 diagnostic categories, we utilized OECD data on non‐COVID‐19 hospital discharges defined as the release of a patient who stayed at least one night in a hospital (referred to as inpatient hospitalizations throughout this paper). The disease classification scheme is provided elsewhere.^19^ The second outcome was the corresponding average length of stays (LOS) for the non‐COVID‐19 inpatient hospitalizations defined as the average number of days patients spent in a hospital. The average LOS data were included to comprehensively assess how the COVID‐19 pandemic may have impacted trends in hospitalizations. In total, 26 countries available in the database had sufficient time series for both outcomes to be included. Additional information about country‐specific data is available in Table S1.

For avoidable hospitalizations, defined as hospitalizations that can be averted through effective intervention in primary care settings, we extracted data on asthma, chronic obstructive pulmonary disease (COPD), congestive heart failure (CHF), hypertension, and diabetes hospital admission rates. Those admissions that resulted from a transfer from another hospital and where the patient died during admission were excluded as these hospitalizations are likely considered to be unavoidable. The hospitalization rates for these five conditions were age‐sex standardized and only included those aged 15 years and greater.

The final outcome was total counts of surgical procedures for the 15 procedures included in the OECD Health Statistics database (Table S2). The surgical procedure data combined both inpatient cases (procedures on patients formally admitted into a hospital for at least a night) and day cases (procedures on patients who formally admitted in a hospital for receiving a planned surgery and discharged the same day). Based on previous studies,^20^ we further grouped surgical procedures by whether they are elective (non‐emergency procedures that can be delayed) or non‐elective (procedures that must be performed urgently to save patients). We created a third group for those conditions that can be either elective or non‐elective procedures (Table S3). There were 27 countries with sufficient time‐series data on surgical procedures that were included.

Finally, we compiled data from multiple sources to assess country‐level factors that may be associated with greater or fewer disruptions in non‐COVID‐19 hospitalizations. We utilized data from the OECD Health Database^19^ to extract baseline country‐level health system characteristics (e.g., healthcare workforce, beds per capita, healthcare spending) and the World Bank for baseline socio‐demographic characteristics (e.g., GDP per capita, fraction of population ≥65). Public Health and Social Measure (PHSM) data (e.g., stringency index, stay‐at‐home orders) were collated from the Oxford COVID‐19 Government Response Tracker (OxCGRT) database.^21^ Information on pandemic preparedness was extracted from the Global Health Security Index.^22^ COVID‐19 severity data (e.g., case rate, death rate, excess deaths) were gathered from the World Health Organization.^23^ Complete details of the country‐level characteristics are available in Table S4.

### 2.2 Construction of time‐series models

Autoregressive integrated moving average (ARIMA) time‐series modeling was used to predict 2020–2021 non‐COVID‐19 inpatient hospitalizations, the corresponding LOS, and surgical procedures as a function of yearly data from 2010 to 2019, with the aim of estimating counterfactual rates if there had been no global COVID‐19 pandemic. Hospitalizations were predicted for each country and diagnosis/surgery combination in the absence of the global pandemic. We also aggregated hospitalization rates across the countries to predict an OECD average of hospital volume. ARIMA models are popular methods for fitting time series (proposed by Box et al.^24^, ^25^) and have been widely used to described and predict relevant trends during the COVID‐19 pandemic.^26^, ^27^ Data used as input into the ARIMA models were log‐transformed prior to modeling. We employed an automated approach for finding the combination of pdq parameter values (ARIMA parameters where *p* is the number of lagged values included in the autoregressive dependence, d is the order of differencing applied to the data, and q is the number of terms in the moving average dependence) that best fits the observed hospitalization trends, based on the Akaike's Information Criterion (AIC). The final model was then used to predict counterfactual expected hospitalizations in the absence of the COVID‐19 pandemic for 2020 and 2021 with corresponding 95% prediction intervals (PIs) as a function of historical trends (2010–2019).

### 2.3 Statistical analysis

We compared predicted country‐level hospitalizations in 2020–2021 with observed hospitalizations to derive measures of the impact of the COVID‐19 pandemic by computing yearly observed‐to‐expected (OE) ratios with values less or greater than one indicating fewer or more hospitalizations than expected. As another measure, we computed the difference of observed hospitalization to expected hospitalization to quantify the change in hospitalizations associated with the pandemic (missed hospitalizations). We used Monte Carlo simulations to evaluate the uncertainty of the model projections by generating 10,000 predictions and calculating 95% uncertainty intervals (UIs) using the 2.5th and 97.5th percentiles of the simulated values for OE ratios and changes in hospitalizations.

Finally, we used mixed linear models to assess associations between country and ICD diagnostic category‐specific OE ratios on health system factors, socio‐demographic characteristics, PHSMs, and COVID‐19 severity levels. To quantify each association, we ran separate models for each variable of interest but included log‐transformed income (pre‐pandemic gross domestic product per capita), pre‐pandemic health expenditure, the stringency index, and COVID‐19 death rate as covariates to improve comparability across countries while also incorporating random intercept terms on country. Owing to high correlation between variables, there were some differences in included covariates in each regression to prevent variance inflation. For example, in the excess deaths models, we removed the COVID‐19 death rate covariate, and in the individual PHSM models (e.g., school closures, stay‐at‐home, etc.), we removed the stringency index. OE ratios were log‐transformed prior to analyses to examine the relative impacts of variables. We standardized all variables to improve comparability across regression parameters. Mixed linear models were stratified by inpatient hospitalization OE ratios and surgical procedure OE ratios. Effect sizes above 0 (or 1 if exponentiated) indicate that the characteristic was associated with fewer disruptions while effect sizes below 0 (or 1 if exponentiated) indicate the factor was associated with greater disruptions.

## 3 RESULTS

### 3.1 Trends in non‐COVID‐19 inpatient hospitalizations

**FIGURE 1:**
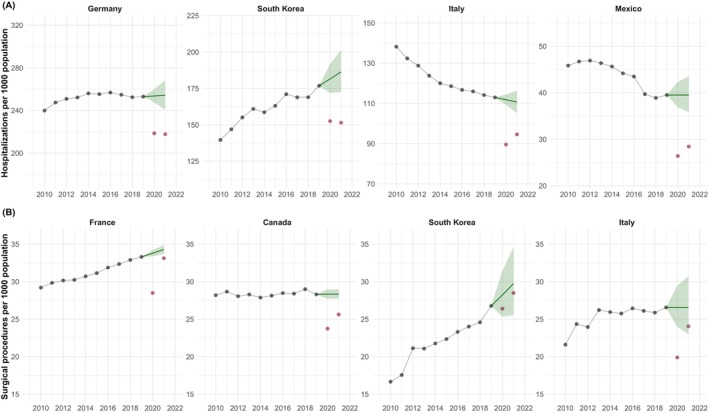
Rates of (A) all‐cause hospitalizations and (B) surgical procedures per 1000 population for select countries, 2010–2021. The countries shown here had the largest number of hospitalizations/surgical procedures in 2019 among the included countries. The black circles represent annual observed rates prior to the pandemic (2010–2019), whereas the red circles are observed rates during the pandemic. The dark green line is the predicted rate had the global coronavirus disease 2019 pandemic never occurred estimated from country‐specific autoregressive integrated moving average (ARIMA) models using pre‐pandemic data (2010–2019). The corresponding shaded areas represent 95% prediction intervals from the ARIMA models.

**FIGURE 2:**
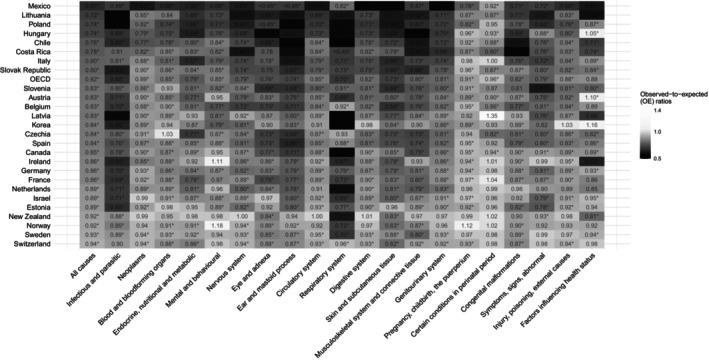
Observed‐to‐expected (OE) ratios for inpatient hospitalizations by diagnostic categories in 2020. The star (*) indicates that the 95% uncertainity interval for the OE ratio does not contain 1. Color gradient‐ and year‐specific version of figure can be found in Figure S1. OECD, Organization for Economic Co‐operation and Development.

Among the 26 OECD countries included in this analysis, 90,400,000 (95% PI 88,800,000–92,000,000) all‐cause non‐COVID‐19 inpatient hospitalizations were expected under normal conditions in 2020, compared with an observed 74,100,000 hospitalizations (Table S5). This corresponded to 16,300,000 (95% UI 14,700,000–17,900,000; 18.0% [16.5%–19.4%]) fewer hospitalizations than expected with an OE ratio of 0.82 (0.81–0.83). Diagnostic categories that contributed most to these declines were diseases of the respiratory (−2,030,000), circulatory (−1,680,000), and musculoskeletal (−1,480,000) systems. Among the OECD countries, Germany (−2,910,000, OE ratio = 0.86), France (−1,700,000, OE ratio = 0.86), Poland (−1,680,000, OE ratio = 0.74), Mexico (−1,670,000, OE ratio = 0.67), and South Korea (−1,500,000, OE ratio = 0.84) had the largest declines in all‐cause hospitalizations in 2020 (Figures 1 and 2).

In 2021, there were 75,200,000 observed all‐cause non‐COVID‐19 hospitalizations compared with 89,900,000 (95% PI 88,300,000–91,500,000) expected hospitalizations (Table S5). This yielded 14,700,000 (95% UI 13,100,000–16,400,000; 16.3% [14.9%–17.9%]) fewer hospitalizations than expected with an OE ratio of 0.84 (0.82–0.85). The same diagnostic categories and countries that experienced the greatest declines in 2020 continued to experience the largest drops in 2021. Countries with the largest improvements in all‐cause OE ratios were Poland (from 0.74 to 0.82), Belgium (from 0.83 to 0.90), Israel (from 0.89 to 0.96), and Italy (from 0.81 to 0.85). Hungary (from 0.74 to 0.59) and Slovak Republic (from 0.80 to 0.73) were countries that had considerably lower OE ratios in 2021 than that in 2020 (Figure S1).

In regards to pandemic‐associated changes in average LOS by diagnostic category, there were increases in average LOS for mental and behavior disorders (5.48 [1.83–8.89] more days, OE ratio = 1.15), respiratory diseases (1.47 [1.22–1.71] more days, OE ratio = 1.20), and infectious diseases (1.39 [1.16–1.62] more days, OE ratio = 1.17) in 2020 compared with what was expected under normal conditions for all OECD countries combined (Figure S2; Table S5). In 2021, respiratory (OE ratio = 1.21 [1.16–1.26]) and infectious diseases (OE ratio = 1.17 [1.12–1.22]) continued to have longer average LOS than expected, but LOS for mental and behavior disorders returned to pre‐pandemic LOS (Figure S2). There were no other substantial changes in LOS for most other diagnostic categories.

### 3.2 Trends in avoidable hospitalizations

In 2020, almost all included countries experienced lower than expected hospitalizations for Asthma and COPD. Mexico (OE ratio = 0.41), United Kingdom (0.50), and Canada (0.52) had the largest declines, whereas Iceland (OE ratio = 0.81), Norway (0.80), and Czechia (0.81) had the smallest drops (Figure S3; Table S6). For CHF and hypertension combined, 14 of 27 countries experienced lower than expected hospitalizations. Mexico (OE ratio = 0.64), Lithuania (0.69), and Poland (0.71) had the largest declines for CHF and hypertension combined in 2020. Similarly, 14 of 27 countries experienced lower than expected hospitalizations for diabetes with Mexico (OE ratio = 0.69), Lithuania (0.62), and Poland (0.65) having the largest declines.

In 2021, almost all countries continued to have lower than expected hospitalizations for Asthma and COPD combined. The United Kingdom (from 0.50 to 0.67) and Israel (from 0.61 to 0.73) experienced marked increases in OE ratios compared to that in 2020, whereas OE ratios decreased by 0.10 for eight countries in 2021 (Figure S3; Table S6). For CHF and hypertension combined, 14 of 27 countries continued to have significantly lower than expected hospitalizations in 2021. Slovakia (from 0.76 to 0.62), Estonia (from 0.97 to 0.83), and Iceland (from 1.00 to 0.86) experienced the greatest declines in OE ratios for CHF and hypertension combined in 2021. For diabetes, 15 of 27 countries had significantly lower than expected hospitalizations in 2021. Countries having substantially lower OE ratios for diabetes hospital admissions in 2021 than that in 2020 included Latvia (from 0.94 to 0.76), Estonia (from 0.92 to 0.75), and Czechia (from 0.87 to 0.73).

### 3.3 Trends in surgical procedures

**FIGURE 3:**
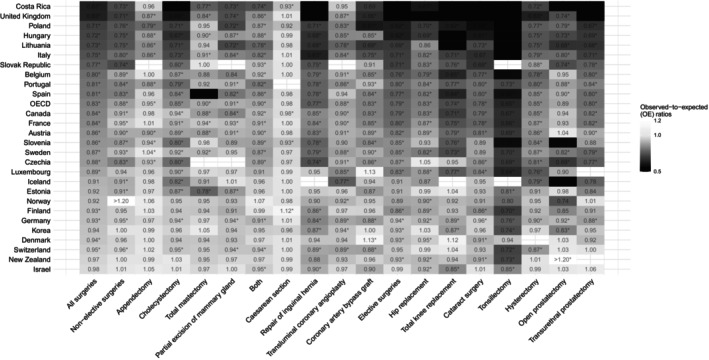
Observed‐to‐expected (OE) ratios for surgeries by procedure in 2020. The star (*) indicates that the 95% uncertainity interval for the OE ratio does not contain 1. Color gradient‐ and year‐specific version of figure can be found in Figure S4. OECD, Organization for Economic Co‐operation and Development.

Among the total 15 surgical procedures included in this analysis, 16,100,000 (95% PI 15,800,000–16,400,000) surgical procedures were expected under normal conditions in 2020, compared with an observed 13,300,000 surgeries (Table S7). This corresponded to −2,780,000 (95% UI −3,080,000 to −2,490,000; 17.3% [15.8%–18.8%]) fewer surgeries than expected with an OE ratio of 0.83 (0.81–0.84). The largest declines were in elective surgeries (−2,070,000, OE ratio = 0.79) followed by non‐elective (−279,000, OE ratio = 0.88) and those that can be considered as both (−422,000, OE ratio = 0.90). Procedures with the largest reductions included knee replacements (−246,000, OE ratio = 0.74), hernia repairs (−235,000, OE ratio = 0.77), and hip replacements (−200,000, OE ratio = 0.83), whereas Caesarean sections (−25,100, OE ratio = 0.98 [0.95–1.02]) and appendectomy (−29,600, OE ratio = 0.95 [0.94–0.96]) had minimal reductions (Figure 3). The United Kingdom (−547,000, OE ratio = 0.63), Italy (−396,000, OE ratio = 0.75), and France (−358,000, OE ratio = 0.84), experienced the largest declines in surgical procedures (Figure 1B). Israel (−4600, OE ratio = 0.98 [0.94–1.02]), New Zealand (−3060, OE ratio = 0.97 [0.93–1.00]), and Switzerland (−11,300, OE ratio = 0.95 [0.91–1.00]) were mostly able to maintain pre‐pandemic trends.

In 2021, there were 14,800,000 observed surgical procedures compared with 16,100,000 (95% PI 15,600,000–16,500,000) expected surgeries (Table S7). This yielded 1,310,000 (95% UI 884,000–1,760,000; 8.17% [5.65%–10.7%]) fewer surgical procedures than expected with an OE ratio of 0.92 (0.89–0.94). Both elective (2021 OE ratio: 0.90) and non‐elective surgeries (2021 OE ratio: 0.96) experienced significant increases in OE ratios while surgeries that can be both (2021 OE ratio: 0.89) remained the same. The United Kingdom (2021 OE ratio: 0.96 [0.89–1.03]), Portugal (2021 OE ratio: 0.98 [0.90–1.08]), Italy (2021 OE ratio: 0.91 [0.78–1.05]), and Belgium (0.95 [0.92–0.99]) had significantly larger OE ratios in 2021 compared to that in 2020 (Figure S4). Hungary (2021 OE ratio: 0.56 [0.52–0.61]) and Denmark (2021 OE ratio: 0.88 [0.85–0.91]) experienced the largest declines of OE ratios in 2021.

### 3.4 Predictors of changes in hospitalizations associated with the COVID‐19 pandemic

**FIGURE 4:**
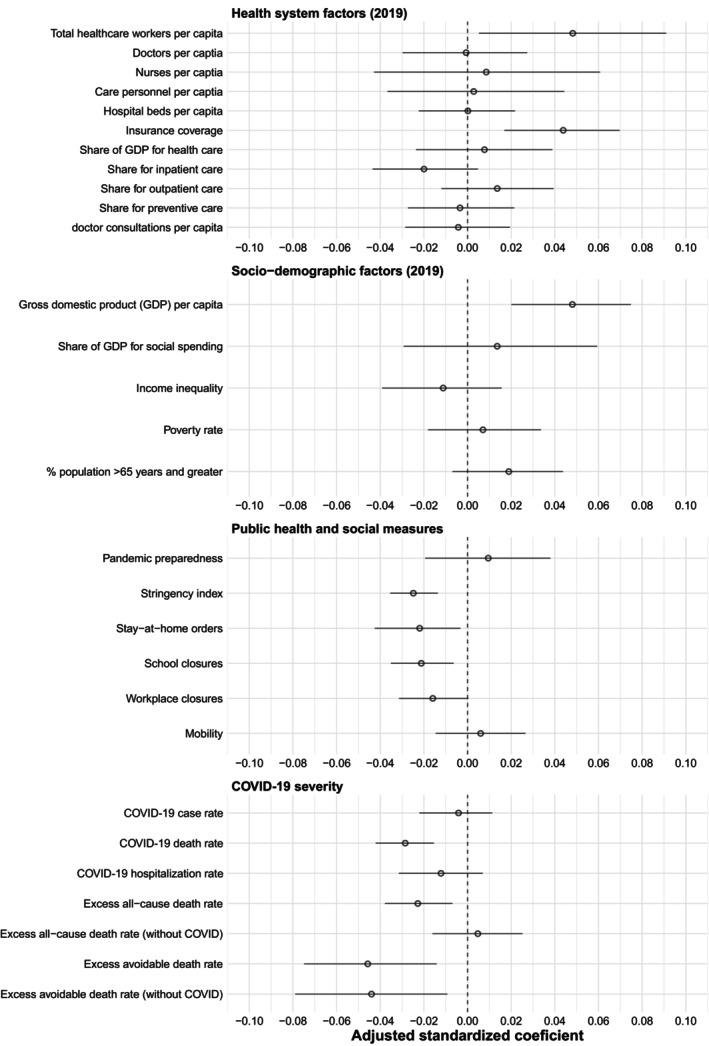
Associations between health system characteristics, socio‐demographic factors, public health and social measures, and coronavirus disease 2019 (COVID‐19) severity on observed‐to‐expected ratios of inpatient hospitalizations. Coefficients are from mixed linear regressions with random intercept terms for country. Separate models were conducted for each variable of interest. In each regression, log‐transformed income (pre‐pandemic gross domestic product per capita), pre‐pandemic health expenditure, the stringency index, and COVID‐19 death rate were included as covariates. Owing to high correlation between variables, there were some differences in included covariates in each regression. In the healthcare workers model, gross domestic product (GDP) per capita was removed due to very high correlation between GDP and healthcare workers (*r* = 0.88). In COVID‐19 severity models, COVID‐19 deaths covariate was removed. In individual public health and social measure models (stay‐at‐home, school closures, workplace closures), the stringency index covariate was removed. The circles represent the standardized coefficients from the multivariate mixed linear regressions while the lines are the corresponding 95% confidence intervals.

For inpatient hospitalizations, total healthcare workers per capita (*β* = 1.02 [95% CI 1.00, 1.04]), insurance coverage (*β* = 1.05 [1.02, 1.09]), and log‐GDP per capita (*β* = 1.00 [1.00, 1.01]) were factors positively associated with OE ratios in adjusted analyses (Figure 4; Table S8). Notably, these results indicate that each 10 per capita increase in healthcare workers was associated with a 2.02% (0.29, 3.80%) increase in OE ratios for inpatient hospitalizations holding country income, healthcare expenditures, PHSMs, and COVID‐19 deaths constant. Similarly, each 5% increase in insurance coverage was associated with a 5.20% (2.00, 8.60%) increase in OE ratios. The stringency index (*β* = 0.98 [0.98, 0.99]), COVID‐19 death rate (*β* = 0.96 [0.94, 0.98]), excess all‐cause death rate (*β* = 0.98 [0.96, 0.99]), and excess avoidable death rate without COVID (*β* = 0.91 [0.84, 0.98]) were some of the factors negatively associated with OE ratios. For the stringency index, each 10‐point increase in the index was associated with a 1.58% (0.87, 2.33%) reduction in OE ratios holding country income, healthcare expenditures, and COVID‐19 deaths constant. In addition, each 1 per 1000 population increase in COVID‐19 deaths, excess all‐cause deaths, and excess avoidable deaths without COVID were each associated with 4.26% (2.33, 6.31%), 2.23% (0.71, 3.71%), and 9.17% (2.20, 15.9%) reductions in OE ratios, respectively.

When surgical procedures were the outcome, results were similar, but there were some differences (Figure S5; Table S9). For example, hospital beds (1.07 [1.01, 1.11]) and mobility (1.08 [1.04, 1.11]) were each associated with larger OE ratios for surgical procedures but not for inpatient hospitalizations. These results illustrate that each five beds per capita increase in hospital beds was associated with a 7.40% (0.61, 15.7%) increase in OE ratios for surgical procedures holding country income, healthcare expenditures, PHSMs, and COVID‐19 deaths constant. Conversely, COVID‐19‐related hospitalizations (*β* = 0.99 [0.99, 0.99]) and income inequality (*β* = 0.96 [0.93, 0.99]) were associated with lower OE ratios for surgical procedures. For COVID‐19‐related hospitalizations, each 10 per 1000 population increase in COVID‐19 hospitalizations was associated with 0.14% (0.02, 0.27%) reduction in OE ratios holding country income, healthcare expenditures, and PHSM constant.

## 4 DISCUSSION

This is the largest study to date to comprehensively investigate disruptions in non‐COVID hospitalizations and ambulatory care services throughout the first 2 years of the pandemic across 32 of the worlds' largest economies. Among the included countries, we observed that the global COVID‐19 pandemic was associated with meaningful health system disruptions for non‐COVID conditions; approximately 16 million missed hospitalizations in 2020 and an additional 15 million missed in 2021. Among the 15 surgical procedures evaluated in this study, we found 2.8 million missed surgical procedures in 2020 but some recovery in services during 2021, with 1.3 million missed. While elective surgeries contributed a majority to these declines, there were considerable numbers of missed non‐elective and heart disease‐related surgeries. We also observed considerable variation in levels of missed hospitalizations and surgeries across the included countries. Several health system factors including total healthcare workforce per capita, insurance coverage, and hospital beds were associated with fewer disruptions. Conversely, we found that more stringent COVID‐19 mitigation strategies and excess COVID‐19 deaths were associated with greater disruptions. The association between both stringent mitigation strategies and excess COVID‐19 deaths may be linked; countries experiencing more COVID‐19 burdens may have had to pursue more stringent measures in response to rising deaths.

Similar to other studies,^28^, ^29^, ^30^ all countries reported consistent declines in non‐COVID respiratory and infectious disease hospitalizations. We observed a 30% drop in respiratory conditions among all included countries combined, while asthma and COPD were avoidable hospitalizations with the sharpest declines. COVID‐19 mitigation measures may have played an important role in these declines as stay‐at‐home policies, mask wearing, and decreases in social mixing may have led to reductions in respiratory virus circulations. This may have subsequently led to fewer hospitalizations attributable to respiratory pathogens and assisted in fewer chronic lung disease exacerbations as a wide majority of chronic lung disease exacerbations are a result of respiratory viral infections.^31^

Although respiratory and infectious disease hospitalizations experienced the sharpest relative declines, these were two of three diagnostic categories that had greater than expected average hospital length of stays (LOS) during the pandemic. On average, hospitalizations were 1.5 days longer for these conditions than expected in the absence of the pandemic, which may suggest that people hospitalized for these conditions may have experienced more severe disease. The other diagnostic category that had longer than expected average LOS was mental and behavioral disorders. LOS for these conditions was on average 5 days longer than expected, potentially due to the adverse effects of the pandemic on mental health outcomes.^32^

The second largest contributor to the decline in hospitalizations were diseases of the circulatory system with a combined 3 million missed hospitalizations in 2020–2021. These declines may be owing to a combination of factors. One potential factor is avoidance of medical care as survey data early in the pandemic suggested that one‐third of participants would stay at home if they thought that they were experiencing a heart attack or stroke due to fear of going to a hospital.^33^ Considering that cardiovascular disease is the leading cause of death in many of the included countries,^34^ the avoidance of care for these conditions may lead to emergency hospital visits and potential increased mortality. Other factors for these declines may potentially be owing to individuals who may need a cardiovascular hospitalization in the future may be dying from COVID‐19 considering similar risk factors, avoiding health care due to loss of employment or remuneration, barriers imposed by COVID‐19 lockdowns, and prioritization of resources and staff for COVID‐19.

Furthermore, we observed marked variability across countries in regard to missed hospitalizations. Mexico, Lithuania, Poland, and Hungary consistently had declines greater than 25%, whereas Switzerland, Sweden, and Norway, and New Zealand had declines of 8% and lower for all‐cause hospitalizations in 2020. By 2021, Switzerland, Sweden, Norway, and Israel had achieved hospitalization rates very close to pre‐pandemic levels while 11 of 27 countries continued to have 15% or lower all‐cause hospitalization rates than expected.

We found several health system‐specific factors that help explain the observed cross‐country variability in missed hospitalizations. Insurance coverage was associated with fewer missed hospitalizations. Countries with a larger fraction of the population insurance may have had a healthier population going into the pandemic,^35^, ^36^ and health insurance may have helped mitigate avoidance in care due to loss wages from the pandemic. Although this could also be related to other factors such as income, which may influence both underlying health and insurance status. In addition, larger numbers of healthcare workers per capita were associated with fewer missed hospitalizations. A larger healthcare workforce may have enabled countries to sustain surges in care resulting from COVID‐19, were less likely to be impacted by staff shortages, and had sufficient staff to maintain ongoing care needs. Interestingly, we also found that hospital beds per capita were associated with fewer disruptions in surgical procedures. Previous work has shown that extra hospital beds enabled health systems to stay below capacity thresholds during COVID‐19 surges while simultaneously continuing other health services uninterrupted and avoiding long waiting lists.^18^ Other studies have also highlighted that the greater number of pre‐pandemic hospital beds was associated with less COVID‐19 mortality^37^, ^38^ and excess mortality associated with the pandemic.^39^ While not a health system factor, we did find that countries with greater income inequality had greater disruptions in surgical procedures. This is consistent with previous research that has reported greater disruptions in health services among disadvantaged groups,^40^ highlighting that resilience policies may need to target groups in the greatest needs of care.

For COVID‐19 response strategies, we observed that countries with more stringent PHSMs, including stay‐at‐home orders, school closures, and workplace closures, had more missed hospitalizations in 2020. This negative association may have appeared because these measures may have led to greater declines in hospitalizations for respiratory conditions and injuries. However, these mitigation measures may have also created substantial barriers to care as restrictions on internal movement may have prevented people from traveling and seeking health services. Simultaneously, stringent public health measures may have adversely impacted peoples' socioeconomic status through potential loss wages and therefore potentially further limit access to care. In addition, the higher levels of stringent measures may be a marker of high levels of COVID‐19 burden causing surges in hospitalizations. This may subsequently lead to cancellations of hospital services or behavioral changes related to fear of COVID‐19 or of further overwhelming health services. The observed associations of COVID‐19 mortality and COVID‐19 hospitalizations on disruptions to care may be another indicator of excess hospital capacity leading to disruptions.

Interestingly, we found that excess mortality associated with the COVID‐19 pandemic was persistently associated with greater missed hospitalizations. Greater excess mortality may also be a marker for higher levels of deaths due to COVID‐19 causing disruptions to health systems. Alternatively, the missed hospitalizations may also be contributing to the observed excess deaths. Indeed, when subtracting out deaths due to COVID‐19 in the excess mortality calculations, greater missed hospitalizations were associated with greater excess all‐cause and avoidable deaths during the pandemic. This is in alignment with other studies linking health service disruptions to thousands of additional excess deaths associated with the pandemic.^41^, ^42^, ^43^

Our analysis should be interpreted in the context of following limitations. First, although we were able to include many countries into our analysis, our results cannot be generalized to other regions or countries. Second, there were slight heterogeneity in definitions and data coverage (e.g., a couple of countries' inpatient data only included hospitalizations from acute care or activity in publicly funded hospitals) across health systems (Tables S1 and S2). Third, our computation of expected hospitalizations assumed that country‐specific historical trends in hospitalizations would continue to have had the pandemic not occurred. In addition, our ARIMA modeling framework is a univariate model and we therefore could not take into account additional factors that may improve predictions, and ARIMA has been shown to be most optimal for time trends with consistent patterns. However, ARIMA models are preferable in short‐term forecasting (this study generates short‐term predictions as we forecast only two‐time steps), and our automated approach allowed us to find the best prediction model. Fourth, there is potential measurement error in some of the included PHSM predictors, as individual metropolitan areas may have deviated from national‐level policies. However, the scope and intensity of PHSM are considered in measurement. Fifth, there may be differences in coding of conditions across countries. Finally, our assessment of country‐level factors was limited by small sample sizes and the descriptive ecologic nature of the data. This prevents our study from making causal connections, and therefore, additional studies are needed to quantify determinants of resilience leveraging longitudinal sub‐national and national data from a wide‐array of settings. Despite these limitations, this analysis provides a comprehensive map of pandemic‐associated disruptions in hospital‐based care across diverse countries and medical conditions.

## 5 CONCLUSIONS

With the World Health Organization declaring an end to COVID‐19 as a global health emergency almost more than a year ago, this provides opportunities to learn and adapt to improve health system resilience. While we observed that all countries experienced disruptions to hospitalizations and surgical procedures with a total of 30 million missed hospitalizations and 4 million missed surgical procedures in 2020–2021, this study highlighted marked cross‐country heterogeneity in disruptions. In particular, countries with greater baseline health system inputs such as healthcare workers, hospital beds, and insurance coverage had disproportionately lower disruptions in care. These findings provide opportunities to learn from health system gaps to improve resilience and avert preventable morbidity and mortality. Considering potential for future epidemics and other major shocks, there is an urgent need to design more resilient health systems ready to address crises while maintaining ongoing care needs. Policy discussions should also consider balancing any potential tradeoffs between health system efficiency and resiliency considering that countries with greater health system inputs and more capacity were more resilient.

## FUNDING INFORMATION

No funding was received to conduct this study.

## CONFLICT OF INTEREST STATEMENT

The authors declare no potential conflicts of interest.

## Supporting information


**Data S1.** Supporting information.

## Data Availability

The data that support the findings of this study are available in the OECD Data Explorer at https://data-explorer.oecd.org/.
