# Supplementary material for: Health system resilience during the COVID‐19 pandemic: A comparative analysis of disruptions in care from 32 countries
Source: Health Serv Res. 2024 Sep 18;59(6):e14382. doi: 10.1111/1475-6773.14382 (PMC11622287; doi:10.1111/1475-6773.14382)
Supplement: Supplementary file 1 — Data S1. Supporting information. [file HESR-59-0-s001.pdf]

Table of Contents

Supplemental Figures **2**

Figure S1. Observed-to-expected (OE) ratios for inpatient hospitalizations by diagnostic categories in (A) 2020 and (B) 2021 [color gradient version of main text Figure 2] 2

Figure S2. Observed-to-expected (OE) ratios of average length of stay by diagnostic categories in (A) 2020 and (B) 2021 4

Figure S3. Observed-to-expected (OE) ratios of avoidable hospitalization age-sex-standardized rates in (A) 2020 and (B) 2021 6

Figure S4. Observed-to-expected (OE) ratios for surgeries by procedure in (A) 2020 and (B) 2021 [color gradient version of main text Figure 3] 8

Figure S5. Associations between health system characteristics, socio-demographic factors, public health and social measures, and COVID-19 burden on observed-to-expected (OE) ratios of surgical procedures 10

Figure S6. Associations between health system characteristics, socio-demographic factors, public health and social measures, and COVID-19 severity on observed-to-expected (OE) ratios of inpatient hospitalizations from univariate mixed regressions 12

Figure S7. Associations between health system characteristics, socio-demographic factors, public health and social measures, and COVID-19 severity on observed-to-expected (OE) ratios of inpatient hospitalizations from univariate mixed regressions 13

Supplemental Tables **14**

Table S1. Description of data sources for inpatient hospitalizations by country 14

Table S2. Descriptions of data sources for surgical procedures by country 16

Table S3. Included surgical procedures by urgency 18

Table S4. Description of country-level characteristics used as covariates 19

Table S5. Expected and observed inpatient hospitalization and average length of stay by country, 2020-2021 21

Table S6. Expected and observed avoidable hospital admissions age-sex-standardized rates by country and sex, 2020-2021 87

Table S7. Expected and observed surgical procedures by country, 2020-2021 110

Table S8. Unadjusted and adjusted coefficients from mixed linear models quantifying the associations between health system characteristics, socio-demographic factors, public health and social measures, and COVID-19 severity on observed-to-expected (OE) ratios of inpatient hospitalizations 155

Table S9. Unadjusted and adjusted coefficients from mixed linear models quantifying the associations between health system characteristics, socio-demographic factors, public health and social measures, and COVID-19 severity on observed-to-expected (OE) ratios of surgical procedures 157

Figure S1. Observed-to-expected (OE) ratios for inpatient hospitalizations by diagnostic categories in (A) 2020 and (B) 2021 [color gradient version of main text Figure 2]

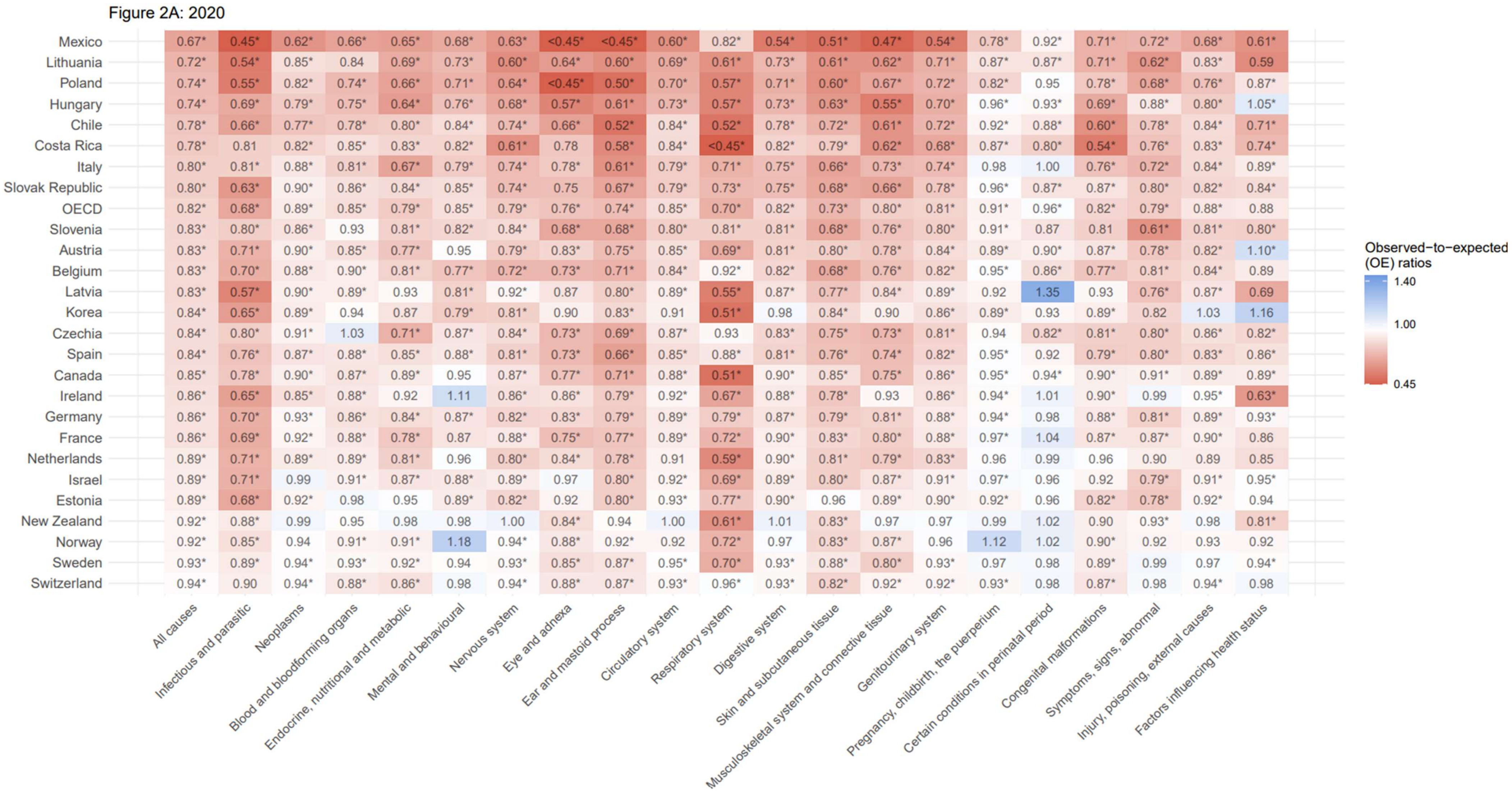

Figure 2B: 2021

	All causes	Infectious and parasitic	Neoplasms	Blood and bloodforming organs	Endocrine, nutritional and metabolic	Mental and behavioural	Nervous system	Eye and adnexa	Ear and mastoid process	Circulatory system	Respiratory system	Digestive system	Skin and subcutaneous tissue	Musculoskeletal system and connective tissue	Genitourinary system	Pregnancy, childbirth, the puerperium	Certain conditions in perinatal period	Congenital malformations	Symptoms, signs, abnormal	Injury, poisoning, external causes	Factors influencing health status
Mexico	0.72*	<0.45*	0.75*	0.72*	0.70*	0.69*	0.72*	<0.45*	0.46*	0.70*	0.70*	0.69*	0.57*	0.67*	0.64*	0.78*	0.95	0.82*	0.79	0.80*	0.78*
Lithuania	0.74*	0.51*	0.85*	0.89	0.80*	0.73*	0.55*	0.72*	0.57*	0.63*	0.82*	0.77*	0.58*	0.64*	0.71*	0.87*	0.87*	0.81*	0.62	0.83*	0.61
Poland	0.82*	0.66*	0.92	0.99	0.75*	0.79*	0.70*	<0.45*	0.64*	0.77*	0.67*	0.82*	0.67*	0.78*	0.84*	0.81*	0.98	0.97	0.83*	0.87*	0.84*
Hungary	0.59*	0.56*	0.63*	0.64*	0.54*	0.63*	0.54*	<0.45*	<0.45*	0.59*	<0.45*	0.58*	0.45*	<0.45*	0.54*	0.85*	0.86*	0.63*	0.66*	0.67*	0.94*
Chile	0.84*	0.62*	0.87*	0.75*	0.94	0.93	0.83*	0.78	0.56*	0.92*	0.57*	0.90*	0.67*	0.73*	0.82*	0.86*	0.79*	0.74*	0.78*	0.88*	0.87*
Costa Rica	0.78*	0.64	0.82*	0.80*	0.83*	0.95	0.54*	0.56*	0.57*	0.89*	<0.45*	0.83*	0.80*	0.61*	0.70*	0.83*	0.69*	0.48*	0.73*	0.83*	0.65*
Italy	0.85*	0.88*	0.94*	0.84*	0.77*	0.86*	0.81*	0.87*	0.71*	0.85*	0.68*	0.83*	0.70*	0.87*	0.81*	1.00	1.17*	0.92*	0.75*	0.87*	0.94*
Slovak Republic	0.73*	0.61*	0.84*	0.76*	0.79*	0.84*	0.62*	0.74	0.55*	0.72*	1.08*	0.70*	0.56*	<0.45*	0.70*	0.93	0.82*	0.88*	0.74*	0.74*	<0.45*
OECD	0.84*	0.70*	0.91*	0.88*	0.83*	0.88*	0.80*	0.81*	0.74*	0.87*	0.68*	0.86*	0.71*	0.82*	0.85*	0.92*	1.00	0.90*	0.82*	0.89*	0.90
Slovenia	0.87*	0.96	0.87*	1.01	0.82*	0.90*	0.81*	0.52*	0.69*	0.82*	0.98	0.80*	0.63*	0.73*	0.83*	0.93*	0.84	0.92	0.68*	0.78*	0.95
Austria	0.87*	0.73*	0.92*	0.91*	0.85*	1.12	0.86*	0.97	0.80*	0.91*	0.63*	0.83*	0.81*	0.84*	0.88*	0.91*	0.96	0.98	0.80*	0.82*	0.88*
Belgium	0.90*	0.78*	0.93*	0.95*	0.98	0.82*	0.82*	0.81*	0.83*	0.91*	0.97	0.90*	0.74*	0.88*	0.91*	1.01	0.99	0.86*	0.88*	0.88*	0.93
Latvia	0.85*	0.50*	0.87*	0.82*	1.14	0.97	0.96	>1.40	0.76*	0.83*	<0.45*	0.83*	0.70*	1.36*	0.85*	0.92	>1.40	1.14*	1.03	0.90*	
Korea	0.81*	0.64*	0.89	0.92	0.87	0.73*	0.75*	0.94	0.74*	0.94	<0.45*	0.98	0.75*	0.84	0.81*	0.87	0.93	0.89	0.93	1.02	1.29
Czechia	0.84*	0.80*	1.09*	1.02	0.66*	0.88*	0.76*	0.65*	0.61*	0.88*	1.28*	0.82*	0.67*	0.67*	0.79*	0.95	0.71*	0.85*	0.78*	0.84*	0.72*
Spain	0.87*	0.76*	0.92*	0.96*	1.00	0.96	0.88	0.83*	0.74*	0.94*	0.59*	0.91*	0.80*	0.85*	0.91*	0.97	0.94	0.90*	0.85*	0.91*	0.94
Canada	0.88*	0.83*	0.92*	0.93*	0.90*	0.95	0.89*	0.83*	0.76*	0.91*	0.62*	0.93*	0.87*	0.74*	0.89*	0.99	0.98	0.93*	0.96	0.93*	0.94*
Ireland	0.89*	0.70*	0.89*	0.93	0.98	1.22*	0.89	0.93	0.86	0.96*	0.70*	0.92*	0.77*	1.00	0.89*	1.02	1.00	0.99	1.04	1.00	0.63*
Germany	0.86*	0.71*	0.92*	0.85*	0.85*	0.91*	0.82*	0.83*	0.76*	0.88*	0.80*	0.86*	0.78*	0.79*	0.86*	0.95	0.99	0.88*	0.82*	0.88*	0.95
France	0.88	0.77*	0.96*	0.86*	0.84*	0.89	0.89*	0.83*	0.83*	0.92*	0.73*	0.93*	0.83*	0.87*	0.92*	1.00	1.14	0.98	0.87*	0.95*	0.87
Netherlands	0.92	0.76*	0.87*	0.91	0.83*	1.09	0.80*	0.82	0.74*	0.91	0.56*	0.92*	0.78*	0.78*	0.80*	1.02	1.06	0.97	0.96	0.90	0.82
Israel	0.96*	0.86*	1.05	1.00	1.07	0.96*	0.99	1.00	0.89*	0.95	0.78*	0.96*	0.87*	0.93	0.94*	0.98	1.02	0.97	0.93	0.97	0.99
Estonia	0.90*	0.69*	0.86*	0.83*	0.86*	0.90*	0.84*	0.91	0.87	0.88*	1.20*	0.87*	0.94	0.80*	0.88*	0.90*	1.00	0.83*	0.76*	0.89*	0.87
Norway	0.97*	0.91	0.97	0.97	0.93	1.20	0.97	0.93	0.98	0.95	0.77*	1.02	0.86	0.93	0.97	>1.40	1.13	0.94	0.99	0.98	0.97
Sweden	0.97	0.95*	1.00	1.01	0.99	0.94	0.99	0.88*	0.86	1.01	0.69*	0.96	0.87	0.81*	0.97	1.00	0.99	0.93	1.11*	1.01	0.94*
Switzerland	0.98*	0.94	0.98	0.90	0.91*	1.02	0.98	0.90	0.94	0.96*	0.98	0.97	0.85*	0.97	0.97	0.97	1.05	0.94*	1.04	0.96	1.00

Observed−to−expected (OE) ratios: 1.40 / 1.00 / 0.45

Figure S2. Observed-to-expected (OE) ratios of average length of stay by diagnostic categories in (A) 2020 and (B) 2021

(A)

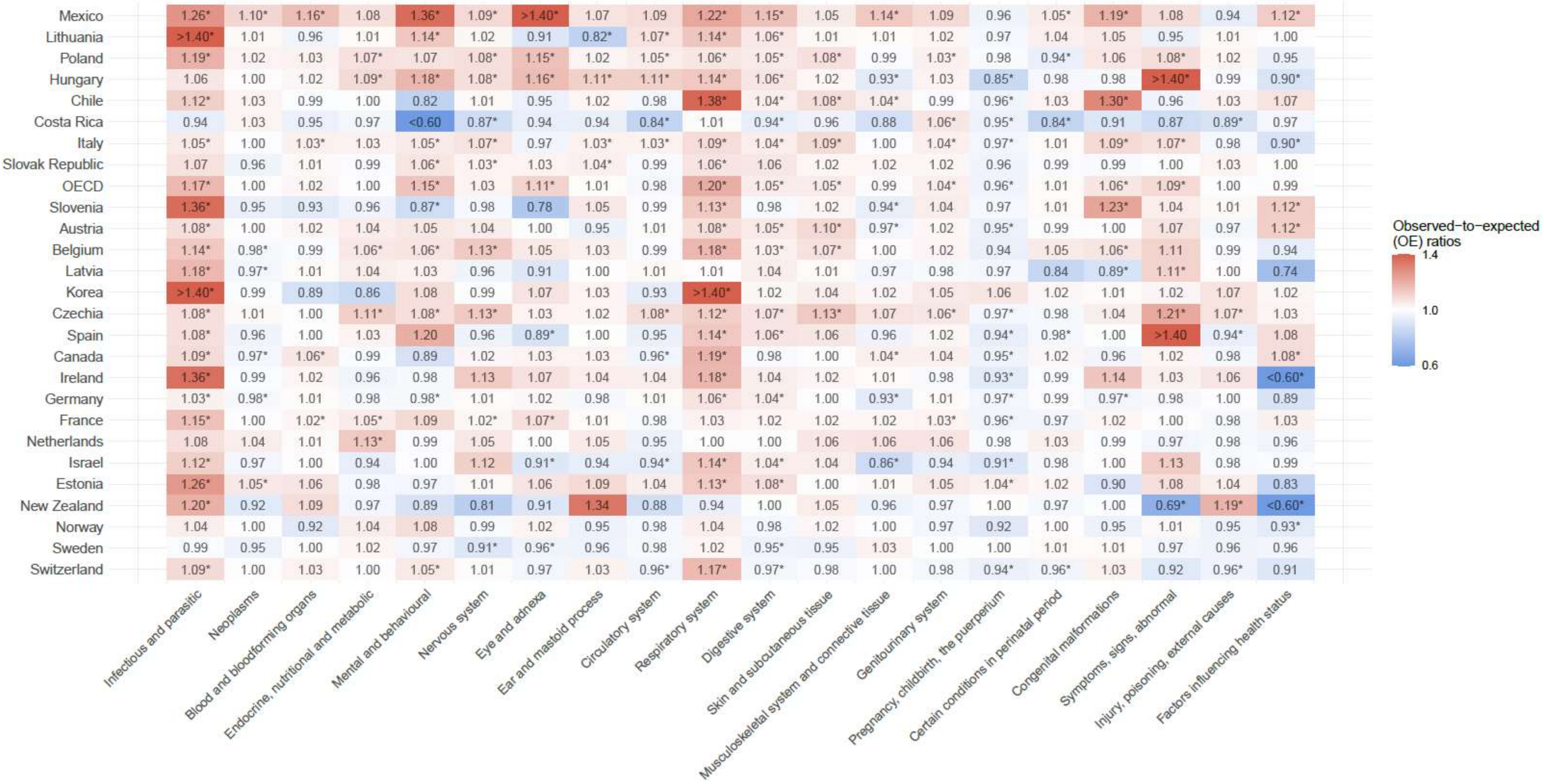

(B)

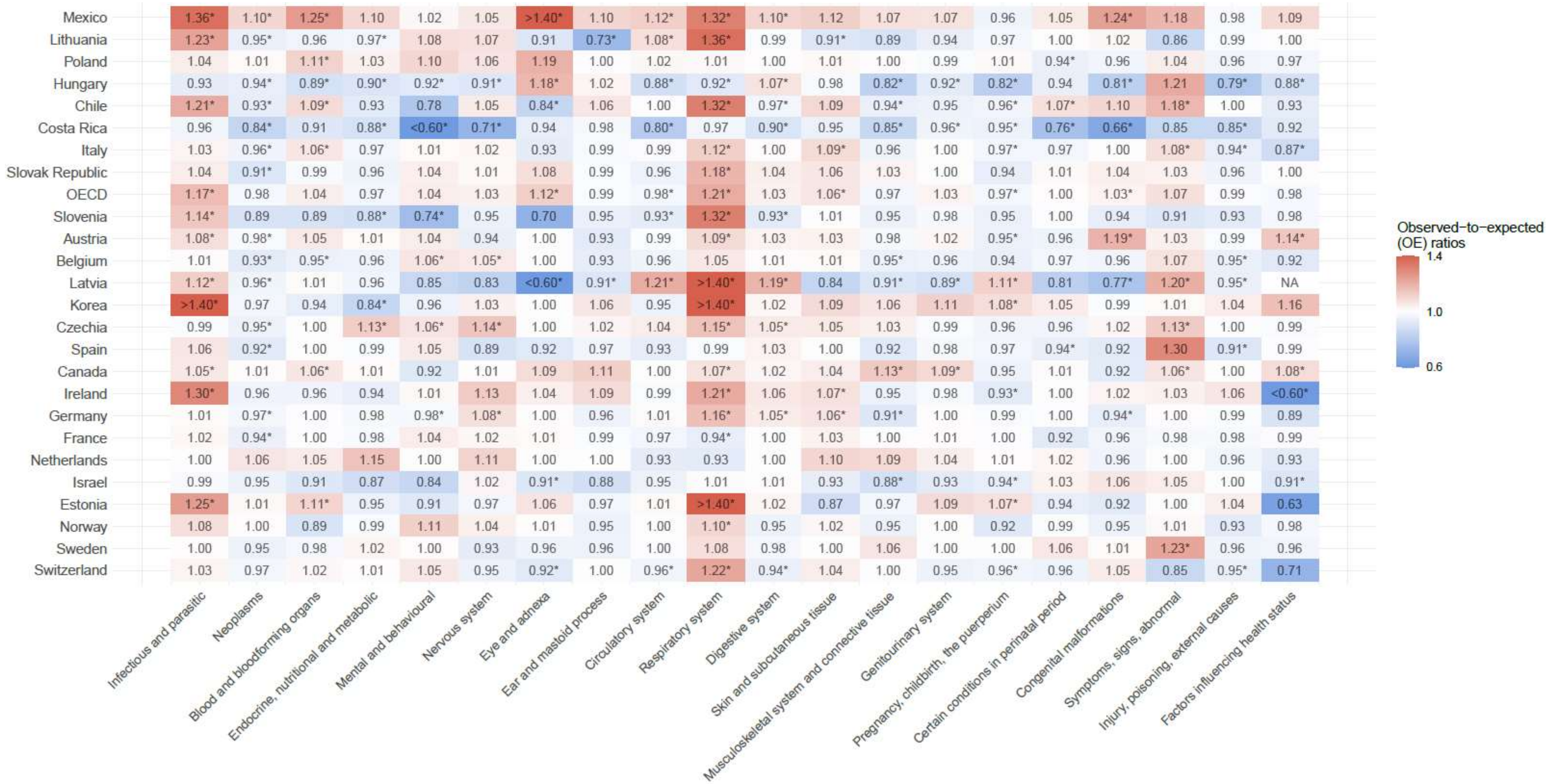
Mexico 1.36* 1.10* 1.25* 1.10 1.02 1.05 >1.40* 1.10 1.12* 1.32* 1.10* 1.12 1.07 1.07 0.96 1.05 1.24* 1.18 0.98 1.09
Lithuania 1.23* 0.95* 0.96 0.97* 1.08 1.07 0.91 0.73* 1.08* 1.36* 0.99 0.91* 0.89 0.94 0.97 1.00 1.02 0.86 0.99 1.00
Poland 1.04 1.01 1.11* 1.03 1.10 1.06 1.19 1.00 1.02 1.01 1.00 1.01 1.00 0.99 1.01 0.94* 0.96 1.04 0.96 0.97
Hungary 0.93 0.94* 0.89* 0.90* 0.92* 0.91* 1.18* 1.02 0.88* 0.92* 1.07* 0.98 0.82* 0.92* 0.82* 0.94 0.81* 1.21 0.79* 0.88*
Chile 1.21* 0.93* 1.09* 0.93 0.78 1.05 0.84* 1.06 1.00 1.32* 0.97* 1.09 0.94* 0.95 0.96* 1.07* 1.10 1.18* 1.00 0.93
Costa Rica 0.96 0.84* 0.91 0.88* <0.60* 0.71* 0.94 0.98 0.80* 0.97 0.90* 0.95 0.85* 0.96* 0.95* 0.76* 0.66* 0.85 0.85* 0.92
Italy 1.03 0.96* 1.06* 0.97 1.01 1.02 0.93 0.99 0.99 1.12* 1.00 1.09* 0.96 1.00 0.97* 0.97 1.00 1.08* 0.94* 0.87*
Slovak Republic 1.04 0.91* 0.99 0.96 1.04 1.01 1.08 0.99 0.96 1.18* 1.04 1.06 1.03 1.00 0.94 1.01 1.04 1.03 0.96 1.00
OECD 1.17* 0.98 1.04 0.97 1.04 1.03 1.12* 0.99 0.98* 1.21* 1.03 1.06* 0.97 1.03 0.97* 1.00 1.03* 1.07 0.99 0.98
Slovenia 1.14* 0.89 0.89 0.88* 0.74* 0.95 0.70 0.95 0.93* 1.32* 0.93* 1.01 0.95 0.98 0.95 1.00 0.94 0.91 0.93 0.98
Austria 1.08* 0.98* 1.05 1.01 1.04 0.94 1.00 0.93 0.99 1.09* 1.03 1.03 0.98 1.02 0.95* 0.96 1.19* 1.03 0.99 1.14*
Belgium 1.01 0.93* 0.95* 0.96 1.06* 1.05* 1.00 0.93 0.96 1.05 1.01 1.01 0.95* 0.96 0.94 0.97 0.96 1.07 0.95* 0.92
Latvia 1.12* 0.96* 1.01 0.96 0.85 0.83 <0.60* 0.91* 1.21* >1.40* 1.19* 0.84 0.91* 0.89* 1.11* 0.81 0.77* 1.20* 0.95* NA
Korea >1.40* 0.97 0.94 0.84* 0.96 1.03 1.00 1.06 0.95 >1.40* 1.02 1.09 1.06 1.11 1.08* 1.05 0.99 1.01 1.04 1.16
Czechia 0.99 0.95* 1.00 1.13* 1.06* 1.14* 1.00 1.02 1.04 1.15* 1.05* 1.05 1.03 0.99 0.96 0.96 1.02 1.13* 1.00 0.99
Spain 1.06 0.92* 1.00 0.99 1.05 0.89 0.92 0.97 0.93 0.99 1.03 1.00 0.92 0.98 0.97 0.94* 0.92 1.30 0.91* 0.99
Canada 1.05* 1.01 1.06* 1.01 0.92 1.01 1.09 1.11 1.00 1.07* 1.02 1.04 1.13* 1.09* 0.95 1.01 0.92 1.06* 1.00 1.08*
Ireland 1.30* 0.96 0.96 0.94 1.01 1.13 1.04 1.09 0.99 1.21* 1.06 1.07* 0.95 0.98 0.93* 1.00 1.02 1.03 1.06 <0.60*
Germany 1.01 0.97* 1.00 0.98 0.98* 1.08* 1.00 0.96 1.01 1.16* 1.05* 1.06* 0.91* 1.00 0.99 1.00 0.94* 1.00 0.99 0.89
France 1.02 0.94* 1.00 0.98 1.04 1.02 1.01 0.99 0.97 0.94* 1.00 1.03 1.00 1.01 1.00 0.92 0.96 0.98 0.98 0.99
Netherlands 1.00 1.06 1.05 1.15 1.00 1.11 1.00 1.00 0.93 0.93 1.00 1.10 1.09 1.04 1.01 1.02 0.96 1.00 0.96 0.93
Israel 0.99 0.95 0.91 0.87 0.84 1.02 0.91* 0.88 0.95 1.01 1.01 0.93 0.88* 0.93 0.94* 1.03 1.06 1.05 1.00 0.91*
Estonia 1.25* 1.01 1.11* 0.95 0.91 0.97 1.06 0.97 1.01 >1.40* 1.02 0.87 0.97 1.09 1.07* 0.94 0.92 1.00 1.04 0.63
Norway 1.08 1.00 0.89 0.99 1.11 1.04 1.01 0.95 1.00 1.10* 0.95 1.02 0.95 1.00 0.92 0.99 0.95 1.01 0.93 0.98
Sweden 1.00 0.95 0.98 1.02 1.00 0.93 0.96 0.96 1.00 1.08 0.98 1.00 1.06 1.00 1.00 1.06 1.01 1.23* 0.96 0.96
Switzerland 1.03 0.97 1.02 1.01 1.05 0.95 0.92* 1.00 0.96* 1.22* 0.94* 1.04 1.00 0.95 0.96* 0.96 1.05 0.85 0.95* 0.71
Infectious and parasitic
Neoplasms
Blood and bloodforming organs
Endocrine, nutritional and metabolic
Mental and behavioural
Nervous system
Eye and adnexa
Ear and mastoid process
Circulatory system
Respiratory system
Digestive system
Skin and subcutaneous tissue
Musculoskeletal system and connective tissue
Genitourinary system
Pregnancy, childbirth, the puerperium
Certain conditions in perinatal period
Congenital malformations
Symptoms, signs, abnormal
Injury, poisoning, external causes
Factors influencing health status
Observed-to-expected (OE) ratios
1.4
1.0
0.6

Figure S3. Observed-to-expected (OE) ratios of avoidable hospitalization age-sex-standardized rates in (A) 2020 and (B) 2021

A)

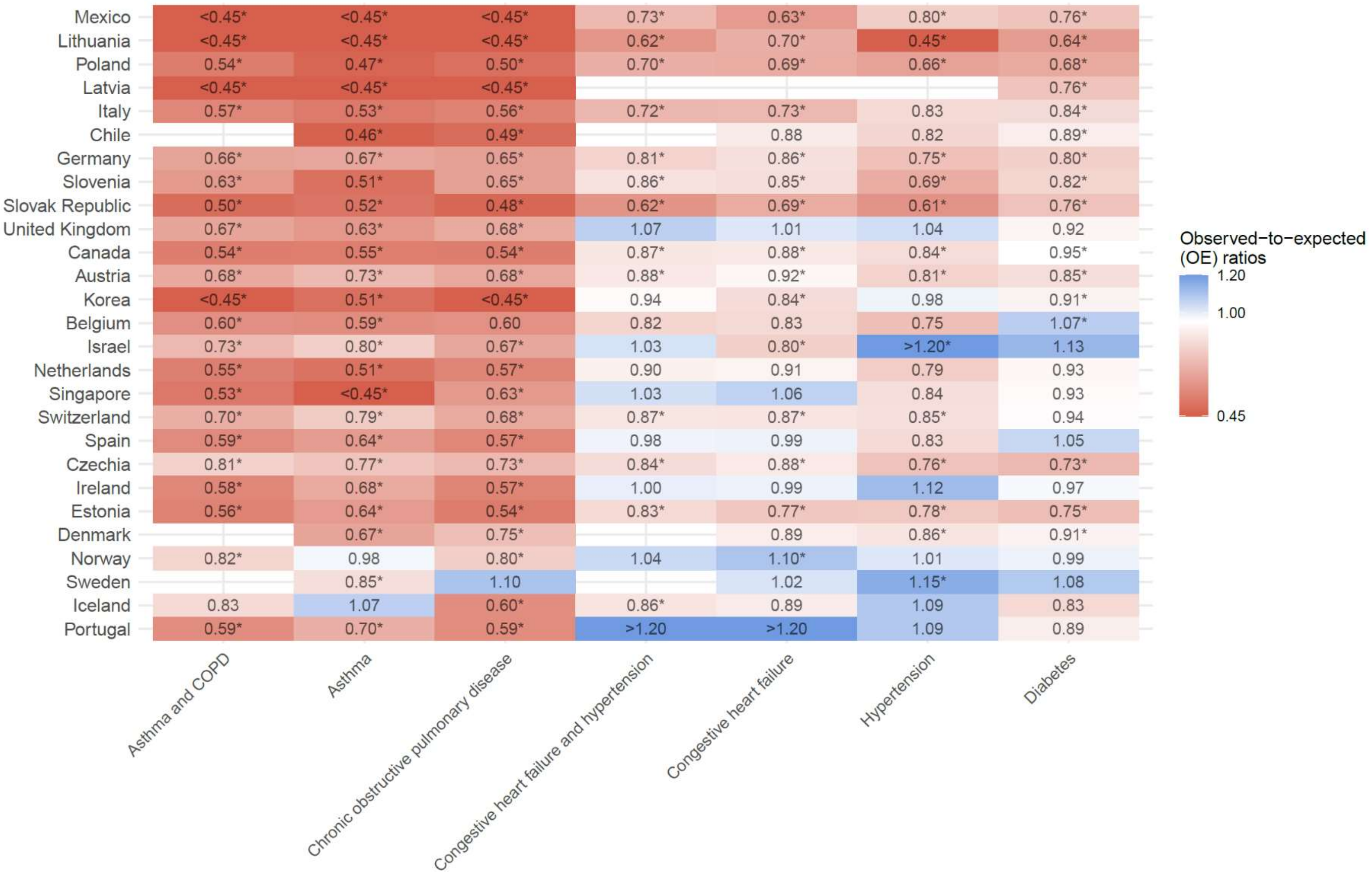

(B)

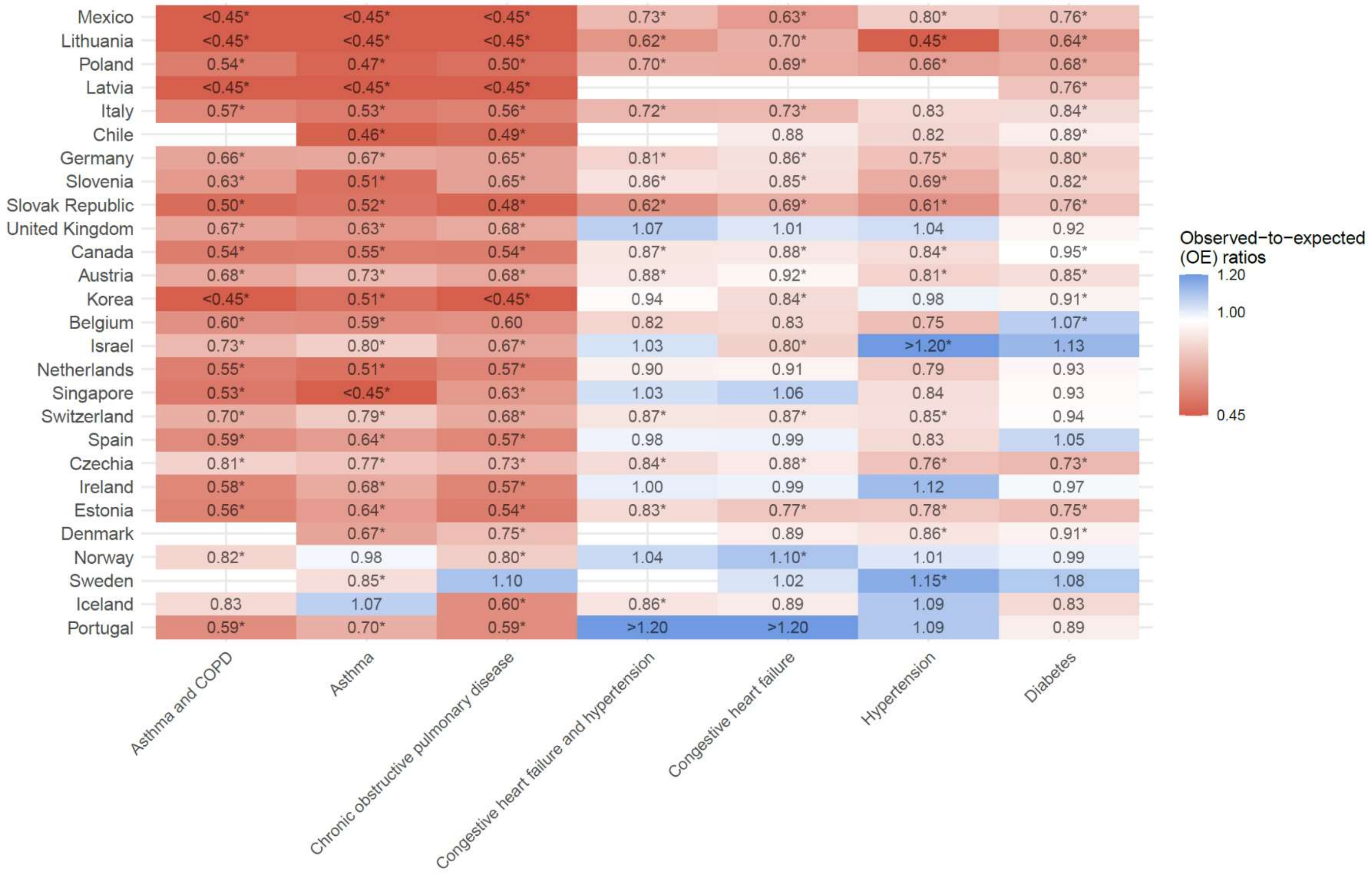

Mexico <0.45* <0.45* <0.45* 0.73* 0.63* 0.80* 0.76*
Lithuania <0.45* <0.45* <0.45* 0.62* 0.70* 0.45* 0.64*
Poland 0.54* 0.47* 0.50* 0.70* 0.69* 0.66* 0.68*
Latvia <0.45* <0.45* <0.45* 0.76*
Italy 0.57* 0.53* 0.56* 0.72* 0.73* 0.83 0.84*
Chile 0.46* 0.49* 0.88 0.82 0.89*
Germany 0.66* 0.67* 0.65* 0.81* 0.86* 0.75* 0.80*
Slovenia 0.63* 0.51* 0.65* 0.86* 0.85* 0.69* 0.82*
Slovak Republic 0.50* 0.52* 0.48* 0.62* 0.69* 0.61* 0.76*
United Kingdom 0.67* 0.63* 0.68* 1.07 1.01 1.04 0.92
Canada 0.54* 0.55* 0.54* 0.87* 0.88* 0.84* 0.95*
Austria 0.68* 0.73* 0.68* 0.88* 0.92* 0.81* 0.85*
Korea <0.45* 0.51* <0.45* 0.94 0.84* 0.98 0.91*
Belgium 0.60* 0.59* 0.60 0.82 0.83 0.75 1.07*
Israel 0.73* 0.80* 0.67* 1.03 0.80* >1.20* 1.13
Netherlands 0.55* 0.51* 0.57* 0.90 0.91 0.79 0.93
Singapore 0.53* <0.45* 0.63* 1.03 1.06 0.84 0.93
Switzerland 0.70* 0.79* 0.68* 0.87* 0.87* 0.85* 0.94
Spain 0.59* 0.64* 0.57* 0.98 0.99 0.83 1.05
Czechia 0.81* 0.77* 0.73* 0.84* 0.88* 0.76* 0.73*
Ireland 0.58* 0.68* 0.57* 1.00 0.99 1.12 0.97
Estonia 0.56* 0.64* 0.54* 0.83* 0.77* 0.78* 0.75*
Denmark 0.67* 0.75* 0.89 0.86* 0.91*
Norway 0.82* 0.98 0.80* 1.04 1.10* 1.01 0.99
Sweden 0.85* 1.10 1.02 1.15* 1.08
Iceland 0.83 1.07 0.60* 0.86* 0.89 1.09 0.83
Portugal 0.59* 0.70* 0.59* >1.20 >1.20 1.09 0.89
Asthma and COPD
Asthma
Chronic obstructive pulmonary disease
Congestive heart failure and hypertension
Congestive heart failure
Hypertension
Diabetes
Observed−to−expected (OE) ratios
1.20
1.00
0.45

Figure S4. Observed-to-expected (OE) ratios for surgeries by procedure in (A) 2020 and (B) 2021 [color gradient version of main text Figure 3]

Figure 3A: 2020

	All surgeries	Non-elective surgeries	Appendectomy	Cholecystectomy	Total mastectomy	Partial excision of mammary gland	Both	Caesarean section	Repair of inguinal hernia	Transluminal coronary angioplasty	Coronary artery bypass graft	Elective surgeries	Hip replacement	Total knee replacement	Cataract surgery	Tonsillectomy	Hysterectomy	Open prostatectomy	Transurethral prostatectomy
Costa Rica	0.63*	0.73*	0.96	0.52*	0.77*	0.73*	0.74*	0.93*	<0.50*	0.95	0.69	0.51*	0.61*	<0.50*	<0.50*	<0.50*	0.72*	0.51*	<0.50*
United Kingdom	0.63*	0.71*	0.87*	0.60*	0.84*	0.74*	0.86*	1.01	<0.50*	0.87*	0.66*	0.51*	0.54*	<0.50*	0.52*	<0.50*	0.63*	0.74*	0.55*
Poland	0.71*	0.76*	0.79*	0.71*	0.95	0.72*	0.87*	0.92	0.71*	0.83*	0.61*	0.62*	0.70*	0.62*	0.58*	<0.50*	0.77*	0.79*	0.67*
Hungary	0.72*	0.75*	0.88*	0.67*	0.90*	0.87*	0.88*	0.99	0.68*	0.85*	0.85*	0.62*	0.69*	0.55*	0.61*	<0.50*	0.75*	0.73*	0.69*
Lithuania	0.73*	0.75*	0.86*	0.71*	0.94	0.72*	0.78*	0.98	0.68*	0.78*	0.69*	0.69*	0.86	0.52*	0.73*	<0.50*	0.75*	0.68*	0.68*
Italy	0.75*	0.80*	0.86*	0.73*	0.91*	0.84*	0.82*	1.01	0.63*	0.84*	0.75*	0.71*	0.82*	0.71*	0.67	0.52*	0.79*	0.80*	0.71*
Slovak Republic	0.77*	0.74*		0.80*	1.00		0.93*	1.00	0.75*		0.91	0.71*	0.83*	0.76*	0.68*		0.88*	0.74*	0.78*
Belgium	0.80*	0.89*	1.00	0.87*	0.88	0.84	0.92*	1.00	0.79*	0.91*	0.85*	0.76*	0.79*	0.65*	0.77*	0.57*	0.78*	0.95	0.80*
Portugal	0.81*	0.84*	0.88*	0.79*	0.92	0.91*	0.82*		0.78*	0.86*	0.93*	0.80*	0.84*	0.77*	0.80*	0.73*	0.80*	0.88*	0.84*
Spain	0.81*	0.83*	0.96	0.84*	0.54*	0.82*	0.86*	0.98	0.78*	0.94	0.84*	0.77*	0.82*	0.64*	0.80*	0.59*	0.85*	0.90*	0.80*
OECD	0.83*	0.88*	0.95*	0.85*	0.90*	0.91*	0.90*	0.98	0.77*	0.88*	0.83*	0.79*	0.83*	0.74*	0.78*	0.65*	0.85*	0.89	0.80*
Canada	0.84*	0.91*	0.98	0.94*	0.88*	0.84*	0.92*	0.98*	0.85*	0.90*	0.83*	0.79*	0.83*	0.71*	0.79*	0.67*	0.85*	0.94	0.82*
France	0.84*	0.95*	1.01	0.91*	0.94*	0.93*	0.91*	1.00	0.84*	0.90*	0.85*	0.80*	0.87*	0.75*	0.78*	0.66*	0.87*	0.93	0.82*
Austria	0.86*	0.90*	0.90*	0.89*	0.88*	0.91*	0.90*	0.98	0.83*	0.91*	0.89*	0.82*	0.89*	0.79*	0.81*	0.69*	0.86*	1.04	0.90*
Slovenia	0.86*	0.87*	0.94*	0.80*	0.98	0.89	0.89*	0.93*	0.78*	0.90	0.84*	0.85*	0.93	0.76*	0.89	0.58*	0.84*	0.56*	0.88
Sweden	0.87*	0.93*	1.04*	0.92*	0.92*	0.95	0.87*	0.97	0.79*	0.88*	0.90*	0.85*	0.82*	0.73*	0.89	0.70*	0.87*	0.82*	0.79*
Czechia	0.88*	0.83*	0.93*	0.80*			0.89*	0.97	0.74*	0.91*	0.86*	0.87*	1.05	0.95	0.86*	0.69*	0.81*	0.69*	0.77*
Luxembourg	0.89*	0.94	0.96	0.90*	0.97	0.97	0.91	0.99	0.95	0.85*	1.13	0.83*	0.88*	0.77*	0.84*	0.64*	0.76*	0.90	
Iceland	0.91	0.91*	0.98	0.82*	0.91	1.01	0.96	1.00		0.77*	0.94	0.91	0.87		0.95		0.79*	<0.50*	0.78
Estonia	0.92	0.91*	0.97	0.87*	0.78*	0.87*	0.96	1.00	0.95	0.96	0.87	0.91	0.99	1.04	0.93	0.81*	0.91	0.98	0.84
Norway	0.92	>1.20	1.06	0.95	0.95	0.93	1.07	0.98	0.90	0.92*	0.95	0.89	0.90*	0.92	0.91	0.80	0.95	0.74	1.01
Finland	0.93*	0.95	1.03	0.94	0.94	0.91	0.99	1.12*	0.86*	0.97	0.96	0.86*	0.89*	0.93	0.86*	0.70*	0.92	0.85	0.91
Germany	0.93*	0.95*	0.97	0.94*	0.97	0.94*	0.91*	1.01	0.84*	0.89*	0.88*	0.94*	0.92*	0.89*	0.96*	0.76*	0.90*	0.92*	0.88*
Korea	0.94	1.00	0.99	0.96	1.05	0.94	0.95	0.96	0.87*	0.94*	1.00	0.93*	1.03	0.87*	0.96	0.74*	0.97	0.83*	0.95
Denmark	0.94*	0.96	1.00	0.94	0.94	0.93	0.97	1.01	0.94	0.94	1.13*	0.93*	0.95*	1.12	0.91*	0.94		1.03	0.92
Switzerland	0.95*	0.96*	1.02	0.95*	0.95	0.94*	0.94*	1.00	0.89*	0.89*	0.88*	0.95	0.99	1.04	0.93	0.72*	0.87*	1.03	1.00
New Zealand	0.97	1.00	0.99	1.03	0.95	0.97	0.97	0.99	0.88	0.93	0.96	0.93*	0.92*	0.94	0.91*	0.73*	1.01	>1.20*	
Israel	0.98	1.01	1.05	1.01	0.97	1.00	0.95*	0.99	0.90*	0.97	0.90	0.99	0.92*	0.85*	1.01	0.85*	0.99	1.03	1.06

Observed-to-expected (OE) ratios
1.2
1.0
0.5

Figure 3B: 2021

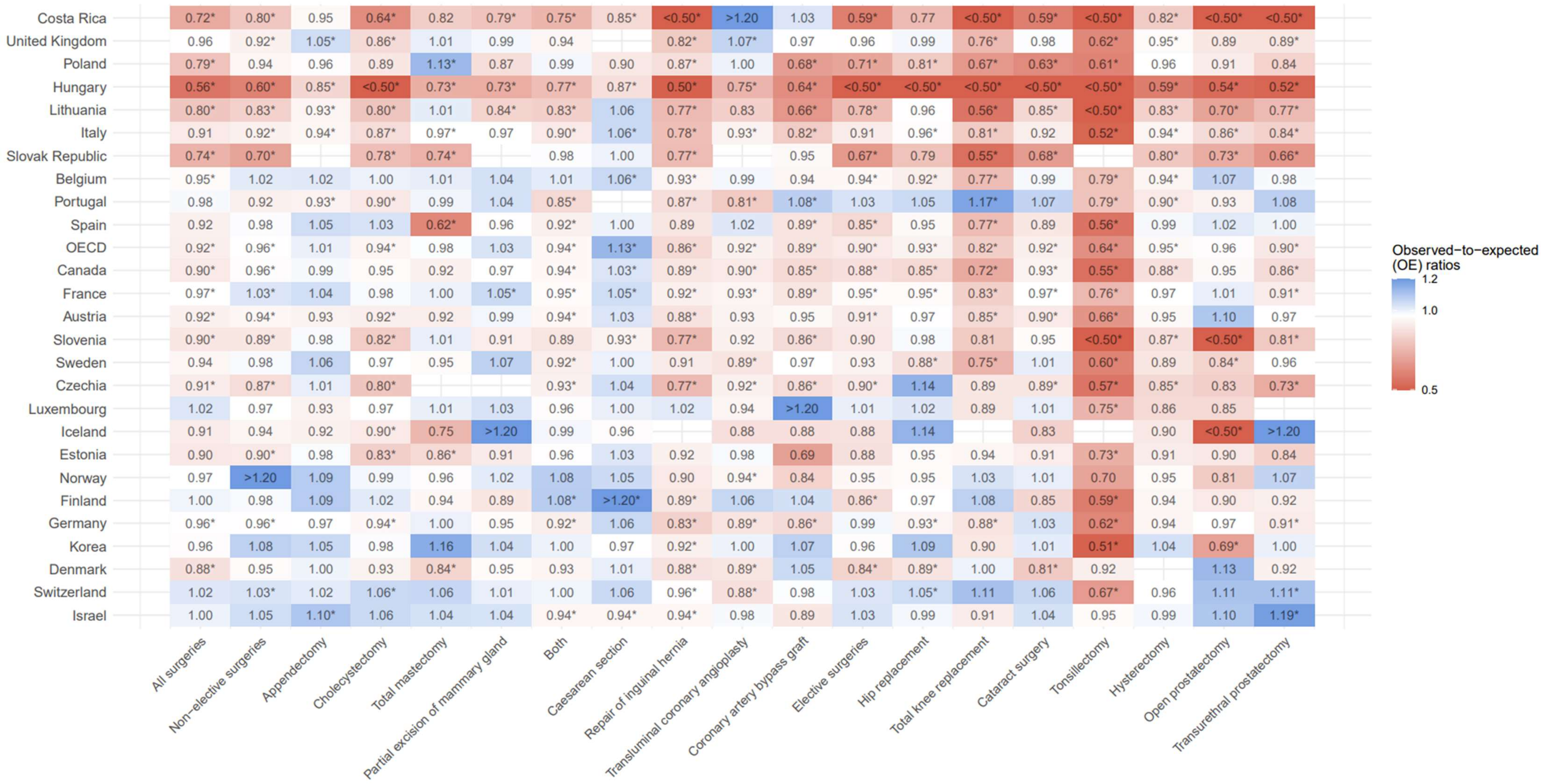

Figure S5. Associations between health system characteristics, socio-demographic factors, public health and social measures, and COVID-19 severity on observed-to-expected (OE) ratios of surgical procedures.

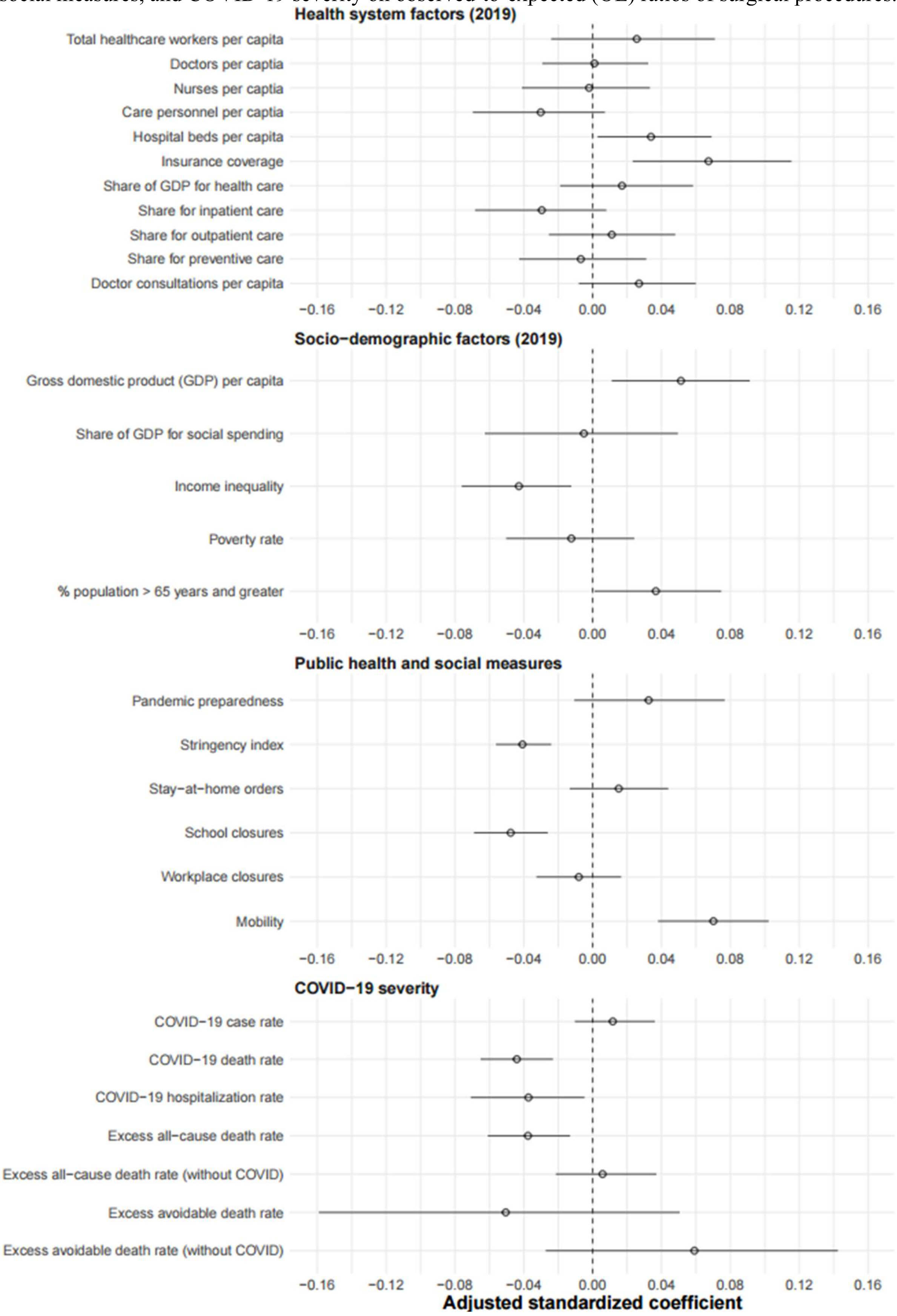

Caption: Coefficients are from mixed linear regressions with random intercept terms for country. Separate models were conducted for each variable of interest. In each regression, log-transformed income (pre-pandemic gross domestic product per capita), pre-pandemic health expenditure, the stringency index, and COVID-19 death rate were included as covariates. Owing to high correlation between variables, there some differences in included covariates in each regression. In the healthcare workers model, gross domestic product (GDP) per capita was removed due to very high correlation between GDP and healthcare workers (r=0.87). In COVID-19 severity models, COVID-19 deaths covariate was removed. In individual public health and social measure models (stay-at-home, school closures, workplace closures), the stringency index covariate was removed. The circles represent the standardized coefficients from the multivariate mixed linear regressions while the lines are the corresponding 95% confidence intervals.

Figure S6. Associations between health system characteristics, socio-demographic factors, public health and social measures, and COVID-19 severity on observed-to-expected (OE) ratios of inpatient hospitalizations from univariate mixed regressions

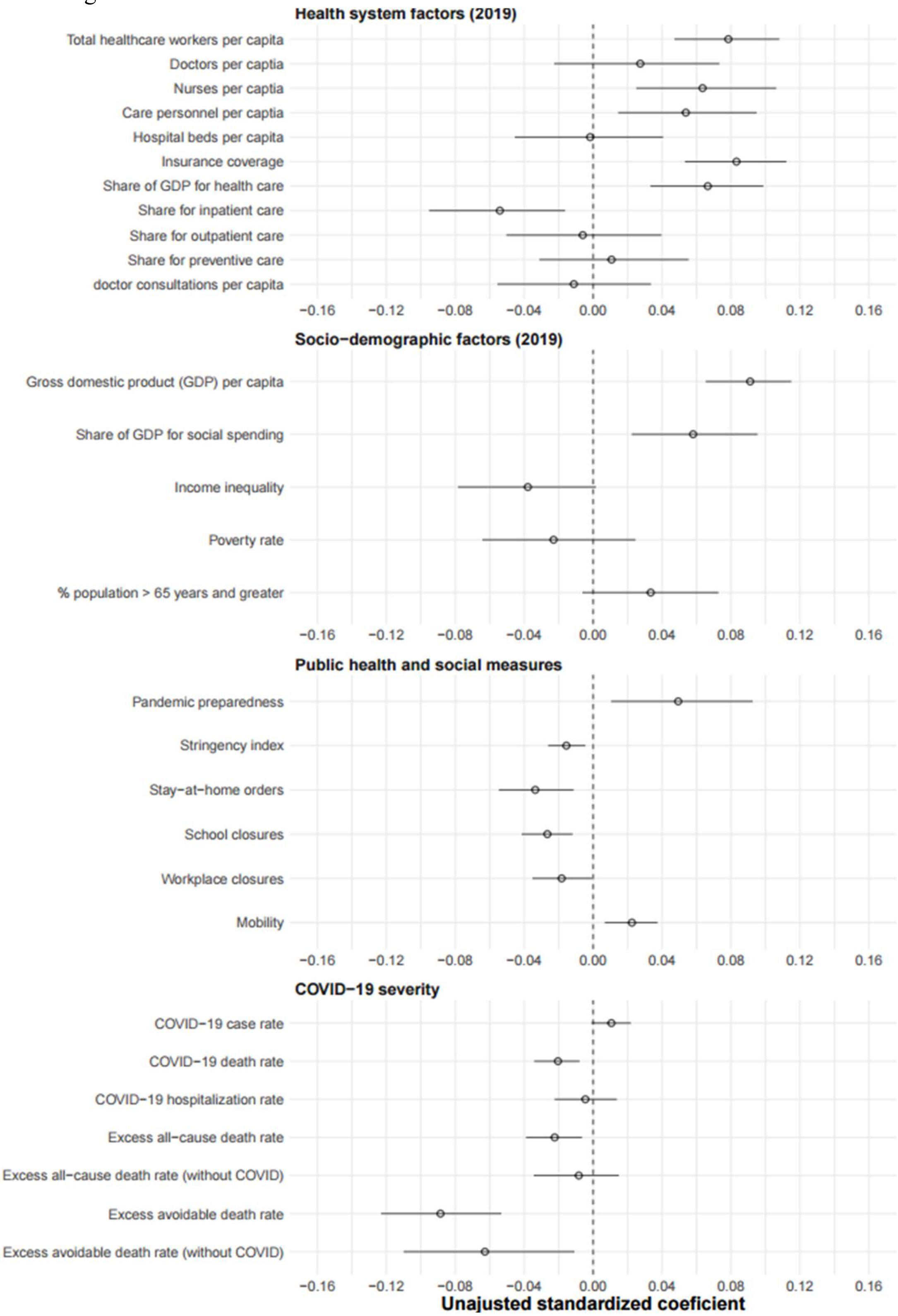

Caption: Coefficients are from mixed linear regressions with random intercept terms for country. Separate models were conducted for each variable of interest. No additional covariates were included in each regression. The circles represent the standardized coefficients from the mixed linear regressions while the lines are the corresponding 95% confidence intervals.

Figure S7. Associations between health system characteristics, socio-demographic factors, public health and social measures, and COVID-19 severity on observed-to-expected (OE) ratios of surgical procedures from univariate mixed regressions

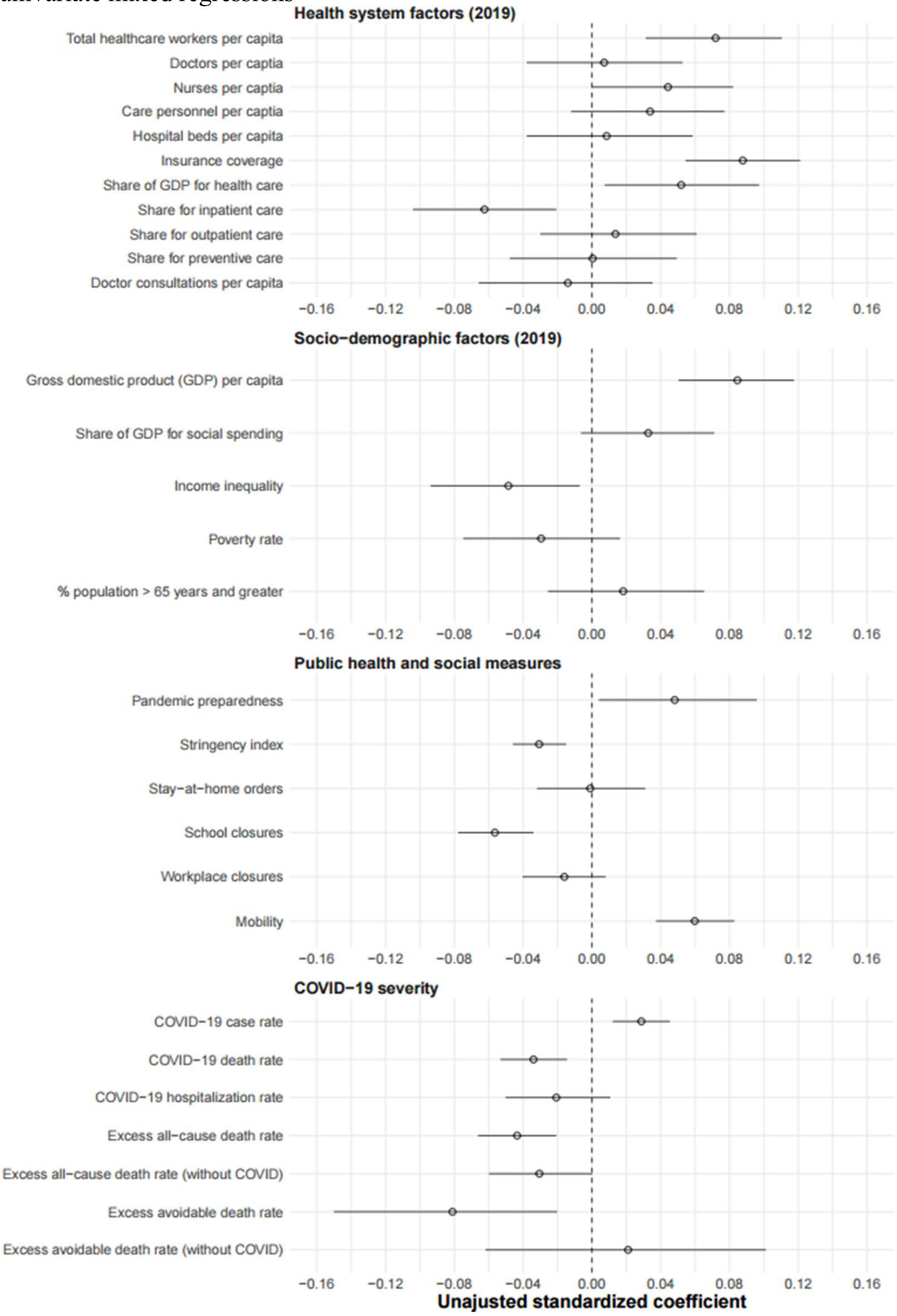

Caption: Coefficients are from mixed linear regressions with random intercept terms for country. Separate models were conducted for each variable of interest. No additional covariates were included in each regression. The circles represent the standardized coefficients from the mixed linear regressions while the lines are the corresponding 95% confidence intervals.

Table S1. Description of data sources for inpatient hospitalizations by country.

Country	Source	Reference year	Relevant notes on data coverage
Austria	Statistics Austria, Hospital Discharge Statistics	Calendar year (1st January to 31st December)	
Belgium	The Federal Public Service of Health, Food Chain Safety and Environment, Directorate 1 - Minimal Clinical Data	Calendar year (1st January to 31st December)	
Canada	Canadian Institute for Health Information, Discharge Abstract Database and Hospital Morbidity Database	Fiscal year (1st April to 31st March)	Data are for acute care hospitals only
Chile	Ministry of Health (MINSAL), Department of Health Statistics and Information (DEIS)	Calendar year (1st January to 31st December)	
Costa Rica	Área de Estadística en Salud, Caja Costarricense de Seguro Social	Calendar year (1st January to 31st December)	Data only include hospitalizations from public facilities belonging to the Social Insurance (covers 95% of the population)
Czech Republic (Czechia)	Institute of Health Information and Statistics of the Czech Republic, National Registry of Hospitalised Patients	Calendar year (1st January to 31st December)	
Estonia	Ministry of Social Affairs, Department of Health Information and Analysis	Calendar year (1st January to 31st December)	
France	Programme de médicalisation des systèmes d'information (PMSI)	Calendar year (1st January to 31st December)	Covers residents of Metropolitan France and overseas departments
Germany	Federal Statistical Office, Hospital statistics 2021	Calendar year (1st January to 31st December)	
Hungary	National Healthcare Service Center; National Directorate General for Hospitals	Calendar year (1st January to 31st December)	
Ireland	Hospital In-Patient Enquiry (HIPE)	Calendar year (1st January to 31st December)	Data only include hospitalizations from publicly funded hospitals
Israel	National Hospital Discharge Database, Health Information Division in the Ministry of Health	Calendar year (1st January to 31st December)	
Italy	General Directorate of Health Planning, National Hospital Discharge Data Base (NHDDB)	Calendar year (1st January to 31st December)	
South Korea	Ministry of Health and Welfare, Health Insurance Review & Assessment Service, Statistics of Healthcare Utilisation	Calendar year (1st January to 31st December)	
Latvia	National Health Service	Calendar year (1st January to 31st December)	
Lithuania	Health Information Centre of Institute of Hygiene, Compulsory Health Insurance Fund Information System (CHIF IS)	Calendar year (1st January to 31st December)	
Mexico	Ministry of Health, Hospital aggregates database 2004-2021	Calendar year (1st January to 31st December)	Data only include hospitalizations from publicly funded hospitals
Netherlands	Hospital Discharge Register	Calendar year (1st January to 31st December)	Data only include hospitalizations for curative (acute) care only
New Zealand	National Minimum Data Set (NMDS), Ministry of Health	Calendar year (1st January to 31st December)	Data only include hospitalizations that were publicly funded. No data in 2021 were available
Norway	Norwegian Patient Register (NPR) in the Norwegian Directorate of Health	Calendar year (1st January to 31st December)	
Poland	National Institute of Public Health-National Institute of Hygiene	Calendar year (1st January to 31st December)	
Slovak Republic	Institute of Health Information and Statistics	Calendar year (1st January to 31st December)	
Slovenia	National Institute of Public Health, Slovenia; National Hospital Healthcare Statistics Database	Calendar year (1st January to 31st December)	

Spain	Instituto Nacional de Estadística - INE (National Statistics Institute), Encuesta de Morbilidad Hospitalaria (Hospital Morbidity Survey)	Calendar year (1st January to 31st December)	
Sweden	National Board of Health and Welfare, National Patient Register	Calendar year (1st January to 31st December)	
Switzerland	Federal Statistical Office (FSO), Neuchâtel. Medical Statistics of Hospitals	Calendar year (1st January to 31st December)	

Table S2. Descriptions of data sources for surgical procedures by country.

Country	Source	Reference year	Relevant notes on data coverage
Austria	Austrian Ministry of Social Affairs, Health, Care and Consumer Protection, Diagnosis and Performance Reporting	Calendar year (1st January to 31st December)	
Belgium	The Federal Public Service of Health, Food Chain Safety and Environment, Directorate 1 - Minimal Clinical Data	Calendar year (1st January to 31st December)	
Canada	Canadian Institute for Health Information, Discharge Abstract Database and Hospital Morbidity Database	Fiscal year (1st April to 31st March)	Data are for acute care hospitals only
Costa Rica	Área de Estadística en Salud, Caja Costarricense de Seguro Social	Calendar year (1st January to 31st December)	Data only include hospitalizations from public facilities belonging to the Social Insurance (covers 95% of the population)
Czech Republic (Czechia)	Institute of Health Information and Statistics of the Czech Republic	Calendar year (1st January to 31st December)	
Denmark	The Danish Health Data Authority, The National Patient Register	Calendar year (1st January to 31st December)	
Estonia	National Institute for Health Development (NIHD)	Calendar year (1st January to 31st December)	
Finland	THL Finnish Institute for Health and Welfare, Care Register for Institutional Healthcare and Social Insurance Institute (KELA)	Calendar year (1st January to 31st December)	
France	Ministère des Solidarités et de la Santé - Direction de la Recherche, des Études, de l'Évaluation et des Statistiques	Calendar year (1st January to 31st December)	Covers residents of Metropolitan France and overseas departments
Germany	Federal Statistical Office, DRG-statistics 2021	Calendar year (1st January to 31st December)	
Hungary	National Healthcare Service Center; National Directorate General for Hospitals	Calendar year (1st January to 31st December)	
Iceland	Directorate of Health and Icelandic Health Insurance	Calendar year (1st January to 31st December)	
Ireland	Hospital In-Patient Enquiry (HIPE)	Calendar year (1st January to 31st December)	Data only include hospitalizations from publicly funded hospitals
Israel	National Hospital Discharge Database, Health Information Division in the Ministry of Health	Calendar year (1st January to 31st December)	
Italy	General Directorate of Health Planning, National Hospital Discharge Data Base (NHDDB)	Calendar year (1st January to 31st December)	
South Korea	National Health Insurance Service, Main Surgery Statistical Yearbook	Calendar year (1st January to 31st December)	
Lithuania	Health Information Centre of Institute of Hygiene	Calendar year (1st January to 31st December)	
Mexico	Ministry of Health, Hospital aggregates database 2004-2021	Calendar year (1st January to 31st December)	Data only include hospitalizations from publicly funded hospitals
Luxembourg	Fichiers de la sécurité sociale	Calendar year (1st January to 31st December)	
New Zealand	National Minimum Data Set (NMDS), Ministry of Health	Calendar year (1st January to 31st December)	Data only include hospitalizations that were publicly funded. No data in 2021 were available
Norway	Norwegian Patient Register (NPR)	Calendar year (1st January to 31st December)	
Poland	National Institute of Public Health-National Institute of Hygiene	Calendar year (1st January to 31st December)	
Portugal	Ministry of Health, Central Administration of the Health System (ACSS), National Hospital Morbility database	Calendar year (1st January to 31st December)	

Slovak Republic	National Health Information Center (NHIC)	Calendar year (1st January to 31st December)	
Slovenia	National Institute of Public Health, Slovenia; e-DRG system database	Calendar year (1st January to 31st December)	
Spain	Ministerio de Sanidad (Ministry of Health), Subdirección General de Información Sanitaria	Calendar year (1st January to 31st December)	
Sweden	National Board of Health and Welfare, National Patient Register	Calendar year (1st January to 31st December)	
Switzerland	Federal Statistical Office (FSO), Neuchâtel. Medical Statistics of Hospitals	Calendar year (1st January to 31st December)	
United Kingdom	National Health Service Digital	Fiscal year (1st April to 31st March)	

Table S3. Included surgical procedures by urgency.

Surgical urgency	Procedure
**Elective**	-Cataract surgery<br>-Knee replacement<br>-Hip replacement<br>-Tonsillectomy<br>-Transurethral prostatectomy<br>-Hysterectomy<br>-Open prostatectomy
**Non-elective**	-Appendectomy<br>-Cholecystectomy<br>-Mastectomy<br>-Partial mastectomy
**Both**	-Transluminal coronary angioplasty<br>-Coronary artery bypass graft<br>-Repair of inguinal hernia<br>-Caesarean section

Table S4. Description of country-level characteristics used as covariates.

Dimension	Indicator	Measurement	Source
Health system	Hospital beds per capita	Total hospital beds per 1000 population in 2019	Organisation for Economic Co-operation and Development (OECD)
Health system	Total health employment rate	Total health and social employment rate per 1,000 population in 2019	Organisation for Economic Co-operation and Development (OECD)
Health system	Doctors per capita	Total practicing physicians per 1,000 population in 2019	Organisation for Economic Co-operation and Development (OECD)
Health system	Nurses per capita	Total practicing nurses providing services directly to patients per 1,000 population in 2019	Organisation for Economic Co-operation and Development (OECD)
Health system	Care personnel per capita	Total practicing caring personnel assisting in institutions and home-based per 1,000 population in 2019	Organisation for Economic Co-operation and Development (OECD)
Health system	Insurance coverage	Percent of population having public or primary voluntary health insurance coverage in 2019	Organisation for Economic Co-operation and Development (OECD)
Health system	Share of GDP for health care	Percent of GDP for health care spending in 2019	Organisation for Economic Co-operation and Development (OECD)
Health system	Share for inpatient care	Percent of health expenditures for inpatient curative and rehabilitative care in 2019	Organisation for Economic Co-operation and Development (OECD)
Health system	Share for outpatient care	Percent of health expenditures for outpatient curative and rehabilitative care in 2019	Organisation for Economic Co-operation and Development (OECD)
Health system	Share for preventive care	Percent of health expenditures for preventive care in 2019	Organisation for Economic Co-operation and Development (OECD)
Health system	Doctor consultations per capita	In-person doctor consultations per capita in 2019	Organisation for Economic Co-operation and Development (OECD)
Health system	Telehealth consultations per capita	Telehealth doctor consultations per capita by pandemic year	Organisation for Economic Co-operation and Development (OECD)
Socio-demographic	Gross domestic product (GDP) per capita	Gross domestic product (GDP) per 1,000 population in 2019	World Bank
Socio-demographic	Share of GDP for social spending	Percent of GDP for social spending in 2019	Organisation for Economic Co-operation and Development (OECD)
Socio-demographic	Income inequality	Income inequality measured from the Gini index in 2019 (or latest year of data)	World Bank
Socio-demographic	Poverty rate	Ratio of the number of people whose income falls below the poverty line (half the median household income of country) in 2019 (or latest year of data)	Organisation for Economic Co-operation and Development (OECD)
Socio-demographic	% population > 65 years	Percent of total population aged 65 years and above in 2019	World Bank
Public health and social measures	Pandemic preparedness	As measured from the Global Health Security Index in 2019	Global Health Security Index

Public health and social measures	Mobility	Average of daily percent change in total mobility compared to pre-pandemic period stratified by pandemic year	Institute for Health Metrics and Evaluation
Public health and social measures	Stringency index	Average of daily stringency index score which captures the strictness of closures and COVID-19 containment policy stratified by pandemic year	Oxford Covid-19 Government Response Tracker (OxCGRT)
Public health and social measures	Stay-at-home orders	Number of days with a required stay-at-home order in effect stratified by pandemic year	Oxford Covid-19 Government Response Tracker (OxCGRT)
Public health and social measures	School closures	Number of days with a required school closure for at least some levels in effect stratified by pandemic year	Oxford Covid-19 Government Response Tracker (OxCGRT)
Public health and social measures	Workplace closures	Number of days with a required school closure for at least some sectors in effect stratified by pandemic year	Oxford Covid-19 Government Response Tracker (OxCGRT)
COVID-19 severity	COVID-19 case rate	Reported COVID-19 case rate per 1,000 population by pandemic year	World Health Organization
COVID-19 severity	COVID-19 death rate	Reported COVID-19 death rate per 1,000 population by pandemic year	World Health Organization
COVID-19 severity	COVID-19 hospitalization rate	Reported COVID-19 hospitalization rate per 1,000 population by pandemic year	Organisation for Economic Co-operation and Development (OECD)
COVID-19 severity	Excess avoidable death rate	Excess age-standardized avoidable death rate in 2020 (computed ourselves using ARIMA methods described in paper)	Organisation for Economic Co-operation and Development (OECD)
COVID-19 severity	Excess avoidable death rate (excluding deaths due to COVID-19)	Excess avoidable death rates after subtracting deaths due to COVID-19 in 2020 (computed ourselves using ARIMA methods described in paper)	Organisation for Economic Co-operation and Development (OECD)
COVID019 severity	Excess all-cause death rate	Excess COVID-19 death rate per 1,000 population by pandemic year	World Health Organization
COVID-19 severity	Excess all-cause death rate (excluding deaths due to COVID-19)	Excess COVID-19 death rate per 1,000 population by pandemic year after subtracting deaths due to COVID-19	World Health Organization

Table S5. Expected and observed inpatient hospitalization and average length of stay by country, 2020-2021.

Location	Year	Condition	Observed hospitalizations	Observed hospitalizations per 1,000 population	Expected hospitalizations	Expected hospitalizations per 1,000 population	Difference in hospitalizations	Difference in rate	Hospitalizations observed-to-expected ratio	Observed length of stay	Expected length of stay	Difference in length of stay	Length of stay observed-to-expected ratio
OECD	2020	All causes	74,100,000	113	90,400,000 (88,800,000 to 92,000,000)	138 (136 to 141)	-16,300,000 (-17,900,000 to -14,700,000)	-24.9 (-27.4 to -22.5)	0.82 (0.81 to 0.83)				
OECD	2021	All causes	75,200,000	116	89,900,000 (88,300,000 to 91,500,000)	138 (136 to 141)	-14,700,000 (-16,400,000 to -13,100,000)	-22.6 (-25.2 to -20.2)	0.84 (0.82 to 0.85)				
OECD	2020	Infectious and parasitic diseases	1,730,000	2.65	2,550,000 (2,480,000 to 2,630,000)	3.90 (3.79 to 4.02)	-818,000 (-892,000 to -745,000)	-1.25 (-1.37 to -1.14)	0.68 (0.66 to 0.70)	9.4	8.01 (7.78 to 8.25)	1.39 (1.16 to 1.62)	1.17 (1.14 to 1.21)
OECD	2021	Infectious and parasitic diseases	1,790,000	2.76	2,580,000 (2,470,000 to 2,690,000)	3.96 (3.80 to 4.13)	-783,000 (-895,000 to -680,000)	-1.21 (-1.38 to -1.05)	0.70 (0.67 to 0.72)	9.38	8.04 (7.71 to 8.38)	1.34 (1.00 to 1.67)	1.17 (1.12 to 1.22)
OECD	2020	Neoplasms	7,050,000	10.8	7,960,000 (7,810,000 to 8,110,000)	12.2 (12.0 to 12.4)	-905,000 (-1,050,000 to -759,000)	-1.39 (-1.61 to -1.16)	0.89 (0.87 to 0.90)	7.38	7.39 (7.23 to 7.55)	-0.006 (-0.164 to 0.152)	1.00 (0.98 to 1.02)
OECD	2021	Neoplasms	7,250,000	11.2	7,960,000 (7,800,000 to 8,120,000)	12.2 (12.0 to 12.5)	-708,000 (-871,000 to -547,000)	-1.09 (-1.34 to -0.842)	0.91 (0.89 to 0.93)	7.17	7.33 (7.10 to 7.56)	-0.161 (-0.388 to 0.058)	0.98 (0.95 to 1.01)
OECD	2020	Diseases of the blood and bloodforming organs	636,000	0.974	753,000 (737,000 to 768,000)	1.15 (1.13 to 1.18)	-116,000 (-132,000 to -101,000)	-0.178 (-0.202 to -0.155)	0.85 (0.83 to 0.86)	6.87	6.73 (6.46 to 7.01)	0.143 (-0.131 to 0.410)	1.02 (0.98 to 1.06)
OECD	2021	Diseases of the blood and bloodforming organs	660,000	1.02	750,000 (728,000 to 772,000)	1.15 (1.12 to 1.19)	-89,500 (-112,000 to -68,100)	-0.138 (-0.172 to -0.105)	0.88 (0.86 to 0.91)	7.01	6.75 (6.36 to 7.15)	0.263 (-0.147 to 0.643)	1.04 (0.98 to 1.10)
OECD	2020	Endocrine, nutritional and metabolic diseases	1,730,000	2.65	2,200,000 (2,140,000 to 2,260,000)	3.37 (3.28 to 3.46)	-472,000 (-528,000 to -415,000)	-0.722 (-0.809 to -0.636)	0.79 (0.77 to 0.81)	7.64	7.67 (7.42 to 7.93)	-0.035 (-0.293 to 0.215)	1.00 (0.96 to 1.03)
OECD	2021	Endocrine, nutritional and metabolic diseases	1,820,000	2.80	2,190,000 (2,120,000 to 2,270,000)	3.37 (3.26 to 3.50)	-372,000 (-452,000 to -295,000)	-0.572 (-0.696 to -0.454)	0.83 (0.80 to 0.86)	7.44	7.68 (7.33 to 8.05)	-0.237 (-0.603 to 0.114)	0.97 (0.93 to 1.02)
OECD	2020	Mental and behavioural disorders	3,470,000	5.31	4,060,000 (3,720,000 to 4,430,000)	6.22 (5.70 to 6.78)	-593,000 (-964,000 to -252,000)	-0.908 (-1.48 to -0.385)	0.85 (0.78 to 0.93)	43.15	37.66 (34.34 to 41.30)	5.48 (1.83 to 8.89)	1.15 (1.04 to 1.26)
OECD	2021	Mental and behavioural disorders	3,550,000	5.47	4,050,000 (3,570,000 to 4,580,000)	6.23 (5.50 to 7.05)	-494,000 (-1,030,000 to -18,700)	-0.760 (-1.58 to -0.029)	0.88 (0.78 to 0.99)	39.36	37.69 (33.01 to 43.03)	1.66 (-3.71 to 6.29)	1.04 (0.91 to 1.19)
OECD	2020	Diseases of the nervous system	2,100,000	3.22	2,670,000 (2,580,000 to 2,780,000)	4.09 (3.94 to 4.25)	-570,000 (-674,000 to -472,000)	-0.873 (-1.03 to -0.722)	0.79 (0.76 to 0.82)	14.32	13.97 (13.08 to 14.91)	0.355 (-0.579 to 1.23)	1.03 (0.96 to 1.09)
OECD	2021	Diseases of the nervous system	2,140,000	3.29	2,660,000 (2,560,000 to 2,770,000)	4.10 (3.95 to 4.26)	-527,000 (-628,000 to -427,000)	-0.811 (-0.967 to -0.657)	0.80 (0.77 to 0.83)	14.84	14.35 (13.10 to 15.72)	0.490 (-0.885 to 1.73)	1.03 (0.94 to 1.13)
OECD	2020	Diseases of the eye and adnexa	684,000	1.05	894,000 (848,000 to 943,000)	1.37 (1.30 to 1.44)	-210,000 (-258,000 to -165,000)	-0.322 (-0.396 to -0.252)	0.76 (0.73 to 0.81)	3.13	2.82 (2.69 to 2.95)	0.310 (0.177 to 0.435)	1.11 (1.06 to 1.16)
OECD	2021	Diseases of the eye and adnexa	694,000	1.07	859,000 (797,000 to 926,000)	1.32 (1.23 to 1.42)	-166,000 (-232,000 to -105,000)	-0.255 (-0.357 to -0.162)	0.81 (0.75 to 0.87)	3.13	2.79 (2.67 to 2.91)	0.336 (0.215 to 0.450)	1.12 (1.07 to 1.17)
OECD	2020	Diseases of the ear and mastoid process	396,000	0.605	533,000 (518,000 to 548,000)	0.816 (0.794 to 0.838)	-137,000 (-152,000 to -123,000)	-0.210 (-0.232 to -0.188)	0.74 (0.72 to 0.76)	3.54	3.52 (3.37 to 3.67)	0.019 (-0.137 to 0.169)	1.01 (0.96 to 1.05)
OECD	2021	Diseases of the ear and mastoid process	389,000	0.599	525,000 (505,000 to 546,000)	0.808 (0.777 to 0.840)	-136,000 (-157,000 to -116,000)	-0.210 (-0.241 to -0.179)	0.74 (0.71 to 0.77)	3.5	3.53 (3.33 to 3.75)	-0.034 (-0.253 to 0.167)	0.99 (0.93 to 1.05)

Location	Year	Condition	Observed hospitalizations	Observed hospitalizations per 1,000 population	Expected hospitalizations	Expected hospitalizations per 1,000 population	Difference in hospitalizations	Difference in rate	Hospitalizations observed-to-expected ratio	Observed length of stay	Expected length of stay	Difference in length of stay	Length of stay observed-to-expected ratio
OECD	2020	Diseases of the circulatory system	9,190,000	14.1	10,900,000 (10,600,000 to 11,200,000)	16.7 (16.2 to 17.1)	-1,680,000 (-1,960,000 to -1,410,000)	-2.58 (-3.01 to -2.16)	0.85 (0.82 to 0.87)	9.64	9.87 (9.62 to 10.13)	-0.236 (-0.503 to 0.014)	0.98 (0.95 to 1.00)
OECD	2021	Diseases of the circulatory system	9,380,000	14.4	10,800,000 (10,500,000 to 11,200,000)	16.7 (16.1 to 17.3)	-1,450,000 (-1,850,000 to -1,070,000)	-2.24 (-2.84 to -1.64)	0.87 (0.84 to 0.90)	9.6	9.84 (9.60 to 10.09)	-0.244 (-0.481 to -0.004)	0.98 (0.95 to 1.00)
OECD	2020	Diseases of the respiratory system	4,840,000	7.41	6,870,000 (6,610,000 to 7,140,000)	10.5 (10.1 to 10.9)	-2,030,000 (-2,300,000 to -1,780,000)	-3.11 (-3.52 to -2.72)	0.70 (0.68 to 0.73)	8.76	7.29 (7.05 to 7.53)	1.47 (1.22 to 1.71)	1.20 (1.16 to 1.24)
OECD	2021	Diseases of the respiratory system	4,650,000	7.15	6,830,000 (6,460,000 to 7,210,000)	10.5 (9.94 to 11.1)	-2,180,000 (-2,570,000 to -1,810,000)	-3.35 (-3.95 to -2.79)	0.68 (0.64 to 0.72)	9.03	7.48 (7.15 to 7.82)	1.55 (1.22 to 1.88)	1.21 (1.16 to 1.26)
OECD	2020	Diseases of the digestive system	6,620,000	10.1	8,050,000 (7,880,000 to 8,240,000)	12.3 (12.1 to 12.6)	-1,430,000 (-1,620,000 to -1,260,000)	-2.20 (-2.47 to -1.92)	0.82 (0.80 to 0.84)	5.45	5.21 (5.03 to 5.40)	0.243 (0.053 to 0.425)	1.05 (1.01 to 1.08)
OECD	2021	Diseases of the digestive system	6,870,000	10.6	8,010,000 (7,760,000 to 8,270,000)	12.3 (11.9 to 12.7)	-1,140,000 (-1,400,000 to -893,000)	-1.76 (-2.15 to -1.37)	0.86 (0.83 to 0.88)	5.33	5.16 (4.90 to 5.43)	0.168 (-0.103 to 0.418)	1.03 (0.98 to 1.09)
OECD	2020	Diseases of the skin and subcutaneous tissue	800,000	1.22	1,100,000 (1,070,000 to 1,130,000)	1.68 (1.63 to 1.73)	-300,000 (-334,000 to -268,000)	-0.460 (-0.511 to -0.410)	0.73 (0.71 to 0.75)	7.91	7.56 (7.29 to 7.85)	0.348 (0.060 to 0.617)	1.05 (1.01 to 1.08)
OECD	2021	Diseases of the skin and subcutaneous tissue	775,000	1.19	1,080,000 (1,050,000 to 1,120,000)	1.67 (1.62 to 1.72)	-309,000 (-342,000 to -276,000)	-0.476 (-0.527 to -0.424)	0.71 (0.69 to 0.74)	8.04	7.59 (7.31 to 7.87)	0.456 (0.169 to 0.731)	1.06 (1.02 to 1.10)
OECD	2020	Diseases of musculoskeletal system and connective tissue	5,940,000	9.1	7,430,000 (7,210,000 to 7,660,000)	11.4 (11.0 to 11.7)	-1,480,000 (-1,720,000 to -1,260,000)	-2.27 (-2.63 to -1.93)	0.80 (0.78 to 0.83)	6.67	6.72 (6.51 to 6.93)	-0.045 (-0.261 to 0.157)	0.99 (0.96 to 1.02)
OECD	2021	Diseases of musculoskeletal system and connective tissue	6,030,000	9.28	7,400,000 (7,090,000 to 7,730,000)	11.4 (10.9 to 11.9)	-1,370,000 (-1,700,000 to -1,060,000)	-2.11 (-2.62 to -1.63)	0.82 (0.78 to 0.85)	6.52	6.71 (6.35 to 7.08)	-0.189 (-0.554 to 0.163)	0.97 (0.92 to 1.03)
OECD	2020	Diseases of the genitourinary system	4,150,000	6.35	5,090,000 (5,030,000 to 5,150,000)	7.79 (7.70 to 7.89)	-944,000 (-1,000,000 to -884,000)	-1.45 (-1.54 to -1.35)	0.81 (0.80 to 0.82)	5.57	5.38 (5.25 to 5.51)	0.192 (0.060 to 0.321)	1.04 (1.01 to 1.06)
OECD	2021	Diseases of the genitourinary system	4,290,000	6.61	5,080,000 (5,020,000 to 5,140,000)	7.82 (7.72 to 7.91)	-786,000 (-850,000 to -724,000)	-1.21 (-1.31 to -1.11)	0.85 (0.83 to 0.86)	5.53	5.39 (5.21 to 5.58)	0.137 (-0.049 to 0.318)	1.03 (0.99 to 1.06)
OECD	2020	Pregnancy, childbirth and the puerperium	6,610,000	10.1	7,260,000 (7,060,000 to 7,470,000)	11.1 (10.8 to 11.4)	-646,000 (-857,000 to -447,000)	-0.989 (-1.31 to -0.684)	0.91 (0.89 to 0.94)	3.19	3.32 (3.27 to 3.37)	-0.132 (-0.181 to -0.083)	0.96 (0.95 to 0.97)
OECD	2021	Pregnancy, childbirth and the puerperium	6,520,000	10.0	7,070,000 (6,790,000 to 7,360,000)	10.9 (10.4 to 11.3)	-549,000 (-839,000 to -272,000)	-0.845 (-1.29 to -0.418)	0.92 (0.89 to 0.96)	3.22	3.30 (3.23 to 3.37)	-0.084 (-0.155 to -0.016)	0.97 (0.95 to 0.99)
OECD	2020	Certain conditions originating in the perinatal period	1,100,000	1.68	1,150,000 (1,120,000 to 1,170,000)	1.75 (1.71 to 1.80)	-45,200 (-74,400 to -17,200)	-0.069 (-0.114 to -0.026)	0.96 (0.94 to 0.98)	9.06	8.98 (8.70 to 9.27)	0.084 (-0.207 to 0.376)	1.01 (0.98 to 1.04)
OECD	2021	Certain conditions originating in the perinatal period	1,110,000	1.70	1,110,000 (1,070,000 to 1,150,000)	1.71 (1.65 to 1.77)	-874 (-41,200 to 37,400)	-0.001 (-0.063 to 0.058)	1.00 (0.96 to 1.03)	9.02	8.99 (8.58 to 9.41)	0.037 (-0.380 to 0.446)	1.00 (0.96 to 1.05)
OECD	2020	Congenital malformations, deformations and chromosomal abnormalities	439,000	0.671	534,000 (526,000 to 542,000)	0.817 (0.805 to 0.829)	-95,100 (-103,000 to -87,200)	-0.146 (-0.158 to -0.133)	0.82 (0.81 to 0.83)	6.6	6.23 (6.10 to 6.37)	0.368 (0.233 to 0.500)	1.06 (1.04 to 1.08)

Location	Year	Condition	Observed hospitalizations	Observed hospitalizations per 1,000 population	Expected hospitalizations	Expected hospitalizations per 1,000 population	Difference in hospitalizations	Difference in rate	Hospitalizations observed-to-expected ratio	Observed length of stay	Expected length of stay	Difference in length of stay	Length of stay observed-to-expected ratio
OECD	2021	Congenital malformations, deformations and chromosomal abnormalities	471,000	0.725	521,000 (510,000 to 533,000)	0.802 (0.785 to 0.820)	-50,200 (-61,300 to -39,200)	-0.077 (-0.094 to -0.060)	0.90 (0.88 to 0.92)	6.42	6.24 (6.10 to 6.38)	0.176 (0.037 to 0.311)	1.03 (1.01 to 1.05)
OECD	2020	Symptoms, signs and abnormal clinical and laboratory findings, n.e.c.	3,140,000	4.81	3,980,000 (3,840,000 to 4,120,000)	6.09 (5.88 to 6.30)	-836,000 (-977,000 to -701,000)	-1.28 (-1.50 to -1.07)	0.79 (0.76 to 0.82)	6.71	6.13 (5.78 to 6.50)	0.574 (0.194 to 0.931)	1.09 (1.03 to 1.16)
OECD	2021	Symptoms, signs and abnormal clinical and laboratory findings, n.e.c.	3,210,000	4.94	3,930,000 (3,790,000 to 4,070,000)	6.04 (5.84 to 6.26)	-716,000 (-856,000 to -584,000)	-1.10 (-1.32 to -0.899)	0.82 (0.79 to 0.85)	6.69	6.22 (5.44 to 7.12)	0.466 (-0.425 to 1.26)	1.07 (0.94 to 1.23)
OECD	2020	Injury, poisoning and other consequences of external causes	7,400,000	11.3	8,450,000 (8,270,000 to 8,640,000)	12.9 (12.7 to 13.2)	-1,050,000 (-1,240,000 to -866,000)	-1.61 (-1.90 to -1.33)	0.88 (0.86 to 0.90)	7.46	7.48 (7.38 to 7.58)	-0.021 (-0.123 to 0.084)	1.00 (0.98 to 1.01)
OECD	2021	Injury, poisoning and other consequences of external causes	7,480,000	11.5	8,390,000 (8,210,000 to 8,580,000)	12.9 (12.6 to 13.2)	-918,000 (-1,110,000 to -732,000)	-1.41 (-1.71 to -1.13)	0.89 (0.87 to 0.91)	7.37	7.43 (7.27 to 7.58)	-0.055 (-0.213 to 0.099)	0.99 (0.97 to 1.01)
OECD	2020	Factors influencing health status and contact with health services	6,060,000	9.28	6,850,000 (6,030,000 to 7,780,000)	10.5 (9.24 to 11.9)	-791,000 (-1,700,000 to 28,600)	-1.21 (-2.61 to 0.044)	0.88 (0.78 to 1.00)	6.32	6.36 (5.74 to 7.05)	-0.037 (-0.745 to 0.592)	0.99 (0.89 to 1.10)
OECD	2021	Factors influencing health status and contact with health services	6,080,000	9.39	6,770,000 (5,640,000 to 8,120,000)	10.4 (8.70 to 12.5)	-681,000 (-2,040,000 to 440,000)	-1.05 (-3.14 to 0.680)	0.90 (0.75 to 1.08)	6.23	6.37 (5.50 to 7.38)	-0.141 (-1.15 to 0.714)	0.98 (0.84 to 1.13)
Austria	2020	All causes	1,770,000	199	2,140,000 (2,110,000 to 2,160,000)	240 (237 to 243)	-363,000 (-390,000 to -337,000)	-40.7 (-43.8 to -37.8)	0.83 (0.82 to 0.84)				
Austria	2021	All causes	1,830,000	204	2,120,000 (2,080,000 to 2,150,000)	236 (232 to 240)	-284,000 (-322,000 to -247,000)	-31.8 (-36.0 to -27.6)	0.87 (0.85 to 0.88)				
Austria	2020	Infectious and parasitic diseases	38,900	4.36	54,400 (52,200 to 56,800)	6.11 (5.86 to 6.37)	-15,600 (-17,900 to -13,300)	-1.75 (-2.01 to -1.49)	0.71 (0.68 to 0.75)	8	7.41 (6.99 to 7.85)	0.593 (0.149 to 1.01)	1.08 (1.02 to 1.14)
Austria	2021	Infectious and parasitic diseases	40,200	4.49	54,700 (51,600 to 58,000)	6.11 (5.76 to 6.48)	-14,500 (-17,800 to -11,400)	-1.62 (-1.99 to -1.27)	0.73 (0.69 to 0.78)	8	7.41 (6.99 to 7.85)	0.593 (0.149 to 1.02)	1.08 (1.02 to 1.15)
Austria	2020	Neoplasms	225,000	25.2	251,000 (245,000 to 257,000)	28.2 (27.5 to 28.8)	-25,900 (-32,000 to -20,200)	-2.91 (-3.59 to -2.26)	0.90 (0.88 to 0.92)	6.6	6.60 (6.53 to 6.67)	0.000 (-0.065 to 0.065)	1.00 (0.99 to 1.01)
Austria	2021	Neoplasms	231,000	25.8	252,000 (244,000 to 261,000)	28.2 (27.2 to 29.1)	-20,700 (-29,200 to -12,500)	-2.31 (-3.26 to -1.40)	0.92 (0.89 to 0.95)	6.5	6.60 (6.51 to 6.69)	-0.100 (-0.193 to -0.004)	0.98 (0.97 to 1.00)
Austria	2020	Diseases of the blood and bloodforming organs	13,200	1.48	15,400 (15,000 to 15,800)	1.73 (1.69 to 1.78)	-2,280 (-2,690 to -1,870)	-0.255 (-0.302 to -0.210)	0.85 (0.83 to 0.88)	6.6	6.47 (6.05 to 6.92)	0.133 (-0.323 to 0.560)	1.02 (0.95 to 1.09)
Austria	2021	Diseases of the blood and bloodforming organs	14,200	1.58	15,500 (15,100 to 15,900)	1.73 (1.69 to 1.78)	-1,340 (-1,760 to -939)	-0.150 (-0.196 to -0.105)	0.91 (0.89 to 0.94)	6.8	6.47 (6.05 to 6.92)	0.333 (-0.112 to 0.758)	1.05 (0.98 to 1.13)
Austria	2020	Endocrine, nutritional and metabolic diseases	42,400	4.76	54,800 (53,000 to 56,700)	6.15 (5.94 to 6.35)	-12,400 (-14,300 to -10,600)	-1.39 (-1.60 to -1.19)	0.77 (0.75 to 0.80)	9.8	9.39 (8.85 to 9.95)	0.414 (-0.137 to 0.949)	1.04 (0.99 to 1.11)

Location	Year	Condition	Observed hospitalizations	Observed hospitalizations per 1,000 population	Expected hospitalizations	Expected hospitalizations per 1,000 population	Difference in hospitalizations	Difference in rate	Hospitalizations observed-to-expected ratio	Observed length of stay	Expected length of stay	Difference in length of stay	Length of stay observed-to-expected ratio
Austria	2021	Endocrine, nutritional and metabolic diseases	45,400	5.07	53,700 (51,200 to 56,300)	5.99 (5.72 to 6.28)	-8,300 (-10,900 to -5,860)	-0.927 (-1.21 to -0.655)	0.85 (0.81 to 0.89)	9.5	9.39 (8.85 to 9.95)	0.114 (-0.446 to 0.646)	1.01 (0.96 to 1.07)
Austria	2020	Mental and behavioural disorders	87,100	9.76	91,700 (86,200 to 97,500)	10.3 (9.67 to 10.9)	-4,580 (-10,300 to 951)	-0.514 (-1.15 to 0.107)	0.95 (0.89 to 1.01)	27	25.60 (21.54 to 30.43)	1.40 (-3.36 to 5.46)	1.05 (0.89 to 1.25)
Austria	2021	Mental and behavioural disorders	92,800	10.4	82,700 (72,100 to 94,900)	9.23 (8.05 to 10.6)	10,100 (-2,100 to 20,700)	1.12 (-0.235 to 2.31)	1.12 (0.98 to 1.29)	26.7	25.60 (20.05 to 32.69)	1.10 (-6.03 to 6.59)	1.04 (0.82 to 1.33)
Austria	2020	Diseases of the nervous system	79,600	8.93	100,000 (96,200 to 105,000)	11.3 (10.8 to 11.8)	-20,900 (-25,400 to -16,600)	-2.34 (-2.85 to -1.86)	0.79 (0.76 to 0.83)	9.4	9.02 (8.45 to 9.61)	0.385 (-0.216 to 0.943)	1.04 (0.98 to 1.11)
Austria	2021	Diseases of the nervous system	86,500	9.66	101,000 (95,000 to 107,000)	11.3 (10.6 to 12.0)	-14,300 (-20,600 to -8,480)	-1.60 (-2.30 to -0.947)	0.86 (0.81 to 0.91)	8.7	9.23 (8.63 to 9.87)	-0.529 (-1.17 to 0.073)	0.94 (0.88 to 1.01)
Austria	2020	Diseases of the eye and adnexa	21,800	2.45	26,400 (24,100 to 29,000)	2.97 (2.70 to 3.25)	-4,620 (-7,130 to -2,310)	-0.518 (-0.800 to -0.259)	0.83 (0.75 to 0.90)	2.6	2.60 (2.37 to 2.85)	0.000 (-0.251 to 0.236)	1.00 (0.91 to 1.10)
Austria	2021	Diseases of the eye and adnexa	22,900	2.56	23,700 (20,800 to 27,000)	2.65 (2.32 to 3.02)	-811 (-4,160 to 2,060)	-0.091 (-0.465 to 0.230)	0.97 (0.85 to 1.10)	2.6	2.60 (2.28 to 2.97)	0.000 (-0.363 to 0.324)	1.00 (0.88 to 1.14)
Austria	2020	Diseases of the ear and mastoid process	13,500	1.52	18,100 (17,500 to 18,700)	2.03 (1.96 to 2.10)	-4,570 (-5,190 to -3,980)	-0.513 (-0.582 to -0.446)	0.75 (0.72 to 0.77)	4	4.20 (3.89 to 4.54)	-0.200 (-0.537 to 0.113)	0.95 (0.88 to 1.03)
Austria	2021	Diseases of the ear and mastoid process	14,000	1.56	17,600 (16,800 to 18,400)	1.97 (1.88 to 2.06)	-3,600 (-4,450 to -2,790)	-0.402 (-0.497 to -0.311)	0.80 (0.76 to 0.83)	3.9	4.20 (3.77 to 4.68)	-0.300 (-0.769 to 0.148)	0.93 (0.84 to 1.04)
Austria	2020	Diseases of the circulatory system	241,000	27.0	282,000 (273,000 to 291,000)	31.6 (30.6 to 32.7)	-41,400 (-50,400 to -32,200)	-4.64 (-5.65 to -3.61)	0.85 (0.83 to 0.88)	10.6	10.50 (10.10 to 10.92)	0.100 (-0.317 to 0.510)	1.01 (0.97 to 1.05)
Austria	2021	Diseases of the circulatory system	254,000	28.3	279,000 (267,000 to 292,000)	31.2 (29.8 to 32.6)	-25,400 (-38,700 to -13,200)	-2.84 (-4.32 to -1.47)	0.91 (0.87 to 0.95)	10.4	10.50 (9.94 to 11.09)	-0.100 (-0.687 to 0.451)	0.99 (0.94 to 1.05)
Austria	2020	Diseases of the respiratory system	102,000	11.4	147,000 (141,000 to 154,000)	16.5 (15.8 to 17.3)	-45,600 (-52,300 to -39,000)	-5.12 (-5.86 to -4.38)	0.69 (0.66 to 0.72)	8.1	7.52 (7.30 to 7.75)	0.581 (0.362 to 0.801)	1.08 (1.05 to 1.11)
Austria	2021	Diseases of the respiratory system	93,200	10.4	147,000 (140,000 to 154,000)	16.4 (15.6 to 17.2)	-53,700 (-60,800 to -47,000)	-5.99 (-6.79 to -5.25)	0.63 (0.61 to 0.66)	8.2	7.52 (7.30 to 7.75)	0.681 (0.451 to 0.898)	1.09 (1.06 to 1.12)
Austria	2020	Diseases of the digestive system	164,000	18.4	203,000 (197,000 to 209,000)	22.8 (22.1 to 23.5)	-38,700 (-45,100 to -32,400)	-4.34 (-5.06 to -3.64)	0.81 (0.78 to 0.84)	5.2	4.94 (4.76 to 5.12)	0.263 (0.072 to 0.444)	1.05 (1.01 to 1.09)
Austria	2021	Diseases of the digestive system	170,000	19.0	204,000 (195,000 to 213,000)	22.8 (21.8 to 23.8)	-34,000 (-43,400 to -25,400)	-3.80 (-4.85 to -2.84)	0.83 (0.80 to 0.87)	5	4.88 (4.63 to 5.14)	0.124 (-0.137 to 0.375)	1.03 (0.97 to 1.08)
Austria	2020	Diseases of the skin and subcutaneous tissue	18,900	2.12	23,500 (22,600 to 24,400)	2.63 (2.54 to 2.73)	-4,610 (-5,490 to -3,760)	-0.517 (-0.616 to -0.421)	0.80 (0.77 to 0.83)	6.9	6.30 (6.00 to 6.62)	0.600 (0.281 to 0.914)	1.10 (1.04 to 1.15)
Austria	2021	Diseases of the skin and subcutaneous tissue	18,400	2.05	22,700 (21,600 to 24,000)	2.54 (2.41 to 2.68)	-4,350 (-5,570 to -3,180)	-0.486 (-0.622 to -0.355)	0.81 (0.77 to 0.85)	6.5	6.30 (5.88 to 6.75)	0.200 (-0.255 to 0.625)	1.03 (0.96 to 1.11)
Austria	2020	Diseases of musculoskeletal system and connective tissue	202,000	22.7	258,000 (250,000 to 266,000)	28.9 (28.1 to 29.9)	-56,100 (-64,200 to -47,900)	-6.29 (-7.20 to -5.37)	0.78 (0.76 to 0.81)	9.5	9.80 (9.53 to 10.08)	-0.300 (-0.569 to -0.026)	0.97 (0.94 to 1.00)
Austria	2021	Diseases of musculoskeletal system and connective tissue	215,000	24.0	256,000 (245,000 to 268,000)	28.6 (27.4 to 29.9)	-40,900 (-52,500 to -29,700)	-4.57 (-5.86 to -3.32)	0.84 (0.80 to 0.88)	9.6	9.80 (9.42 to 10.20)	-0.200 (-0.604 to 0.184)	0.98 (0.94 to 1.02)

Location	Year	Condition	Observed hospitalizations	Observed hospitalizations per 1,000 population	Expected hospitalizations	Expected hospitalizations per 1,000 population	Difference in hospitalizations	Difference in rate	Hospitalizations observed-to-expected ratio	Observed length of stay	Expected length of stay	Difference in length of stay	Length of stay observed-to-expected ratio
Austria	2020	Diseases of the genitourinary system	106,000	11.9	125,000 (123,000 to 127,000)	14.0 (13.8 to 14.2)	-19,500 (-21,300 to -17,700)	-2.18 (-2.39 to -1.98)	0.84 (0.83 to 0.86)	5.2	5.12 (4.97 to 5.28)	0.081 (-0.078 to 0.230)	1.02 (0.99 to 1.05)
Austria	2021	Diseases of the genitourinary system	110,000	12.3	125,000 (121,000 to 129,000)	14.0 (13.5 to 14.4)	-14,900 (-19,000 to -10,900)	-1.66 (-2.12 to -1.22)	0.88 (0.85 to 0.91)	5.2	5.12 (4.97 to 5.28)	0.081 (-0.077 to 0.230)	1.02 (0.99 to 1.05)
Austria	2020	Pregnancy, childbirth and the puerperium	101,000	11.3	113,000 (108,000 to 119,000)	12.7 (12.2 to 13.3)	-12,800 (-18,100 to -7,830)	-1.44 (-2.03 to -0.878)	0.89 (0.85 to 0.93)	3.7	3.90 (3.81 to 3.99)	-0.200 (-0.290 to -0.113)	0.95 (0.93 to 0.97)
Austria	2021	Pregnancy, childbirth and the puerperium	103,000	11.5	114,000 (109,000 to 119,000)	12.7 (12.2 to 13.3)	-10,800 (-16,000 to -5,720)	-1.20 (-1.79 to -0.639)	0.91 (0.87 to 0.95)	3.7	3.90 (3.73 to 4.08)	-0.200 (-0.375 to -0.032)	0.95 (0.91 to 0.99)
Austria	2020	Certain conditions originating in the perinatal period	11,400	1.28	12,700 (12,000 to 13,300)	1.42 (1.35 to 1.49)	-1,230 (-1,870 to -608)	-0.137 (-0.210 to -0.068)	0.90 (0.86 to 0.95)	11.2	11.26 (10.70 to 11.84)	-0.057 (-0.638 to 0.490)	0.99 (0.95 to 1.05)
Austria	2021	Certain conditions originating in the perinatal period	12,200	1.36	12,700 (12,100 to 13,400)	1.42 (1.35 to 1.49)	-512 (-1,180 to 123)	-0.057 (-0.132 to 0.014)	0.96 (0.91 to 1.01)	10.8	11.26 (10.70 to 11.84)	-0.457 (-1.03 to 0.107)	0.96 (0.91 to 1.01)
Austria	2020	Congenital malformations, deformations and chromosomal abnormalities	12,000	1.34	13,800 (13,100 to 14,400)	1.54 (1.47 to 1.62)	-1,780 (-2,430 to -1,170)	-0.199 (-0.272 to -0.131)	0.87 (0.83 to 0.91)	5.9	5.89 (4.97 to 6.97)	0.011 (-1.06 to 0.910)	1.00 (0.85 to 1.18)
Austria	2021	Congenital malformations, deformations and chromosomal abnormalities	13,300	1.48	13,500 (12,600 to 14,400)	1.51 (1.41 to 1.61)	-208 (-1,120 to 639)	-0.023 (-0.125 to 0.071)	0.98 (0.92 to 1.05)	7	5.89 (4.97 to 6.97)	1.11 (0.055 to 2.03)	1.19 (1.01 to 1.41)
Austria	2020	Symptoms, signs and abnormal clinical and laboratory findings, n.e.c.	68,500	7.68	88,100 (83,600 to 92,900)	9.88 (9.37 to 10.4)	-19,600 (-24,300 to -15,200)	-2.20 (-2.73 to -1.71)	0.78 (0.74 to 0.82)	6.2	5.80 (5.41 to 6.22)	0.400 (-0.005 to 0.787)	1.07 (1.00 to 1.15)
Austria	2021	Symptoms, signs and abnormal clinical and laboratory findings, n.e.c.	70,400	7.86	88,500 (82,200 to 95,300)	9.88 (9.17 to 10.6)	-18,100 (-24,800 to -11,700)	-2.02 (-2.77 to -1.31)	0.80 (0.74 to 0.86)	6	5.80 (5.26 to 6.40)	0.200 (-0.408 to 0.745)	1.03 (0.94 to 1.14)
Austria	2020	Injury, poisoning and other consequences of external causes	213,000	23.9	259,000 (251,000 to 267,000)	29.0 (28.1 to 29.9)	-45,600 (-53,700 to -37,700)	-5.11 (-6.02 to -4.22)	0.82 (0.80 to 0.85)	7.4	7.60 (7.40 to 7.81)	-0.199 (-0.409 to 0.004)	0.97 (0.95 to 1.00)
Austria	2021	Injury, poisoning and other consequences of external causes	214,000	23.9	260,000 (249,000 to 272,000)	29.0 (27.8 to 30.3)	-46,000 (-57,400 to -34,900)	-5.14 (-6.41 to -3.90)	0.82 (0.79 to 0.86)	7.5	7.60 (7.40 to 7.81)	-0.099 (-0.308 to 0.105)	0.99 (0.96 to 1.01)
Austria	2020	Factors influencing health status and contact with health services	12,800	1.43	11,600 (10,600 to 12,700)	1.30 (1.19 to 1.42)	1,170 (75 to 2,180)	0.131 (0.008 to 0.245)	1.10 (1.01 to 1.21)	4.1	3.66 (3.40 to 3.93)	0.442 (0.166 to 0.698)	1.12 (1.04 to 1.21)
Austria	2021	Factors influencing health status and contact with health services	10,200	1.14	11,600 (10,600 to 12,700)	1.30 (1.19 to 1.42)	-1,450 (-2,530 to -441)	-0.162 (-0.283 to -0.049)	0.88 (0.80 to 0.96)	4.1	3.60 (3.35 to 3.87)	0.500 (0.229 to 0.750)	1.14 (1.06 to 1.22)
Belgium	2020	All causes	1,600,000	139	1,930,000 (1,910,000 to 1,950,000)	167 (166 to 169)	-325,000 (-345,000 to -306,000)	-28.2 (-29.9 to -26.5)	0.83 (0.82 to 0.84)				

Location	Year	Condition	Observed hospitalizations	Observed hospitalizations per 1,000 population	Expected hospitalizations	Expected hospitalizations per 1,000 population	Difference in hospitalizations	Difference in rate	Hospitalizations observed-to-expected ratio	Observed length of stay	Expected length of stay	Difference in length of stay	Length of stay observed-to-expected ratio
Belgium	2021	All causes	1,750,000	151	1,940,000 (1,910,000 to 1,960,000)	167 (165 to 170)	-186,000 (-214,000 to -158,000)	-16.1 (-18.5 to -13.7)	0.90 (0.89 to 0.92)				
Belgium	2020	Infectious and parasitic diseases	42,400	3.68	60,800 (55,200 to 66,900)	5.27 (4.78 to 5.80)	-18,300 (-24,500 to -12,800)	-1.59 (-2.12 to -1.11)	0.70 (0.63 to 0.77)	8.9	7.83 (7.67 to 8.00)	1.07 (0.895 to 1.24)	1.14 (1.11 to 1.16)
Belgium	2021	Infectious and parasitic diseases	47,600	4.11	61,000 (53,200 to 69,900)	5.27 (4.59 to 6.04)	-13,400 (-22,300 to -5,660)	-1.16 (-1.92 to -0.488)	0.78 (0.68 to 0.89)	7.9	7.83 (7.67 to 8.00)	0.067 (-0.104 to 0.235)	1.01 (0.99 to 1.03)
Belgium	2020	Neoplasms	111,000	9.65	126,000 (123,000 to 129,000)	10.9 (10.7 to 11.2)	-14,900 (-18,100 to -11,500)	-1.29 (-1.57 to -1.000)	0.88 (0.86 to 0.91)	7.1	7.25 (7.12 to 7.39)	-0.152 (-0.288 to -0.013)	0.98 (0.96 to 1.00)
Belgium	2021	Neoplasms	118,000	10.2	127,000 (122,000 to 131,000)	10.9 (10.5 to 11.3)	-8,720 (-13,500 to -4,240)	-0.753 (-1.16 to -0.365)	0.93 (0.90 to 0.97)	6.6	7.11 (6.92 to 7.30)	-0.507 (-0.695 to -0.319)	0.93 (0.90 to 0.95)
Belgium	2020	Diseases of the blood and bloodforming organs	15,100	1.31	16,900 (16,300 to 17,500)	1.47 (1.41 to 1.52)	-1,760 (-2,380 to -1,170)	-0.153 (-0.206 to -0.101)	0.90 (0.86 to 0.93)	7.4	7.49 (7.22 to 7.76)	-0.088 (-0.368 to 0.178)	0.99 (0.95 to 1.02)
Belgium	2021	Diseases of the blood and bloodforming organs	16,200	1.40	17,000 (16,400 to 17,600)	1.47 (1.41 to 1.52)	-805 (-1,420 to -216)	-0.069 (-0.123 to -0.019)	0.95 (0.92 to 0.99)	7.1	7.49 (7.22 to 7.76)	-0.388 (-0.657 to -0.132)	0.95 (0.92 to 0.98)
Belgium	2020	Endocrine, nutritional and metabolic diseases	45,900	3.97	56,300 (53,700 to 59,100)	4.88 (4.65 to 5.12)	-10,500 (-13,200 to -7,780)	-0.907 (-1.15 to -0.674)	0.81 (0.78 to 0.86)	6.1	5.78 (5.53 to 6.04)	0.325 (0.065 to 0.572)	1.06 (1.01 to 1.10)
Belgium	2021	Endocrine, nutritional and metabolic diseases	55,700	4.8	56,600 (52,800 to 60,600)	4.88 (4.56 to 5.23)	-899 (-4,870 to 2,920)	-0.078 (-0.420 to 0.252)	0.98 (0.92 to 1.06)	5.4	5.65 (5.31 to 6.02)	-0.253 (-0.616 to 0.092)	0.96 (0.90 to 1.02)
Belgium	2020	Mental and behavioural disorders	26,300	2.28	34,200 (32,800 to 35,800)	2.97 (2.84 to 3.10)	-7,930 (-9,480 to -6,420)	-0.687 (-0.822 to -0.556)	0.77 (0.74 to 0.80)	9.3	8.74 (8.42 to 9.08)	0.561 (0.222 to 0.887)	1.06 (1.02 to 1.11)
Belgium	2021	Mental and behavioural disorders	27,700	2.39	33,600 (31,600 to 35,800)	2.90 (2.73 to 3.09)	-5,910 (-8,050 to -3,850)	-0.510 (-0.694 to -0.333)	0.82 (0.78 to 0.88)	9.1	8.58 (8.13 to 9.05)	0.519 (0.057 to 0.960)	1.06 (1.01 to 1.12)
Belgium	2020	Diseases of the nervous system	105,000	9.07	144,000 (133,000 to 157,000)	12.5 (11.5 to 13.6)	-39,700 (-52,200 to -28,300)	-3.44 (-4.53 to -2.45)	0.72 (0.67 to 0.79)	4.3	3.80 (3.70 to 3.90)	0.498 (0.397 to 0.596)	1.13 (1.10 to 1.16)
Belgium	2021	Diseases of the nervous system	124,000	10.7	151,000 (134,000 to 170,000)	13.0 (11.6 to 14.7)	-27,000 (-46,000 to -10,200)	-2.33 (-3.97 to -0.880)	0.82 (0.73 to 0.92)	3.8	3.61 (3.48 to 3.75)	0.186 (0.051 to 0.315)	1.05 (1.01 to 1.09)
Belgium	2020	Diseases of the eye and adnexa	8,990	0.779	12,400 (11,800 to 13,000)	1.07 (1.02 to 1.13)	-3,370 (-4,020 to -2,770)	-0.292 (-0.348 to -0.240)	0.73 (0.69 to 0.76)	2	1.90 (1.76 to 2.05)	0.100 (-0.053 to 0.241)	1.05 (0.97 to 1.14)
Belgium	2021	Diseases of the eye and adnexa	9,860	0.851	12,200 (11,300 to 13,100)	1.05 (0.978 to 1.13)	-2,310 (-3,210 to -1,460)	-0.199 (-0.277 to -0.126)	0.81 (0.75 to 0.87)	1.9	1.90 (1.71 to 2.12)	0.000 (-0.213 to 0.197)	1.00 (0.90 to 1.12)
Belgium	2020	Diseases of the ear and mastoid process	8,540	0.74	12,000 (11,600 to 12,400)	1.04 (1.01 to 1.07)	-3,450 (-3,850 to -3,050)	-0.299 (-0.333 to -0.265)	0.71 (0.69 to 0.74)	3.1	3.00 (2.82 to 3.19)	0.100 (-0.088 to 0.280)	1.03 (0.97 to 1.10)
Belgium	2021	Diseases of the ear and mastoid process	10,000	0.867	12,000 (11,700 to 12,400)	1.04 (1.01 to 1.07)	-2,000 (-2,400 to -1,610)	-0.172 (-0.207 to -0.139)	0.83 (0.81 to 0.86)	2.8	3.00 (2.75 to 3.27)	-0.200 (-0.467 to 0.048)	0.93 (0.86 to 1.02)
Belgium	2020	Diseases of the circulatory system	183,000	15.9	218,000 (214,000 to 222,000)	18.9 (18.5 to 19.2)	-34,800 (-38,800 to -30,900)	-3.01 (-3.36 to -2.68)	0.84 (0.83 to 0.86)	7.1	7.20 (6.96 to 7.45)	-0.100 (-0.355 to 0.147)	0.99 (0.95 to 1.02)
Belgium	2021	Diseases of the circulatory system	198,000	17.1	217,000 (212,000 to 223,000)	18.7 (18.3 to 19.2)	-19,600 (-25,200 to -14,200)	-1.69 (-2.18 to -1.22)	0.91 (0.89 to 0.93)	6.9	7.20 (6.86 to 7.56)	-0.300 (-0.656 to 0.041)	0.96 (0.91 to 1.01)
Belgium	2020	Diseases of the respiratory system	143,000	12.4	155,000 (146,000 to 164,000)	13.4 (12.6 to 14.2)	-11,800 (-21,100 to -2,760)	-1.02 (-1.83 to -0.239)	0.92 (0.87 to 0.98)	8.7	7.40 (7.08 to 7.74)	1.30 (0.970 to 1.62)	1.18 (1.13 to 1.23)

Location	Year	Condition	Observed hospitalizations	Observed hospitalizations per 1,000 population	Expected hospitalizations	Expected hospitalizations per 1,000 population	Difference in hospitalizations	Difference in rate	Hospitalizations observed-to-expected ratio	Observed length of stay	Expected length of stay	Difference in length of stay	Length of stay observed-to-expected ratio
Belgium	2021	Diseases of the respiratory system	150,000	12.9	155,000 (146,000 to 165,000)	13.4 (12.6 to 14.2)	-5,370 (-14,800 to 3,600)	-0.463 (-1.28 to 0.310)	0.97 (0.91 to 1.02)	7.8	7.40 (6.95 to 7.88)	0.400 (-0.075 to 0.853)	1.05 (0.99 to 1.12)
Belgium	2020	Diseases of the digestive system	155,000	13.4	188,000 (184,000 to 192,000)	16.3 (15.9 to 16.7)	-33,000 (-37,300 to -28,800)	-2.86 (-3.23 to -2.49)	0.82 (0.81 to 0.84)	5.1	4.93 (4.80 to 5.06)	0.170 (0.040 to 0.298)	1.03 (1.01 to 1.06)
Belgium	2021	Diseases of the digestive system	170,000	14.7	189,000 (185,000 to 193,000)	16.3 (15.9 to 16.7)	-18,700 (-22,900 to -14,600)	-1.61 (-1.97 to -1.26)	0.90 (0.88 to 0.92)	4.9	4.86 (4.68 to 5.04)	0.040 (-0.146 to 0.215)	1.01 (0.97 to 1.05)
Belgium	2020	Diseases of the skin and subcutaneous tissue	12,900	1.12	18,900 (18,100 to 19,700)	1.63 (1.57 to 1.71)	-5,960 (-6,780 to -5,180)	-0.516 (-0.587 to -0.449)	0.68 (0.66 to 0.71)	6.9	6.45 (6.30 to 6.60)	0.453 (0.295 to 0.602)	1.07 (1.04 to 1.10)
Belgium	2021	Diseases of the skin and subcutaneous tissue	14,400	1.25	19,600 (18,400 to 20,800)	1.69 (1.59 to 1.80)	-5,140 (-6,340 to -4,000)	-0.444 (-0.547 to -0.345)	0.74 (0.70 to 0.78)	6.4	6.34 (6.18 to 6.49)	0.063 (-0.095 to 0.221)	1.01 (0.99 to 1.04)
Belgium	2020	Diseases of musculoskeletal system and connective tissue	128,000	11.1	169,000 (164,000 to 174,000)	14.6 (14.2 to 15.1)	-41,300 (-46,100 to -36,600)	-3.58 (-4.00 to -3.17)	0.76 (0.73 to 0.78)	5.6	5.60 (5.45 to 5.75)	0.004 (-0.147 to 0.150)	1.00 (0.97 to 1.03)
Belgium	2021	Diseases of musculoskeletal system and connective tissue	150,000	13.0	170,000 (165,000 to 175,000)	14.6 (14.2 to 15.1)	-19,700 (-24,500 to -15,000)	-1.70 (-2.11 to -1.29)	0.88 (0.86 to 0.91)	5.2	5.49 (5.29 to 5.71)	-0.295 (-0.507 to -0.085)	0.95 (0.91 to 0.98)
Belgium	2020	Diseases of the genitourinary system	85,800	7.43	104,000 (101,000 to 108,000)	9.04 (8.75 to 9.34)	-18,500 (-22,000 to -15,100)	-1.60 (-1.90 to -1.31)	0.82 (0.80 to 0.85)	4.6	4.50 (4.30 to 4.71)	0.100 (-0.108 to 0.302)	1.02 (0.98 to 1.07)
Belgium	2021	Diseases of the genitourinary system	94,900	8.19	105,000 (101,000 to 108,000)	9.04 (8.75 to 9.34)	-9,840 (-13,300 to -6,450)	-0.849 (-1.14 to -0.557)	0.91 (0.88 to 0.94)	4.3	4.50 (4.22 to 4.79)	-0.200 (-0.492 to 0.074)	0.96 (0.90 to 1.02)
Belgium	2020	Pregnancy, childbirth and the puerperium	131,000	11.4	137,000 (135,000 to 140,000)	11.9 (11.7 to 12.1)	-6,250 (-8,730 to -3,810)	-0.542 (-0.757 to -0.330)	0.95 (0.94 to 0.97)	3.4	3.60 (3.23 to 4.02)	-0.200 (-0.623 to 0.165)	0.94 (0.85 to 1.05)
Belgium	2021	Pregnancy, childbirth and the puerperium	137,000	11.8	135,000 (132,000 to 139,000)	11.7 (11.4 to 12.0)	1,170 (-2,340 to 4,560)	0.101 (-0.202 to 0.393)	1.01 (0.98 to 1.03)	3.4	3.60 (3.08 to 4.20)	-0.200 (-0.801 to 0.309)	0.94 (0.81 to 1.10)
Belgium	2020	Certain conditions originating in the perinatal period	4,280	0.371	5,000 (4,570 to 5,470)	0.433 (0.396 to 0.474)	-719 (-1,180 to -280)	-0.062 (-0.102 to -0.024)	0.86 (0.78 to 0.94)	9.4	8.95 (8.25 to 9.71)	0.451 (-0.316 to 1.14)	1.05 (0.97 to 1.14)
Belgium	2021	Certain conditions originating in the perinatal period	5,000	0.431	5,020 (4,590 to 5,490)	0.433 (0.396 to 0.474)	-27 (-487 to 401)	-0.002 (-0.042 to 0.035)	0.99 (0.91 to 1.09)	8.7	8.95 (8.25 to 9.71)	-0.249 (-1.02 to 0.450)	0.97 (0.90 to 1.05)
Belgium	2020	Congenital malformations, deformations and chromosomal abnormalities	7,420	0.643	9,610 (9,030 to 10,200)	0.833 (0.783 to 0.887)	-2,190 (-2,810 to -1,620)	-0.190 (-0.243 to -0.140)	0.77 (0.73 to 0.82)	4.7	4.43 (4.31 to 4.56)	0.270 (0.140 to 0.393)	1.06 (1.03 to 1.09)
Belgium	2021	Congenital malformations, deformations and chromosomal abnormalities	8,280	0.715	9,650 (8,840 to 10,500)	0.833 (0.763 to 0.910)	-1,370 (-2,250 to -557)	-0.118 (-0.194 to -0.048)	0.86 (0.79 to 0.94)	4.2	4.36 (4.19 to 4.54)	-0.161 (-0.337 to 0.008)	0.96 (0.93 to 1.00)
Belgium	2020	Symptoms, signs and abnormal clinical and laboratory findings, n.e.c.	66,100	5.73	81,700 (77,700 to 85,900)	7.08 (6.74 to 7.44)	-15,600 (-19,700 to -11,600)	-1.35 (-1.71 to -1.00)	0.81 (0.77 to 0.85)	5.1	4.59 (4.10 to 5.15)	0.507 (-0.056 to 1.00)	1.11 (0.99 to 1.24)

Location	Year	Condition	Observed hospitalizations	Observed hospitalizations per 1,000 population	Expected hospitalizations	Expected hospitalizations per 1,000 population	Difference in hospitalizations	Difference in rate	Hospitalizations observed-to-expected ratio	Observed length of stay	Expected length of stay	Difference in length of stay	Length of stay observed-to-expected ratio
Belgium	2021	Symptoms, signs and abnormal clinical and laboratory findings, n.e.c.	72,600	6.27	82,000 (78,100 to 86,200)	7.08 (6.74 to 7.44)	-9,440 (-13,600 to -5,490)	-0.815 (-1.17 to -0.474)	0.88 (0.84 to 0.93)	4.9	4.59 (4.10 to 5.15)	0.307 (-0.257 to 0.807)	1.07 (0.95 to 1.20)
Belgium	2020	Injury, poisoning and other consequences of external causes	155,000	13.4	185,000 (181,000 to 188,000)	16.0 (15.7 to 16.3)	-29,700 (-33,400 to -26,100)	-2.58 (-2.89 to -2.26)	0.84 (0.82 to 0.86)	8.5	8.57 (8.25 to 8.89)	-0.065 (-0.396 to 0.256)	0.99 (0.96 to 1.03)
Belgium	2021	Injury, poisoning and other consequences of external causes	163,000	14.1	185,000 (182,000 to 189,000)	16.0 (15.7 to 16.3)	-22,600 (-26,400 to -18,900)	-1.95 (-2.28 to -1.63)	0.88 (0.86 to 0.90)	8.1	8.57 (8.25 to 8.89)	-0.465 (-0.790 to -0.152)	0.95 (0.91 to 0.98)
Belgium	2020	Factors influencing health status and contact with health services	170,000	14.7	192,000 (164,000 to 223,000)	16.6 (14.2 to 19.3)	-21,700 (-52,700 to 4,820)	-1.88 (-4.56 to 0.417)	0.89 (0.76 to 1.03)	4.7	5.00 (4.12 to 6.06)	-0.300 (-1.37 to 0.582)	0.94 (0.77 to 1.14)
Belgium	2021	Factors influencing health status and contact with health services	180,000	15.5	192,000 (155,000 to 239,000)	16.6 (13.4 to 20.6)	-12,900 (-58,600 to 23,800)	-1.11 (-5.06 to 2.06)	0.93 (0.75 to 1.15)	4.6	5.00 (3.81 to 6.56)	-0.400 (-1.96 to 0.779)	0.92 (0.70 to 1.20)
Canada	2020	All causes	2,700,000	71.1	3,190,000 (3,130,000 to 3,260,000)	84.0 (82.4 to 85.6)	-490,000 (-552,000 to -430,000)	-12.9 (-14.5 to -11.3)	0.85 (0.83 to 0.86)				
Canada	2021	All causes	2,840,000	74.2	3,210,000 (3,150,000 to 3,270,000)	84.0 (82.4 to 85.6)	-376,000 (-438,000 to -315,000)	-9.82 (-11.5 to -8.25)	0.88 (0.87 to 0.90)				
Canada	2020	Infectious and parasitic diseases	66,100	1.74	85,100 (81,300 to 89,000)	2.24 (2.14 to 2.34)	-19,000 (-22,800 to -15,200)	-0.498 (-0.600 to -0.400)	0.78 (0.74 to 0.81)	10	9.20 (8.93 to 9.47)	0.800 (0.526 to 1.06)	1.09 (1.06 to 1.12)
Canada	2021	Infectious and parasitic diseases	71,400	1.87	85,500 (80,300 to 91,200)	2.24 (2.10 to 2.38)	-14,100 (-19,700 to -8,840)	-0.369 (-0.515 to -0.231)	0.83 (0.78 to 0.89)	9.7	9.20 (8.82 to 9.59)	0.500 (0.108 to 0.876)	1.05 (1.01 to 1.10)
Canada	2020	Neoplasms	181,000	4.75	201,000 (197,000 to 205,000)	5.29 (5.19 to 5.39)	-20,600 (-24,400 to -17,000)	-0.543 (-0.642 to -0.446)	0.90 (0.88 to 0.91)	8.1	8.33 (8.12 to 8.54)	-0.226 (-0.439 to -0.018)	0.97 (0.95 to 1.00)
Canada	2021	Neoplasms	184,000	4.82	200,000 (195,000 to 205,000)	5.23 (5.09 to 5.37)	-15,600 (-21,000 to -10,200)	-0.407 (-0.548 to -0.265)	0.92 (0.90 to 0.95)	8.3	8.25 (7.96 to 8.55)	0.048 (-0.256 to 0.344)	1.01 (0.97 to 1.04)
Canada	2020	Diseases of the blood and bloodforming organs	25,700	0.676	29,700 (28,700 to 30,700)	0.781 (0.755 to 0.807)	-3,970 (-4,950 to -3,020)	-0.104 (-0.130 to -0.079)	0.87 (0.84 to 0.89)	7.1	6.71 (6.46 to 6.97)	0.391 (0.141 to 0.641)	1.06 (1.02 to 1.10)
Canada	2021	Diseases of the blood and bloodforming organs	27,600	0.722	29,900 (28,500 to 31,300)	0.781 (0.745 to 0.818)	-2,230 (-3,660 to -862)	-0.058 (-0.096 to -0.023)	0.93 (0.88 to 0.97)	7.1	6.71 (6.46 to 6.97)	0.391 (0.133 to 0.637)	1.06 (1.02 to 1.10)
Canada	2020	Endocrine, nutritional and metabolic diseases	76,500	2.01	86,400 (84,300 to 88,500)	2.27 (2.22 to 2.33)	-9,830 (-12,000 to -7,740)	-0.258 (-0.315 to -0.203)	0.89 (0.86 to 0.91)	7.9	8.00 (7.59 to 8.44)	-0.100 (-0.527 to 0.315)	0.99 (0.94 to 1.04)
Canada	2021	Endocrine, nutritional and metabolic diseases	78,700	2.06	87,900 (84,900 to 91,000)	2.30 (2.22 to 2.38)	-9,190 (-12,300 to -6,190)	-0.240 (-0.321 to -0.162)	0.90 (0.87 to 0.93)	8.1	8.00 (7.42 to 8.63)	0.100 (-0.519 to 0.677)	1.01 (0.94 to 1.09)
Canada	2020	Mental and behavioural disorders	252,000	6.62	265,000 (241,000 to 292,000)	6.97 (6.34 to 7.66)	-13,200 (-40,100 to 10,700)	-0.348 (-1.05 to 0.281)	0.95 (0.86 to 1.04)	19.1	21.50 (16.71 to 27.67)	-2.40 (-8.58 to 2.47)	0.89 (0.69 to 1.15)

Location	Year	Condition	Observed hospitalizations	Observed hospitalizations per 1,000 population	Expected hospitalizations	Expected hospitalizations per 1,000 population	Difference in hospitalizations	Difference in rate	Hospitalizations observed-to-expected ratio	Observed length of stay	Expected length of stay	Difference in length of stay	Length of stay observed-to-expected ratio
Canada	2021	Mental and behavioural disorders	265,000	6.92	277,000 (242,000 to 317,000)	7.25 (6.34 to 8.29)	-12,600 (-53,200 to 23,200)	-0.329 (-1.39 to 0.607)	0.95 (0.83 to 1.10)	19.7	21.50 (15.05 to 30.71)	-1.80 (-11.07 to 4.62)	0.92 (0.64 to 1.31)
Canada	2020	Diseases of the nervous system	54,300	1.43	62,400 (61,100 to 63,700)	1.64 (1.61 to 1.68)	-8,040 (-9,400 to -6,740)	-0.211 (-0.247 to -0.177)	0.87 (0.85 to 0.89)	14.2	13.91 (13.13 to 14.75)	0.285 (-0.545 to 1.07)	1.02 (0.96 to 1.08)
Canada	2021	Diseases of the nervous system	56,500	1.48	63,200 (61,300 to 65,100)	1.65 (1.60 to 1.70)	-6,740 (-8,670 to -4,850)	-0.176 (-0.227 to -0.127)	0.89 (0.87 to 0.92)	14	13.91 (13.13 to 14.75)	0.085 (-0.763 to 0.880)	1.01 (0.95 to 1.07)
Canada	2020	Diseases of the eye and adnexa	4,330	0.114	5,640 (5,160 to 6,170)	0.148 (0.136 to 0.162)	-1,320 (-1,840 to -830)	-0.035 (-0.048 to -0.022)	0.77 (0.70 to 0.84)	3.6	3.50 (3.12 to 3.93)	0.100 (-0.327 to 0.486)	1.03 (0.92 to 1.16)
Canada	2021	Diseases of the eye and adnexa	4,570	0.119	5,480 (4,830 to 6,210)	0.143 (0.126 to 0.162)	-911 (-1,650 to -276)	-0.024 (-0.043 to -0.007)	0.83 (0.73 to 0.94)	3.8	3.50 (2.97 to 4.12)	0.300 (-0.329 to 0.818)	1.09 (0.92 to 1.27)
Canada	2020	Diseases of the ear and mastoid process	6,170	0.162	8,670 (8,400 to 8,950)	0.228 (0.221 to 0.235)	-2,500 (-2,790 to -2,230)	-0.066 (-0.073 to -0.059)	0.71 (0.69 to 0.73)	3.6	3.50 (3.22 to 3.81)	0.100 (-0.216 to 0.385)	1.03 (0.94 to 1.12)
Canada	2021	Diseases of the ear and mastoid process	6,480	0.169	8,510 (8,240 to 8,790)	0.223 (0.215 to 0.230)	-2,030 (-2,300 to -1,770)	-0.053 (-0.060 to -0.046)	0.76 (0.74 to 0.79)	3.9	3.50 (3.11 to 3.94)	0.400 (-0.044 to 0.796)	1.11 (0.99 to 1.26)
Canada	2020	Diseases of the circulatory system	349,000	9.19	398,000 (393,000 to 402,000)	10.5 (10.3 to 10.6)	-48,200 (-53,000 to -43,500)	-1.27 (-1.39 to -1.14)	0.88 (0.87 to 0.89)	7.7	8.00 (7.85 to 8.16)	-0.300 (-0.458 to -0.151)	0.96 (0.94 to 0.98)
Canada	2021	Diseases of the circulatory system	366,000	9.56	400,000 (395,000 to 405,000)	10.5 (10.3 to 10.6)	-34,200 (-39,100 to -29,500)	-0.895 (-1.02 to -0.772)	0.91 (0.90 to 0.93)	8	8.00 (7.79 to 8.22)	0.000 (-0.220 to 0.217)	1.00 (0.97 to 1.03)
Canada	2020	Diseases of the respiratory system	144,000	3.77	281,000 (256,000 to 308,000)	7.38 (6.72 to 8.10)	-137,000 (-164,000 to -112,000)	-3.60 (-4.30 to -2.95)	0.51 (0.47 to 0.56)	8.9	7.50 (7.32 to 7.69)	1.40 (1.21 to 1.58)	1.19 (1.16 to 1.22)
Canada	2021	Diseases of the respiratory system	174,000	4.56	282,000 (247,000 to 322,000)	7.38 (6.47 to 8.41)	-108,000 (-148,000 to -73,200)	-2.82 (-3.88 to -1.92)	0.62 (0.54 to 0.70)	8	7.50 (7.24 to 7.77)	0.500 (0.231 to 0.759)	1.07 (1.03 to 1.10)
Canada	2020	Diseases of the digestive system	285,000	7.51	316,000 (309,000 to 322,000)	8.30 (8.14 to 8.46)	-30,200 (-36,500 to -24,200)	-0.793 (-0.960 to -0.635)	0.90 (0.89 to 0.92)	5.7	5.80 (5.66 to 5.95)	-0.100 (-0.248 to 0.045)	0.98 (0.96 to 1.01)
Canada	2021	Diseases of the digestive system	294,000	7.7	317,000 (311,000 to 324,000)	8.30 (8.14 to 8.46)	-22,900 (-29,100 to -16,600)	-0.598 (-0.762 to -0.433)	0.93 (0.91 to 0.95)	5.9	5.80 (5.60 to 6.01)	0.100 (-0.107 to 0.302)	1.02 (0.98 to 1.05)
Canada	2020	Diseases of the skin and subcutaneous tissue	31,000	0.814	36,400 (34,800 to 38,000)	0.956 (0.915 to 0.998)	-5,400 (-7,030 to -3,880)	-0.142 (-0.185 to -0.102)	0.85 (0.81 to 0.89)	9.3	9.30 (8.88 to 9.74)	0.000 (-0.442 to 0.427)	1.00 (0.95 to 1.05)
Canada	2021	Diseases of the skin and subcutaneous tissue	31,700	0.829	36,600 (34,400 to 38,900)	0.956 (0.899 to 1.02)	-4,830 (-7,150 to -2,670)	-0.126 (-0.187 to -0.070)	0.87 (0.82 to 0.92)	9.7	9.30 (8.71 to 9.93)	0.400 (-0.237 to 0.996)	1.04 (0.98 to 1.11)
Canada	2020	Diseases of musculoskeletal system and connective tissue	154,000	4.06	206,000 (199,000 to 214,000)	5.42 (5.22 to 5.63)	-51,800 (-59,900 to -44,300)	-1.36 (-1.58 to -1.16)	0.75 (0.72 to 0.78)	5.6	5.40 (5.24 to 5.56)	0.201 (0.041 to 0.354)	1.04 (1.01 to 1.07)
Canada	2021	Diseases of musculoskeletal system and connective tissue	156,000	4.07	211,000 (200,000 to 223,000)	5.52 (5.23 to 5.82)	-55,500 (-67,200 to -44,600)	-1.45 (-1.76 to -1.17)	0.74 (0.70 to 0.78)	6	5.30 (5.08 to 5.52)	0.700 (0.475 to 0.918)	1.13 (1.09 to 1.18)
Canada	2020	Diseases of the genitourinary system	140,000	3.68	164,000 (160,000 to 167,000)	4.30 (4.21 to 4.39)	-23,700 (-27,000 to -20,400)	-0.622 (-0.710 to -0.536)	0.86 (0.84 to 0.87)	5.6	5.40 (5.18 to 5.63)	0.200 (-0.035 to 0.421)	1.04 (0.99 to 1.08)

Location	Year	Condition	Observed hospitalizations	Observed hospitalizations per 1,000 population	Expected hospitalizations	Expected hospitalizations per 1,000 population	Difference in hospitalizations	Difference in rate	Hospitalizations observed-to-expected ratio	Observed length of stay	Expected length of stay	Difference in length of stay	Length of stay observed-to-expected ratio
Canada	2021	Diseases of the genitourinary system	143,000	3.74	161,000 (156,000 to 166,000)	4.20 (4.07 to 4.34)	-17,500 (-22,800 to -12,500)	-0.458 (-0.596 to -0.327)	0.89 (0.86 to 0.92)	5.9	5.40 (5.09 to 5.73)	0.500 (0.168 to 0.807)	1.09 (1.03 to 1.16)
Canada	2020	Pregnancy, childbirth and the puerperium	388,000	10.2	408,000 (403,000 to 414,000)	10.7 (10.6 to 10.9)	-20,000 (-25,500 to -14,600)	-0.525 (-0.670 to -0.385)	0.95 (0.94 to 0.96)	2.1	2.20 (2.11 to 2.29)	-0.100 (-0.188 to -0.014)	0.95 (0.92 to 0.99)
Canada	2021	Pregnancy, childbirth and the puerperium	402,000	10.5	405,000 (397,000 to 412,000)	10.6 (10.4 to 10.8)	-3,140 (-10,900 to 4,340)	-0.082 (-0.284 to 0.113)	0.99 (0.97 to 1.01)	2.1	2.20 (2.08 to 2.33)	-0.100 (-0.227 to 0.021)	0.95 (0.90 to 1.01)
Canada	2020	Certain conditions originating in the perinatal period	25,800	0.679	27,600 (26,600 to 28,600)	0.725 (0.698 to 0.753)	-1,760 (-2,800 to -745)	-0.046 (-0.074 to -0.020)	0.94 (0.90 to 0.97)	7.5	7.37 (6.98 to 7.78)	0.132 (-0.285 to 0.522)	1.02 (0.96 to 1.07)
Canada	2021	Certain conditions originating in the perinatal period	27,600	0.721	28,200 (26,800 to 29,800)	0.738 (0.700 to 0.779)	-680 (-2,220 to 806)	-0.018 (-0.058 to 0.021)	0.98 (0.93 to 1.03)	7.3	7.24 (6.71 to 7.81)	0.062 (-0.509 to 0.586)	1.01 (0.93 to 1.09)
Canada	2020	Congenital malformations, deformations and chromosomal abnormalities	11,600	0.304	12,900 (12,500 to 13,200)	0.338 (0.330 to 0.347)	-1,300 (-1,630 to -980)	-0.034 (-0.043 to -0.026)	0.90 (0.88 to 0.92)	7.7	8.00 (7.15 to 8.95)	-0.300 (-1.24 to 0.555)	0.96 (0.86 to 1.08)
Canada	2021	Congenital malformations, deformations and chromosomal abnormalities	11,800	0.309	12,700 (12,200 to 13,100)	0.331 (0.319 to 0.343)	-825 (-1,280 to -387)	-0.022 (-0.033 to -0.010)	0.93 (0.90 to 0.97)	7.4	8.00 (6.83 to 9.38)	-0.600 (-1.95 to 0.562)	0.92 (0.79 to 1.08)
Canada	2020	Symptoms, signs and abnormal clinical and laboratory findings, n.e.c.	142,000	3.73	156,000 (150,000 to 163,000)	4.11 (3.93 to 4.29)	-14,600 (-21,400 to -7,830)	-0.384 (-0.563 to -0.206)	0.91 (0.87 to 0.95)	7.1	6.97 (6.64 to 7.31)	0.132 (-0.209 to 0.456)	1.02 (0.97 to 1.07)
Canada	2021	Symptoms, signs and abnormal clinical and laboratory findings, n.e.c.	149,000	3.89	154,000 (145,000 to 164,000)	4.04 (3.80 to 4.30)	-5,530 (-15,500 to 3,910)	-0.145 (-0.406 to 0.102)	0.96 (0.91 to 1.03)	7.4	6.97 (6.64 to 7.31)	0.432 (0.090 to 0.769)	1.06 (1.01 to 1.12)
Canada	2020	Injury, poisoning and other consequences of external causes	235,000	6.17	264,000 (259,000 to 270,000)	6.95 (6.81 to 7.09)	-29,600 (-35,100 to -24,300)	-0.779 (-0.923 to -0.638)	0.89 (0.87 to 0.91)	8.8	9.00 (8.67 to 9.34)	-0.200 (-0.540 to 0.120)	0.98 (0.94 to 1.01)
Canada	2021	Injury, poisoning and other consequences of external causes	247,000	6.46	266,000 (260,000 to 271,000)	6.95 (6.81 to 7.09)	-18,800 (-24,200 to -13,300)	-0.490 (-0.633 to -0.347)	0.93 (0.91 to 0.95)	9	9.00 (8.54 to 9.48)	0.000 (-0.480 to 0.456)	1.00 (0.95 to 1.05)
Canada	2020	Factors influencing health status and contact with health services	133,000	3.49	149,000 (146,000 to 152,000)	3.92 (3.85 to 3.99)	-16,100 (-18,800 to -13,500)	-0.424 (-0.494 to -0.354)	0.89 (0.88 to 0.91)	13	11.99 (11.25 to 12.79)	1.01 (0.196 to 1.74)	1.08 (1.02 to 1.15)
Canada	2021	Factors influencing health status and contact with health services	141,000	3.68	149,000 (145,000 to 153,000)	3.89 (3.79 to 3.99)	-8,300 (-12,200 to -4,490)	-0.217 (-0.320 to -0.117)	0.94 (0.92 to 0.97)	13	11.99 (11.25 to 12.79)	1.01 (0.196 to 1.73)	1.08 (1.02 to 1.15)
Switzerland	2020	All causes	1,370,000	159	1,460,000 (1,430,000 to 1,490,000)	169 (166 to 173)	-88,600 (-120,000 to -58,800)	-10.3 (-13.8 to -6.80)	0.94 (0.92 to 0.96)				
Switzerland	2021	All causes	1,440,000	165	1,470,000 (1,440,000 to 1,500,000)	169 (166 to 173)	-31,400 (-62,200 to -1,080)	-3.61 (-7.15 to -0.124)	0.98 (0.96 to 1.00)				

Location	Year	Condition	Observed hospitalizations	Observed hospitalizations per 1,000 population	Expected hospitalizations	Expected hospitalizations per 1,000 population	Difference in hospitalizations	Difference in rate	Hospitalizations observed-to-expected ratio	Observed length of stay	Expected length of stay	Difference in length of stay	Length of stay observed-to-expected ratio
Switzerland	2020	Infectious and parasitic diseases	33,500	3.88	37,300 (33,000 to 42,100)	4.32 (3.82 to 4.87)	-3,780 (-8,550 to 458)	-0.438 (-0.990 to 0.053)	0.90 (0.80 to 1.01)	8.5	7.80 (7.46 to 8.16)	0.700 (0.342 to 1.04)	1.09 (1.04 to 1.14)
Switzerland	2021	Infectious and parasitic diseases	35,500	4.08	37,600 (33,300 to 42,400)	4.32 (3.82 to 4.87)	-2,080 (-7,030 to 2,150)	-0.239 (-0.807 to 0.247)	0.94 (0.83 to 1.06)	8	7.80 (7.32 to 8.31)	0.200 (-0.315 to 0.681)	1.03 (0.96 to 1.09)
Switzerland	2020	Neoplasms	123,000	14.3	130,000 (125,000 to 136,000)	15.1 (14.4 to 15.8)	-7,180 (-13,300 to -1,600)	-0.832 (-1.54 to -0.185)	0.94 (0.90 to 0.99)	7.9	7.91 (7.74 to 8.07)	-0.006 (-0.172 to 0.156)	1.00 (0.98 to 1.02)
Switzerland	2021	Neoplasms	128,000	14.8	131,000 (123,000 to 140,000)	15.1 (14.2 to 16.1)	-2,930 (-11,300 to 4,860)	-0.336 (-1.30 to 0.558)	0.98 (0.92 to 1.04)	7.6	7.81 (7.59 to 8.04)	-0.213 (-0.445 to 0.013)	0.97 (0.94 to 1.00)
Switzerland	2020	Diseases of the blood and bloodforming organs	5,810	0.672	6,600 (6,030 to 7,220)	0.764 (0.698 to 0.836)	-787 (-1,400 to -210)	-0.091 (-0.162 to -0.024)	0.88 (0.81 to 0.97)	7.2	7.00 (6.74 to 7.26)	0.203 (-0.056 to 0.458)	1.03 (0.99 to 1.07)
Switzerland	2021	Diseases of the blood and bloodforming organs	6,000	0.69	6,650 (5,850 to 7,550)	0.764 (0.672 to 0.868)	-642 (-1,530 to 168)	-0.074 (-0.176 to 0.019)	0.90 (0.80 to 1.03)	7	6.90 (6.54 to 7.27)	0.105 (-0.265 to 0.454)	1.02 (0.96 to 1.07)
Switzerland	2020	Endocrine, nutritional and metabolic diseases	21,200	2.45	24,600 (23,500 to 25,800)	2.85 (2.72 to 2.99)	-3,430 (-4,630 to -2,290)	-0.398 (-0.536 to -0.265)	0.86 (0.82 to 0.90)	6.9	6.92 (6.69 to 7.17)	-0.024 (-0.269 to 0.213)	1.00 (0.96 to 1.03)
Switzerland	2021	Endocrine, nutritional and metabolic diseases	23,200	2.67	25,500 (23,800 to 27,200)	2.93 (2.74 to 3.13)	-2,270 (-4,050 to -624)	-0.260 (-0.466 to -0.072)	0.91 (0.85 to 0.97)	6.8	6.75 (6.43 to 7.09)	0.048 (-0.294 to 0.373)	1.01 (0.96 to 1.06)
Switzerland	2020	Mental and behavioural disorders	104,000	12	106,000 (101,000 to 110,000)	12.2 (11.7 to 12.8)	-2,080 (-6,660 to 2,330)	-0.241 (-0.771 to 0.270)	0.98 (0.94 to 1.02)	27.5	26.11 (25.01 to 27.25)	1.39 (0.264 to 2.50)	1.05 (1.01 to 1.10)
Switzerland	2021	Mental and behavioural disorders	109,000	12.5	107,000 (100,000 to 113,000)	12.2 (11.5 to 13.0)	2,590 (-3,910 to 8,880)	0.298 (-0.449 to 1.02)	1.02 (0.97 to 1.09)	26.9	25.52 (24.03 to 27.11)	1.38 (-0.196 to 2.87)	1.05 (0.99 to 1.12)
Switzerland	2020	Diseases of the nervous system	36,200	4.19	38,400 (36,800 to 40,100)	4.45 (4.26 to 4.65)	-2,230 (-3,920 to -603)	-0.258 (-0.454 to -0.070)	0.94 (0.90 to 0.98)	12.9	12.80 (12.18 to 13.45)	0.100 (-0.552 to 0.715)	1.01 (0.96 to 1.06)
Switzerland	2021	Diseases of the nervous system	37,800	4.34	38,700 (37,100 to 40,400)	4.45 (4.26 to 4.65)	-927 (-2,580 to 726)	-0.106 (-0.296 to 0.083)	0.98 (0.94 to 1.02)	12.2	12.80 (11.94 to 13.73)	-0.600 (-1.52 to 0.256)	0.95 (0.89 to 1.02)
Switzerland	2020	Diseases of the eye and adnexa	10,600	1.23	12,100 (11,600 to 12,700)	1.40 (1.34 to 1.47)	-1,490 (-2,090 to -926)	-0.173 (-0.242 to -0.107)	0.88 (0.84 to 0.92)	2.3	2.38 (2.29 to 2.47)	-0.077 (-0.174 to 0.014)	0.97 (0.93 to 1.01)
Switzerland	2021	Diseases of the eye and adnexa	11,200	1.29	12,500 (11,200 to 13,900)	1.44 (1.29 to 1.60)	-1,280 (-2,690 to 5)	-0.147 (-0.309 to 0.001)	0.90 (0.81 to 1.00)	2.2	2.40 (2.28 to 2.52)	-0.196 (-0.320 to -0.075)	0.92 (0.87 to 0.97)
Switzerland	2020	Diseases of the ear and mastoid process	6,740	0.78	7,710 (7,210 to 8,250)	0.893 (0.835 to 0.955)	-971 (-1,510 to -475)	-0.112 (-0.175 to -0.055)	0.87 (0.82 to 0.93)	3.5	3.40 (3.22 to 3.59)	0.100 (-0.092 to 0.274)	1.03 (0.97 to 1.08)
Switzerland	2021	Diseases of the ear and mastoid process	7,280	0.836	7,770 (7,060 to 8,550)	0.893 (0.812 to 0.982)	-492 (-1,250 to 217)	-0.057 (-0.143 to 0.025)	0.94 (0.85 to 1.03)	3.4	3.40 (3.15 to 3.67)	0.000 (-0.275 to 0.249)	1.00 (0.93 to 1.08)
Switzerland	2020	Diseases of the circulatory system	148,000	17.1	160,000 (156,000 to 163,000)	18.5 (18.1 to 18.9)	-11,700 (-15,300 to -8,190)	-1.36 (-1.77 to -0.948)	0.93 (0.91 to 0.95)	8.3	8.62 (8.40 to 8.84)	-0.319 (-0.549 to -0.102)	0.96 (0.94 to 0.99)
Switzerland	2021	Diseases of the circulatory system	154,000	17.7	161,000 (157,000 to 164,000)	18.5 (18.1 to 18.9)	-6,700 (-10,200 to -3,220)	-0.770 (-1.18 to -0.370)	0.96 (0.94 to 0.98)	8.3	8.62 (8.40 to 8.84)	-0.319 (-0.541 to -0.097)	0.96 (0.94 to 0.99)

Location	Year	Condition	Observed hospitalizations	Observed hospitalizations per 1,000 population	Expected hospitalizations	Expected hospitalizations per 1,000 population	Difference in hospitalizations	Difference in rate	Hospitalizations observed-to-expected ratio	Observed length of stay	Expected length of stay	Difference in length of stay	Length of stay observed-to-expected ratio
Switzerland	2020	Diseases of the respiratory system	90,900	10.5	95,000 (91,100 to 99,100)	11.0 (10.5 to 11.5)	-4,090 (-8,190 to -175)	-0.473 (-0.948 to -0.020)	0.96 (0.92 to 1.00)	7.5	6.41 (6.25 to 6.57)	1.09 (0.938 to 1.25)	1.17 (1.14 to 1.20)
Switzerland	2021	Diseases of the respiratory system	94,000	10.8	96,100 (92,000 to 100,000)	11.0 (10.6 to 11.5)	-2,180 (-6,310 to 1,840)	-0.250 (-0.725 to 0.212)	0.98 (0.94 to 1.02)	7.7	6.32 (6.10 to 6.54)	1.38 (1.16 to 1.61)	1.22 (1.18 to 1.26)
Switzerland	2020	Diseases of the digestive system	112,000	12.9	120,000 (116,000 to 125,000)	13.9 (13.4 to 14.5)	-8,510 (-13,400 to -3,760)	-0.985 (-1.55 to -0.435)	0.93 (0.89 to 0.97)	5.4	5.57 (5.42 to 5.72)	-0.168 (-0.315 to -0.023)	0.97 (0.94 to 1.00)
Switzerland	2021	Diseases of the digestive system	118,000	13.6	122,000 (116,000 to 128,000)	14.0 (13.4 to 14.7)	-3,880 (-9,880 to 1,870)	-0.445 (-1.14 to 0.215)	0.97 (0.92 to 1.02)	5.3	5.61 (5.33 to 5.91)	-0.314 (-0.607 to -0.035)	0.94 (0.90 to 0.99)
Switzerland	2020	Diseases of the skin and subcutaneous tissue	13,800	1.59	16,800 (16,200 to 17,500)	1.95 (1.87 to 2.03)	-3,080 (-3,790 to -2,400)	-0.357 (-0.438 to -0.278)	0.82 (0.78 to 0.85)	6.4	6.52 (6.21 to 6.84)	-0.117 (-0.437 to 0.193)	0.98 (0.94 to 1.03)
Switzerland	2021	Diseases of the skin and subcutaneous tissue	14,400	1.65	17,000 (16,300 to 17,700)	1.95 (1.87 to 2.03)	-2,580 (-3,300 to -1,910)	-0.297 (-0.379 to -0.219)	0.85 (0.81 to 0.88)	6.6	6.34 (5.92 to 6.79)	0.262 (-0.202 to 0.688)	1.04 (0.97 to 1.12)
Switzerland	2020	Diseases of musculoskeletal system and connective tissue	165,000	19.1	180,000 (172,000 to 189,000)	20.9 (19.9 to 21.9)	-15,200 (-24,400 to -6,460)	-1.76 (-2.83 to -0.748)	0.92 (0.87 to 0.96)	7	7.02 (6.85 to 7.19)	-0.016 (-0.183 to 0.150)	1.00 (0.97 to 1.02)
Switzerland	2021	Diseases of musculoskeletal system and connective tissue	177,000	20.3	183,000 (172,000 to 195,000)	21.0 (19.8 to 22.4)	-6,160 (-17,900 to 4,770)	-0.707 (-2.06 to 0.548)	0.97 (0.91 to 1.03)	6.9	6.93 (6.70 to 7.17)	-0.034 (-0.270 to 0.197)	1.00 (0.96 to 1.03)
Switzerland	2020	Diseases of the genitourinary system	76,300	8.83	82,700 (79,100 to 86,400)	9.57 (9.15 to 10.0)	-6,380 (-10,100 to -2,870)	-0.739 (-1.17 to -0.333)	0.92 (0.88 to 0.96)	4	4.10 (3.89 to 4.32)	-0.100 (-0.322 to 0.109)	0.98 (0.93 to 1.03)
Switzerland	2021	Diseases of the genitourinary system	81,200	9.33	83,300 (78,200 to 88,700)	9.57 (8.99 to 10.2)	-2,120 (-7,410 to 2,970)	-0.243 (-0.852 to 0.341)	0.97 (0.92 to 1.04)	3.9	4.10 (3.81 to 4.42)	-0.200 (-0.523 to 0.092)	0.95 (0.88 to 1.02)
Switzerland	2020	Pregnancy, childbirth and the puerperium	98,200	11.4	106,000 (102,000 to 110,000)	12.2 (11.8 to 12.7)	-7,470 (-11,400 to -3,760)	-0.865 (-1.32 to -0.436)	0.93 (0.90 to 0.96)	3.7	3.92 (3.82 to 4.02)	-0.220 (-0.322 to -0.121)	0.94 (0.92 to 0.97)
Switzerland	2021	Pregnancy, childbirth and the puerperium	103,000	11.8	106,000 (103,000 to 110,000)	12.2 (11.8 to 12.7)	-3,440 (-7,400 to 372)	-0.395 (-0.850 to 0.043)	0.97 (0.93 to 1.00)	3.7	3.84 (3.70 to 3.99)	-0.141 (-0.284 to -0.003)	0.96 (0.93 to 1.00)
Switzerland	2020	Certain conditions originating in the perinatal period	30,300	3.5	30,800 (28,300 to 33,400)	3.56 (3.28 to 3.87)	-497 (-3,100 to 1,980)	-0.058 (-0.359 to 0.229)	0.98 (0.91 to 1.07)	5.8	6.02 (5.84 to 6.21)	-0.225 (-0.413 to -0.042)	0.96 (0.93 to 0.99)
Switzerland	2021	Certain conditions originating in the perinatal period	32,700	3.76	31,000 (27,600 to 34,900)	3.56 (3.17 to 4.01)	1,700 (-2,240 to 5,140)	0.196 (-0.257 to 0.590)	1.05 (0.94 to 1.19)	5.7	5.97 (5.61 to 6.35)	-0.268 (-0.651 to 0.089)	0.96 (0.90 to 1.02)
Switzerland	2020	Congenital malformations, deformations and chromosomal abnormalities	9,030	1.05	10,400 (10,000 to 10,700)	1.20 (1.16 to 1.24)	-1,340 (-1,720 to -965)	-0.155 (-0.199 to -0.112)	0.87 (0.84 to 0.90)	6.2	6.01 (5.79 to 6.23)	0.193 (-0.020 to 0.407)	1.03 (1.00 to 1.07)
Switzerland	2021	Congenital malformations, deformations and chromosomal abnormalities	9,780	1.12	10,400 (10,100 to 10,800)	1.20 (1.16 to 1.24)	-672 (-1,060 to -310)	-0.077 (-0.122 to -0.036)	0.94 (0.90 to 0.97)	6.2	5.91 (5.62 to 6.22)	0.285 (-0.021 to 0.584)	1.05 (1.00 to 1.10)

Location	Year	Condition	Observed hospitalizations	Observed hospitalizations per 1,000 population	Expected hospitalizations	Expected hospitalizations per 1,000 population	Difference in hospitalizations	Difference in rate	Hospitalizations observed-to-expected ratio	Observed length of stay	Expected length of stay	Difference in length of stay	Length of stay observed-to-expected ratio
Switzerland	2020	Symptoms, signs and abnormal clinical and laboratory findings, n.e.c.	43,400	5.03	44,600 (42,200 to 47,100)	5.16 (4.88 to 5.45)	-1,110 (-3,530 to 1,280)	-0.128 (-0.409 to 0.149)	0.98 (0.92 to 1.03)	6.8	7.40 (5.96 to 9.19)	-0.600 (-2.36 to 0.864)	0.92 (0.74 to 1.15)
Switzerland	2021	Symptoms, signs and abnormal clinical and laboratory findings, n.e.c.	46,900	5.39	44,900 (41,500 to 48,500)	5.16 (4.77 to 5.58)	1,990 (-1,650 to 5,400)	0.229 (-0.189 to 0.621)	1.04 (0.97 to 1.13)	6.3	7.40 (5.45 to 10.05)	-1.10 (-3.69 to 0.883)	0.85 (0.63 to 1.16)
Switzerland	2020	Injury, poisoning and other consequences of external causes	178,000	20.6	190,000 (180,000 to 200,000)	22.0 (20.8 to 23.2)	-11,500 (-21,600 to -1,850)	-1.33 (-2.50 to -0.214)	0.94 (0.89 to 0.99)	6.9	7.19 (6.98 to 7.41)	-0.289 (-0.505 to -0.078)	0.96 (0.93 to 0.99)
Switzerland	2021	Injury, poisoning and other consequences of external causes	184,000	21.1	191,000 (181,000 to 202,000)	22.0 (20.8 to 23.2)	-7,710 (-18,100 to 2,140)	-0.886 (-2.08 to 0.246)	0.96 (0.91 to 1.01)	6.8	7.19 (6.98 to 7.41)	-0.389 (-0.605 to -0.178)	0.95 (0.92 to 0.97)
Switzerland	2020	Factors influencing health status and contact with health services	65,400	7.57	66,500 (61,800 to 71,600)	7.70 (7.15 to 8.28)	-1,060 (-6,080 to 3,740)	-0.123 (-0.703 to 0.432)	0.98 (0.92 to 1.06)	4.6	5.04 (4.23 to 6.00)	-0.438 (-1.40 to 0.367)	0.91 (0.77 to 1.09)
Switzerland	2021	Factors influencing health status and contact with health services	66,500	7.64	66,600 (56,500 to 78,500)	7.65 (6.49 to 9.02)	-117 (-11,900 to 9,840)	-0.013 (-1.37 to 1.13)	1.00 (0.85 to 1.17)	3.9	5.52 (3.74 to 8.15)	-1.62 (-4.26 to 0.164)	0.71 (0.48 to 1.04)
Chile	2020	All causes	1,280,000	65.6	1,650,000 (1,600,000 to 1,700,000)	84.7 (82.2 to 87.2)	-370,000 (-419,000 to -321,000)	-19.0 (-21.5 to -16.5)	0.78 (0.75 to 0.80)				
Chile	2021	All causes	1,380,000	70	1,650,000 (1,580,000 to 1,720,000)	83.7 (80.2 to 87.3)	-269,000 (-341,000 to -201,000)	-13.7 (-17.3 to -10.2)	0.84 (0.80 to 0.87)				
Chile	2020	Infectious and parasitic diseases	25,000	1.29	37,700 (34,400 to 41,200)	1.94 (1.77 to 2.12)	-12,600 (-16,100 to -9,400)	-0.650 (-0.830 to -0.483)	0.66 (0.61 to 0.73)	10.4	9.30 (8.44 to 10.25)	1.10 (0.140 to 1.96)	1.12 (1.01 to 1.23)
Chile	2021	Infectious and parasitic diseases	23,600	1.2	38,100 (33,600 to 43,300)	1.94 (1.71 to 2.20)	-14,500 (-19,600 to -9,970)	-0.737 (-0.995 to -0.507)	0.62 (0.55 to 0.70)	11.6	9.61 (8.37 to 11.03)	1.99 (0.584 to 3.22)	1.21 (1.05 to 1.38)
Chile	2020	Neoplasms	102,000	5.23	132,000 (128,000 to 136,000)	6.80 (6.60 to 7.01)	-30,600 (-34,700 to -26,800)	-1.57 (-1.78 to -1.38)	0.77 (0.75 to 0.79)	6.7	6.53 (6.22 to 6.85)	0.172 (-0.148 to 0.480)	1.03 (0.98 to 1.08)
Chile	2021	Neoplasms	116,000	5.89	134,000 (130,000 to 138,000)	6.80 (6.60 to 7.01)	-18,000 (-22,000 to -14,100)	-0.915 (-1.12 to -0.715)	0.87 (0.84 to 0.89)	6.1	6.53 (6.22 to 6.85)	-0.428 (-0.748 to -0.119)	0.93 (0.89 to 0.98)
Chile	2020	Diseases of the blood and bloodforming organs	10,500	0.542	13,500 (12,800 to 14,200)	0.692 (0.655 to 0.731)	-2,930 (-3,690 to -2,230)	-0.151 (-0.190 to -0.115)	0.78 (0.74 to 0.83)	6.4	6.44 (6.09 to 6.80)	-0.038 (-0.413 to 0.317)	0.99 (0.94 to 1.05)
Chile	2021	Diseases of the blood and bloodforming organs	10,500	0.536	14,100 (13,000 to 15,200)	0.715 (0.662 to 0.773)	-3,530 (-4,650 to -2,490)	-0.179 (-0.236 to -0.127)	0.75 (0.69 to 0.81)	7	6.44 (6.09 to 6.80)	0.562 (0.200 to 0.903)	1.09 (1.03 to 1.15)
Chile	2020	Endocrine, nutritional and metabolic diseases	38,200	1.96	47,900 (45,500 to 50,300)	2.46 (2.34 to 2.59)	-9,690 (-12,100 to -7,360)	-0.498 (-0.624 to -0.378)	0.80 (0.76 to 0.84)	7	7.00 (6.28 to 7.80)	0.000 (-0.791 to 0.724)	1.00 (0.90 to 1.12)
Chile	2021	Endocrine, nutritional and metabolic diseases	45,900	2.33	48,800 (44,600 to 53,300)	2.48 (2.27 to 2.71)	-2,900 (-7,460 to 1,280)	-0.147 (-0.379 to 0.065)	0.94 (0.86 to 1.03)	6.5	7.00 (6.01 to 8.15)	-0.500 (-1.67 to 0.497)	0.93 (0.80 to 1.08)

Location	Year	Condition	Observed hospitalizations	Observed hospitalizations per 1,000 population	Expected hospitalizations	Expected hospitalizations per 1,000 population	Difference in hospitalizations	Difference in rate	Hospitalizations observed-to-expected ratio	Observed length of stay	Expected length of stay	Difference in length of stay	Length of stay observed-to-expected ratio
Chile	2020	Mental and behavioural disorders	26,300	1.35	31,200 (28,500 to 34,100)	1.60 (1.46 to 1.75)	-4,870 (-7,840 to -2,200)	-0.250 (-0.403 to -0.113)	0.84 (0.77 to 0.92)	26.6	32.25 (23.80 to 43.70)	-5.65 (-16.90 to 2.95)	0.82 (0.61 to 1.12)
Chile	2021	Mental and behavioural disorders	29,400	1.5	31,500 (28,800 to 34,500)	1.60 (1.46 to 1.75)	-2,080 (-5,050 to 635)	-0.105 (-0.257 to 0.032)	0.93 (0.85 to 1.02)	25.2	32.25 (23.80 to 43.70)	-7.05 (-18.80 to 1.37)	0.78 (0.57 to 1.06)
Chile	2020	Diseases of the nervous system	25,000	1.29	33,600 (31,200 to 36,200)	1.73 (1.61 to 1.86)	-8,610 (-11,200 to -6,240)	-0.443 (-0.577 to -0.321)	0.74 (0.69 to 0.80)	8.2	8.09 (7.28 to 8.99)	0.111 (-0.795 to 0.905)	1.01 (0.91 to 1.12)
Chile	2021	Diseases of the nervous system	28,000	1.43	34,000 (30,600 to 37,700)	1.73 (1.56 to 1.92)	-5,950 (-9,630 to -2,520)	-0.302 (-0.489 to -0.128)	0.83 (0.74 to 0.92)	8.5	8.09 (7.28 to 8.99)	0.411 (-0.503 to 1.23)	1.05 (0.94 to 1.17)
Chile	2020	Diseases of the eye and adnexa	8,000	0.411	12,200 (9,100 to 16,300)	0.626 (0.468 to 0.837)	-4,170 (-8,230 to -1,130)	-0.214 (-0.423 to -0.058)	0.66 (0.49 to 0.88)	2.4	2.53 (2.27 to 2.82)	-0.133 (-0.426 to 0.119)	0.95 (0.85 to 1.05)
Chile	2021	Diseases of the eye and adnexa	9,640	0.49	12,300 (8,160 to 18,600)	0.626 (0.415 to 0.945)	-2,670 (-8,760 to 1,490)	-0.136 (-0.445 to 0.076)	0.78 (0.52 to 1.18)	2.1	2.49 (2.19 to 2.83)	-0.390 (-0.728 to -0.085)	0.84 (0.74 to 0.96)
Chile	2020	Diseases of the ear and mastoid process	2,950	0.152	5,700 (5,410 to 6,010)	0.293 (0.278 to 0.309)	-2,750 (-3,070 to -2,470)	-0.141 (-0.158 to -0.127)	0.52 (0.49 to 0.54)	2.6	2.55 (2.27 to 2.85)	0.054 (-0.249 to 0.324)	1.02 (0.91 to 1.14)
Chile	2021	Diseases of the ear and mastoid process	3,260	0.165	5,770 (5,350 to 6,220)	0.293 (0.272 to 0.316)	-2,510 (-2,950 to -2,100)	-0.128 (-0.150 to -0.107)	0.56 (0.52 to 0.61)	2.7	2.55 (2.27 to 2.85)	0.154 (-0.156 to 0.425)	1.06 (0.95 to 1.19)
Chile	2020	Diseases of the circulatory system	113,000	5.79	134,000 (129,000 to 139,000)	6.89 (6.62 to 7.16)	-21,300 (-26,700 to -16,100)	-1.10 (-1.37 to -0.827)	0.84 (0.81 to 0.88)	8.8	9.00 (8.40 to 9.64)	-0.200 (-0.852 to 0.401)	0.98 (0.91 to 1.05)
Chile	2021	Diseases of the circulatory system	125,000	6.33	136,000 (128,000 to 143,000)	6.89 (6.51 to 7.28)	-11,000 (-18,700 to -3,720)	-0.560 (-0.951 to -0.189)	0.92 (0.87 to 0.97)	9	9.00 (8.17 to 9.92)	0.000 (-0.916 to 0.838)	1.00 (0.91 to 1.10)
Chile	2020	Diseases of the respiratory system	80,400	4.13	155,000 (149,000 to 162,000)	7.99 (7.64 to 8.35)	-74,900 (-82,000 to -68,200)	-3.85 (-4.21 to -3.50)	0.52 (0.50 to 0.54)	9.1	6.60 (5.86 to 7.44)	2.50 (1.66 to 3.26)	1.38 (1.22 to 1.56)
Chile	2021	Diseases of the respiratory system	87,400	4.44	153,000 (144,000 to 163,000)	7.80 (7.32 to 8.31)	-66,100 (-75,800 to -56,700)	-3.36 (-3.85 to -2.88)	0.57 (0.54 to 0.61)	8.7	6.60 (5.58 to 7.81)	2.10 (0.888 to 3.12)	1.32 (1.11 to 1.56)
Chile	2020	Diseases of the digestive system	182,000	9.36	233,000 (220,000 to 246,000)	12.0 (11.3 to 12.7)	-50,600 (-64,100 to -37,500)	-2.60 (-3.30 to -1.93)	0.78 (0.74 to 0.83)	4.6	4.44 (4.34 to 4.54)	0.160 (0.059 to 0.258)	1.04 (1.01 to 1.06)
Chile	2021	Diseases of the digestive system	213,000	10.8	235,000 (222,000 to 249,000)	12.0 (11.3 to 12.7)	-22,600 (-36,900 to -9,390)	-1.15 (-1.87 to -0.477)	0.90 (0.85 to 0.96)	4.3	4.44 (4.34 to 4.54)	-0.140 (-0.241 to -0.041)	0.97 (0.95 to 0.99)
Chile	2020	Diseases of the skin and subcutaneous tissue	18,200	0.933	25,300 (24,200 to 26,400)	1.30 (1.24 to 1.36)	-7,100 (-8,200 to -6,040)	-0.365 (-0.421 to -0.311)	0.72 (0.69 to 0.75)	8	7.40 (6.93 to 7.90)	0.600 (0.102 to 1.08)	1.08 (1.01 to 1.16)
Chile	2021	Diseases of the skin and subcutaneous tissue	17,100	0.87	25,600 (24,000 to 27,200)	1.30 (1.22 to 1.38)	-8,420 (-10,000 to -6,900)	-0.428 (-0.509 to -0.351)	0.67 (0.63 to 0.71)	8.1	7.40 (6.75 to 8.12)	0.700 (-0.017 to 1.36)	1.09 (1.00 to 1.20)
Chile	2020	Diseases of musculoskeletal system and connective tissue	58,000	2.98	94,500 (88,300 to 101,000)	4.86 (4.54 to 5.20)	-36,500 (-43,100 to -30,300)	-1.88 (-2.22 to -1.56)	0.61 (0.57 to 0.66)	3.7	3.55 (3.42 to 3.68)	0.152 (0.020 to 0.276)	1.04 (1.01 to 1.08)
Chile	2021	Diseases of musculoskeletal system and connective tissue	72,800	3.7	99,000 (89,900 to 109,000)	5.03 (4.57 to 5.54)	-26,300 (-36,500 to -17,500)	-1.34 (-1.85 to -0.889)	0.73 (0.67 to 0.81)	3.3	3.50 (3.32 to 3.68)	-0.197 (-0.385 to -0.023)	0.94 (0.90 to 0.99)
Chile	2020	Diseases of the genitourinary system	106,000	5.46	147,000 (142,000 to 152,000)	7.54 (7.28 to 7.80)	-40,500 (-45,700 to -35,400)	-2.08 (-2.35 to -1.82)	0.72 (0.70 to 0.75)	4.7	4.74 (4.41 to 5.09)	-0.037 (-0.394 to 0.291)	0.99 (0.92 to 1.07)

Location	Year	Condition	Observed hospitalizations	Observed hospitalizations per 1,000 population	Expected hospitalizations	Expected hospitalizations per 1,000 population	Difference in hospitalizations	Difference in rate	Hospitalizations observed-to-expected ratio	Observed length of stay	Expected length of stay	Difference in length of stay	Length of stay observed-to-expected ratio
Chile	2021	Diseases of the genitourinary system	122,000	6.2	148,000 (143,000 to 154,000)	7.54 (7.28 to 7.80)	-26,300 (-31,700 to -21,400)	-1.34 (-1.61 to -1.09)	0.82 (0.79 to 0.85)	4.5	4.74 (4.41 to 5.09)	-0.237 (-0.588 to 0.101)	0.95 (0.88 to 1.02)
Chile	2020	Pregnancy, childbirth and the puerperium	244,000	12.5	264,000 (247,000 to 283,000)	13.6 (12.7 to 14.5)	-20,800 (-39,000 to -3,940)	-1.07 (-2.00 to -0.202)	0.92 (0.86 to 0.98)	2.9	3.01 (2.95 to 3.07)	-0.110 (-0.169 to -0.050)	0.96 (0.94 to 0.98)
Chile	2021	Pregnancy, childbirth and the puerperium	222,000	11.3	258,000 (235,000 to 284,000)	13.1 (11.9 to 14.4)	-35,800 (-61,800 to -12,200)	-1.82 (-3.14 to -0.621)	0.86 (0.78 to 0.95)	2.9	3.01 (2.95 to 3.07)	-0.110 (-0.171 to -0.049)	0.96 (0.94 to 0.98)
Chile	2020	Certain conditions originating in the perinatal period	32,300	1.66	36,700 (32,600 to 41,200)	1.88 (1.68 to 2.12)	-4,390 (-9,040 to -374)	-0.226 (-0.465 to -0.019)	0.88 (0.78 to 0.99)	10.6	10.31 (9.90 to 10.74)	0.291 (-0.135 to 0.704)	1.03 (0.99 to 1.07)
Chile	2021	Certain conditions originating in the perinatal period	29,300	1.49	37,100 (31,400 to 43,800)	1.88 (1.60 to 2.22)	-7,770 (-14,600 to -2,140)	-0.395 (-0.741 to -0.109)	0.79 (0.67 to 0.93)	11.3	10.52 (9.93 to 11.14)	0.778 (0.154 to 1.38)	1.07 (1.01 to 1.14)
Chile	2020	Congenital malformations, deformations and chromosomal abnormalities	13,300	0.682	21,900 (20,300 to 23,700)	1.13 (1.04 to 1.22)	-8,680 (-10,400 to -7,060)	-0.446 (-0.533 to -0.363)	0.60 (0.56 to 0.65)	9.1	7.01 (6.17 to 7.95)	2.09 (1.15 to 2.92)	1.30 (1.15 to 1.47)
Chile	2021	Congenital malformations, deformations and chromosomal abnormalities	16,500	0.837	22,200 (20,600 to 24,000)	1.13 (1.04 to 1.22)	-5,720 (-7,460 to -4,080)	-0.290 (-0.379 to -0.207)	0.74 (0.69 to 0.80)	7.7	7.01 (6.17 to 7.95)	0.694 (-0.259 to 1.52)	1.10 (0.97 to 1.25)
Chile	2020	Symptoms, signs and abnormal clinical and laboratory findings, n.e.c.	21,000	1.08	26,900 (24,400 to 29,500)	1.38 (1.25 to 1.52)	-5,820 (-8,470 to -3,380)	-0.299 (-0.435 to -0.174)	0.78 (0.71 to 0.86)	4	4.17 (3.86 to 4.50)	-0.167 (-0.504 to 0.142)	0.96 (0.89 to 1.04)
Chile	2021	Symptoms, signs and abnormal clinical and laboratory findings, n.e.c.	19,400	0.986	24,800 (20,100 to 30,700)	1.26 (1.02 to 1.56)	-5,440 (-11,400 to -650)	-0.276 (-0.578 to -0.033)	0.78 (0.63 to 0.97)	4.9	4.17 (3.86 to 4.50)	0.733 (0.398 to 1.04)	1.18 (1.09 to 1.27)
Chile	2020	Injury, poisoning and other consequences of external causes	132,000	6.78	157,000 (153,000 to 161,000)	8.07 (7.86 to 8.29)	-25,100 (-29,300 to -21,000)	-1.29 (-1.51 to -1.08)	0.84 (0.82 to 0.86)	6.9	6.70 (6.36 to 7.06)	0.200 (-0.146 to 0.542)	1.03 (0.98 to 1.09)
Chile	2021	Injury, poisoning and other consequences of external causes	138,000	7.03	157,000 (151,000 to 163,000)	7.98 (7.69 to 8.29)	-18,700 (-24,700 to -12,800)	-0.950 (-1.26 to -0.650)	0.88 (0.85 to 0.92)	6.7	6.70 (6.23 to 7.21)	0.000 (-0.509 to 0.483)	1.00 (0.93 to 1.08)
Chile	2020	Factors influencing health status and contact with health services	39,800	2.05	55,800 (51,400 to 60,600)	2.87 (2.64 to 3.12)	-16,000 (-20,800 to -11,500)	-0.822 (-1.07 to -0.591)	0.71 (0.66 to 0.78)	3.2	3.00 (2.56 to 3.51)	0.200 (-0.307 to 0.638)	1.07 (0.91 to 1.25)
Chile	2021	Factors influencing health status and contact with health services	49,100	2.49	56,500 (50,200 to 63,500)	2.87 (2.55 to 3.23)	-7,420 (-14,500 to -1,260)	-0.377 (-0.736 to -0.064)	0.87 (0.77 to 0.97)	2.8	3.00 (2.40 to 3.75)	-0.200 (-0.940 to 0.398)	0.93 (0.75 to 1.17)
Costa Rica	2020	All causes	197,000	38.5	251,000 (245,000 to 257,000)	49.1 (47.9 to 50.3)	-54,100 (-60,300 to -48,100)	-10.6 (-11.8 to -9.42)	0.78 (0.77 to 0.80)				
Costa Rica	2021	All causes	193,000	37.3	248,000 (240,000 to 257,000)	48.1 (46.4 to 49.7)	-55,500 (-64,100 to -47,100)	-10.7 (-12.4 to -9.12)	0.78 (0.75 to 0.80)				

Location	Year	Condition	Observed hospitalizations	Observed hospitalizations per 1,000 population	Expected hospitalizations	Expected hospitalizations per 1,000 population	Difference in hospitalizations	Difference in rate	Hospitalizations observed-to-expected ratio	Observed length of stay	Expected length of stay	Difference in length of stay	Length of stay observed-to-expected ratio
Costa Rica	2020	Infectious and parasitic diseases	2,960	0.579	3,660 (2,010 to 6,670)	0.717 (0.394 to 1.30)	-708 (-3,660 to 914)	-0.139 (-0.716 to 0.179)	0.81 (0.45 to 1.45)	7.4	7.90 (5.86 to 10.64)	-0.500 (-3.18 to 1.55)	0.94 (0.70 to 1.26)
Costa Rica	2021	Infectious and parasitic diseases	2,370	0.46	3,700 (1,590 to 8,630)	0.717 (0.308 to 1.67)	-1,330 (-6,430 to 785)	-0.257 (-1.25 to 0.152)	0.64 (0.27 to 1.49)	7.6	7.90 (5.18 to 12.04)	-0.300 (-4.36 to 2.43)	0.96 (0.64 to 1.47)
Costa Rica	2020	Neoplasms	13,600	2.66	16,600 (15,900 to 17,300)	3.24 (3.11 to 3.38)	-2,990 (-3,710 to -2,310)	-0.586 (-0.726 to -0.452)	0.82 (0.79 to 0.85)	7.7	7.50 (6.95 to 8.10)	0.200 (-0.387 to 0.760)	1.03 (0.95 to 1.11)
Costa Rica	2021	Neoplasms	13,700	2.65	16,700 (16,000 to 17,500)	3.24 (3.11 to 3.38)	-3,080 (-3,800 to -2,380)	-0.596 (-0.737 to -0.461)	0.82 (0.78 to 0.85)	6.3	7.50 (6.73 to 8.36)	-1.20 (-2.04 to -0.437)	0.84 (0.76 to 0.94)
Costa Rica	2020	Diseases of the blood and bloodforming organs	1,250	0.244	1,470 (1,290 to 1,670)	0.287 (0.253 to 0.326)	-220 (-411 to -51)	-0.043 (-0.080 to -0.010)	0.85 (0.75 to 0.96)	7.5	7.92 (7.11 to 8.82)	-0.419 (-1.33 to 0.373)	0.95 (0.85 to 1.05)
Costa Rica	2021	Diseases of the blood and bloodforming organs	1,180	0.23	1,480 (1,240 to 1,770)	0.287 (0.240 to 0.343)	-298 (-587 to -56)	-0.058 (-0.114 to -0.011)	0.80 (0.67 to 0.95)	7.2	7.92 (7.11 to 8.82)	-0.719 (-1.62 to 0.074)	0.91 (0.82 to 1.01)
Costa Rica	2020	Endocrine, nutritional and metabolic diseases	3,810	0.745	4,610 (4,150 to 5,130)	0.902 (0.812 to 1.00)	-805 (-1,320 to -329)	-0.157 (-0.258 to -0.064)	0.83 (0.74 to 0.92)	10.1	10.40 (9.79 to 11.03)	-0.296 (-0.936 to 0.305)	0.97 (0.92 to 1.03)
Costa Rica	2021	Endocrine, nutritional and metabolic diseases	3,880	0.751	4,660 (4,010 to 5,410)	0.902 (0.777 to 1.05)	-783 (-1,530 to -127)	-0.152 (-0.296 to -0.025)	0.83 (0.72 to 0.97)	9.2	10.40 (9.79 to 11.03)	-1.20 (-1.84 to -0.597)	0.88 (0.83 to 0.94)
Costa Rica	2020	Mental and behavioural disorders	3,760	0.735	4,590 (3,960 to 5,310)	0.898 (0.776 to 1.04)	-831 (-1,550 to -200)	-0.163 (-0.303 to -0.039)	0.82 (0.71 to 0.95)	19	37.13 (17.46 to 78.96)	-18.13 (-59.75 to 1.25)	0.51 (0.24 to 1.07)
Costa Rica	2021	Mental and behavioural disorders	4,420	0.855	4,640 (4,000 to 5,370)	0.898 (0.776 to 1.04)	-221 (-953 to 407)	-0.043 (-0.185 to 0.079)	0.95 (0.82 to 1.10)	15.3	37.13 (17.46 to 78.96)	-21.83 (-63.36 to -1.78)	0.41 (0.19 to 0.90)
Costa Rica	2020	Diseases of the nervous system	2,070	0.406	3,420 (3,160 to 3,700)	0.669 (0.619 to 0.724)	-1,350 (-1,640 to -1,090)	-0.264 (-0.320 to -0.212)	0.61 (0.56 to 0.66)	9.7	11.15 (10.10 to 12.30)	-1.45 (-2.59 to -0.405)	0.87 (0.79 to 0.96)
Costa Rica	2021	Diseases of the nervous system	1,850	0.359	3,460 (3,090 to 3,860)	0.669 (0.599 to 0.749)	-1,600 (-2,020 to -1,240)	-0.310 (-0.391 to -0.240)	0.54 (0.48 to 0.60)	7.9	11.15 (10.10 to 12.30)	-3.25 (-4.38 to -2.22)	0.71 (0.64 to 0.78)
Costa Rica	2020	Diseases of the eye and adnexa	573	0.112	737 (552 to 983)	0.144 (0.108 to 0.192)	-164 (-411 to 20)	-0.032 (-0.080 to 0.004)	0.78 (0.58 to 1.04)	4.4	4.70 (4.27 to 5.17)	-0.300 (-0.783 to 0.129)	0.94 (0.85 to 1.03)
Costa Rica	2021	Diseases of the eye and adnexa	419	0.081	744 (495 to 1,120)	0.144 (0.096 to 0.217)	-325 (-700 to -75)	-0.063 (-0.135 to -0.015)	0.56 (0.37 to 0.85)	4.4	4.70 (4.11 to 5.38)	-0.300 (-0.981 to 0.282)	0.94 (0.82 to 1.07)
Costa Rica	2020	Diseases of the ear and mastoid process	173	0.034	297 (230 to 384)	0.058 (0.045 to 0.075)	-124 (-208 to -57)	-0.024 (-0.041 to -0.011)	0.58 (0.45 to 0.75)	4.8	5.10 (4.64 to 5.60)	-0.300 (-0.801 to 0.153)	0.94 (0.86 to 1.03)
Costa Rica	2021	Diseases of the ear and mastoid process	152	0.029	268 (186 to 385)	0.052 (0.036 to 0.075)	-116 (-234 to -35)	-0.022 (-0.045 to -0.007)	0.57 (0.39 to 0.81)	5	5.10 (4.47 to 5.82)	-0.100 (-0.829 to 0.541)	0.98 (0.86 to 1.12)
Costa Rica	2020	Diseases of the circulatory system	15,500	3.03	18,500 (17,300 to 19,800)	3.62 (3.38 to 3.86)	-2,990 (-4,240 to -1,810)	-0.586 (-0.829 to -0.353)	0.84 (0.79 to 0.90)	7.1	8.50 (7.84 to 9.22)	-1.40 (-2.11 to -0.737)	0.84 (0.77 to 0.91)
Costa Rica	2021	Diseases of the circulatory system	16,700	3.23	18,700 (17,000 to 20,500)	3.62 (3.29 to 3.97)	-2,000 (-3,840 to -332)	-0.388 (-0.744 to -0.064)	0.89 (0.81 to 0.98)	6.8	8.50 (7.58 to 9.53)	-1.70 (-2.72 to -0.782)	0.80 (0.71 to 0.90)

Location	Year	Condition	Observed hospitalizations	Observed hospitalizations per 1,000 population	Expected hospitalizations	Expected hospitalizations per 1,000 population	Difference in hospitalizations	Difference in rate	Hospitalizations observed-to-expected ratio	Observed length of stay	Expected length of stay	Difference in length of stay	Length of stay observed-to-expected ratio
Costa Rica	2020	Diseases of the respiratory system	6,430	1.26	18,300 (15,600 to 21,400)	3.57 (3.05 to 4.19)	-11,800 (-15,100 to -9,170)	-2.31 (-2.95 to -1.79)	0.35 (0.30 to 0.41)	7.3	7.23 (6.87 to 7.61)	0.072 (-0.307 to 0.440)	1.01 (0.96 to 1.06)
Costa Rica	2021	Diseases of the respiratory system	7,790	1.51	18,500 (15,700 to 21,600)	3.57 (3.05 to 4.19)	-10,700 (-13,900 to -7,910)	-2.07 (-2.68 to -1.53)	0.42 (0.36 to 0.50)	7	7.23 (6.87 to 7.61)	-0.228 (-0.612 to 0.131)	0.97 (0.92 to 1.02)
Costa Rica	2020	Diseases of the digestive system	20,000	3.91	24,400 (23,600 to 25,400)	4.78 (4.61 to 4.96)	-4,470 (-5,360 to -3,590)	-0.874 (-1.05 to -0.703)	0.82 (0.79 to 0.85)	4.7	5.00 (4.81 to 5.19)	-0.300 (-0.494 to -0.117)	0.94 (0.90 to 0.98)
Costa Rica	2021	Diseases of the digestive system	20,100	3.88	24,100 (22,900 to 25,400)	4.68 (4.44 to 4.93)	-4,090 (-5,370 to -2,880)	-0.792 (-1.04 to -0.557)	0.83 (0.79 to 0.87)	4.5	5.00 (4.74 to 5.28)	-0.500 (-0.783 to -0.232)	0.90 (0.85 to 0.95)
Costa Rica	2020	Diseases of the skin and subcutaneous tissue	2,840	0.556	3,600 (3,420 to 3,800)	0.705 (0.669 to 0.744)	-762 (-957 to -579)	-0.149 (-0.187 to -0.113)	0.79 (0.75 to 0.83)	9	9.40 (8.83 to 10.00)	-0.400 (-1.01 to 0.158)	0.96 (0.90 to 1.02)
Costa Rica	2021	Diseases of the skin and subcutaneous tissue	2,820	0.547	3,530 (3,280 to 3,800)	0.684 (0.634 to 0.737)	-706 (-975 to -454)	-0.137 (-0.189 to -0.088)	0.80 (0.74 to 0.86)	8.9	9.40 (8.61 to 10.26)	-0.500 (-1.35 to 0.297)	0.95 (0.87 to 1.03)
Costa Rica	2020	Diseases of musculoskeletal system and connective tissue	3,930	0.769	6,380 (5,780 to 7,030)	1.25 (1.13 to 1.38)	-2,450 (-3,100 to -1,860)	-0.479 (-0.606 to -0.364)	0.62 (0.56 to 0.68)	7.6	8.63 (7.44 to 10.01)	-1.03 (-2.39 to 0.139)	0.88 (0.76 to 1.02)
Costa Rica	2021	Diseases of musculoskeletal system and connective tissue	3,910	0.758	6,440 (5,840 to 7,100)	1.25 (1.13 to 1.38)	-2,530 (-3,190 to -1,940)	-0.490 (-0.618 to -0.375)	0.61 (0.55 to 0.67)	7.3	8.63 (7.44 to 10.01)	-1.33 (-2.73 to -0.134)	0.85 (0.73 to 0.98)
Costa Rica	2020	Diseases of the genitourinary system	13,000	2.55	19,200 (18,000 to 20,500)	3.76 (3.52 to 4.01)	-6,170 (-7,470 to -4,960)	-1.21 (-1.46 to -0.971)	0.68 (0.64 to 0.72)	5.3	5.01 (4.87 to 5.16)	0.290 (0.142 to 0.432)	1.06 (1.03 to 1.09)
Costa Rica	2021	Diseases of the genitourinary system	13,500	2.62	19,400 (17,700 to 21,300)	3.76 (3.43 to 4.12)	-5,880 (-7,710 to -4,200)	-1.14 (-1.49 to -0.814)	0.70 (0.64 to 0.76)	4.8	5.01 (4.87 to 5.16)	-0.210 (-0.357 to -0.068)	0.96 (0.93 to 0.99)
Costa Rica	2020	Pregnancy, childbirth and the puerperium	66,000	12.9	75,800 (71,800 to 80,000)	14.8 (14.0 to 15.7)	-9,770 (-13,900 to -5,850)	-1.91 (-2.72 to -1.14)	0.87 (0.83 to 0.92)	2.2	2.31 (2.25 to 2.37)	-0.110 (-0.172 to -0.049)	0.95 (0.93 to 0.98)
Costa Rica	2021	Pregnancy, childbirth and the puerperium	61,300	11.9	74,200 (68,800 to 80,100)	14.4 (13.3 to 15.5)	-13,000 (-18,900 to -7,460)	-2.51 (-3.66 to -1.45)	0.83 (0.76 to 0.89)	2.2	2.31 (2.25 to 2.37)	-0.110 (-0.171 to -0.050)	0.95 (0.93 to 0.98)
Costa Rica	2020	Certain conditions originating in the perinatal period	9,600	1.88	11,900 (11,100 to 12,800)	2.34 (2.17 to 2.51)	-2,350 (-3,260 to -1,510)	-0.459 (-0.638 to -0.296)	0.80 (0.75 to 0.86)	5.2	6.22 (6.00 to 6.45)	-1.02 (-1.25 to -0.797)	0.84 (0.81 to 0.87)
Costa Rica	2021	Certain conditions originating in the perinatal period	8,330	1.61	12,100 (10,900 to 13,400)	2.34 (2.11 to 2.59)	-3,730 (-5,070 to -2,520)	-0.723 (-0.982 to -0.488)	0.69 (0.62 to 0.77)	4.7	6.22 (6.00 to 6.45)	-1.52 (-1.74 to -1.30)	0.76 (0.73 to 0.78)
Costa Rica	2020	Congenital malformations, deformations and chromosomal abnormalities	1,930	0.378	3,550 (3,210 to 3,920)	0.694 (0.628 to 0.768)	-1,620 (-1,990 to -1,280)	-0.317 (-0.389 to -0.249)	0.54 (0.49 to 0.60)	4.8	5.30 (4.21 to 6.67)	-0.500 (-1.85 to 0.603)	0.91 (0.72 to 1.14)
Costa Rica	2021	Congenital malformations, deformations and chromosomal abnormalities	1,740	0.336	3,580 (3,240 to 3,960)	0.694 (0.628 to 0.768)	-1,850 (-2,220 to -1,510)	-0.358 (-0.430 to -0.292)	0.48 (0.44 to 0.54)	3.5	5.30 (3.83 to 7.33)	-1.80 (-3.83 to -0.346)	0.66 (0.48 to 0.91)

Location	Year	Condition	Observed hospitalizations	Observed hospitalizations per 1,000 population	Expected hospitalizations	Expected hospitalizations per 1,000 population	Difference in hospitalizations	Difference in rate	Hospitalizations observed-to-expected ratio	Observed length of stay	Expected length of stay	Difference in length of stay	Length of stay observed-to-expected ratio
Costa Rica	2020	Symptoms, signs and abnormal clinical and laboratory findings, n.e.c.	2,750	0.538	3,600 (3,340 to 3,890)	0.705 (0.653 to 0.762)	-856 (-1,150 to -588)	-0.168 (-0.225 to -0.115)	0.76 (0.71 to 0.82)	4.6	5.30 (3.74 to 7.50)	-0.700 (-2.93 to 0.868)	0.87 (0.61 to 1.23)
Costa Rica	2021	Symptoms, signs and abnormal clinical and laboratory findings, n.e.c.	2,700	0.522	3,680 (3,380 to 4,000)	0.712 (0.655 to 0.774)	-982 (-1,300 to -691)	-0.190 (-0.252 to -0.134)	0.73 (0.67 to 0.80)	4.5	5.30 (3.24 to 8.67)	-0.800 (-4.11 to 1.23)	0.85 (0.52 to 1.38)
Costa Rica	2020	Injury, poisoning and other consequences of external causes	18,900	3.7	22,700 (21,600 to 23,900)	4.44 (4.22 to 4.67)	-3,750 (-4,930 to -2,660)	-0.733 (-0.965 to -0.520)	0.83 (0.79 to 0.88)	6.7	7.51 (6.79 to 8.31)	-0.811 (-1.60 to -0.088)	0.89 (0.81 to 0.99)
Costa Rica	2021	Injury, poisoning and other consequences of external causes	19,100	3.7	22,900 (21,800 to 24,100)	4.44 (4.22 to 4.67)	-3,840 (-5,000 to -2,720)	-0.743 (-0.968 to -0.527)	0.83 (0.79 to 0.88)	6.4	7.51 (6.79 to 8.31)	-1.11 (-1.88 to -0.386)	0.85 (0.77 to 0.94)
Costa Rica	2020	Factors influencing health status and contact with health services	7,570	1.48	10,300 (9,060 to 11,700)	2.01 (1.77 to 2.28)	-2,720 (-4,100 to -1,510)	-0.532 (-0.802 to -0.295)	0.74 (0.65 to 0.83)	4.1	4.23 (3.68 to 4.86)	-0.130 (-0.750 to 0.434)	0.97 (0.85 to 1.12)
Costa Rica	2021	Factors influencing health status and contact with health services	6,800	1.32	10,400 (8,690 to 12,400)	2.01 (1.68 to 2.41)	-3,590 (-5,690 to -1,940)	-0.695 (-1.10 to -0.375)	0.65 (0.54 to 0.78)	3.9	4.23 (3.68 to 4.86)	-0.330 (-0.953 to 0.211)	0.92 (0.80 to 1.06)
Czechia	2020	All causes	1,700,000	159	2,020,000 (1,940,000 to 2,110,000)	189 (181 to 198)	-323,000 (-412,000 to -239,000)	-30.2 (-38.5 to -22.3)	0.84 (0.80 to 0.88)				
Czechia	2021	All causes	1,680,000	160	1,990,000 (1,870,000 to 2,110,000)	189 (178 to 201)	-311,000 (-437,000 to -193,000)	-29.6 (-41.6 to -18.4)	0.84 (0.79 to 0.90)				
Czechia	2020	Infectious and parasitic diseases	40,900	3.83	51,100 (48,100 to 54,400)	4.78 (4.49 to 5.09)	-10,200 (-13,400 to -7,150)	-0.953 (-1.25 to -0.668)	0.80 (0.75 to 0.85)	10.4	9.66 (9.34 to 9.99)	0.741 (0.410 to 1.06)	1.08 (1.04 to 1.11)
Czechia	2021	Infectious and parasitic diseases	40,400	3.84	50,200 (47,200 to 53,400)	4.78 (4.49 to 5.09)	-9,860 (-13,100 to -6,800)	-0.938 (-1.25 to -0.647)	0.80 (0.76 to 0.86)	9.6	9.66 (9.34 to 9.99)	-0.059 (-0.386 to 0.254)	0.99 (0.96 to 1.03)
Czechia	2020	Neoplasms	125,000	11.7	137,000 (130,000 to 143,000)	12.8 (12.2 to 13.4)	-12,000 (-18,600 to -5,550)	-1.12 (-1.74 to -0.519)	0.91 (0.87 to 0.96)	7.7	7.59 (7.34 to 7.84)	0.113 (-0.136 to 0.355)	1.01 (0.98 to 1.05)
Czechia	2021	Neoplasms	143,000	13.6	131,000 (123,000 to 141,000)	12.5 (11.7 to 13.4)	11,800 (2,770 to 20,400)	1.13 (0.263 to 1.94)	1.09 (1.02 to 1.17)	7.1	7.47 (7.14 to 7.83)	-0.375 (-0.738 to -0.039)	0.95 (0.91 to 0.99)
Czechia	2020	Diseases of the blood and bloodforming organs	14,600	1.36	14,100 (12,400 to 16,000)	1.32 (1.16 to 1.49)	477 (-1,410 to 2,120)	0.045 (-0.132 to 0.199)	1.03 (0.91 to 1.17)	7.2	7.20 (6.61 to 7.85)	0.000 (-0.663 to 0.588)	1.00 (0.92 to 1.09)
Czechia	2021	Diseases of the blood and bloodforming organs	14,100	1.34	13,800 (12,200 to 15,700)	1.32 (1.16 to 1.49)	215 (-1,610 to 1,860)	0.020 (-0.154 to 0.177)	1.02 (0.90 to 1.15)	7.2	7.20 (6.37 to 8.13)	0.000 (-0.929 to 0.822)	1.00 (0.89 to 1.13)
Czechia	2020	Endocrine, nutritional and metabolic diseases	40,100	3.75	56,500 (54,700 to 58,400)	5.28 (5.12 to 5.46)	-16,400 (-18,300 to -14,600)	-1.54 (-1.71 to -1.37)	0.71 (0.69 to 0.73)	9.7	8.70 (8.03 to 9.42)	1.00 (0.286 to 1.65)	1.11 (1.03 to 1.21)
Czechia	2021	Endocrine, nutritional and metabolic diseases	37,600	3.58	57,300 (53,900 to 60,800)	5.45 (5.13 to 5.79)	-19,600 (-23,200 to -16,200)	-1.87 (-2.21 to -1.54)	0.66 (0.62 to 0.70)	9.8	8.70 (7.77 to 9.74)	1.10 (0.064 to 2.02)	1.13 (1.01 to 1.26)

Location	Year	Condition	Observed hospitalizations	Observed hospitalizations per 1,000 population	Expected hospitalizations	Expected hospitalizations per 1,000 population	Difference in hospitalizations	Difference in rate	Hospitalizations observed-to-expected ratio	Observed length of stay	Expected length of stay	Difference in length of stay	Length of stay observed-to-expected ratio
Czechia	2020	Mental and behavioural disorders	60,800	5.68	69,700 (66,400 to 73,100)	6.51 (6.21 to 6.83)	-8,890 (-12,200 to -5,600)	-0.831 (-1.14 to -0.524)	0.87 (0.83 to 0.92)	44.1	40.84 (39.68 to 42.02)	3.26 (2.09 to 4.45)	1.08 (1.05 to 1.11)
Czechia	2021	Mental and behavioural disorders	60,200	5.73	68,400 (63,900 to 73,200)	6.51 (6.08 to 6.97)	-8,180 (-13,100 to -3,690)	-0.779 (-1.24 to -0.351)	0.88 (0.82 to 0.94)	43.4	40.84 (39.68 to 42.02)	2.56 (1.37 to 3.69)	1.06 (1.03 to 1.09)
Czechia	2020	Diseases of the nervous system	53,200	4.98	63,200 (60,000 to 66,600)	5.91 (5.61 to 6.23)	-9,990 (-13,300 to -6,830)	-0.934 (-1.24 to -0.639)	0.84 (0.80 to 0.89)	13.7	12.10 (11.07 to 13.22)	1.60 (0.493 to 2.61)	1.13 (1.04 to 1.24)
Czechia	2021	Diseases of the nervous system	47,100	4.48	62,100 (58,900 to 65,400)	5.91 (5.61 to 6.23)	-15,000 (-18,400 to -11,800)	-1.43 (-1.75 to -1.12)	0.76 (0.72 to 0.80)	13.8	12.10 (10.67 to 13.72)	1.70 (0.122 to 3.15)	1.14 (1.01 to 1.30)
Czechia	2020	Diseases of the eye and adnexa	14,700	1.38	20,200 (17,900 to 22,900)	1.89 (1.67 to 2.14)	-5,490 (-8,180 to -3,160)	-0.513 (-0.765 to -0.296)	0.73 (0.64 to 0.82)	3.7	3.60 (3.43 to 3.77)	0.100 (-0.073 to 0.266)	1.03 (0.98 to 1.08)
Czechia	2021	Diseases of the eye and adnexa	13,200	1.26	20,300 (16,400 to 25,200)	1.93 (1.56 to 2.39)	-7,080 (-11,900 to -3,130)	-0.674 (-1.13 to -0.298)	0.65 (0.53 to 0.81)	3.6	3.60 (3.37 to 3.85)	0.000 (-0.245 to 0.237)	1.00 (0.94 to 1.07)
Czechia	2020	Diseases of the ear and mastoid process	10,500	0.979	15,200 (13,900 to 16,600)	1.42 (1.30 to 1.55)	-4,680 (-6,100 to -3,380)	-0.438 (-0.570 to -0.316)	0.69 (0.63 to 0.76)	4.5	4.40 (4.27 to 4.54)	0.099 (-0.038 to 0.235)	1.02 (0.99 to 1.06)
Czechia	2021	Diseases of the ear and mastoid process	9,140	0.87	14,900 (13,400 to 16,600)	1.42 (1.28 to 1.58)	-5,790 (-7,520 to -4,280)	-0.551 (-0.716 to -0.407)	0.61 (0.55 to 0.68)	4.4	4.30 (4.12 to 4.49)	0.096 (-0.097 to 0.278)	1.02 (0.98 to 1.07)
Czechia	2020	Diseases of the circulatory system	216,000	20.2	247,000 (232,000 to 262,000)	23.1 (21.7 to 24.5)	-30,900 (-46,400 to -16,100)	-2.89 (-4.34 to -1.50)	0.87 (0.82 to 0.93)	10.6	9.80 (9.37 to 10.24)	0.801 (0.359 to 1.22)	1.08 (1.04 to 1.13)
Czechia	2021	Diseases of the circulatory system	209,000	19.9	237,000 (218,000 to 259,000)	22.6 (20.7 to 24.6)	-28,700 (-50,300 to -9,070)	-2.73 (-4.78 to -0.863)	0.88 (0.81 to 0.96)	10	9.60 (9.02 to 10.23)	0.397 (-0.230 to 0.988)	1.04 (0.98 to 1.11)
Czechia	2020	Diseases of the respiratory system	118,000	11.1	127,000 (117,000 to 138,000)	11.9 (10.9 to 12.9)	-9,000 (-20,000 to 1,110)	-0.842 (-1.87 to 0.104)	0.93 (0.86 to 1.01)	11.4	10.20 (9.81 to 10.61)	1.20 (0.801 to 1.59)	1.12 (1.08 to 1.16)
Czechia	2021	Diseases of the respiratory system	160,000	15.2	125,000 (111,000 to 141,000)	11.9 (10.6 to 13.4)	34,800 (19,400 to 48,600)	3.31 (1.85 to 4.63)	1.28 (1.14 to 1.44)	12	10.41 (9.85 to 11.00)	1.59 (0.996 to 2.16)	1.15 (1.09 to 1.22)
Czechia	2020	Diseases of the digestive system	151,000	14.1	183,000 (173,000 to 193,000)	17.1 (16.2 to 18.1)	-31,600 (-42,300 to -21,500)	-2.95 (-3.95 to -2.01)	0.83 (0.78 to 0.88)	6.3	5.90 (5.79 to 6.02)	0.398 (0.284 to 0.511)	1.07 (1.05 to 1.09)
Czechia	2021	Diseases of the digestive system	146,000	13.9	180,000 (170,000 to 190,000)	17.1 (16.2 to 18.1)	-33,100 (-43,400 to -23,500)	-3.15 (-4.13 to -2.23)	0.82 (0.77 to 0.86)	6.1	5.80 (5.56 to 6.06)	0.295 (0.038 to 0.540)	1.05 (1.01 to 1.10)
Czechia	2020	Diseases of the skin and subcutaneous tissue	18,300	1.71	24,400 (22,600 to 26,400)	2.28 (2.11 to 2.47)	-6,080 (-8,070 to -4,180)	-0.569 (-0.754 to -0.391)	0.75 (0.69 to 0.81)	11	9.70 (9.30 to 10.12)	1.30 (0.874 to 1.70)	1.13 (1.09 to 1.18)
Czechia	2021	Diseases of the skin and subcutaneous tissue	16,000	1.52	24,000 (21,400 to 26,800)	2.28 (2.04 to 2.55)	-7,970 (-10,800 to -5,500)	-0.758 (-1.02 to -0.524)	0.67 (0.60 to 0.74)	10.2	9.70 (9.13 to 10.30)	0.500 (-0.099 to 1.06)	1.05 (0.99 to 1.12)
Czechia	2020	Diseases of musculoskeletal system and connective tissue	115,000	10.8	157,000 (146,000 to 169,000)	14.7 (13.7 to 15.8)	-42,300 (-54,100 to -31,200)	-3.96 (-5.06 to -2.91)	0.73 (0.68 to 0.79)	9.4	8.80 (7.97 to 9.72)	0.600 (-0.312 to 1.42)	1.07 (0.97 to 1.18)
Czechia	2021	Diseases of musculoskeletal system and connective tissue	104,000	9.88	155,000 (139,000 to 172,000)	14.7 (13.3 to 16.3)	-50,700 (-68,000 to -35,600)	-4.82 (-6.47 to -3.39)	0.67 (0.60 to 0.74)	9.1	8.80 (7.64 to 10.13)	0.300 (-1.03 to 1.46)	1.03 (0.90 to 1.19)
Czechia	2020	Diseases of the genitourinary system	121,000	11.3	149,000 (140,000 to 158,000)	13.9 (13.1 to 14.8)	-28,100 (-37,400 to -19,600)	-2.63 (-3.50 to -1.83)	0.81 (0.76 to 0.86)	5.9	5.58 (5.43 to 5.73)	0.320 (0.168 to 0.469)	1.06 (1.03 to 1.09)

Location	Year	Condition	Observed hospitalizations	Observed hospitalizations per 1,000 population	Expected hospitalizations	Expected hospitalizations per 1,000 population	Difference in hospitalizations	Difference in rate	Hospitalizations observed-to-expected ratio	Observed length of stay	Expected length of stay	Difference in length of stay	Length of stay observed-to-expected ratio
Czechia	2021	Diseases of the genitourinary system	115,000	10.9	146,000 (134,000 to 159,000)	13.9 (12.8 to 15.2)	-31,200 (-43,900 to -19,400)	-2.97 (-4.17 to -1.84)	0.79 (0.72 to 0.86)	5.6	5.66 (5.45 to 5.88)	-0.061 (-0.283 to 0.152)	0.99 (0.95 to 1.03)
Czechia	2020	Pregnancy, childbirth and the puerperium	130,000	12.2	139,000 (130,000 to 149,000)	13.0 (12.2 to 13.9)	-8,680 (-18,100 to 340)	-0.811 (-1.69 to 0.032)	0.94 (0.88 to 1.00)	3.9	4.02 (3.92 to 4.12)	-0.120 (-0.225 to -0.018)	0.97 (0.95 to 1.00)
Czechia	2021	Pregnancy, childbirth and the puerperium	130,000	12.4	137,000 (124,000 to 150,000)	13.0 (11.8 to 14.3)	-6,520 (-20,100 to 5,510)	-0.621 (-1.91 to 0.525)	0.95 (0.87 to 1.04)	3.8	3.94 (3.80 to 4.09)	-0.141 (-0.278 to 0.000)	0.96 (0.93 to 1.00)
Czechia	2020	Certain conditions originating in the perinatal period	7,320	0.684	8,920 (8,340 to 9,540)	0.834 (0.779 to 0.892)	-1,600 (-2,220 to -998)	-0.149 (-0.208 to -0.093)	0.82 (0.77 to 0.88)	10.8	11.00 (9.91 to 12.21)	-0.200 (-1.40 to 0.897)	0.98 (0.89 to 1.09)
Czechia	2021	Certain conditions originating in the perinatal period	5,620	0.535	7,910 (7,190 to 8,700)	0.753 (0.684 to 0.828)	-2,280 (-3,080 to -1,560)	-0.217 (-0.293 to -0.148)	0.71 (0.65 to 0.78)	10.6	11.00 (9.49 to 12.75)	-0.400 (-2.15 to 1.11)	0.96 (0.83 to 1.12)
Czechia	2020	Congenital malformations, deformations and chromosomal abnormalities	8,690	0.812	10,800 (10,300 to 11,200)	1.01 (0.963 to 1.05)	-2,070 (-2,550 to -1,620)	-0.194 (-0.238 to -0.152)	0.81 (0.77 to 0.84)	5.3	5.10 (4.73 to 5.50)	0.200 (-0.195 to 0.569)	1.04 (0.96 to 1.12)
Czechia	2021	Congenital malformations, deformations and chromosomal abnormalities	8,840	0.841	10,400 (9,760 to 11,000)	0.988 (0.929 to 1.05)	-1,540 (-2,200 to -922)	-0.146 (-0.209 to -0.088)	0.85 (0.80 to 0.91)	5.2	5.10 (4.58 to 5.68)	0.100 (-0.488 to 0.616)	1.02 (0.91 to 1.13)
Czechia	2020	Symptoms, signs and abnormal clinical and laboratory findings, n.e.c.	53,500	5	66,900 (60,900 to 73,400)	6.25 (5.69 to 6.86)	-13,400 (-20,000 to -7,340)	-1.25 (-1.87 to -0.687)	0.80 (0.73 to 0.88)	7.2	5.93 (5.34 to 6.59)	1.27 (0.622 to 1.84)	1.21 (1.09 to 1.34)
Czechia	2021	Symptoms, signs and abnormal clinical and laboratory findings, n.e.c.	51,100	4.87	65,700 (57,600 to 75,000)	6.25 (5.48 to 7.14)	-14,600 (-23,800 to -6,400)	-1.39 (-2.26 to -0.609)	0.78 (0.68 to 0.89)	6.7	5.93 (5.34 to 6.59)	0.768 (0.111 to 1.36)	1.13 (1.02 to 1.26)
Czechia	2020	Injury, poisoning and other consequences of external causes	147,000	13.8	172,000 (164,000 to 180,000)	16.1 (15.3 to 16.8)	-24,500 (-32,300 to -16,700)	-2.29 (-3.02 to -1.56)	0.86 (0.82 to 0.90)	9.9	9.26 (8.79 to 9.75)	0.640 (0.151 to 1.10)	1.07 (1.02 to 1.13)
Czechia	2021	Injury, poisoning and other consequences of external causes	141,000	13.4	169,000 (158,000 to 180,000)	16.1 (15.1 to 17.1)	-27,800 (-38,800 to -17,300)	-2.64 (-3.70 to -1.65)	0.84 (0.78 to 0.89)	9.1	9.14 (8.58 to 9.73)	-0.038 (-0.640 to 0.529)	1.00 (0.93 to 1.06)
Czechia	2020	Factors influencing health status and contact with health services	255,000	23.8	310,000 (287,000 to 334,000)	29.0 (26.8 to 31.3)	-54,900 (-79,100 to -32,100)	-5.13 (-7.40 to -3.00)	0.82 (0.76 to 0.89)	7.7	7.50 (6.84 to 8.22)	0.200 (-0.507 to 0.869)	1.03 (0.94 to 1.13)
Czechia	2021	Factors influencing health status and contact with health services	226,000	21.5	312,000 (280,000 to 348,000)	29.7 (26.7 to 33.1)	-86,300 (-123,000 to -53,500)	-8.22 (-11.7 to -5.09)	0.72 (0.65 to 0.81)	7.4	7.50 (6.59 to 8.54)	-0.100 (-1.16 to 0.800)	0.99 (0.86 to 1.12)
Germany	2020	All causes	18,200,000	219	21,100,000 (20,600,000 to 21,600,000)	254 (248 to 260)	-2,910,000 (-3,450,000 to -2,410,000)	-35.0 (-41.4 to -28.9)	0.86 (0.84 to 0.88)				
Germany	2021	All causes	18,100,000	218	21,200,000 (20,000,000 to 22,300,000)	254 (241 to 268)	-3,030,000 (-4,230,000 to -1,890,000)	-36.4 (-50.9 to -22.7)	0.86 (0.81 to 0.91)				

Location	Year	Condition	Observed hospitalizations	Observed hospitalizations per 1,000 population	Expected hospitalizations	Expected hospitalizations per 1,000 population	Difference in hospitalizations	Difference in rate	Hospitalizations observed-to-expected ratio	Observed length of stay	Expected length of stay	Difference in length of stay	Length of stay observed-to-expected ratio
Germany	2020	Infectious and parasitic diseases	447,000	5.38	637,000 (611,000 to 664,000)	7.66 (7.35 to 7.99)	-190,000 (-217,000 to -164,000)	-2.28 (-2.61 to -1.97)	0.70 (0.67 to 0.73)	8	7.74 (7.60 to 7.88)	0.260 (0.123 to 0.396)	1.03 (1.02 to 1.05)
Germany	2021	Infectious and parasitic diseases	450,000	5.4	637,000 (597,000 to 680,000)	7.66 (7.18 to 8.18)	-188,000 (-231,000 to -148,000)	-2.26 (-2.77 to -1.78)	0.71 (0.66 to 0.75)	7.8	7.74 (7.60 to 7.88)	0.060 (-0.078 to 0.193)	1.01 (0.99 to 1.03)
Germany	2020	Neoplasms	1,900,000	22.9	2,050,000 (2,010,000 to 2,090,000)	24.7 (24.2 to 25.1)	-149,000 (-187,000 to -111,000)	-1.79 (-2.24 to -1.34)	0.93 (0.91 to 0.94)	8.4	8.58 (8.45 to 8.70)	-0.176 (-0.305 to -0.047)	0.98 (0.96 to 0.99)
Germany	2021	Neoplasms	1,890,000	22.7	2,050,000 (2,010,000 to 2,090,000)	24.7 (24.2 to 25.1)	-163,000 (-203,000 to -123,000)	-1.96 (-2.44 to -1.48)	0.92 (0.90 to 0.94)	8.2	8.45 (8.28 to 8.63)	-0.254 (-0.436 to -0.078)	0.97 (0.95 to 0.99)
Germany	2020	Diseases of the blood and bloodforming organs	114,000	1.37	133,000 (127,000 to 139,000)	1.60 (1.52 to 1.67)	-18,700 (-25,200 to -12,500)	-0.225 (-0.304 to -0.151)	0.86 (0.82 to 0.90)	6.6	6.55 (6.40 to 6.71)	0.049 (-0.104 to 0.204)	1.01 (0.98 to 1.03)
Germany	2021	Diseases of the blood and bloodforming organs	113,000	1.36	133,000 (126,000 to 141,000)	1.60 (1.51 to 1.70)	-20,500 (-28,600 to -13,000)	-0.247 (-0.344 to -0.156)	0.85 (0.80 to 0.90)	6.4	6.41 (6.20 to 6.62)	-0.006 (-0.220 to 0.203)	1.00 (0.97 to 1.03)
Germany	2020	Endocrine, nutritional and metabolic diseases	471,000	5.66	559,000 (547,000 to 571,000)	6.72 (6.58 to 6.87)	-88,100 (-100,000 to -76,000)	-1.06 (-1.21 to -0.914)	0.84 (0.82 to 0.86)	8.3	8.48 (8.26 to 8.69)	-0.176 (-0.393 to 0.034)	0.98 (0.95 to 1.00)
Germany	2021	Endocrine, nutritional and metabolic diseases	476,000	5.72	559,000 (542,000 to 577,000)	6.72 (6.52 to 6.93)	-83,000 (-101,000 to -65,800)	-0.998 (-1.21 to -0.791)	0.85 (0.83 to 0.88)	8.2	8.35 (8.06 to 8.66)	-0.154 (-0.462 to 0.136)	0.98 (0.95 to 1.02)
Germany	2020	Mental and behavioural disorders	1,180,000	14.2	1,350,000 (1,340,000 to 1,360,000)	16.3 (16.1 to 16.4)	-175,000 (-186,000 to -164,000)	-2.10 (-2.24 to -1.97)	0.87 (0.86 to 0.88)	26.5	27.00 (26.84 to 27.17)	-0.503 (-0.663 to -0.343)	0.98 (0.98 to 0.99)
Germany	2021	Mental and behavioural disorders	1,220,000	14.6	1,340,000 (1,320,000 to 1,350,000)	16.1 (15.9 to 16.3)	-121,000 (-137,000 to -106,000)	-1.46 (-1.65 to -1.27)	0.91 (0.90 to 0.92)	26.9	27.51 (27.04 to 27.99)	-0.610 (-1.09 to -0.137)	0.98 (0.96 to 0.99)
Germany	2020	Diseases of the nervous system	660,000	7.93	804,000 (760,000 to 851,000)	9.67 (9.14 to 10.2)	-145,000 (-191,000 to -100,000)	-1.74 (-2.30 to -1.21)	0.82 (0.78 to 0.87)	8.9	8.80 (8.58 to 9.03)	0.100 (-0.125 to 0.324)	1.01 (0.99 to 1.04)
Germany	2021	Diseases of the nervous system	664,000	7.98	805,000 (760,000 to 852,000)	9.67 (9.14 to 10.2)	-141,000 (-189,000 to -95,900)	-1.69 (-2.28 to -1.15)	0.82 (0.78 to 0.87)	9.5	8.80 (8.49 to 9.12)	0.700 (0.378 to 1.01)	1.08 (1.04 to 1.12)
Germany	2020	Diseases of the eye and adnexa	282,000	3.4	341,000 (331,000 to 350,000)	4.10 (3.99 to 4.21)	-58,300 (-67,900 to -49,100)	-0.701 (-0.817 to -0.590)	0.83 (0.81 to 0.85)	3.1	3.05 (2.92 to 3.19)	0.051 (-0.083 to 0.182)	1.02 (0.97 to 1.06)
Germany	2021	Diseases of the eye and adnexa	284,000	3.41	341,000 (332,000 to 350,000)	4.10 (3.99 to 4.21)	-56,900 (-66,300 to -47,600)	-0.684 (-0.797 to -0.572)	0.83 (0.81 to 0.86)	3	3.00 (2.82 to 3.19)	0.001 (-0.195 to 0.185)	1.00 (0.94 to 1.07)
Germany	2020	Diseases of the ear and mastoid process	127,000	1.52	161,000 (154,000 to 168,000)	1.93 (1.85 to 2.02)	-34,100 (-41,100 to -27,300)	-0.410 (-0.494 to -0.328)	0.79 (0.75 to 0.82)	4.5	4.60 (4.47 to 4.73)	-0.100 (-0.231 to 0.021)	0.98 (0.95 to 1.00)
Germany	2021	Diseases of the ear and mastoid process	122,000	1.46	161,000 (154,000 to 168,000)	1.93 (1.85 to 2.02)	-39,200 (-46,000 to -32,500)	-0.471 (-0.553 to -0.390)	0.76 (0.73 to 0.79)	4.4	4.60 (4.32 to 4.89)	-0.200 (-0.496 to 0.072)	0.96 (0.90 to 1.02)
Germany	2020	Diseases of the circulatory system	2,730,000	32.8	3,080,000 (2,980,000 to 3,190,000)	37.1 (35.8 to 38.4)	-353,000 (-462,000 to -250,000)	-4.25 (-5.56 to -3.00)	0.89 (0.86 to 0.92)	9	8.91 (8.68 to 9.13)	0.095 (-0.129 to 0.315)	1.01 (0.99 to 1.04)
Germany	2021	Diseases of the circulatory system	2,710,000	32.6	3,080,000 (2,980,000 to 3,190,000)	37.1 (35.8 to 38.4)	-373,000 (-482,000 to -271,000)	-4.48 (-5.79 to -3.26)	0.88 (0.85 to 0.91)	8.9	8.81 (8.50 to 9.13)	0.089 (-0.223 to 0.396)	1.01 (0.98 to 1.05)

Location	Year	Condition	Observed hospitalizations	Observed hospitalizations per 1,000 population	Expected hospitalizations	Expected hospitalizations per 1,000 population	Difference in hospitalizations	Difference in rate	Hospitalizations observed-to-expected ratio	Observed length of stay	Expected length of stay	Difference in length of stay	Length of stay observed-to-expected ratio
Germany	2020	Diseases of the respiratory system	1,070,000	12.9	1,350,000 (1,260,000 to 1,450,000)	16.3 (15.1 to 17.5)	-279,000 (-383,000 to -185,000)	-3.36 (-4.60 to -2.22)	0.79 (0.74 to 0.85)	8	7.52 (7.27 to 7.77)	0.484 (0.229 to 0.729)	1.06 (1.03 to 1.10)
Germany	2021	Diseases of the respiratory system	1,070,000	12.8	1,340,000 (1,240,000 to 1,450,000)	16.1 (14.9 to 17.4)	-272,000 (-381,000 to -169,000)	-3.26 (-4.58 to -2.03)	0.80 (0.74 to 0.86)	8.6	7.43 (7.09 to 7.79)	1.17 (0.803 to 1.51)	1.16 (1.10 to 1.21)
Germany	2020	Diseases of the digestive system	1,700,000	20.5	1,960,000 (1,890,000 to 2,040,000)	23.6 (22.7 to 24.5)	-259,000 (-337,000 to -186,000)	-3.12 (-4.05 to -2.23)	0.87 (0.83 to 0.90)	5.7	5.47 (5.35 to 5.60)	0.228 (0.105 to 0.347)	1.04 (1.02 to 1.06)
Germany	2021	Diseases of the digestive system	1,680,000	20.2	1,960,000 (1,860,000 to 2,070,000)	23.6 (22.3 to 24.9)	-280,000 (-390,000 to -175,000)	-3.37 (-4.69 to -2.10)	0.86 (0.81 to 0.91)	5.6	5.35 (5.18 to 5.52)	0.254 (0.085 to 0.423)	1.05 (1.02 to 1.08)
Germany	2020	Diseases of the skin and subcutaneous tissue	246,000	2.95	309,000 (297,000 to 322,000)	3.72 (3.57 to 3.87)	-63,500 (-75,700 to -51,500)	-0.763 (-0.910 to -0.619)	0.79 (0.76 to 0.83)	7.3	7.32 (7.11 to 7.54)	-0.023 (-0.246 to 0.194)	1.00 (0.97 to 1.03)
Germany	2021	Diseases of the skin and subcutaneous tissue	240,000	2.88	307,000 (281,000 to 336,000)	3.69 (3.38 to 4.03)	-67,500 (-95,700 to -42,100)	-0.812 (-1.15 to -0.507)	0.78 (0.71 to 0.85)	7.6	7.15 (6.86 to 7.46)	0.450 (0.146 to 0.746)	1.06 (1.02 to 1.11)
Germany	2020	Diseases of musculoskeletal system and connective tissue	1,880,000	22.6	2,310,000 (2,230,000 to 2,400,000)	27.8 (26.9 to 28.8)	-433,000 (-517,000 to -352,000)	-5.21 (-6.21 to -4.23)	0.81 (0.78 to 0.84)	10.5	11.30 (11.02 to 11.59)	-0.804 (-1.09 to -0.522)	0.93 (0.91 to 0.95)
Germany	2021	Diseases of musculoskeletal system and connective tissue	1,840,000	22.1	2,320,000 (2,240,000 to 2,400,000)	27.8 (26.9 to 28.8)	-478,000 (-561,000 to -397,000)	-5.74 (-6.74 to -4.77)	0.79 (0.77 to 0.82)	10.5	11.51 (10.88 to 12.17)	-1.01 (-1.67 to -0.389)	0.91 (0.86 to 0.96)
Germany	2020	Diseases of the genitourinary system	967,000	11.6	1,090,000 (1,060,000 to 1,120,000)	13.1 (12.8 to 13.5)	-126,000 (-156,000 to -95,800)	-1.51 (-1.88 to -1.15)	0.88 (0.86 to 0.91)	5.3	5.24 (5.15 to 5.33)	0.063 (-0.031 to 0.156)	1.01 (0.99 to 1.03)
Germany	2021	Diseases of the genitourinary system	954,000	11.5	1,100,000 (1,060,000 to 1,150,000)	13.3 (12.8 to 13.8)	-150,000 (-196,000 to -108,000)	-1.81 (-2.35 to -1.29)	0.86 (0.83 to 0.90)	5.2	5.18 (5.05 to 5.31)	0.025 (-0.105 to 0.155)	1.00 (0.98 to 1.03)
Germany	2020	Pregnancy, childbirth and the puerperium	935,000	11.2	995,000 (949,000 to 1,040,000)	12.0 (11.4 to 12.5)	-60,100 (-108,000 to -14,200)	-0.723 (-1.29 to -0.171)	0.94 (0.90 to 0.98)	3.5	3.62 (3.56 to 3.68)	-0.120 (-0.180 to -0.060)	0.97 (0.95 to 0.98)
Germany	2021	Pregnancy, childbirth and the puerperium	948,000	11.4	995,000 (931,000 to 1,060,000)	12.0 (11.2 to 12.8)	-46,800 (-117,000 to 17,800)	-0.563 (-1.40 to 0.214)	0.95 (0.89 to 1.02)	3.5	3.54 (3.46 to 3.62)	-0.043 (-0.125 to 0.037)	0.99 (0.97 to 1.01)
Germany	2020	Certain conditions originating in the perinatal period	190,000	2.29	194,000 (182,000 to 206,000)	2.33 (2.19 to 2.48)	-3,640 (-16,200 to 7,880)	-0.044 (-0.195 to 0.095)	0.98 (0.92 to 1.04)	8.4	8.46 (8.22 to 8.70)	-0.057 (-0.300 to 0.186)	0.99 (0.97 to 1.02)
Germany	2021	Certain conditions originating in the perinatal period	193,000	2.32	194,000 (178,000 to 212,000)	2.33 (2.14 to 2.55)	-1,400 (-19,400 to 14,800)	-0.017 (-0.233 to 0.177)	0.99 (0.91 to 1.08)	8.3	8.32 (7.98 to 8.66)	-0.017 (-0.361 to 0.321)	1.00 (0.96 to 1.04)
Germany	2020	Congenital malformations, deformations and chromosomal abnormalities	95,200	1.14	108,000 (106,000 to 111,000)	1.30 (1.27 to 1.33)	-13,000 (-15,700 to -10,400)	-0.157 (-0.189 to -0.125)	0.88 (0.86 to 0.90)	6.1	6.30 (6.11 to 6.49)	-0.200 (-0.387 to -0.013)	0.97 (0.94 to 1.00)
Germany	2021	Congenital malformations, deformations and chromosomal abnormalities	95,700	1.15	108,000 (105,000 to 112,000)	1.30 (1.26 to 1.35)	-12,500 (-16,400 to -8,850)	-0.150 (-0.197 to -0.106)	0.88 (0.85 to 0.92)	5.9	6.30 (6.04 to 6.57)	-0.400 (-0.672 to -0.141)	0.94 (0.90 to 0.98)

Location	Year	Condition	Observed hospitalizations	Observed hospitalizations per 1,000 population	Expected hospitalizations	Expected hospitalizations per 1,000 population	Difference in hospitalizations	Difference in rate	Hospitalizations observed-to-expected ratio	Observed length of stay	Expected length of stay	Difference in length of stay	Length of stay observed-to-expected ratio
Germany	2020	Symptoms, signs and abnormal clinical and laboratory findings, n.e.c.	696,000	8.37	861,000 (809,000 to 916,000)	10.4 (9.73 to 11.0)	-165,000 (-221,000 to -113,000)	-1.98 (-2.66 to -1.36)	0.81 (0.76 to 0.86)	4.8	4.90 (4.71 to 5.10)	-0.099 (-0.294 to 0.086)	0.98 (0.94 to 1.02)
Germany	2021	Symptoms, signs and abnormal clinical and laboratory findings, n.e.c.	698,000	8.39	851,000 (768,000 to 944,000)	10.2 (9.23 to 11.3)	-154,000 (-247,000 to -70,300)	-1.85 (-2.97 to -0.845)	0.82 (0.74 to 0.91)	4.9	4.90 (4.66 to 5.15)	0.002 (-0.249 to 0.248)	1.00 (0.95 to 1.05)
Germany	2020	Injury, poisoning and other consequences of external causes	1,810,000	21.7	2,020,000 (1,990,000 to 2,060,000)	24.3 (23.9 to 24.8)	-218,000 (-257,000 to -179,000)	-2.62 (-3.09 to -2.15)	0.89 (0.88 to 0.91)	7.9	7.94 (7.81 to 8.07)	-0.036 (-0.168 to 0.094)	1.00 (0.98 to 1.01)
Germany	2021	Injury, poisoning and other consequences of external causes	1,780,000	21.4	2,020,000 (1,970,000 to 2,080,000)	24.3 (23.7 to 25.0)	-244,000 (-300,000 to -190,000)	-2.94 (-3.61 to -2.29)	0.88 (0.86 to 0.90)	7.8	7.87 (7.69 to 8.06)	-0.072 (-0.255 to 0.109)	0.99 (0.97 to 1.01)
Germany	2020	Factors influencing health status and contact with health services	680,000	8.18	734,000 (696,000 to 774,000)	8.83 (8.37 to 9.30)	-53,500 (-93,600 to -15,500)	-0.644 (-1.13 to -0.186)	0.93 (0.88 to 0.98)	4.2	4.70 (4.14 to 5.34)	-0.500 (-1.14 to 0.080)	0.89 (0.79 to 1.02)
Germany	2021	Factors influencing health status and contact with health services	698,000	8.39	734,000 (681,000 to 791,000)	8.83 (8.19 to 9.51)	-36,200 (-93,400 to 15,900)	-0.435 (-1.12 to 0.192)	0.95 (0.88 to 1.02)	4.2	4.70 (3.93 to 5.63)	-0.500 (-1.42 to 0.265)	0.89 (0.75 to 1.07)
Spain	2020	All causes	4,120,000	86.9	4,900,000 (4,780,000 to 5,020,000)	103 (101 to 106)	-782,000 (-900,000 to -668,000)	-16.5 (-19.0 to -14.1)	0.84 (0.82 to 0.86)				
Spain	2021	All causes	4,270,000	90.1	4,900,000 (4,740,000 to 5,070,000)	103 (100.0 to 107)	-630,000 (-801,000 to -469,000)	-13.3 (-16.9 to -9.90)	0.87 (0.84 to 0.90)				
Spain	2020	Infectious and parasitic diseases	93,600	1.98	123,000 (115,000 to 131,000)	2.60 (2.44 to 2.77)	-29,400 (-37,500 to -21,800)	-0.620 (-0.793 to -0.461)	0.76 (0.71 to 0.81)	11	10.20 (9.48 to 10.97)	0.800 (0.037 to 1.53)	1.08 (1.00 to 1.16)
Spain	2021	Infectious and parasitic diseases	97,300	2.05	128,000 (117,000 to 140,000)	2.71 (2.47 to 2.96)	-31,100 (-43,300 to -20,100)	-0.655 (-0.912 to -0.424)	0.76 (0.69 to 0.83)	10.8	10.20 (9.20 to 11.31)	0.600 (-0.511 to 1.60)	1.06 (0.95 to 1.17)
Spain	2020	Neoplasms	409,000	8.64	472,000 (465,000 to 479,000)	9.96 (9.81 to 10.1)	-62,100 (-69,100 to -55,100)	-1.31 (-1.46 to -1.16)	0.87 (0.86 to 0.88)	7.9	8.22 (7.86 to 8.60)	-0.324 (-0.702 to 0.033)	0.96 (0.92 to 1.00)
Spain	2021	Neoplasms	439,000	9.25	475,000 (465,000 to 485,000)	10.0 (9.80 to 10.2)	-36,100 (-46,300 to -26,200)	-0.762 (-0.976 to -0.552)	0.92 (0.90 to 0.94)	7.6	8.24 (7.80 to 8.71)	-0.642 (-1.11 to -0.193)	0.92 (0.87 to 0.98)
Spain	2020	Diseases of the blood and bloodforming organs	38,200	0.807	43,600 (42,700 to 44,400)	0.920 (0.902 to 0.938)	-5,350 (-6,190 to -4,500)	-0.113 (-0.131 to -0.095)	0.88 (0.86 to 0.89)	7.6	7.60 (7.19 to 8.03)	0.000 (-0.443 to 0.400)	1.00 (0.94 to 1.06)
Spain	2021	Diseases of the blood and bloodforming organs	41,700	0.879	43,600 (42,800 to 44,500)	0.920 (0.902 to 0.938)	-1,930 (-2,780 to -1,110)	-0.041 (-0.059 to -0.023)	0.96 (0.94 to 0.97)	7.6	7.60 (7.03 to 8.22)	0.000 (-0.611 to 0.571)	1.00 (0.93 to 1.08)
Spain	2020	Endocrine, nutritional and metabolic diseases	75,700	1.6	88,700 (85,800 to 91,500)	1.87 (1.81 to 1.93)	-12,900 (-15,800 to -10,200)	-0.273 (-0.333 to -0.214)	0.85 (0.83 to 0.88)	7.3	7.10 (6.68 to 7.55)	0.200 (-0.244 to 0.613)	1.03 (0.97 to 1.09)
Spain	2021	Endocrine, nutritional and metabolic diseases	88,700	1.87	88,700 (84,800 to 92,900)	1.87 (1.79 to 1.96)	-15 (-4,190 to 3,890)	-0.000 (-0.088 to 0.082)	1.00 (0.95 to 1.05)	7	7.10 (6.19 to 8.15)	-0.100 (-1.15 to 0.807)	0.99 (0.86 to 1.13)

Location	Year	Condition	Observed hospitalizations	Observed hospitalizations per 1,000 population	Expected hospitalizations	Expected hospitalizations per 1,000 population	Difference in hospitalizations	Difference in rate	Hospitalizations observed-to-expected ratio	Observed length of stay	Expected length of stay	Difference in length of stay	Length of stay observed-to-expected ratio
Spain	2020	Mental and behavioural disorders	104,000	2.2	119,000 (114,000 to 123,000)	2.50 (2.41 to 2.60)	-14,400 (-19,000 to -10,000)	-0.304 (-0.401 to -0.211)	0.88 (0.85 to 0.91)	60.8	50.60 (35.04 to 73.07)	10.20 (-11.85 to 25.73)	1.20 (0.84 to 1.73)
Spain	2021	Mental and behavioural disorders	114,000	2.4	119,000 (113,000 to 125,000)	2.50 (2.37 to 2.64)	-4,850 (-11,400 to 1,150)	-0.102 (-0.241 to 0.024)	0.96 (0.91 to 1.01)	53.1	50.60 (30.10 to 85.08)	2.50 (-32.53 to 23.04)	1.05 (0.62 to 1.77)
Spain	2020	Diseases of the nervous system	98,500	2.08	122,000 (112,000 to 133,000)	2.57 (2.35 to 2.81)	-23,400 (-34,800 to -13,100)	-0.494 (-0.734 to -0.277)	0.81 (0.74 to 0.88)	10.7	11.20 (9.96 to 12.59)	-0.500 (-1.92 to 0.727)	0.96 (0.85 to 1.07)
Spain	2021	Diseases of the nervous system	108,000	2.27	122,000 (108,000 to 138,000)	2.57 (2.27 to 2.92)	-14,200 (-30,400 to 156)	-0.299 (-0.641 to 0.003)	0.88 (0.78 to 1.00)	10	11.20 (8.62 to 14.56)	-1.20 (-4.65 to 1.38)	0.89 (0.68 to 1.16)
Spain	2020	Diseases of the eye and adnexa	16,400	0.346	22,400 (20,600 to 24,300)	0.472 (0.434 to 0.513)	-5,940 (-7,850 to -4,150)	-0.125 (-0.166 to -0.088)	0.73 (0.68 to 0.80)	3.3	3.70 (3.35 to 4.09)	-0.400 (-0.789 to -0.045)	0.89 (0.81 to 0.99)
Spain	2021	Diseases of the eye and adnexa	17,600	0.372	21,200 (18,800 to 23,800)	0.446 (0.396 to 0.502)	-3,520 (-6,180 to -1,210)	-0.074 (-0.130 to -0.025)	0.83 (0.74 to 0.94)	3.4	3.70 (3.21 to 4.27)	-0.300 (-0.879 to 0.199)	0.92 (0.79 to 1.06)
Spain	2020	Diseases of the ear and mastoid process	14,900	0.314	22,700 (21,500 to 24,000)	0.480 (0.454 to 0.507)	-7,820 (-9,100 to -6,590)	-0.165 (-0.192 to -0.139)	0.66 (0.62 to 0.69)	2.9	2.90 (2.72 to 3.09)	0.000 (-0.185 to 0.178)	1.00 (0.94 to 1.07)
Spain	2021	Diseases of the ear and mastoid process	16,800	0.355	22,700 (21,000 to 24,600)	0.480 (0.443 to 0.519)	-5,900 (-7,760 to -4,170)	-0.124 (-0.164 to -0.088)	0.74 (0.68 to 0.80)	2.8	2.90 (2.65 to 3.17)	-0.100 (-0.367 to 0.147)	0.97 (0.88 to 1.06)
Spain	2020	Diseases of the circulatory system	524,000	11.1	617,000 (603,000 to 632,000)	13.0 (12.7 to 13.3)	-93,300 (-108,000 to -78,900)	-1.97 (-2.28 to -1.66)	0.85 (0.83 to 0.87)	8.6	9.10 (8.45 to 9.80)	-0.500 (-1.19 to 0.146)	0.95 (0.88 to 1.02)
Spain	2021	Diseases of the circulatory system	582,000	12.3	618,000 (598,000 to 639,000)	13.0 (12.6 to 13.5)	-35,500 (-56,100 to -15,500)	-0.749 (-1.18 to -0.328)	0.94 (0.91 to 0.97)	8.5	9.10 (8.20 to 10.10)	-0.600 (-1.59 to 0.297)	0.93 (0.84 to 1.04)
Spain	2020	Diseases of the respiratory system	529,000	11.2	603,000 (553,000 to 658,000)	12.7 (11.7 to 13.9)	-74,500 (-129,000 to -25,000)	-1.57 (-2.73 to -0.528)	0.88 (0.80 to 0.95)	8.3	7.30 (7.05 to 7.56)	1.00 (0.742 to 1.24)	1.14 (1.10 to 1.18)
Spain	2021	Diseases of the respiratory system	358,000	7.55	604,000 (534,000 to 683,000)	12.7 (11.3 to 14.4)	-246,000 (-326,000 to -177,000)	-5.18 (-6.87 to -3.74)	0.59 (0.52 to 0.67)	7.2	7.30 (6.94 to 7.67)	-0.100 (-0.473 to 0.255)	0.99 (0.94 to 1.04)
Spain	2020	Diseases of the digestive system	495,000	10.4	609,000 (581,000 to 638,000)	12.9 (12.3 to 13.5)	-114,000 (-143,000 to -86,400)	-2.41 (-3.03 to -1.82)	0.81 (0.78 to 0.85)	5.9	5.55 (5.36 to 5.74)	0.351 (0.158 to 0.539)	1.06 (1.03 to 1.10)
Spain	2021	Diseases of the digestive system	554,000	11.7	610,000 (571,000 to 651,000)	12.9 (12.0 to 13.7)	-55,800 (-96,800 to -17,200)	-1.18 (-2.04 to -0.362)	0.91 (0.85 to 0.97)	5.7	5.55 (5.36 to 5.74)	0.151 (-0.043 to 0.339)	1.03 (0.99 to 1.06)
Spain	2020	Diseases of the skin and subcutaneous tissue	43,000	0.907	56,800 (53,200 to 60,700)	1.20 (1.12 to 1.28)	-13,800 (-17,700 to -10,200)	-0.292 (-0.375 to -0.215)	0.76 (0.71 to 0.81)	9.2	8.70 (8.02 to 9.44)	0.500 (-0.250 to 1.19)	1.06 (0.97 to 1.15)
Spain	2021	Diseases of the skin and subcutaneous tissue	45,400	0.958	56,900 (51,800 to 62,500)	1.20 (1.09 to 1.32)	-11,500 (-17,200 to -6,340)	-0.242 (-0.364 to -0.134)	0.80 (0.72 to 0.88)	8.7	8.70 (7.75 to 9.77)	0.000 (-1.08 to 0.958)	1.00 (0.89 to 1.12)
Spain	2020	Diseases of musculoskeletal system and connective tissue	269,000	5.67	362,000 (347,000 to 377,000)	7.63 (7.32 to 7.96)	-92,900 (-108,000 to -77,900)	-1.96 (-2.28 to -1.64)	0.74 (0.71 to 0.78)	4.9	5.10 (4.75 to 5.48)	-0.200 (-0.581 to 0.154)	0.96 (0.89 to 1.03)
Spain	2021	Diseases of musculoskeletal system and connective tissue	309,000	6.51	362,000 (341,000 to 384,000)	7.63 (7.19 to 8.10)	-53,500 (-75,400 to -32,300)	-1.13 (-1.59 to -0.682)	0.85 (0.80 to 0.91)	4.7	5.10 (4.61 to 5.64)	-0.400 (-0.934 to 0.093)	0.92 (0.83 to 1.02)
Spain	2020	Diseases of the genitourinary system	294,000	6.21	359,000 (346,000 to 373,000)	7.58 (7.30 to 7.87)	-64,600 (-78,200 to -51,200)	-1.36 (-1.65 to -1.08)	0.82 (0.79 to 0.85)	5.2	5.10 (4.86 to 5.36)	0.100 (-0.158 to 0.344)	1.02 (0.97 to 1.07)

Location	Year	Condition	Observed hospitalizations	Observed hospitalizations per 1,000 population	Expected hospitalizations	Expected hospitalizations per 1,000 population	Difference in hospitalizations	Difference in rate	Hospitalizations observed-to-expected ratio	Observed length of stay	Expected length of stay	Difference in length of stay	Length of stay observed-to-expected ratio
Spain	2021	Diseases of the genitourinary system	332,000	7.01	366,000 (347,000 to 386,000)	7.73 (7.33 to 8.15)	-34,000 (-54,500 to -15,600)	-0.717 (-1.15 to -0.329)	0.91 (0.86 to 0.96)	5	5.10 (4.76 to 5.46)	-0.100 (-0.466 to 0.240)	0.98 (0.91 to 1.05)
Spain	2020	Pregnancy, childbirth and the puerperium	381,000	8.04	400,000 (381,000 to 420,000)	8.45 (8.05 to 8.86)	-19,400 (-39,400 to -538)	-0.410 (-0.832 to -0.011)	0.95 (0.91 to 1.00)	2.9	3.10 (2.95 to 3.26)	-0.200 (-0.358 to -0.054)	0.94 (0.89 to 0.98)
Spain	2021	Pregnancy, childbirth and the puerperium	375,000	7.9	385,000 (360,000 to 412,000)	8.12 (7.59 to 8.70)	-10,500 (-38,100 to 15,300)	-0.222 (-0.804 to 0.323)	0.97 (0.91 to 1.04)	3	3.10 (2.89 to 3.32)	-0.100 (-0.323 to 0.108)	0.97 (0.90 to 1.04)
Spain	2020	Certain conditions originating in the perinatal period	55,200	1.17	60,000 (54,200 to 66,500)	1.27 (1.14 to 1.40)	-4,830 (-11,100 to 1,060)	-0.102 (-0.234 to 0.022)	0.92 (0.83 to 1.02)	8.6	8.82 (8.62 to 9.02)	-0.219 (-0.426 to -0.017)	0.98 (0.95 to 1.00)
Spain	2021	Certain conditions originating in the perinatal period	56,500	1.19	60,100 (52,000 to 69,400)	1.27 (1.10 to 1.46)	-3,630 (-12,900 to 4,320)	-0.077 (-0.272 to 0.091)	0.94 (0.81 to 1.08)	8.3	8.82 (8.62 to 9.02)	-0.519 (-0.724 to -0.319)	0.94 (0.92 to 0.96)
Spain	2020	Congenital malformations, deformations and chromosomal abnormalities	27,700	0.585	34,900 (33,400 to 36,500)	0.737 (0.705 to 0.770)	-7,180 (-8,770 to -5,680)	-0.152 (-0.185 to -0.120)	0.79 (0.76 to 0.83)	5.2	5.20 (4.76 to 5.68)	0.000 (-0.486 to 0.445)	1.00 (0.91 to 1.09)
Spain	2021	Congenital malformations, deformations and chromosomal abnormalities	30,600	0.646	34,100 (32,000 to 36,300)	0.719 (0.675 to 0.766)	-3,480 (-5,720 to -1,400)	-0.073 (-0.121 to -0.029)	0.90 (0.84 to 0.96)	4.8	5.20 (4.59 to 5.90)	-0.400 (-1.10 to 0.205)	0.92 (0.81 to 1.04)
Spain	2020	Symptoms, signs and abnormal clinical and laboratory findings, n.e.c.	164,000	3.46	205,000 (189,000 to 222,000)	4.32 (3.98 to 4.68)	-40,800 (-57,600 to -25,000)	-0.862 (-1.22 to -0.528)	0.80 (0.74 to 0.87)	14.8	9.70 (6.29 to 14.95)	5.10 (-0.230 to 8.48)	1.53 (0.98 to 2.34)
Spain	2021	Symptoms, signs and abnormal clinical and laboratory findings, n.e.c.	173,000	3.65	205,000 (183,000 to 230,000)	4.32 (3.85 to 4.84)	-31,700 (-55,900 to -9,720)	-0.668 (-1.18 to -0.205)	0.85 (0.76 to 0.95)	12.6	9.70 (5.26 to 17.88)	2.90 (-5.42 to 7.33)	1.30 (0.70 to 2.39)
Spain	2020	Injury, poisoning and other consequences of external causes	384,000	8.11	462,000 (449,000 to 475,000)	9.75 (9.48 to 10.0)	-77,800 (-91,400 to -64,800)	-1.64 (-1.93 to -1.37)	0.83 (0.81 to 0.86)	8	8.47 (8.08 to 8.88)	-0.475 (-0.890 to -0.084)	0.94 (0.90 to 0.99)
Spain	2021	Injury, poisoning and other consequences of external causes	426,000	8.97	467,000 (449,000 to 487,000)	9.86 (9.47 to 10.3)	-42,000 (-61,000 to -23,200)	-0.885 (-1.29 to -0.488)	0.91 (0.87 to 0.95)	7.7	8.47 (7.73 to 9.29)	-0.775 (-1.59 to -0.043)	0.91 (0.83 to 0.99)
Spain	2020	Factors influencing health status and contact with health services	99,800	2.11	116,000 (107,000 to 127,000)	2.46 (2.25 to 2.69)	-16,600 (-27,400 to -6,680)	-0.351 (-0.577 to -0.141)	0.86 (0.78 to 0.94)	7.4	6.84 (6.01 to 7.78)	0.564 (-0.365 to 1.37)	1.08 (0.95 to 1.23)
Spain	2021	Factors influencing health status and contact with health services	109,000	2.3	115,000 (103,000 to 129,000)	2.44 (2.17 to 2.73)	-6,540 (-20,400 to 5,900)	-0.138 (-0.431 to 0.125)	0.94 (0.84 to 1.06)	6.8	6.84 (6.01 to 7.78)	-0.036 (-0.967 to 0.793)	0.99 (0.88 to 1.13)
Estonia	2020	All causes	180,000	135	202,000 (198,000 to 206,000)	152 (149 to 155)	-21,500 (-25,700 to -17,600)	-16.2 (-19.4 to -13.2)	0.89 (0.88 to 0.91)				
Estonia	2021	All causes	179,000	135	199,000 (193,000 to 205,000)	149 (145 to 154)	-19,700 (-25,200 to -14,300)	-14.8 (-19.0 to -10.7)	0.90 (0.88 to 0.93)				
Estonia	2020	Infectious and parasitic diseases	5,530	4.16	8,130 (7,140 to 9,260)	6.11 (5.37 to 6.97)	-2,600 (-3,710 to -1,610)	-1.96 (-2.79 to -1.21)	0.68 (0.60 to 0.77)	10.4	8.28 (7.40 to 9.27)	2.12 (1.13 to 2.99)	1.26 (1.12 to 1.40)

Location	Year	Condition	Observed hospitalizations	Observed hospitalizations per 1,000 population	Expected hospitalizations	Expected hospitalizations per 1,000 population	Difference in hospitalizations	Difference in rate	Hospitalizations observed-to-expected ratio	Observed length of stay	Expected length of stay	Difference in length of stay	Length of stay observed-to-expected ratio
Estonia	2021	Infectious and parasitic diseases	5,540	4.17	7,980 (6,950 to 9,170)	6.00 (5.22 to 6.89)	-2,440 (-3,620 to -1,420)	-1.83 (-2.72 to -1.07)	0.69 (0.60 to 0.80)	10.4	8.35 (7.39 to 9.43)	2.05 (0.993 to 2.98)	1.25 (1.11 to 1.40)
Estonia	2020	Neoplasms	24,900	18.7	27,100 (26,200 to 28,000)	20.4 (19.7 to 21.1)	-2,250 (-3,160 to -1,380)	-1.69 (-2.38 to -1.04)	0.92 (0.89 to 0.95)	7.7	7.30 (6.92 to 7.70)	0.400 (0.009 to 0.787)	1.05 (1.00 to 1.11)
Estonia	2021	Neoplasms	23,500	17.7	27,300 (25,400 to 29,400)	20.5 (19.1 to 22.1)	-3,780 (-5,940 to -1,850)	-2.84 (-4.46 to -1.39)	0.86 (0.80 to 0.93)	7.4	7.30 (6.77 to 7.87)	0.100 (-0.467 to 0.617)	1.01 (0.94 to 1.09)
Estonia	2020	Diseases of the blood and bloodforming organs	2,110	1.59	2,150 (2,010 to 2,310)	1.62 (1.51 to 1.74)	-39 (-190 to 106)	-0.030 (-0.143 to 0.080)	0.98 (0.92 to 1.05)	7	6.61 (6.15 to 7.09)	0.394 (-0.090 to 0.853)	1.06 (0.99 to 1.14)
Estonia	2021	Diseases of the blood and bloodforming organs	1,820	1.37	2,210 (2,000 to 2,440)	1.66 (1.50 to 1.83)	-386 (-615 to -178)	-0.290 (-0.462 to -0.134)	0.83 (0.75 to 0.91)	7.3	6.61 (6.15 to 7.09)	0.694 (0.204 to 1.15)	1.11 (1.03 to 1.19)
Estonia	2020	Endocrine, nutritional and metabolic diseases	3,900	2.94	4,100 (3,800 to 4,420)	3.08 (2.86 to 3.32)	-194 (-510 to 107)	-0.146 (-0.384 to 0.081)	0.95 (0.88 to 1.03)	7.2	7.38 (6.87 to 7.92)	-0.176 (-0.711 to 0.331)	0.98 (0.91 to 1.05)
Estonia	2021	Endocrine, nutritional and metabolic diseases	3,540	2.66	4,100 (3,690 to 4,560)	3.08 (2.77 to 3.43)	-561 (-1,030 to -152)	-0.422 (-0.772 to -0.114)	0.86 (0.78 to 0.96)	7	7.38 (6.87 to 7.92)	-0.376 (-0.922 to 0.132)	0.95 (0.88 to 1.02)
Estonia	2020	Mental and behavioural disorders	11,100	8.34	12,500 (11,700 to 13,300)	9.39 (8.79 to 10.0)	-1,390 (-2,260 to -615)	-1.05 (-1.70 to -0.462)	0.89 (0.83 to 0.95)	16.6	17.16 (15.65 to 18.82)	-0.562 (-2.25 to 0.956)	0.97 (0.88 to 1.06)
Estonia	2021	Mental and behavioural disorders	11,300	8.48	12,500 (11,400 to 13,700)	9.39 (8.55 to 10.3)	-1,210 (-2,460 to -80)	-0.911 (-1.85 to -0.060)	0.90 (0.82 to 0.99)	15.7	17.16 (15.65 to 18.82)	-1.46 (-3.12 to 0.071)	0.91 (0.83 to 1.00)
Estonia	2020	Diseases of the nervous system	6,160	4.64	7,480 (6,980 to 8,020)	5.63 (5.25 to 6.03)	-1,320 (-1,850 to -821)	-0.992 (-1.39 to -0.618)	0.82 (0.77 to 0.88)	7.7	7.60 (6.54 to 8.83)	0.100 (-1.16 to 1.19)	1.01 (0.87 to 1.18)
Estonia	2021	Diseases of the nervous system	6,310	4.74	7,490 (6,990 to 8,030)	5.63 (5.25 to 6.03)	-1,180 (-1,720 to -680)	-0.890 (-1.29 to -0.511)	0.84 (0.79 to 0.90)	7.4	7.60 (6.15 to 9.40)	-0.200 (-2.00 to 1.24)	0.97 (0.79 to 1.20)
Estonia	2020	Diseases of the eye and adnexa	1,750	1.32	1,910 (1,720 to 2,120)	1.44 (1.29 to 1.59)	-155 (-359 to 38)	-0.116 (-0.270 to 0.029)	0.92 (0.83 to 1.02)	1.7	1.60 (1.38 to 1.86)	0.100 (-0.167 to 0.332)	1.06 (0.91 to 1.24)
Estonia	2021	Diseases of the eye and adnexa	1,730	1.3	1,910 (1,650 to 2,210)	1.44 (1.24 to 1.66)	-178 (-476 to 91)	-0.134 (-0.358 to 0.068)	0.91 (0.78 to 1.06)	1.7	1.60 (1.29 to 1.98)	0.100 (-0.277 to 0.406)	1.06 (0.86 to 1.31)
Estonia	2020	Diseases of the ear and mastoid process	1,040	0.782	1,300 (1,130 to 1,510)	0.981 (0.849 to 1.13)	-264 (-466 to -90)	-0.199 (-0.351 to -0.067)	0.80 (0.69 to 0.92)	3.5	3.20 (2.84 to 3.60)	0.300 (-0.102 to 0.662)	1.09 (0.97 to 1.23)
Estonia	2021	Diseases of the ear and mastoid process	1,090	0.817	1,240 (1,020 to 1,530)	0.935 (0.763 to 1.15)	-158 (-440 to 74)	-0.118 (-0.331 to 0.056)	0.87 (0.71 to 1.07)	3.1	3.20 (2.71 to 3.78)	-0.100 (-0.680 to 0.397)	0.97 (0.82 to 1.15)
Estonia	2020	Diseases of the circulatory system	30,800	23.1	33,000 (31,400 to 34,600)	24.8 (23.7 to 26.1)	-2,230 (-3,870 to -655)	-1.68 (-2.91 to -0.492)	0.93 (0.89 to 0.98)	10.6	10.20 (9.59 to 10.85)	0.400 (-0.227 to 1.01)	1.04 (0.98 to 1.11)
Estonia	2021	Diseases of the circulatory system	28,100	21.1	32,100 (29,900 to 34,300)	24.1 (22.5 to 25.8)	-3,940 (-6,230 to -1,850)	-2.96 (-4.68 to -1.39)	0.88 (0.82 to 0.94)	10.3	10.20 (9.34 to 11.14)	0.100 (-0.822 to 0.965)	1.01 (0.93 to 1.10)
Estonia	2020	Diseases of the respiratory system	13,300	9.98	17,200 (16,300 to 18,100)	12.9 (12.2 to 13.6)	-3,910 (-4,860 to -2,980)	-2.94 (-3.66 to -2.24)	0.77 (0.73 to 0.82)	6.2	5.50 (5.14 to 5.88)	0.700 (0.311 to 1.07)	1.13 (1.05 to 1.21)
Estonia	2021	Diseases of the respiratory system	20,100	15.1	16,800 (15,500 to 18,100)	12.6 (11.7 to 13.6)	3,370 (2,040 to 4,610)	2.53 (1.53 to 3.47)	1.20 (1.11 to 1.30)	8.5	5.50 (5.00 to 6.05)	3.00 (2.46 to 3.50)	1.55 (1.41 to 1.70)
Estonia	2020	Diseases of the digestive system	14,800	11.1	16,500 (15,300 to 17,700)	12.4 (11.5 to 13.3)	-1,670 (-2,920 to -507)	-1.25 (-2.20 to -0.381)	0.90 (0.84 to 0.97)	5.6	5.20 (4.84 to 5.58)	0.400 (0.016 to 0.752)	1.08 (1.00 to 1.16)

Location	Year	Condition	Observed hospitalizations	Observed hospitalizations per 1,000 population	Expected hospitalizations	Expected hospitalizations per 1,000 population	Difference in hospitalizations	Difference in rate	Hospitalizations observed-to-expected ratio	Observed length of stay	Expected length of stay	Difference in length of stay	Length of stay observed-to-expected ratio
Estonia	2021	Diseases of the digestive system	14,300	10.8	16,500 (14,800 to 18,300)	12.4 (11.2 to 13.8)	-2,140 (-3,970 to -471)	-1.61 (-2.98 to -0.354)	0.87 (0.78 to 0.97)	5.3	5.20 (4.70 to 5.75)	0.100 (-0.450 to 0.596)	1.02 (0.92 to 1.13)
Estonia	2020	Diseases of the skin and subcutaneous tissue	2,960	2.23	3,100 (2,420 to 3,970)	2.33 (1.82 to 2.98)	-136 (-983 to 555)	-0.102 (-0.740 to 0.417)	0.96 (0.75 to 1.23)	10.2	10.20 (8.38 to 12.42)	0.000 (-2.26 to 1.77)	1.00 (0.82 to 1.21)
Estonia	2021	Diseases of the skin and subcutaneous tissue	2,900	2.18	3,100 (2,190 to 4,400)	2.33 (1.64 to 3.30)	-201 (-1,500 to 707)	-0.151 (-1.13 to 0.531)	0.94 (0.66 to 1.32)	8.9	10.20 (7.72 to 13.47)	-1.30 (-4.63 to 1.22)	0.87 (0.66 to 1.16)
Estonia	2020	Diseases of musculoskeletal system and connective tissue	11,700	8.8	13,100 (12,300 to 13,900)	9.83 (9.26 to 10.4)	-1,380 (-2,180 to -606)	-1.03 (-1.64 to -0.456)	0.89 (0.84 to 0.95)	6.8	6.70 (6.41 to 7.01)	0.100 (-0.214 to 0.395)	1.01 (0.97 to 1.06)
Estonia	2021	Diseases of musculoskeletal system and connective tissue	10,500	7.9	13,100 (12,000 to 14,300)	9.83 (9.03 to 10.7)	-2,570 (-3,730 to -1,530)	-1.93 (-2.80 to -1.15)	0.80 (0.74 to 0.87)	6.5	6.70 (6.29 to 7.14)	-0.200 (-0.635 to 0.219)	0.97 (0.91 to 1.03)
Estonia	2020	Diseases of the genitourinary system	10,100	7.63	11,200 (10,700 to 11,800)	8.43 (8.01 to 8.88)	-1,070 (-1,650 to -525)	-0.807 (-1.24 to -0.395)	0.90 (0.86 to 0.95)	5.9	5.60 (5.03 to 6.23)	0.300 (-0.321 to 0.865)	1.05 (0.95 to 1.17)
Estonia	2021	Diseases of the genitourinary system	9,700	7.29	11,000 (10,200 to 11,800)	8.27 (7.69 to 8.89)	-1,300 (-2,120 to -530)	-0.980 (-1.59 to -0.398)	0.88 (0.82 to 0.95)	6.1	5.60 (4.82 to 6.51)	0.500 (-0.418 to 1.30)	1.09 (0.94 to 1.27)
Estonia	2020	Pregnancy, childbirth and the puerperium	17,000	12.8	18,500 (17,500 to 19,600)	13.9 (13.2 to 14.7)	-1,520 (-2,600 to -504)	-1.15 (-1.95 to -0.379)	0.92 (0.87 to 0.97)	2.9	2.80 (2.73 to 2.87)	0.100 (0.034 to 0.166)	1.04 (1.01 to 1.06)
Estonia	2021	Pregnancy, childbirth and the puerperium	16,200	12.2	18,000 (16,600 to 19,500)	13.5 (12.5 to 14.7)	-1,780 (-3,280 to -384)	-1.34 (-2.46 to -0.289)	0.90 (0.83 to 0.98)	3	2.80 (2.71 to 2.90)	0.200 (0.102 to 0.292)	1.07 (1.04 to 1.11)
Estonia	2020	Certain conditions originating in the perinatal period	3,520	2.65	3,650 (3,340 to 4,000)	2.75 (2.51 to 3.01)	-137 (-485 to 181)	-0.103 (-0.365 to 0.136)	0.96 (0.88 to 1.05)	6.5	6.40 (5.94 to 6.90)	0.100 (-0.393 to 0.568)	1.02 (0.94 to 1.10)
Estonia	2021	Certain conditions originating in the perinatal period	3,650	2.74	3,660 (3,340 to 4,000)	2.75 (2.51 to 3.01)	-9 (-357 to 299)	-0.007 (-0.268 to 0.225)	1.00 (0.91 to 1.09)	6	6.40 (5.75 to 7.12)	-0.400 (-1.11 to 0.252)	0.94 (0.84 to 1.04)
Estonia	2020	Congenital malformations, deformations and chromosomal abnormalities	1,680	1.26	2,050 (1,930 to 2,170)	1.54 (1.45 to 1.63)	-368 (-491 to -250)	-0.277 (-0.370 to -0.188)	0.82 (0.77 to 0.87)	4.6	5.08 (4.60 to 5.62)	-0.484 (-1.02 to 0.001)	0.90 (0.82 to 1.00)
Estonia	2021	Congenital malformations, deformations and chromosomal abnormalities	1,690	1.27	2,050 (1,880 to 2,230)	1.54 (1.42 to 1.67)	-355 (-536 to -188)	-0.267 (-0.403 to -0.141)	0.83 (0.76 to 0.90)	4.7	5.08 (4.60 to 5.62)	-0.384 (-0.938 to 0.106)	0.92 (0.83 to 1.02)
Estonia	2020	Symptoms, signs and abnormal clinical and laboratory findings, n.e.c.	1,870	1.41	2,400 (2,280 to 2,530)	1.81 (1.72 to 1.90)	-533 (-659 to -415)	-0.401 (-0.495 to -0.312)	0.78 (0.74 to 0.82)	4.1	3.80 (3.27 to 4.42)	0.300 (-0.314 to 0.826)	1.08 (0.93 to 1.25)
Estonia	2021	Symptoms, signs and abnormal clinical and laboratory findings, n.e.c.	1,820	1.37	2,400 (2,290 to 2,530)	1.81 (1.72 to 1.90)	-587 (-712 to -471)	-0.441 (-0.535 to -0.354)	0.76 (0.72 to 0.79)	3.8	3.80 (3.07 to 4.70)	0.000 (-0.907 to 0.726)	1.00 (0.81 to 1.24)

Location	Year	Condition	Observed hospitalizations	Observed hospitalizations per 1,000 population	Expected hospitalizations	Expected hospitalizations per 1,000 population	Difference in hospitalizations	Difference in rate	Hospitalizations observed-to-expected ratio	Observed length of stay	Expected length of stay	Difference in length of stay	Length of stay observed-to-expected ratio
Estonia	2020	Injury, poisoning and other consequences of external causes	14,400	10.8	15,600 (14,900 to 16,300)	11.7 (11.2 to 12.3)	-1,200 (-1,930 to -519)	-0.904 (-1.45 to -0.390)	0.92 (0.88 to 0.97)	8.3	8.01 (7.69 to 8.34)	0.292 (-0.038 to 0.621)	1.04 (1.00 to 1.08)
Estonia	2021	Injury, poisoning and other consequences of external causes	13,800	10.4	15,600 (14,900 to 16,300)	11.7 (11.2 to 12.3)	-1,750 (-2,480 to -1,060)	-1.32 (-1.86 to -0.798)	0.89 (0.85 to 0.93)	8.3	8.01 (7.69 to 8.34)	0.292 (-0.038 to 0.614)	1.04 (1.00 to 1.08)
Estonia	2020	Factors influencing health status and contact with health services	1,600	1.2	1,690 (1,350 to 2,120)	1.27 (1.02 to 1.59)	-96 (-522 to 243)	-0.072 (-0.393 to 0.183)	0.94 (0.75 to 1.18)	4.9	5.88 (3.03 to 11.42)	-0.979 (-6.59 to 1.88)	0.83 (0.43 to 1.62)
Estonia	2021	Factors influencing health status and contact with health services	1,480	1.11	1,700 (1,240 to 2,330)	1.27 (0.928 to 1.75)	-214 (-838 to 240)	-0.161 (-0.629 to 0.180)	0.87 (0.64 to 1.19)	3.7	5.88 (3.03 to 11.42)	-2.18 (-7.93 to 0.714)	0.63 (0.32 to 1.24)
France	2020	All causes	10,600,000	157	12,300,000 (11,200,000 to 13,500,000)	183 (166 to 200)	-1,700,000 (-2,900,000 to -630,000)	-25.1 (-42.9 to -9.32)	0.86 (0.79 to 0.94)				
France	2021	All causes	10,900,000	161	12,400,000 (10,800,000 to 14,100,000)	183 (160 to 208)	-1,450,000 (-3,210,000 to 71,000)	-21.4 (-47.3 to 1.05)	0.88 (0.77 to 1.01)				
France	2020	Infectious and parasitic diseases	152,000	2.24	219,000 (211,000 to 226,000)	3.24 (3.13 to 3.35)	-67,000 (-74,400 to -59,700)	-0.991 (-1.10 to -0.883)	0.69 (0.67 to 0.72)	7.5	6.50 (6.32 to 6.68)	1.00 (0.817 to 1.18)	1.15 (1.12 to 1.19)
France	2021	Infectious and parasitic diseases	169,000	2.49	219,000 (209,000 to 230,000)	3.24 (3.09 to 3.39)	-50,200 (-61,100 to -40,200)	-0.741 (-0.902 to -0.593)	0.77 (0.73 to 0.81)	6.6	6.50 (6.25 to 6.76)	0.100 (-0.165 to 0.347)	1.02 (0.98 to 1.06)
France	2020	Neoplasms	649,000	9.61	706,000 (684,000 to 728,000)	10.4 (10.1 to 10.8)	-56,400 (-78,000 to -35,100)	-0.835 (-1.15 to -0.519)	0.92 (0.89 to 0.95)	7.2	7.20 (7.05 to 7.36)	0.000 (-0.160 to 0.154)	1.00 (0.98 to 1.02)
France	2021	Neoplasms	670,000	9.88	701,000 (671,000 to 732,000)	10.3 (9.90 to 10.8)	-31,100 (-61,000 to -1,480)	-0.460 (-0.900 to -0.022)	0.96 (0.92 to 1.00)	6.8	7.20 (6.98 to 7.42)	-0.400 (-0.626 to -0.189)	0.94 (0.92 to 0.97)
France	2020	Diseases of the blood and bloodforming organs	153,000	2.27	175,000 (171,000 to 179,000)	2.59 (2.53 to 2.64)	-21,600 (-25,400 to -17,800)	-0.319 (-0.376 to -0.263)	0.88 (0.86 to 0.90)	6.3	6.16 (6.06 to 6.26)	0.143 (0.041 to 0.241)	1.02 (1.01 to 1.04)
France	2021	Diseases of the blood and bloodforming organs	155,000	2.29	180,000 (174,000 to 185,000)	2.65 (2.57 to 2.74)	-24,400 (-30,100 to -19,000)	-0.361 (-0.444 to -0.280)	0.86 (0.84 to 0.89)	6.1	6.11 (5.98 to 6.26)	-0.014 (-0.162 to 0.127)	1.00 (0.97 to 1.02)
France	2020	Endocrine, nutritional and metabolic diseases	237,000	3.5	304,000 (290,000 to 319,000)	4.50 (4.29 to 4.72)	-67,400 (-82,100 to -53,500)	-0.998 (-1.22 to -0.792)	0.78 (0.74 to 0.82)	6.2	5.90 (5.72 to 6.08)	0.300 (0.116 to 0.478)	1.05 (1.02 to 1.08)
France	2021	Endocrine, nutritional and metabolic diseases	256,000	3.78	305,000 (291,000 to 320,000)	4.50 (4.29 to 4.72)	-48,800 (-63,600 to -34,800)	-0.720 (-0.938 to -0.514)	0.84 (0.80 to 0.88)	5.8	5.90 (5.65 to 6.16)	-0.100 (-0.357 to 0.151)	0.98 (0.94 to 1.03)
France	2020	Mental and behavioural disorders	671,000	9.93	775,000 (360,000 to 1,670,000)	11.5 (5.33 to 24.7)	-104,000 (-1,000,000 to 308,000)	-1.53 (-14.8 to 4.56)	0.87 (0.40 to 1.85)	25.3	23.20 (9.51 to 56.59)	2.10 (-30.62 to 15.79)	1.09 (0.45 to 2.66)
France	2021	Mental and behavioural disorders	693,000	10.2	777,000 (263,000 to 2,290,000)	11.5 (3.88 to 33.9)	-84,100 (-1,590,000 to 433,000)	-1.24 (-23.4 to 6.39)	0.89 (0.30 to 2.67)	24.2	23.20 (6.57 to 81.88)	1.00 (-57.12 to 17.70)	1.04 (0.30 to 3.72)
France	2020	Diseases of the nervous system	207,000	3.06	235,000 (230,000 to 240,000)	3.48 (3.40 to 3.56)	-28,300 (-33,900 to -23,000)	-0.419 (-0.502 to -0.340)	0.88 (0.86 to 0.90)	6.2	6.10 (6.01 to 6.19)	0.100 (0.008 to 0.190)	1.02 (1.00 to 1.03)

Location	Year	Condition	Observed hospitalizations	Observed hospitalizations per 1,000 population	Expected hospitalizations	Expected hospitalizations per 1,000 population	Difference in hospitalizations	Difference in rate	Hospitalizations observed-to-expected ratio	Observed length of stay	Expected length of stay	Difference in length of stay	Length of stay observed-to-expected ratio
France	2021	Diseases of the nervous system	204,000	3.01	230,000 (222,000 to 237,000)	3.39 (3.28 to 3.50)	-26,100 (-33,700 to -18,600)	-0.385 (-0.497 to -0.274)	0.89 (0.86 to 0.92)	6.2	6.10 (5.97 to 6.23)	0.100 (-0.032 to 0.226)	1.02 (0.99 to 1.04)
France	2020	Diseases of the eye and adnexa	66,400	0.983	88,600 (84,800 to 92,500)	1.31 (1.26 to 1.37)	-22,100 (-26,100 to -18,300)	-0.328 (-0.386 to -0.272)	0.75 (0.72 to 0.78)	2.4	2.24 (2.13 to 2.35)	0.164 (0.055 to 0.270)	1.07 (1.02 to 1.13)
France	2021	Diseases of the eye and adnexa	67,400	0.995	81,000 (76,100 to 86,100)	1.19 (1.12 to 1.27)	-13,500 (-18,500 to -8,660)	-0.199 (-0.273 to -0.128)	0.83 (0.78 to 0.89)	2.3	2.27 (2.12 to 2.43)	0.027 (-0.131 to 0.176)	1.01 (0.95 to 1.08)
France	2020	Diseases of the ear and mastoid process	38,800	0.574	50,500 (49,500 to 51,600)	0.748 (0.732 to 0.763)	-11,700 (-12,800 to -10,600)	-0.173 (-0.189 to -0.157)	0.77 (0.75 to 0.79)	2.9	2.87 (2.78 to 2.96)	0.032 (-0.063 to 0.124)	1.01 (0.98 to 1.04)
France	2021	Diseases of the ear and mastoid process	40,700	0.601	48,900 (47,900 to 50,000)	0.722 (0.707 to 0.738)	-8,200 (-9,280 to -7,180)	-0.121 (-0.137 to -0.106)	0.83 (0.81 to 0.85)	2.8	2.84 (2.71 to 2.97)	-0.037 (-0.168 to 0.090)	0.99 (0.94 to 1.03)
France	2020	Diseases of the circulatory system	1,210,000	17.9	1,360,000 (1,330,000 to 1,390,000)	20.1 (19.6 to 20.6)	-146,000 (-179,000 to -114,000)	-2.16 (-2.65 to -1.69)	0.89 (0.87 to 0.91)	6.4	6.50 (6.32 to 6.69)	-0.100 (-0.284 to 0.086)	0.98 (0.96 to 1.01)
France	2021	Diseases of the circulatory system	1,260,000	18.6	1,360,000 (1,320,000 to 1,410,000)	20.1 (19.4 to 20.8)	-105,000 (-150,000 to -59,100)	-1.54 (-2.22 to -0.873)	0.92 (0.89 to 0.96)	6.3	6.50 (6.24 to 6.77)	-0.200 (-0.468 to 0.056)	0.97 (0.93 to 1.01)
France	2020	Diseases of the respiratory system	527,000	7.8	728,000 (685,000 to 774,000)	10.8 (10.1 to 11.5)	-201,000 (-248,000 to -158,000)	-2.98 (-3.66 to -2.34)	0.72 (0.68 to 0.77)	6.9	6.70 (6.43 to 6.98)	0.200 (-0.085 to 0.474)	1.03 (0.99 to 1.07)
France	2021	Diseases of the respiratory system	530,000	7.82	730,000 (670,000 to 796,000)	10.8 (9.89 to 11.7)	-200,000 (-266,000 to -140,000)	-2.95 (-3.92 to -2.07)	0.73 (0.67 to 0.79)	6.3	6.70 (6.32 to 7.10)	-0.400 (-0.804 to -0.025)	0.94 (0.89 to 1.00)
France	2020	Diseases of the digestive system	769,000	11.4	859,000 (841,000 to 879,000)	12.7 (12.4 to 13.0)	-90,100 (-109,000 to -71,300)	-1.33 (-1.62 to -1.05)	0.90 (0.88 to 0.92)	5.3	5.20 (5.09 to 5.31)	0.100 (-0.011 to 0.209)	1.02 (1.00 to 1.04)
France	2021	Diseases of the digestive system	791,000	11.7	847,000 (821,000 to 874,000)	12.5 (12.1 to 12.9)	-56,500 (-83,200 to -30,300)	-0.833 (-1.23 to -0.447)	0.93 (0.90 to 0.96)	5.2	5.20 (5.05 to 5.36)	0.000 (-0.159 to 0.154)	1.00 (0.97 to 1.03)
France	2020	Diseases of the skin and subcutaneous tissue	98,400	1.46	119,000 (116,000 to 123,000)	1.76 (1.72 to 1.81)	-20,800 (-24,100 to -17,500)	-0.308 (-0.357 to -0.259)	0.83 (0.80 to 0.85)	6.4	6.30 (6.12 to 6.48)	0.100 (-0.086 to 0.279)	1.02 (0.99 to 1.05)
France	2021	Diseases of the skin and subcutaneous tissue	95,800	1.41	115,000 (108,000 to 123,000)	1.70 (1.60 to 1.81)	-19,400 (-26,700 to -12,500)	-0.286 (-0.394 to -0.184)	0.83 (0.78 to 0.88)	6.5	6.30 (6.05 to 6.56)	0.200 (-0.051 to 0.446)	1.03 (0.99 to 1.07)
France	2020	Diseases of musculoskeletal system and connective tissue	580,000	8.59	724,000 (704,000 to 745,000)	10.7 (10.4 to 11.0)	-144,000 (-165,000 to -124,000)	-2.13 (-2.44 to -1.84)	0.80 (0.78 to 0.82)	5.4	5.31 (5.16 to 5.46)	0.092 (-0.065 to 0.242)	1.02 (0.99 to 1.05)
France	2021	Diseases of musculoskeletal system and connective tissue	621,000	9.16	712,000 (669,000 to 758,000)	10.5 (9.87 to 11.2)	-91,600 (-138,000 to -48,400)	-1.35 (-2.03 to -0.714)	0.87 (0.82 to 0.93)	5.2	5.22 (5.01 to 5.44)	-0.018 (-0.233 to 0.193)	1.00 (0.96 to 1.04)
France	2020	Diseases of the genitourinary system	512,000	7.57	581,000 (576,000 to 585,000)	8.59 (8.53 to 8.66)	-69,100 (-73,500 to -64,800)	-1.02 (-1.09 to -0.959)	0.88 (0.87 to 0.89)	4.7	4.57 (4.48 to 4.67)	0.130 (0.035 to 0.225)	1.03 (1.01 to 1.05)
France	2021	Diseases of the genitourinary system	530,000	7.82	579,000 (570,000 to 590,000)	8.55 (8.40 to 8.70)	-49,200 (-59,300 to -39,100)	-0.727 (-0.875 to -0.578)	0.92 (0.90 to 0.93)	4.6	4.57 (4.48 to 4.67)	0.030 (-0.067 to 0.124)	1.01 (0.99 to 1.03)
France	2020	Pregnancy, childbirth and the puerperium	863,000	12.8	887,000 (877,000 to 897,000)	13.1 (13.0 to 13.3)	-24,700 (-35,100 to -14,700)	-0.365 (-0.519 to -0.217)	0.97 (0.96 to 0.98)	4.1	4.26 (4.16 to 4.36)	-0.158 (-0.257 to -0.060)	0.96 (0.94 to 0.99)

Location	Year	Condition	Observed hospitalizations	Observed hospitalizations per 1,000 population	Expected hospitalizations	Expected hospitalizations per 1,000 population	Difference in hospitalizations	Difference in rate	Hospitalizations observed-to-expected ratio	Observed length of stay	Expected length of stay	Difference in length of stay	Length of stay observed-to-expected ratio
France	2021	Pregnancy, childbirth and the puerperium	874,000	12.9	875,000 (861,000 to 889,000)	12.9 (12.7 to 13.1)	-1,200 (-15,500 to 13,000)	-0.018 (-0.228 to 0.192)	1.00 (0.98 to 1.02)	4.2	4.22 (4.08 to 4.36)	-0.016 (-0.155 to 0.118)	1.00 (0.96 to 1.03)
France	2020	Certain conditions originating in the perinatal period	127,000	1.88	122,000 (110,000 to 135,000)	1.81 (1.63 to 2.00)	4,900 (-8,440 to 17,000)	0.073 (-0.125 to 0.251)	1.04 (0.94 to 1.15)	10.9	11.29 (10.66 to 11.96)	-0.390 (-1.06 to 0.230)	0.97 (0.91 to 1.02)
France	2021	Certain conditions originating in the perinatal period	132,000	1.94	116,000 (100,000 to 134,000)	1.71 (1.48 to 1.98)	15,900 (-2,370 to 31,700)	0.235 (-0.035 to 0.467)	1.14 (0.98 to 1.32)	10.7	11.59 (10.69 to 12.57)	-0.889 (-1.88 to 0.032)	0.92 (0.85 to 1.00)
France	2020	Congenital malformations, deformations and chromosomal abnormalities	39,300	0.582	45,200 (43,700 to 46,900)	0.670 (0.646 to 0.694)	-5,910 (-7,490 to -4,310)	-0.087 (-0.111 to -0.064)	0.87 (0.84 to 0.90)	5.6	5.50 (5.31 to 5.70)	0.100 (-0.096 to 0.290)	1.02 (0.98 to 1.05)
France	2021	Congenital malformations, deformations and chromosomal abnormalities	42,500	0.628	43,600 (41,500 to 45,900)	0.644 (0.612 to 0.677)	-1,080 (-3,300 to 1,050)	-0.016 (-0.049 to 0.016)	0.98 (0.93 to 1.03)	5.3	5.50 (5.23 to 5.78)	-0.200 (-0.482 to 0.066)	0.96 (0.92 to 1.01)
France	2020	Symptoms, signs and abnormal clinical and laboratory findings, n.e.c.	651,000	9.63	751,000 (705,000 to 801,000)	11.1 (10.4 to 11.8)	-101,000 (-149,000 to -53,500)	-1.49 (-2.21 to -0.791)	0.87 (0.81 to 0.92)	5	5.00 (4.14 to 6.04)	0.000 (-1.05 to 0.875)	1.00 (0.83 to 1.21)
France	2021	Symptoms, signs and abnormal clinical and laboratory findings, n.e.c.	655,000	9.67	754,000 (689,000 to 824,000)	11.1 (10.2 to 12.2)	-98,300 (-170,000 to -34,600)	-1.45 (-2.51 to -0.511)	0.87 (0.79 to 0.95)	4.9	5.00 (3.83 to 6.53)	-0.100 (-1.60 to 1.07)	0.98 (0.75 to 1.28)
France	2020	Injury, poisoning and other consequences of external causes	765,000	11.3	847,000 (831,000 to 862,000)	12.5 (12.3 to 12.8)	-81,100 (-97,100 to -65,700)	-1.20 (-1.44 to -0.972)	0.90 (0.89 to 0.92)	5.3	5.40 (5.26 to 5.55)	-0.100 (-0.242 to 0.043)	0.98 (0.96 to 1.01)
France	2021	Injury, poisoning and other consequences of external causes	796,000	11.7	842,000 (820,000 to 864,000)	12.4 (12.1 to 12.8)	-46,000 (-68,400 to -23,800)	-0.679 (-1.01 to -0.352)	0.95 (0.92 to 0.97)	5.3	5.40 (5.20 to 5.61)	-0.100 (-0.313 to 0.100)	0.98 (0.94 to 1.02)
France	2020	Factors influencing health status and contact with health services	2,320,000	34.4	2,690,000 (1,940,000 to 3,730,000)	39.8 (28.7 to 55.2)	-367,000 (-1,440,000 to 387,000)	-5.43 (-21.2 to 5.72)	0.86 (0.62 to 1.20)	15.3	14.80 (6.44 to 34.02)	0.500 (-18.72 to 8.90)	1.03 (0.45 to 2.39)
France	2021	Factors influencing health status and contact with health services	2,350,000	34.6	2,700,000 (1,700,000 to 4,290,000)	39.8 (25.1 to 63.2)	-350,000 (-1,920,000 to 641,000)	-5.16 (-28.3 to 9.46)	0.87 (0.55 to 1.38)	14.7	14.80 (4.56 to 48.02)	-0.100 (-33.12 to 10.19)	0.99 (0.31 to 3.26)
Hungary	2020	All causes	1,360,000	140	1,840,000 (1,800,000 to 1,880,000)	189 (185 to 193)	-479,000 (-517,000 to -442,000)	-49.1 (-53.0 to -45.3)	0.74 (0.72 to 0.76)				
Hungary	2021	All causes	1,070,000	111	1,820,000 (1,770,000 to 1,870,000)	187 (182 to 193)	-743,000 (-796,000 to -692,000)	-76.6 (-82.0 to -71.3)	0.59 (0.57 to 0.61)				
Hungary	2020	Infectious and parasitic diseases	37,500	3.85	54,800 (51,000 to 58,700)	5.62 (5.24 to 6.02)	-17,200 (-21,200 to -13,500)	-1.77 (-2.17 to -1.38)	0.69 (0.64 to 0.74)	10.8	10.20 (9.50 to 10.95)	0.600 (-0.138 to 1.28)	1.06 (0.99 to 1.13)
Hungary	2021	Infectious and parasitic diseases	32,500	3.35	57,600 (52,100 to 63,600)	5.93 (5.37 to 6.55)	-25,100 (-30,900 to -19,800)	-2.58 (-3.19 to -2.04)	0.56 (0.51 to 0.62)	9.5	10.20 (9.23 to 11.27)	-0.700 (-1.76 to 0.280)	0.93 (0.84 to 1.03)

Location	Year	Condition	Observed hospitalizations	Observed hospitalizations per 1,000 population	Expected hospitalizations	Expected hospitalizations per 1,000 population	Difference in hospitalizations	Difference in rate	Hospitalizations observed-to-expected ratio	Observed length of stay	Expected length of stay	Difference in length of stay	Length of stay observed-to-expected ratio
Hungary	2020	Neoplasms	182,000	18.7	231,000 (223,000 to 239,000)	23.7 (22.9 to 24.5)	-49,000 (-57,700 to -40,900)	-5.03 (-5.92 to -4.20)	0.79 (0.76 to 0.82)	6.4	6.39 (6.27 to 6.52)	0.007 (-0.119 to 0.131)	1.00 (0.98 to 1.02)
Hungary	2021	Neoplasms	144,000	14.8	230,000 (222,000 to 238,000)	23.7 (22.9 to 24.5)	-86,100 (-94,500 to -78,200)	-8.87 (-9.73 to -8.05)	0.63 (0.60 to 0.65)	5.9	6.29 (6.12 to 6.47)	-0.388 (-0.565 to -0.215)	0.94 (0.91 to 0.96)
Hungary	2020	Diseases of the blood and bloodforming organs	18,900	1.93	25,200 (23,800 to 26,600)	2.58 (2.44 to 2.73)	-6,300 (-7,810 to -4,930)	-0.646 (-0.801 to -0.506)	0.75 (0.71 to 0.79)	7.7	7.57 (7.25 to 7.90)	0.132 (-0.193 to 0.448)	1.02 (0.98 to 1.06)
Hungary	2021	Diseases of the blood and bloodforming organs	16,000	1.65	25,200 (23,500 to 27,000)	2.59 (2.42 to 2.78)	-9,170 (-11,000 to -7,490)	-0.944 (-1.14 to -0.772)	0.64 (0.59 to 0.68)	6.7	7.57 (7.25 to 7.90)	-0.868 (-1.20 to -0.558)	0.89 (0.85 to 0.92)
Hungary	2020	Endocrine, nutritional and metabolic diseases	30,100	3.08	46,800 (42,100 to 52,100)	4.80 (4.32 to 5.34)	-16,800 (-22,000 to -12,100)	-1.72 (-2.25 to -1.24)	0.64 (0.58 to 0.71)	7	6.42 (5.93 to 6.94)	0.585 (0.075 to 1.06)	1.09 (1.01 to 1.18)
Hungary	2021	Endocrine, nutritional and metabolic diseases	24,400	2.51	44,800 (38,500 to 52,000)	4.61 (3.97 to 5.36)	-20,400 (-27,700 to -14,100)	-2.10 (-2.86 to -1.45)	0.54 (0.47 to 0.63)	5.8	6.42 (5.93 to 6.94)	-0.615 (-1.14 to -0.130)	0.90 (0.84 to 0.98)
Hungary	2020	Mental and behavioural disorders	62,100	6.37	82,000 (78,900 to 85,200)	8.41 (8.10 to 8.74)	-19,900 (-23,200 to -16,800)	-2.04 (-2.38 to -1.73)	0.76 (0.73 to 0.79)	39.3	33.32 (31.62 to 35.12)	5.98 (4.17 to 7.72)	1.18 (1.12 to 1.24)
Hungary	2021	Mental and behavioural disorders	49,800	5.12	79,000 (74,800 to 83,400)	8.14 (7.71 to 8.59)	-29,200 (-33,800 to -25,000)	-3.01 (-3.48 to -2.58)	0.63 (0.60 to 0.67)	31.3	33.96 (31.52 to 36.58)	-2.66 (-5.29 to -0.169)	0.92 (0.86 to 0.99)
Hungary	2020	Diseases of the nervous system	34,300	3.52	50,300 (48,700 to 52,000)	5.16 (4.99 to 5.33)	-16,000 (-17,600 to -14,300)	-1.64 (-1.81 to -1.47)	0.68 (0.66 to 0.71)	13.4	12.40 (11.78 to 13.05)	1.00 (0.360 to 1.63)	1.08 (1.03 to 1.14)
Hungary	2021	Diseases of the nervous system	26,400	2.72	49,300 (47,100 to 51,700)	5.08 (4.85 to 5.32)	-22,900 (-25,300 to -20,600)	-2.36 (-2.60 to -2.12)	0.54 (0.51 to 0.56)	11.3	12.40 (11.54 to 13.33)	-1.10 (-2.01 to -0.238)	0.91 (0.85 to 0.98)
Hungary	2020	Diseases of the eye and adnexa	30,800	3.16	54,200 (49,300 to 59,700)	5.56 (5.05 to 6.12)	-23,400 (-28,800 to -18,400)	-2.40 (-2.95 to -1.89)	0.57 (0.52 to 0.63)	2.1	1.81 (1.70 to 1.92)	0.294 (0.181 to 0.397)	1.16 (1.09 to 1.23)
Hungary	2021	Diseases of the eye and adnexa	20,300	2.09	54,000 (47,100 to 61,800)	5.56 (4.86 to 6.37)	-33,700 (-41,500 to -26,900)	-3.47 (-4.27 to -2.77)	0.38 (0.33 to 0.43)	2.1	1.78 (1.68 to 1.90)	0.315 (0.201 to 0.421)	1.18 (1.11 to 1.25)
Hungary	2020	Diseases of the ear and mastoid process	7,720	0.792	12,700 (12,100 to 13,300)	1.30 (1.24 to 1.36)	-4,970 (-5,590 to -4,380)	-0.509 (-0.573 to -0.450)	0.61 (0.58 to 0.64)	4.9	4.40 (4.10 to 4.71)	0.502 (0.186 to 0.798)	1.11 (1.04 to 1.19)
Hungary	2021	Diseases of the ear and mastoid process	5,580	0.575	12,600 (11,800 to 13,500)	1.30 (1.22 to 1.39)	-7,050 (-7,920 to -6,220)	-0.726 (-0.816 to -0.641)	0.44 (0.41 to 0.47)	4.5	4.40 (4.10 to 4.71)	0.102 (-0.212 to 0.391)	1.02 (0.95 to 1.10)
Hungary	2020	Diseases of the circulatory system	212,000	21.7	291,000 (284,000 to 299,000)	29.9 (29.1 to 30.6)	-79,300 (-86,700 to -72,100)	-8.14 (-8.90 to -7.40)	0.73 (0.71 to 0.75)	14	12.56 (12.23 to 12.90)	1.44 (1.10 to 1.76)	1.11 (1.09 to 1.14)
Hungary	2021	Diseases of the circulatory system	167,000	17.2	284,000 (274,000 to 294,000)	29.2 (28.2 to 30.3)	-117,000 (-128,000 to -107,000)	-12.0 (-13.1 to -11.0)	0.59 (0.57 to 0.61)	11.1	12.66 (12.31 to 13.01)	-1.56 (-1.92 to -1.21)	0.88 (0.85 to 0.90)
Hungary	2020	Diseases of the respiratory system	79,700	8.17	139,000 (129,000 to 151,000)	14.3 (13.2 to 15.5)	-59,700 (-71,100 to -49,100)	-6.12 (-7.29 to -5.04)	0.57 (0.53 to 0.62)	10	8.80 (8.40 to 9.22)	1.20 (0.781 to 1.60)	1.14 (1.08 to 1.19)
Hungary	2021	Diseases of the respiratory system	58,000	5.98	139,000 (124,000 to 155,000)	14.3 (12.8 to 16.0)	-80,700 (-97,500 to -66,200)	-8.31 (-10.0 to -6.81)	0.42 (0.37 to 0.47)	8.1	8.80 (8.24 to 9.40)	-0.700 (-1.30 to -0.128)	0.92 (0.86 to 0.98)
Hungary	2020	Diseases of the digestive system	96,600	9.91	133,000 (130,000 to 136,000)	13.7 (13.4 to 13.9)	-36,600 (-39,300 to -33,900)	-3.75 (-4.03 to -3.48)	0.73 (0.71 to 0.74)	5.8	5.47 (5.29 to 5.66)	0.328 (0.141 to 0.512)	1.06 (1.02 to 1.10)
Hungary	2021	Diseases of the digestive system	76,200	7.85	132,000 (128,000 to 136,000)	13.6 (13.2 to 14.0)	-55,400 (-59,300 to -51,700)	-5.71 (-6.11 to -5.32)	0.58 (0.56 to 0.60)	5.7	5.35 (5.10 to 5.61)	0.354 (0.092 to 0.601)	1.07 (1.02 to 1.12)
Hungary	2020	Diseases of the skin and	15,800	1.62	25,100 (23,800 to 26,400)	2.57 (2.44 to 2.71)	-9,250 (-10,500 to -8,040)	-0.949 (-1.08 to -0.824)	0.63 (0.60 to 0.66)	6.3	6.20 (5.91 to 6.50)	0.100 (-0.199 to 0.392)	1.02 (0.97 to 1.07)

Location	Year	Condition	Observed hospitalizations	Observed hospitalizations per 1,000 population	Expected hospitalizations	Expected hospitalizations per 1,000 population	Difference in hospitalizations	Difference in rate	Hospitalizations observed-to-expected ratio	Observed length of stay	Expected length of stay	Difference in length of stay	Length of stay observed-to-expected ratio
		subcutaneous tissue											
Hungary	2021	Diseases of the skin and subcutaneous tissue	11,100	1.14	24,500 (22,800 to 26,400)	2.53 (2.35 to 2.72)	-13,400 (-15,300 to -11,700)	-1.38 (-1.58 to -1.21)	0.45 (0.42 to 0.49)	6.1	6.20 (5.89 to 6.52)	-0.100 (-0.420 to 0.207)	0.98 (0.94 to 1.04)
Hungary	2020	Diseases of musculoskeletal system and connective tissue	99,600	10.2	182,000 (175,000 to 190,000)	18.7 (17.9 to 19.5)	-82,400 (-90,200 to -75,000)	-8.45 (-9.25 to -7.69)	0.55 (0.52 to 0.57)	11.4	12.31 (12.17 to 12.46)	-0.910 (-1.06 to -0.765)	0.93 (0.92 to 0.94)
Hungary	2021	Diseases of musculoskeletal system and connective tissue	58,200	5.99	179,000 (163,000 to 197,000)	18.4 (16.8 to 20.2)	-121,000 (-138,000 to -105,000)	-12.4 (-14.2 to -10.8)	0.33 (0.30 to 0.36)	10.1	12.31 (12.17 to 12.46)	-2.21 (-2.36 to -2.06)	0.82 (0.81 to 0.83)
Hungary	2020	Diseases of the genitourinary system	83,800	8.6	119,000 (114,000 to 125,000)	12.3 (11.7 to 12.8)	-35,600 (-41,100 to -30,500)	-3.66 (-4.22 to -3.12)	0.70 (0.67 to 0.73)	7.3	7.12 (6.83 to 7.43)	0.179 (-0.125 to 0.477)	1.03 (0.98 to 1.07)
Hungary	2021	Diseases of the genitourinary system	64,600	6.66	120,000 (113,000 to 126,000)	12.3 (11.7 to 13.0)	-55,000 (-61,800 to -48,500)	-5.66 (-6.37 to -5.00)	0.54 (0.51 to 0.57)	6.7	7.24 (6.83 to 7.69)	-0.544 (-0.987 to -0.124)	0.92 (0.87 to 0.98)
Hungary	2020	Pregnancy, childbirth and the puerperium	116,000	11.9	121,000 (116,000 to 126,000)	12.4 (11.9 to 12.9)	-5,050 (-10,000 to -205)	-0.518 (-1.03 to -0.021)	0.96 (0.92 to 1.00)	4.3	5.03 (4.90 to 5.16)	-0.730 (-0.866 to -0.598)	0.85 (0.83 to 0.88)
Hungary	2021	Pregnancy, childbirth and the puerperium	103,000	10.6	120,000 (113,000 to 127,000)	12.4 (11.7 to 13.1)	-17,600 (-24,800 to -10,800)	-1.81 (-2.55 to -1.11)	0.85 (0.81 to 0.90)	4.1	5.03 (4.90 to 5.16)	-0.930 (-1.07 to -0.800)	0.82 (0.79 to 0.84)
Hungary	2020	Certain conditions originating in the perinatal period	24,000	2.46	25,700 (24,300 to 27,100)	2.63 (2.49 to 2.78)	-1,730 (-3,200 to -367)	-0.178 (-0.328 to -0.038)	0.93 (0.88 to 0.98)	8.3	8.50 (8.01 to 9.02)	-0.200 (-0.726 to 0.289)	0.98 (0.92 to 1.04)
Hungary	2021	Certain conditions originating in the perinatal period	22,000	2.26	25,600 (24,200 to 27,000)	2.63 (2.49 to 2.78)	-3,630 (-5,070 to -2,270)	-0.374 (-0.522 to -0.234)	0.86 (0.81 to 0.91)	8	8.50 (7.82 to 9.24)	-0.500 (-1.23 to 0.180)	0.94 (0.87 to 1.02)
Hungary	2020	Congenital malformations, deformations and chromosomal abnormalities	5,410	0.555	7,860 (7,440 to 8,310)	0.807 (0.763 to 0.853)	-2,460 (-2,900 to -2,040)	-0.252 (-0.297 to -0.209)	0.69 (0.65 to 0.73)	4.7	4.80 (4.51 to 5.11)	-0.100 (-0.417 to 0.192)	0.98 (0.92 to 1.04)
Hungary	2021	Congenital malformations, deformations and chromosomal abnormalities	4,960	0.511	7,830 (7,240 to 8,470)	0.807 (0.746 to 0.872)	-2,870 (-3,500 to -2,270)	-0.296 (-0.361 to -0.234)	0.63 (0.59 to 0.69)	3.9	4.80 (4.39 to 5.25)	-0.900 (-1.35 to -0.489)	0.81 (0.74 to 0.89)
Hungary	2020	Symptoms, signs and abnormal clinical and laboratory findings, n.e.c.	50,000	5.12	56,600 (52,700 to 60,800)	5.81 (5.40 to 6.24)	-6,650 (-10,800 to -2,780)	-0.682 (-1.10 to -0.285)	0.88 (0.82 to 0.95)	11.6	8.10 (6.22 to 10.55)	3.50 (1.05 to 5.38)	1.43 (1.10 to 1.86)
Hungary	2021	Symptoms, signs and abnormal clinical and laboratory findings, n.e.c.	38,800	4	59,200 (53,500 to 65,600)	6.10 (5.51 to 6.75)	-20,400 (-26,800 to -14,700)	-2.10 (-2.76 to -1.52)	0.66 (0.59 to 0.73)	9.8	8.10 (5.57 to 11.77)	1.70 (-1.98 to 4.27)	1.21 (0.83 to 1.77)
Hungary	2020	Injury, poisoning and other consequences of external causes	106,000	10.9	133,000 (131,000 to 136,000)	13.7 (13.4 to 13.9)	-26,900 (-29,600 to -24,200)	-2.76 (-3.04 to -2.48)	0.80 (0.78 to 0.81)	8.4	8.50 (8.17 to 8.85)	-0.100 (-0.451 to 0.238)	0.99 (0.95 to 1.03)

Location	Year	Condition	Observed hospitalizations	Observed hospitalizations per 1,000 population	Expected hospitalizations	Expected hospitalizations per 1,000 population	Difference in hospitalizations	Difference in rate	Hospitalizations observed-to-expected ratio	Observed length of stay	Expected length of stay	Difference in length of stay	Length of stay observed-to-expected ratio
Hungary	2021	Injury, poisoning and other consequences of external causes	89,100	9.18	133,000 (130,000 to 135,000)	13.7 (13.4 to 13.9)	-43,600 (-46,300 to -41,000)	-4.49 (-4.77 to -4.22)	0.67 (0.66 to 0.68)	6.7	8.50 (8.03 to 9.00)	-1.80 (-2.29 to -1.34)	0.79 (0.74 to 0.83)
Hungary	2020	Factors influencing health status and contact with health services	69,900	7.17	66,500 (64,000 to 69,100)	6.82 (6.56 to 7.09)	3,410 (830 to 5,900)	0.350 (0.085 to 0.605)	1.05 (1.01 to 1.09)	3.2	3.56 (3.46 to 3.66)	-0.358 (-0.456 to -0.264)	0.90 (0.88 to 0.92)
Hungary	2021	Factors influencing health status and contact with health services	62,400	6.43	66,200 (63,700 to 68,800)	6.82 (6.56 to 7.09)	-3,810 (-6,410 to -1,320)	-0.392 (-0.660 to -0.136)	0.94 (0.91 to 0.98)	3.1	3.52 (3.38 to 3.65)	-0.417 (-0.556 to -0.282)	0.88 (0.85 to 0.92)
Ireland	2020	All causes	570,000	114	665,000 (639,000 to 692,000)	133 (128 to 139)	-95,100 (-122,000 to -68,400)	-19.1 (-24.5 to -13.7)	0.86 (0.82 to 0.89)				
Ireland	2021	All causes	600,000	119	671,000 (645,000 to 698,000)	133 (128 to 139)	-70,500 (-98,000 to -44,700)	-14.0 (-19.5 to -8.89)	0.89 (0.86 to 0.93)				
Ireland	2020	Infectious and parasitic diseases	13,500	2.7	20,600 (18,900 to 22,300)	4.12 (3.80 to 4.48)	-7,100 (-8,880 to -5,470)	-1.42 (-1.78 to -1.10)	0.65 (0.60 to 0.71)	7	5.16 (4.58 to 5.82)	1.84 (1.17 to 2.42)	1.36 (1.20 to 1.53)
Ireland	2021	Infectious and parasitic diseases	15,200	3.01	21,500 (19,300 to 24,000)	4.27 (3.83 to 4.78)	-6,360 (-8,850 to -4,070)	-1.26 (-1.76 to -0.808)	0.70 (0.63 to 0.79)	6.7	5.16 (4.58 to 5.82)	1.54 (0.883 to 2.12)	1.30 (1.15 to 1.46)
Ireland	2020	Neoplasms	30,000	6.02	35,200 (33,400 to 37,000)	7.06 (6.70 to 7.43)	-5,180 (-7,010 to -3,370)	-1.04 (-1.41 to -0.676)	0.85 (0.81 to 0.90)	9.9	10.00 (9.46 to 10.57)	-0.100 (-0.678 to 0.428)	0.99 (0.94 to 1.05)
Ireland	2021	Neoplasms	31,600	6.28	35,500 (33,000 to 38,200)	7.06 (6.56 to 7.59)	-3,930 (-6,680 to -1,420)	-0.781 (-1.33 to -0.282)	0.89 (0.83 to 0.96)	9.6	10.00 (9.24 to 10.82)	-0.400 (-1.22 to 0.374)	0.96 (0.89 to 1.04)
Ireland	2020	Diseases of the blood and bloodforming organs	6,850	1.37	7,810 (6,830 to 8,930)	1.57 (1.37 to 1.79)	-962 (-2,090 to -9)	-0.193 (-0.419 to -0.002)	0.88 (0.77 to 1.00)	5.3	5.20 (4.80 to 5.64)	0.100 (-0.339 to 0.495)	1.02 (0.94 to 1.10)
Ireland	2021	Diseases of the blood and bloodforming organs	7,360	1.46	7,880 (6,520 to 9,530)	1.57 (1.30 to 1.89)	-529 (-2,170 to 823)	-0.105 (-0.431 to 0.163)	0.93 (0.77 to 1.13)	5	5.20 (4.64 to 5.83)	-0.200 (-0.821 to 0.361)	0.96 (0.86 to 1.08)
Ireland	2020	Endocrine, nutritional and metabolic diseases	11,100	2.24	12,100 (11,100 to 13,100)	2.42 (2.23 to 2.63)	-931 (-1,960 to 3)	-0.187 (-0.392 to 0.001)	0.92 (0.85 to 1.00)	7.9	8.21 (7.71 to 8.74)	-0.312 (-0.832 to 0.185)	0.96 (0.90 to 1.02)
Ireland	2021	Endocrine, nutritional and metabolic diseases	11,900	2.37	12,200 (11,200 to 13,200)	2.42 (2.23 to 2.63)	-252 (-1,280 to 711)	-0.050 (-0.255 to 0.141)	0.98 (0.90 to 1.06)	8	8.54 (7.42 to 9.82)	-0.536 (-1.85 to 0.581)	0.94 (0.81 to 1.08)
Ireland	2020	Mental and behavioural disorders	8,320	1.67	7,470 (6,570 to 8,490)	1.50 (1.32 to 1.70)	845 (-180 to 1,740)	0.170 (-0.036 to 0.348)	1.11 (0.98 to 1.26)	11.2	11.44 (9.90 to 13.23)	-0.242 (-1.99 to 1.29)	0.98 (0.85 to 1.13)
Ireland	2021	Mental and behavioural disorders	9,200	1.83	7,540 (6,290 to 9,040)	1.50 (1.25 to 1.80)	1,660 (161 to 2,890)	0.329 (0.032 to 0.575)	1.22 (1.02 to 1.46)	11.6	11.44 (9.90 to 13.23)	0.158 (-1.62 to 1.66)	1.01 (0.88 to 1.17)
Ireland	2020	Diseases of the nervous system	17,400	3.49	20,300 (18,700 to 21,900)	4.06 (3.76 to 4.40)	-2,870 (-4,540 to -1,340)	-0.576 (-0.911 to -0.269)	0.86 (0.79 to 0.93)	7	6.20 (5.42 to 7.09)	0.800 (-0.097 to 1.58)	1.13 (0.99 to 1.29)
Ireland	2021	Diseases of the nervous system	18,300	3.63	20,500 (18,300 to 22,900)	4.06 (3.63 to 4.55)	-2,180 (-4,620 to 15)	-0.434 (-0.918 to 0.003)	0.89 (0.80 to 1.00)	7	6.20 (5.13 to 7.49)	0.800 (-0.465 to 1.85)	1.13 (0.94 to 1.36)
Ireland	2020	Diseases of the eye and adnexa	4,100	0.821	4,760 (4,320 to 5,250)	0.955 (0.867 to 1.05)	-667 (-1,160 to -224)	-0.134 (-0.233 to -0.045)	0.86 (0.78 to 0.95)	3	2.80 (2.54 to 3.08)	0.200 (-0.082 to 0.460)	1.07 (0.97 to 1.18)

Location	Year	Condition	Observed hospitalizations	Observed hospitalizations per 1,000 population	Expected hospitalizations	Expected hospitalizations per 1,000 population	Difference in hospitalizations	Difference in rate	Hospitalizations observed-to-expected ratio	Observed length of stay	Expected length of stay	Difference in length of stay	Length of stay observed-to-expected ratio
Ireland	2021	Diseases of the eye and adnexa	4,460	0.887	4,810 (4,190 to 5,520)	0.955 (0.833 to 1.10)	-343 (-1,050 to 275)	-0.068 (-0.209 to 0.055)	0.93 (0.81 to 1.07)	2.9	2.80 (2.45 to 3.21)	0.100 (-0.305 to 0.455)	1.04 (0.90 to 1.19)
Ireland	2020	Diseases of the ear and mastoid process	2,510	0.503	3,160 (2,810 to 3,550)	0.633 (0.563 to 0.712)	-651 (-1,030 to -297)	-0.131 (-0.207 to -0.060)	0.79 (0.71 to 0.89)	2.4	2.30 (2.02 to 2.61)	0.100 (-0.209 to 0.380)	1.04 (0.92 to 1.19)
Ireland	2021	Diseases of the ear and mastoid process	2,730	0.542	3,190 (2,700 to 3,760)	0.633 (0.536 to 0.748)	-459 (-1,050 to 35)	-0.091 (-0.209 to 0.007)	0.86 (0.72 to 1.01)	2.5	2.30 (1.92 to 2.75)	0.200 (-0.257 to 0.573)	1.09 (0.91 to 1.30)
Ireland	2020	Diseases of the circulatory system	52,400	10.5	56,800 (54,500 to 59,200)	11.4 (10.9 to 11.9)	-4,370 (-6,820 to -2,050)	-0.877 (-1.37 to -0.412)	0.92 (0.88 to 0.96)	8.2	7.90 (7.33 to 8.52)	0.300 (-0.323 to 0.868)	1.04 (0.96 to 1.12)
Ireland	2021	Diseases of the circulatory system	54,800	10.9	57,300 (55,000 to 59,800)	11.4 (10.9 to 11.9)	-2,520 (-4,970 to -176)	-0.500 (-0.987 to -0.035)	0.96 (0.92 to 1.00)	7.8	7.90 (7.10 to 8.79)	-0.100 (-0.964 to 0.708)	0.99 (0.89 to 1.10)
Ireland	2020	Diseases of the respiratory system	52,700	10.6	78,700 (69,400 to 89,200)	15.8 (13.9 to 17.9)	-26,000 (-36,400 to -16,600)	-5.21 (-7.31 to -3.33)	0.67 (0.59 to 0.76)	7.8	6.61 (6.07 to 7.21)	1.19 (0.591 to 1.73)	1.18 (1.08 to 1.28)
Ireland	2021	Diseases of the respiratory system	55,400	11	79,400 (66,500 to 94,900)	15.8 (13.2 to 18.9)	-24,100 (-39,400 to -11,200)	-4.78 (-7.83 to -2.22)	0.70 (0.58 to 0.83)	8	6.61 (6.07 to 7.21)	1.39 (0.792 to 1.94)	1.21 (1.11 to 1.32)
Ireland	2020	Diseases of the digestive system	46,400	9.31	52,400 (50,700 to 54,200)	10.5 (10.2 to 10.9)	-6,040 (-7,870 to -4,330)	-1.21 (-1.58 to -0.869)	0.88 (0.85 to 0.91)	6	5.78 (5.39 to 6.19)	0.223 (-0.189 to 0.608)	1.04 (0.97 to 1.11)
Ireland	2021	Diseases of the digestive system	49,000	9.73	52,900 (51,100 to 54,900)	10.5 (10.1 to 10.9)	-3,990 (-5,920 to -2,090)	-0.792 (-1.18 to -0.416)	0.92 (0.89 to 0.96)	6.1	5.78 (5.39 to 6.19)	0.323 (-0.094 to 0.708)	1.06 (0.98 to 1.13)
Ireland	2020	Diseases of the skin and subcutaneous tissue	9,490	1.9	12,200 (11,200 to 13,300)	2.45 (2.25 to 2.67)	-2,720 (-3,830 to -1,720)	-0.545 (-0.767 to -0.345)	0.78 (0.71 to 0.85)	6.3	6.16 (5.78 to 6.56)	0.143 (-0.255 to 0.515)	1.02 (0.96 to 1.09)
Ireland	2021	Diseases of the skin and subcutaneous tissue	9,510	1.89	12,300 (10,900 to 13,900)	2.45 (2.17 to 2.76)	-2,810 (-4,410 to -1,420)	-0.558 (-0.875 to -0.283)	0.77 (0.68 to 0.87)	6.6	6.16 (5.78 to 6.56)	0.443 (0.039 to 0.821)	1.07 (1.01 to 1.14)
Ireland	2020	Diseases of musculoskeletal system and connective tissue	24,500	4.91	26,300 (23,700 to 29,200)	5.28 (4.75 to 5.86)	-1,850 (-4,740 to 769)	-0.370 (-0.951 to 0.154)	0.93 (0.84 to 1.03)	5.5	5.43 (4.80 to 6.15)	0.066 (-0.657 to 0.705)	1.01 (0.89 to 1.15)
Ireland	2021	Diseases of musculoskeletal system and connective tissue	25,700	5.1	25,700 (20,400 to 32,500)	5.11 (4.04 to 6.45)	-15 (-6,860 to 5,450)	-0.003 (-1.36 to 1.08)	1.00 (0.79 to 1.27)	5.2	5.46 (4.66 to 6.40)	-0.262 (-1.20 to 0.531)	0.95 (0.81 to 1.11)
Ireland	2020	Diseases of the genitourinary system	32,600	6.55	37,800 (35,600 to 40,100)	7.58 (7.14 to 8.05)	-5,160 (-7,490 to -2,990)	-1.03 (-1.50 to -0.599)	0.86 (0.81 to 0.92)	6.1	6.20 (5.96 to 6.45)	-0.100 (-0.347 to 0.134)	0.98 (0.95 to 1.02)
Ireland	2021	Diseases of the genitourinary system	34,000	6.75	38,200 (35,100 to 41,500)	7.58 (6.97 to 8.25)	-4,210 (-7,580 to -1,100)	-0.836 (-1.51 to -0.219)	0.89 (0.82 to 0.97)	6.1	6.20 (5.87 to 6.55)	-0.100 (-0.455 to 0.232)	0.98 (0.93 to 1.04)
Ireland	2020	Pregnancy, childbirth and the puerperium	91,700	18.4	97,900 (94,400 to 101,000)	19.6 (18.9 to 20.4)	-6,210 (-9,820 to -2,750)	-1.25 (-1.97 to -0.551)	0.94 (0.90 to 0.97)	2.6	2.80 (2.73 to 2.87)	-0.200 (-0.268 to -0.135)	0.93 (0.91 to 0.95)
Ireland	2021	Pregnancy, childbirth and the puerperium	97,700	19.4	95,800 (91,100 to 101,000)	19.0 (18.1 to 20.0)	1,820 (-3,250 to 6,490)	0.361 (-0.646 to 1.29)	1.02 (0.97 to 1.07)	2.6	2.80 (2.71 to 2.90)	-0.200 (-0.296 to -0.108)	0.93 (0.90 to 0.96)
Ireland	2020	Certain conditions originating in the perinatal period	9,590	1.92	9,530 (9,050 to 10,000)	1.91 (1.82 to 2.01)	62 (-436 to 557)	0.012 (-0.087 to 0.112)	1.01 (0.96 to 1.06)	8.1	8.20 (7.60 to 8.85)	-0.100 (-0.755 to 0.496)	0.99 (0.91 to 1.07)
Ireland	2021	Certain conditions originating in the perinatal period	9,610	1.91	9,620 (8,940 to 10,400)	1.91 (1.78 to 2.06)	-17 (-748 to 664)	-0.003 (-0.149 to 0.132)	1.00 (0.93 to 1.07)	8.2	8.20 (7.36 to 9.13)	0.000 (-0.936 to 0.838)	1.00 (0.90 to 1.11)

Location	Year	Condition	Observed hospitalizations	Observed hospitalizations per 1,000 population	Expected hospitalizations	Expected hospitalizations per 1,000 population	Difference in hospitalizations	Difference in rate	Hospitalizations observed-to-expected ratio	Observed length of stay	Expected length of stay	Difference in length of stay	Length of stay observed-to-expected ratio
Ireland	2020	Congenital malformations, deformations and chromosomal abnormalities	3,190	0.64	3,540 (3,280 to 3,810)	0.709 (0.659 to 0.764)	-345 (-620 to -94)	-0.069 (-0.124 to -0.019)	0.90 (0.84 to 0.97)	7.9	6.93 (5.88 to 8.16)	0.972 (-0.249 to 2.01)	1.14 (0.97 to 1.34)
Ireland	2021	Congenital malformations, deformations and chromosomal abnormalities	3,410	0.677	3,430 (3,090 to 3,810)	0.682 (0.614 to 0.757)	-23 (-404 to 324)	-0.005 (-0.080 to 0.064)	0.99 (0.89 to 1.10)	7.1	6.93 (5.88 to 8.16)	0.172 (-1.06 to 1.23)	1.02 (0.87 to 1.21)
Ireland	2020	Symptoms, signs and abnormal clinical and laboratory findings, n.e.c.	84,300	16.9	85,400 (75,600 to 96,600)	17.1 (15.2 to 19.4)	-1,140 (-12,600 to 8,660)	-0.229 (-2.54 to 1.74)	0.99 (0.87 to 1.11)	3.1	3.00 (2.70 to 3.33)	0.100 (-0.227 to 0.399)	1.03 (0.93 to 1.15)
Ireland	2021	Symptoms, signs and abnormal clinical and laboratory findings, n.e.c.	88,400	17.6	85,000 (64,500 to 112,000)	16.9 (12.8 to 22.2)	3,450 (-23,500 to 23,600)	0.685 (-4.68 to 4.70)	1.04 (0.79 to 1.36)	3.1	3.00 (2.59 to 3.48)	0.100 (-0.385 to 0.508)	1.03 (0.89 to 1.20)
Ireland	2020	Injury, poisoning and other consequences of external causes	54,900	11	57,800 (54,900 to 60,900)	11.6 (11.0 to 12.2)	-2,910 (-5,910 to -27)	-0.583 (-1.19 to -0.005)	0.95 (0.90 to 1.00)	7	6.60 (6.16 to 7.07)	0.400 (-0.076 to 0.826)	1.06 (0.99 to 1.13)
Ireland	2021	Injury, poisoning and other consequences of external causes	58,400	11.6	58,400 (55,500 to 61,500)	11.6 (11.0 to 12.2)	49 (-2,950 to 2,900)	0.010 (-0.586 to 0.576)	1.00 (0.95 to 1.05)	7	6.60 (5.99 to 7.28)	0.400 (-0.280 to 1.01)	1.06 (0.96 to 1.17)
Ireland	2020	Factors influencing health status and contact with health services	14,000	2.8	22,100 (20,700 to 23,600)	4.43 (4.15 to 4.73)	-8,120 (-9,590 to -6,740)	-1.63 (-1.92 to -1.35)	0.63 (0.59 to 0.67)	6.3	11.20 (10.16 to 12.34)	-4.90 (-6.02 to -3.86)	0.56 (0.51 to 0.62)
Ireland	2021	Factors influencing health status and contact with health services	13,900	2.77	22,300 (20,900 to 23,800)	4.43 (4.15 to 4.73)	-8,360 (-9,870 to -6,960)	-1.66 (-1.96 to -1.38)	0.63 (0.59 to 0.67)	6.1	11.20 (9.76 to 12.85)	-5.10 (-6.73 to -3.64)	0.54 (0.48 to 0.63)
Israel	2020	All causes	1,230,000	133	1,380,000 (1,360,000 to 1,400,000)	150 (147 to 152)	-149,000 (-171,000 to -128,000)	-16.2 (-18.6 to -13.9)	0.89 (0.88 to 0.91)				
Israel	2021	All causes	1,330,000	142	1,390,000 (1,370,000 to 1,410,000)	148 (146 to 150)	-59,600 (-82,100 to -38,300)	-6.36 (-8.76 to -4.09)	0.96 (0.94 to 0.97)				
Israel	2020	Infectious and parasitic diseases	17,900	1.95	25,400 (23,000 to 27,900)	2.75 (2.50 to 3.03)	-7,440 (-10,000 to -5,130)	-0.807 (-1.09 to -0.556)	0.71 (0.64 to 0.78)	6.7	5.99 (5.52 to 6.49)	0.715 (0.210 to 1.18)	1.12 (1.03 to 1.21)
Israel	2021	Infectious and parasitic diseases	22,200	2.37	25,800 (23,400 to 28,400)	2.75 (2.50 to 3.03)	-3,560 (-6,240 to -1,230)	-0.380 (-0.666 to -0.131)	0.86 (0.78 to 0.95)	5.9	5.99 (5.52 to 6.49)	-0.085 (-0.585 to 0.377)	0.99 (0.91 to 1.07)
Israel	2020	Neoplasms	51,600	5.6	51,900 (49,700 to 54,300)	5.64 (5.39 to 5.89)	-345 (-2,700 to 1,900)	-0.037 (-0.293 to 0.206)	0.99 (0.95 to 1.04)	6.2	6.40 (6.02 to 6.80)	-0.200 (-0.608 to 0.178)	0.97 (0.91 to 1.03)
Israel	2021	Neoplasms	54,200	5.78	51,800 (48,600 to 55,100)	5.52 (5.19 to 5.88)	2,400 (-1,010 to 5,480)	0.256 (-0.108 to 0.585)	1.05 (0.98 to 1.11)	6.1	6.40 (5.87 to 6.98)	-0.300 (-0.876 to 0.219)	0.95 (0.87 to 1.04)

Location	Year	Condition	Observed hospitalizations	Observed hospitalizations per 1,000 population	Expected hospitalizations	Expected hospitalizations per 1,000 population	Difference in hospitalizations	Difference in rate	Hospitalizations observed-to-expected ratio	Observed length of stay	Expected length of stay	Difference in length of stay	Length of stay observed-to-expected ratio
Israel	2020	Diseases of the blood and bloodforming organs	15,100	1.64	16,700 (16,000 to 17,400)	1.81 (1.73 to 1.89)	-1,520 (-2,230 to -825)	-0.165 (-0.242 to -0.090)	0.91 (0.87 to 0.95)	5.3	5.28 (4.42 to 6.32)	0.018 (-1.01 to 0.887)	1.00 (0.84 to 1.20)
Israel	2021	Diseases of the blood and bloodforming organs	16,700	1.78	16,600 (15,600 to 17,600)	1.77 (1.67 to 1.88)	52 (-1,010 to 1,020)	0.006 (-0.108 to 0.109)	1.00 (0.94 to 1.07)	5.1	5.60 (4.63 to 6.78)	-0.503 (-1.65 to 0.492)	0.91 (0.76 to 1.11)
Israel	2020	Endocrine, nutritional and metabolic diseases	26,600	2.88	30,500 (27,200 to 34,100)	3.31 (2.95 to 3.70)	-3,890 (-7,460 to -647)	-0.422 (-0.810 to -0.070)	0.87 (0.78 to 0.98)	5.6	5.97 (4.81 to 7.40)	-0.368 (-1.83 to 0.773)	0.94 (0.75 to 1.16)
Israel	2021	Endocrine, nutritional and metabolic diseases	33,300	3.55	31,000 (26,400 to 36,300)	3.31 (2.82 to 3.88)	2,290 (-3,010 to 6,830)	0.244 (-0.321 to 0.729)	1.07 (0.92 to 1.26)	5.2	5.97 (4.81 to 7.40)	-0.768 (-2.15 to 0.409)	0.87 (0.71 to 1.09)
Israel	2020	Mental and behavioural disorders	27,800	3.02	31,700 (30,400 to 33,000)	3.44 (3.30 to 3.58)	-3,830 (-5,170 to -2,580)	-0.416 (-0.561 to -0.280)	0.88 (0.84 to 0.92)	45.7	45.90 (36.88 to 57.12)	-0.200 (-11.23 to 8.89)	1.00 (0.80 to 1.24)
Israel	2021	Mental and behavioural disorders	30,800	3.28	32,200 (30,900 to 33,500)	3.44 (3.30 to 3.58)	-1,420 (-2,760 to -153)	-0.152 (-0.294 to -0.016)	0.96 (0.92 to 1.00)	38.7	45.90 (33.69 to 62.54)	-7.20 (-23.86 to 4.89)	0.84 (0.62 to 1.14)
Israel	2020	Diseases of the nervous system	19,000	2.06	21,300 (20,400 to 22,200)	2.31 (2.21 to 2.41)	-2,300 (-3,250 to -1,370)	-0.250 (-0.352 to -0.149)	0.89 (0.85 to 0.93)	8.6	7.71 (6.48 to 9.18)	0.888 (-0.629 to 2.13)	1.12 (0.93 to 1.33)
Israel	2021	Diseases of the nervous system	21,300	2.28	21,700 (20,700 to 22,600)	2.31 (2.21 to 2.41)	-306 (-1,280 to 621)	-0.033 (-0.137 to 0.066)	0.99 (0.94 to 1.03)	7.9	7.71 (6.48 to 9.18)	0.188 (-1.27 to 1.42)	1.02 (0.86 to 1.22)
Israel	2020	Diseases of the eye and adnexa	17,000	1.85	17,500 (16,200 to 18,900)	1.90 (1.76 to 2.05)	-473 (-1,890 to 826)	-0.051 (-0.205 to 0.090)	0.97 (0.90 to 1.05)	2.8	3.07 (2.82 to 3.34)	-0.267 (-0.539 to -0.021)	0.91 (0.84 to 0.99)
Israel	2021	Diseases of the eye and adnexa	17,200	1.84	17,300 (15,500 to 19,300)	1.85 (1.66 to 2.06)	-76 (-2,070 to 1,700)	-0.008 (-0.221 to 0.182)	1.00 (0.89 to 1.11)	2.8	3.07 (2.82 to 3.34)	-0.267 (-0.541 to -0.019)	0.91 (0.84 to 0.99)
Israel	2020	Diseases of the ear and mastoid process	8,380	0.909	10,500 (10,100 to 10,900)	1.14 (1.10 to 1.19)	-2,140 (-2,550 to -1,730)	-0.232 (-0.277 to -0.188)	0.80 (0.77 to 0.83)	3.1	3.30 (2.95 to 3.70)	-0.200 (-0.594 to 0.156)	0.94 (0.84 to 1.05)
Israel	2021	Diseases of the ear and mastoid process	9,480	1.01	10,700 (10,300 to 11,100)	1.14 (1.10 to 1.19)	-1,210 (-1,640 to -806)	-0.129 (-0.176 to -0.086)	0.89 (0.85 to 0.92)	2.9	3.30 (2.81 to 3.87)	-0.400 (-0.980 to 0.092)	0.88 (0.75 to 1.03)
Israel	2020	Diseases of the circulatory system	112,000	12.2	123,000 (118,000 to 127,000)	13.3 (12.8 to 13.8)	-10,400 (-15,200 to -5,610)	-1.12 (-1.65 to -0.609)	0.92 (0.88 to 0.95)	6.1	6.50 (6.17 to 6.85)	-0.400 (-0.750 to -0.061)	0.94 (0.89 to 0.99)
Israel	2021	Diseases of the circulatory system	119,000	12.7	125,000 (118,000 to 132,000)	13.3 (12.6 to 14.0)	-6,040 (-13,000 to 695)	-0.645 (-1.39 to 0.074)	0.95 (0.90 to 1.01)	6.2	6.50 (6.04 to 7.00)	-0.300 (-0.816 to 0.171)	0.95 (0.88 to 1.03)
Israel	2020	Diseases of the respiratory system	69,900	7.59	101,000 (95,400 to 106,000)	10.9 (10.4 to 11.6)	-30,900 (-36,600 to -25,600)	-3.35 (-3.97 to -2.77)	0.69 (0.66 to 0.73)	6.1	5.36 (4.97 to 5.78)	0.744 (0.330 to 1.13)	1.14 (1.06 to 1.23)
Israel	2021	Diseases of the respiratory system	79,900	8.53	103,000 (94,900 to 111,000)	10.9 (10.1 to 11.8)	-22,600 (-30,700 to -14,900)	-2.41 (-3.28 to -1.59)	0.78 (0.72 to 0.84)	5.4	5.36 (4.97 to 5.78)	0.044 (-0.374 to 0.423)	1.01 (0.94 to 1.08)
Israel	2020	Diseases of the digestive system	94,900	10.3	107,000 (104,000 to 111,000)	11.6 (11.2 to 12.0)	-12,000 (-15,500 to -8,580)	-1.31 (-1.69 to -0.931)	0.89 (0.86 to 0.92)	4.1	3.94 (3.80 to 4.07)	0.165 (0.029 to 0.295)	1.04 (1.01 to 1.08)
Israel	2021	Diseases of the digestive system	105,000	11.2	109,000 (105,000 to 112,000)	11.6 (11.2 to 12.0)	-3,830 (-7,500 to -361)	-0.409 (-0.800 to -0.039)	0.96 (0.93 to 1.00)	4	3.98 (3.84 to 4.12)	0.023 (-0.124 to 0.163)	1.01 (0.97 to 1.04)
Israel	2020	Diseases of the skin and subcutaneous tissue	19,300	2.09	24,000 (23,100 to 25,000)	2.61 (2.51 to 2.71)	-4,760 (-5,700 to -3,840)	-0.516 (-0.619 to -0.416)	0.80 (0.77 to 0.83)	7.7	7.39 (6.47 to 8.45)	0.305 (-0.723 to 1.23)	1.04 (0.91 to 1.19)

Location	Year	Condition	Observed hospitalizations	Observed hospitalizations per 1,000 population	Expected hospitalizations	Expected hospitalizations per 1,000 population	Difference in hospitalizations	Difference in rate	Hospitalizations observed-to-expected ratio	Observed length of stay	Expected length of stay	Difference in length of stay	Length of stay observed-to-expected ratio
Israel	2021	Diseases of the skin and subcutaneous tissue	21,200	2.26	24,400 (23,100 to 25,800)	2.61 (2.47 to 2.76)	-3,260 (-4,640 to -1,980)	-0.348 (-0.495 to -0.211)	0.87 (0.82 to 0.91)	6.9	7.39 (6.47 to 8.45)	-0.495 (-1.53 to 0.431)	0.93 (0.82 to 1.07)
Israel	2020	Diseases of musculoskeletal system and connective tissue	39,500	4.29	45,600 (42,600 to 48,700)	4.95 (4.63 to 5.28)	-6,080 (-9,190 to -3,100)	-0.660 (-0.997 to -0.336)	0.87 (0.81 to 0.93)	4.7	5.45 (5.11 to 5.81)	-0.747 (-1.11 to -0.406)	0.86 (0.81 to 0.92)
Israel	2021	Diseases of musculoskeletal system and connective tissue	43,100	4.6	46,300 (42,200 to 50,900)	4.95 (4.50 to 5.43)	-3,240 (-7,830 to 962)	-0.346 (-0.836 to 0.103)	0.93 (0.85 to 1.02)	4.8	5.45 (5.11 to 5.81)	-0.647 (-1.01 to -0.301)	0.88 (0.83 to 0.94)
Israel	2020	Diseases of the genitourinary system	72,000	7.81	79,400 (77,200 to 81,700)	8.62 (8.38 to 8.87)	-7,450 (-9,730 to -5,260)	-0.808 (-1.06 to -0.571)	0.91 (0.88 to 0.93)	3.9	4.16 (3.88 to 4.47)	-0.265 (-0.569 to 0.023)	0.94 (0.87 to 1.01)
Israel	2021	Diseases of the genitourinary system	76,100	8.12	80,800 (78,500 to 83,100)	8.62 (8.38 to 8.87)	-4,640 (-7,000 to -2,370)	-0.495 (-0.747 to -0.253)	0.94 (0.92 to 0.97)	3.9	4.21 (3.87 to 4.58)	-0.306 (-0.680 to 0.032)	0.93 (0.85 to 1.01)
Israel	2020	Pregnancy, childbirth and the puerperium	188,000	20.4	194,000 (190,000 to 198,000)	21.0 (20.6 to 21.5)	-5,940 (-10,100 to -1,880)	-0.645 (-1.10 to -0.204)	0.97 (0.95 to 0.99)	2.9	3.20 (3.20 to 3.20)	-0.300 (-0.300 to -0.300)	0.91 (0.91 to 0.91)
Israel	2021	Pregnancy, childbirth and the puerperium	189,000	20.2	193,000 (188,000 to 199,000)	20.6 (20.0 to 21.3)	-3,880 (-9,860 to 1,900)	-0.414 (-1.05 to 0.202)	0.98 (0.95 to 1.01)	3	3.20 (3.20 to 3.20)	-0.200 (-0.200 to -0.200)	0.94 (0.94 to 0.94)
Israel	2020	Certain conditions originating in the perinatal period	13,300	1.44	13,800 (13,000 to 14,700)	1.50 (1.42 to 1.59)	-550 (-1,390 to 242)	-0.060 (-0.151 to 0.026)	0.96 (0.91 to 1.02)	5.3	5.41 (4.78 to 6.13)	-0.111 (-0.824 to 0.522)	0.98 (0.87 to 1.11)
Israel	2021	Certain conditions originating in the perinatal period	14,400	1.53	14,100 (13,300 to 14,900)	1.50 (1.42 to 1.59)	291 (-548 to 1,090)	0.031 (-0.058 to 0.116)	1.02 (0.96 to 1.08)	5.3	5.14 (4.31 to 6.13)	0.163 (-0.865 to 1.00)	1.03 (0.86 to 1.23)
Israel	2020	Congenital malformations, deformations and chromosomal abnormalities	10,700	1.17	11,700 (10,400 to 13,100)	1.27 (1.13 to 1.42)	-926 (-2,330 to 356)	-0.100 (-0.253 to 0.039)	0.92 (0.82 to 1.03)	5.2	5.20 (4.51 to 6.00)	0.000 (-0.808 to 0.697)	1.00 (0.87 to 1.15)
Israel	2021	Congenital malformations, deformations and chromosomal abnormalities	11,500	1.22	11,900 (10,100 to 13,900)	1.27 (1.08 to 1.49)	-408 (-2,560 to 1,340)	-0.044 (-0.273 to 0.143)	0.97 (0.82 to 1.13)	5.5	5.20 (4.25 to 6.37)	0.300 (-0.855 to 1.26)	1.06 (0.87 to 1.30)
Israel	2020	Symptoms, signs and abnormal clinical and laboratory findings, n.e.c.	127,000	13.8	160,000 (149,000 to 172,000)	17.3 (16.1 to 18.7)	-32,900 (-45,200 to -21,800)	-3.57 (-4.90 to -2.36)	0.79 (0.74 to 0.85)	7	6.22 (5.44 to 7.10)	0.783 (-0.091 to 1.55)	1.13 (0.99 to 1.28)
Israel	2021	Symptoms, signs and abnormal clinical and laboratory findings, n.e.c.	146,000	15.6	158,000 (142,000 to 175,000)	16.8 (15.2 to 18.7)	-11,400 (-28,800 to 4,140)	-1.22 (-3.07 to 0.442)	0.93 (0.84 to 1.03)	6.5	6.22 (5.44 to 7.10)	0.283 (-0.617 to 1.05)	1.05 (0.91 to 1.19)
Israel	2020	Injury, poisoning and other consequences of external causes	85,300	9.25	93,600 (90,700 to 96,500)	10.2 (9.85 to 10.5)	-8,320 (-11,200 to -5,450)	-0.903 (-1.22 to -0.591)	0.91 (0.88 to 0.94)	6.2	6.30 (5.95 to 6.67)	-0.102 (-0.472 to 0.247)	0.98 (0.93 to 1.04)
Israel	2021	Injury, poisoning and other consequences of external causes	91,100	9.72	94,200 (90,100 to 98,400)	10.0 (9.61 to 10.5)	-3,090 (-7,270 to 919)	-0.330 (-0.776 to 0.098)	0.97 (0.93 to 1.01)	6.2	6.20 (5.46 to 7.05)	-0.005 (-0.838 to 0.742)	1.00 (0.88 to 1.14)

Location	Year	Condition	Observed hospitalizations	Observed hospitalizations per 1,000 population	Expected hospitalizations	Expected hospitalizations per 1,000 population	Difference in hospitalizations	Difference in rate	Hospitalizations observed-to-expected ratio	Observed length of stay	Expected length of stay	Difference in length of stay	Length of stay observed-to-expected ratio
Israel	2020	Factors influencing health status and contact with health services	215,000	23.3	225,000 (218,000 to 233,000)	24.4 (23.6 to 25.3)	-10,600 (-18,600 to -2,950)	-1.15 (-2.02 to -0.320)	0.95 (0.92 to 0.99)	5.7	5.78 (5.62 to 5.94)	-0.078 (-0.243 to 0.082)	0.99 (0.96 to 1.01)
Israel	2021	Factors influencing health status and contact with health services	225,000	24	226,000 (216,000 to 238,000)	24.2 (23.0 to 25.4)	-1,170 (-12,400 to 9,720)	-0.125 (-1.33 to 1.04)	0.99 (0.95 to 1.05)	5.4	5.95 (5.78 to 6.12)	-0.551 (-0.718 to -0.385)	0.91 (0.88 to 0.93)
Italy	2020	All causes	5,320,000	89.5	6,650,000 (6,500,000 to 6,800,000)	112 (109 to 114)	-1,320,000 (-1,480,000 to -1,180,000)	-22.3 (-24.9 to -19.8)	0.80 (0.78 to 0.82)				
Italy	2021	All causes	5,600,000	94.6	6,550,000 (6,220,000 to 6,890,000)	111 (105 to 116)	-951,000 (-1,290,000 to -623,000)	-16.1 (-21.8 to -10.5)	0.85 (0.81 to 0.90)				
Italy	2020	Infectious and parasitic diseases	103,000	1.74	128,000 (120,000 to 137,000)	2.15 (2.01 to 2.30)	-24,800 (-33,700 to -16,700)	-0.418 (-0.567 to -0.281)	0.81 (0.75 to 0.86)	13	12.39 (11.85 to 12.95)	0.613 (0.054 to 1.14)	1.05 (1.00 to 1.10)
Italy	2021	Infectious and parasitic diseases	112,000	1.89	127,000 (119,000 to 136,000)	2.15 (2.01 to 2.30)	-15,400 (-24,100 to -7,220)	-0.261 (-0.407 to -0.122)	0.88 (0.82 to 0.94)	13	12.68 (11.91 to 13.50)	0.320 (-0.506 to 1.09)	1.03 (0.96 to 1.09)
Italy	2020	Neoplasms	573,000	9.63	654,000 (639,000 to 668,000)	11.0 (10.8 to 11.2)	-80,900 (-95,400 to -67,100)	-1.36 (-1.61 to -1.13)	0.88 (0.86 to 0.90)	8.1	8.11 (7.98 to 8.23)	-0.006 (-0.133 to 0.119)	1.00 (0.98 to 1.01)
Italy	2021	Neoplasms	607,000	10.3	645,000 (614,000 to 677,000)	10.9 (10.4 to 11.4)	-37,800 (-69,900 to -7,240)	-0.640 (-1.18 to -0.122)	0.94 (0.90 to 0.99)	7.7	8.01 (7.84 to 8.19)	-0.312 (-0.495 to -0.143)	0.96 (0.94 to 0.98)
Italy	2020	Diseases of the blood and bloodforming organs	44,600	0.751	55,300 (53,000 to 57,600)	0.930 (0.892 to 0.968)	-10,600 (-12,900 to -8,400)	-0.179 (-0.217 to -0.141)	0.81 (0.78 to 0.84)	8.9	8.60 (8.47 to 8.73)	0.300 (0.163 to 0.428)	1.03 (1.02 to 1.05)
Italy	2021	Diseases of the blood and bloodforming organs	45,000	0.761	53,700 (50,700 to 56,800)	0.907 (0.857 to 0.961)	-8,680 (-11,900 to -5,660)	-0.147 (-0.201 to -0.096)	0.84 (0.79 to 0.89)	9.1	8.60 (8.41 to 8.79)	0.500 (0.312 to 0.685)	1.06 (1.04 to 1.08)
Italy	2020	Endocrine, nutritional and metabolic diseases	104,000	1.74	155,000 (143,000 to 169,000)	2.61 (2.41 to 2.84)	-51,700 (-65,000 to -39,800)	-0.870 (-1.09 to -0.669)	0.67 (0.61 to 0.72)	6.6	6.40 (6.20 to 6.60)	0.200 (-0.004 to 0.396)	1.03 (1.00 to 1.06)
Italy	2021	Endocrine, nutritional and metabolic diseases	119,000	2.02	155,000 (138,000 to 173,000)	2.61 (2.33 to 2.93)	-35,400 (-54,800 to -18,600)	-0.599 (-0.927 to -0.315)	0.77 (0.69 to 0.86)	6.2	6.40 (6.12 to 6.69)	-0.200 (-0.482 to 0.081)	0.97 (0.93 to 1.01)
Italy	2020	Mental and behavioural disorders	127,000	2.13	161,000 (157,000 to 164,000)	2.70 (2.64 to 2.76)	-33,700 (-37,300 to -30,300)	-0.568 (-0.628 to -0.509)	0.79 (0.77 to 0.81)	14.6	13.92 (13.64 to 14.21)	0.680 (0.393 to 0.962)	1.05 (1.03 to 1.07)
Italy	2021	Mental and behavioural disorders	135,000	2.29	157,000 (152,000 to 162,000)	2.65 (2.57 to 2.73)	-21,400 (-26,400 to -16,700)	-0.362 (-0.446 to -0.282)	0.86 (0.84 to 0.89)	14.1	13.94 (13.55 to 14.33)	0.164 (-0.229 to 0.545)	1.01 (0.98 to 1.04)
Italy	2020	Diseases of the nervous system	118,000	1.99	159,000 (156,000 to 162,000)	2.68 (2.63 to 2.73)	-41,300 (-44,300 to -38,300)	-0.694 (-0.745 to -0.644)	0.74 (0.73 to 0.76)	15.1	14.14 (13.76 to 14.53)	0.960 (0.572 to 1.34)	1.07 (1.04 to 1.10)
Italy	2021	Diseases of the nervous system	126,000	2.12	155,000 (149,000 to 162,000)	2.63 (2.52 to 2.74)	-29,800 (-36,400 to -23,400)	-0.503 (-0.616 to -0.397)	0.81 (0.78 to 0.84)	14.5	14.28 (13.74 to 14.85)	0.218 (-0.360 to 0.762)	1.02 (0.98 to 1.06)
Italy	2020	Diseases of the eye and adnexa	38,600	0.649	49,400 (46,900 to 51,900)	0.830 (0.789 to 0.873)	-10,800 (-13,400 to -8,320)	-0.181 (-0.225 to -0.140)	0.78 (0.74 to 0.82)	2.8	2.90 (2.74 to 3.07)	-0.100 (-0.267 to 0.059)	0.97 (0.91 to 1.02)

Location	Year	Condition	Observed hospitalizations	Observed hospitalizations per 1,000 population	Expected hospitalizations	Expected hospitalizations per 1,000 population	Difference in hospitalizations	Difference in rate	Hospitalizations observed-to-expected ratio	Observed length of stay	Expected length of stay	Difference in length of stay	Length of stay observed-to-expected ratio
Italy	2021	Diseases of the eye and adnexa	39,900	0.674	45,900 (42,700 to 49,300)	0.776 (0.723 to 0.834)	-6,010 (-9,330 to -2,920)	-0.102 (-0.158 to -0.049)	0.87 (0.81 to 0.93)	2.7	2.90 (2.68 to 3.14)	-0.200 (-0.436 to 0.023)	0.93 (0.86 to 1.01)
Italy	2020	Diseases of the ear and mastoid process	15,400	0.259	25,100 (23,800 to 26,500)	0.422 (0.400 to 0.446)	-9,740 (-11,200 to -8,440)	-0.164 (-0.188 to -0.142)	0.61 (0.58 to 0.65)	3.7	3.59 (3.49 to 3.68)	0.114 (0.017 to 0.210)	1.03 (1.00 to 1.06)
Italy	2021	Diseases of the ear and mastoid process	16,700	0.283	23,600 (20,900 to 26,700)	0.399 (0.353 to 0.451)	-6,860 (-9,950 to -4,170)	-0.116 (-0.168 to -0.071)	0.71 (0.63 to 0.80)	3.5	3.55 (3.46 to 3.65)	-0.053 (-0.155 to 0.043)	0.99 (0.96 to 1.01)
Italy	2020	Diseases of the circulatory system	839,000	14.1	1,060,000 (1,040,000 to 1,080,000)	17.8 (17.4 to 18.2)	-219,000 (-242,000 to -196,000)	-3.68 (-4.07 to -3.29)	0.79 (0.78 to 0.81)	9.7	9.40 (9.25 to 9.55)	0.300 (0.151 to 0.449)	1.03 (1.02 to 1.05)
Italy	2021	Diseases of the circulatory system	894,000	15.1	1,050,000 (1,000,000 to 1,100,000)	17.7 (16.9 to 18.6)	-156,000 (-208,000 to -107,000)	-2.64 (-3.51 to -1.81)	0.85 (0.81 to 0.89)	9.3	9.40 (9.19 to 9.61)	-0.100 (-0.310 to 0.111)	0.99 (0.97 to 1.01)
Italy	2020	Diseases of the respiratory system	426,000	7.16	601,000 (570,000 to 633,000)	10.1 (9.58 to 10.7)	-175,000 (-208,000 to -143,000)	-2.94 (-3.49 to -2.41)	0.71 (0.67 to 0.75)	10.4	9.58 (9.41 to 9.75)	0.819 (0.645 to 0.990)	1.09 (1.07 to 1.11)
Italy	2021	Diseases of the respiratory system	404,000	6.83	598,000 (567,000 to 630,000)	10.1 (9.58 to 10.7)	-193,000 (-226,000 to -162,000)	-3.27 (-3.82 to -2.74)	0.68 (0.64 to 0.71)	10.8	9.66 (9.42 to 9.91)	1.14 (0.893 to 1.38)	1.12 (1.09 to 1.15)
Italy	2020	Diseases of the digestive system	439,000	7.39	582,000 (571,000 to 593,000)	9.79 (9.60 to 9.98)	-143,000 (-154,000 to -132,000)	-2.40 (-2.59 to -2.21)	0.75 (0.74 to 0.77)	7	6.70 (6.61 to 6.79)	0.300 (0.207 to 0.391)	1.04 (1.03 to 1.06)
Italy	2021	Diseases of the digestive system	478,000	8.08	573,000 (548,000 to 599,000)	9.69 (9.27 to 10.1)	-95,100 (-120,000 to -70,800)	-1.61 (-2.03 to -1.20)	0.83 (0.80 to 0.87)	6.7	6.70 (6.61 to 6.79)	0.000 (-0.091 to 0.093)	1.00 (0.99 to 1.01)
Italy	2020	Diseases of the skin and subcutaneous tissue	28,400	0.477	42,800 (40,400 to 45,300)	0.719 (0.679 to 0.762)	-14,400 (-16,900 to -12,000)	-0.242 (-0.284 to -0.202)	0.66 (0.63 to 0.70)	7.5	6.89 (6.61 to 7.18)	0.611 (0.322 to 0.882)	1.09 (1.04 to 1.13)
Italy	2021	Diseases of the skin and subcutaneous tissue	28,300	0.478	40,400 (37,300 to 43,900)	0.684 (0.631 to 0.742)	-12,200 (-15,500 to -9,050)	-0.206 (-0.262 to -0.153)	0.70 (0.65 to 0.76)	7.5	6.89 (6.61 to 7.18)	0.611 (0.330 to 0.883)	1.09 (1.05 to 1.13)
Italy	2020	Diseases of musculoskeletal system and connective tissue	322,000	5.42	440,000 (428,000 to 453,000)	7.41 (7.21 to 7.62)	-119,000 (-131,000 to -107,000)	-1.99 (-2.21 to -1.79)	0.73 (0.71 to 0.75)	6.3	6.33 (6.11 to 6.55)	-0.027 (-0.251 to 0.190)	1.00 (0.96 to 1.03)
Italy	2021	Diseases of musculoskeletal system and connective tissue	379,000	6.41	433,000 (407,000 to 461,000)	7.33 (6.88 to 7.80)	-54,400 (-81,900 to -28,200)	-0.920 (-1.39 to -0.477)	0.87 (0.82 to 0.93)	6	6.25 (5.95 to 6.57)	-0.254 (-0.562 to 0.046)	0.96 (0.91 to 1.01)
Italy	2020	Diseases of the genitourinary system	318,000	5.35	430,000 (414,000 to 447,000)	7.24 (6.97 to 7.52)	-112,000 (-129,000 to -96,100)	-1.89 (-2.16 to -1.62)	0.74 (0.71 to 0.77)	5.9	5.70 (5.63 to 5.77)	0.200 (0.133 to 0.266)	1.04 (1.02 to 1.05)
Italy	2021	Diseases of the genitourinary system	351,000	5.94	434,000 (399,000 to 472,000)	7.34 (6.75 to 7.98)	-82,700 (-121,000 to -48,100)	-1.40 (-2.04 to -0.814)	0.81 (0.74 to 0.88)	5.7	5.70 (5.61 to 5.79)	0.000 (-0.095 to 0.094)	1.00 (0.98 to 1.02)
Italy	2020	Pregnancy, childbirth and the puerperium	478,000	8.05	489,000 (477,000 to 501,000)	8.23 (8.02 to 8.43)	-10,500 (-23,000 to 1,840)	-0.177 (-0.387 to 0.031)	0.98 (0.95 to 1.00)	3.7	3.82 (3.74 to 3.90)	-0.120 (-0.205 to -0.039)	0.97 (0.95 to 0.99)
Italy	2021	Pregnancy, childbirth and the puerperium	466,000	7.88	467,000 (451,000 to 484,000)	7.90 (7.63 to 8.18)	-1,280 (-17,800 to 15,300)	-0.022 (-0.301 to 0.258)	1.00 (0.96 to 1.03)	3.7	3.82 (3.74 to 3.90)	-0.120 (-0.204 to -0.039)	0.97 (0.95 to 0.99)
Italy	2020	Certain conditions originating in the perinatal period	60,000	1.01	59,700 (55,400 to 64,300)	1.00 (0.932 to 1.08)	286 (-4,280 to 4,450)	0.005 (-0.072 to 0.075)	1.00 (0.93 to 1.08)	9.5	9.40 (9.03 to 9.79)	0.100 (-0.293 to 0.481)	1.01 (0.97 to 1.05)

Location	Year	Condition	Observed hospitalizations	Observed hospitalizations per 1,000 population	Expected hospitalizations	Expected hospitalizations per 1,000 population	Difference in hospitalizations	Difference in rate	Hospitalizations observed-to-expected ratio	Observed length of stay	Expected length of stay	Difference in length of stay	Length of stay observed-to-expected ratio
Italy	2021	Certain conditions originating in the perinatal period	65,100	1.1	55,800 (50,200 to 62,000)	0.944 (0.849 to 1.05)	9,340 (3,080 to 14,900)	0.158 (0.052 to 0.252)	1.17 (1.05 to 1.30)	9.1	9.40 (8.88 to 9.95)	-0.300 (-0.853 to 0.220)	0.97 (0.91 to 1.02)
Italy	2020	Congenital malformations, deformations and chromosomal abnormalities	45,300	0.762	59,600 (56,700 to 62,600)	1.00 (0.954 to 1.05)	-14,300 (-17,300 to -11,400)	-0.240 (-0.291 to -0.193)	0.76 (0.72 to 0.80)	5.9	5.40 (5.24 to 5.56)	0.500 (0.338 to 0.654)	1.09 (1.06 to 1.12)
Italy	2021	Congenital malformations, deformations and chromosomal abnormalities	52,900	0.895	57,700 (53,800 to 61,900)	0.976 (0.910 to 1.05)	-4,830 (-9,030 to -887)	-0.082 (-0.153 to -0.015)	0.92 (0.85 to 0.98)	5.4	5.40 (5.18 to 5.63)	0.000 (-0.241 to 0.223)	1.00 (0.96 to 1.04)
Italy	2020	Symptoms, signs and abnormal clinical and laboratory findings, n.e.c.	162,000	2.73	225,000 (217,000 to 232,000)	3.78 (3.66 to 3.91)	-62,300 (-69,800 to -54,900)	-1.05 (-1.17 to -0.924)	0.72 (0.70 to 0.75)	8.6	8.03 (7.78 to 8.30)	0.567 (0.301 to 0.828)	1.07 (1.04 to 1.11)
Italy	2021	Symptoms, signs and abnormal clinical and laboratory findings, n.e.c.	160,000	2.71	214,000 (205,000 to 225,000)	3.63 (3.46 to 3.80)	-54,300 (-64,400 to -44,600)	-0.918 (-1.09 to -0.754)	0.75 (0.71 to 0.78)	8.8	8.17 (7.80 to 8.55)	0.632 (0.233 to 1.00)	1.08 (1.03 to 1.13)
Italy	2020	Injury, poisoning and other consequences of external causes	494,000	8.32	592,000 (571,000 to 613,000)	9.96 (9.61 to 10.3)	-97,300 (-119,000 to -76,300)	-1.64 (-2.00 to -1.28)	0.84 (0.81 to 0.87)	10	10.23 (9.98 to 10.49)	-0.230 (-0.481 to 0.022)	0.98 (0.95 to 1.00)
Italy	2021	Injury, poisoning and other consequences of external causes	513,000	8.68	591,000 (546,000 to 640,000)	10.00 (9.24 to 10.8)	-77,700 (-126,000 to -32,300)	-1.31 (-2.14 to -0.547)	0.87 (0.80 to 0.94)	9.7	10.36 (10.01 to 10.73)	-0.662 (-1.04 to -0.300)	0.94 (0.90 to 0.97)
Italy	2020	Factors influencing health status and contact with health services	587,000	9.87	661,000 (643,000 to 680,000)	11.1 (10.8 to 11.4)	-74,300 (-93,300 to -55,700)	-1.25 (-1.57 to -0.938)	0.89 (0.86 to 0.91)	6	6.70 (6.45 to 6.95)	-0.695 (-0.955 to -0.451)	0.90 (0.86 to 0.93)
Italy	2021	Factors influencing health status and contact with health services	603,000	10.2	644,000 (619,000 to 671,000)	10.9 (10.5 to 11.3)	-41,000 (-67,500 to -16,200)	-0.694 (-1.14 to -0.274)	0.94 (0.90 to 0.97)	5.9	6.79 (6.44 to 7.16)	-0.892 (-1.26 to -0.545)	0.87 (0.82 to 0.92)
Korea	2020	All causes	7,910,000	153	9,410,000 (8,910,000 to 9,940,000)	182 (172 to 192)	-1,500,000 (-2,020,000 to -1,010,000)	-29.0 (-39.0 to -19.5)	0.84 (0.80 to 0.89)				
Korea	2021	All causes	7,840,000	151	9,650,000 (8,930,000 to 10,400,000)	186 (173 to 201)	-1,810,000 (-2,570,000 to -1,080,000)	-34.9 (-49.7 to -20.8)	0.81 (0.75 to 0.88)				
Korea	2020	Infectious and parasitic diseases	320,000	6.18	496,000 (457,000 to 538,000)	9.57 (8.82 to 10.4)	-176,000 (-218,000 to -138,000)	-3.39 (-4.21 to -2.66)	0.65 (0.59 to 0.70)	17.4	11.00 (9.68 to 12.50)	6.40 (4.89 to 7.74)	1.58 (1.39 to 1.80)
Korea	2021	Infectious and parasitic diseases	328,000	6.35	514,000 (459,000 to 577,000)	9.94 (8.86 to 11.2)	-186,000 (-248,000 to -130,000)	-3.59 (-4.79 to -2.51)	0.64 (0.57 to 0.72)	19.2	11.00 (9.18 to 13.18)	8.20 (6.00 to 10.02)	1.75 (1.45 to 2.09)
Korea	2020	Neoplasms	1,140,000	22	1,280,000 (1,160,000 to 1,420,000)	24.7 (22.3 to 27.5)	-141,000 (-280,000 to -12,800)	-2.72 (-5.40 to -0.246)	0.89 (0.80 to 0.99)	11.3	11.41 (10.90 to 11.94)	-0.107 (-0.638 to 0.401)	0.99 (0.95 to 1.04)
Korea	2021	Neoplasms	1,190,000	23	1,340,000 (1,160,000 to 1,560,000)	26.0 (22.4 to 30.1)	-153,000 (-365,000 to 32,000)	-2.96 (-7.05 to 0.618)	0.89 (0.77 to 1.03)	11.1	11.41 (10.90 to 11.94)	-0.307 (-0.825 to 0.209)	0.97 (0.93 to 1.02)

Location	Year	Condition	Observed hospitalizations	Observed hospitalizations per 1,000 population	Expected hospitalizations	Expected hospitalizations per 1,000 population	Difference in hospitalizations	Difference in rate	Hospitalizations observed-to-expected ratio	Observed length of stay	Expected length of stay	Difference in length of stay	Length of stay observed-to-expected ratio
Korea	2020	Diseases of the blood and bloodforming organs	25,700	0.495	27,400 (21,800 to 34,300)	0.528 (0.421 to 0.661)	-1,700 (-8,600 to 3,800)	-0.033 (-0.166 to 0.073)	0.94 (0.75 to 1.17)	8.6	9.72 (7.79 to 12.12)	-1.12 (-3.50 to 0.787)	0.89 (0.71 to 1.10)
Korea	2021	Diseases of the blood and bloodforming organs	25,100	0.485	27,300 (21,800 to 34,200)	0.528 (0.421 to 0.661)	-2,220 (-9,270 to 3,360)	-0.043 (-0.179 to 0.065)	0.92 (0.73 to 1.15)	9.1	9.72 (7.79 to 12.12)	-0.616 (-2.99 to 1.32)	0.94 (0.75 to 1.17)
Korea	2020	Endocrine, nutritional and metabolic diseases	130,000	2.52	151,000 (127,000 to 178,000)	2.91 (2.46 to 3.44)	-20,300 (-48,500 to 2,570)	-0.393 (-0.936 to 0.050)	0.87 (0.73 to 1.02)	17.8	20.75 (17.67 to 24.36)	-2.95 (-6.67 to 0.185)	0.86 (0.73 to 1.01)
Korea	2021	Endocrine, nutritional and metabolic diseases	131,000	2.53	151,000 (127,000 to 178,000)	2.91 (2.46 to 3.44)	-19,900 (-47,700 to 3,700)	-0.384 (-0.922 to 0.071)	0.87 (0.73 to 1.03)	17.5	20.75 (17.67 to 24.36)	-3.25 (-6.96 to -0.210)	0.84 (0.72 to 0.99)
Korea	2020	Mental and behavioural disorders	205,000	3.96	259,000 (233,000 to 288,000)	4.99 (4.49 to 5.55)	-53,800 (-81,800 to -28,000)	-1.04 (-1.58 to -0.540)	0.79 (0.71 to 0.88)	200.4	185.45 (165.82 to 207.40)	14.95 (-7.12 to 34.61)	1.08 (0.97 to 1.21)
Korea	2021	Mental and behavioural disorders	189,000	3.66	258,000 (232,000 to 287,000)	4.99 (4.49 to 5.55)	-69,200 (-98,400 to -43,400)	-1.34 (-1.90 to -0.838)	0.73 (0.66 to 0.81)	186.6	195.08 (166.53 to 228.52)	-8.48 (-41.12 to 20.27)	0.96 (0.82 to 1.12)
Korea	2020	Diseases of the nervous system	232,000	4.48	288,000 (267,000 to 311,000)	5.56 (5.15 to 6.00)	-55,800 (-78,600 to -34,800)	-1.08 (-1.52 to -0.671)	0.81 (0.75 to 0.87)	72.3	72.70 (62.14 to 85.06)	-0.400 (-12.43 to 10.32)	0.99 (0.85 to 1.17)
Korea	2021	Diseases of the nervous system	228,000	4.41	302,000 (272,000 to 337,000)	5.84 (5.25 to 6.51)	-74,300 (-109,000 to -43,500)	-1.44 (-2.10 to -0.840)	0.75 (0.68 to 0.84)	81.1	78.53 (62.90 to 98.05)	2.57 (-17.08 to 17.94)	1.03 (0.83 to 1.28)
Korea	2020	Diseases of the eye and adnexa	64,200	1.24	71,000 (62,900 to 80,100)	1.37 (1.21 to 1.55)	-6,740 (-16,000 to 1,430)	-0.130 (-0.308 to 0.028)	0.90 (0.80 to 1.02)	3.1	2.90 (2.03 to 4.14)	0.200 (-1.01 to 1.06)	1.07 (0.75 to 1.52)
Korea	2021	Diseases of the eye and adnexa	66,400	1.28	70,900 (59,700 to 84,100)	1.37 (1.15 to 1.63)	-4,400 (-18,100 to 6,690)	-0.085 (-0.350 to 0.129)	0.94 (0.79 to 1.11)	2.9	2.90 (1.75 to 4.79)	0.000 (-1.87 to 1.16)	1.00 (0.61 to 1.67)
Korea	2020	Diseases of the ear and mastoid process	75,800	1.46	91,600 (80,000 to 105,000)	1.77 (1.54 to 2.02)	-15,800 (-29,200 to -4,170)	-0.304 (-0.563 to -0.080)	0.83 (0.72 to 0.95)	4.6	4.47 (4.26 to 4.71)	0.125 (-0.100 to 0.347)	1.03 (0.98 to 1.08)
Korea	2021	Diseases of the ear and mastoid process	67,700	1.31	91,400 (75,600 to 111,000)	1.77 (1.46 to 2.14)	-23,700 (-42,800 to -7,780)	-0.458 (-0.828 to -0.150)	0.74 (0.61 to 0.90)	4.6	4.35 (4.05 to 4.67)	0.248 (-0.073 to 0.551)	1.06 (0.98 to 1.14)
Korea	2020	Diseases of the circulatory system	529,000	10.2	583,000 (525,000 to 646,000)	11.2 (10.1 to 12.5)	-54,000 (-117,000 to 4,920)	-1.04 (-2.26 to 0.095)	0.91 (0.82 to 1.01)	31.6	33.84 (30.56 to 37.47)	-2.24 (-5.87 to 1.11)	0.93 (0.84 to 1.04)
Korea	2021	Diseases of the circulatory system	547,000	10.6	582,000 (502,000 to 673,000)	11.2 (9.71 to 13.0)	-34,300 (-125,000 to 45,600)	-0.663 (-2.41 to 0.881)	0.94 (0.81 to 1.09)	32.2	33.84 (30.56 to 37.47)	-1.64 (-5.32 to 1.68)	0.95 (0.86 to 1.05)
Korea	2020	Diseases of the respiratory system	531,000	10.3	1,030,000 (887,000 to 1,210,000)	19.9 (17.1 to 23.3)	-503,000 (-674,000 to -354,000)	-9.70 (-13.0 to -6.84)	0.51 (0.44 to 0.60)	18.7	12.00 (10.59 to 13.60)	6.70 (5.11 to 8.10)	1.56 (1.38 to 1.76)
Korea	2021	Diseases of the respiratory system	429,000	8.29	1,030,000 (831,000 to 1,280,000)	19.9 (16.1 to 24.8)	-603,000 (-853,000 to -403,000)	-11.7 (-16.5 to -7.78)	0.42 (0.33 to 0.52)	21.9	12.00 (10.05 to 14.33)	9.90 (7.53 to 11.85)	1.82 (1.52 to 2.18)
Korea	2020	Diseases of the digestive system	671,000	12.9	685,000 (609,000 to 770,000)	13.2 (11.7 to 14.9)	-13,700 (-98,700 to 60,600)	-0.264 (-1.90 to 1.17)	0.98 (0.87 to 1.10)	6.5	6.40 (5.07 to 8.07)	0.100 (-1.61 to 1.44)	1.02 (0.80 to 1.28)
Korea	2021	Diseases of the digestive system	673,000	13	684,000 (608,000 to 769,000)	13.2 (11.7 to 14.9)	-10,700 (-96,900 to 64,600)	-0.206 (-1.87 to 1.25)	0.98 (0.87 to 1.11)	6.5	6.40 (4.61 to 8.89)	0.100 (-2.34 to 1.88)	1.02 (0.74 to 1.41)
Korea	2020	Diseases of the skin and	61,900	1.2	73,900 (67,800 to 80,600)	1.43 (1.31 to 1.56)	-12,000 (-18,800 to -5,850)	-0.231 (-0.363 to -0.113)	0.84 (0.77 to 0.91)	16.2	15.60 (13.29 to 18.31)	0.600 (-2.07 to 2.91)	1.04 (0.89 to 1.22)

Location	Year	Condition	Observed hospitalizations	Observed hospitalizations per 1,000 population	Expected hospitalizations	Expected hospitalizations per 1,000 population	Difference in hospitalizations	Difference in rate	Hospitalizations observed-to-expected ratio	Observed length of stay	Expected length of stay	Difference in length of stay	Length of stay observed-to-expected ratio
		subcutaneous tissue											
Korea	2021	Diseases of the skin and subcutaneous tissue	55,100	1.07	73,800 (65,300 to 83,400)	1.43 (1.26 to 1.61)	-18,700 (-28,200 to -10,100)	-0.361 (-0.544 to -0.195)	0.75 (0.66 to 0.85)	17	15.60 (12.44 to 19.57)	1.40 (-2.69 to 4.57)	1.09 (0.86 to 1.37)
Korea	2020	Diseases of musculoskeletal system and connective tissue	1,210,000	23.3	1,340,000 (1,200,000 to 1,490,000)	25.8 (23.2 to 28.8)	-132,000 (-286,000 to 4,450)	-2.54 (-5.51 to 0.086)	0.90 (0.81 to 1.00)	10	9.81 (8.85 to 10.87)	0.191 (-0.862 to 1.14)	1.02 (0.92 to 1.13)
Korea	2021	Diseases of musculoskeletal system and connective tissue	1,170,000	22.5	1,390,000 (1,090,000 to 1,770,000)	26.8 (21.1 to 34.1)	-223,000 (-601,000 to 78,100)	-4.31 (-11.6 to 1.51)	0.84 (0.66 to 1.07)	10	9.43 (8.16 to 10.90)	0.567 (-0.859 to 1.84)	1.06 (0.92 to 1.23)
Korea	2020	Diseases of the genitourinary system	365,000	7.03	425,000 (406,000 to 444,000)	8.20 (7.84 to 8.57)	-60,400 (-79,300 to -42,100)	-1.16 (-1.53 to -0.811)	0.86 (0.82 to 0.90)	12	11.40 (10.40 to 12.49)	0.600 (-0.469 to 1.61)	1.05 (0.96 to 1.15)
Korea	2021	Diseases of the genitourinary system	358,000	6.92	440,000 (414,000 to 469,000)	8.51 (7.99 to 9.07)	-82,200 (-111,000 to -55,400)	-1.59 (-2.14 to -1.07)	0.81 (0.76 to 0.87)	12.7	11.40 (10.02 to 12.98)	1.30 (-0.249 to 2.71)	1.11 (0.98 to 1.27)
Korea	2020	Pregnancy, childbirth and the puerperium	216,000	4.17	242,000 (217,000 to 270,000)	4.67 (4.19 to 5.22)	-26,100 (-54,000 to -391)	-0.503 (-1.04 to -0.008)	0.89 (0.80 to 1.00)	4.6	4.35 (4.08 to 4.64)	0.252 (-0.042 to 0.527)	1.06 (0.99 to 1.13)
Korea	2021	Pregnancy, childbirth and the puerperium	209,000	4.05	242,000 (207,000 to 282,000)	4.67 (4.00 to 5.46)	-32,300 (-73,300 to 2,260)	-0.624 (-1.42 to 0.044)	0.87 (0.74 to 1.01)	4.7	4.35 (4.08 to 4.64)	0.352 (0.070 to 0.618)	1.08 (1.02 to 1.15)
Korea	2020	Certain conditions originating in the perinatal period	34,100	0.658	36,800 (25,800 to 52,400)	0.710 (0.498 to 1.01)	-2,690 (-18,400 to 8,360)	-0.052 (-0.354 to 0.161)	0.93 (0.65 to 1.32)	13.1	12.90 (10.13 to 16.42)	0.200 (-3.37 to 2.96)	1.02 (0.80 to 1.29)
Korea	2021	Certain conditions originating in the perinatal period	34,200	0.66	36,700 (22,300 to 60,600)	0.710 (0.430 to 1.17)	-2,590 (-26,400 to 12,100)	-0.050 (-0.510 to 0.234)	0.93 (0.56 to 1.55)	13.5	12.90 (9.17 to 18.15)	0.600 (-4.55 to 4.32)	1.05 (0.75 to 1.47)
Korea	2020	Congenital malformations, deformations and chromosomal abnormalities	31,200	0.601	34,800 (31,500 to 38,500)	0.672 (0.607 to 0.743)	-3,660 (-7,310 to -381)	-0.071 (-0.141 to -0.007)	0.89 (0.81 to 0.99)	8.5	8.40 (8.02 to 8.79)	0.104 (-0.299 to 0.488)	1.01 (0.97 to 1.06)
Korea	2021	Congenital malformations, deformations and chromosomal abnormalities	30,800	0.595	34,800 (30,100 to 40,100)	0.672 (0.582 to 0.775)	-3,970 (-9,360 to 653)	-0.077 (-0.181 to 0.013)	0.89 (0.77 to 1.02)	8.4	8.47 (8.00 to 8.98)	-0.075 (-0.576 to 0.404)	0.99 (0.94 to 1.05)
Korea	2020	Symptoms, signs and abnormal clinical and laboratory findings, n.e.c.	213,000	4.1	258,000 (198,000 to 336,000)	4.97 (3.81 to 6.49)	-45,200 (-124,000 to 15,700)	-0.872 (-2.38 to 0.302)	0.82 (0.63 to 1.08)	7.8	7.62 (7.01 to 8.29)	0.176 (-0.487 to 0.789)	1.02 (0.94 to 1.11)
Korea	2021	Symptoms, signs and abnormal clinical and laboratory findings, n.e.c.	239,000	4.62	257,000 (177,000 to 375,000)	4.97 (3.41 to 7.25)	-18,400 (-133,000 to 63,900)	-0.355 (-2.57 to 1.23)	0.93 (0.64 to 1.36)	7.7	7.62 (7.01 to 8.29)	0.076 (-0.592 to 0.697)	1.01 (0.93 to 1.10)
Korea	2020	Injury, poisoning and other consequences of external causes	1,450,000	27.9	1,400,000 (1,210,000 to 1,620,000)	27.0 (23.3 to 31.2)	46,800 (-174,000 to 239,000)	0.903 (-3.36 to 4.62)	1.03 (0.89 to 1.20)	12.3	11.50 (10.32 to 12.81)	0.800 (-0.475 to 2.00)	1.07 (0.96 to 1.19)

Location	Year	Condition	Observed hospitalizations	Observed hospitalizations per 1,000 population	Expected hospitalizations	Expected hospitalizations per 1,000 population	Difference in hospitalizations	Difference in rate	Hospitalizations observed-to-expected ratio	Observed length of stay	Expected length of stay	Difference in length of stay	Length of stay observed-to-expected ratio
Korea	2021	Injury, poisoning and other consequences of external causes	1,420,000	27.4	1,400,000 (1,210,000 to 1,620,000)	27.0 (23.3 to 31.2)	23,200 (-197,000 to 211,000)	0.448 (-3.81 to 4.07)	1.02 (0.88 to 1.17)	12	11.50 (9.87 to 13.40)	0.500 (-1.41 to 2.17)	1.04 (0.90 to 1.22)
Korea	2020	Factors influencing health status and contact with health services	409,000	7.89	351,000 (208,000 to 592,000)	6.78 (4.02 to 11.4)	57,900 (-189,000 to 200,000)	1.12 (-3.65 to 3.85)	1.16 (0.68 to 1.95)	5.8	5.70 (4.92 to 6.61)	0.100 (-0.804 to 0.882)	1.02 (0.88 to 1.18)
Korea	2021	Factors influencing health status and contact with health services	451,000	8.71	351,000 (168,000 to 734,000)	6.78 (3.24 to 14.2)	100,000 (-274,000 to 283,000)	1.94 (-5.30 to 5.48)	1.29 (0.62 to 2.69)	6.6	5.70 (4.63 to 7.02)	0.900 (-0.431 to 2.01)	1.16 (0.94 to 1.44)
Lithuania	2020	All causes	438,000	157	610,000 (602,000 to 618,000)	218 (215 to 221)	-172,000 (-180,000 to -163,000)	-61.4 (-64.3 to -58.4)	0.72 (0.71 to 0.73)				
Lithuania	2021	All causes	450,000	161	605,000 (594,000 to 617,000)	216 (212 to 220)	-155,000 (-167,000 to -144,000)	-55.3 (-59.5 to -51.2)	0.74 (0.73 to 0.76)				
Lithuania	2020	Infectious and parasitic diseases	15,200	5.45	28,200 (26,700 to 29,800)	10.1 (9.54 to 10.7)	-13,000 (-14,600 to -11,400)	-4.64 (-5.21 to -4.09)	0.54 (0.51 to 0.57)	15.6	10.32 (9.11 to 11.69)	5.28 (3.90 to 6.48)	1.51 (1.33 to 1.71)
Lithuania	2021	Infectious and parasitic diseases	14,500	5.18	28,300 (26,700 to 29,900)	10.1 (9.54 to 10.7)	-13,700 (-15,400 to -12,200)	-4.91 (-5.49 to -4.37)	0.51 (0.49 to 0.54)	12.7	10.32 (9.11 to 11.69)	2.38 (0.985 to 3.59)	1.23 (1.08 to 1.39)
Lithuania	2020	Neoplasms	45,400	16.3	53,500 (51,700 to 55,300)	19.1 (18.5 to 19.8)	-8,040 (-9,930 to -6,250)	-2.88 (-3.55 to -2.24)	0.85 (0.82 to 0.88)	7.3	7.19 (7.00 to 7.39)	0.108 (-0.090 to 0.305)	1.01 (0.99 to 1.04)
Lithuania	2021	Neoplasms	45,700	16.3	53,600 (51,800 to 55,400)	19.1 (18.5 to 19.8)	-7,870 (-9,730 to -6,100)	-2.81 (-3.47 to -2.18)	0.85 (0.82 to 0.88)	6.7	7.09 (6.82 to 7.37)	-0.386 (-0.664 to -0.115)	0.95 (0.91 to 0.98)
Lithuania	2020	Diseases of the blood and bloodforming organs	4,900	1.75	5,860 (4,860 to 7,070)	2.10 (1.74 to 2.53)	-963 (-2,180 to 31)	-0.344 (-0.780 to 0.011)	0.84 (0.69 to 1.01)	6.4	6.64 (6.39 to 6.90)	-0.236 (-0.495 to 0.015)	0.96 (0.93 to 1.00)
Lithuania	2021	Diseases of the blood and bloodforming organs	5,250	1.88	5,870 (4,510 to 7,660)	2.10 (1.61 to 2.73)	-620 (-2,380 to 754)	-0.221 (-0.850 to 0.269)	0.89 (0.69 to 1.17)	6.4	6.64 (6.39 to 6.90)	-0.236 (-0.492 to 0.011)	0.96 (0.93 to 1.00)
Lithuania	2020	Endocrine, nutritional and metabolic diseases	7,390	2.64	10,700 (9,530 to 12,000)	3.83 (3.41 to 4.31)	-3,320 (-4,660 to -2,140)	-1.19 (-1.67 to -0.767)	0.69 (0.61 to 0.78)	7.3	7.22 (7.00 to 7.46)	0.076 (-0.158 to 0.307)	1.01 (0.98 to 1.04)
Lithuania	2021	Endocrine, nutritional and metabolic diseases	8,540	3.05	10,700 (9,550 to 12,100)	3.83 (3.41 to 4.31)	-2,190 (-3,540 to -991)	-0.782 (-1.26 to -0.354)	0.80 (0.71 to 0.90)	7	7.22 (7.00 to 7.46)	-0.224 (-0.457 to -0.003)	0.97 (0.94 to 1.00)
Lithuania	2020	Mental and behavioural disorders	25,400	9.11	34,600 (33,200 to 36,100)	12.4 (11.9 to 12.9)	-9,180 (-10,700 to -7,710)	-3.29 (-3.81 to -2.76)	0.73 (0.70 to 0.77)	20.7	18.17 (16.65 to 19.83)	2.53 (0.835 to 4.07)	1.14 (1.04 to 1.24)
Lithuania	2021	Mental and behavioural disorders	25,400	9.08	34,700 (33,300 to 36,200)	12.4 (11.9 to 12.9)	-9,280 (-10,800 to -7,830)	-3.31 (-3.85 to -2.80)	0.73 (0.70 to 0.76)	19.6	18.17 (16.65 to 19.83)	1.43 (-0.248 to 2.95)	1.08 (0.99 to 1.18)
Lithuania	2020	Diseases of the nervous system	13,100	4.7	22,100 (19,400 to 25,000)	7.89 (6.95 to 8.96)	-8,910 (-11,800 to -6,250)	-3.19 (-4.23 to -2.24)	0.60 (0.53 to 0.68)	7.5	7.38 (6.60 to 8.24)	0.123 (-0.735 to 0.889)	1.02 (0.91 to 1.13)
Lithuania	2021	Diseases of the nervous system	12,200	4.37	22,100 (19,500 to 25,100)	7.89 (6.95 to 8.96)	-9,870 (-12,900 to -7,260)	-3.52 (-4.61 to -2.59)	0.55 (0.49 to 0.63)	7.9	7.38 (6.60 to 8.24)	0.523 (-0.351 to 1.30)	1.07 (0.96 to 1.20)
Lithuania	2020	Diseases of the eye and adnexa	11,000	3.94	17,200 (15,800 to 18,800)	6.16 (5.64 to 6.72)	-6,200 (-7,750 to -4,790)	-2.22 (-2.77 to -1.71)	0.64 (0.59 to 0.70)	2.4	2.64 (2.27 to 3.08)	-0.243 (-0.680 to 0.137)	0.91 (0.78 to 1.06)

Location	Year	Condition	Observed hospitalizations	Observed hospitalizations per 1,000 population	Expected hospitalizations	Expected hospitalizations per 1,000 population	Difference in hospitalizations	Difference in rate	Hospitalizations observed-to-expected ratio	Observed length of stay	Expected length of stay	Difference in length of stay	Length of stay observed-to-expected ratio
Lithuania	2021	Diseases of the eye and adnexa	12,400	4.41	17,300 (15,800 to 18,800)	6.16 (5.64 to 6.72)	-4,900 (-6,490 to -3,470)	-1.75 (-2.32 to -1.24)	0.72 (0.66 to 0.78)	2.4	2.64 (2.27 to 3.08)	-0.243 (-0.678 to 0.134)	0.91 (0.78 to 1.06)
Lithuania	2020	Diseases of the ear and mastoid process	2,300	0.825	3,840 (3,570 to 4,120)	1.37 (1.28 to 1.48)	-1,530 (-1,820 to -1,260)	-0.548 (-0.652 to -0.451)	0.60 (0.56 to 0.65)	5.1	6.20 (5.83 to 6.59)	-1.10 (-1.50 to -0.733)	0.82 (0.77 to 0.87)
Lithuania	2021	Diseases of the ear and mastoid process	2,090	0.747	3,690 (3,330 to 4,090)	1.32 (1.19 to 1.46)	-1,600 (-2,000 to -1,250)	-0.572 (-0.713 to -0.445)	0.57 (0.51 to 0.63)	4.5	6.20 (5.83 to 6.59)	-1.70 (-2.09 to -1.32)	0.73 (0.68 to 0.77)
Lithuania	2020	Diseases of the circulatory system	80,100	28.6	116,000 (109,000 to 125,000)	41.7 (38.9 to 44.7)	-36,400 (-44,800 to -28,600)	-13.0 (-16.0 to -10.2)	0.69 (0.64 to 0.74)	8.4	7.87 (7.39 to 8.39)	0.529 (0.022 to 1.01)	1.07 (1.00 to 1.14)
Lithuania	2021	Diseases of the circulatory system	73,700	26.3	117,000 (106,000 to 129,000)	41.7 (37.8 to 46.0)	-43,000 (-55,000 to -32,000)	-15.3 (-19.6 to -11.4)	0.63 (0.57 to 0.70)	8.5	7.87 (7.39 to 8.39)	0.629 (0.115 to 1.12)	1.08 (1.01 to 1.15)
Lithuania	2020	Diseases of the respiratory system	39,300	14	64,600 (58,600 to 71,200)	23.1 (21.0 to 25.5)	-25,300 (-31,900 to -19,400)	-9.07 (-11.4 to -6.93)	0.61 (0.55 to 0.67)	7.3	6.40 (6.15 to 6.66)	0.900 (0.645 to 1.15)	1.14 (1.10 to 1.19)
Lithuania	2021	Diseases of the respiratory system	52,900	18.9	64,700 (58,700 to 71,400)	23.1 (21.0 to 25.5)	-11,800 (-18,400 to -5,890)	-4.22 (-6.58 to -2.10)	0.82 (0.74 to 0.90)	8.7	6.40 (6.05 to 6.76)	2.30 (1.94 to 2.65)	1.36 (1.29 to 1.44)
Lithuania	2020	Diseases of the digestive system	40,700	14.6	55,800 (54,900 to 56,700)	20.0 (19.7 to 20.3)	-15,100 (-16,000 to -14,200)	-5.39 (-5.71 to -5.07)	0.73 (0.72 to 0.74)	5.3	5.02 (4.86 to 5.19)	0.280 (0.114 to 0.443)	1.06 (1.02 to 1.09)
Lithuania	2021	Diseases of the digestive system	42,900	15.3	55,900 (54,700 to 57,200)	20.0 (19.5 to 20.4)	-13,000 (-14,200 to -11,800)	-4.64 (-5.08 to -4.20)	0.77 (0.75 to 0.78)	4.9	4.94 (4.72 to 5.17)	-0.040 (-0.273 to 0.179)	0.99 (0.95 to 1.04)
Lithuania	2020	Diseases of the skin and subcutaneous tissue	5,840	2.09	9,520 (8,770 to 10,300)	3.41 (3.14 to 3.70)	-3,680 (-4,490 to -2,930)	-1.32 (-1.61 to -1.05)	0.61 (0.57 to 0.67)	6.7	6.61 (6.39 to 6.84)	0.088 (-0.135 to 0.308)	1.01 (0.98 to 1.05)
Lithuania	2021	Diseases of the skin and subcutaneous tissue	5,560	1.99	9,540 (8,490 to 10,700)	3.41 (3.03 to 3.83)	-3,980 (-5,140 to -2,920)	-1.42 (-1.83 to -1.04)	0.58 (0.52 to 0.66)	6	6.61 (6.39 to 6.84)	-0.612 (-0.835 to -0.390)	0.91 (0.88 to 0.94)
Lithuania	2020	Diseases of musculoskeletal system and connective tissue	34,400	12.3	55,500 (49,400 to 62,400)	19.9 (17.7 to 22.3)	-21,100 (-28,000 to -14,900)	-7.55 (-10.0 to -5.33)	0.62 (0.55 to 0.70)	7.1	7.04 (6.05 to 8.20)	0.056 (-1.11 to 1.03)	1.01 (0.86 to 1.17)
Lithuania	2021	Diseases of musculoskeletal system and connective tissue	35,700	12.8	55,600 (47,100 to 65,700)	19.9 (16.8 to 23.5)	-19,900 (-29,800 to -11,400)	-7.12 (-10.7 to -4.08)	0.64 (0.54 to 0.76)	6.3	7.04 (6.05 to 8.20)	-0.744 (-1.88 to 0.247)	0.89 (0.77 to 1.04)
Lithuania	2020	Diseases of the genitourinary system	25,400	9.1	36,000 (33,700 to 38,500)	12.9 (12.1 to 13.8)	-10,600 (-13,000 to -8,280)	-3.79 (-4.66 to -2.96)	0.71 (0.66 to 0.75)	5.2	5.10 (4.89 to 5.32)	0.100 (-0.117 to 0.315)	1.02 (0.98 to 1.06)
Lithuania	2021	Diseases of the genitourinary system	25,500	9.1	36,100 (32,900 to 39,700)	12.9 (11.7 to 14.2)	-10,600 (-14,200 to -7,440)	-3.80 (-5.08 to -2.66)	0.71 (0.64 to 0.77)	4.8	5.10 (4.80 to 5.41)	-0.300 (-0.611 to 0.000)	0.94 (0.89 to 1.00)
Lithuania	2020	Pregnancy, childbirth and the puerperium	30,500	10.9	35,200 (33,600 to 36,900)	12.6 (12.0 to 13.2)	-4,660 (-6,420 to -3,040)	-1.67 (-2.30 to -1.09)	0.87 (0.83 to 0.91)	3.8	3.90 (3.67 to 4.15)	-0.100 (-0.340 to 0.138)	0.97 (0.92 to 1.04)
Lithuania	2021	Pregnancy, childbirth and the puerperium	30,000	10.7	34,400 (32,100 to 36,800)	12.3 (11.5 to 13.1)	-4,370 (-6,770 to -2,120)	-1.56 (-2.42 to -0.755)	0.87 (0.82 to 0.93)	3.8	3.90 (3.58 to 4.25)	-0.100 (-0.457 to 0.233)	0.97 (0.89 to 1.07)
Lithuania	2020	Certain conditions originating in the perinatal period	9,040	3.23	10,400 (9,660 to 11,200)	3.73 (3.46 to 4.02)	-1,380 (-2,190 to -608)	-0.493 (-0.785 to -0.217)	0.87 (0.80 to 0.94)	5.9	5.70 (5.36 to 6.06)	0.200 (-0.163 to 0.555)	1.04 (0.97 to 1.10)
Lithuania	2021	Certain conditions originating in the perinatal period	9,030	3.23	10,400 (9,680 to 11,300)	3.73 (3.46 to 4.02)	-1,410 (-2,220 to -660)	-0.502 (-0.792 to -0.236)	0.87 (0.80 to 0.93)	5.7	5.70 (5.22 to 6.22)	0.000 (-0.528 to 0.477)	1.00 (0.92 to 1.09)

Location	Year	Condition	Observed hospitalizations	Observed hospitalizations per 1,000 population	Expected hospitalizations	Expected hospitalizations per 1,000 population	Difference in hospitalizations	Difference in rate	Hospitalizations observed-to-expected ratio	Observed length of stay	Expected length of stay	Difference in length of stay	Length of stay observed-to-expected ratio
Lithuania	2020	Congenital malformations, deformations and chromosomal abnormalities	2,920	1.05	4,120 (3,720 to 4,560)	1.47 (1.33 to 1.63)	-1,200 (-1,650 to -790)	-0.428 (-0.589 to -0.283)	0.71 (0.64 to 0.79)	6.1	5.81 (4.88 to 6.91)	0.295 (-0.841 to 1.22)	1.05 (0.88 to 1.25)
Lithuania	2021	Congenital malformations, deformations and chromosomal abnormalities	3,360	1.2	4,130 (3,570 to 4,770)	1.47 (1.27 to 1.70)	-770 (-1,420 to -216)	-0.275 (-0.506 to -0.077)	0.81 (0.70 to 0.94)	5.9	5.81 (4.88 to 6.91)	0.095 (-1.02 to 1.03)	1.02 (0.85 to 1.21)
Lithuania	2020	Symptoms, signs and abnormal clinical and laboratory findings, n.e.c.	3,080	1.1	4,930 (3,200 to 7,600)	1.76 (1.14 to 2.72)	-1,850 (-4,430 to -114)	-0.661 (-1.58 to -0.041)	0.62 (0.41 to 0.96)	4	4.20 (2.58 to 6.84)	-0.200 (-2.88 to 1.43)	0.95 (0.58 to 1.56)
Lithuania	2021	Symptoms, signs and abnormal clinical and laboratory findings, n.e.c.	3,040	1.09	4,940 (2,680 to 9,110)	1.76 (0.956 to 3.25)	-1,900 (-6,100 to 361)	-0.677 (-2.18 to 0.129)	0.62 (0.33 to 1.13)	3.6	4.20 (2.11 to 8.38)	-0.600 (-4.74 to 1.50)	0.86 (0.43 to 1.71)
Lithuania	2020	Injury, poisoning and other consequences of external causes	31,700	11.3	38,300 (35,600 to 41,300)	13.7 (12.7 to 14.8)	-6,640 (-9,650 to -3,880)	-2.38 (-3.45 to -1.39)	0.83 (0.77 to 0.89)	7.6	7.50 (6.98 to 8.05)	0.100 (-0.437 to 0.613)	1.01 (0.95 to 1.09)
Lithuania	2021	Injury, poisoning and other consequences of external causes	31,900	11.4	38,400 (34,500 to 42,700)	13.7 (12.3 to 15.3)	-6,500 (-10,800 to -2,660)	-2.32 (-3.87 to -0.950)	0.83 (0.75 to 0.92)	7.4	7.50 (6.78 to 8.30)	-0.100 (-0.913 to 0.609)	0.99 (0.89 to 1.09)
Lithuania	2020	Factors influencing health status and contact with health services	10,400	3.72	17,500 (7,530 to 40,900)	6.28 (2.69 to 14.6)	-7,150 (-31,100 to 2,850)	-2.56 (-11.1 to 1.02)	0.59 (0.25 to 1.38)	2.8	2.80 (0.96 to 8.17)	0.000 (-5.53 to 1.83)	1.00 (0.34 to 2.89)
Lithuania	2021	Factors influencing health status and contact with health services	10,600	3.8	17,600 (7,550 to 40,900)	6.28 (2.69 to 14.6)	-6,930 (-30,100 to 3,090)	-2.47 (-10.8 to 1.10)	0.61 (0.26 to 1.41)	2.8	2.80 (0.62 to 12.72)	0.000 (-9.88 to 2.18)	1.00 (0.22 to 4.51)
Latvia	2020	All causes	261,000	137	313,000 (294,000 to 334,000)	165 (155 to 176)	-52,700 (-73,100 to -33,700)	-27.8 (-38.5 to -17.7)	0.83 (0.78 to 0.89)				
Latvia	2021	All causes	265,000	141	311,000 (292,000 to 331,000)	165 (155 to 176)	-45,700 (-67,000 to -26,700)	-24.3 (-35.5 to -14.2)	0.85 (0.80 to 0.91)				
Latvia	2020	Infectious and parasitic diseases	7,540	3.97	13,200 (11,400 to 15,300)	6.96 (6.02 to 8.04)	-5,680 (-7,740 to -3,900)	-2.99 (-4.07 to -2.05)	0.57 (0.49 to 0.66)	10.6	9.02 (8.16 to 9.97)	1.58 (0.607 to 2.47)	1.18 (1.06 to 1.30)
Latvia	2021	Infectious and parasitic diseases	6,490	3.45	13,100 (10,700 to 16,100)	6.96 (5.67 to 8.54)	-6,620 (-9,590 to -4,140)	-3.51 (-5.09 to -2.20)	0.50 (0.40 to 0.61)	10.1	9.02 (8.16 to 9.97)	1.08 (0.128 to 1.95)	1.12 (1.01 to 1.24)
Latvia	2020	Neoplasms	34,400	18.1	38,200 (36,800 to 39,800)	20.1 (19.3 to 20.9)	-3,810 (-5,300 to -2,320)	-2.01 (-2.79 to -1.22)	0.90 (0.87 to 0.94)	7.3	7.50 (7.39 to 7.63)	-0.204 (-0.331 to -0.086)	0.97 (0.96 to 0.99)
Latvia	2021	Neoplasms	33,600	17.8	38,700 (36,600 to 40,900)	20.6 (19.4 to 21.7)	-5,180 (-7,420 to -3,130)	-2.75 (-3.94 to -1.66)	0.87 (0.82 to 0.91)	7.1	7.41 (7.24 to 7.58)	-0.310 (-0.484 to -0.146)	0.96 (0.94 to 0.98)
Latvia	2020	Diseases of the blood and bloodforming organs	2,200	1.16	2,470 (2,280 to 2,670)	1.30 (1.20 to 1.41)	-264 (-466 to -73)	-0.139 (-0.245 to -0.038)	0.89 (0.83 to 0.97)	6.2	6.14 (5.75 to 6.56)	0.060 (-0.349 to 0.458)	1.01 (0.95 to 1.08)
Latvia	2021	Diseases of the blood and bloodforming organs	2,010	1.06	2,440 (2,180 to 2,740)	1.30 (1.16 to 1.45)	-439 (-731 to -178)	-0.233 (-0.388 to -0.095)	0.82 (0.73 to 0.92)	6.2	6.14 (5.75 to 6.56)	0.060 (-0.342 to 0.453)	1.01 (0.95 to 1.08)

Location	Year	Condition	Observed hospitalizations	Observed hospitalizations per 1,000 population	Expected hospitalizations	Expected hospitalizations per 1,000 population	Difference in hospitalizations	Difference in rate	Hospitalizations observed-to-expected ratio	Observed length of stay	Expected length of stay	Difference in length of stay	Length of stay observed-to-expected ratio
Latvia	2020	Endocrine, nutritional and metabolic diseases	3,980	2.09	4,260 (3,600 to 5,050)	2.24 (1.90 to 2.66)	-288 (-1,080 to 369)	-0.151 (-0.566 to 0.194)	0.93 (0.79 to 1.10)	6.6	6.34 (5.95 to 6.76)	0.260 (-0.148 to 0.647)	1.04 (0.98 to 1.11)
Latvia	2021	Endocrine, nutritional and metabolic diseases	4,830	2.56	4,230 (3,570 to 5,010)	2.24 (1.90 to 2.66)	604 (-170 to 1,250)	0.321 (-0.090 to 0.665)	1.14 (0.97 to 1.35)	6.1	6.34 (5.95 to 6.76)	-0.240 (-0.661 to 0.143)	0.96 (0.90 to 1.02)
Latvia	2020	Mental and behavioural disorders	19,800	10.4	24,400 (20,800 to 28,500)	12.8 (11.0 to 15.0)	-4,580 (-8,690 to -1,030)	-2.41 (-4.57 to -0.542)	0.81 (0.70 to 0.95)	30.2	29.30 (21.84 to 39.31)	0.900 (-9.14 to 8.33)	1.03 (0.77 to 1.38)
Latvia	2021	Mental and behavioural disorders	23,400	12.4	24,200 (20,700 to 28,300)	12.8 (11.0 to 15.0)	-822 (-4,930 to 2,680)	-0.436 (-2.61 to 1.42)	0.97 (0.83 to 1.13)	24.9	29.30 (19.33 to 44.40)	-4.40 (-19.51 to 5.48)	0.85 (0.56 to 1.28)
Latvia	2020	Diseases of the nervous system	7,040	3.7	7,620 (7,060 to 8,220)	4.01 (3.71 to 4.33)	-582 (-1,190 to -13)	-0.306 (-0.624 to -0.007)	0.92 (0.86 to 1.00)	9.4	9.80 (8.41 to 11.42)	-0.400 (-2.03 to 0.971)	0.96 (0.82 to 1.12)
Latvia	2021	Diseases of the nervous system	6,920	3.67	7,180 (6,440 to 8,000)	3.81 (3.42 to 4.25)	-265 (-1,070 to 461)	-0.140 (-0.568 to 0.245)	0.96 (0.87 to 1.07)	8.1	9.80 (7.90 to 12.16)	-1.70 (-4.06 to 0.208)	0.83 (0.67 to 1.03)
Latvia	2020	Diseases of the eye and adnexa	2,770	1.46	3,190 (1,460 to 7,010)	1.68 (0.766 to 3.69)	-426 (-4,330 to 1,340)	-0.224 (-2.28 to 0.702)	0.87 (0.39 to 1.93)	2.6	2.85 (1.89 to 4.30)	-0.247 (-1.75 to 0.727)	0.91 (0.60 to 1.39)
Latvia	2021	Diseases of the eye and adnexa	6,030	3.2	3,170 (1,440 to 6,950)	1.68 (0.766 to 3.69)	2,860 (-881 to 4,560)	1.52 (-0.468 to 2.42)	1.90 (0.87 to 4.09)	1.4	2.85 (1.89 to 4.30)	-1.45 (-2.89 to -0.482)	0.49 (0.33 to 0.74)
Latvia	2020	Diseases of the ear and mastoid process	1,790	0.94	2,220 (1,900 to 2,600)	1.17 (1.00 to 1.37)	-437 (-810 to -118)	-0.230 (-0.426 to -0.062)	0.80 (0.69 to 0.94)	5.5	5.50 (5.16 to 5.86)	0.000 (-0.356 to 0.337)	1.00 (0.94 to 1.07)
Latvia	2021	Diseases of the ear and mastoid process	1,680	0.889	2,210 (1,770 to 2,750)	1.17 (0.940 to 1.46)	-530 (-1,080 to -97)	-0.281 (-0.570 to -0.051)	0.76 (0.61 to 0.95)	5	5.50 (5.03 to 6.01)	-0.500 (-1.02 to -0.027)	0.91 (0.83 to 0.99)
Latvia	2020	Diseases of the circulatory system	54,400	28.6	60,900 (58,700 to 63,200)	32.0 (30.9 to 33.3)	-6,460 (-8,760 to -4,260)	-3.40 (-4.61 to -2.24)	0.89 (0.86 to 0.93)	6.9	6.80 (6.61 to 7.00)	0.101 (-0.102 to 0.287)	1.01 (0.99 to 1.04)
Latvia	2021	Diseases of the circulatory system	49,900	26.5	60,400 (57,300 to 63,600)	32.0 (30.4 to 33.8)	-10,500 (-13,700 to -7,380)	-5.56 (-7.29 to -3.91)	0.83 (0.78 to 0.87)	8.2	6.80 (6.61 to 7.00)	1.40 (1.20 to 1.59)	1.21 (1.17 to 1.24)
Latvia	2020	Diseases of the respiratory system	17,300	9.11	31,400 (27,500 to 35,900)	16.5 (14.4 to 18.9)	-14,100 (-18,600 to -10,100)	-7.42 (-9.80 to -5.34)	0.55 (0.48 to 0.63)	6.2	6.16 (6.01 to 6.31)	0.043 (-0.112 to 0.195)	1.01 (0.98 to 1.03)
Latvia	2021	Diseases of the respiratory system	13,600	7.2	31,200 (27,200 to 35,600)	16.5 (14.4 to 18.9)	-17,600 (-21,900 to -13,600)	-9.33 (-11.6 to -7.22)	0.44 (0.38 to 0.50)	8.9	6.16 (6.01 to 6.31)	2.74 (2.59 to 2.90)	1.45 (1.41 to 1.48)
Latvia	2020	Diseases of the digestive system	23,200	12.2	26,800 (25,700 to 27,900)	14.1 (13.5 to 14.7)	-3,610 (-4,730 to -2,520)	-1.90 (-2.49 to -1.33)	0.87 (0.83 to 0.90)	5.7	5.48 (5.14 to 5.85)	0.217 (-0.139 to 0.563)	1.04 (0.98 to 1.11)
Latvia	2021	Diseases of the digestive system	22,000	11.7	26,600 (25,500 to 27,700)	14.1 (13.5 to 14.7)	-4,590 (-5,710 to -3,490)	-2.44 (-3.03 to -1.85)	0.83 (0.79 to 0.86)	6.5	5.48 (5.14 to 5.85)	1.02 (0.660 to 1.36)	1.19 (1.11 to 1.26)
Latvia	2020	Diseases of the skin and subcutaneous tissue	3,160	1.66	4,120 (3,750 to 4,520)	2.17 (1.97 to 2.38)	-958 (-1,350 to -595)	-0.504 (-0.708 to -0.313)	0.77 (0.70 to 0.84)	9.7	9.60 (8.35 to 11.04)	0.100 (-1.36 to 1.33)	1.01 (0.88 to 1.16)
Latvia	2021	Diseases of the skin and subcutaneous tissue	2,850	1.51	4,080 (3,720 to 4,480)	2.17 (1.97 to 2.38)	-1,230 (-1,630 to -870)	-0.655 (-0.867 to -0.462)	0.70 (0.64 to 0.77)	8.1	9.60 (7.88 to 11.70)	-1.50 (-3.64 to 0.221)	0.84 (0.69 to 1.03)
Latvia	2020	Diseases of musculoskeletal system and connective tissue	15,100	7.92	17,800 (15,700 to 20,200)	9.38 (8.28 to 10.6)	-2,770 (-5,150 to -696)	-1.46 (-2.71 to -0.366)	0.84 (0.75 to 0.96)	8.1	8.37 (7.96 to 8.80)	-0.269 (-0.693 to 0.135)	0.97 (0.92 to 1.02)
Latvia	2021	Diseases of musculoskeletal system and connective tissue	24,100	12.8	17,700 (14,800 to 21,100)	9.38 (7.87 to 11.2)	6,420 (2,940 to 9,320)	3.41 (1.56 to 4.94)	1.36 (1.14 to 1.63)	7.6	8.37 (7.96 to 8.80)	-0.769 (-1.19 to -0.358)	0.91 (0.86 to 0.95)

Location	Year	Condition	Observed hospitalizations	Observed hospitalizations per 1,000 population	Expected hospitalizations	Expected hospitalizations per 1,000 population	Difference in hospitalizations	Difference in rate	Hospitalizations observed-to-expected ratio	Observed length of stay	Expected length of stay	Difference in length of stay	Length of stay observed-to-expected ratio
Latvia	2020	Diseases of the genitourinary system	14,200	7.49	16,000 (14,800 to 17,300)	8.43 (7.79 to 9.13)	-1,790 (-3,120 to -569)	-0.940 (-1.64 to -0.300)	0.89 (0.82 to 0.96)	5.3	5.40 (5.01 to 5.82)	-0.100 (-0.520 to 0.287)	0.98 (0.91 to 1.06)
Latvia	2021	Diseases of the genitourinary system	13,600	7.2	15,900 (14,700 to 17,200)	8.43 (7.79 to 9.13)	-2,320 (-3,630 to -1,080)	-1.23 (-1.93 to -0.571)	0.85 (0.79 to 0.93)	4.8	5.40 (4.86 to 6.00)	-0.600 (-1.20 to -0.050)	0.89 (0.80 to 0.99)
Latvia	2020	Pregnancy, childbirth and the puerperium	23,300	12.2	25,300 (22,800 to 28,000)	13.3 (12.0 to 14.7)	-2,030 (-4,680 to 479)	-1.07 (-2.46 to 0.252)	0.92 (0.83 to 1.02)	3.5	3.60 (3.45 to 3.76)	-0.100 (-0.258 to 0.052)	0.97 (0.93 to 1.02)
Latvia	2021	Pregnancy, childbirth and the puerperium	23,100	12.3	25,100 (21,700 to 28,900)	13.3 (11.5 to 15.4)	-1,930 (-5,830 to 1,390)	-1.02 (-3.10 to 0.740)	0.92 (0.80 to 1.06)	4	3.60 (3.39 to 3.83)	0.400 (0.172 to 0.615)	1.11 (1.04 to 1.18)
Latvia	2020	Certain conditions originating in the perinatal period	2,570	1.35	1,900 (559 to 6,460)	1.00 (0.294 to 3.40)	667 (-3,960 to 2,010)	0.351 (-2.08 to 1.06)	1.35 (0.39 to 4.59)	8.9	10.56 (6.91 to 16.13)	-1.66 (-7.28 to 2.01)	0.84 (0.55 to 1.29)
Latvia	2021	Certain conditions originating in the perinatal period	2,840	1.51	1,880 (554 to 6,410)	1.00 (0.294 to 3.40)	955 (-3,510 to 2,290)	0.507 (-1.86 to 1.22)	1.51 (0.45 to 5.18)	8.6	10.56 (6.91 to 16.13)	-1.96 (-7.40 to 1.70)	0.81 (0.54 to 1.25)
Latvia	2020	Congenital malformations, deformations and chromosomal abnormalities	1,890	0.995	2,030 (1,850 to 2,230)	1.07 (0.976 to 1.17)	-144 (-345 to 38)	-0.076 (-0.181 to 0.020)	0.93 (0.85 to 1.02)	6.8	7.65 (6.97 to 8.40)	-0.850 (-1.61 to -0.177)	0.89 (0.81 to 0.97)
Latvia	2021	Congenital malformations, deformations and chromosomal abnormalities	2,300	1.22	2,020 (1,840 to 2,210)	1.07 (0.976 to 1.17)	283 (85 to 460)	0.150 (0.045 to 0.244)	1.14 (1.04 to 1.25)	5.9	7.65 (6.97 to 8.40)	-1.75 (-2.50 to -1.07)	0.77 (0.70 to 0.85)
Latvia	2020	Symptoms, signs and abnormal clinical and laboratory findings, n.e.c.	806	0.424	1,060 (901 to 1,250)	0.558 (0.474 to 0.655)	-254 (-439 to -99)	-0.133 (-0.231 to -0.052)	0.76 (0.65 to 0.89)	5	4.51 (4.13 to 4.93)	0.490 (0.077 to 0.870)	1.11 (1.02 to 1.21)
Latvia	2021	Symptoms, signs and abnormal clinical and laboratory findings, n.e.c.	1,080	0.574	1,050 (836 to 1,320)	0.558 (0.443 to 0.701)	31 (-237 to 247)	0.017 (-0.126 to 0.131)	1.03 (0.82 to 1.30)	5.4	4.51 (4.13 to 4.93)	0.890 (0.469 to 1.28)	1.20 (1.10 to 1.31)
Latvia	2020	Injury, poisoning and other consequences of external causes	24,400	12.8	28,000 (26,000 to 30,100)	14.7 (13.7 to 15.8)	-3,580 (-5,720 to -1,580)	-1.88 (-3.01 to -0.833)	0.87 (0.81 to 0.94)	6.8	6.77 (6.56 to 6.99)	0.029 (-0.194 to 0.243)	1.00 (0.97 to 1.04)
Latvia	2021	Injury, poisoning and other consequences of external causes	24,900	13.2	27,700 (25,800 to 29,900)	14.7 (13.7 to 15.8)	-2,880 (-4,980 to -949)	-1.53 (-2.64 to -0.503)	0.90 (0.83 to 0.96)	6.4	6.77 (6.56 to 6.99)	-0.371 (-0.591 to -0.158)	0.95 (0.92 to 0.98)
Latvia	2020	Factors influencing health status and contact with health services	857	0.451	1,240 (751 to 2,060)	0.655 (0.395 to 1.09)	-388 (-1,200 to 112)	-0.204 (-0.631 to 0.059)	0.69 (0.42 to 1.15)	3.5	4.70 (2.87 to 7.68)	-1.20 (-4.32 to 0.603)	0.74 (0.45 to 1.21)
Mexico	2020	All causes	3,370,000	26.4	5,050,000 (4,710,000 to 5,410,000)	39.5 (36.9 to 42.3)	-1,670,000 (-2,030,000 to -1,340,000)	-13.1 (-15.9 to -10.5)	0.67 (0.62 to 0.72)				
Mexico	2021	All causes	3,670,000	28.5	5,100,000 (4,620,000 to 5,620,000)	39.5 (35.8 to 43.6)	-1,430,000 (-1,940,000 to -945,000)	-11.1 (-15.1 to -7.33)	0.72 (0.65 to 0.80)				

Location	Year	Condition	Observed hospitalizations	Observed hospitalizations per 1,000 population	Expected hospitalizations	Expected hospitalizations per 1,000 population	Difference in hospitalizations	Difference in rate	Hospitalizations observed-to-expected ratio	Observed length of stay	Expected length of stay	Difference in length of stay	Length of stay observed-to-expected ratio
Mexico	2020	Infectious and parasitic diseases	54,700	0.428	121,000 (89,000 to 166,000)	0.950 (0.696 to 1.30)	-66,600 (-111,000 to -34,300)	-0.521 (-0.867 to -0.268)	0.45 (0.33 to 0.61)	6.7	5.30 (4.50 to 6.24)	1.40 (0.444 to 2.21)	1.26 (1.07 to 1.49)
Mexico	2021	Infectious and parasitic diseases	53,900	0.418	122,000 (79,000 to 190,000)	0.950 (0.612 to 1.47)	-68,600 (-136,000 to -24,000)	-0.532 (-1.06 to -0.186)	0.44 (0.28 to 0.69)	7.2	5.30 (4.20 to 6.68)	1.90 (0.514 to 3.01)	1.36 (1.08 to 1.72)
Mexico	2020	Neoplasms	195,000	1.52	316,000 (297,000 to 336,000)	2.47 (2.33 to 2.63)	-121,000 (-142,000 to -103,000)	-0.951 (-1.11 to -0.808)	0.62 (0.58 to 0.65)	5.7	5.17 (4.71 to 5.68)	0.525 (0.028 to 0.991)	1.10 (1.00 to 1.21)
Mexico	2021	Neoplasms	239,000	1.86	319,000 (293,000 to 348,000)	2.47 (2.27 to 2.70)	-79,800 (-108,000 to -53,200)	-0.619 (-0.839 to -0.412)	0.75 (0.69 to 0.82)	5.7	5.17 (4.71 to 5.68)	0.525 (0.029 to 0.979)	1.10 (1.01 to 1.21)
Mexico	2020	Diseases of the blood and bloodforming organs	26,200	0.205	39,600 (35,700 to 43,900)	0.310 (0.279 to 0.343)	-13,300 (-17,700 to -9,520)	-0.104 (-0.138 to -0.074)	0.66 (0.60 to 0.73)	6.4	5.52 (5.22 to 5.83)	0.882 (0.572 to 1.17)	1.16 (1.10 to 1.22)
Mexico	2021	Diseases of the blood and bloodforming organs	28,700	0.222	39,900 (36,000 to 44,300)	0.310 (0.279 to 0.343)	-11,300 (-15,700 to -7,290)	-0.087 (-0.122 to -0.057)	0.72 (0.65 to 0.80)	6.9	5.52 (5.22 to 5.83)	1.38 (1.07 to 1.67)	1.25 (1.18 to 1.32)
Mexico	2020	Endocrine, nutritional and metabolic diseases	110,000	0.862	170,000 (159,000 to 182,000)	1.33 (1.25 to 1.43)	-60,100 (-72,000 to -49,000)	-0.470 (-0.563 to -0.383)	0.65 (0.60 to 0.69)	5.9	5.47 (4.96 to 6.04)	0.426 (-0.136 to 0.935)	1.08 (0.98 to 1.19)
Mexico	2021	Endocrine, nutritional and metabolic diseases	117,000	0.905	167,000 (152,000 to 184,000)	1.30 (1.18 to 1.43)	-50,700 (-67,700 to -35,700)	-0.393 (-0.525 to -0.277)	0.70 (0.63 to 0.77)	6	5.47 (4.96 to 6.04)	0.526 (-0.032 to 1.04)	1.10 (0.99 to 1.21)
Mexico	2020	Mental and behavioural disorders	33,700	0.264	49,300 (47,100 to 51,500)	0.385 (0.369 to 0.403)	-15,600 (-17,800 to -13,400)	-0.122 (-0.139 to -0.105)	0.68 (0.65 to 0.72)	36.1	26.46 (21.62 to 32.37)	9.64 (3.73 to 14.51)	1.36 (1.12 to 1.67)
Mexico	2021	Mental and behavioural disorders	34,100	0.264	49,700 (46,700 to 53,000)	0.385 (0.362 to 0.411)	-15,600 (-18,800 to -12,600)	-0.121 (-0.146 to -0.098)	0.69 (0.64 to 0.73)	26.9	26.46 (21.62 to 32.37)	0.444 (-5.44 to 5.30)	1.02 (0.83 to 1.25)
Mexico	2020	Diseases of the nervous system	37,400	0.293	59,400 (54,600 to 64,500)	0.465 (0.428 to 0.505)	-22,000 (-27,100 to -17,200)	-0.172 (-0.212 to -0.135)	0.63 (0.58 to 0.68)	8.1	7.40 (6.84 to 8.01)	0.700 (0.106 to 1.25)	1.09 (1.01 to 1.18)
Mexico	2021	Diseases of the nervous system	42,900	0.332	59,900 (53,300 to 67,400)	0.465 (0.413 to 0.522)	-17,100 (-24,700 to -10,500)	-0.132 (-0.191 to -0.082)	0.72 (0.63 to 0.80)	7.8	7.40 (6.62 to 8.27)	0.400 (-0.469 to 1.17)	1.05 (0.94 to 1.18)
Mexico	2020	Diseases of the eye and adnexa	8,370	0.065	25,700 (21,700 to 30,500)	0.201 (0.170 to 0.238)	-17,300 (-22,000 to -13,300)	-0.136 (-0.172 to -0.104)	0.33 (0.28 to 0.39)	4	2.60 (2.12 to 3.19)	1.40 (0.810 to 1.89)	1.54 (1.25 to 1.89)
Mexico	2021	Diseases of the eye and adnexa	8,770	0.068	25,900 (20,400 to 33,000)	0.201 (0.158 to 0.256)	-17,200 (-24,300 to -11,600)	-0.133 (-0.188 to -0.090)	0.34 (0.27 to 0.43)	4.2	2.60 (1.95 to 3.47)	1.60 (0.731 to 2.25)	1.62 (1.21 to 2.15)
Mexico	2020	Diseases of the ear and mastoid process	2,920	0.023	6,850 (6,180 to 7,600)	0.054 (0.048 to 0.059)	-3,930 (-4,680 to -3,270)	-0.031 (-0.037 to -0.026)	0.43 (0.38 to 0.47)	3.1	2.90 (2.53 to 3.33)	0.197 (-0.246 to 0.562)	1.07 (0.93 to 1.22)
Mexico	2021	Diseases of the ear and mastoid process	3,000	0.023	6,540 (5,660 to 7,560)	0.051 (0.044 to 0.059)	-3,540 (-4,580 to -2,660)	-0.027 (-0.035 to -0.021)	0.46 (0.40 to 0.53)	3.2	2.90 (2.53 to 3.33)	0.297 (-0.132 to 0.672)	1.10 (0.96 to 1.27)
Mexico	2020	Diseases of the circulatory system	153,000	1.2	255,000 (239,000 to 272,000)	1.99 (1.87 to 2.13)	-102,000 (-119,000 to -86,100)	-0.796 (-0.934 to -0.674)	0.60 (0.56 to 0.64)	6.4	5.89 (5.40 to 6.44)	0.505 (-0.023 to 1.01)	1.09 (1.00 to 1.19)
Mexico	2021	Diseases of the circulatory system	181,000	1.4	257,000 (234,000 to 282,000)	1.99 (1.82 to 2.19)	-76,000 (-101,000 to -53,100)	-0.589 (-0.780 to -0.412)	0.70 (0.64 to 0.77)	6.6	5.89 (5.40 to 6.44)	0.705 (0.159 to 1.19)	1.12 (1.02 to 1.22)

Location	Year	Condition	Observed hospitalizations	Observed hospitalizations per 1,000 population	Expected hospitalizations	Expected hospitalizations per 1,000 population	Difference in hospitalizations	Difference in rate	Hospitalizations observed-to-expected ratio	Observed length of stay	Expected length of stay	Difference in length of stay	Length of stay observed-to-expected ratio
Mexico	2020	Diseases of the respiratory system	202,000	1.58	248,000 (218,000 to 281,000)	1.94 (1.70 to 2.20)	-45,500 (-78,700 to -15,500)	-0.356 (-0.616 to -0.121)	0.82 (0.72 to 0.93)	7.3	6.00 (5.45 to 6.60)	1.30 (0.692 to 1.85)	1.22 (1.10 to 1.34)
Mexico	2021	Diseases of the respiratory system	175,000	1.36	250,000 (208,000 to 299,000)	1.94 (1.62 to 2.32)	-74,500 (-124,000 to -32,500)	-0.578 (-0.960 to -0.252)	0.70 (0.59 to 0.84)	7.9	6.00 (5.24 to 6.87)	1.90 (1.04 to 2.67)	1.32 (1.15 to 1.51)
Mexico	2020	Diseases of the digestive system	331,000	2.59	610,000 (569,000 to 654,000)	4.78 (4.46 to 5.12)	-280,000 (-324,000 to -239,000)	-2.19 (-2.53 to -1.87)	0.54 (0.51 to 0.58)	4.6	3.99 (3.70 to 4.30)	0.613 (0.298 to 0.895)	1.15 (1.07 to 1.24)
Mexico	2021	Diseases of the digestive system	425,000	3.29	616,000 (558,000 to 679,000)	4.78 (4.33 to 5.27)	-191,000 (-257,000 to -133,000)	-1.48 (-1.99 to -1.03)	0.69 (0.62 to 0.76)	4.4	3.99 (3.70 to 4.30)	0.413 (0.095 to 0.700)	1.10 (1.02 to 1.19)
Mexico	2020	Diseases of the skin and subcutaneous tissue	30,200	0.236	59,500 (53,800 to 65,700)	0.465 (0.421 to 0.514)	-29,300 (-35,400 to -23,600)	-0.229 (-0.277 to -0.185)	0.51 (0.46 to 0.56)	6.3	6.00 (5.30 to 6.79)	0.300 (-0.490 to 1.00)	1.05 (0.93 to 1.19)
Mexico	2021	Diseases of the skin and subcutaneous tissue	34,100	0.264	60,000 (52,200 to 69,100)	0.465 (0.404 to 0.536)	-26,000 (-35,000 to -18,000)	-0.201 (-0.272 to -0.140)	0.57 (0.49 to 0.65)	6.7	6.00 (5.04 to 7.14)	0.700 (-0.419 to 1.66)	1.12 (0.94 to 1.33)
Mexico	2020	Diseases of musculoskeletal system and connective tissue	47,300	0.37	100,000 (94,200 to 107,000)	0.786 (0.737 to 0.837)	-53,000 (-59,800 to -47,000)	-0.415 (-0.468 to -0.367)	0.47 (0.44 to 0.50)	5.1	4.48 (4.05 to 4.97)	0.615 (0.129 to 1.05)	1.14 (1.03 to 1.26)
Mexico	2021	Diseases of musculoskeletal system and connective tissue	66,300	0.514	98,900 (90,400 to 108,000)	0.767 (0.701 to 0.839)	-32,600 (-41,900 to -24,200)	-0.253 (-0.325 to -0.188)	0.67 (0.61 to 0.73)	4.8	4.48 (4.05 to 4.97)	0.315 (-0.157 to 0.746)	1.07 (0.97 to 1.18)
Mexico	2020	Diseases of the genitourinary system	235,000	1.84	433,000 (408,000 to 459,000)	3.39 (3.19 to 3.59)	-198,000 (-224,000 to -174,000)	-1.55 (-1.75 to -1.36)	0.54 (0.51 to 0.57)	4.7	4.30 (3.84 to 4.82)	0.400 (-0.129 to 0.866)	1.09 (0.97 to 1.23)
Mexico	2021	Diseases of the genitourinary system	281,000	2.18	437,000 (402,000 to 474,000)	3.39 (3.12 to 3.68)	-156,000 (-194,000 to -122,000)	-1.21 (-1.50 to -0.944)	0.64 (0.59 to 0.70)	4.6	4.30 (3.66 to 5.05)	0.300 (-0.450 to 0.949)	1.07 (0.91 to 1.26)
Mexico	2020	Pregnancy, childbirth and the puerperium	1,250,000	9.81	1,600,000 (1,480,000 to 1,730,000)	12.5 (11.6 to 13.5)	-345,000 (-477,000 to -225,000)	-2.70 (-3.74 to -1.76)	0.78 (0.72 to 0.85)	1.8	1.88 (1.76 to 2.01)	-0.079 (-0.203 to 0.039)	0.96 (0.90 to 1.02)
Mexico	2021	Pregnancy, childbirth and the puerperium	1,230,000	9.53	1,570,000 (1,410,000 to 1,750,000)	12.2 (10.9 to 13.6)	-341,000 (-524,000 to -180,000)	-2.64 (-4.06 to -1.39)	0.78 (0.70 to 0.87)	1.8	1.88 (1.76 to 2.01)	-0.079 (-0.210 to 0.041)	0.96 (0.90 to 1.02)
Mexico	2020	Certain conditions originating in the perinatal period	160,000	1.25	175,000 (162,000 to 189,000)	1.37 (1.27 to 1.48)	-14,800 (-28,900 to -1,790)	-0.116 (-0.226 to -0.014)	0.92 (0.85 to 0.99)	9.3	8.87 (8.48 to 9.28)	0.429 (0.017 to 0.817)	1.05 (1.00 to 1.10)
Mexico	2021	Certain conditions originating in the perinatal period	163,000	1.26	171,000 (154,000 to 191,000)	1.33 (1.19 to 1.48)	-8,830 (-28,800 to 8,680)	-0.068 (-0.223 to 0.067)	0.95 (0.85 to 1.06)	9.5	9.05 (8.49 to 9.64)	0.454 (-0.148 to 1.01)	1.05 (0.98 to 1.12)
Mexico	2020	Congenital malformations, deformations and chromosomal abnormalities	34,700	0.271	48,900 (45,600 to 52,300)	0.382 (0.357 to 0.409)	-14,200 (-17,600 to -11,000)	-0.111 (-0.138 to -0.086)	0.71 (0.66 to 0.76)	8.1	6.80 (6.26 to 7.39)	1.30 (0.718 to 1.85)	1.19 (1.10 to 1.30)
Mexico	2021	Congenital malformations, deformations and chromosomal abnormalities	39,000	0.302	47,500 (43,100 to 52,300)	0.368 (0.334 to 0.406)	-8,530 (-13,300 to -4,290)	-0.066 (-0.103 to -0.033)	0.82 (0.75 to 0.90)	8.4	6.80 (6.05 to 7.65)	1.60 (0.754 to 2.34)	1.24 (1.10 to 1.39)

Location	Year	Condition	Observed hospitalizations	Observed hospitalizations per 1,000 population	Expected hospitalizations	Expected hospitalizations per 1,000 population	Difference in hospitalizations	Difference in rate	Hospitalizations observed-to-expected ratio	Observed length of stay	Expected length of stay	Difference in length of stay	Length of stay observed-to-expected ratio
Mexico	2020	Symptoms, signs and abnormal clinical and laboratory findings, n.e.c.	79,900	0.625	111,000 (85,200 to 145,000)	0.869 (0.667 to 1.13)	-31,100 (-64,300 to -5,350)	-0.244 (-0.503 to -0.042)	0.72 (0.55 to 0.94)	6.5	6.00 (4.89 to 7.36)	0.500 (-0.879 to 1.62)	1.08 (0.88 to 1.33)
Mexico	2021	Symptoms, signs and abnormal clinical and laboratory findings, n.e.c.	88,600	0.687	112,000 (77,000 to 163,000)	0.869 (0.597 to 1.26)	-23,400 (-75,200 to 11,500)	-0.181 (-0.583 to 0.090)	0.79 (0.54 to 1.15)	7.1	6.00 (4.49 to 8.01)	1.10 (-0.950 to 2.63)	1.18 (0.88 to 1.59)
Mexico	2020	Injury, poisoning and other consequences of external causes	295,000	2.31	435,000 (416,000 to 455,000)	3.40 (3.25 to 3.56)	-140,000 (-160,000 to -121,000)	-1.10 (-1.25 to -0.948)	0.68 (0.65 to 0.71)	5.8	6.20 (5.73 to 6.71)	-0.400 (-0.910 to 0.081)	0.94 (0.86 to 1.01)
Mexico	2021	Injury, poisoning and other consequences of external causes	350,000	2.72	439,000 (419,000 to 459,000)	3.40 (3.25 to 3.56)	-88,700 (-109,000 to -68,800)	-0.688 (-0.845 to -0.533)	0.80 (0.76 to 0.84)	6.1	6.20 (5.54 to 6.94)	-0.100 (-0.842 to 0.558)	0.98 (0.88 to 1.10)
Mexico	2020	Factors influencing health status and contact with health services	85,800	0.672	140,000 (122,000 to 161,000)	1.10 (0.956 to 1.26)	-54,600 (-74,800 to -36,100)	-0.427 (-0.585 to -0.282)	0.61 (0.53 to 0.70)	3.6	3.20 (2.87 to 3.57)	0.400 (0.026 to 0.737)	1.12 (1.01 to 1.26)
Mexico	2021	Factors influencing health status and contact with health services	110,000	0.853	142,000 (116,000 to 172,000)	1.10 (0.902 to 1.34)	-31,600 (-61,800 to -6,860)	-0.245 (-0.479 to -0.053)	0.78 (0.64 to 0.94)	3.5	3.20 (2.74 to 3.74)	0.300 (-0.216 to 0.759)	1.09 (0.94 to 1.28)
Netherlands	2020	All causes	1,300,000	74.5	1,460,000 (1,320,000 to 1,630,000)	84.0 (75.4 to 93.6)	-166,000 (-334,000 to -19,100)	-9.54 (-19.1 to -1.09)	0.89 (0.80 to 0.99)				
Netherlands	2021	All causes	1,310,000	74.5	1,410,000 (1,210,000 to 1,650,000)	80.6 (69.2 to 93.8)	-107,000 (-337,000 to 93,500)	-6.10 (-19.2 to 5.33)	0.92 (0.79 to 1.08)				
Netherlands	2020	Infectious and parasitic diseases	23,900	1.37	33,700 (29,700 to 38,300)	1.93 (1.70 to 2.20)	-9,840 (-14,400 to -5,840)	-0.564 (-0.825 to -0.335)	0.71 (0.62 to 0.80)	7.1	6.60 (6.03 to 7.22)	0.500 (-0.125 to 1.09)	1.08 (0.98 to 1.18)
Netherlands	2021	Infectious and parasitic diseases	25,800	1.47	33,900 (28,300 to 40,600)	1.93 (1.61 to 2.32)	-8,120 (-14,900 to -2,570)	-0.463 (-0.852 to -0.146)	0.76 (0.63 to 0.91)	6.6	6.60 (5.81 to 7.50)	0.000 (-0.907 to 0.776)	1.00 (0.88 to 1.13)
Netherlands	2020	Neoplasms	146,000	8.4	165,000 (156,000 to 174,000)	9.46 (8.95 to 10.0)	-18,600 (-28,100 to -9,630)	-1.06 (-1.61 to -0.552)	0.89 (0.84 to 0.94)	4.9	4.71 (4.38 to 5.06)	0.190 (-0.162 to 0.519)	1.04 (0.97 to 1.12)
Netherlands	2021	Neoplasms	145,000	8.28	166,000 (148,000 to 186,000)	9.46 (8.44 to 10.6)	-20,700 (-40,700 to -2,790)	-1.18 (-2.32 to -0.159)	0.87 (0.78 to 0.98)	4.8	4.53 (4.09 to 5.02)	0.273 (-0.217 to 0.708)	1.06 (0.96 to 1.17)
Netherlands	2020	Diseases of the blood and bloodforming organs	15,700	0.9	17,600 (16,100 to 19,200)	1.01 (0.925 to 1.10)	-1,910 (-3,480 to -439)	-0.109 (-0.199 to -0.025)	0.89 (0.82 to 0.97)	3.8	3.76 (3.44 to 4.12)	0.038 (-0.312 to 0.358)	1.01 (0.92 to 1.10)
Netherlands	2021	Diseases of the blood and bloodforming organs	16,100	0.921	17,700 (15,600 to 20,000)	1.01 (0.892 to 1.14)	-1,560 (-3,960 to 478)	-0.089 (-0.226 to 0.027)	0.91 (0.80 to 1.03)	3.8	3.63 (3.19 to 4.12)	0.172 (-0.318 to 0.612)	1.05 (0.92 to 1.19)
Netherlands	2020	Endocrine, nutritional and metabolic diseases	31,300	1.79	38,700 (36,900 to 40,500)	2.22 (2.12 to 2.32)	-7,430 (-9,310 to -5,640)	-0.426 (-0.534 to -0.323)	0.81 (0.77 to 0.85)	4.6	4.06 (3.61 to 4.57)	0.539 (0.032 to 0.988)	1.13 (1.01 to 1.27)
Netherlands	2021	Endocrine, nutritional and metabolic diseases	32,100	1.83	38,900 (37,100 to 40,800)	2.22 (2.12 to 2.32)	-6,780 (-8,640 to -5,010)	-0.386 (-0.493 to -0.286)	0.83 (0.79 to 0.87)	4.4	3.83 (3.25 to 4.53)	0.565 (-0.126 to 1.16)	1.15 (0.97 to 1.36)

Location	Year	Condition	Observed hospitalizations	Observed hospitalizations per 1,000 population	Expected hospitalizations	Expected hospitalizations per 1,000 population	Difference in hospitalizations	Difference in rate	Hospitalizations observed-to-expected ratio	Observed length of stay	Expected length of stay	Difference in length of stay	Length of stay observed-to-expected ratio
Netherlands	2020	Mental and behavioural disorders	6,870	0.394	7,180 (5,820 to 8,840)	0.411 (0.334 to 0.507)	-309 (-1,990 to 1,040)	-0.018 (-0.114 to 0.059)	0.96 (0.78 to 1.18)	9.4	9.50 (6.93 to 13.02)	-0.100 (-3.53 to 2.47)	0.99 (0.73 to 1.36)
Netherlands	2021	Mental and behavioural disorders	7,030	0.401	6,460 (4,810 to 8,680)	0.369 (0.274 to 0.495)	564 (-1,620 to 2,190)	0.032 (-0.092 to 0.125)	1.09 (0.81 to 1.45)	9.5	9.50 (6.09 to 14.83)	0.000 (-5.32 to 3.44)	1.00 (0.64 to 1.57)
Netherlands	2020	Diseases of the nervous system	32,400	1.86	40,400 (37,100 to 44,000)	2.32 (2.13 to 2.52)	-7,960 (-11,600 to -4,650)	-0.456 (-0.664 to -0.267)	0.80 (0.74 to 0.87)	3.9	3.70 (2.56 to 5.34)	0.200 (-1.47 to 1.33)	1.05 (0.73 to 1.52)
Netherlands	2021	Diseases of the nervous system	30,300	1.73	38,000 (33,700 to 42,900)	2.17 (1.92 to 2.45)	-7,700 (-12,600 to -3,400)	-0.439 (-0.716 to -0.194)	0.80 (0.71 to 0.90)	4.1	3.70 (2.20 to 6.22)	0.400 (-2.15 to 1.91)	1.11 (0.66 to 1.87)
Netherlands	2020	Diseases of the eye and adnexa	3,000	0.172	3,570 (3,000 to 4,240)	0.205 (0.172 to 0.243)	-574 (-1,250 to -2)	-0.033 (-0.072 to -0.000)	0.84 (0.71 to 1.00)	2.5	2.50 (2.07 to 3.01)	0.000 (-0.514 to 0.428)	1.00 (0.83 to 1.21)
Netherlands	2021	Diseases of the eye and adnexa	2,700	0.154	3,290 (2,570 to 4,200)	0.187 (0.147 to 0.240)	-583 (-1,510 to 120)	-0.033 (-0.086 to 0.007)	0.82 (0.64 to 1.05)	2.5	2.50 (1.92 to 3.25)	0.000 (-0.762 to 0.582)	1.00 (0.77 to 1.30)
Netherlands	2020	Diseases of the ear and mastoid process	6,320	0.363	8,130 (7,060 to 9,350)	0.466 (0.405 to 0.536)	-1,800 (-3,050 to -751)	-0.103 (-0.175 to -0.043)	0.78 (0.67 to 0.89)	2.1	2.00 (1.56 to 2.57)	0.100 (-0.470 to 0.544)	1.05 (0.82 to 1.35)
Netherlands	2021	Diseases of the ear and mastoid process	6,010	0.343	8,170 (6,700 to 9,970)	0.466 (0.382 to 0.568)	-2,160 (-3,970 to -679)	-0.123 (-0.226 to -0.039)	0.74 (0.60 to 0.90)	2	2.00 (1.41 to 2.84)	0.000 (-0.850 to 0.581)	1.00 (0.70 to 1.41)
Netherlands	2020	Diseases of the circulatory system	204,000	11.7	224,000 (197,000 to 255,000)	12.8 (11.3 to 14.6)	-19,800 (-51,200 to 7,150)	-1.14 (-2.93 to 0.410)	0.91 (0.80 to 1.04)	5.4	5.68 (5.40 to 5.97)	-0.279 (-0.579 to 0.003)	0.95 (0.90 to 1.00)
Netherlands	2021	Diseases of the circulatory system	206,000	11.7	225,000 (187,000 to 270,000)	12.8 (10.7 to 15.4)	-19,500 (-65,500 to 17,800)	-1.11 (-3.73 to 1.02)	0.91 (0.76 to 1.09)	5.3	5.70 (5.26 to 6.17)	-0.397 (-0.861 to 0.041)	0.93 (0.86 to 1.01)
Netherlands	2020	Diseases of the respiratory system	81,200	4.65	139,000 (127,000 to 151,000)	7.95 (7.28 to 8.68)	-57,500 (-70,000 to -46,000)	-3.30 (-4.01 to -2.64)	0.59 (0.54 to 0.64)	5.6	5.60 (5.17 to 6.07)	0.000 (-0.470 to 0.437)	1.00 (0.92 to 1.08)
Netherlands	2021	Diseases of the respiratory system	78,700	4.49	139,000 (128,000 to 152,000)	7.95 (7.28 to 8.68)	-60,800 (-73,500 to -49,200)	-3.47 (-4.19 to -2.81)	0.56 (0.52 to 0.62)	5.2	5.60 (5.00 to 6.28)	-0.400 (-1.08 to 0.212)	0.93 (0.83 to 1.04)
Netherlands	2020	Diseases of the digestive system	129,000	7.38	142,000 (134,000 to 151,000)	8.16 (7.71 to 8.63)	-13,600 (-21,800 to -5,600)	-0.778 (-1.25 to -0.321)	0.90 (0.86 to 0.96)	4.3	4.30 (3.83 to 4.83)	0.000 (-0.524 to 0.476)	1.00 (0.89 to 1.12)
Netherlands	2021	Diseases of the digestive system	129,000	7.37	140,000 (129,000 to 152,000)	7.99 (7.38 to 8.65)	-10,900 (-22,400 to -299)	-0.620 (-1.28 to -0.017)	0.92 (0.85 to 1.00)	4.3	4.30 (3.65 to 5.07)	0.000 (-0.756 to 0.646)	1.00 (0.85 to 1.18)
Netherlands	2020	Diseases of the skin and subcutaneous tissue	11,300	0.646	14,000 (12,600 to 15,600)	0.802 (0.721 to 0.892)	-2,720 (-4,310 to -1,340)	-0.156 (-0.247 to -0.077)	0.81 (0.72 to 0.89)	4.9	4.63 (4.27 to 5.01)	0.275 (-0.110 to 0.627)	1.06 (0.98 to 1.15)
Netherlands	2021	Diseases of the skin and subcutaneous tissue	11,000	0.625	14,100 (12,100 to 16,300)	0.802 (0.691 to 0.932)	-3,110 (-5,370 to -1,170)	-0.177 (-0.306 to -0.067)	0.78 (0.67 to 0.90)	4.9	4.46 (3.98 to 4.99)	0.443 (-0.085 to 0.908)	1.10 (0.98 to 1.23)
Netherlands	2020	Diseases of musculoskeletal system and connective tissue	87,200	5	111,000 (104,000 to 119,000)	6.37 (5.94 to 6.82)	-23,800 (-31,900 to -16,200)	-1.37 (-1.83 to -0.930)	0.79 (0.73 to 0.84)	3	2.82 (2.44 to 3.26)	0.178 (-0.254 to 0.555)	1.06 (0.92 to 1.23)
Netherlands	2021	Diseases of musculoskeletal system and connective tissue	84,800	4.83	108,000 (98,100 to 119,000)	6.17 (5.59 to 6.80)	-23,400 (-34,500 to -13,400)	-1.34 (-1.97 to -0.764)	0.78 (0.71 to 0.86)	2.9	2.65 (2.17 to 3.25)	0.245 (-0.335 to 0.743)	1.09 (0.90 to 1.34)

Location	Year	Condition	Observed hospitalizations	Observed hospitalizations per 1,000 population	Expected hospitalizations	Expected hospitalizations per 1,000 population	Difference in hospitalizations	Difference in rate	Hospitalizations observed-to-expected ratio	Observed length of stay	Expected length of stay	Difference in length of stay	Length of stay observed-to-expected ratio
Netherlands	2020	Diseases of the genitourinary system	79,700	4.57	96,400 (89,200 to 104,000)	5.53 (5.12 to 5.97)	-16,700 (-24,400 to -9,600)	-0.955 (-1.40 to -0.550)	0.83 (0.77 to 0.89)	3.8	3.58 (3.24 to 3.95)	0.222 (-0.140 to 0.559)	1.06 (0.96 to 1.17)
Netherlands	2021	Diseases of the genitourinary system	77,700	4.43	96,900 (86,800 to 108,000)	5.53 (4.95 to 6.17)	-19,200 (-30,300 to -9,070)	-1.09 (-1.73 to -0.517)	0.80 (0.72 to 0.90)	3.6	3.46 (3.01 to 3.98)	0.139 (-0.388 to 0.568)	1.04 (0.90 to 1.19)
Netherlands	2020	Pregnancy, childbirth and the puerperium	116,000	6.64	121,000 (99,700 to 147,000)	6.93 (5.71 to 8.41)	-5,120 (-30,800 to 16,200)	-0.293 (-1.77 to 0.931)	0.96 (0.79 to 1.16)	2.5	2.54 (2.40 to 2.69)	-0.041 (-0.194 to 0.100)	0.98 (0.93 to 1.04)
Netherlands	2021	Pregnancy, childbirth and the puerperium	124,000	7.1	122,000 (92,500 to 160,000)	6.93 (5.27 to 9.12)	2,900 (-35,300 to 32,600)	0.166 (-2.01 to 1.86)	1.02 (0.78 to 1.36)	2.5	2.48 (2.29 to 2.69)	0.017 (-0.198 to 0.211)	1.01 (0.93 to 1.09)
Netherlands	2020	Certain conditions originating in the perinatal period	80,200	4.6	81,200 (77,500 to 85,100)	4.65 (4.44 to 4.88)	-950 (-4,770 to 2,820)	-0.054 (-0.274 to 0.162)	0.99 (0.94 to 1.04)	4.3	4.16 (3.93 to 4.40)	0.141 (-0.107 to 0.365)	1.03 (0.98 to 1.09)
Netherlands	2021	Certain conditions originating in the perinatal period	86,700	4.94	81,600 (76,400 to 87,200)	4.65 (4.36 to 4.97)	5,090 (-484 to 10,200)	0.290 (-0.028 to 0.582)	1.06 (0.99 to 1.13)	4.1	4.02 (3.71 to 4.36)	0.077 (-0.265 to 0.393)	1.02 (0.94 to 1.11)
Netherlands	2020	Congenital malformations, deformations and chromosomal abnormalities	8,390	0.481	8,780 (7,920 to 9,730)	0.503 (0.454 to 0.558)	-382 (-1,340 to 460)	-0.022 (-0.077 to 0.026)	0.96 (0.86 to 1.06)	5.9	5.94 (5.47 to 6.44)	-0.035 (-0.539 to 0.431)	0.99 (0.92 to 1.08)
Netherlands	2021	Congenital malformations, deformations and chromosomal abnormalities	8,160	0.465	8,390 (7,250 to 9,700)	0.479 (0.414 to 0.553)	-229 (-1,580 to 908)	-0.013 (-0.090 to 0.052)	0.97 (0.84 to 1.13)	5.7	5.94 (5.47 to 6.44)	-0.235 (-0.746 to 0.224)	0.96 (0.88 to 1.04)
Netherlands	2020	Symptoms, signs and abnormal clinical and laboratory findings, n.e.c.	61,500	3.52	68,400 (50,500 to 92,800)	3.92 (2.90 to 5.32)	-6,980 (-31,300 to 11,100)	-0.401 (-1.79 to 0.639)	0.90 (0.66 to 1.22)	3	3.10 (2.83 to 3.39)	-0.100 (-0.387 to 0.162)	0.97 (0.89 to 1.06)
Netherlands	2021	Symptoms, signs and abnormal clinical and laboratory findings, n.e.c.	58,800	3.35	61,300 (39,900 to 94,200)	3.50 (2.27 to 5.37)	-2,540 (-36,200 to 18,900)	-0.145 (-2.07 to 1.08)	0.96 (0.62 to 1.47)	3.1	3.10 (2.73 to 3.52)	0.000 (-0.418 to 0.373)	1.00 (0.88 to 1.14)
Netherlands	2020	Injury, poisoning and other consequences of external causes	130,000	7.48	146,000 (130,000 to 165,000)	8.39 (7.47 to 9.43)	-15,900 (-34,100 to 316)	-0.910 (-1.95 to 0.018)	0.89 (0.79 to 1.00)	5.2	5.30 (4.98 to 5.64)	-0.100 (-0.439 to 0.218)	0.98 (0.92 to 1.04)
Netherlands	2021	Injury, poisoning and other consequences of external causes	132,000	7.54	147,000 (125,000 to 174,000)	8.39 (7.11 to 9.90)	-14,900 (-41,300 to 7,520)	-0.849 (-2.35 to 0.429)	0.90 (0.76 to 1.06)	5.1	5.30 (4.62 to 6.08)	-0.200 (-0.970 to 0.473)	0.96 (0.84 to 1.10)
Netherlands	2020	Factors influencing health status and contact with health services	44,100	2.53	52,200 (29,900 to 91,200)	2.99 (1.72 to 5.23)	-8,080 (-47,200 to 14,000)	-0.463 (-2.71 to 0.803)	0.85 (0.48 to 1.46)	2.7	2.80 (2.47 to 3.17)	-0.100 (-0.473 to 0.226)	0.96 (0.85 to 1.09)
Netherlands	2021	Factors influencing health status and contact with health services	43,100	2.46	52,500 (23,900 to 115,000)	2.99 (1.36 to 6.58)	-9,420 (-71,300 to 18,900)	-0.538 (-4.07 to 1.08)	0.82 (0.38 to 1.78)	2.6	2.80 (2.35 to 3.34)	-0.200 (-0.747 to 0.236)	0.93 (0.78 to 1.10)
Norway	2020	All causes	796,000	148	867,000 (850,000 to 883,000)	161 (158 to 164)	-70,400 (-87,100 to -54,400)	-13.1 (-16.2 to -10.1)	0.92 (0.90 to 0.94)				

Location	Year	Condition	Observed hospitalizations	Observed hospitalizations per 1,000 population	Expected hospitalizations	Expected hospitalizations per 1,000 population	Difference in hospitalizations	Difference in rate	Hospitalizations observed-to-expected ratio	Observed length of stay	Expected length of stay	Difference in length of stay	Length of stay observed-to-expected ratio
Norway	2021	All causes	835,000	154	864,000 (841,000 to 888,000)	160 (155 to 164)	-28,500 (-52,400 to -5,340)	-5.27 (-9.69 to -0.986)	0.97 (0.94 to 0.99)				
Norway	2020	Infectious and parasitic diseases	15,500	2.89	18,200 (16,800 to 19,800)	3.39 (3.12 to 3.68)	-2,680 (-4,210 to -1,250)	-0.499 (-0.783 to -0.233)	0.85 (0.79 to 0.93)	5.3	5.10 (4.75 to 5.48)	0.200 (-0.178 to 0.554)	1.04 (0.97 to 1.12)
Norway	2021	Infectious and parasitic diseases	15,900	2.95	17,600 (15,700 to 19,700)	3.25 (2.90 to 3.65)	-1,670 (-3,840 to 215)	-0.308 (-0.711 to 0.040)	0.91 (0.81 to 1.01)	5.5	5.10 (4.61 to 5.64)	0.400 (-0.140 to 0.880)	1.08 (0.98 to 1.19)
Norway	2020	Neoplasms	70,700	13.1	74,900 (69,300 to 81,000)	13.9 (12.9 to 15.1)	-4,180 (-10,400 to 1,370)	-0.777 (-1.94 to 0.254)	0.94 (0.87 to 1.02)	5.3	5.29 (5.00 to 5.59)	0.010 (-0.291 to 0.297)	1.00 (0.95 to 1.06)
Norway	2021	Neoplasms	73,000	13.5	75,300 (67,400 to 84,100)	13.9 (12.5 to 15.6)	-2,310 (-11,100 to 5,650)	-0.427 (-2.06 to 1.04)	0.97 (0.87 to 1.08)	5.2	5.18 (4.79 to 5.60)	0.018 (-0.406 to 0.408)	1.00 (0.93 to 1.09)
Norway	2020	Diseases of the blood and bloodforming organs	6,430	1.19	7,040 (6,440 to 7,710)	1.31 (1.20 to 1.43)	-616 (-1,270 to -14)	-0.115 (-0.237 to -0.003)	0.91 (0.83 to 1.00)	3.6	3.91 (3.40 to 4.50)	-0.311 (-0.899 to 0.204)	0.92 (0.80 to 1.06)
Norway	2021	Diseases of the blood and bloodforming organs	6,900	1.28	7,080 (6,240 to 8,040)	1.31 (1.15 to 1.49)	-183 (-1,120 to 641)	-0.034 (-0.208 to 0.119)	0.97 (0.86 to 1.10)	3.5	3.91 (3.40 to 4.50)	-0.411 (-1.00 to 0.095)	0.89 (0.78 to 1.03)
Norway	2020	Endocrine, nutritional and metabolic diseases	16,200	3.02	17,900 (16,600 to 19,400)	3.33 (3.08 to 3.60)	-1,680 (-3,160 to -360)	-0.313 (-0.588 to -0.067)	0.91 (0.84 to 0.98)	3.9	3.75 (3.51 to 4.01)	0.152 (-0.106 to 0.391)	1.04 (0.97 to 1.11)
Norway	2021	Endocrine, nutritional and metabolic diseases	16,700	3.09	18,000 (16,100 to 20,100)	3.33 (2.98 to 3.72)	-1,320 (-3,420 to 597)	-0.245 (-0.632 to 0.110)	0.93 (0.83 to 1.04)	3.7	3.75 (3.51 to 4.01)	-0.048 (-0.310 to 0.192)	0.99 (0.92 to 1.05)
Norway	2020	Mental and behavioural disorders	71,800	13.3	60,800 (20,700 to 179,000)	11.3 (3.84 to 33.2)	11,100 (-105,000 to 50,400)	2.05 (-19.4 to 9.37)	1.18 (0.41 to 3.36)	17.2	15.88 (5.23 to 48.21)	1.32 (-31.75 to 12.06)	1.08 (0.35 to 3.35)
Norway	2021	Mental and behavioural disorders	73,000	13.5	61,100 (20,800 to 179,000)	11.3 (3.84 to 33.2)	11,900 (-108,000 to 52,800)	2.21 (-20.0 to 9.77)	1.20 (0.40 to 3.62)	17.7	15.88 (5.23 to 48.21)	1.82 (-30.82 to 12.33)	1.11 (0.36 to 3.30)
Norway	2020	Diseases of the nervous system	27,000	5.02	28,700 (27,800 to 29,600)	5.34 (5.17 to 5.51)	-1,680 (-2,580 to -772)	-0.311 (-0.480 to -0.143)	0.94 (0.91 to 0.97)	3.9	3.96 (3.61 to 4.34)	-0.056 (-0.434 to 0.294)	0.99 (0.90 to 1.08)
Norway	2021	Diseases of the nervous system	27,500	5.08	28,300 (27,400 to 29,300)	5.24 (5.07 to 5.41)	-826 (-1,770 to 83)	-0.153 (-0.327 to 0.015)	0.97 (0.94 to 1.00)	4.1	3.96 (3.61 to 4.34)	0.144 (-0.237 to 0.490)	1.04 (0.95 to 1.14)
Norway	2020	Diseases of the eye and adnexa	4,710	0.876	5,380 (5,060 to 5,720)	1.00 (0.940 to 1.06)	-667 (-1,010 to -354)	-0.124 (-0.188 to -0.066)	0.88 (0.82 to 0.93)	2.6	2.54 (2.38 to 2.71)	0.059 (-0.113 to 0.219)	1.02 (0.96 to 1.09)
Norway	2021	Diseases of the eye and adnexa	5,050	0.934	5,410 (5,010 to 5,840)	1.00 (0.926 to 1.08)	-356 (-789 to 50)	-0.066 (-0.146 to 0.009)	0.93 (0.86 to 1.01)	2.5	2.48 (2.26 to 2.72)	0.017 (-0.215 to 0.233)	1.01 (0.92 to 1.10)
Norway	2020	Diseases of the ear and mastoid process	3,820	0.71	4,150 (3,840 to 4,480)	0.771 (0.715 to 0.832)	-327 (-658 to -30)	-0.061 (-0.122 to -0.006)	0.92 (0.85 to 0.99)	2	2.10 (1.98 to 2.22)	-0.100 (-0.225 to 0.017)	0.95 (0.90 to 1.01)
Norway	2021	Diseases of the ear and mastoid process	4,070	0.752	4,170 (3,870 to 4,500)	0.771 (0.715 to 0.832)	-102 (-432 to 188)	-0.019 (-0.080 to 0.035)	0.98 (0.90 to 1.05)	2	2.10 (1.94 to 2.28)	-0.100 (-0.277 to 0.065)	0.95 (0.88 to 1.03)
Norway	2020	Diseases of the circulatory system	97,700	18.2	106,000 (97,200 to 115,000)	19.7 (18.1 to 21.5)	-8,180 (-17,600 to 695)	-1.52 (-3.26 to 0.129)	0.92 (0.85 to 1.01)	4.2	4.30 (4.06 to 4.55)	-0.100 (-0.359 to 0.138)	0.98 (0.92 to 1.03)
Norway	2021	Diseases of the circulatory system	102,000	18.8	106,000 (94,300 to 120,000)	19.7 (17.4 to 22.2)	-4,970 (-19,000 to 7,280)	-0.918 (-3.51 to 1.35)	0.95 (0.84 to 1.08)	4.3	4.30 (3.96 to 4.66)	0.000 (-0.362 to 0.328)	1.00 (0.92 to 1.08)

Location	Year	Condition	Observed hospitalizations	Observed hospitalizations per 1,000 population	Expected hospitalizations	Expected hospitalizations per 1,000 population	Difference in hospitalizations	Difference in rate	Hospitalizations observed-to-expected ratio	Observed length of stay	Expected length of stay	Difference in length of stay	Length of stay observed-to-expected ratio
Norway	2020	Diseases of the respiratory system	49,400	9.18	68,800 (65,200 to 72,600)	12.8 (12.1 to 13.5)	-19,400 (-23,200 to -15,800)	-3.61 (-4.32 to -2.94)	0.72 (0.68 to 0.76)	5	4.81 (4.60 to 5.03)	0.191 (-0.033 to 0.406)	1.04 (0.99 to 1.09)
Norway	2021	Diseases of the respiratory system	53,200	9.83	69,200 (65,500 to 73,000)	12.8 (12.1 to 13.5)	-16,000 (-19,900 to -12,400)	-2.96 (-3.68 to -2.29)	0.77 (0.73 to 0.81)	5.2	4.72 (4.43 to 5.03)	0.480 (0.173 to 0.775)	1.10 (1.03 to 1.18)
Norway	2020	Diseases of the digestive system	63,400	11.8	65,500 (63,000 to 68,200)	12.2 (11.7 to 12.7)	-2,130 (-4,770 to 448)	-0.396 (-0.887 to 0.083)	0.97 (0.93 to 1.01)	4	4.10 (3.85 to 4.37)	-0.100 (-0.368 to 0.145)	0.98 (0.92 to 1.04)
Norway	2021	Diseases of the digestive system	66,900	12.4	65,900 (62,300 to 69,700)	12.2 (11.5 to 12.9)	992 (-2,900 to 4,630)	0.183 (-0.535 to 0.855)	1.02 (0.96 to 1.07)	3.9	4.10 (3.75 to 4.48)	-0.200 (-0.591 to 0.155)	0.95 (0.87 to 1.04)
Norway	2020	Diseases of the skin and subcutaneous tissue	8,950	1.66	10,800 (10,000 to 11,700)	2.02 (1.86 to 2.18)	-1,900 (-2,780 to -1,100)	-0.353 (-0.518 to -0.204)	0.83 (0.76 to 0.89)	5.1	5.00 (4.49 to 5.57)	0.100 (-0.455 to 0.612)	1.02 (0.92 to 1.14)
Norway	2021	Diseases of the skin and subcutaneous tissue	9,810	1.81	11,400 (9,610 to 13,600)	2.12 (1.78 to 2.52)	-1,630 (-3,800 to 253)	-0.301 (-0.702 to 0.047)	0.86 (0.72 to 1.03)	5.1	5.00 (4.30 to 5.82)	0.100 (-0.709 to 0.813)	1.02 (0.88 to 1.19)
Norway	2020	Diseases of musculoskeletal system and connective tissue	45,500	8.46	52,500 (49,600 to 55,600)	9.76 (9.23 to 10.3)	-6,980 (-10,100 to -4,120)	-1.30 (-1.87 to -0.765)	0.87 (0.82 to 0.92)	3.6	3.61 (3.43 to 3.80)	-0.007 (-0.194 to 0.171)	1.00 (0.95 to 1.05)
Norway	2021	Diseases of musculoskeletal system and connective tissue	49,100	9.07	52,800 (48,800 to 57,200)	9.76 (9.02 to 10.6)	-3,720 (-8,010 to 436)	-0.688 (-1.48 to 0.081)	0.93 (0.86 to 1.01)	3.4	3.57 (3.39 to 3.76)	-0.174 (-0.363 to 0.002)	0.95 (0.90 to 1.00)
Norway	2020	Diseases of the genitourinary system	45,200	8.41	47,200 (43,900 to 50,700)	8.77 (8.16 to 9.42)	-1,940 (-5,510 to 1,350)	-0.360 (-1.02 to 0.250)	0.96 (0.89 to 1.03)	3.4	3.50 (3.17 to 3.87)	-0.100 (-0.467 to 0.238)	0.97 (0.88 to 1.08)
Norway	2021	Diseases of the genitourinary system	46,100	8.52	47,400 (44,200 to 51,000)	8.77 (8.16 to 9.42)	-1,380 (-4,910 to 1,920)	-0.255 (-0.907 to 0.354)	0.97 (0.90 to 1.04)	3.5	3.50 (3.04 to 4.03)	0.000 (-0.530 to 0.463)	1.00 (0.87 to 1.15)
Norway	2020	Pregnancy, childbirth and the puerperium	9,380	1.74	8,340 (4,560 to 15,300)	1.55 (0.847 to 2.84)	1,040 (-6,010 to 4,780)	0.193 (-1.12 to 0.889)	1.12 (0.61 to 2.04)	2.2	2.40 (1.90 to 3.04)	-0.200 (-0.859 to 0.308)	0.92 (0.72 to 1.16)
Norway	2021	Pregnancy, childbirth and the puerperium	9,850	1.82	6,670 (2,840 to 15,700)	1.23 (0.524 to 2.90)	3,180 (-5,770 to 7,010)	0.588 (-1.07 to 1.30)	1.48 (0.63 to 3.47)	2.2	2.40 (1.72 to 3.35)	-0.200 (-1.16 to 0.473)	0.92 (0.66 to 1.27)
Norway	2020	Certain conditions originating in the perinatal period	7,100	1.32	6,950 (6,060 to 7,970)	1.29 (1.13 to 1.48)	146 (-871 to 1,040)	0.027 (-0.162 to 0.194)	1.02 (0.89 to 1.17)	8.9	8.88 (8.32 to 9.47)	0.024 (-0.563 to 0.579)	1.00 (0.94 to 1.07)
Norway	2021	Certain conditions originating in the perinatal period	7,490	1.38	6,630 (5,460 to 8,050)	1.23 (1.01 to 1.49)	855 (-554 to 2,010)	0.158 (-0.102 to 0.372)	1.13 (0.93 to 1.37)	8.8	8.88 (8.32 to 9.47)	-0.076 (-0.674 to 0.476)	0.99 (0.93 to 1.06)
Norway	2020	Congenital malformations, deformations and chromosomal abnormalities	5,440	1.01	6,050 (5,680 to 6,460)	1.13 (1.05 to 1.20)	-616 (-1,010 to -239)	-0.115 (-0.188 to -0.044)	0.90 (0.84 to 0.96)	4.5	4.73 (4.48 to 4.99)	-0.228 (-0.497 to 0.023)	0.95 (0.90 to 1.01)
Norway	2021	Congenital malformations, deformations and chromosomal abnormalities	5,570	1.03	5,910 (5,400 to 6,480)	1.09 (0.998 to 1.20)	-343 (-901 to 178)	-0.063 (-0.167 to 0.033)	0.94 (0.86 to 1.03)	4.5	4.73 (4.48 to 4.99)	-0.228 (-0.500 to 0.025)	0.95 (0.90 to 1.01)

Location	Year	Condition	Observed hospitalizations	Observed hospitalizations per 1,000 population	Expected hospitalizations	Expected hospitalizations per 1,000 population	Difference in hospitalizations	Difference in rate	Hospitalizations observed-to-expected ratio	Observed length of stay	Expected length of stay	Difference in length of stay	Length of stay observed-to-expected ratio
Norway	2020	Symptoms, signs and abnormal clinical and laboratory findings, n.e.c.	56,000	10.4	60,900 (55,700 to 66,600)	11.3 (10.4 to 12.4)	-4,890 (-10,500 to 151)	-0.909 (-1.95 to 0.028)	0.92 (0.84 to 1.00)	2.2	2.17 (1.96 to 2.40)	0.032 (-0.195 to 0.235)	1.01 (0.92 to 1.12)
Norway	2021	Symptoms, signs and abnormal clinical and laboratory findings, n.e.c.	60,800	11.3	61,200 (56,000 to 67,000)	11.3 (10.4 to 12.4)	-389 (-6,090 to 4,820)	-0.072 (-1.13 to 0.892)	0.99 (0.91 to 1.09)	2.2	2.17 (1.96 to 2.40)	0.032 (-0.198 to 0.238)	1.01 (0.92 to 1.12)
Norway	2020	Injury, poisoning and other consequences of external causes	75,600	14.1	81,200 (73,800 to 89,400)	15.1 (13.7 to 16.6)	-5,610 (-13,700 to 1,810)	-1.04 (-2.55 to 0.337)	0.93 (0.85 to 1.02)	4	4.20 (3.86 to 4.58)	-0.200 (-0.592 to 0.145)	0.95 (0.87 to 1.04)
Norway	2021	Injury, poisoning and other consequences of external causes	80,000	14.8	81,700 (71,300 to 93,500)	15.1 (13.2 to 17.3)	-1,720 (-13,600 to 8,500)	-0.318 (-2.52 to 1.57)	0.98 (0.85 to 1.12)	3.9	4.20 (3.72 to 4.74)	-0.300 (-0.831 to 0.177)	0.93 (0.82 to 1.05)
Norway	2020	Factors influencing health status and contact with health services	116,000	21.6	126,000 (103,000 to 153,000)	23.4 (19.1 to 28.5)	-9,600 (-37,100 to 12,600)	-1.78 (-6.89 to 2.35)	0.92 (0.76 to 1.12)	3.8	4.10 (3.88 to 4.33)	-0.300 (-0.533 to -0.081)	0.93 (0.88 to 0.98)
Norway	2021	Factors influencing health status and contact with health services	123,000	22.8	126,000 (103,000 to 154,000)	23.4 (19.1 to 28.5)	-3,270 (-31,500 to 19,500)	-0.604 (-5.83 to 3.60)	0.97 (0.80 to 1.19)	4	4.10 (3.79 to 4.43)	-0.100 (-0.427 to 0.211)	0.98 (0.90 to 1.06)
New Zealand	2020	All causes	679,000	133	741,000 (727,000 to 755,000)	146 (143 to 148)	-61,800 (-76,400 to -47,800)	-12.1 (-15.0 to -9.39)	0.92 (0.90 to 0.93)				
New Zealand	2020	Infectious and parasitic diseases	15,500	3.05	17,600 (16,200 to 19,100)	3.46 (3.19 to 3.75)	-2,050 (-3,530 to -651)	-0.402 (-0.694 to -0.128)	0.88 (0.81 to 0.96)	5.5	4.60 (3.94 to 5.37)	0.900 (0.140 to 1.55)	1.20 (1.03 to 1.39)
New Zealand	2020	Neoplasms	36,900	7.24	37,200 (35,000 to 39,600)	7.31 (6.87 to 7.77)	-324 (-2,680 to 1,880)	-0.064 (-0.526 to 0.369)	0.99 (0.93 to 1.05)	6.8	7.40 (6.17 to 8.88)	-0.600 (-2.09 to 0.635)	0.92 (0.77 to 1.10)
New Zealand	2020	Diseases of the blood and bloodforming organs	4,820	0.947	5,090 (4,680 to 5,540)	1.00 (0.918 to 1.09)	-270 (-729 to 148)	-0.053 (-0.143 to 0.029)	0.95 (0.87 to 1.03)	5.1	4.70 (3.83 to 5.76)	0.400 (-0.644 to 1.26)	1.09 (0.89 to 1.33)
New Zealand	2020	Endocrine, nutritional and metabolic diseases	12,500	2.45	12,700 (11,100 to 14,500)	2.49 (2.19 to 2.84)	-241 (-2,020 to 1,300)	-0.047 (-0.397 to 0.255)	0.98 (0.86 to 1.12)	6.3	6.50 (5.03 to 8.39)	-0.200 (-2.11 to 1.25)	0.97 (0.75 to 1.25)
New Zealand	2020	Mental and behavioural disorders	24,800	4.88	25,300 (24,100 to 26,600)	4.98 (4.73 to 5.23)	-510 (-1,800 to 737)	-0.100 (-0.354 to 0.145)	0.98 (0.93 to 1.03)	30.3	34.00 (26.18 to 44.16)	-3.70 (-14.02 to 4.09)	0.89 (0.68 to 1.16)
New Zealand	2020	Diseases of the nervous system	17,400	3.41	17,400 (16,600 to 18,100)	3.41 (3.27 to 3.56)	-4 (-772 to 734)	-0.001 (-0.152 to 0.144)	1.00 (0.96 to 1.04)	11.6	14.30 (10.21 to 20.04)	-2.70 (-8.33 to 1.40)	0.81 (0.58 to 1.14)
New Zealand	2020	Diseases of the eye and adnexa	2,640	0.519	3,140 (2,880 to 3,420)	0.616 (0.566 to 0.671)	-494 (-772 to -239)	-0.097 (-0.152 to -0.047)	0.84 (0.77 to 0.92)	3	3.30 (1.71 to 6.38)	-0.300 (-3.31 to 1.29)	0.91 (0.48 to 1.76)
New Zealand	2020	Diseases of the ear and mastoid process	3,780	0.742	4,020 (3,750 to 4,300)	0.789 (0.737 to 0.845)	-240 (-523 to 28)	-0.047 (-0.103 to 0.006)	0.94 (0.88 to 1.01)	2.7	2.01 (1.24 to 3.25)	0.688 (-0.560 to 1.46)	1.34 (0.83 to 2.18)
New Zealand	2020	Diseases of the circulatory system	71,400	14	71,500 (69,600 to 73,400)	14.0 (13.7 to 14.4)	-101 (-2,020 to 1,790)	-0.020 (-0.398 to 0.351)	1.00 (0.97 to 1.03)	8.5	9.70 (7.57 to 12.43)	-1.20 (-3.87 to 0.933)	0.88 (0.69 to 1.12)

Location	Year	Condition	Observed hospitalizations	Observed hospitalizations per 1,000 population	Expected hospitalizations	Expected hospitalizations per 1,000 population	Difference in hospitalizations	Difference in rate	Hospitalizations observed-to-expected ratio	Observed length of stay	Expected length of stay	Difference in length of stay	Length of stay observed-to-expected ratio
New Zealand	2020	Diseases of the respiratory system	40,100	7.88	65,600 (61,900 to 69,400)	12.9 (12.2 to 13.6)	-25,500 (-29,300 to -21,800)	-5.00 (-5.76 to -4.28)	0.61 (0.58 to 0.65)	5.1	5.40 (4.17 to 6.99)	-0.300 (-1.93 to 0.934)	0.94 (0.73 to 1.22)
New Zealand	2020	Diseases of the digestive system	62,400	12.3	61,600 (59,000 to 64,300)	12.1 (11.6 to 12.6)	724 (-2,010 to 3,300)	0.142 (-0.394 to 0.649)	1.01 (0.97 to 1.06)	4	4.00 (3.60 to 4.44)	0.000 (-0.434 to 0.396)	1.00 (0.90 to 1.11)
New Zealand	2020	Diseases of the skin and subcutaneous tissue	16,700	3.27	20,000 (19,200 to 20,700)	3.92 (3.78 to 4.07)	-3,300 (-4,060 to -2,560)	-0.649 (-0.798 to -0.504)	0.83 (0.80 to 0.87)	4.4	4.21 (3.92 to 4.51)	0.192 (-0.120 to 0.480)	1.05 (0.97 to 1.12)
New Zealand	2020	Diseases of musculoskeletal system and connective tissue	43,200	8.49	44,600 (41,100 to 48,400)	8.76 (8.08 to 9.50)	-1,410 (-5,130 to 2,030)	-0.276 (-1.01 to 0.399)	0.97 (0.89 to 1.05)	4.3	4.50 (3.49 to 5.80)	-0.200 (-1.47 to 0.820)	0.96 (0.74 to 1.24)
New Zealand	2020	Diseases of the genitourinary system	33,800	6.64	34,900 (33,600 to 36,300)	6.87 (6.60 to 7.14)	-1,150 (-2,550 to 186)	-0.225 (-0.502 to 0.036)	0.97 (0.93 to 1.01)	3.8	3.90 (3.36 to 4.52)	-0.100 (-0.728 to 0.440)	0.97 (0.84 to 1.13)
New Zealand	2020	Pregnancy, childbirth and the puerperium	63,700	12.5	64,500 (62,900 to 66,100)	12.7 (12.4 to 13.0)	-802 (-2,420 to 746)	-0.158 (-0.475 to 0.147)	0.99 (0.96 to 1.01)	2.6	2.60 (2.54 to 2.66)	0.000 (-0.065 to 0.063)	1.00 (0.98 to 1.03)
New Zealand	2020	Certain conditions originating in the perinatal period	17,300	3.4	16,900 (16,200 to 17,600)	3.32 (3.19 to 3.46)	404 (-324 to 1,100)	0.079 (-0.064 to 0.216)	1.02 (0.98 to 1.07)	7.8	8.00 (7.57 to 8.45)	-0.200 (-0.644 to 0.224)	0.97 (0.92 to 1.03)
New Zealand	2020	Congenital malformations, deformations and chromosomal abnormalities	4,060	0.797	4,500 (4,040 to 5,000)	0.883 (0.794 to 0.982)	-437 (-928 to 15)	-0.086 (-0.182 to 0.003)	0.90 (0.81 to 1.00)	5.3	5.30 (3.77 to 7.46)	-0.004 (-2.15 to 1.51)	1.00 (0.71 to 1.40)
New Zealand	2020	Symptoms, signs and abnormal clinical and laboratory findings, n.e.c.	55,100	10.8	59,400 (55,700 to 63,400)	11.7 (10.9 to 12.5)	-4,350 (-8,450 to -595)	-0.854 (-1.66 to -0.117)	0.93 (0.87 to 0.99)	3.5	5.06 (3.75 to 6.82)	-1.56 (-3.35 to -0.260)	0.69 (0.51 to 0.93)
New Zealand	2020	Injury, poisoning and other consequences of external causes	87,100	17.1	89,100 (85,600 to 92,800)	17.5 (16.8 to 18.2)	-2,040 (-5,740 to 1,510)	-0.401 (-1.13 to 0.297)	0.98 (0.94 to 1.02)	5.1	4.29 (3.80 to 4.85)	0.808 (0.238 to 1.30)	1.19 (1.05 to 1.34)
New Zealand	2020	Factors influencing health status and contact with health services	66,200	13	81,800 (77,100 to 86,800)	16.1 (15.2 to 17.1)	-15,600 (-20,600 to -10,900)	-3.07 (-4.06 to -2.14)	0.81 (0.76 to 0.86)	2.9	6.46 (5.36 to 7.77)	-3.56 (-4.87 to -2.48)	0.45 (0.37 to 0.54)
Poland	2020	All causes	4,700,000	124	6,380,000 (6,030,000 to 6,750,000)	168 (159 to 178)	-1,680,000 (-2,050,000 to -1,330,000)	-44.2 (-54.0 to -35.2)	0.74 (0.70 to 0.78)				
Poland	2021	All causes	5,210,000	138	6,350,000 (5,860,000 to 6,880,000)	168 (155 to 182)	-1,140,000 (-1,680,000 to -657,000)	-30.2 (-44.5 to -17.4)	0.82 (0.76 to 0.89)				
Poland	2020	Infectious and parasitic diseases	93,200	2.46	171,000 (155,000 to 187,000)	4.50 (4.10 to 4.94)	-77,400 (-93,600 to -62,500)	-2.04 (-2.47 to -1.65)	0.55 (0.50 to 0.60)	9.5	7.97 (7.62 to 8.34)	1.53 (1.17 to 1.88)	1.19 (1.14 to 1.25)
Poland	2021	Infectious and parasitic diseases	113,000	2.99	170,000 (149,000 to 194,000)	4.50 (3.95 to 5.13)	-57,100 (-80,600 to -36,300)	-1.51 (-2.14 to -0.960)	0.66 (0.58 to 0.76)	8.3	7.97 (7.62 to 8.34)	0.332 (-0.045 to 0.682)	1.04 (0.99 to 1.09)
Poland	2020	Neoplasms	478,000	12.6	583,000 (540,000 to 630,000)	15.4 (14.3 to 16.6)	-105,000 (-152,000 to -61,400)	-2.77 (-4.01 to -1.62)	0.82 (0.76 to 0.89)	5.8	5.66 (5.52 to 5.81)	0.138 (-0.007 to 0.284)	1.02 (1.00 to 1.05)
Poland	2021	Neoplasms	534,000	14.1	581,000 (521,000 to 648,000)	15.4 (13.8 to 17.2)	-47,300 (-114,000 to 11,700)	-1.25 (-3.01 to 0.310)	0.92 (0.82 to 1.02)	5.6	5.53 (5.33 to 5.73)	0.072 (-0.134 to 0.270)	1.01 (0.98 to 1.05)

Location	Year	Condition	Observed hospitalizations	Observed hospitalizations per 1,000 population	Expected hospitalizations	Expected hospitalizations per 1,000 population	Difference in hospitalizations	Difference in rate	Hospitalizations observed-to-expected ratio	Observed length of stay	Expected length of stay	Difference in length of stay	Length of stay observed-to-expected ratio
Poland	2020	Diseases of the blood and bloodforming organs	48,400	1.28	65,700 (61,400 to 70,300)	1.73 (1.62 to 1.86)	-17,300 (-22,000 to -13,000)	-0.458 (-0.579 to -0.343)	0.74 (0.69 to 0.79)	6.6	6.40 (6.17 to 6.64)	0.200 (-0.038 to 0.423)	1.03 (0.99 to 1.07)
Poland	2021	Diseases of the blood and bloodforming organs	66,800	1.77	67,100 (61,000 to 73,900)	1.78 (1.62 to 1.96)	-365 (-7,140 to 5,560)	-0.010 (-0.189 to 0.147)	0.99 (0.90 to 1.09)	7.1	6.40 (6.08 to 6.74)	0.700 (0.357 to 1.03)	1.11 (1.05 to 1.17)
Poland	2020	Endocrine, nutritional and metabolic diseases	137,000	3.62	207,000 (194,000 to 221,000)	5.47 (5.13 to 5.84)	-70,300 (-84,100 to -57,400)	-1.86 (-2.22 to -1.52)	0.66 (0.62 to 0.70)	5.9	5.51 (5.33 to 5.69)	0.392 (0.210 to 0.569)	1.07 (1.04 to 1.11)
Poland	2021	Endocrine, nutritional and metabolic diseases	155,000	4.11	207,000 (189,000 to 226,000)	5.47 (5.00 to 5.99)	-51,400 (-71,100 to -33,500)	-1.36 (-1.88 to -0.887)	0.75 (0.69 to 0.82)	5.6	5.42 (5.17 to 5.68)	0.182 (-0.074 to 0.428)	1.03 (0.99 to 1.08)
Poland	2020	Mental and behavioural disorders	189,000	4.99	265,000 (252,000 to 279,000)	7.00 (6.65 to 7.37)	-76,400 (-90,200 to -63,100)	-2.02 (-2.38 to -1.66)	0.71 (0.68 to 0.75)	35.5	33.28 (27.84 to 39.77)	2.22 (-4.15 to 7.67)	1.07 (0.90 to 1.28)
Poland	2021	Mental and behavioural disorders	209,000	5.53	264,000 (251,000 to 278,000)	7.00 (6.65 to 7.37)	-55,500 (-69,300 to -42,100)	-1.47 (-1.84 to -1.12)	0.79 (0.75 to 0.83)	36.6	33.28 (27.84 to 39.77)	3.32 (-3.43 to 8.78)	1.10 (0.91 to 1.32)
Poland	2020	Diseases of the nervous system	157,000	4.13	246,000 (235,000 to 257,000)	6.48 (6.19 to 6.79)	-89,300 (-101,000 to -78,000)	-2.35 (-2.65 to -2.06)	0.64 (0.61 to 0.67)	8.5	7.90 (7.55 to 8.27)	0.600 (0.233 to 0.952)	1.08 (1.03 to 1.13)
Poland	2021	Diseases of the nervous system	171,000	4.54	245,000 (223,000 to 268,000)	6.48 (5.92 to 7.11)	-73,500 (-97,300 to -52,100)	-1.95 (-2.58 to -1.38)	0.70 (0.64 to 0.77)	8.4	7.90 (7.40 to 8.43)	0.500 (-0.030 to 0.986)	1.06 (1.00 to 1.13)
Poland	2020	Diseases of the eye and adnexa	44,200	1.17	155,000 (103,000 to 235,000)	4.10 (2.71 to 6.20)	-111,000 (-191,000 to -59,600)	-2.93 (-5.03 to -1.57)	0.28 (0.19 to 0.43)	3	2.60 (2.26 to 2.99)	0.400 (0.012 to 0.749)	1.15 (1.00 to 1.33)
Poland	2021	Diseases of the eye and adnexa	51,700	1.37	155,000 (102,000 to 234,000)	4.10 (2.71 to 6.20)	-103,000 (-183,000 to -51,200)	-2.73 (-4.86 to -1.36)	0.33 (0.22 to 0.50)	3.1	2.60 (2.13 to 3.17)	0.500 (-0.080 to 0.964)	1.19 (0.97 to 1.45)
Poland	2020	Diseases of the ear and mastoid process	21,300	0.562	42,400 (38,700 to 46,400)	1.12 (1.02 to 1.22)	-21,100 (-25,100 to -17,400)	-0.557 (-0.663 to -0.460)	0.50 (0.46 to 0.55)	4	3.90 (3.74 to 4.07)	0.098 (-0.067 to 0.257)	1.02 (0.98 to 1.07)
Poland	2021	Diseases of the ear and mastoid process	27,000	0.714	42,200 (38,600 to 46,200)	1.12 (1.02 to 1.22)	-15,300 (-19,300 to -11,700)	-0.405 (-0.512 to -0.310)	0.64 (0.58 to 0.70)	3.8	3.81 (3.47 to 4.18)	-0.007 (-0.379 to 0.334)	1.00 (0.91 to 1.10)
Poland	2020	Diseases of the circulatory system	712,000	18.8	1,020,000 (960,000 to 1,070,000)	26.8 (25.3 to 28.3)	-303,000 (-362,000 to -249,000)	-8.00 (-9.56 to -6.56)	0.70 (0.66 to 0.74)	6.9	6.60 (6.40 to 6.80)	0.300 (0.104 to 0.498)	1.05 (1.02 to 1.08)
Poland	2021	Diseases of the circulatory system	777,000	20.6	1,010,000 (957,000 to 1,070,000)	26.8 (25.3 to 28.3)	-235,000 (-293,000 to -179,000)	-6.22 (-7.75 to -4.74)	0.77 (0.73 to 0.81)	6.7	6.60 (6.17 to 7.07)	0.100 (-0.363 to 0.527)	1.02 (0.95 to 1.09)
Poland	2020	Diseases of the respiratory system	278,000	7.33	487,000 (438,000 to 541,000)	12.9 (11.6 to 14.3)	-209,000 (-264,000 to -160,000)	-5.53 (-6.96 to -4.23)	0.57 (0.51 to 0.63)	7.2	6.80 (6.60 to 7.01)	0.400 (0.191 to 0.599)	1.06 (1.03 to 1.09)
Poland	2021	Diseases of the respiratory system	323,000	8.57	485,000 (436,000 to 539,000)	12.9 (11.6 to 14.3)	-162,000 (-215,000 to -113,000)	-4.29 (-5.70 to -3.00)	0.67 (0.60 to 0.74)	6.9	6.80 (6.52 to 7.09)	0.100 (-0.192 to 0.390)	1.01 (0.97 to 1.06)
Poland	2020	Diseases of the digestive system	375,000	9.9	532,000 (500,000 to 566,000)	14.0 (13.2 to 14.9)	-156,000 (-190,000 to -124,000)	-4.13 (-5.01 to -3.27)	0.71 (0.66 to 0.75)	5.3	5.05 (4.91 to 5.19)	0.253 (0.113 to 0.390)	1.05 (1.02 to 1.08)
Poland	2021	Diseases of the digestive system	433,000	11.5	530,000 (485,000 to 578,000)	14.0 (12.9 to 15.3)	-96,200 (-144,000 to -52,300)	-2.55 (-3.82 to -1.38)	0.82 (0.75 to 0.89)	5	5.00 (4.81 to 5.19)	0.005 (-0.191 to 0.195)	1.00 (0.96 to 1.04)
Poland	2020	Diseases of the skin and subcutaneous tissue	60,100	1.58	99,500 (92,000 to 108,000)	2.62 (2.43 to 2.84)	-39,400 (-47,400 to -31,900)	-1.04 (-1.25 to -0.842)	0.60 (0.56 to 0.65)	7.1	6.60 (6.49 to 6.72)	0.499 (0.381 to 0.612)	1.08 (1.06 to 1.09)

Location	Year	Condition	Observed hospitalizations	Observed hospitalizations per 1,000 population	Expected hospitalizations	Expected hospitalizations per 1,000 population	Difference in hospitalizations	Difference in rate	Hospitalizations observed-to-expected ratio	Observed length of stay	Expected length of stay	Difference in length of stay	Length of stay observed-to-expected ratio
Poland	2021	Diseases of the skin and subcutaneous tissue	66,600	1.76	99,100 (91,600 to 107,000)	2.62 (2.43 to 2.84)	-32,500 (-40,500 to -25,200)	-0.861 (-1.07 to -0.668)	0.67 (0.62 to 0.73)	6.6	6.50 (6.26 to 6.76)	0.096 (-0.162 to 0.347)	1.01 (0.98 to 1.06)
Poland	2020	Diseases of musculoskeletal system and connective tissue	273,000	7.2	407,000 (381,000 to 433,000)	10.7 (10.1 to 11.4)	-134,000 (-161,000 to -109,000)	-3.53 (-4.25 to -2.87)	0.67 (0.63 to 0.71)	8	8.09 (7.60 to 8.62)	-0.095 (-0.621 to 0.395)	0.99 (0.93 to 1.05)
Poland	2021	Diseases of musculoskeletal system and connective tissue	324,000	8.6	415,000 (379,000 to 454,000)	11.0 (10.0 to 12.0)	-90,200 (-129,000 to -54,300)	-2.39 (-3.43 to -1.44)	0.78 (0.71 to 0.86)	7.9	7.89 (7.22 to 8.63)	0.006 (-0.729 to 0.671)	1.00 (0.92 to 1.09)
Poland	2020	Diseases of the genitourinary system	290,000	7.65	405,000 (386,000 to 424,000)	10.7 (10.2 to 11.2)	-115,000 (-134,000 to -95,800)	-3.02 (-3.54 to -2.53)	0.72 (0.68 to 0.75)	4.7	4.55 (4.45 to 4.65)	0.150 (0.046 to 0.253)	1.03 (1.01 to 1.06)
Poland	2021	Diseases of the genitourinary system	337,000	8.92	403,000 (377,000 to 431,000)	10.7 (9.98 to 11.4)	-66,200 (-94,600 to -40,200)	-1.75 (-2.51 to -1.07)	0.84 (0.78 to 0.89)	4.5	4.55 (4.45 to 4.65)	-0.050 (-0.151 to 0.052)	0.99 (0.97 to 1.01)
Poland	2020	Pregnancy, childbirth and the puerperium	457,000	12.1	557,000 (519,000 to 598,000)	14.7 (13.7 to 15.8)	-100,000 (-141,000 to -61,600)	-2.64 (-3.72 to -1.63)	0.82 (0.76 to 0.88)	4.3	4.37 (4.27 to 4.46)	-0.068 (-0.164 to 0.027)	0.98 (0.96 to 1.01)
Poland	2021	Pregnancy, childbirth and the puerperium	449,000	11.9	555,000 (517,000 to 596,000)	14.7 (13.7 to 15.8)	-106,000 (-147,000 to -68,300)	-2.82 (-3.90 to -1.81)	0.81 (0.75 to 0.87)	4.4	4.34 (4.21 to 4.47)	0.064 (-0.068 to 0.193)	1.01 (0.98 to 1.05)
Poland	2020	Certain conditions originating in the perinatal period	147,000	3.89	155,000 (146,000 to 165,000)	4.09 (3.84 to 4.36)	-7,680 (-17,800 to 1,770)	-0.203 (-0.470 to 0.047)	0.95 (0.89 to 1.01)	6	6.36 (6.03 to 6.70)	-0.358 (-0.699 to -0.024)	0.94 (0.90 to 1.00)
Poland	2021	Certain conditions originating in the perinatal period	151,000	4	154,000 (145,000 to 164,000)	4.09 (3.84 to 4.36)	-3,330 (-13,300 to 6,170)	-0.088 (-0.352 to 0.164)	0.98 (0.92 to 1.04)	6	6.36 (6.03 to 6.70)	-0.358 (-0.695 to -0.037)	0.94 (0.90 to 0.99)
Poland	2020	Congenital malformations, deformations and chromosomal abnormalities	43,700	1.15	56,000 (51,200 to 61,200)	1.48 (1.35 to 1.61)	-12,300 (-17,400 to -7,490)	-0.324 (-0.460 to -0.198)	0.78 (0.72 to 0.85)	5.7	5.40 (5.11 to 5.71)	0.300 (-0.003 to 0.591)	1.06 (1.00 to 1.12)
Poland	2021	Congenital malformations, deformations and chromosomal abnormalities	54,100	1.43	55,800 (49,200 to 63,200)	1.48 (1.30 to 1.67)	-1,620 (-8,980 to 4,970)	-0.043 (-0.238 to 0.132)	0.97 (0.86 to 1.10)	5.2	5.40 (4.77 to 6.11)	-0.200 (-0.913 to 0.420)	0.96 (0.85 to 1.09)
Poland	2020	Symptoms, signs and abnormal clinical and laboratory findings, n.e.c.	190,000	5.02	281,000 (254,000 to 310,000)	7.41 (6.70 to 8.19)	-90,600 (-120,000 to -63,700)	-2.39 (-3.16 to -1.68)	0.68 (0.61 to 0.75)	5.2	4.80 (4.45 to 5.18)	0.400 (0.025 to 0.748)	1.08 (1.00 to 1.17)
Poland	2021	Symptoms, signs and abnormal clinical and laboratory findings, n.e.c.	231,000	6.11	280,000 (243,000 to 322,000)	7.41 (6.43 to 8.53)	-48,900 (-91,600 to -11,000)	-1.29 (-2.43 to -0.292)	0.83 (0.72 to 0.95)	5	4.80 (4.31 to 5.34)	0.200 (-0.341 to 0.683)	1.04 (0.94 to 1.16)
Poland	2020	Injury, poisoning and other consequences of external causes	313,000	8.26	412,000 (393,000 to 432,000)	10.9 (10.4 to 11.4)	-98,900 (-119,000 to -79,800)	-2.61 (-3.14 to -2.11)	0.76 (0.72 to 0.80)	5.7	5.60 (5.39 to 5.82)	0.100 (-0.116 to 0.307)	1.02 (0.98 to 1.06)
Poland	2021	Injury, poisoning and other consequences of external causes	357,000	9.47	410,000 (391,000 to 430,000)	10.9 (10.4 to 11.4)	-52,800 (-72,600 to -34,000)	-1.40 (-1.92 to -0.902)	0.87 (0.83 to 0.91)	5.4	5.60 (5.31 to 5.91)	-0.200 (-0.507 to 0.093)	0.96 (0.91 to 1.02)

Location	Year	Condition	Observed hospitalizations	Observed hospitalizations per 1,000 population	Expected hospitalizations	Expected hospitalizations per 1,000 population	Difference in hospitalizations	Difference in rate	Hospitalizations observed-to-expected ratio	Observed length of stay	Expected length of stay	Difference in length of stay	Length of stay observed-to-expected ratio
Poland	2020	Factors influencing health status and contact with health services	392,000	10.4	452,000 (408,000 to 500,000)	11.9 (10.8 to 13.2)	-59,300 (-107,000 to -17,100)	-1.57 (-2.83 to -0.450)	0.87 (0.79 to 0.96)	4.6	4.87 (4.53 to 5.23)	-0.267 (-0.631 to 0.069)	0.95 (0.88 to 1.02)
Poland	2021	Factors influencing health status and contact with health services	380,000	10.1	450,000 (390,000 to 519,000)	11.9 (10.3 to 13.7)	-70,300 (-140,000 to -10,300)	-1.86 (-3.71 to -0.273)	0.84 (0.73 to 0.97)	4.7	4.87 (4.53 to 5.23)	-0.167 (-0.521 to 0.170)	0.97 (0.90 to 1.04)
Slovak Republic	2020	All causes	840,000	154	1,050,000 (1,000,000 to 1,090,000)	192 (183 to 200)	-206,000 (-255,000 to -160,000)	-37.7 (-46.6 to -29.3)	0.80 (0.77 to 0.84)				
Slovak Republic	2021	All causes	762,000	140	1,040,000 (997,000 to 1,090,000)	192 (183 to 200)	-281,000 (-329,000 to -236,000)	-51.6 (-60.3 to -43.4)	0.73 (0.70 to 0.76)				
Slovak Republic	2020	Infectious and parasitic diseases	21,000	3.84	33,300 (30,400 to 36,400)	6.09 (5.56 to 6.67)	-12,300 (-15,400 to -9,410)	-2.25 (-2.82 to -1.72)	0.63 (0.58 to 0.69)	7.5	6.99 (6.40 to 7.64)	0.506 (-0.143 to 1.10)	1.07 (0.98 to 1.17)
Slovak Republic	2021	Infectious and parasitic diseases	21,300	3.92	34,700 (30,500 to 39,400)	6.37 (5.60 to 7.24)	-13,400 (-18,100 to -9,100)	-2.45 (-3.33 to -1.67)	0.61 (0.54 to 0.70)	7.3	6.99 (6.40 to 7.64)	0.306 (-0.340 to 0.903)	1.04 (0.96 to 1.14)
Slovak Republic	2020	Neoplasms	84,900	15.6	94,300 (90,300 to 98,400)	17.3 (16.5 to 18.0)	-9,350 (-13,500 to -5,380)	-1.71 (-2.47 to -0.986)	0.90 (0.86 to 0.94)	7.1	7.40 (7.01 to 7.82)	-0.300 (-0.719 to 0.097)	0.96 (0.91 to 1.01)
Slovak Republic	2021	Neoplasms	79,300	14.6	94,100 (88,500 to 100,000)	17.3 (16.2 to 18.4)	-14,800 (-20,700 to -9,060)	-2.71 (-3.79 to -1.66)	0.84 (0.79 to 0.90)	6.7	7.40 (6.85 to 7.99)	-0.700 (-1.29 to -0.161)	0.91 (0.84 to 0.98)
Slovak Republic	2020	Diseases of the blood and bloodforming organs	9,700	1.78	11,300 (10,700 to 12,000)	2.08 (1.97 to 2.19)	-1,630 (-2,250 to -1,030)	-0.299 (-0.412 to -0.189)	0.86 (0.81 to 0.90)	6.8	6.70 (6.24 to 7.20)	0.100 (-0.402 to 0.567)	1.01 (0.94 to 1.09)
Slovak Republic	2021	Diseases of the blood and bloodforming organs	8,950	1.64	11,700 (10,900 to 12,700)	2.15 (2.00 to 2.32)	-2,780 (-3,710 to -1,920)	-0.510 (-0.681 to -0.352)	0.76 (0.71 to 0.82)	6.6	6.70 (6.05 to 7.42)	-0.100 (-0.817 to 0.551)	0.99 (0.89 to 1.09)
Slovak Republic	2020	Endocrine, nutritional and metabolic diseases	21,100	3.87	25,200 (22,400 to 28,400)	4.62 (4.10 to 5.20)	-4,080 (-7,140 to -1,220)	-0.746 (-1.31 to -0.224)	0.84 (0.75 to 0.95)	7.3	7.40 (6.95 to 7.88)	-0.100 (-0.581 to 0.350)	0.99 (0.93 to 1.05)
Slovak Republic	2021	Endocrine, nutritional and metabolic diseases	20,000	3.67	25,200 (21,300 to 29,700)	4.62 (3.91 to 5.46)	-5,170 (-9,760 to -1,360)	-0.950 (-1.79 to -0.249)	0.79 (0.67 to 0.94)	7.1	7.40 (6.77 to 8.09)	-0.300 (-0.983 to 0.323)	0.96 (0.88 to 1.05)
Slovak Republic	2020	Mental and behavioural disorders	37,700	6.91	44,600 (42,600 to 46,700)	8.16 (7.80 to 8.55)	-6,860 (-8,950 to -4,850)	-1.26 (-1.64 to -0.889)	0.85 (0.81 to 0.89)	29.2	27.50 (26.49 to 28.55)	1.70 (0.674 to 2.71)	1.06 (1.02 to 1.10)
Slovak Republic	2021	Mental and behavioural disorders	37,200	6.83	44,500 (41,700 to 47,500)	8.16 (7.65 to 8.72)	-7,290 (-10,200 to -4,460)	-1.34 (-1.88 to -0.818)	0.84 (0.78 to 0.89)	28.5	27.50 (26.09 to 28.99)	1.00 (-0.494 to 2.37)	1.04 (0.98 to 1.09)
Slovak Republic	2020	Diseases of the nervous system	27,600	5.06	37,100 (35,200 to 39,100)	6.79 (6.44 to 7.16)	-9,460 (-11,500 to -7,560)	-1.73 (-2.11 to -1.38)	0.74 (0.71 to 0.79)	7.2	7.00 (6.82 to 7.18)	0.200 (0.016 to 0.379)	1.03 (1.00 to 1.06)
Slovak Republic	2021	Diseases of the nervous system	23,500	4.32	37,800 (34,500 to 41,400)	6.94 (6.34 to 7.60)	-14,300 (-17,900 to -11,000)	-2.62 (-3.28 to -2.01)	0.62 (0.57 to 0.68)	7.1	7.00 (6.75 to 7.26)	0.100 (-0.156 to 0.352)	1.01 (0.98 to 1.05)
Slovak Republic	2020	Diseases of the eye and adnexa	6,930	1.27	9,240 (6,300 to 13,500)	1.69 (1.15 to 2.48)	-2,310 (-6,590 to 649)	-0.423 (-1.21 to 0.119)	0.75 (0.51 to 1.10)	4	3.89 (3.47 to 4.38)	0.106 (-0.376 to 0.543)	1.03 (0.91 to 1.16)
Slovak Republic	2021	Diseases of the eye and adnexa	6,860	1.26	9,220 (5,360 to 15,800)	1.69 (0.984 to 2.91)	-2,360 (-8,900 to 1,500)	-0.432 (-1.63 to 0.274)	0.74 (0.44 to 1.28)	4.2	3.89 (3.47 to 4.38)	0.306 (-0.173 to 0.734)	1.08 (0.96 to 1.21)

Location	Year	Condition	Observed hospitalizations	Observed hospitalizations per 1,000 population	Expected hospitalizations	Expected hospitalizations per 1,000 population	Difference in hospitalizations	Difference in rate	Hospitalizations observed-to-expected ratio	Observed length of stay	Expected length of stay	Difference in length of stay	Length of stay observed-to-expected ratio
Slovak Republic	2020	Diseases of the ear and mastoid process	6,590	1.21	9,780 (9,180 to 10,400)	1.79 (1.68 to 1.91)	-3,200 (-3,840 to -2,590)	-0.585 (-0.704 to -0.474)	0.67 (0.63 to 0.72)	5	4.83 (4.75 to 4.92)	0.170 (0.087 to 0.254)	1.04 (1.02 to 1.05)
Slovak Republic	2021	Diseases of the ear and mastoid process	5,350	0.983	9,760 (9,160 to 10,400)	1.79 (1.68 to 1.91)	-4,410 (-5,080 to -3,810)	-0.809 (-0.932 to -0.700)	0.55 (0.51 to 0.58)	4.7	4.76 (4.64 to 4.88)	-0.061 (-0.177 to 0.058)	0.99 (0.96 to 1.01)
Slovak Republic	2020	Diseases of the circulatory system	121,000	22.1	153,000 (140,000 to 166,000)	27.9 (25.7 to 30.4)	-31,900 (-45,300 to -19,600)	-5.85 (-8.30 to -3.59)	0.79 (0.73 to 0.86)	6.7	6.80 (6.44 to 7.18)	-0.100 (-0.480 to 0.262)	0.99 (0.93 to 1.04)
Slovak Republic	2021	Diseases of the circulatory system	109,000	20.1	152,000 (140,000 to 166,000)	27.9 (25.7 to 30.4)	-42,900 (-56,000 to -30,400)	-7.88 (-10.3 to -5.58)	0.72 (0.66 to 0.78)	6.5	6.80 (6.30 to 7.34)	-0.300 (-0.838 to 0.196)	0.96 (0.89 to 1.03)
Slovak Republic	2020	Diseases of the respiratory system	56,300	10.3	76,900 (71,900 to 82,200)	14.1 (13.2 to 15.1)	-20,500 (-25,900 to -15,600)	-3.76 (-4.74 to -2.86)	0.73 (0.69 to 0.78)	7.7	7.24 (7.10 to 7.38)	0.460 (0.319 to 0.602)	1.06 (1.04 to 1.08)
Slovak Republic	2021	Diseases of the respiratory system	82,600	15.2	76,700 (71,800 to 82,000)	14.1 (13.2 to 15.1)	5,860 (662 to 10,800)	1.08 (0.122 to 1.99)	1.08 (1.01 to 1.15)	8.5	7.18 (6.99 to 7.38)	1.32 (1.12 to 1.51)	1.18 (1.15 to 1.22)
Slovak Republic	2020	Diseases of the digestive system	73,000	13.4	97,900 (91,700 to 104,000)	17.9 (16.8 to 19.1)	-24,900 (-31,600 to -18,500)	-4.55 (-5.78 to -3.38)	0.75 (0.70 to 0.80)	5.6	5.30 (4.97 to 5.65)	0.300 (-0.053 to 0.627)	1.06 (0.99 to 1.13)
Slovak Republic	2021	Diseases of the digestive system	68,400	12.6	97,700 (91,500 to 104,000)	17.9 (16.8 to 19.1)	-29,300 (-35,700 to -23,000)	-5.38 (-6.55 to -4.22)	0.70 (0.66 to 0.75)	5.5	5.30 (4.84 to 5.81)	0.200 (-0.308 to 0.656)	1.04 (0.95 to 1.14)
Slovak Republic	2020	Diseases of the skin and subcutaneous tissue	10,500	1.92	15,400 (14,500 to 16,300)	2.82 (2.66 to 2.99)	-4,900 (-5,800 to -4,060)	-0.898 (-1.06 to -0.744)	0.68 (0.64 to 0.72)	7.1	6.95 (6.62 to 7.30)	0.148 (-0.203 to 0.486)	1.02 (0.97 to 1.07)
Slovak Republic	2021	Diseases of the skin and subcutaneous tissue	8,620	1.58	15,400 (14,500 to 16,300)	2.82 (2.66 to 2.99)	-6,740 (-7,660 to -5,880)	-1.24 (-1.41 to -1.08)	0.56 (0.53 to 0.59)	7.2	6.81 (6.35 to 7.30)	0.392 (-0.098 to 0.845)	1.06 (0.99 to 1.13)
Slovak Republic	2020	Diseases of musculoskeletal system and connective tissue	55,800	10.2	84,000 (77,100 to 91,400)	15.4 (14.1 to 16.7)	-28,200 (-35,500 to -21,400)	-5.16 (-6.50 to -3.91)	0.66 (0.61 to 0.72)	7.1	6.96 (6.63 to 7.32)	0.137 (-0.219 to 0.475)	1.02 (0.97 to 1.07)
Slovak Republic	2021	Diseases of musculoskeletal system and connective tissue	38,400	7.06	86,700 (76,900 to 97,800)	15.9 (14.1 to 17.9)	-48,300 (-59,400 to -38,100)	-8.86 (-10.9 to -6.99)	0.44 (0.39 to 0.50)	7	6.83 (6.37 to 7.32)	0.172 (-0.315 to 0.643)	1.03 (0.96 to 1.10)
Slovak Republic	2020	Diseases of the genitourinary system	39,100	7.17	50,100 (46,300 to 54,200)	9.18 (8.49 to 9.92)	-11,000 (-15,100 to -7,230)	-2.01 (-2.76 to -1.32)	0.78 (0.72 to 0.84)	5.1	5.00 (4.65 to 5.38)	0.100 (-0.282 to 0.457)	1.02 (0.95 to 1.10)
Slovak Republic	2021	Diseases of the genitourinary system	35,200	6.47	50,000 (44,800 to 55,800)	9.18 (8.22 to 10.2)	-14,800 (-20,600 to -9,560)	-2.71 (-3.77 to -1.76)	0.70 (0.63 to 0.79)	5	5.00 (4.51 to 5.55)	0.000 (-0.545 to 0.492)	1.00 (0.90 to 1.11)
Slovak Republic	2020	Pregnancy, childbirth and the puerperium	72,600	13.3	75,900 (73,400 to 78,600)	13.9 (13.4 to 14.4)	-3,350 (-5,920 to -795)	-0.614 (-1.08 to -0.146)	0.96 (0.92 to 0.99)	4.7	4.90 (4.63 to 5.19)	-0.200 (-0.490 to 0.071)	0.96 (0.91 to 1.02)
Slovak Republic	2021	Pregnancy, childbirth and the puerperium	70,700	13	75,800 (70,600 to 81,300)	13.9 (13.0 to 14.9)	-5,070 (-10,500 to 95)	-0.931 (-1.93 to 0.017)	0.93 (0.87 to 1.00)	4.6	4.90 (4.52 to 5.31)	-0.300 (-0.717 to 0.077)	0.94 (0.87 to 1.02)
Slovak Republic	2020	Certain conditions originating in the perinatal period	16,500	3.02	19,000 (16,500 to 21,800)	3.48 (3.03 to 4.00)	-2,540 (-5,380 to -54)	-0.465 (-0.985 to -0.010)	0.87 (0.75 to 1.00)	7.9	7.99 (7.36 to 8.68)	-0.094 (-0.779 to 0.542)	0.99 (0.91 to 1.07)
Slovak Republic	2021	Certain conditions originating in the perinatal period	15,600	2.87	19,000 (16,500 to 21,800)	3.48 (3.03 to 4.00)	-3,350 (-6,240 to -894)	-0.614 (-1.15 to -0.164)	0.82 (0.71 to 0.95)	8.1	7.99 (7.36 to 8.68)	0.106 (-0.585 to 0.741)	1.01 (0.93 to 1.10)

Location	Year	Condition	Observed hospitalizations	Observed hospitalizations per 1,000 population	Expected hospitalizations	Expected hospitalizations per 1,000 population	Difference in hospitalizations	Difference in rate	Hospitalizations observed-to-expected ratio	Observed length of stay	Expected length of stay	Difference in length of stay	Length of stay observed-to-expected ratio
Slovak Republic	2020	Congenital malformations, deformations and chromosomal abnormalities	5,160	0.946	5,960 (5,600 to 6,350)	1.09 (1.03 to 1.16)	-796 (-1,180 to -434)	-0.146 (-0.216 to -0.079)	0.87 (0.81 to 0.92)	5.7	5.78 (5.23 to 6.39)	-0.082 (-0.683 to 0.474)	0.99 (0.89 to 1.09)
Slovak Republic	2021	Congenital malformations, deformations and chromosomal abnormalities	5,250	0.963	5,950 (5,580 to 6,340)	1.09 (1.03 to 1.16)	-701 (-1,090 to -339)	-0.129 (-0.201 to -0.062)	0.88 (0.83 to 0.94)	6	5.78 (5.23 to 6.39)	0.218 (-0.391 to 0.760)	1.04 (0.94 to 1.15)
Slovak Republic	2020	Symptoms, signs and abnormal clinical and laboratory findings, n.e.c.	29,800	5.46	37,400 (31,000 to 45,100)	6.85 (5.67 to 8.26)	-7,580 (-15,400 to -1,270)	-1.39 (-2.82 to -0.233)	0.80 (0.66 to 0.96)	6.5	6.50 (6.16 to 6.86)	0.000 (-0.369 to 0.344)	1.00 (0.95 to 1.06)
Slovak Republic	2021	Symptoms, signs and abnormal clinical and laboratory findings, n.e.c.	27,600	5.07	37,300 (30,900 to 45,000)	6.85 (5.67 to 8.26)	-9,700 (-17,500 to -3,410)	-1.78 (-3.21 to -0.626)	0.74 (0.61 to 0.89)	6.7	6.50 (6.02 to 7.02)	0.200 (-0.330 to 0.675)	1.03 (0.95 to 1.11)
Slovak Republic	2020	Injury, poisoning and other consequences of external causes	64,300	11.8	78,000 (74,900 to 81,300)	14.3 (13.7 to 14.9)	-13,700 (-17,000 to -10,600)	-2.51 (-3.11 to -1.93)	0.82 (0.79 to 0.86)	6.3	6.14 (5.77 to 6.54)	0.158 (-0.235 to 0.526)	1.03 (0.96 to 1.09)
Slovak Republic	2021	Injury, poisoning and other consequences of external causes	57,500	10.6	77,900 (74,700 to 81,200)	14.3 (13.7 to 14.9)	-20,400 (-23,700 to -17,300)	-3.74 (-4.34 to -3.17)	0.74 (0.71 to 0.77)	5.9	6.14 (5.77 to 6.54)	-0.242 (-0.647 to 0.134)	0.96 (0.90 to 1.02)
Slovak Republic	2020	Factors influencing health status and contact with health services	80,700	14.8	96,300 (89,200 to 104,000)	17.6 (16.3 to 19.0)	-15,600 (-23,300 to -8,560)	-2.86 (-4.27 to -1.57)	0.84 (0.78 to 0.90)	4	4.00 (3.71 to 4.31)	0.000 (-0.313 to 0.289)	1.00 (0.93 to 1.08)
Slovak Republic	2021	Factors influencing health status and contact with health services	40,600	7.45	96,100 (89,000 to 104,000)	17.6 (16.3 to 19.0)	-55,500 (-63,100 to -48,500)	-10.2 (-11.6 to -8.90)	0.42 (0.39 to 0.46)	4	4.00 (3.60 to 4.45)	0.000 (-0.465 to 0.398)	1.00 (0.90 to 1.11)
Slovenia	2020	All causes	300,000	143	364,000 (352,000 to 376,000)	173 (167 to 179)	-63,400 (-76,200 to -51,100)	-30.2 (-36.3 to -24.3)	0.83 (0.80 to 0.85)				
Slovenia	2021	All causes	318,000	151	365,000 (348,000 to 383,000)	173 (165 to 182)	-46,400 (-64,300 to -29,500)	-22.0 (-30.5 to -14.0)	0.87 (0.83 to 0.92)				
Slovenia	2020	Infectious and parasitic diseases	9,360	4.45	11,700 (11,100 to 12,400)	5.58 (5.27 to 5.91)	-2,370 (-3,080 to -1,720)	-1.13 (-1.46 to -0.817)	0.80 (0.75 to 0.84)	9.3	6.82 (6.30 to 7.38)	2.48 (1.94 to 3.01)	1.36 (1.26 to 1.48)
Slovenia	2021	Infectious and parasitic diseases	11,100	5.25	11,500 (10,700 to 12,400)	5.47 (5.10 to 5.86)	-447 (-1,260 to 338)	-0.212 (-0.596 to 0.160)	0.96 (0.90 to 1.03)	7.8	6.86 (6.24 to 7.53)	0.945 (0.284 to 1.56)	1.14 (1.04 to 1.25)
Slovenia	2020	Neoplasms	32,100	15.3	37,200 (35,100 to 39,500)	17.7 (16.7 to 18.8)	-5,130 (-7,350 to -2,960)	-2.44 (-3.49 to -1.41)	0.86 (0.81 to 0.92)	6.9	7.30 (6.21 to 8.57)	-0.400 (-1.69 to 0.699)	0.95 (0.80 to 1.11)
Slovenia	2021	Neoplasms	32,600	15.5	37,300 (35,200 to 39,600)	17.7 (16.7 to 18.8)	-4,740 (-6,950 to -2,570)	-2.25 (-3.30 to -1.22)	0.87 (0.82 to 0.93)	6.5	7.30 (5.81 to 9.17)	-0.800 (-2.71 to 0.689)	0.89 (0.71 to 1.12)
Slovenia	2020	Diseases of the blood and bloodforming organs	2,460	1.17	2,640 (2,380 to 2,920)	1.26 (1.13 to 1.39)	-180 (-463 to 72)	-0.086 (-0.220 to 0.034)	0.93 (0.84 to 1.03)	5.6	6.05 (4.38 to 8.36)	-0.452 (-2.81 to 1.22)	0.93 (0.67 to 1.28)

Location	Year	Condition	Observed hospitalizations	Observed hospitalizations per 1,000 population	Expected hospitalizations	Expected hospitalizations per 1,000 population	Difference in hospitalizations	Difference in rate	Hospitalizations observed-to-expected ratio	Observed length of stay	Expected length of stay	Difference in length of stay	Length of stay observed-to-expected ratio
Slovenia	2021	Diseases of the blood and bloodforming organs	2,540	1.21	2,520 (2,010 to 3,170)	1.20 (0.953 to 1.51)	17 (-630 to 541)	0.008 (-0.299 to 0.257)	1.01 (0.80 to 1.27)	5.4	6.05 (4.38 to 8.36)	-0.652 (-2.95 to 1.01)	0.89 (0.65 to 1.23)
Slovenia	2020	Endocrine, nutritional and metabolic diseases	6,240	2.97	7,690 (7,110 to 8,310)	3.66 (3.38 to 3.95)	-1,450 (-2,070 to -879)	-0.691 (-0.986 to -0.418)	0.81 (0.75 to 0.88)	5.8	6.04 (5.56 to 6.55)	-0.235 (-0.743 to 0.240)	0.96 (0.89 to 1.04)
Slovenia	2021	Endocrine, nutritional and metabolic diseases	6,360	3.02	7,710 (7,130 to 8,330)	3.66 (3.38 to 3.95)	-1,350 (-1,970 to -782)	-0.642 (-0.936 to -0.371)	0.82 (0.76 to 0.89)	5.3	6.04 (5.56 to 6.55)	-0.735 (-1.25 to -0.255)	0.88 (0.81 to 0.95)
Slovenia	2020	Mental and behavioural disorders	9,770	4.65	11,800 (11,500 to 12,200)	5.63 (5.46 to 5.81)	-2,080 (-2,450 to -1,710)	-0.988 (-1.17 to -0.812)	0.82 (0.80 to 0.85)	27.3	31.50 (29.63 to 33.49)	-4.20 (-6.12 to -2.34)	0.87 (0.82 to 0.92)
Slovenia	2021	Mental and behavioural disorders	10,700	5.08	11,900 (11,500 to 12,300)	5.63 (5.46 to 5.81)	-1,170 (-1,550 to -805)	-0.556 (-0.734 to -0.382)	0.90 (0.87 to 0.93)	23.4	31.50 (28.89 to 34.35)	-8.10 (-10.95 to -5.49)	0.74 (0.68 to 0.81)
Slovenia	2020	Diseases of the nervous system	6,340	3.01	7,580 (7,300 to 7,880)	3.61 (3.47 to 3.75)	-1,250 (-1,540 to -968)	-0.594 (-0.734 to -0.460)	0.84 (0.80 to 0.87)	8.5	8.70 (7.27 to 10.41)	-0.200 (-1.92 to 1.25)	0.98 (0.82 to 1.17)
Slovenia	2021	Diseases of the nervous system	5,940	2.82	7,330 (6,950 to 7,730)	3.48 (3.30 to 3.67)	-1,390 (-1,790 to -1,020)	-0.659 (-0.847 to -0.481)	0.81 (0.77 to 0.85)	8.3	8.70 (6.75 to 11.21)	-0.400 (-2.91 to 1.58)	0.95 (0.74 to 1.23)
Slovenia	2020	Diseases of the eye and adnexa	2,910	1.38	4,270 (3,700 to 4,940)	2.03 (1.76 to 2.35)	-1,360 (-2,040 to -787)	-0.649 (-0.969 to -0.374)	0.68 (0.59 to 0.79)	3.8	4.87 (3.19 to 7.42)	-1.07 (-3.68 to 0.581)	0.78 (0.51 to 1.18)
Slovenia	2021	Diseases of the eye and adnexa	2,250	1.07	4,290 (3,490 to 5,270)	2.03 (1.65 to 2.50)	-2,040 (-3,030 to -1,240)	-0.966 (-1.44 to -0.590)	0.52 (0.43 to 0.64)	3.4	4.87 (3.19 to 7.42)	-1.47 (-4.06 to 0.230)	0.70 (0.46 to 1.07)
Slovenia	2020	Diseases of the ear and mastoid process	1,340	0.638	1,970 (1,840 to 2,100)	0.937 (0.877 to 1.00)	-629 (-765 to -501)	-0.299 (-0.364 to -0.239)	0.68 (0.64 to 0.73)	3.9	3.70 (3.38 to 4.05)	0.200 (-0.153 to 0.524)	1.05 (0.96 to 1.16)
Slovenia	2021	Diseases of the ear and mastoid process	1,380	0.654	2,010 (1,800 to 2,250)	0.954 (0.853 to 1.07)	-631 (-872 to -416)	-0.300 (-0.414 to -0.197)	0.69 (0.61 to 0.77)	3.5	3.70 (3.25 to 4.21)	-0.200 (-0.718 to 0.242)	0.95 (0.83 to 1.07)
Slovenia	2020	Diseases of the circulatory system	34,500	16.4	43,100 (40,900 to 45,500)	20.5 (19.5 to 21.7)	-8,670 (-11,000 to -6,430)	-4.12 (-5.24 to -3.06)	0.80 (0.76 to 0.84)	7.9	7.96 (7.61 to 8.32)	-0.058 (-0.422 to 0.293)	0.99 (0.95 to 1.04)
Slovenia	2021	Diseases of the circulatory system	35,400	16.8	43,300 (41,000 to 45,600)	20.5 (19.5 to 21.7)	-7,840 (-10,300 to -5,580)	-3.72 (-4.88 to -2.65)	0.82 (0.77 to 0.86)	7.4	7.96 (7.61 to 8.32)	-0.558 (-0.924 to -0.214)	0.93 (0.89 to 0.97)
Slovenia	2020	Diseases of the respiratory system	25,000	11.9	31,000 (30,000 to 32,000)	14.7 (14.3 to 15.2)	-6,030 (-7,040 to -5,040)	-2.87 (-3.35 to -2.40)	0.81 (0.78 to 0.83)	7.8	6.90 (6.49 to 7.33)	0.900 (0.484 to 1.31)	1.13 (1.07 to 1.20)
Slovenia	2021	Diseases of the respiratory system	31,000	14.7	31,600 (30,200 to 33,100)	15.0 (14.3 to 15.7)	-670 (-2,130 to 741)	-0.318 (-1.01 to 0.352)	0.98 (0.94 to 1.02)	9.1	6.90 (6.33 to 7.52)	2.20 (1.58 to 2.76)	1.32 (1.21 to 1.44)
Slovenia	2020	Diseases of the digestive system	22,800	10.8	28,200 (27,100 to 29,400)	13.4 (12.9 to 14.0)	-5,480 (-6,670 to -4,380)	-2.61 (-3.17 to -2.08)	0.81 (0.77 to 0.84)	5.5	5.60 (5.33 to 5.88)	-0.100 (-0.380 to 0.166)	0.98 (0.94 to 1.03)
Slovenia	2021	Diseases of the digestive system	22,800	10.8	28,600 (27,200 to 30,100)	13.6 (12.9 to 14.3)	-5,820 (-7,300 to -4,400)	-2.76 (-3.47 to -2.09)	0.80 (0.76 to 0.84)	5.2	5.60 (5.23 to 6.00)	-0.400 (-0.803 to -0.023)	0.93 (0.87 to 1.00)
Slovenia	2020	Diseases of the skin and subcutaneous tissue	2,800	1.33	4,100 (3,730 to 4,500)	1.95 (1.77 to 2.14)	-1,300 (-1,700 to -930)	-0.618 (-0.809 to -0.442)	0.68 (0.62 to 0.75)	7.2	7.06 (6.76 to 7.37)	0.142 (-0.178 to 0.441)	1.02 (0.98 to 1.07)
Slovenia	2021	Diseases of the skin and subcutaneous tissue	2,570	1.22	4,110 (3,600 to 4,690)	1.95 (1.71 to 2.23)	-1,530 (-2,130 to -1,020)	-0.727 (-1.01 to -0.485)	0.63 (0.55 to 0.72)	7.1	7.06 (6.76 to 7.37)	0.042 (-0.269 to 0.338)	1.01 (0.96 to 1.05)

Location	Year	Condition	Observed hospitalizations	Observed hospitalizations per 1,000 population	Expected hospitalizations	Expected hospitalizations per 1,000 population	Difference in hospitalizations	Difference in rate	Hospitalizations observed-to-expected ratio	Observed length of stay	Expected length of stay	Difference in length of stay	Length of stay observed-to-expected ratio
Slovenia	2020	Diseases of musculoskeletal system and connective tissue	17,700	8.41	23,100 (21,700 to 24,700)	11.0 (10.3 to 11.8)	-5,470 (-7,060 to -4,030)	-2.60 (-3.36 to -1.92)	0.76 (0.71 to 0.81)	5.5	5.84 (5.55 to 6.14)	-0.336 (-0.629 to -0.051)	0.94 (0.90 to 0.99)
Slovenia	2021	Diseases of musculoskeletal system and connective tissue	16,800	7.99	23,200 (21,200 to 25,500)	11.0 (10.0 to 12.1)	-6,370 (-8,650 to -4,330)	-3.02 (-4.10 to -2.06)	0.73 (0.66 to 0.80)	5.4	5.68 (5.29 to 6.09)	-0.276 (-0.694 to 0.112)	0.95 (0.89 to 1.02)
Slovenia	2020	Diseases of the genitourinary system	17,600	8.35	22,000 (20,800 to 23,200)	10.4 (9.89 to 11.0)	-4,410 (-5,670 to -3,220)	-2.10 (-2.70 to -1.53)	0.80 (0.76 to 0.84)	4.9	4.70 (4.46 to 4.96)	0.200 (-0.056 to 0.449)	1.04 (0.99 to 1.10)
Slovenia	2021	Diseases of the genitourinary system	18,300	8.68	22,000 (20,800 to 23,300)	10.4 (9.89 to 11.0)	-3,730 (-4,980 to -2,510)	-1.77 (-2.36 to -1.19)	0.83 (0.79 to 0.88)	4.6	4.70 (4.44 to 4.98)	-0.100 (-0.375 to 0.162)	0.98 (0.92 to 1.04)
Slovenia	2020	Pregnancy, childbirth and the puerperium	24,200	11.5	26,400 (25,200 to 27,800)	12.6 (12.0 to 13.2)	-2,260 (-3,590 to -1,000)	-1.08 (-1.71 to -0.477)	0.91 (0.87 to 0.96)	3.8	3.90 (3.66 to 4.16)	-0.100 (-0.359 to 0.144)	0.97 (0.91 to 1.04)
Slovenia	2021	Pregnancy, childbirth and the puerperium	24,600	11.6	26,500 (24,700 to 28,400)	12.6 (11.7 to 13.5)	-1,960 (-3,900 to -170)	-0.929 (-1.85 to -0.080)	0.93 (0.86 to 0.99)	3.7	3.90 (3.57 to 4.27)	-0.200 (-0.566 to 0.136)	0.95 (0.87 to 1.04)
Slovenia	2020	Certain conditions originating in the perinatal period	4,790	2.28	5,510 (4,530 to 6,700)	2.62 (2.16 to 3.19)	-726 (-1,920 to 238)	-0.345 (-0.914 to 0.113)	0.87 (0.71 to 1.05)	6.7	6.62 (6.15 to 7.12)	0.084 (-0.409 to 0.554)	1.01 (0.94 to 1.09)
Slovenia	2021	Certain conditions originating in the perinatal period	4,640	2.2	5,500 (4,360 to 6,950)	2.61 (2.07 to 3.30)	-863 (-2,290 to 261)	-0.409 (-1.09 to 0.124)	0.84 (0.67 to 1.06)	6.6	6.62 (6.15 to 7.12)	-0.016 (-0.532 to 0.446)	1.00 (0.93 to 1.07)
Slovenia	2020	Congenital malformations, deformations and chromosomal abnormalities	2,680	1.28	3,320 (1,810 to 6,090)	1.58 (0.861 to 2.90)	-638 (-3,340 to 858)	-0.304 (-1.59 to 0.408)	0.81 (0.45 to 1.47)	6	4.90 (4.16 to 5.77)	1.10 (0.231 to 1.85)	1.23 (1.04 to 1.45)
Slovenia	2021	Congenital malformations, deformations and chromosomal abnormalities	3,060	1.45	3,330 (1,410 to 7,850)	1.58 (0.670 to 3.72)	-270 (-4,770 to 1,650)	-0.128 (-2.26 to 0.782)	0.92 (0.39 to 2.17)	4.6	4.90 (4.16 to 5.77)	-0.295 (-1.15 to 0.457)	0.94 (0.80 to 1.11)
Slovenia	2020	Symptoms, signs and abnormal clinical and laboratory findings, n.e.c.	8,540	4.06	13,900 (10,800 to 17,900)	6.62 (5.13 to 8.53)	-5,370 (-9,330 to -2,290)	-2.55 (-4.44 to -1.09)	0.61 (0.48 to 0.79)	4.7	4.50 (4.15 to 4.88)	0.200 (-0.187 to 0.557)	1.04 (0.96 to 1.13)
Slovenia	2021	Symptoms, signs and abnormal clinical and laboratory findings, n.e.c.	9,470	4.49	14,000 (10,800 to 18,000)	6.62 (5.13 to 8.53)	-4,480 (-8,480 to -1,370)	-2.13 (-4.02 to -0.651)	0.68 (0.53 to 0.87)	4.1	4.50 (4.01 to 5.05)	-0.400 (-0.955 to 0.085)	0.91 (0.81 to 1.02)
Slovenia	2020	Injury, poisoning and other consequences of external causes	25,400	12.1	31,300 (29,400 to 33,300)	14.9 (14.0 to 15.8)	-5,930 (-7,930 to -4,020)	-2.82 (-3.77 to -1.91)	0.81 (0.76 to 0.86)	7.1	7.00 (6.48 to 7.56)	0.100 (-0.476 to 0.618)	1.01 (0.94 to 1.10)
Slovenia	2021	Injury, poisoning and other consequences of external causes	24,500	11.6	31,400 (29,500 to 33,400)	14.9 (14.0 to 15.8)	-6,850 (-8,820 to -5,010)	-3.25 (-4.18 to -2.38)	0.78 (0.74 to 0.83)	6.5	7.00 (6.28 to 7.80)	-0.500 (-1.31 to 0.225)	0.93 (0.83 to 1.04)
Slovenia	2020	Factors influencing health status and contact with health services	44,000	21	55,000 (44,800 to 67,600)	26.2 (21.3 to 32.1)	-11,000 (-23,800 to -689)	-5.21 (-11.3 to -0.328)	0.80 (0.65 to 0.98)	4.6	4.09 (3.66 to 4.58)	0.506 (0.030 to 0.940)	1.12 (1.01 to 1.26)

Location	Year	Condition	Observed hospitalizations	Observed hospitalizations per 1,000 population	Expected hospitalizations	Expected hospitalizations per 1,000 population	Difference in hospitalizations	Difference in rate	Hospitalizations observed-to-expected ratio	Observed length of stay	Expected length of stay	Difference in length of stay	Length of stay observed-to-expected ratio
Slovenia	2021	Factors influencing health status and contact with health services	52,400	24.8	55,200 (41,200 to 73,800)	26.2 (19.6 to 35.0)	-2,790 (-21,500 to 11,300)	-1.32 (-10.2 to 5.35)	0.95 (0.71 to 1.27)	4	4.09 (3.66 to 4.58)	-0.094 (-0.572 to 0.344)	0.98 (0.87 to 1.09)
Sweden	2020	All causes	1,280,000	124	1,380,000 (1,340,000 to 1,420,000)	133 (130 to 137)	-100,000 (-137,000 to -63,000)	-9.67 (-13.3 to -6.08)	0.93 (0.90 to 0.95)				
Sweden	2021	All causes	1,310,000	126	1,350,000 (1,300,000 to 1,410,000)	130 (125 to 135)	-39,200 (-90,800 to 11,000)	-3.76 (-8.72 to 1.05)	0.97 (0.94 to 1.01)				
Sweden	2020	Infectious and parasitic diseases	36,300	3.5	40,900 (40,300 to 41,500)	3.95 (3.90 to 4.01)	-4,680 (-5,260 to -4,080)	-0.452 (-0.508 to -0.394)	0.89 (0.87 to 0.90)	5.7	5.77 (5.53 to 6.03)	-0.074 (-0.327 to 0.173)	0.99 (0.95 to 1.03)
Sweden	2021	Infectious and parasitic diseases	37,800	3.63	39,900 (39,300 to 40,500)	3.83 (3.77 to 3.89)	-2,090 (-2,680 to -1,510)	-0.200 (-0.257 to -0.145)	0.95 (0.93 to 0.96)	5.8	5.77 (5.53 to 6.03)	0.026 (-0.233 to 0.273)	1.00 (0.96 to 1.05)
Sweden	2020	Neoplasms	84,800	8.19	89,800 (87,700 to 91,900)	8.67 (8.47 to 8.88)	-4,950 (-7,090 to -2,880)	-0.478 (-0.685 to -0.278)	0.94 (0.92 to 0.97)	6.2	6.50 (6.17 to 6.85)	-0.300 (-0.644 to 0.033)	0.95 (0.91 to 1.01)
Sweden	2021	Neoplasms	86,800	8.33	87,000 (84,200 to 90,000)	8.36 (8.08 to 8.64)	-224 (-3,210 to 2,640)	-0.022 (-0.309 to 0.254)	1.00 (0.96 to 1.03)	6.2	6.50 (6.04 to 7.00)	-0.300 (-0.797 to 0.153)	0.95 (0.89 to 1.03)
Sweden	2020	Diseases of the blood and bloodforming organs	10,800	1.04	11,600 (11,300 to 12,000)	1.12 (1.09 to 1.16)	-816 (-1,160 to -467)	-0.079 (-0.112 to -0.045)	0.93 (0.90 to 0.96)	4.5	4.50 (4.28 to 4.73)	0.000 (-0.239 to 0.219)	1.00 (0.95 to 1.05)
Sweden	2021	Diseases of the blood and bloodforming organs	11,500	1.1	11,300 (10,900 to 11,800)	1.09 (1.04 to 1.14)	132 (-376 to 606)	0.013 (-0.036 to 0.058)	1.01 (0.97 to 1.06)	4.4	4.50 (4.19 to 4.83)	-0.100 (-0.435 to 0.215)	0.98 (0.91 to 1.05)
Sweden	2020	Endocrine, nutritional and metabolic diseases	25,200	2.43	27,400 (25,500 to 29,500)	2.65 (2.46 to 2.85)	-2,260 (-4,390 to -310)	-0.219 (-0.424 to -0.030)	0.92 (0.85 to 0.99)	4.4	4.30 (3.77 to 4.91)	0.100 (-0.516 to 0.627)	1.02 (0.90 to 1.17)
Sweden	2021	Endocrine, nutritional and metabolic diseases	26,200	2.52	26,500 (23,900 to 29,400)	2.55 (2.29 to 2.82)	-289 (-3,200 to 2,320)	-0.028 (-0.307 to 0.222)	0.99 (0.89 to 1.10)	4.4	4.30 (3.56 to 5.19)	0.100 (-0.792 to 0.842)	1.02 (0.85 to 1.24)
Sweden	2020	Mental and behavioural disorders	94,100	9.08	99,700 (90,800 to 109,000)	9.63 (8.77 to 10.6)	-5,630 (-15,300 to 2,870)	-0.544 (-1.48 to 0.278)	0.94 (0.86 to 1.03)	15	15.53 (14.01 to 17.22)	-0.531 (-2.20 to 0.968)	0.97 (0.87 to 1.07)
Sweden	2021	Mental and behavioural disorders	94,100	9.03	100,000 (87,900 to 114,000)	9.63 (8.44 to 11.0)	-6,200 (-20,300 to 5,940)	-0.595 (-1.95 to 0.570)	0.94 (0.82 to 1.07)	15.6	15.53 (14.01 to 17.22)	0.069 (-1.61 to 1.60)	1.00 (0.91 to 1.11)
Sweden	2020	Diseases of the nervous system	32,800	3.17	35,200 (33,000 to 37,500)	3.40 (3.19 to 3.62)	-2,370 (-4,620 to -223)	-0.229 (-0.446 to -0.022)	0.93 (0.88 to 0.99)	4.7	5.16 (4.72 to 5.63)	-0.458 (-0.926 to -0.032)	0.91 (0.84 to 0.99)
Sweden	2021	Diseases of the nervous system	33,700	3.23	34,200 (31,200 to 37,400)	3.28 (3.00 to 3.59)	-507 (-3,730 to 2,410)	-0.049 (-0.358 to 0.231)	0.99 (0.90 to 1.08)	4.8	5.16 (4.72 to 5.63)	-0.358 (-0.841 to 0.069)	0.93 (0.85 to 1.01)
Sweden	2020	Diseases of the eye and adnexa	6,590	0.637	7,780 (7,200 to 8,420)	0.752 (0.695 to 0.813)	-1,190 (-1,830 to -599)	-0.115 (-0.177 to -0.058)	0.85 (0.78 to 0.92)	2.3	2.40 (2.30 to 2.50)	-0.100 (-0.202 to -0.001)	0.96 (0.92 to 1.00)
Sweden	2021	Diseases of the eye and adnexa	6,860	0.659	7,830 (7,010 to 8,740)	0.752 (0.673 to 0.839)	-970 (-1,910 to -156)	-0.093 (-0.184 to -0.015)	0.88 (0.78 to 0.98)	2.3	2.40 (2.26 to 2.55)	-0.100 (-0.244 to 0.037)	0.96 (0.90 to 1.02)
Sweden	2020	Diseases of the ear and mastoid process	5,730	0.553	6,610 (5,920 to 7,370)	0.638 (0.572 to 0.712)	-881 (-1,650 to -203)	-0.085 (-0.160 to -0.020)	0.87 (0.78 to 0.97)	2.2	2.30 (2.15 to 2.47)	-0.100 (-0.265 to 0.054)	0.96 (0.89 to 1.03)

Location	Year	Condition	Observed hospitalizations	Observed hospitalizations per 1,000 population	Expected hospitalizations	Expected hospitalizations per 1,000 population	Difference in hospitalizations	Difference in rate	Hospitalizations observed-to-expected ratio	Observed length of stay	Expected length of stay	Difference in length of stay	Length of stay observed-to-expected ratio
Sweden	2021	Diseases of the ear and mastoid process	5,750	0.552	6,650 (5,690 to 7,760)	0.638 (0.547 to 0.745)	-898 (-2,000 to 69)	-0.086 (-0.192 to 0.007)	0.86 (0.74 to 1.01)	2.2	2.30 (2.09 to 2.54)	-0.100 (-0.335 to 0.115)	0.96 (0.87 to 1.06)
Sweden	2020	Diseases of the circulatory system	162,000	15.6	171,000 (166,000 to 175,000)	16.5 (16.0 to 16.9)	-8,790 (-13,800 to -3,880)	-0.849 (-1.34 to -0.375)	0.95 (0.92 to 0.98)	5.1	5.21 (5.01 to 5.42)	-0.107 (-0.310 to 0.094)	0.98 (0.94 to 1.02)
Sweden	2021	Diseases of the circulatory system	167,000	16.1	165,000 (159,000 to 172,000)	15.9 (15.3 to 16.5)	1,970 (-4,850 to 8,340)	0.189 (-0.466 to 0.801)	1.01 (0.97 to 1.05)	5.1	5.12 (4.84 to 5.41)	-0.015 (-0.300 to 0.257)	1.00 (0.94 to 1.05)
Sweden	2020	Diseases of the respiratory system	69,500	6.71	99,600 (92,100 to 108,000)	9.62 (8.89 to 10.4)	-30,200 (-38,400 to -22,700)	-2.91 (-3.71 to -2.19)	0.70 (0.64 to 0.75)	5.1	5.00 (4.80 to 5.22)	0.098 (-0.116 to 0.305)	1.02 (0.98 to 1.06)
Sweden	2021	Diseases of the respiratory system	69,500	6.67	100,000 (89,700 to 112,000)	9.62 (8.61 to 10.8)	-30,800 (-42,700 to -20,400)	-2.96 (-4.10 to -1.96)	0.69 (0.62 to 0.77)	5.3	4.91 (4.46 to 5.39)	0.394 (-0.076 to 0.837)	1.08 (0.99 to 1.19)
Sweden	2020	Diseases of the digestive system	102,000	9.9	110,000 (104,000 to 117,000)	10.6 (10.1 to 11.3)	-7,710 (-14,000 to -1,700)	-0.744 (-1.36 to -0.164)	0.93 (0.88 to 0.98)	4.2	4.40 (4.21 to 4.59)	-0.200 (-0.398 to -0.010)	0.95 (0.91 to 1.00)
Sweden	2021	Diseases of the digestive system	107,000	10.3	111,000 (102,000 to 120,000)	10.6 (9.83 to 11.5)	-3,910 (-13,200 to 4,510)	-0.376 (-1.27 to 0.433)	0.96 (0.89 to 1.04)	4.3	4.40 (4.14 to 4.68)	-0.100 (-0.375 to 0.164)	0.98 (0.92 to 1.04)
Sweden	2020	Diseases of the skin and subcutaneous tissue	9,660	0.933	11,000 (9,890 to 12,200)	1.06 (0.955 to 1.18)	-1,330 (-2,510 to -228)	-0.129 (-0.242 to -0.022)	0.88 (0.79 to 0.98)	5.8	6.10 (5.66 to 6.57)	-0.300 (-0.764 to 0.137)	0.95 (0.88 to 1.02)
Sweden	2021	Diseases of the skin and subcutaneous tissue	9,610	0.923	11,100 (9,520 to 12,800)	1.06 (0.914 to 1.23)	-1,440 (-3,180 to 74)	-0.138 (-0.305 to 0.007)	0.87 (0.75 to 1.01)	6.1	6.10 (5.49 to 6.77)	0.000 (-0.671 to 0.606)	1.00 (0.90 to 1.11)
Sweden	2020	Diseases of musculoskeletal system and connective tissue	64,800	6.26	81,300 (74,600 to 88,500)	7.85 (7.21 to 8.55)	-16,400 (-23,700 to -9,710)	-1.59 (-2.28 to -0.938)	0.80 (0.73 to 0.87)	3.4	3.30 (3.08 to 3.53)	0.101 (-0.128 to 0.320)	1.03 (0.96 to 1.10)
Sweden	2021	Diseases of musculoskeletal system and connective tissue	66,000	6.33	81,700 (72,400 to 92,200)	7.85 (6.96 to 8.86)	-15,800 (-26,300 to -6,300)	-1.51 (-2.52 to -0.605)	0.81 (0.72 to 0.91)	3.3	3.11 (2.83 to 3.42)	0.191 (-0.120 to 0.476)	1.06 (0.97 to 1.17)
Sweden	2020	Diseases of the genitourinary system	65,400	6.32	70,300 (67,100 to 73,700)	6.79 (6.48 to 7.12)	-4,870 (-8,270 to -1,670)	-0.470 (-0.799 to -0.161)	0.93 (0.89 to 0.98)	4.1	4.10 (3.91 to 4.30)	0.000 (-0.200 to 0.191)	1.00 (0.95 to 1.05)
Sweden	2021	Diseases of the genitourinary system	68,400	6.57	70,700 (66,200 to 75,600)	6.79 (6.35 to 7.26)	-2,290 (-7,120 to 2,380)	-0.220 (-0.684 to 0.229)	0.97 (0.91 to 1.04)	4.1	4.10 (3.83 to 4.39)	0.000 (-0.290 to 0.267)	1.00 (0.93 to 1.07)
Sweden	2020	Pregnancy, childbirth and the puerperium	119,000	11.5	122,000 (117,000 to 127,000)	11.8 (11.3 to 12.3)	-3,140 (-8,400 to 1,980)	-0.303 (-0.811 to 0.191)	0.97 (0.93 to 1.02)	2.5	2.50 (2.36 to 2.65)	0.000 (-0.147 to 0.142)	1.00 (0.94 to 1.06)
Sweden	2021	Pregnancy, childbirth and the puerperium	121,000	11.6	121,000 (113,000 to 128,000)	11.6 (10.9 to 12.3)	560 (-6,960 to 7,640)	0.054 (-0.668 to 0.733)	1.00 (0.95 to 1.07)	2.5	2.50 (2.30 to 2.71)	0.000 (-0.215 to 0.191)	1.00 (0.92 to 1.08)
Sweden	2020	Certain conditions originating in the perinatal period	17,200	1.66	17,500 (16,100 to 19,100)	1.69 (1.56 to 1.84)	-353 (-1,890 to 1,070)	-0.034 (-0.183 to 0.103)	0.98 (0.90 to 1.07)	9.1	9.00 (8.36 to 9.69)	0.100 (-0.590 to 0.756)	1.01 (0.94 to 1.09)
Sweden	2021	Certain conditions originating in the perinatal period	17,400	1.67	17,600 (15,700 to 19,800)	1.69 (1.50 to 1.91)	-188 (-2,440 to 1,810)	-0.018 (-0.234 to 0.174)	0.99 (0.88 to 1.12)	9.2	8.71 (7.84 to 9.68)	0.490 (-0.454 to 1.36)	1.06 (0.95 to 1.17)
Sweden	2020	Congenital malformations, deformations and chromosomal abnormalities	6,140	0.593	6,880 (6,490 to 7,300)	0.665 (0.627 to 0.705)	-750 (-1,170 to -352)	-0.072 (-0.113 to -0.034)	0.89 (0.84 to 0.95)	5.7	5.65 (5.21 to 6.12)	0.054 (-0.424 to 0.484)	1.01 (0.93 to 1.09)

Location	Year	Condition	Observed hospitalizations	Observed hospitalizations per 1,000 population	Expected hospitalizations	Expected hospitalizations per 1,000 population	Difference in hospitalizations	Difference in rate	Hospitalizations observed-to-expected ratio	Observed length of stay	Expected length of stay	Difference in length of stay	Length of stay observed-to-expected ratio
Sweden	2021	Congenital malformations, deformations and chromosomal abnormalities	6,150	0.591	6,620 (6,090 to 7,190)	0.635 (0.584 to 0.690)	-465 (-1,040 to 52)	-0.045 (-0.099 to 0.005)	0.93 (0.86 to 1.01)	5.7	5.65 (5.21 to 6.12)	0.054 (-0.418 to 0.498)	1.01 (0.93 to 1.10)
Sweden	2020	Symptoms, signs and abnormal clinical and laboratory findings, n.e.c.	109,000	10.6	110,000 (104,000 to 116,000)	10.6 (10.1 to 11.2)	-830 (-7,060 to 4,950)	-0.080 (-0.682 to 0.478)	0.99 (0.94 to 1.05)	3	3.10 (2.99 to 3.21)	-0.100 (-0.210 to 0.004)	0.97 (0.93 to 1.00)
Sweden	2021	Symptoms, signs and abnormal clinical and laboratory findings, n.e.c.	119,000	11.4	107,000 (98,900 to 115,000)	10.3 (9.50 to 11.1)	12,300 (3,820 to 20,300)	1.18 (0.367 to 1.95)	1.11 (1.03 to 1.21)	3.8	3.10 (2.95 to 3.26)	0.700 (0.543 to 0.847)	1.23 (1.17 to 1.29)
Sweden	2020	Injury, poisoning and other consequences of external causes	121,000	11.7	125,000 (121,000 to 130,000)	12.1 (11.7 to 12.5)	-4,150 (-8,440 to 42)	-0.401 (-0.815 to 0.004)	0.97 (0.93 to 1.00)	4.7	4.90 (4.50 to 5.34)	-0.200 (-0.635 to 0.205)	0.96 (0.88 to 1.05)
Sweden	2021	Injury, poisoning and other consequences of external causes	124,000	11.9	123,000 (117,000 to 129,000)	11.8 (11.3 to 12.4)	1,010 (-5,130 to 6,610)	0.097 (-0.492 to 0.635)	1.01 (0.96 to 1.06)	4.7	4.90 (4.34 to 5.53)	-0.200 (-0.839 to 0.355)	0.96 (0.85 to 1.08)
Sweden	2020	Factors influencing health status and contact with health services	138,000	13.3	147,000 (142,000 to 152,000)	14.2 (13.7 to 14.6)	-9,070 (-14,000 to -4,370)	-0.876 (-1.36 to -0.422)	0.94 (0.91 to 0.97)	6.3	6.53 (4.90 to 8.71)	-0.231 (-2.42 to 1.39)	0.96 (0.72 to 1.28)
Sweden	2021	Factors influencing health status and contact with health services	135,000	13	145,000 (138,000 to 152,000)	13.9 (13.3 to 14.6)	-9,360 (-16,100 to -2,850)	-0.899 (-1.55 to -0.274)	0.94 (0.89 to 0.98)	6.3	6.53 (4.90 to 8.71)	-0.231 (-2.42 to 1.44)	0.96 (0.72 to 1.30)

Table S6. Expected and observed avoidable hospital admissions age-sex-standardized rates by country and sex, 2020-2021

Country	Hospital admission	Sex	2020 Results				2021 Results			
			Observed rate	Expected rate	Rate difference	Observed-to-expected ratio	Observed rate	Expected rate	Rate difference	Observed-to-expected ratio
Austria	Asthma and COPD	Total	157	221 (207 to 235)	-63.4 (-78.0 to -49.8)	0.71 (0.67 to 0.76)	144	211 (193 to 231)	-67.4 (-87.3 to -49.0)	0.68 (0.62 to 0.75)
Austria	Asthma and COPD	Female	129	186 (177 to 194)	-56.1 (-64.9 to -47.6)	0.70 (0.67 to 0.73)	123	179 (167 to 191)	-55.2 (-67.6 to -43.7)	0.69 (0.65 to 0.74)
Austria	Asthma and COPD	Male	194	268 (247 to 292)	-74.3 (-98.3 to -52.6)	0.72 (0.66 to 0.79)	172	256 (228 to 289)	-84.3 (-117 to -54.6)	0.67 (0.60 to 0.76)
Austria	Asthma	Total	14.7	20.6 (19.0 to 22.4)	-5.91 (-7.74 to -4.32)	0.71 (0.66 to 0.77)	14.3	19.5 (17.3 to 21.9)	-5.19 (-7.62 to -3.05)	0.73 (0.65 to 0.82)
Austria	Asthma	Female	16.4	23.3 (21.3 to 25.6)	-6.95 (-9.22 to -4.84)	0.70 (0.64 to 0.77)	16.4	22.0 (19.3 to 25.1)	-5.58 (-8.62 to -2.89)	0.75 (0.66 to 0.85)
Austria	Asthma	Male	12.8	17.3 (15.7 to 19.1)	-4.50 (-6.31 to -2.87)	0.74 (0.67 to 0.82)	12	16.4 (14.3 to 18.9)	-4.45 (-6.89 to -2.36)	0.73 (0.64 to 0.84)
Austria	COPD	Total	142	200 (186 to 215)	-57.5 (-72.8 to -43.1)	0.71 (0.66 to 0.77)	130	192 (173 to 213)	-62.3 (-82.7 to -43.8)	0.68 (0.61 to 0.75)
Austria	COPD	Female	113	162 (154 to 171)	-49.2 (-58.2 to -40.7)	0.70 (0.66 to 0.74)	107	157 (145 to 169)	-49.7 (-62.3 to -38.1)	0.68 (0.63 to 0.74)
Austria	COPD	Male	181	251 (229 to 275)	-69.9 (-94.4 to -47.4)	0.72 (0.66 to 0.79)	160	240 (211 to 273)	-79.9 (-113 to -50.1)	0.67 (0.59 to 0.76)
Austria	Congestive heart failure and hypertension	Total	358	432 (402 to 464)	-73.7 (-106 to -44.0)	0.83 (0.77 to 0.89)	366	417 (376 to 462)	-51.3 (-95.9 to -10.3)	0.88 (0.79 to 0.97)
Austria	Congestive heart failure and hypertension	Female	346	425 (393 to 461)	-78.9 (-115 to -46.4)	0.81 (0.75 to 0.88)	352	411 (367 to 459)	-58.6 (-107 to -15.2)	0.86 (0.77 to 0.96)
Austria	Congestive heart failure and hypertension	Male	370	437 (408 to 468)	-67.1 (-98.8 to -38.4)	0.85 (0.79 to 0.91)	380	422 (383 to 466)	-42.6 (-86.5 to -2.72)	0.90 (0.81 to 0.99)
Austria	Congestive heart failure	Total	207	243 (229 to 257)	-35.7 (-50.0 to -22.3)	0.85 (0.81 to 0.90)	220	238 (220 to 258)	-18.5 (-38.3 to -0.768)	0.92 (0.85 to 1.00)
Austria	Congestive heart failure	Female	170	204 (189 to 219)	-33.3 (-48.7 to -19.3)	0.84 (0.78 to 0.90)	182	204 (184 to 226)	-21.3 (-43.6 to -1.11)	0.90 (0.81 to 0.99)
Austria	Congestive heart failure	Male	257	308 (288 to 330)	-51.5 (-72.9 to -31.6)	0.83 (0.78 to 0.89)	270	308 (280 to 339)	-38.0 (-68.9 to -10.4)	0.88 (0.80 to 0.96)
Austria	Diabetes	Total	119	151 (144 to 157)	-32.1 (-38.8 to -25.7)	0.79 (0.75 to 0.82)	120	141 (133 to 150)	-21.5 (-30.2 to -13.3)	0.85 (0.80 to 0.90)
Austria	Diabetes	Female	93.5	118 (112 to 125)	-24.9 (-31.9 to -18.4)	0.79 (0.75 to 0.84)	93.9	110 (102 to 119)	-16.2 (-25.5 to -7.68)	0.85 (0.79 to 0.92)
Austria	Diabetes	Male	147	188 (180 to 196)	-40.7 (-49.1 to -32.6)	0.78 (0.75 to 0.82)	148	176 (166 to 188)	-28.2 (-39.5 to -17.3)	0.84 (0.79 to 0.90)
Austria	Hypertension	Total	151	190 (170 to 211)	-38.6 (-60.1 to -18.7)	0.80 (0.72 to 0.89)	146	180 (154 to 210)	-34.0 (-63.3 to -8.90)	0.81 (0.70 to 0.94)
Austria	Hypertension	Female	176	226 (203 to 252)	-49.8 (-76.1 to -26.4)	0.78 (0.70 to 0.87)	170	215 (185 to 251)	-45.8 (-80.7 to -14.9)	0.79 (0.68 to 0.92)
Austria	Hypertension	Male	113	135 (120 to 152)	-21.9 (-39.4 to -6.41)	0.84 (0.74 to 0.95)	110	127 (107 to 150)	-17.3 (-41.1 to 2.50)	0.86 (0.73 to 1.02)
Belgium	Asthma and COPD	Total	206	322 (229 to 455)	-117 (-247 to -22.3)	0.64 (0.45 to 0.90)	194	322 (198 to 524)	-128 (-328 to -3.47)	0.60 (0.37 to 0.98)

Belgium	Asthma and COPD	Female	172	276 (204 to 373)	-104 (-203 to -32.2)	0.62 (0.46 to 0.84)	171	276 (180 to 423)	-105 (-251 to -7.85)	0.62 (0.41 to 0.96)
Belgium	Asthma and COPD	Male	257	395 (268 to 582)	-138 (-327 to -11.2)	0.65 (0.44 to 0.96)	232	395 (228 to 683)	-163 (-447 to 3.77)	0.59 (0.34 to 1.02)
Belgium	Asthma	Total	15.7	27.4 (23.8 to 31.5)	-11.7 (-15.8 to -8.22)	0.57 (0.50 to 0.66)	16.2	27.4 (22.5 to 33.4)	-11.2 (-17.0 to -6.26)	0.59 (0.49 to 0.72)
Belgium	Asthma	Female	18.6	32.9 (27.8 to 38.9)	-14.3 (-20.2 to -9.19)	0.57 (0.48 to 0.67)	18.5	32.9 (26.0 to 41.6)	-14.4 (-23.2 to -7.73)	0.56 (0.44 to 0.71)
Belgium	Asthma	Male	12.4	20.8 (17.8 to 24.4)	-8.40 (-11.9 to -5.35)	0.60 (0.51 to 0.70)	13.4	20.8 (16.6 to 26.0)	-7.40 (-12.6 to -3.19)	0.64 (0.51 to 0.81)
Belgium	COPD	Total	190	295 (193 to 450)	-105 (-263 to -3.93)	0.64 (0.42 to 0.98)	178	295 (162 to 537)	-116 (-361 to 15.2)	0.60 (0.33 to 1.09)
Belgium	COPD	Female	153	243 (161 to 367)	-90.1 (-213 to -7.27)	0.63 (0.42 to 0.95)	153	243 (136 to 436)	-90.2 (-283 to 17.5)	0.63 (0.35 to 1.13)
Belgium	COPD	Male	244	374 (241 to 580)	-130 (-336 to 4.45)	0.65 (0.42 to 1.02)	218	374 (201 to 695)	-156 (-475 to 18.9)	0.58 (0.31 to 1.10)
Belgium	Congestive heart failure and hypertension	Total	154	200 (170 to 234)	-45.6 (-80.9 to -16.4)	0.77 (0.66 to 0.90)	163	200 (160 to 250)	-36.5 (-87.4 to 3.34)	0.82 (0.65 to 1.02)
Belgium	Congestive heart failure and hypertension	Female	136	176 (159 to 195)	-40.5 (-59.7 to -23.5)	0.77 (0.69 to 0.85)	145	176 (159 to 195)	-30.9 (-49.1 to -14.1)	0.82 (0.75 to 0.91)
Belgium	Congestive heart failure and hypertension	Male	179	232 (188 to 285)	-52.2 (-104 to -9.61)	0.77 (0.63 to 0.95)	186	232 (173 to 310)	-45.3 (-125 to 12.2)	0.80 (0.60 to 1.07)
Belgium	Congestive heart failure	Total	132	171 (129 to 227)	-38.3 (-94.0 to 4.00)	0.78 (0.58 to 1.03)	142	171 (114 to 255)	-29.3 (-114 to 25.7)	0.83 (0.55 to 1.22)
Belgium	Congestive heart failure	Female	114	146 (112 to 192)	-32.7 (-78.1 to 2.17)	0.78 (0.59 to 1.02)	124	146 (99.7 to 214)	-22.2 (-89.4 to 24.3)	0.85 (0.58 to 1.24)
Belgium	Congestive heart failure	Male	159	204 (152 to 274)	-45.4 (-114 to 7.09)	0.78 (0.58 to 1.05)	164	204 (134 to 310)	-39.5 (-146 to 29.9)	0.81 (0.53 to 1.22)
Belgium	Diabetes	Total	128	133 (127 to 138)	-5.05 (-10.9 to 0.303)	0.96 (0.92 to 1.00)	136	127 (120 to 135)	9.17 (1.14 to 16.5)	1.07 (1.01 to 1.14)
Belgium	Diabetes	Female	92	104 (97.8 to 110)	-11.6 (-17.5 to -5.83)	0.89 (0.84 to 0.94)	99.1	98.4 (90.8 to 107)	0.727 (-7.52 to 8.29)	1.01 (0.93 to 1.09)
Belgium	Diabetes	Male	170	167 (160 to 176)	2.21 (-6.01 to 10.2)	1.01 (0.97 to 1.06)	181	162 (151 to 173)	19.2 (7.69 to 29.8)	1.12 (1.04 to 1.20)
Belgium	Hypertension	Total	21.6	28.9 (14.9 to 56.1)	-7.30 (-35.0 to 6.35)	0.75 (0.38 to 1.42)	21.7	28.9 (11.3 to 73.8)	-7.20 (-51.7 to 10.4)	0.75 (0.30 to 1.91)
Belgium	Hypertension	Female	22.1	29.8 (14.6 to 60.8)	-7.70 (-38.2 to 7.62)	0.74 (0.37 to 1.53)	21.2	29.8 (10.9 to 81.8)	-8.60 (-59.3 to 10.4)	0.71 (0.26 to 1.97)
Belgium	Hypertension	Male	20.8	27.5 (15.1 to 50.0)	-6.70 (-29.1 to 5.72)	0.76 (0.42 to 1.38)	21.7	27.5 (11.8 to 64.1)	-5.80 (-43.5 to 9.84)	0.79 (0.33 to 1.83)
Canada	Asthma and COPD	Total	125	244 (228 to 260)	-118 (-135 to -102)	0.51 (0.48 to 0.55)	132	244 (221 to 268)	-111 (-135 to -89.0)	0.54 (0.50 to 0.60)
Canada	Asthma and COPD	Female	119	260 (245 to 276)	-141 (-156 to -126)	0.46 (0.43 to 0.49)	130	260 (245 to 276)	-131 (-147 to -116)	0.50 (0.47 to 0.53)
Canada	Asthma and COPD	Male	137	250 (231 to 271)	-113 (-134 to -93.6)	0.55 (0.51 to 0.59)	139	250 (223 to 280)	-111 (-142 to -84.7)	0.56 (0.50 to 0.62)
Canada	Asthma	Total	6.7	14.7 (13.3 to 16.2)	-7.98 (-9.46 to -6.66)	0.46 (0.41 to 0.50)	7.7	14.1 (12.7 to 15.5)	-6.36 (-7.79 to -5.05)	0.55 (0.50 to 0.60)

Canada	Asthma	Female	8.6	18.8 (16.8 to 21.0)	-10.2 (-12.4 to -8.25)	0.46 (0.41 to 0.51)	10	18.8 (16.1 to 22.0)	-8.80 (-12.1 to -6.07)	0.53 (0.45 to 0.62)
Canada	Asthma	Male	4.5	8.40 (7.42 to 9.51)	-3.90 (-5.00 to -2.93)	0.54 (0.47 to 0.61)	5	8.40 (7.05 to 10.0)	-3.40 (-4.97 to -2.03)	0.60 (0.50 to 0.71)
Canada	COPD	Total	119	230 (215 to 245)	-111 (-126 to -96.1)	0.52 (0.48 to 0.55)	125	230 (209 to 252)	-105 (-127 to -84.7)	0.54 (0.49 to 0.60)
Canada	COPD	Female	111	240 (226 to 254)	-129 (-144 to -116)	0.46 (0.43 to 0.49)	120	240 (226 to 254)	-120 (-135 to -107)	0.50 (0.47 to 0.53)
Canada	COPD	Male	133	242 (223 to 262)	-109 (-130 to -90.6)	0.55 (0.51 to 0.59)	134	242 (216 to 271)	-108 (-137 to -82.0)	0.55 (0.50 to 0.62)
Canada	Congestive heart failure and hypertension	Total	168	199 (189 to 210)	-31.7 (-42.4 to -21.7)	0.84 (0.80 to 0.89)	174	199 (189 to 210)	-25.8 (-36.6 to -15.7)	0.87 (0.83 to 0.92)
Canada	Congestive heart failure and hypertension	Female	148	176 (167 to 184)	-27.3 (-35.7 to -19.2)	0.84 (0.81 to 0.89)	156	176 (167 to 184)	-19.5 (-27.8 to -11.6)	0.89 (0.85 to 0.93)
Canada	Congestive heart failure and hypertension	Male	192	222 (207 to 238)	-29.4 (-45.5 to -14.2)	0.87 (0.81 to 0.93)	195	222 (201 to 245)	-26.9 (-50.0 to -6.27)	0.88 (0.80 to 0.97)
Canada	Congestive heart failure	Total	154	184 (174 to 194)	-29.4 (-39.4 to -19.9)	0.84 (0.80 to 0.89)	161	184 (174 to 194)	-22.5 (-32.8 to -12.9)	0.88 (0.83 to 0.93)
Canada	Congestive heart failure	Female	134	159 (152 to 167)	-24.7 (-32.6 to -17.3)	0.84 (0.80 to 0.89)	143	159 (152 to 167)	-15.7 (-23.7 to -8.10)	0.90 (0.86 to 0.95)
Canada	Congestive heart failure	Male	181	209 (194 to 225)	-28.1 (-43.7 to -13.7)	0.87 (0.81 to 0.93)	184	209 (189 to 232)	-25.0 (-47.7 to -4.23)	0.88 (0.79 to 0.98)
Canada	Diabetes	Total	94.1	98.7 (94.7 to 103)	-4.60 (-8.74 to -0.585)	0.95 (0.92 to 0.99)	93.8	98.7 (94.7 to 103)	-4.90 (-9.05 to -0.883)	0.95 (0.91 to 0.99)
Canada	Diabetes	Female	74.8	80.1 (75.9 to 84.6)	-5.30 (-9.81 to -1.00)	0.93 (0.88 to 0.99)	75.3	80.1 (75.9 to 84.6)	-4.80 (-9.27 to -0.562)	0.94 (0.89 to 0.99)
Canada	Diabetes	Male	118	121 (117 to 126)	-3.92 (-8.86 to 0.699)	0.97 (0.93 to 1.01)	116	121 (117 to 126)	-5.42 (-10.2 to -0.862)	0.96 (0.92 to 0.99)
Canada	Hypertension	Total	13.2	14.6 (13.6 to 15.7)	-1.40 (-2.46 to -0.422)	0.90 (0.84 to 0.97)	12.3	14.6 (13.2 to 16.1)	-2.30 (-3.83 to -0.935)	0.84 (0.76 to 0.93)
Canada	Hypertension	Female	13.9	15.3 (14.0 to 16.8)	-1.40 (-2.82 to -0.114)	0.91 (0.83 to 0.99)	12.7	15.3 (13.5 to 17.4)	-2.60 (-4.68 to -0.785)	0.83 (0.73 to 0.94)
Canada	Hypertension	Male	11.5	12.8 (12.2 to 13.4)	-1.28 (-1.88 to -0.722)	0.90 (0.86 to 0.94)	11	12.9 (12.2 to 13.5)	-1.85 (-2.49 to -1.24)	0.86 (0.82 to 0.90)
Switzerland	Asthma and COPD	Total	128	176 (150 to 206)	-48.5 (-78.9 to -23.1)	0.72 (0.62 to 0.85)	123	176 (141 to 220)	-53.1 (-96.3 to -18.7)	0.70 (0.56 to 0.87)
Switzerland	Asthma and COPD	Female	120	170 (145 to 201)	-50.4 (-80.0 to -24.4)	0.70 (0.60 to 0.83)	119	170 (135 to 215)	-51.5 (-97.3 to -16.8)	0.70 (0.55 to 0.88)
Switzerland	Asthma and COPD	Male	139	187 (159 to 220)	-47.6 (-80.5 to -19.0)	0.75 (0.63 to 0.88)	130	187 (149 to 235)	-57.0 (-106 to -19.2)	0.70 (0.55 to 0.87)
Switzerland	Asthma	Total	20.5	24.5 (22.2 to 27.1)	-4.00 (-6.57 to -1.65)	0.84 (0.76 to 0.93)	19.3	24.5 (21.3 to 28.2)	-5.20 (-8.85 to -1.87)	0.79 (0.69 to 0.91)
Switzerland	Asthma	Female	25.4	31.9 (28.9 to 35.2)	-6.50 (-9.76 to -3.47)	0.80 (0.72 to 0.88)	22.2	31.9 (27.8 to 36.7)	-9.70 (-14.4 to -5.55)	0.70 (0.61 to 0.80)
Switzerland	Asthma	Male	15	15.9 (13.4 to 18.9)	-0.900 (-3.86 to 1.61)	0.94 (0.80 to 1.12)	16.1	15.9 (12.5 to 20.3)	0.200 (-4.28 to 3.58)	1.01 (0.79 to 1.29)
Switzerland	COPD	Total	107	151 (125 to 183)	-44.4 (-76.6 to -18.1)	0.71 (0.58 to 0.86)	104	151 (116 to 198)	-47.8 (-95.5 to -12.0)	0.68 (0.52 to 0.90)

Switzerland	COPD	Female	94.6	148 (124 to 177)	-53.3 (-82.0 to -29.6)	0.64 (0.54 to 0.76)	96.7	158 (123 to 203)	-61.2 (-108 to -26.5)	0.61 (0.47 to 0.78)
Switzerland	COPD	Male	124	171 (143 to 205)	-46.7 (-80.0 to -18.7)	0.73 (0.61 to 0.87)	114	171 (133 to 220)	-57.2 (-106 to -18.8)	0.67 (0.52 to 0.86)
Switzerland	Congestive heart failure and hypertension	Total	248	294 (273 to 318)	-46.1 (-69.0 to -24.4)	0.84 (0.78 to 0.91)	263	302 (271 to 337)	-39.5 (-73.7 to -8.75)	0.87 (0.78 to 0.97)
Switzerland	Congestive heart failure and hypertension	Female	222	268 (247 to 291)	-46.5 (-69.2 to -25.8)	0.83 (0.76 to 0.90)	237	276 (245 to 310)	-38.9 (-73.7 to -8.76)	0.86 (0.76 to 0.96)
Switzerland	Congestive heart failure and hypertension	Male	286	324 (298 to 353)	-37.9 (-67.3 to -10.7)	0.88 (0.81 to 0.96)	299	324 (287 to 366)	-25.0 (-66.8 to 11.6)	0.92 (0.82 to 1.04)
Switzerland	Congestive heart failure	Total	203	245 (224 to 268)	-41.5 (-64.5 to -20.6)	0.83 (0.76 to 0.91)	220	254 (224 to 288)	-34.2 (-68.4 to -4.13)	0.87 (0.76 to 0.98)
Switzerland	Congestive heart failure	Female	167	207 (188 to 227)	-39.6 (-60.3 to -20.9)	0.81 (0.73 to 0.89)	184	215 (188 to 246)	-30.6 (-61.7 to -3.30)	0.86 (0.75 to 0.98)
Switzerland	Congestive heart failure	Male	257	301 (276 to 328)	-44.1 (-71.8 to -19.7)	0.85 (0.78 to 0.93)	270	311 (275 to 351)	-40.4 (-81.3 to -4.38)	0.87 (0.77 to 0.98)
Switzerland	Diabetes	Total	76.8	86.9 (79.1 to 95.5)	-10.1 (-18.7 to -2.11)	0.88 (0.80 to 0.97)	81.6	86.9 (76.0 to 99.3)	-5.30 (-17.8 to 5.44)	0.94 (0.82 to 1.07)
Switzerland	Diabetes	Female	49.7	59.0 (53.1 to 65.5)	-9.30 (-15.7 to -3.45)	0.84 (0.76 to 0.94)	52.7	59.0 (50.9 to 68.4)	-6.30 (-15.7 to 1.78)	0.89 (0.77 to 1.03)
Switzerland	Diabetes	Male	110	121 (107 to 137)	-10.8 (-26.7 to 3.15)	0.91 (0.81 to 1.03)	117	121 (102 to 144)	-4.10 (-26.7 to 15.2)	0.97 (0.81 to 1.15)
Switzerland	Hypertension	Total	44.9	50.6 (46.4 to 55.2)	-5.70 (-10.2 to -1.57)	0.89 (0.81 to 0.97)	43	50.6 (44.8 to 57.2)	-7.60 (-14.2 to -1.86)	0.85 (0.75 to 0.96)
Switzerland	Hypertension	Female	54.6	62.7 (59.0 to 66.6)	-8.10 (-12.0 to -4.40)	0.87 (0.82 to 0.93)	52.2	62.7 (59.0 to 66.6)	-10.5 (-14.5 to -6.88)	0.83 (0.78 to 0.88)
Switzerland	Hypertension	Male	29.5	33.0 (29.3 to 37.1)	-3.50 (-7.62 to 0.245)	0.89 (0.79 to 1.01)	28.6	33.0 (27.9 to 39.0)	-4.40 (-10.7 to 0.647)	0.87 (0.73 to 1.02)
Chile	Asthma	Total	8.7	18.2 (15.4 to 21.6)	-9.54 (-12.9 to -6.68)	0.48 (0.40 to 0.57)	8.4	18.2 (15.4 to 21.6)	-9.84 (-13.3 to -6.98)	0.46 (0.39 to 0.55)
Chile	Asthma	Female	10.6	23.6 (19.8 to 28.1)	-13.0 (-17.5 to -9.22)	0.45 (0.38 to 0.53)	10.3	23.6 (19.8 to 28.1)	-13.3 (-17.8 to -9.52)	0.44 (0.37 to 0.52)
Chile	Asthma	Male	6.7	12.5 (10.3 to 15.1)	-5.78 (-8.39 to -3.61)	0.54 (0.44 to 0.65)	6.5	12.5 (10.3 to 15.1)	-5.98 (-8.54 to -3.81)	0.52 (0.43 to 0.63)
Chile	COPD	Total	35.4	67.2 (59.1 to 76.4)	-31.8 (-41.2 to -23.8)	0.53 (0.46 to 0.60)	31.4	63.7 (53.2 to 76.4)	-32.3 (-44.8 to -21.8)	0.49 (0.41 to 0.59)
Chile	COPD	Female	29.2	62.8 (54.7 to 72.1)	-33.6 (-43.0 to -25.7)	0.46 (0.40 to 0.53)	27.3	59.5 (49.0 to 72.3)	-32.2 (-44.9 to -21.6)	0.46 (0.38 to 0.56)
Chile	COPD	Male	43.6	73.5 (65.0 to 83.2)	-29.9 (-39.5 to -21.3)	0.59 (0.52 to 0.67)	37.1	69.9 (58.7 to 83.2)	-32.8 (-46.3 to -21.8)	0.53 (0.44 to 0.63)
Chile	Congestive heart failure	Total	80.9	94.3 (86.0 to 103)	-13.4 (-22.5 to -5.18)	0.86 (0.78 to 0.94)	83.4	94.3 (82.7 to 107)	-10.9 (-24.2 to 0.697)	0.88 (0.77 to 1.01)
Chile	Congestive heart failure	Female	70.1	83.8 (75.5 to 93.0)	-13.7 (-22.9 to -5.40)	0.84 (0.75 to 0.93)	72.6	83.8 (72.3 to 97.1)	-11.2 (-24.3 to 0.281)	0.87 (0.75 to 1.00)
Chile	Congestive heart failure	Male	93.2	106 (96.8 to 117)	-13.3 (-23.9 to -3.74)	0.88 (0.80 to 0.96)	95.8	106 (93.1 to 122)	-10.7 (-26.3 to 2.61)	0.90 (0.78 to 1.03)
Chile	Diabetes	Total	101	113 (108 to 117)	-11.8 (-16.1 to -7.53)	0.90 (0.86 to 0.93)	96.9	109 (103 to 115)	-11.7 (-17.6 to -5.95)	0.89 (0.85 to 0.94)

Chile	Diabetes	Female	65	71.8 (67.7 to 76.1)	-6.80 (-11.0 to -2.75)	0.91 (0.86 to 0.96)	62	67.7 (62.4 to 73.5)	-5.74 (-11.5 to -0.556)	0.92 (0.84 to 0.99)
Chile	Diabetes	Male	142	160 (154 to 166)	-18.0 (-23.8 to -12.4)	0.89 (0.86 to 0.92)	137	157 (149 to 165)	-19.5 (-27.8 to -11.8)	0.88 (0.83 to 0.92)
Chile	Hypertension	Total	14.3	16.9 (14.5 to 19.8)	-2.63 (-5.44 to -0.165)	0.84 (0.72 to 0.99)	12.6	15.3 (12.3 to 19.1)	-2.72 (-6.42 to 0.419)	0.82 (0.66 to 1.03)
Chile	Hypertension	Female	13.1	15.0 (12.9 to 17.5)	-1.90 (-4.42 to 0.231)	0.87 (0.75 to 1.02)	11.4	13.4 (10.8 to 16.7)	-2.00 (-5.26 to 0.623)	0.85 (0.68 to 1.06)
Chile	Hypertension	Male	15.7	22.1 (18.9 to 25.9)	-6.38 (-10.2 to -3.19)	0.71 (0.61 to 0.83)	13.8	22.9 (16.7 to 31.4)	-9.10 (-17.7 to -2.90)	0.60 (0.44 to 0.83)
Czechia	Asthma and COPD	Total	123	156 (141 to 172)	-33.1 (-49.7 to -18.3)	0.79 (0.71 to 0.87)	119	148 (125 to 176)	-28.7 (-55.9 to -5.59)	0.81 (0.68 to 0.96)
Czechia	Asthma and COPD	Female	101	144 (130 to 160)	-43.2 (-59.5 to -28.6)	0.70 (0.63 to 0.78)	104	147 (129 to 168)	-42.7 (-63.2 to -24.8)	0.71 (0.62 to 0.81)
Czechia	Asthma and COPD	Male	153	188 (167 to 211)	-34.7 (-58.1 to -13.6)	0.82 (0.73 to 0.92)	141	179 (137 to 232)	-38.0 (-91.6 to 3.58)	0.79 (0.61 to 1.03)
Czechia	Asthma	Total	20.2	26.3 (23.9 to 28.9)	-6.05 (-8.63 to -3.63)	0.77 (0.70 to 0.85)	19.3	25.1 (20.3 to 31.1)	-5.85 (-11.8 to -0.953)	0.77 (0.62 to 0.95)
Czechia	Asthma	Female	26	33.1 (30.8 to 35.5)	-7.05 (-9.60 to -4.74)	0.79 (0.73 to 0.85)	25	30.9 (26.4 to 36.3)	-5.95 (-11.3 to -1.32)	0.81 (0.69 to 0.95)
Czechia	Asthma	Male	13.5	18.1 (15.6 to 21.0)	-4.60 (-7.47 to -2.12)	0.75 (0.64 to 0.86)	12.6	18.1 (14.7 to 22.3)	-5.50 (-9.67 to -2.11)	0.70 (0.57 to 0.86)
Czechia	COPD	Total	103	137 (124 to 151)	-34.3 (-48.4 to -21.5)	0.75 (0.68 to 0.83)	100	137 (119 to 158)	-37.0 (-57.5 to -19.1)	0.73 (0.63 to 0.84)
Czechia	COPD	Female	75	105 (94.5 to 117)	-30.2 (-42.2 to -19.4)	0.71 (0.64 to 0.79)	79.3	105 (90.4 to 122)	-25.9 (-43.8 to -11.2)	0.75 (0.64 to 0.88)
Czechia	COPD	Male	140	173 (161 to 185)	-33.0 (-45.2 to -21.7)	0.81 (0.76 to 0.87)	128	166 (151 to 183)	-38.0 (-55.2 to -22.8)	0.77 (0.70 to 0.85)
Czechia	Congestive heart failure and hypertension	Total	437	498 (444 to 558)	-60.7 (-121 to -7.31)	0.88 (0.78 to 0.98)	416	498 (423 to 585)	-81.2 (-171 to -6.78)	0.84 (0.71 to 0.98)
Czechia	Congestive heart failure and hypertension	Female	355	409 (360 to 464)	-53.3 (-108 to -5.37)	0.87 (0.77 to 0.99)	338	409 (342 to 488)	-71.1 (-153 to -4.70)	0.83 (0.69 to 0.99)
Czechia	Congestive heart failure and hypertension	Male	541	612 (549 to 681)	-70.7 (-140 to -8.48)	0.88 (0.79 to 0.98)	517	612 (525 to 712)	-94.3 (-193 to -8.25)	0.85 (0.73 to 0.98)
Czechia	Congestive heart failure	Total	366	406 (374 to 440)	-40.2 (-75.2 to -8.44)	0.90 (0.83 to 0.98)	357	406 (361 to 455)	-48.5 (-99.0 to -4.44)	0.88 (0.78 to 0.99)
Czechia	Congestive heart failure	Female	278	309 (282 to 338)	-31.1 (-61.3 to -3.87)	0.90 (0.82 to 0.99)	273	309 (271 to 351)	-35.9 (-78.8 to 1.05)	0.88 (0.78 to 1.00)
Czechia	Congestive heart failure	Male	481	534 (495 to 576)	-53.4 (-95.8 to -14.1)	0.90 (0.83 to 0.97)	468	534 (480 to 594)	-65.9 (-127 to -12.1)	0.88 (0.79 to 0.97)
Czechia	Diabetes	Total	126	145 (134 to 158)	-18.9 (-31.1 to -7.64)	0.87 (0.80 to 0.94)	100	138 (123 to 155)	-37.6 (-54.7 to -22.2)	0.73 (0.65 to 0.82)
Czechia	Diabetes	Female	94.7	111 (102 to 121)	-16.5 (-26.5 to -7.47)	0.85 (0.78 to 0.93)	82.6	104 (92.4 to 118)	-21.8 (-35.0 to -9.92)	0.79 (0.70 to 0.89)
Czechia	Diabetes	Male	161	183 (168 to 200)	-22.3 (-38.9 to -6.98)	0.88 (0.81 to 0.96)	118	175 (155 to 198)	-57.0 (-80.4 to -36.6)	0.67 (0.59 to 0.76)
Czechia	Hypertension	Total	71.5	84.6 (73.4 to 97.4)	-13.1 (-26.0 to -2.16)	0.85 (0.73 to 0.97)	59.3	77.8 (63.7 to 95.0)	-18.5 (-35.5 to -4.11)	0.76 (0.63 to 0.94)

Czechia	Hypertension	Female	77.8	91.6 (77.6 to 108)	-13.8 (-30.2 to 0.392)	0.85 (0.72 to 1.01)	64.8	84.0 (66.4 to 106)	-19.2 (-42.0 to -1.52)	0.77 (0.61 to 0.98)
Czechia	Hypertension	Male	60.2	71.5 (63.6 to 80.5)	-11.3 (-20.2 to -3.41)	0.84 (0.75 to 0.95)	49.2	65.9 (55.8 to 77.9)	-16.7 (-29.0 to -6.56)	0.75 (0.63 to 0.88)
Germany	Asthma and COPD	Total		291 (253 to 336)			193	291 (253 to 336)	-98.1 (-142 to -59.9)	0.66 (0.58 to 0.76)
Germany	Asthma and COPD	Female		255 (211 to 307)			174	255 (211 to 307)	-80.9 (-133 to -37.9)	0.68 (0.57 to 0.82)
Germany	Asthma and COPD	Male		343 (308 to 381)			219	343 (308 to 381)	-124 (-163 to -89.0)	0.64 (0.57 to 0.71)
Germany	Asthma	Total		31.7 (27.5 to 36.6)			21.3	31.7 (25.9 to 38.8)	-10.4 (-17.8 to -4.73)	0.67 (0.55 to 0.82)
Germany	Asthma	Female		39.6 (34.3 to 45.7)			25.7	39.6 (32.3 to 48.6)	-13.9 (-23.0 to -6.49)	0.65 (0.53 to 0.80)
Germany	Asthma	Male		24.0 (21.1 to 27.3)			16.5	25.4 (21.0 to 30.8)	-8.92 (-14.3 to -4.41)	0.65 (0.54 to 0.79)
Germany	COPD	Total		263 (229 to 302)			172	263 (229 to 302)	-90.9 (-131 to -57.3)	0.65 (0.57 to 0.75)
Germany	COPD	Female		219 (182 to 265)			148	219 (182 to 265)	-71.1 (-116 to -33.8)	0.68 (0.56 to 0.81)
Germany	COPD	Male		322 (289 to 358)			203	322 (289 to 358)	-119 (-153 to -86.8)	0.63 (0.57 to 0.70)
Germany	Congestive heart failure and hypertension	Total		698 (683 to 713)			566	698 (683 to 713)	-132 (-147 to -117)	0.81 (0.79 to 0.83)
Germany	Congestive heart failure and hypertension	Female		699 (680 to 719)			556	699 (680 to 719)	-144 (-163 to -125)	0.79 (0.77 to 0.82)
Germany	Congestive heart failure and hypertension	Male		689 (675 to 703)			572	689 (675 to 703)	-116 (-130 to -102)	0.83 (0.81 to 0.85)
Germany	Congestive heart failure	Total		424 (398 to 451)			363	424 (388 to 463)	-61.0 (-100 to -25.8)	0.86 (0.78 to 0.93)
Germany	Congestive heart failure	Female		367 (344 to 390)			313	367 (335 to 401)	-53.5 (-87.8 to -22.6)	0.85 (0.78 to 0.93)
Germany	Congestive heart failure	Male		497 (467 to 529)			426	497 (455 to 543)	-70.7 (-118 to -28.9)	0.86 (0.78 to 0.94)
Germany	Diabetes	Total		215 (201 to 229)			172	215 (195 to 236)	-42.8 (-64.3 to -23.2)	0.80 (0.73 to 0.88)
Germany	Diabetes	Female		149 (146 to 153)			121	142 (137 to 147)	-21.1 (-26.2 to -16.2)	0.85 (0.82 to 0.88)
Germany	Diabetes	Male		282 (271 to 293)			232	282 (266 to 298)	-49.7 (-65.7 to -34.6)	0.82 (0.78 to 0.87)
Germany	Hypertension	Total		269 (247 to 294)			203	269 (238 to 305)	-66.2 (-102 to -35.0)	0.75 (0.67 to 0.85)
Germany	Hypertension	Female		347 (310 to 387)			243	347 (310 to 387)	-104 (-143 to -68.7)	0.70 (0.63 to 0.78)
Germany	Hypertension	Male		193 (175 to 212)			146	193 (168 to 221)	-46.4 (-74.4 to -21.5)	0.76 (0.66 to 0.87)
Denmark	Asthma	Total	56.5	65.8 (58.9 to 73.6)	-9.31 (-17.3 to -2.19)	0.86 (0.77 to 0.96)	51	76.7 (68.5 to 85.7)	-25.7 (-34.8 to -17.5)	0.67 (0.59 to 0.74)

Denmark	Asthma	Female	77.6	90.8 (82.4 to 100)	-13.2 (-22.4 to - 4.70)	0.85 (0.78 to 0.94)	70.3	104 (93.9 to 114)	-33.2 (-44.0 to - 23.8)	0.68 (0.62 to 0.75)
Denmark	Asthma	Male	34.3	45.9 (37.4 to 56.3)	-11.6 (-22.2 to - 3.03)	0.75 (0.61 to 0.92)	30.5	45.9 (34.4 to 61.3)	-15.4 (-31.1 to - 4.02)	0.66 (0.49 to 0.88)
Denmark	COPD	Total	233	302 (284 to 322)	-69.9 (-89.4 to - 51.1)	0.77 (0.72 to 0.82)	226	302 (277 to 331)	-76.3 (-104 to - 50.5)	0.75 (0.68 to 0.82)
Denmark	COPD	Female	236	314 (294 to 336)	-78.0 (-99.3 to - 58.5)	0.75 (0.70 to 0.80)	234	314 (286 to 345)	-80.3 (-111 to - 52.4)	0.74 (0.68 to 0.82)
Denmark	COPD	Male	231	291 (272 to 311)	-60.0 (-80.4 to - 41.3)	0.79 (0.74 to 0.85)	220	291 (265 to 320)	-70.8 (-99.7 to - 44.3)	0.76 (0.69 to 0.83)
Denmark	Congestive heart failure	Total	160	178 (163 to 194)	-17.8 (-34.1 to - 2.90)	0.90 (0.82 to 0.98)	159	178 (157 to 201)	-18.9 (-42.3 to 2.37)	0.89 (0.79 to 1.02)
Denmark	Congestive heart failure	Female	106	116 (107 to 126)	-9.97 (-20.0 to - 0.885)	0.91 (0.84 to 0.99)	106	116 (107 to 126)	-10.4 (-20.5 to - 1.11)	0.91 (0.84 to 0.99)
Denmark	Congestive heart failure	Male	233	255 (234 to 277)	-21.6 (-44.3 to - 0.472)	0.92 (0.84 to 1.00)	231	255 (226 to 287)	-23.6 (-55.9 to 5.61)	0.91 (0.81 to 1.02)
Denmark	Diabetes	Total	111	118 (113 to 123)	-7.14 (-12.1 to - 2.38)	0.94 (0.90 to 0.98)	102	113 (106 to 119)	-10.4 (-17.2 to - 4.04)	0.91 (0.86 to 0.96)
Denmark	Diabetes	Female	74.8	84.8 (80.3 to 89.7)	-10.0 (-14.7 to - 5.52)	0.88 (0.84 to 0.93)	71.7	80.6 (74.5 to 87.2)	-8.92 (-15.5 to - 2.89)	0.89 (0.82 to 0.96)
Denmark	Diabetes	Male	152	157 (149 to 164)	-4.29 (-12.0 to 2.95)	0.97 (0.93 to 1.02)	138	151 (141 to 161)	-12.7 (-23.2 to - 2.46)	0.92 (0.86 to 0.98)
Denmark	Hypertension	Total	46.4	50.6 (45.7 to 56.1)	-4.23 (-9.73 to 0.677)	0.92 (0.83 to 1.01)	41.2	47.7 (41.2 to 55.1)	-6.45 (-13.8 to - 0.065)	0.86 (0.75 to 1.00)
Denmark	Hypertension	Female	50.2	54.2 (46.6 to 62.9)	-3.97 (-12.6 to 3.73)	0.93 (0.80 to 1.08)	44.7	50.9 (41.2 to 62.8)	-6.16 (-18.3 to 3.61)	0.88 (0.71 to 1.09)
Denmark	Hypertension	Male	39.6	44.2 (39.2 to 49.8)	-4.57 (-10.2 to 0.334)	0.90 (0.80 to 1.01)	35.1	41.7 (35.2 to 49.4)	-6.59 (-14.3 to - 0.046)	0.84 (0.71 to 1.00)
Spain	Asthma and COPD	Total	149	226 (198 to 257)	-76.9 (-108 to - 48.8)	0.66 (0.58 to 0.75)	132	226 (188 to 271)	-93.5 (-139 to - 55.9)	0.59 (0.49 to 0.70)
Spain	Asthma and COPD	Female	93.6	141 (129 to 155)	-47.5 (-61.1 to - 35.3)	0.66 (0.61 to 0.73)	87.7	141 (129 to 155)	-53.4 (-67.3 to - 41.4)	0.62 (0.57 to 0.68)
Spain	Asthma and COPD	Male	230	346 (303 to 396)	-116 (-166 to - 72.7)	0.66 (0.58 to 0.76)	199	346 (287 to 418)	-148 (-221 to - 89.0)	0.57 (0.47 to 0.69)
Spain	Asthma	Total	23.8	34.8 (27.9 to 43.5)	-11.0 (-19.7 to - 3.97)	0.68 (0.55 to 0.86)	22.4	34.8 (25.4 to 47.7)	-12.4 (-25.2 to - 3.05)	0.64 (0.47 to 0.88)
Spain	Asthma	Female	31.6	49.3 (40.4 to 60.2)	-17.7 (-28.5 to - 8.77)	0.64 (0.53 to 0.78)	29.7	49.3 (37.2 to 65.4)	-19.6 (-36.1 to - 7.63)	0.60 (0.45 to 0.80)
Spain	Asthma	Male	13.7	19.6 (14.8 to 25.8)	-5.86 (-12.1 to - 1.09)	0.70 (0.53 to 0.93)	13.1	19.6 (14.8 to 25.8)	-6.46 (-12.7 to - 1.72)	0.67 (0.51 to 0.88)
Spain	COPD	Total	125	191 (169 to 215)	-65.9 (-89.4 to - 44.5)	0.65 (0.58 to 0.74)	110	191 (161 to 226)	-81.1 (-116 to - 51.5)	0.57 (0.49 to 0.68)
Spain	COPD	Female	62	96.1 (85.7 to 108)	-34.1 (-45.7 to - 23.5)	0.65 (0.58 to 0.73)	58	96.1 (81.7 to 113)	-38.1 (-54.9 to - 23.6)	0.60 (0.51 to 0.71)
Spain	COPD	Male	216	330 (290 to 375)	-113 (-159 to - 73.3)	0.66 (0.58 to 0.75)	186	330 (275 to 395)	-144 (-208 to - 88.7)	0.56 (0.47 to 0.68)
Spain	Congestive heart failure and hypertension	Total	146	175 (136 to 225)	-28.8 (-78.7 to 10.2)	0.84 (0.65 to 1.07)	172	175 (123 to 249)	-3.30 (-75.3 to 49.6)	0.98 (0.70 to 1.41)
Spain	Congestive heart failure and hypertension	Female	124	148 (114 to 191)	-24.0 (-64.9 to 9.21)	0.84 (0.66 to 1.08)	148	148 (103 to 212)	0.700 (-63.3 to 46.0)	1.00 (0.70 to 1.45)

Spain	Congestive heart failure and hypertension	Male	175	210 (164 to 269)	-35.0 (-94.8 to 11.1)	0.83 (0.65 to 1.07)	200	210 (148 to 298)	-9.60 (-97.4 to 53.5)	0.95 (0.67 to 1.36)
Spain	Congestive heart failure	Total	141	169 (130 to 220)	-27.7 (-78.2 to 11.3)	0.84 (0.64 to 1.09)	167	169 (117 to 245)	-2.40 (-78.8 to 49.6)	0.99 (0.68 to 1.42)
Spain	Congestive heart failure	Female	120	143 (109 to 186)	-23.1 (-66.9 to 11.3)	0.84 (0.64 to 1.10)	144	143 (97.7 to 208)	1.30 (-65.4 to 46.0)	1.01 (0.69 to 1.47)
Spain	Congestive heart failure	Male	169	202 (156 to 262)	-33.6 (-93.7 to 12.7)	0.83 (0.64 to 1.08)	194	202 (141 to 291)	-8.10 (-96.5 to 54.6)	0.96 (0.67 to 1.39)
Spain	Diabetes	Total	52	53.2 (48.4 to 58.5)	-1.22 (-6.50 to 3.53)	0.98 (0.89 to 1.07)	56.8	54.1 (43.8 to 66.9)	2.65 (-10.4 to 12.9)	1.05 (0.85 to 1.30)
Spain	Diabetes	Female	32.8	36.3 (33.4 to 39.4)	-3.51 (-6.60 to -0.665)	0.90 (0.83 to 0.98)	36.2	36.8 (30.6 to 44.2)	-0.621 (-7.99 to 5.49)	0.98 (0.82 to 1.18)
Spain	Diabetes	Male	74.2	72.4 (65.1 to 80.6)	1.78 (-6.30 to 9.08)	1.02 (0.92 to 1.14)	81.4	73.8 (58.0 to 93.8)	7.63 (-12.6 to 23.5)	1.10 (0.87 to 1.41)
Spain	Hypertension	Total	4.8	6.00 (4.14 to 8.71)	-1.20 (-3.90 to 0.657)	0.80 (0.55 to 1.16)	5	6.00 (3.54 to 10.2)	-1.00 (-5.20 to 1.46)	0.83 (0.49 to 1.41)
Spain	Hypertension	Female	3.9	4.80 (3.07 to 7.51)	-0.900 (-3.62 to 0.837)	0.81 (0.52 to 1.27)	4.2	4.80 (2.55 to 9.03)	-0.600 (-4.86 to 1.64)	0.88 (0.46 to 1.64)
Spain	Hypertension	Male	6	6.77 (5.21 to 8.81)	-0.774 (-2.86 to 0.767)	0.89 (0.68 to 1.15)	5.9	6.20 (4.28 to 8.99)	-0.301 (-3.08 to 1.61)	0.95 (0.66 to 1.38)
Estonia	Asthma and COPD	Total	80.7	117 (99.1 to 137)	-35.9 (-56.0 to -18.6)	0.69 (0.59 to 0.81)	65.6	117 (92.7 to 147)	-51.0 (-81.2 to -27.1)	0.56 (0.45 to 0.71)
Estonia	Asthma and COPD	Female	53.2	80.1 (68.4 to 93.9)	-26.9 (-40.3 to -15.3)	0.66 (0.57 to 0.78)	44.7	80.1 (64.0 to 100)	-35.4 (-55.2 to -19.2)	0.56 (0.45 to 0.70)
Estonia	Asthma and COPD	Male	119	166 (138 to 200)	-46.9 (-80.7 to -19.3)	0.72 (0.60 to 0.86)	93.8	166 (128 to 216)	-72.0 (-122 to -33.4)	0.57 (0.44 to 0.74)
Estonia	Asthma	Total	16.2	25.5 (18.9 to 34.4)	-9.30 (-18.2 to -2.83)	0.64 (0.47 to 0.85)	16.2	25.5 (16.7 to 38.9)	-9.30 (-22.7 to -0.622)	0.64 (0.42 to 0.96)
Estonia	Asthma	Female	19.8	32.8 (25.2 to 42.7)	-13.0 (-22.9 to -5.53)	0.60 (0.46 to 0.78)	19.9	32.8 (22.6 to 47.6)	-12.9 (-28.4 to -2.76)	0.61 (0.41 to 0.88)
Estonia	Asthma	Male	11	15.9 (10.1 to 25.0)	-4.90 (-13.8 to 0.944)	0.69 (0.44 to 1.09)	11	15.9 (8.41 to 30.1)	-4.90 (-18.9 to 2.56)	0.69 (0.37 to 1.30)
Estonia	COPD	Total	64.5	91.1 (78.1 to 106)	-26.6 (-41.7 to -13.7)	0.71 (0.61 to 0.82)	49.4	91.1 (73.2 to 113)	-41.7 (-63.9 to -23.5)	0.54 (0.44 to 0.68)
Estonia	COPD	Female	33.5	47.3 (39.4 to 56.8)	-13.8 (-23.2 to -5.71)	0.71 (0.59 to 0.85)	24.8	47.3 (36.5 to 61.2)	-22.5 (-36.0 to -11.6)	0.52 (0.41 to 0.68)
Estonia	COPD	Male	108	150 (126 to 179)	-42.1 (-70.8 to -17.4)	0.72 (0.60 to 0.86)	82.9	150 (117 to 193)	-67.1 (-110 to -33.8)	0.55 (0.43 to 0.71)
Estonia	Congestive heart failure and hypertension	Total	328	339 (315 to 366)	-10.8 (-36.9 to 13.9)	0.97 (0.90 to 1.04)	265	319 (287 to 355)	-54.2 (-89.7 to -22.3)	0.83 (0.75 to 0.92)
Estonia	Congestive heart failure and hypertension	Female	283	303 (283 to 325)	-20.2 (-41.7 to -0.206)	0.93 (0.87 to 1.00)	222	283 (257 to 312)	-61.8 (-90.8 to -35.3)	0.78 (0.71 to 0.86)
Estonia	Congestive heart failure and hypertension	Male	385	401 (342 to 469)	-15.5 (-82.7 to 43.3)	0.96 (0.82 to 1.13)	316	401 (320 to 501)	-84.8 (-187 to -6.36)	0.79 (0.63 to 0.98)
Estonia	Congestive heart failure	Total	243	269 (242 to 299)	-25.6 (-55.7 to 1.71)	0.90 (0.81 to 1.01)	208	269 (231 to 312)	-60.7 (-105 to -22.8)	0.77 (0.66 to 0.90)
Estonia	Congestive heart failure	Female	198	230 (204 to 261)	-32.0 (-62.3 to -5.28)	0.86 (0.76 to 0.97)	170	230 (194 to 274)	-60.1 (-104 to -23.5)	0.74 (0.62 to 0.88)

Estonia	Congestive heart failure	Male	300	313 (274 to 358)	-12.7 (-58.7 to 27.1)	0.96 (0.84 to 1.10)	254	313 (259 to 379)	-59.1 (-126 to -5.40)	0.81 (0.67 to 0.98)
Estonia	Diabetes	Total	98.1	107 (92.2 to 124)	-9.00 (-26.4 to 5.65)	0.92 (0.79 to 1.06)	79.9	107 (86.6 to 132)	-27.2 (-51.6 to -6.98)	0.75 (0.61 to 0.92)
Estonia	Diabetes	Female	82.7	86.8 (72.2 to 104)	-4.10 (-21.4 to 10.5)	0.95 (0.79 to 1.15)	67.3	86.8 (66.9 to 113)	-19.5 (-45.4 to 0.635)	0.78 (0.60 to 1.01)
Estonia	Diabetes	Male	114	128 (113 to 146)	-13.8 (-31.3 to 1.36)	0.89 (0.79 to 1.01)	94.6	128 (107 to 153)	-33.4 (-59.0 to -12.1)	0.74 (0.62 to 0.89)
Estonia	Hypertension	Total	85.3	81.9 (76.8 to 87.3)	3.43 (-1.97 to 8.40)	1.04 (0.98 to 1.11)	56.8	72.8 (66.5 to 79.8)	-16.0 (-23.0 to -9.78)	0.78 (0.71 to 0.85)
Estonia	Hypertension	Female	84.6	83.6 (79.6 to 87.8)	1.03 (-3.16 to 4.93)	1.01 (0.96 to 1.06)	51.4	74.5 (69.5 to 79.8)	-23.1 (-28.5 to -18.0)	0.69 (0.64 to 0.74)
Estonia	Hypertension	Male	84.6	77.6 (68.1 to 88.4)	7.01 (-3.69 to 16.5)	1.09 (0.96 to 1.24)	61.7	68.9 (57.3 to 82.8)	-7.18 (-21.3 to 4.40)	0.90 (0.74 to 1.08)
United Kingdom	Asthma and COPD	Total	158	314 (288 to 342)	-156 (-185 to -130)	0.50 (0.46 to 0.55)	210	314 (278 to 355)	-104 (-144 to -66.8)	0.67 (0.59 to 0.76)
United Kingdom	Asthma and COPD	Female	169	342 (313 to 372)	-173 (-204 to -145)	0.49 (0.45 to 0.54)	235	342 (313 to 372)	-106 (-137 to -78.3)	0.69 (0.63 to 0.75)
United Kingdom	Asthma and COPD	Male	149	269 (265 to 274)	-120 (-124 to -116)	0.55 (0.55 to 0.56)	185	266 (261 to 270)	-80.2 (-85.1 to -75.3)	0.70 (0.69 to 0.71)
United Kingdom	Asthma	Total	34.4	75.2 (69.1 to 82.0)	-40.8 (-47.7 to -34.6)	0.46 (0.42 to 0.50)	47.2	74.9 (68.7 to 81.7)	-27.7 (-34.4 to -21.4)	0.63 (0.58 to 0.69)
United Kingdom	Asthma	Female	46.6	109 (102 to 118)	-62.7 (-71.0 to -54.9)	0.43 (0.40 to 0.46)	65.5	109 (102 to 118)	-44.0 (-52.4 to -36.3)	0.60 (0.56 to 0.64)
United Kingdom	Asthma	Male	21.7	41.8 (38.3 to 45.7)	-20.1 (-24.0 to -16.7)	0.52 (0.48 to 0.57)	27.9	41.8 (38.3 to 45.7)	-13.9 (-17.7 to -10.4)	0.67 (0.61 to 0.73)
United Kingdom	COPD	Total	124	239 (218 to 262)	-116 (-139 to -94.5)	0.52 (0.47 to 0.57)	163	239 (210 to 272)	-75.9 (-108 to -47.5)	0.68 (0.60 to 0.77)
United Kingdom	COPD	Female	122	243 (215 to 275)	-121 (-154 to -93.1)	0.50 (0.44 to 0.57)	170	243 (215 to 275)	-73.5 (-106 to -44.7)	0.70 (0.61 to 0.79)
United Kingdom	COPD	Male	127	226 (221 to 232)	-99.1 (-105 to -93.8)	0.56 (0.55 to 0.58)	158	223 (217 to 228)	-65.2 (-70.8 to -59.9)	0.71 (0.69 to 0.72)
United Kingdom	Congestive heart failure and hypertension	Total	117	126 (112 to 140)	-8.61 (-23.7 to 4.65)	0.93 (0.83 to 1.04)	134	126 (112 to 140)	8.49 (-6.35 to 21.7)	1.07 (0.95 to 1.19)
United Kingdom	Congestive heart failure and hypertension	Female	102	108 (95.9 to 121)	-5.11 (-18.5 to 6.73)	0.95 (0.85 to 1.07)	114	108 (95.9 to 121)	6.19 (-6.89 to 17.9)	1.06 (0.94 to 1.19)
United Kingdom	Congestive heart failure and hypertension	Male	136	150 (134 to 168)	-14.7 (-33.0 to 1.74)	0.90 (0.80 to 1.01)	162	150 (134 to 168)	12.1 (-5.89 to 28.4)	1.08 (0.97 to 1.21)
United Kingdom	Congestive heart failure	Total	97.9	111 (98.4 to 126)	-13.2 (-27.7 to -0.575)	0.88 (0.78 to 0.99)	113	111 (98.4 to 126)	1.46 (-13.3 to 14.1)	1.01 (0.89 to 1.14)
United Kingdom	Congestive heart failure	Female	82.2	92.3 (81.6 to 104)	-10.1 (-22.0 to 0.477)	0.89 (0.79 to 1.01)	95.5	92.3 (81.6 to 104)	3.25 (-8.92 to 14.0)	1.04 (0.91 to 1.17)
United Kingdom	Congestive heart failure	Male	119	138 (121 to 156)	-18.7 (-37.2 to -2.27)	0.86 (0.76 to 0.98)	136	138 (121 to 156)	-2.12 (-20.1 to 14.4)	0.98 (0.87 to 1.12)
United Kingdom	Diabetes	Total	73	84.3 (78.0 to 91.1)	-11.3 (-18.0 to -5.03)	0.87 (0.80 to 0.94)	79.6	86.9 (73.0 to 103)	-7.34 (-23.9 to 6.57)	0.92 (0.77 to 1.09)
United Kingdom	Diabetes	Female	58.4	68.2 (65.1 to 71.5)	-9.80 (-13.1 to -6.73)	0.86 (0.82 to 0.90)	65.2	68.2 (63.8 to 72.8)	-3.00 (-7.59 to 1.35)	0.96 (0.90 to 1.02)

United Kingdom	Diabetes	Male	89.5	101 (90.9 to 112)	-11.2 (-21.8 to -1.44)	0.89 (0.80 to 0.98)	96.1	104 (82.9 to 131)	-8.27 (-35.1 to 12.8)	0.92 (0.73 to 1.15)
United Kingdom	Hypertension	Total	19.1	19.6 (17.5 to 22.0)	-0.545 (-2.89 to 1.59)	0.97 (0.87 to 1.09)	21.4	20.6 (17.6 to 24.2)	0.763 (-2.82 to 3.83)	1.04 (0.88 to 1.22)
United Kingdom	Hypertension	Female	20.3	21.2 (18.7 to 24.0)	-0.908 (-3.74 to 1.62)	0.96 (0.84 to 1.09)	18.3	22.4 (18.7 to 26.7)	-4.08 (-8.46 to -0.545)	0.82 (0.68 to 0.97)
United Kingdom	Hypertension	Male	16.6	17.0 (15.2 to 19.0)	-0.394 (-2.38 to 1.40)	0.98 (0.87 to 1.09)	26.7	17.7 (15.1 to 20.7)	8.98 (6.08 to 11.6)	1.51 (1.29 to 1.77)
Ireland	Asthma and COPD	Total	290	429 (398 to 462)	-139 (-172 to -109)	0.68 (0.63 to 0.73)	248	429 (398 to 462)	-181 (-215 to -151)	0.58 (0.54 to 0.62)
Ireland	Asthma and COPD	Female	291	419 (386 to 455)	-128 (-162 to -95.4)	0.69 (0.64 to 0.75)	253	419 (386 to 455)	-165 (-201 to -133)	0.60 (0.56 to 0.66)
Ireland	Asthma and COPD	Male	299	404 (375 to 437)	-106 (-138 to -75.2)	0.74 (0.68 to 0.80)	248	404 (363 to 451)	-156 (-202 to -115)	0.61 (0.55 to 0.68)
Ireland	Asthma	Total	33.5	41.7 (38.1 to 45.6)	-8.19 (-12.2 to -4.55)	0.80 (0.73 to 0.88)	28.5	41.7 (38.1 to 45.6)	-13.2 (-17.2 to -9.60)	0.68 (0.62 to 0.75)
Ireland	Asthma	Female	43.1	55.0 (49.5 to 61.1)	-11.9 (-18.0 to -6.48)	0.78 (0.71 to 0.87)	36	55.0 (49.5 to 61.1)	-19.0 (-25.1 to -13.6)	0.65 (0.59 to 0.73)
Ireland	Asthma	Male	23.1	28.3 (24.8 to 32.3)	-5.20 (-9.07 to -1.71)	0.82 (0.72 to 0.93)	20.6	28.3 (23.5 to 34.1)	-7.70 (-13.5 to -2.93)	0.73 (0.60 to 0.88)
Ireland	COPD	Total	256	366 (344 to 391)	-110 (-134 to -87.3)	0.70 (0.66 to 0.75)	219	385 (354 to 418)	-165 (-198 to -134)	0.57 (0.53 to 0.62)
Ireland	COPD	Female	248	364 (334 to 396)	-116 (-149 to -86.0)	0.68 (0.62 to 0.74)	217	364 (334 to 396)	-146 (-179 to -117)	0.60 (0.55 to 0.65)
Ireland	COPD	Male	276	376 (346 to 409)	-100 (-133 to -70.2)	0.73 (0.67 to 0.80)	228	376 (334 to 424)	-149 (-196 to -106)	0.60 (0.54 to 0.68)
Ireland	Congestive heart failure and hypertension	Total	186	191 (173 to 211)	-5.20 (-25.4 to 12.9)	0.97 (0.88 to 1.07)	190	191 (166 to 220)	-0.700 (-29.5 to 24.6)	1.00 (0.87 to 1.15)
Ireland	Congestive heart failure and hypertension	Female	156	163 (149 to 178)	-7.48 (-22.7 to 6.55)	0.95 (0.87 to 1.04)	166	163 (149 to 178)	3.22 (-11.8 to 16.8)	1.02 (0.93 to 1.11)
Ireland	Congestive heart failure and hypertension	Male	228	232 (207 to 261)	-4.10 (-33.2 to 22.0)	0.98 (0.87 to 1.11)	223	232 (197 to 274)	-9.00 (-51.0 to 27.0)	0.96 (0.81 to 1.14)
Ireland	Congestive heart failure	Total	152	158 (144 to 173)	-6.20 (-22.1 to 8.12)	0.96 (0.87 to 1.06)	156	158 (138 to 180)	-2.30 (-24.4 to 17.2)	0.99 (0.86 to 1.12)
Ireland	Congestive heart failure	Female	119	126 (115 to 138)	-6.90 (-18.7 to 3.71)	0.95 (0.86 to 1.03)	128	126 (111 to 143)	1.80 (-15.6 to 17.0)	1.01 (0.89 to 1.15)
Ireland	Congestive heart failure	Male	199	204 (182 to 229)	-5.20 (-30.0 to 17.0)	0.97 (0.87 to 1.09)	195	204 (173 to 240)	-9.20 (-45.2 to 21.1)	0.95 (0.81 to 1.12)
Ireland	Diabetes	Total	88.5	97.4 (76.7 to 124)	-8.90 (-35.0 to 11.9)	0.91 (0.72 to 1.16)	94.4	97.4 (69.5 to 137)	-3.00 (-41.8 to 25.3)	0.97 (0.69 to 1.37)
Ireland	Diabetes	Female	60.4	71.7 (56.5 to 90.9)	-11.3 (-30.4 to 4.01)	0.84 (0.67 to 1.07)	69.2	71.7 (51.2 to 100)	-2.50 (-31.7 to 17.8)	0.97 (0.69 to 1.35)
Ireland	Diabetes	Male	123	130 (101 to 167)	-6.70 (-44.0 to 22.1)	0.95 (0.74 to 1.22)	125	130 (90.7 to 186)	-4.80 (-61.3 to 33.7)	0.96 (0.67 to 1.37)
Ireland	Hypertension	Total	34.1	31.1 (26.5 to 36.4)	3.03 (-2.34 to 7.62)	1.10 (0.94 to 1.29)	34.7	31.1 (26.5 to 36.4)	3.63 (-1.68 to 8.14)	1.12 (0.95 to 1.31)
Ireland	Hypertension	Female	36.5	33.2 (28.7 to 38.3)	3.34 (-1.72 to 7.76)	1.10 (0.96 to 1.27)	38.5	33.2 (28.7 to 38.3)	5.34 (0.223 to 9.77)	1.16 (1.01 to 1.34)

Ireland	Hypertension	Male	29.6	27.5 (23.1 to 32.8)	2.06 (-3.21 to 6.38)	1.07 (0.90 to 1.27)	28.8	27.5 (23.1 to 32.8)	1.26 (-4.15 to 5.69)	1.05 (0.87 to 1.25)
Iceland	Asthma and COPD	Total	115	143 (109 to 186)	-27.3 (-70.6 to 5.86)	0.81 (0.62 to 1.05)	119	143 (97.7 to 208)	-23.8 (-89.6 to 20.5)	0.83 (0.57 to 1.21)
Iceland	Asthma and COPD	Female	133	246 (189 to 321)	-114 (-188 to -57.6)	0.54 (0.41 to 0.70)	144	246 (189 to 321)	-102 (-178 to -45.7)	0.58 (0.45 to 0.76)
Iceland	Asthma and COPD	Male	93.4	87.7 (61.3 to 126)	5.70 (-32.6 to 32.0)	1.06 (0.74 to 1.52)	82	87.7 (52.8 to 146)	-5.70 (-62.7 to 28.5)	0.94 (0.57 to 1.53)
Iceland	Asthma	Total	5.1	5.90 (3.30 to 10.6)	-0.800 (-5.30 to 1.82)	0.86 (0.49 to 1.55)	6.3	5.90 (2.59 to 13.4)	0.400 (-7.24 to 3.72)	1.07 (0.47 to 2.44)
Iceland	Asthma	Female	8.9	8.70 (3.86 to 19.6)	0.200 (-10.7 to 5.11)	1.02 (0.45 to 2.35)	7.8	8.70 (2.75 to 27.5)	-0.900 (-19.5 to 5.09)	0.90 (0.29 to 2.88)
Iceland	Asthma	Male	0.8	6.98 (2.54 to 19.2)	-6.18 (-18.7 to -1.79)	0.11 (0.04 to 0.31)	4.2	6.98 (2.54 to 19.2)	-2.78 (-14.6 to 1.65)	0.60 (0.22 to 1.65)
Iceland	COPD	Total	110	188 (142 to 251)	-78.1 (-140 to -30.8)	0.59 (0.44 to 0.78)	112	188 (142 to 251)	-75.8 (-138 to -28.9)	0.60 (0.45 to 0.80)
Iceland	COPD	Female	124	228 (178 to 291)	-104 (-168 to -52.9)	0.54 (0.42 to 0.70)	136	228 (178 to 291)	-91.5 (-156 to -42.5)	0.60 (0.47 to 0.76)
Iceland	COPD	Male	92.6	85.0 (59.6 to 121)	7.60 (-28.1 to 33.1)	1.09 (0.77 to 1.56)	77.8	85.0 (51.4 to 141)	-7.20 (-63.2 to 25.9)	0.92 (0.55 to 1.50)
Iceland	Congestive heart failure and hypertension	Total	204	204 (188 to 221)	-0.444 (-17.2 to 15.2)	1.00 (0.92 to 1.08)	176	204 (188 to 221)	-27.9 (-45.2 to -12.2)	0.86 (0.80 to 0.94)
Iceland	Congestive heart failure and hypertension	Female	186	145 (138 to 151)	41.5 (34.9 to 47.9)	1.29 (1.23 to 1.35)	139	135 (126 to 143)	4.29 (-4.29 to 12.4)	1.03 (0.97 to 1.10)
Iceland	Congestive heart failure and hypertension	Male	228	256 (214 to 305)	-27.1 (-75.7 to 13.6)	0.89 (0.75 to 1.06)	230	256 (214 to 305)	-25.6 (-74.4 to 15.7)	0.90 (0.76 to 1.07)
Iceland	Congestive heart failure	Total	174	178 (146 to 218)	-4.60 (-45.3 to 28.3)	0.97 (0.79 to 1.19)	159	178 (134 to 237)	-19.5 (-78.2 to 23.7)	0.89 (0.67 to 1.17)
Iceland	Congestive heart failure	Female	150	126 (106 to 151)	23.5 (-1.15 to 44.1)	1.19 (0.99 to 1.42)	120	126 (106 to 151)	-6.38 (-31.2 to 14.4)	0.95 (0.79 to 1.14)
Iceland	Congestive heart failure	Male	208	272 (192 to 387)	-64.2 (-178 to 15.5)	0.76 (0.54 to 1.08)	215	272 (166 to 447)	-57.3 (-229 to 47.5)	0.79 (0.48 to 1.28)
Iceland	Diabetes	Total	37.4	36.0 (29.3 to 44.2)	1.40 (-6.77 to 7.90)	1.04 (0.85 to 1.27)	29.8	36.0 (26.9 to 48.2)	-6.20 (-18.4 to 2.83)	0.83 (0.62 to 1.10)
Iceland	Diabetes	Female	28.1	28.2 (21.2 to 37.5)	-0.071 (-9.48 to 6.85)	1.00 (0.75 to 1.32)	27.6	28.2 (21.2 to 37.5)	-0.571 (-9.86 to 6.47)	0.98 (0.74 to 1.31)
Iceland	Diabetes	Male	49.1	44.7 (33.2 to 60.2)	4.39 (-11.1 to 15.7)	1.10 (0.82 to 1.47)	33	45.5 (32.0 to 64.7)	-12.5 (-32.0 to 1.15)	0.73 (0.51 to 1.04)
Iceland	Hypertension	Total	29.9	18.4 (12.6 to 26.8)	11.5 (3.02 to 17.2)	1.63 (1.11 to 2.36)	17.3	15.9 (9.33 to 27.1)	1.39 (-9.83 to 7.90)	1.09 (0.64 to 1.84)
Iceland	Hypertension	Female	36.3	26.6 (17.9 to 39.5)	9.70 (-3.04 to 18.3)	1.36 (0.92 to 2.02)	18.8	26.6 (15.2 to 46.5)	-7.80 (-27.2 to 3.54)	0.71 (0.41 to 1.23)
Iceland	Hypertension	Male	20.4	11.4 (6.49 to 19.9)	9.04 (0.548 to 13.9)	1.80 (1.03 to 3.13)	14.9	9.35 (4.24 to 20.6)	5.55 (-5.72 to 10.7)	1.59 (0.72 to 3.51)
Israel	Asthma and COPD	Total	127	209 (192 to 228)	-82.1 (-101 to -64.3)	0.61 (0.56 to 0.66)	148	203 (179 to 230)	-55.6 (-82.4 to -31.5)	0.73 (0.64 to 0.82)
Israel	Asthma and COPD	Female	97.7	168 (156 to 181)	-70.6 (-83.2 to -58.6)	0.58 (0.54 to 0.63)	119	163 (147 to 181)	-44.0 (-62.0 to -28.1)	0.73 (0.66 to 0.81)

Israel	Asthma and COPD	Male	165	268 (237 to 304)	-104 (-139 to -72.4)	0.61 (0.54 to 0.69)	183	268 (225 to 320)	-85.8 (-138 to -43.0)	0.68 (0.57 to 0.81)
Israel	Asthma	Total	22.8	39.5 (36.5 to 42.7)	-16.7 (-20.0 to -13.7)	0.58 (0.53 to 0.62)	30.3	38.0 (34.0 to 42.5)	-7.73 (-12.2 to -3.72)	0.80 (0.71 to 0.89)
Israel	Asthma	Female	28.6	50.1 (46.4 to 54.2)	-21.5 (-25.5 to -17.8)	0.57 (0.53 to 0.62)	36.9	48.2 (43.1 to 53.8)	-11.3 (-16.9 to -6.33)	0.77 (0.69 to 0.85)
Israel	Asthma	Male	15.8	27.4 (24.0 to 31.3)	-11.6 (-15.5 to -8.27)	0.58 (0.50 to 0.66)	22.4	27.4 (22.7 to 33.1)	-5.00 (-10.8 to -0.282)	0.82 (0.67 to 0.99)
Israel	COPD	Total	104	174 (157 to 194)	-70.1 (-89.5 to -53.0)	0.60 (0.54 to 0.66)	117	174 (150 to 202)	-57.2 (-85.0 to -33.3)	0.67 (0.58 to 0.78)
Israel	COPD	Female	69.1	118 (110 to 128)	-49.3 (-58.5 to -40.3)	0.58 (0.54 to 0.63)	82.3	115 (103 to 129)	-33.0 (-46.4 to -20.6)	0.71 (0.64 to 0.80)
Israel	COPD	Male	149	241 (211 to 275)	-92.3 (-126 to -62.3)	0.62 (0.54 to 0.70)	160	241 (200 to 290)	-80.8 (-130 to -40.1)	0.66 (0.55 to 0.80)
Israel	Congestive heart failure and hypertension	Total	300	340 (306 to 378)	-40.4 (-77.3 to -6.70)	0.88 (0.80 to 0.98)	349	340 (293 to 395)	8.60 (-44.2 to 56.5)	1.03 (0.89 to 1.19)
Israel	Congestive heart failure and hypertension	Female	267	301 (278 to 326)	-33.4 (-58.5 to -10.2)	0.89 (0.82 to 0.96)	307	301 (268 to 337)	6.50 (-30.6 to 38.8)	1.02 (0.91 to 1.14)
Israel	Congestive heart failure and hypertension	Male	336	363 (320 to 411)	-26.6 (-75.3 to 15.5)	0.93 (0.82 to 1.05)	395	363 (320 to 411)	32.3 (-17.0 to 74.4)	1.09 (0.96 to 1.23)
Israel	Congestive heart failure	Total	200	251 (226 to 279)	-50.8 (-79.7 to -25.2)	0.80 (0.72 to 0.89)	202	251 (216 to 292)	-49.4 (-89.9 to -14.6)	0.80 (0.69 to 0.93)
Israel	Congestive heart failure	Female	177	214 (194 to 236)	-37.0 (-59.6 to -16.7)	0.83 (0.75 to 0.91)	174	214 (186 to 246)	-40.1 (-72.1 to -11.9)	0.81 (0.71 to 0.94)
Israel	Congestive heart failure	Male	228	293 (267 to 322)	-65.8 (-95.1 to -39.6)	0.78 (0.71 to 0.85)	236	293 (267 to 322)	-57.8 (-86.3 to -31.2)	0.80 (0.73 to 0.88)
Israel	Diabetes	Total	68.7	77.1 (66.5 to 89.2)	-8.36 (-20.3 to 2.54)	0.89 (0.77 to 1.04)	90.4	80.1 (62.9 to 102)	10.3 (-12.0 to 28.0)	1.13 (0.88 to 1.45)
Israel	Diabetes	Female	47.5	53.0 (42.7 to 65.8)	-5.50 (-18.4 to 4.82)	0.90 (0.72 to 1.11)	60	53.0 (39.1 to 71.9)	7.00 (-11.9 to 20.8)	1.13 (0.83 to 1.53)
Israel	Diabetes	Male	93.2	105 (89.5 to 122)	-11.4 (-28.5 to 3.77)	0.89 (0.77 to 1.04)	126	105 (85.8 to 128)	21.2 (-1.86 to 39.5)	1.20 (0.99 to 1.46)
Israel	Hypertension	Total	99.4	77.8 (59.3 to 102)	21.6 (-2.43 to 39.8)	1.28 (0.98 to 1.67)	147	77.8 (59.3 to 102)	69.2 (44.2 to 87.3)	1.89 (1.43 to 2.46)
Israel	Hypertension	Female	90.3	82.6 (68.1 to 100)	7.73 (-9.49 to 22.3)	1.09 (0.90 to 1.33)	133	82.6 (68.1 to 100)	50.8 (33.4 to 65.2)	1.62 (1.33 to 1.96)
Israel	Hypertension	Male	109	88.4 (63.5 to 123)	20.2 (-15.1 to 45.1)	1.23 (0.88 to 1.71)	160	88.4 (55.3 to 141)	71.2 (17.3 to 104)	1.81 (1.12 to 2.89)
Italy	Asthma and COPD	Total	24.2	42.0 (35.4 to 49.7)	-17.8 (-25.2 to -11.2)	0.58 (0.49 to 0.68)	23	40.1 (33.6 to 47.9)	-17.1 (-25.1 to -10.8)	0.57 (0.48 to 0.68)
Italy	Asthma and COPD	Female	20	36.5 (31.6 to 42.2)	-16.5 (-22.2 to -11.7)	0.55 (0.47 to 0.63)	19.8	35.4 (30.5 to 41.1)	-15.6 (-21.4 to -10.8)	0.56 (0.48 to 0.65)
Italy	Asthma and COPD	Male	30.2	50.5 (41.6 to 61.4)	-20.3 (-31.0 to -11.4)	0.60 (0.49 to 0.73)	27.6	47.8 (39.0 to 58.7)	-20.2 (-31.0 to -11.3)	0.58 (0.47 to 0.71)
Italy	Asthma	Total	4.5	9.00 (7.53 to 10.8)	-4.50 (-6.28 to -3.03)	0.50 (0.42 to 0.60)	4.8	9.00 (6.99 to 11.6)	-4.20 (-6.78 to -2.22)	0.53 (0.41 to 0.68)
Italy	Asthma	Female	4.9	10.3 (8.97 to 11.8)	-5.40 (-6.93 to -4.08)	0.48 (0.41 to 0.55)	5.5	9.82 (8.08 to 11.9)	-4.32 (-6.42 to -2.59)	0.56 (0.46 to 0.68)

Italy	Asthma	Male	3.9	6.90 (5.56 to 8.56)	-3.00 (-4.66 to -1.68)	0.57 (0.46 to 0.70)	4.1	6.90 (5.09 to 9.36)	-2.80 (-5.24 to -1.01)	0.59 (0.44 to 0.80)
Italy	COPD	Total	19.7	33.9 (27.8 to 41.2)	-14.2 (-21.6 to -8.19)	0.58 (0.48 to 0.71)	18.1	32.3 (26.3 to 39.7)	-14.2 (-21.5 to -8.10)	0.56 (0.46 to 0.69)
Italy	COPD	Female	15.1	26.7 (22.4 to 31.8)	-11.6 (-16.7 to -7.37)	0.57 (0.48 to 0.67)	14.3	25.8 (21.6 to 31.0)	-11.5 (-16.6 to -7.24)	0.55 (0.46 to 0.66)
Italy	COPD	Male	26.3	44.2 (35.7 to 54.7)	-17.9 (-28.3 to -9.43)	0.59 (0.48 to 0.74)	23.5	41.9 (33.5 to 52.3)	-18.4 (-28.9 to -9.87)	0.56 (0.45 to 0.70)
Italy	Congestive heart failure and hypertension	Total	165	232 (194 to 277)	-67.0 (-112 to -28.9)	0.71 (0.60 to 0.85)	168	232 (181 to 299)	-64.3 (-130 to -12.6)	0.72 (0.56 to 0.93)
Italy	Congestive heart failure and hypertension	Female	135	189 (158 to 226)	-54.5 (-91.4 to -23.7)	0.71 (0.60 to 0.85)	136	189 (147 to 244)	-53.2 (-108 to -11.3)	0.72 (0.56 to 0.92)
Italy	Congestive heart failure and hypertension	Male	205	290 (243 to 347)	-84.9 (-141 to -37.8)	0.71 (0.59 to 0.84)	210	290 (226 to 374)	-80.5 (-163 to -14.7)	0.72 (0.56 to 0.93)
Italy	Congestive heart failure	Total	158	220 (185 to 263)	-62.5 (-105 to -27.0)	0.72 (0.60 to 0.85)	160	220 (172 to 282)	-60.1 (-123 to -11.0)	0.73 (0.57 to 0.94)
Italy	Congestive heart failure	Female	128	179 (150 to 213)	-50.5 (-84.1 to -22.2)	0.72 (0.60 to 0.85)	129	179 (139 to 229)	-49.4 (-100 to -11.4)	0.72 (0.56 to 0.92)
Italy	Congestive heart failure	Male	196	276 (231 to 331)	-80.0 (-135 to -35.2)	0.71 (0.59 to 0.85)	200	276 (215 to 356)	-76.0 (-153 to -14.5)	0.73 (0.57 to 0.93)
Italy	Diabetes	Total	31.1	39.7 (37.0 to 42.7)	-8.64 (-11.6 to -6.00)	0.78 (0.73 to 0.84)	31	37.1 (33.6 to 41.0)	-6.08 (-9.99 to -2.55)	0.84 (0.76 to 0.92)
Italy	Diabetes	Female	20.3	27.1 (26.3 to 28.0)	-6.84 (-7.68 to -6.05)	0.75 (0.73 to 0.77)	20.3	25.3 (23.7 to 27.1)	-5.01 (-6.75 to -3.43)	0.80 (0.75 to 0.86)
Italy	Diabetes	Male	43.8	55.7 (50.3 to 61.7)	-11.9 (-18.0 to -6.42)	0.79 (0.71 to 0.87)	43.8	52.7 (45.6 to 60.9)	-8.94 (-17.1 to -1.87)	0.83 (0.72 to 0.96)
Italy	Hypertension	Total	7.6	10.7 (8.23 to 13.9)	-3.11 (-6.27 to -0.619)	0.71 (0.55 to 0.92)	7.9	9.48 (6.53 to 13.8)	-1.58 (-5.88 to 1.30)	0.83 (0.57 to 1.20)
Italy	Hypertension	Female	6.5	9.07 (7.03 to 11.7)	-2.57 (-5.18 to -0.555)	0.72 (0.56 to 0.92)	6.6	7.91 (5.52 to 11.3)	-1.31 (-4.79 to 1.03)	0.83 (0.58 to 1.19)
Italy	Hypertension	Male	8.8	12.4 (9.39 to 16.3)	-3.59 (-7.54 to -0.612)	0.71 (0.54 to 0.93)	9.3	11.1 (7.51 to 16.5)	-1.82 (-7.25 to 1.80)	0.84 (0.56 to 1.24)
Korea	Asthma and COPD	Total	135	230 (195 to 271)	-95.2 (-136 to -60.6)	0.59 (0.50 to 0.69)	99.7	230 (182 to 290)	-130 (-190 to -81.3)	0.43 (0.34 to 0.55)
Korea	Asthma and COPD	Female	112	209 (174 to 251)	-96.9 (-139 to -62.1)	0.54 (0.45 to 0.64)	79	209 (161 to 270)	-130 (-192 to -81.4)	0.38 (0.29 to 0.49)
Korea	Asthma and COPD	Male	174	260 (229 to 294)	-86.0 (-120 to -55.3)	0.67 (0.59 to 0.76)	134	247 (207 to 294)	-113 (-161 to -73.5)	0.54 (0.45 to 0.65)
Korea	Asthma	Total	40.6	65.2 (57.9 to 73.6)	-24.6 (-32.9 to -17.3)	0.62 (0.55 to 0.70)	31.4	62.2 (52.4 to 73.7)	-30.8 (-42.5 to -21.0)	0.51 (0.42 to 0.60)
Korea	Asthma	Female	45.8	81.8 (70.7 to 94.7)	-36.0 (-48.9 to -25.0)	0.56 (0.48 to 0.65)	34.8	81.8 (66.5 to 101)	-47.0 (-66.0 to -31.9)	0.43 (0.35 to 0.52)
Korea	Asthma	Male	34.4	49.2 (44.1 to 54.8)	-14.8 (-20.4 to -9.52)	0.70 (0.63 to 0.78)	27.8	45.9 (39.3 to 53.5)	-18.1 (-25.7 to -11.4)	0.61 (0.52 to 0.71)
Korea	COPD	Total	94.1	162 (135 to 193)	-67.4 (-98.4 to -40.9)	0.58 (0.49 to 0.70)	68.3	162 (126 to 207)	-93.2 (-141 to -56.3)	0.42 (0.33 to 0.55)
Korea	COPD	Female	66	159 (131 to 193)	-93.2 (-127 to -65.3)	0.41 (0.34 to 0.50)	44.2	159 (131 to 193)	-115 (-148 to -87.9)	0.28 (0.23 to 0.33)

Korea	COPD	Male	139	211 (184 to 241)	-71.4 (-101 to -45.0)	0.66 (0.58 to 0.76)	106	201 (166 to 242)	-95.2 (-136 to -60.4)	0.53 (0.44 to 0.64)
Korea	Congestive heart failure and hypertension	Total	142	162 (150 to 174)	-20.0 (-32.1 to -8.09)	0.88 (0.82 to 0.95)	142	151 (136 to 168)	-9.48 (-26.4 to 5.48)	0.94 (0.84 to 1.04)
Korea	Congestive heart failure and hypertension	Female	143	167 (156 to 180)	-24.0 (-36.3 to -12.4)	0.86 (0.80 to 0.92)	143	155 (140 to 172)	-11.9 (-28.9 to 2.71)	0.92 (0.83 to 1.02)
Korea	Congestive heart failure and hypertension	Male	136	148 (136 to 160)	-11.8 (-24.8 to -0.456)	0.92 (0.85 to 1.00)	135	138 (115 to 165)	-2.33 (-30.2 to 20.8)	0.98 (0.82 to 1.18)
Korea	Congestive heart failure	Total	82.1	94.5 (87.8 to 102)	-12.4 (-19.5 to -5.61)	0.87 (0.81 to 0.94)	79.1	94.5 (85.1 to 105)	-15.4 (-25.7 to -5.98)	0.84 (0.75 to 0.93)
Korea	Congestive heart failure	Female	79.7	91.1 (84.9 to 97.8)	-11.4 (-18.0 to -5.25)	0.87 (0.82 to 0.94)	77.5	88.4 (80.0 to 97.7)	-10.9 (-20.1 to -2.37)	0.88 (0.79 to 0.97)
Korea	Congestive heart failure	Male	83.6	91.1 (84.4 to 98.5)	-7.55 (-14.8 to -0.727)	0.92 (0.85 to 0.99)	79.2	91.1 (84.4 to 98.5)	-11.9 (-19.3 to -5.28)	0.87 (0.80 to 0.94)
Korea	Diabetes	Total	201	226 (219 to 233)	-24.9 (-32.0 to -18.1)	0.89 (0.86 to 0.92)	196	216 (207 to 225)	-19.6 (-28.9 to -10.3)	0.91 (0.87 to 0.95)
Korea	Diabetes	Female	168	192 (187 to 198)	-23.6 (-29.0 to -18.4)	0.88 (0.85 to 0.90)	164	182 (175 to 189)	-17.9 (-25.2 to -10.9)	0.90 (0.87 to 0.94)
Korea	Diabetes	Male	237	263 (253 to 275)	-26.4 (-37.6 to -15.8)	0.90 (0.86 to 0.94)	232	253 (239 to 269)	-21.6 (-37.0 to -6.74)	0.91 (0.86 to 0.97)
Korea	Hypertension	Total	59.8	71.0 (65.0 to 77.4)	-11.2 (-17.6 to -5.31)	0.84 (0.77 to 0.92)	62.5	63.6 (56.2 to 71.9)	-1.08 (-9.55 to 6.45)	0.98 (0.87 to 1.12)
Korea	Hypertension	Female	63.5	77.2 (70.8 to 84.2)	-13.7 (-20.6 to -7.33)	0.82 (0.76 to 0.90)	65.8	69.3 (61.3 to 78.4)	-3.50 (-12.5 to 4.60)	0.95 (0.84 to 1.08)
Korea	Hypertension	Male	52.4	58.9 (53.2 to 65.2)	-6.48 (-12.8 to -0.737)	0.89 (0.80 to 0.99)	56	52.5 (45.5 to 60.7)	3.47 (-4.74 to 10.7)	1.07 (0.92 to 1.23)
Lithuania	Asthma and COPD	Total	117	219 (197 to 243)	-102 (-126 to -80.3)	0.53 (0.48 to 0.59)	86	207 (178 to 240)	-121 (-154 to -91.6)	0.42 (0.36 to 0.48)
Lithuania	Asthma and COPD	Female	69.6	146 (125 to 170)	-76.1 (-100 to -55.7)	0.48 (0.41 to 0.56)	49.2	136 (110 to 169)	-87.1 (-121 to -60.5)	0.36 (0.29 to 0.45)
Lithuania	Asthma and COPD	Male	180	321 (296 to 347)	-140 (-166 to -116)	0.56 (0.52 to 0.61)	135	305 (273 to 341)	-170 (-207 to -138)	0.44 (0.39 to 0.49)
Lithuania	Asthma	Total	20.1	40.1 (35.3 to 45.6)	-20.0 (-25.5 to -15.1)	0.50 (0.44 to 0.57)	14.4	37.7 (31.4 to 45.2)	-23.3 (-30.8 to -17.1)	0.38 (0.32 to 0.46)
Lithuania	Asthma	Female	24.2	52.3 (44.5 to 61.5)	-28.1 (-37.0 to -20.3)	0.46 (0.40 to 0.54)	16.7	52.3 (41.6 to 65.7)	-35.6 (-49.1 to -25.0)	0.32 (0.25 to 0.40)
Lithuania	Asthma	Male	15.1	31.5 (24.4 to 40.7)	-16.4 (-25.6 to -9.42)	0.48 (0.37 to 0.62)	11.3	31.5 (21.9 to 45.2)	-20.2 (-33.9 to -10.8)	0.36 (0.25 to 0.51)
Lithuania	COPD	Total	96.8	179 (162 to 198)	-82.1 (-102 to -65.1)	0.54 (0.49 to 0.60)	71.7	169 (146 to 195)	-97.4 (-123 to -74.9)	0.42 (0.37 to 0.49)
Lithuania	COPD	Female	45.4	104 (83.4 to 129)	-58.1 (-83.4 to -38.2)	0.44 (0.35 to 0.54)	32.4	104 (76.2 to 141)	-71.1 (-108 to -44.2)	0.31 (0.23 to 0.42)
Lithuania	COPD	Male	165	292 (272 to 312)	-126 (-147 to -107)	0.57 (0.53 to 0.61)	124	278 (253 to 307)	-155 (-184 to -129)	0.44 (0.40 to 0.49)
Lithuania	Congestive heart failure and hypertension	Total	483	703 (651 to 760)	-221 (-279 to -167)	0.69 (0.63 to 0.74)	418	675 (605 to 753)	-257 (-335 to -187)	0.62 (0.56 to 0.69)

Lithuania	Congestive heart failure and hypertension	Female	419	652 (601 to 706)	-232 (-288 to -182)	0.64 (0.59 to 0.70)	366	621 (554 to 696)	-256 (-329 to -189)	0.59 (0.53 to 0.66)
Lithuania	Congestive heart failure and hypertension	Male	556	782 (707 to 865)	-226 (-310 to -150)	0.71 (0.64 to 0.79)	478	782 (678 to 901)	-304 (-423 to -200)	0.61 (0.53 to 0.71)
Lithuania	Congestive heart failure	Total	363	479 (444 to 516)	-116 (-153 to -81.0)	0.76 (0.70 to 0.82)	316	452 (406 to 503)	-136 (-188 to -89.6)	0.70 (0.63 to 0.78)
Lithuania	Congestive heart failure	Female	289	405 (373 to 440)	-116 (-151 to -83.4)	0.71 (0.66 to 0.78)	256	377 (335 to 424)	-121 (-167 to -78.2)	0.68 (0.61 to 0.77)
Lithuania	Congestive heart failure	Male	454	571 (521 to 625)	-116 (-170 to -65.6)	0.80 (0.73 to 0.87)	389	546 (480 to 621)	-157 (-231 to -89.4)	0.71 (0.63 to 0.81)
Lithuania	Diabetes	Total	146	237 (211 to 267)	-90.8 (-121 to -64.0)	0.62 (0.55 to 0.70)	152	237 (211 to 267)	-85.0 (-115 to -58.4)	0.64 (0.57 to 0.72)
Lithuania	Diabetes	Female	116	212 (177 to 253)	-95.3 (-137 to -61.4)	0.55 (0.46 to 0.65)	115	212 (177 to 253)	-96.6 (-138 to -62.0)	0.54 (0.45 to 0.65)
Lithuania	Diabetes	Male	179	262 (235 to 293)	-83.7 (-114 to -56.4)	0.68 (0.61 to 0.76)	192	262 (235 to 293)	-69.9 (-100 to -42.8)	0.73 (0.66 to 0.82)
Lithuania	Hypertension	Total	120	225 (201 to 252)	-105 (-132 to -80.7)	0.53 (0.48 to 0.60)	102	225 (192 to 264)	-123 (-161 to -89.8)	0.45 (0.39 to 0.53)
Lithuania	Hypertension	Female	130	236 (205 to 271)	-105 (-140 to -74.6)	0.55 (0.48 to 0.64)	109	236 (205 to 271)	-126 (-162 to -96.3)	0.46 (0.40 to 0.53)
Lithuania	Hypertension	Male	101	185 (176 to 196)	-84.1 (-94.5 to -74.4)	0.55 (0.52 to 0.58)	88.8	185 (172 to 200)	-96.6 (-111 to -83.2)	0.48 (0.44 to 0.52)
Latvia	Asthma and COPD	Total	161	272 (240 to 309)	-111 (-147 to -79.3)	0.59 (0.52 to 0.67)	95.9	272 (240 to 309)	-176 (-214 to -145)	0.35 (0.31 to 0.40)
Latvia	Asthma and COPD	Female	116	205 (179 to 235)	-88.8 (-119 to -62.4)	0.57 (0.49 to 0.65)	58.5	205 (179 to 235)	-147 (-176 to -120)	0.29 (0.25 to 0.33)
Latvia	Asthma and COPD	Male	222	363 (322 to 411)	-142 (-188 to -99.9)	0.61 (0.54 to 0.69)	146	363 (322 to 411)	-218 (-266 to -176)	0.40 (0.35 to 0.45)
Latvia	Asthma	Total	60	101 (85.9 to 119)	-41.2 (-58.8 to -25.9)	0.59 (0.51 to 0.70)	29.9	101 (85.9 to 119)	-71.3 (-89.5 to -56.0)	0.30 (0.25 to 0.35)
Latvia	Asthma	Female	66.1	113 (95.3 to 135)	-47.2 (-68.6 to -29.0)	0.58 (0.49 to 0.69)	32.5	113 (95.3 to 135)	-80.8 (-102 to -62.9)	0.29 (0.24 to 0.34)
Latvia	Asthma	Male	54.1	88.7 (74.3 to 106)	-34.6 (-51.8 to -20.0)	0.61 (0.51 to 0.73)	28	88.7 (74.3 to 106)	-60.7 (-77.4 to -46.2)	0.32 (0.27 to 0.38)
Latvia	COPD	Total	101	171 (153 to 191)	-70.0 (-90.2 to -51.8)	0.59 (0.53 to 0.66)	65.9	171 (153 to 191)	-105 (-125 to -87.0)	0.38 (0.34 to 0.43)
Latvia	COPD	Female	50.1	91.6 (80.0 to 105)	-41.5 (-54.5 to -29.8)	0.55 (0.48 to 0.63)	26	91.6 (80.0 to 105)	-65.6 (-79.0 to -53.9)	0.28 (0.25 to 0.33)
Latvia	COPD	Male	168	274 (243 to 310)	-107 (-141 to -76.2)	0.61 (0.54 to 0.69)	118	274 (243 to 310)	-157 (-192 to -126)	0.43 (0.38 to 0.48)
Latvia	Diabetes	Total	123	131 (117 to 147)	-7.47 (-23.1 to 6.81)	0.94 (0.84 to 1.06)	100	131 (117 to 147)	-30.8 (-46.6 to -16.8)	0.76 (0.68 to 0.86)
Latvia	Diabetes	Female	104	120 (106 to 135)	-15.9 (-32.1 to -1.99)	0.87 (0.76 to 0.98)	84.5	120 (106 to 135)	-35.1 (-50.7 to -20.9)	0.71 (0.63 to 0.80)
Latvia	Diabetes	Male	143	156 (141 to 174)	-13.5 (-30.9 to 2.41)	0.91 (0.82 to 1.02)	115	156 (135 to 181)	-41.1 (-65.7 to -19.3)	0.74 (0.64 to 0.86)
Mexico	Asthma and COPD	Total	30.9	75.5 (67.6 to 84.3)	-44.6 (-53.3 to -36.6)	0.41 (0.37 to 0.46)	18.3	70.9 (60.6 to 82.9)	-52.6 (-64.5 to -42.4)	0.26 (0.22 to 0.30)
Mexico	Asthma and COPD	Female	29.8	76.9 (69.0 to 85.6)	-47.1 (-55.9 to -39.1)	0.39 (0.35 to 0.43)	17.4	72.1 (62.0 to 84.0)	-54.7 (-66.6 to -44.5)	0.24 (0.21 to 0.28)

Mexico	Asthma and COPD	Male	32.8	75.0 (67.0 to 84.0)	-42.2 (-51.3 to -34.1)	0.44 (0.39 to 0.49)	19.9	70.3 (59.9 to 82.5)	-50.4 (-62.8 to -40.0)	0.28 (0.24 to 0.33)
Mexico	Asthma	Total	2.8	6.54 (5.44 to 7.87)	-3.74 (-5.03 to -2.63)	0.43 (0.36 to 0.52)	2.2	5.86 (4.51 to 7.61)	-3.66 (-5.45 to -2.33)	0.38 (0.29 to 0.49)
Mexico	Asthma	Female	3.8	9.26 (7.93 to 10.8)	-5.46 (-7.03 to -4.16)	0.41 (0.35 to 0.48)	3	8.32 (6.68 to 10.4)	-5.32 (-7.36 to -3.72)	0.36 (0.29 to 0.45)
Mexico	Asthma	Male	1.6	3.74 (2.84 to 4.93)	-2.14 (-3.34 to -1.24)	0.43 (0.32 to 0.56)	1.5	3.33 (2.26 to 4.92)	-1.83 (-3.45 to -0.756)	0.45 (0.30 to 0.66)
Mexico	COPD	Total	28.1	69.0 (61.4 to 77.6)	-40.9 (-49.5 to -33.4)	0.41 (0.36 to 0.46)	16.1	65.2 (55.3 to 76.9)	-49.1 (-60.9 to -39.1)	0.25 (0.21 to 0.29)
Mexico	COPD	Female	26	67.7 (60.1 to 76.3)	-41.7 (-50.4 to -34.2)	0.38 (0.34 to 0.43)	14.4	64.1 (54.1 to 75.9)	-49.7 (-61.8 to -39.8)	0.22 (0.19 to 0.27)
Mexico	COPD	Male	31.2	71.4 (63.9 to 79.9)	-40.2 (-48.7 to -32.6)	0.44 (0.39 to 0.49)	18.4	67.2 (57.4 to 78.7)	-48.8 (-60.5 to -39.1)	0.27 (0.23 to 0.32)
Mexico	Congestive heart failure and hypertension	Total	66.7	104 (97.3 to 111)	-37.3 (-44.2 to -30.6)	0.64 (0.60 to 0.69)	67.6	92.6 (79.9 to 107)	-25.0 (-39.6 to -12.6)	0.73 (0.63 to 0.84)
Mexico	Congestive heart failure and hypertension	Female	65.3	112 (104 to 120)	-46.7 (-55.0 to -39.1)	0.58 (0.54 to 0.63)	65.6	106 (96.2 to 118)	-40.9 (-52.2 to -30.7)	0.62 (0.56 to 0.68)
Mexico	Congestive heart failure and hypertension	Male	67.7	110 (102 to 120)	-42.7 (-51.9 to -34.4)	0.61 (0.57 to 0.66)	69.2	106 (94.8 to 119)	-37.0 (-50.0 to -25.7)	0.65 (0.58 to 0.73)
Mexico	Congestive heart failure	Total	32.5	56.6 (54.5 to 58.7)	-24.1 (-26.2 to -22.0)	0.57 (0.55 to 0.60)	34.9	55.0 (52.2 to 57.9)	-20.1 (-23.1 to -17.4)	0.63 (0.60 to 0.67)
Mexico	Congestive heart failure	Female	30.8	55.2 (53.1 to 57.3)	-24.4 (-26.4 to -22.3)	0.56 (0.54 to 0.58)	32.9	53.5 (50.7 to 56.4)	-20.6 (-23.5 to -17.8)	0.62 (0.58 to 0.65)
Mexico	Congestive heart failure	Male	34.2	57.5 (54.7 to 60.4)	-23.3 (-26.2 to -20.6)	0.60 (0.57 to 0.62)	36.8	56.0 (52.2 to 60.0)	-19.2 (-23.1 to -15.4)	0.66 (0.61 to 0.70)
Mexico	Diabetes	Total	143	207 (196 to 219)	-64.2 (-76.1 to -52.8)	0.69 (0.65 to 0.73)	142	186 (164 to 210)	-43.7 (-68.1 to -22.5)	0.76 (0.68 to 0.86)
Mexico	Diabetes	Female	122	184 (174 to 194)	-61.3 (-71.5 to -51.8)	0.67 (0.63 to 0.70)	119	163 (145 to 183)	-43.5 (-64.3 to -25.8)	0.73 (0.65 to 0.82)
Mexico	Diabetes	Male	167	234 (221 to 247)	-67.2 (-80.0 to -54.9)	0.71 (0.68 to 0.75)	168	212 (188 to 239)	-44.2 (-71.0 to -20.3)	0.79 (0.70 to 0.89)
Mexico	Hypertension	Total	34.1	49.0 (44.8 to 53.5)	-14.9 (-19.5 to -10.7)	0.70 (0.64 to 0.76)	32.6	41.0 (33.6 to 49.9)	-8.35 (-17.2 to -1.10)	0.80 (0.65 to 0.97)
Mexico	Hypertension	Female	34.5	51.1 (46.6 to 56.1)	-16.6 (-21.6 to -12.0)	0.67 (0.61 to 0.74)	32.7	43.0 (34.9 to 53.0)	-10.3 (-20.2 to -2.18)	0.76 (0.62 to 0.94)
Mexico	Hypertension	Male	33.5	46.4 (42.0 to 51.2)	-12.9 (-17.6 to -8.58)	0.72 (0.66 to 0.80)	32.4	38.6 (31.0 to 48.2)	-6.21 (-15.6 to 1.43)	0.84 (0.68 to 1.05)
Netherlands	Asthma and COPD	Total	138	229 (202 to 260)	-91.2 (-122 to -64.4)	0.60 (0.53 to 0.68)	125	227 (192 to 268)	-102 (-143 to -66.8)	0.55 (0.47 to 0.65)
Netherlands	Asthma and COPD	Female	148	242 (211 to 277)	-93.8 (-129 to -63.0)	0.61 (0.53 to 0.70)	136	242 (200 to 293)	-105 (-157 to -63.3)	0.56 (0.47 to 0.68)
Netherlands	Asthma and COPD	Male	132	216 (201 to 232)	-84.4 (-101 to -69.5)	0.61 (0.57 to 0.65)	116	232 (208 to 260)	-116 (-144 to -91.8)	0.50 (0.45 to 0.56)
Netherlands	Asthma	Total	20.3	34.5 (30.7 to 38.8)	-14.2 (-18.5 to -10.4)	0.59 (0.52 to 0.66)	17.7	34.5 (30.7 to 38.8)	-16.8 (-21.0 to -13.0)	0.51 (0.46 to 0.58)
Netherlands	Asthma	Female	27.3	47.3 (41.6 to 53.7)	-20.0 (-26.4 to -14.4)	0.58 (0.51 to 0.66)	23.3	47.3 (41.6 to 53.7)	-24.0 (-30.5 to -18.3)	0.49 (0.43 to 0.56)

Netherlands	Asthma	Male	12.5	20.4 (17.9 to 23.3)	-7.94 (-10.8 to -5.42)	0.61 (0.54 to 0.70)	11.6	20.4 (17.9 to 23.3)	-8.84 (-11.8 to -6.30)	0.57 (0.50 to 0.65)
Netherlands	COPD	Total	118	188 (162 to 219)	-70.9 (-101 to -44.6)	0.62 (0.54 to 0.72)	107	188 (157 to 226)	-80.8 (-119 to -49.8)	0.57 (0.47 to 0.68)
Netherlands	COPD	Female	121	196 (168 to 229)	-75.3 (-108 to -47.8)	0.62 (0.53 to 0.72)	113	196 (157 to 244)	-82.8 (-131 to -43.7)	0.58 (0.46 to 0.72)
Netherlands	COPD	Male	119	196 (178 to 215)	-76.8 (-96.3 to -58.7)	0.61 (0.55 to 0.67)	105	210 (182 to 242)	-105 (-139 to -76.9)	0.50 (0.43 to 0.58)
Netherlands	Congestive heart failure and hypertension	Total	156	176 (151 to 206)	-20.7 (-49.7 to 4.54)	0.88 (0.76 to 1.03)	160	176 (142 to 219)	-17.0 (-59.1 to 17.4)	0.90 (0.73 to 1.12)
Netherlands	Congestive heart failure and hypertension	Female	135	152 (131 to 177)	-17.2 (-41.8 to 4.01)	0.89 (0.76 to 1.03)	140	152 (123 to 188)	-12.4 (-49.4 to 16.8)	0.92 (0.74 to 1.14)
Netherlands	Congestive heart failure and hypertension	Male	185	210 (180 to 246)	-25.7 (-60.8 to 5.10)	0.88 (0.75 to 1.03)	187	210 (168 to 263)	-23.3 (-74.5 to 17.6)	0.89 (0.72 to 1.10)
Netherlands	Congestive heart failure	Total	143	162 (139 to 188)	-19.1 (-45.1 to 3.64)	0.88 (0.76 to 1.03)	148	162 (130 to 201)	-13.9 (-53.7 to 17.2)	0.91 (0.73 to 1.13)
Netherlands	Congestive heart failure	Female	120	135 (117 to 157)	-15.2 (-37.1 to 3.56)	0.89 (0.76 to 1.03)	127	135 (110 to 167)	-8.30 (-40.2 to 17.3)	0.94 (0.76 to 1.16)
Netherlands	Congestive heart failure	Male	174	198 (170 to 231)	-24.4 (-57.7 to 3.82)	0.88 (0.75 to 1.02)	176	198 (159 to 247)	-21.5 (-70.1 to 17.5)	0.89 (0.72 to 1.11)
Netherlands	Diabetes	Total	47	50.7 (46.6 to 55.3)	-3.73 (-8.34 to 0.449)	0.93 (0.85 to 1.01)	45.1	48.7 (43.1 to 54.9)	-3.56 (-9.81 to 1.86)	0.93 (0.82 to 1.04)
Netherlands	Diabetes	Female	32.3	36.0 (33.7 to 38.5)	-3.70 (-6.16 to -1.35)	0.90 (0.84 to 0.96)	33	34.5 (32.2 to 36.9)	-1.46 (-3.90 to 0.863)	0.96 (0.89 to 1.03)
Netherlands	Diabetes	Male	64.6	69.5 (62.2 to 77.7)	-4.90 (-13.0 to 2.35)	0.93 (0.83 to 1.04)	60	69.5 (59.4 to 81.3)	-9.50 (-21.2 to 0.636)	0.86 (0.74 to 1.01)
Netherlands	Hypertension	Total	13.2	14.9 (12.2 to 18.2)	-1.70 (-4.92 to 0.922)	0.89 (0.73 to 1.08)	11.8	14.9 (11.2 to 19.8)	-3.10 (-7.93 to 0.637)	0.79 (0.60 to 1.06)
Netherlands	Hypertension	Female	14.8	16.7 (13.8 to 20.2)	-1.90 (-5.38 to 1.01)	0.89 (0.73 to 1.07)	12.6	16.7 (12.7 to 21.9)	-4.10 (-9.25 to -0.145)	0.75 (0.58 to 0.99)
Netherlands	Hypertension	Male	11	12.3 (9.83 to 15.4)	-1.30 (-4.45 to 1.16)	0.89 (0.71 to 1.12)	10.4	12.3 (8.95 to 16.9)	-1.90 (-6.49 to 1.46)	0.85 (0.62 to 1.16)
Norway	Asthma and COPD	Total	208	259 (247 to 271)	-51.6 (-64.1 to -40.3)	0.80 (0.76 to 0.84)	212	259 (243 to 277)	-47.3 (-64.8 to -30.8)	0.82 (0.77 to 0.87)
Norway	Asthma and COPD	Female	217	286 (267 to 307)	-69.2 (-89.7 to -49.6)	0.76 (0.71 to 0.81)	224	286 (267 to 307)	-61.7 (-82.8 to -42.1)	0.78 (0.73 to 0.84)
Norway	Asthma and COPD	Male	200	245 (225 to 267)	-45.0 (-67.3 to -24.6)	0.82 (0.75 to 0.89)	201	245 (217 to 277)	-44.5 (-76.7 to -15.1)	0.82 (0.72 to 0.93)
Norway	Asthma	Total	19.6	20.1 (18.4 to 22.1)	-0.541 (-2.46 to 1.23)	0.97 (0.89 to 1.07)	18.4	18.9 (16.6 to 21.5)	-0.467 (-3.02 to 1.79)	0.98 (0.86 to 1.11)
Norway	Asthma	Female	24.4	26.9 (24.1 to 30.1)	-2.53 (-5.77 to 0.287)	0.91 (0.81 to 1.01)	22.1	25.4 (21.6 to 29.7)	-3.25 (-7.57 to 0.456)	0.87 (0.74 to 1.02)
Norway	Asthma	Male	14	12.6 (10.6 to 15.0)	1.39 (-1.06 to 3.36)	1.11 (0.93 to 1.32)	14.1	11.7 (9.13 to 15.0)	2.40 (-0.788 to 4.92)	1.21 (0.95 to 1.54)
Norway	COPD	Total	188	243 (229 to 258)	-55.3 (-69.9 to -41.6)	0.77 (0.73 to 0.82)	193	243 (229 to 258)	-49.8 (-64.4 to -36.0)	0.80 (0.75 to 0.84)
Norway	COPD	Female	193	247 (232 to 263)	-54.2 (-69.7 to -38.9)	0.78 (0.73 to 0.83)	202	247 (232 to 263)	-44.4 (-60.3 to -29.8)	0.82 (0.77 to 0.87)

Norway	COPD	Male	186	232 (213 to 252)	-45.3 (-65.6 to -26.9)	0.80 (0.74 to 0.87)	187	232 (206 to 260)	-44.9 (-73.7 to -19.3)	0.81 (0.72 to 0.91)
Norway	Congestive heart failure and hypertension	Total	219	223 (205 to 241)	-3.70 (-22.2 to 13.5)	0.98 (0.91 to 1.07)	233	223 (205 to 241)	9.90 (-8.65 to 27.3)	1.04 (0.96 to 1.13)
Norway	Congestive heart failure and hypertension	Female	179	179 (164 to 195)	0.346 (-15.8 to 15.0)	1.00 (0.92 to 1.09)	190	179 (164 to 195)	11.2 (-4.65 to 25.9)	1.06 (0.98 to 1.16)
Norway	Congestive heart failure and hypertension	Male	275	282 (259 to 308)	-7.71 (-33.1 to 16.2)	0.97 (0.89 to 1.06)	292	282 (259 to 308)	9.79 (-16.1 to 33.3)	1.03 (0.95 to 1.13)
Norway	Congestive heart failure	Total	183	178 (166 to 191)	5.02 (-7.99 to 16.9)	1.03 (0.96 to 1.10)	195	178 (166 to 191)	17.0 (4.13 to 28.7)	1.10 (1.02 to 1.17)
Norway	Congestive heart failure	Female	138	131 (120 to 143)	7.04 (-4.98 to 18.1)	1.05 (0.97 to 1.15)	148	131 (120 to 143)	17.1 (5.10 to 28.3)	1.13 (1.04 to 1.24)
Norway	Congestive heart failure	Male	247	254 (248 to 260)	-6.89 (-12.7 to -1.05)	0.97 (0.95 to 1.00)	263	253 (247 to 259)	9.73 (3.88 to 15.5)	1.04 (1.01 to 1.06)
Norway	Diabetes	Total	68.6	71.3 (65.2 to 78.0)	-2.70 (-9.53 to 3.41)	0.96 (0.88 to 1.05)	70.4	71.3 (62.8 to 80.9)	-0.900 (-10.5 to 7.48)	0.99 (0.87 to 1.12)
Norway	Diabetes	Female	55.3	57.1 (50.7 to 64.3)	-1.80 (-8.89 to 4.67)	0.97 (0.86 to 1.09)	57.6	57.1 (48.2 to 67.6)	0.500 (-9.96 to 9.48)	1.01 (0.85 to 1.20)
Norway	Diabetes	Male	84.5	88.5 (80.8 to 96.9)	-4.00 (-12.3 to 3.68)	0.95 (0.87 to 1.05)	85.3	88.5 (77.8 to 101)	-3.20 (-15.5 to 7.58)	0.96 (0.85 to 1.10)
Norway	Hypertension	Total	36	37.2 (31.8 to 43.5)	-1.20 (-7.42 to 4.28)	0.97 (0.83 to 1.13)	37.6	37.2 (29.8 to 46.4)	0.400 (-8.80 to 7.85)	1.01 (0.81 to 1.26)
Norway	Hypertension	Female	41.2	41.9 (35.9 to 48.9)	-0.700 (-7.73 to 5.24)	0.98 (0.84 to 1.15)	42	41.9 (33.6 to 52.2)	0.100 (-10.0 to 8.36)	1.00 (0.81 to 1.25)
Norway	Hypertension	Male	27.4	29.1 (24.1 to 35.1)	-1.70 (-7.67 to 3.30)	0.94 (0.78 to 1.14)	29.5	29.1 (22.3 to 38.0)	0.400 (-8.52 to 7.21)	1.01 (0.78 to 1.32)
Poland	Asthma and COPD	Total	98.3	170 (159 to 182)	-71.8 (-84.0 to -60.5)	0.58 (0.54 to 0.62)	82.8	153 (131 to 179)	-70.0 (-95.3 to -48.4)	0.54 (0.47 to 0.63)
Poland	Asthma and COPD	Female	81.3	148 (137 to 159)	-66.2 (-77.8 to -55.8)	0.55 (0.51 to 0.59)	71.4	133 (113 to 157)	-61.7 (-86.3 to -41.5)	0.54 (0.45 to 0.63)
Poland	Asthma and COPD	Male	122	203 (188 to 219)	-80.6 (-96.2 to -66.1)	0.60 (0.56 to 0.65)	99.1	181 (153 to 214)	-82.1 (-115 to -54.7)	0.55 (0.46 to 0.64)
Poland	Asthma	Total	37.2	67.6 (63.3 to 72.2)	-30.4 (-34.9 to -26.1)	0.55 (0.52 to 0.59)	31.1	66.0 (58.3 to 74.8)	-34.9 (-43.7 to -27.2)	0.47 (0.42 to 0.53)
Poland	Asthma	Female	42.1	86.4 (76.7 to 97.2)	-44.3 (-55.3 to -34.7)	0.49 (0.43 to 0.55)	36.2	87.4 (75.2 to 102)	-51.2 (-65.4 to -39.1)	0.41 (0.36 to 0.48)
Poland	Asthma	Male	31.4	58.1 (50.5 to 67.0)	-26.7 (-35.6 to -19.1)	0.54 (0.47 to 0.62)	25.1	58.2 (48.6 to 69.8)	-33.1 (-44.9 to -23.5)	0.43 (0.36 to 0.52)
Poland	COPD	Total	61.1	109 (100 to 119)	-48.4 (-58.2 to -39.3)	0.56 (0.51 to 0.61)	51.7	103 (90.8 to 116)	-50.9 (-64.4 to -39.2)	0.50 (0.45 to 0.57)
Poland	COPD	Female	39.2	74.2 (67.7 to 81.3)	-35.0 (-42.2 to -28.5)	0.53 (0.48 to 0.58)	35.2	70.1 (61.6 to 79.8)	-34.9 (-44.7 to -26.4)	0.50 (0.44 to 0.57)
Poland	COPD	Male	91	149 (136 to 165)	-58.4 (-73.7 to -44.8)	0.61 (0.55 to 0.67)	74	132 (106 to 164)	-57.7 (-89.9 to -32.0)	0.56 (0.45 to 0.70)
Poland	Congestive heart failure and hypertension	Total	545	769 (698 to 848)	-224 (-305 to -151)	0.71 (0.64 to 0.78)	537	769 (698 to 848)	-232 (-312 to -161)	0.70 (0.63 to 0.77)

Poland	Congestive heart failure and hypertension	Female	423	656 (592 to 727)	-232 (-303 to -168)	0.65 (0.58 to 0.72)	418	656 (592 to 727)	-238 (-309 to -173)	0.64 (0.57 to 0.71)
Poland	Congestive heart failure and hypertension	Male	696	985 (886 to 1097)	-289 (-400 to -192)	0.71 (0.63 to 0.78)	685	985 (847 to 1146)	-300 (-463 to -164)	0.70 (0.60 to 0.81)
Poland	Congestive heart failure	Total	465	661 (580 to 753)	-196 (-289 to -114)	0.70 (0.62 to 0.80)	459	661 (549 to 796)	-202 (-336 to -89.0)	0.69 (0.58 to 0.84)
Poland	Congestive heart failure	Female	341	488 (428 to 555)	-147 (-213 to -87.4)	0.70 (0.62 to 0.80)	337	478 (410 to 558)	-142 (-220 to -74.4)	0.70 (0.61 to 0.82)
Poland	Congestive heart failure	Male	622	862 (755 to 986)	-240 (-365 to -134)	0.72 (0.63 to 0.82)	612	862 (714 to 1042)	-251 (-434 to -101)	0.71 (0.59 to 0.86)
Poland	Diabetes	Total	120	184 (172 to 196)	-64.0 (-75.6 to -52.4)	0.65 (0.61 to 0.70)	121	178 (162 to 194)	-56.4 (-73.3 to -41.0)	0.68 (0.62 to 0.75)
Poland	Diabetes	Female	93.2	149 (140 to 159)	-56.0 (-65.9 to -46.7)	0.62 (0.59 to 0.67)	94.3	143 (130 to 156)	-48.5 (-62.1 to -36.4)	0.66 (0.60 to 0.72)
Poland	Diabetes	Male	148	221 (207 to 236)	-72.8 (-87.2 to -59.4)	0.67 (0.63 to 0.71)	150	216 (197 to 236)	-65.3 (-85.8 to -46.4)	0.70 (0.64 to 0.76)
Poland	Hypertension	Total	79.7	127 (114 to 140)	-46.9 (-60.6 to -34.5)	0.63 (0.57 to 0.70)	78.5	120 (103 to 138)	-41.1 (-59.9 to -24.8)	0.66 (0.57 to 0.76)
Poland	Hypertension	Female	82.4	131 (117 to 148)	-49.0 (-65.2 to -34.2)	0.63 (0.56 to 0.71)	80.8	124 (105 to 146)	-42.8 (-65.1 to -23.9)	0.65 (0.55 to 0.77)
Poland	Hypertension	Male	74.2	117 (107 to 127)	-42.5 (-53.2 to -33.0)	0.64 (0.58 to 0.69)	73.3	111 (98.0 to 125)	-37.5 (-52.2 to -24.6)	0.66 (0.58 to 0.75)
Portugal	Asthma and COPD	Total	63.8	90.7 (74.2 to 111)	-26.9 (-46.8 to -10.4)	0.70 (0.58 to 0.86)	53.8	90.7 (74.2 to 111)	-36.9 (-57.3 to -20.7)	0.59 (0.48 to 0.72)
Portugal	Asthma and COPD	Female	44.6	68.6 (59.6 to 78.8)	-24.0 (-34.3 to -15.1)	0.65 (0.56 to 0.75)	36.6	68.6 (59.6 to 78.8)	-32.0 (-42.4 to -23.1)	0.53 (0.46 to 0.61)
Portugal	Asthma and COPD	Male	89.2	120 (93.8 to 155)	-31.3 (-65.3 to -4.37)	0.74 (0.58 to 0.95)	76.1	120 (93.8 to 155)	-44.4 (-79.3 to -17.0)	0.63 (0.49 to 0.82)
Portugal	Asthma	Total	10	13.3 (11.1 to 15.9)	-3.30 (-5.82 to -1.13)	0.75 (0.63 to 0.90)	9.3	13.3 (10.3 to 17.1)	-4.00 (-7.85 to -1.02)	0.70 (0.54 to 0.90)
Portugal	Asthma	Female	12.9	18.6 (15.9 to 21.8)	-5.70 (-8.91 to -3.00)	0.69 (0.59 to 0.81)	11.3	18.6 (14.8 to 23.3)	-7.30 (-12.0 to -3.60)	0.61 (0.49 to 0.76)
Portugal	Asthma	Male	6.5	7.10 (5.47 to 9.21)	-0.600 (-2.75 to 1.05)	0.92 (0.70 to 1.19)	6.7	7.10 (4.91 to 10.3)	-0.400 (-3.57 to 1.78)	0.94 (0.65 to 1.36)
Portugal	COPD	Total	53.8	75.3 (56.9 to 99.6)	-21.5 (-45.8 to -3.18)	0.71 (0.54 to 0.94)	44.5	75.3 (56.9 to 99.6)	-30.8 (-55.7 to -12.6)	0.59 (0.44 to 0.78)
Portugal	COPD	Female	31.7	47.8 (36.8 to 62.1)	-16.1 (-30.6 to -5.11)	0.66 (0.51 to 0.86)	25.2	47.8 (36.8 to 62.1)	-22.6 (-37.0 to -11.5)	0.53 (0.41 to 0.69)
Portugal	COPD	Male	82.6	111 (83.3 to 149)	-28.8 (-66.5 to -0.366)	0.74 (0.55 to 1.00)	69.4	111 (83.3 to 149)	-42.0 (-79.2 to -14.0)	0.62 (0.47 to 0.83)
Portugal	Congestive heart failure and hypertension	Total	169	102 (69.5 to 149)	66.7 (19.4 to 99.1)	1.65 (1.13 to 2.43)	167	102 (59.3 to 175)	65.5 (-5.35 to 108)	1.64 (0.97 to 2.83)
Portugal	Congestive heart failure and hypertension	Female	150	85.4 (56.6 to 129)	64.5 (20.8 to 93.5)	1.76 (1.16 to 2.66)	153	85.4 (47.7 to 153)	67.5 (-2.38 to 105)	1.79 (0.98 to 3.20)
Portugal	Congestive heart failure and hypertension	Male	190	121 (84.6 to 174)	68.7 (16.0 to 106)	1.57 (1.09 to 2.26)	183	121 (72.9 to 202)	61.7 (-16.6 to 110)	1.51 (0.92 to 2.50)

Portugal	Congestive heart failure	Total	161	93.9 (63.5 to 139)	67.1 (22.6 to 97.6)	1.71 (1.16 to 2.54)	159	93.9 (54.0 to 163)	64.9 (-3.61 to 106)	1.69 (0.98 to 2.99)
Portugal	Congestive heart failure	Female	143	78.7 (51.5 to 120)	64.5 (22.1 to 91.7)	1.82 (1.18 to 2.78)	145	78.7 (43.2 to 143)	66.0 (2.14 to 102)	1.84 (1.01 to 3.39)
Portugal	Congestive heart failure	Male	181	112 (77.8 to 160)	69.4 (22.5 to 103)	1.62 (1.14 to 2.33)	174	112 (67.0 to 186)	61.9 (-14.8 to 107)	1.55 (0.92 to 2.62)
Portugal	Diabetes	Total	54.1	59.7 (49.6 to 71.9)	-5.60 (-17.4 to 4.58)	0.91 (0.76 to 1.09)	53.4	59.7 (45.9 to 77.7)	-6.30 (-24.1 to 7.31)	0.89 (0.69 to 1.16)
Portugal	Diabetes	Female	42.9	43.7 (38.7 to 49.4)	-0.822 (-6.42 to 4.15)	0.98 (0.87 to 1.11)	42	40.2 (33.9 to 47.8)	1.76 (-5.71 to 8.10)	1.04 (0.88 to 1.24)
Portugal	Diabetes	Male	67	73.7 (60.9 to 89.2)	-6.70 (-22.6 to 6.36)	0.91 (0.75 to 1.10)	66.4	73.7 (56.2 to 96.6)	-7.30 (-30.2 to 10.3)	0.90 (0.69 to 1.18)
Portugal	Hypertension	Total	7.7	8.00 (5.23 to 12.2)	-0.300 (-4.55 to 2.47)	0.96 (0.63 to 1.47)	8.7	8.00 (4.38 to 14.6)	0.700 (-5.98 to 4.34)	1.09 (0.59 to 1.99)
Portugal	Hypertension	Female	6.7	6.80 (4.24 to 10.9)	-0.100 (-4.27 to 2.45)	0.99 (0.61 to 1.58)	8.2	6.80 (3.49 to 13.3)	1.40 (-4.98 to 4.73)	1.21 (0.62 to 2.36)
Portugal	Hypertension	Male	8.9	9.60 (6.40 to 14.4)	-0.700 (-5.47 to 2.52)	0.93 (0.62 to 1.40)	9.4	9.60 (5.42 to 17.0)	-0.200 (-7.72 to 3.99)	0.98 (0.55 to 1.74)
Singapore	Asthma and COPD	Total	166	267 (253 to 282)	-102 (-116 to -87.1)	0.62 (0.59 to 0.66)	142	267 (239 to 299)	-125 (-157 to -96.9)	0.53 (0.48 to 0.59)
Singapore	Asthma and COPD	Female	116	215 (200 to 231)	-98.4 (-114 to -83.5)	0.54 (0.50 to 0.58)	101	215 (200 to 231)	-114 (-130 to -98.8)	0.47 (0.44 to 0.51)
Singapore	Asthma and COPD	Male	236	352 (316 to 392)	-116 (-156 to -79.6)	0.67 (0.60 to 0.75)	201	352 (302 to 410)	-151 (-208 to -101)	0.57 (0.49 to 0.66)
Singapore	Asthma	Total	61.1	115 (107 to 123)	-53.6 (-62.0 to -45.8)	0.53 (0.50 to 0.57)	49.1	115 (104 to 127)	-65.6 (-77.5 to -54.9)	0.43 (0.39 to 0.47)
Singapore	Asthma	Female	70.6	145 (130 to 162)	-74.7 (-91.9 to -59.6)	0.49 (0.43 to 0.54)	55.6	145 (125 to 169)	-89.7 (-114 to -69.3)	0.38 (0.33 to 0.45)
Singapore	Asthma	Male	48.8	76.0 (69.9 to 82.7)	-27.2 (-34.0 to -21.2)	0.64 (0.59 to 0.70)	40.8	76.0 (69.9 to 82.7)	-35.2 (-42.0 to -29.2)	0.54 (0.49 to 0.58)
Singapore	COPD	Total	104	152 (139 to 165)	-47.2 (-60.8 to -34.9)	0.69 (0.63 to 0.75)	92.8	147 (130 to 166)	-53.8 (-72.8 to -36.7)	0.63 (0.56 to 0.72)
Singapore	COPD	Female	45.9	71.0 (62.3 to 80.9)	-25.1 (-35.0 to -16.2)	0.65 (0.57 to 0.74)	45.5	71.0 (59.0 to 85.4)	-25.5 (-39.5 to -13.5)	0.64 (0.53 to 0.77)
Singapore	COPD	Male	187	276 (242 to 315)	-88.6 (-128 to -54.1)	0.68 (0.60 to 0.78)	160	276 (229 to 333)	-116 (-172 to -68.0)	0.58 (0.48 to 0.70)
Singapore	Congestive heart failure and hypertension	Total	212	227 (202 to 254)	-14.7 (-42.5 to 10.2)	0.94 (0.83 to 1.05)	221	214 (183 to 252)	6.42 (-30.7 to 38.0)	1.03 (0.88 to 1.21)
Singapore	Congestive heart failure and hypertension	Female	176	194 (171 to 219)	-17.5 (-43.4 to 5.76)	0.91 (0.80 to 1.03)	183	183 (153 to 218)	-0.090 (-35.3 to 29.1)	1.00 (0.84 to 1.19)
Singapore	Congestive heart failure and hypertension	Male	254	262 (233 to 295)	-8.06 (-40.8 to 21.4)	0.97 (0.86 to 1.09)	265	248 (210 to 293)	16.7 (-28.1 to 55.0)	1.07 (0.90 to 1.26)
Singapore	Congestive heart failure	Total	173	184 (162 to 209)	-11.7 (-36.4 to 10.2)	0.94 (0.83 to 1.06)	184	173 (145 to 207)	10.7 (-23.3 to 38.8)	1.06 (0.89 to 1.27)
Singapore	Congestive heart failure	Female	136	150 (133 to 169)	-13.4 (-32.8 to 3.69)	0.91 (0.81 to 1.03)	145	141 (119 to 166)	4.78 (-21.5 to 26.6)	1.03 (0.87 to 1.22)
Singapore	Congestive heart failure	Male	216	222 (193 to 256)	-6.17 (-39.3 to 23.1)	0.97 (0.85 to 1.12)	229	209 (171 to 255)	20.6 (-26.6 to 58.3)	1.10 (0.90 to 1.34)

Singapore	Diabetes	Total	395	441 (324 to 602)	-46.6 (-210 to 70.3)	0.89 (0.65 to 1.22)	411	441 (285 to 684)	-30.0 (-276 to 129)	0.93 (0.60 to 1.46)
Singapore	Diabetes	Female	342	390 (279 to 545)	-47.3 (-201 to 62.3)	0.88 (0.63 to 1.22)	353	390 (242 to 626)	-36.2 (-267 to 112)	0.91 (0.57 to 1.46)
Singapore	Diabetes	Male	453	496 (372 to 663)	-43.4 (-210 to 81.3)	0.91 (0.68 to 1.22)	474	496 (330 to 748)	-22.3 (-272 to 148)	0.96 (0.64 to 1.45)
Singapore	Hypertension	Total	39.3	43.6 (37.8 to 50.3)	-4.30 (-11.0 to 1.42)	0.90 (0.78 to 1.04)	36.8	43.6 (35.6 to 53.3)	-6.80 (-16.1 to 1.21)	0.84 (0.70 to 1.03)
Singapore	Hypertension	Female	39.7	45.7 (39.1 to 53.5)	-6.00 (-14.0 to 0.787)	0.87 (0.74 to 1.02)	37.3	45.7 (36.6 to 57.1)	-8.40 (-19.5 to 0.764)	0.82 (0.66 to 1.02)
Singapore	Hypertension	Male	38.3	40.7 (34.2 to 48.5)	-2.40 (-10.1 to 4.06)	0.94 (0.79 to 1.12)	35.6	40.7 (31.8 to 52.1)	-5.10 (-16.6 to 3.79)	0.87 (0.68 to 1.12)
Slovak Republic	Asthma and COPD	Total	121	178 (157 to 200)	-56.4 (-79.2 to -36.2)	0.68 (0.60 to 0.77)	80.9	163 (138 to 194)	-82.4 (-113 to -56.7)	0.50 (0.42 to 0.59)
Slovak Republic	Asthma and COPD	Female	101	161 (138 to 188)	-60.1 (-87.1 to -36.8)	0.63 (0.54 to 0.73)	72.2	148 (119 to 183)	-75.3 (-110 to -46.3)	0.49 (0.40 to 0.61)
Slovak Republic	Asthma and COPD	Male	150	203 (184 to 225)	-52.9 (-74.3 to -34.1)	0.74 (0.67 to 0.82)	94.3	188 (163 to 216)	-93.2 (-121 to -69.1)	0.50 (0.44 to 0.58)
Slovak Republic	Asthma	Total	47.4	68.8 (57.0 to 83.0)	-21.4 (-35.3 to -9.45)	0.69 (0.57 to 0.83)	32.5	62.4 (47.8 to 81.3)	-29.9 (-48.9 to -15.3)	0.52 (0.40 to 0.68)
Slovak Republic	Asthma	Female	55.7	86.9 (71.1 to 106)	-31.2 (-50.5 to -15.6)	0.64 (0.52 to 0.78)	39.8	78.4 (59.0 to 104)	-38.6 (-64.7 to -19.7)	0.51 (0.38 to 0.67)
Slovak Republic	Asthma	Male	38.4	48.0 (40.6 to 56.9)	-9.64 (-18.5 to -2.25)	0.80 (0.68 to 0.94)	24.8	43.9 (34.6 to 55.7)	-19.1 (-30.8 to -9.73)	0.57 (0.45 to 0.72)
Slovak Republic	COPD	Total	73.8	109 (97.7 to 121)	-35.1 (-47.6 to -23.7)	0.68 (0.61 to 0.76)	48.4	101 (86.7 to 118)	-52.8 (-69.6 to -38.3)	0.48 (0.41 to 0.56)
Slovak Republic	COPD	Female	45.3	74.6 (65.2 to 85.4)	-29.3 (-40.2 to -19.9)	0.61 (0.53 to 0.70)	32.4	69.7 (57.6 to 84.3)	-37.3 (-52.0 to -25.3)	0.46 (0.38 to 0.56)
Slovak Republic	COPD	Male	112	155 (140 to 172)	-43.3 (-60.1 to -27.8)	0.72 (0.65 to 0.80)	69.4	144 (124 to 167)	-74.3 (-97.7 to -54.5)	0.48 (0.42 to 0.56)
Slovak Republic	Congestive heart failure and hypertension	Total	581	762 (694 to 836)	-181 (-257 to -114)	0.76 (0.69 to 0.84)	466	755 (684 to 834)	-289 (-368 to -219)	0.62 (0.56 to 0.68)
Slovak Republic	Congestive heart failure and hypertension	Female	513	681 (600 to 774)	-169 (-257 to -86.5)	0.75 (0.67 to 0.86)	402	681 (569 to 816)	-279 (-414 to -168)	0.59 (0.49 to 0.71)
Slovak Republic	Congestive heart failure and hypertension	Male	659	823 (759 to 892)	-164 (-231 to -99.8)	0.80 (0.74 to 0.87)	538	823 (759 to 892)	-285 (-354 to -222)	0.65 (0.60 to 0.71)
Slovak Republic	Congestive heart failure	Total	426	526 (445 to 623)	-100 (-198 to -19.7)	0.81 (0.68 to 0.96)	364	526 (415 to 668)	-162 (-300 to -51.6)	0.69 (0.55 to 0.88)
Slovak Republic	Congestive heart failure	Female	338	393 (345 to 447)	-55.1 (-109 to -7.78)	0.86 (0.76 to 0.98)	290	393 (345 to 447)	-103 (-155 to -54.1)	0.74 (0.65 to 0.84)
Slovak Republic	Congestive heart failure	Male	535	659 (546 to 795)	-124 (-259 to -10.5)	0.81 (0.67 to 0.98)	453	659 (505 to 859)	-206 (-408 to -53.5)	0.69 (0.53 to 0.89)
Slovak Republic	Diabetes	Total	188	223 (207 to 239)	-34.6 (-51.0 to -19.2)	0.84 (0.79 to 0.91)	170	223 (207 to 239)	-53.1 (-69.7 to -37.8)	0.76 (0.71 to 0.82)
Slovak Republic	Diabetes	Female	156	187 (173 to 202)	-31.2 (-46.8 to -17.0)	0.83 (0.77 to 0.90)	141	187 (167 to 209)	-45.6 (-67.7 to -25.6)	0.76 (0.68 to 0.85)
Slovak Republic	Diabetes	Male	224	242 (223 to 263)	-18.1 (-38.8 to 2.06)	0.93 (0.85 to 1.01)	201	242 (223 to 263)	-41.3 (-61.6 to -21.1)	0.83 (0.77 to 0.91)

Slovak Republic	Hypertension	Total	155	190 (167 to 216)	-34.6 (-61.5 to -11.9)	0.82 (0.72 to 0.93)	102	166 (135 to 204)	-64.2 (-103 to -33.4)	0.61 (0.50 to 0.75)
Slovak Republic	Hypertension	Female	175	218 (191 to 250)	-43.5 (-74.1 to -16.1)	0.80 (0.70 to 0.92)	112	189 (152 to 234)	-77.3 (-123 to -40.2)	0.59 (0.48 to 0.74)
Slovak Republic	Hypertension	Male	124	147 (126 to 171)	-22.9 (-46.9 to -2.10)	0.84 (0.73 to 0.98)	84.7	131 (103 to 168)	-46.5 (-83.4 to -17.8)	0.65 (0.50 to 0.83)
Slovenia	Asthma and COPD	Total	75	123 (115 to 133)	-48.4 (-57.6 to -39.5)	0.61 (0.57 to 0.65)	75.8	120 (108 to 133)	-44.2 (-57.2 to -32.4)	0.63 (0.57 to 0.70)
Slovenia	Asthma and COPD	Female	57.5	104 (94.3 to 115)	-46.6 (-57.4 to -36.8)	0.55 (0.50 to 0.61)	62.1	104 (90.5 to 120)	-42.0 (-57.5 to -28.3)	0.60 (0.52 to 0.69)
Slovenia	Asthma and COPD	Male	99.1	153 (140 to 166)	-53.7 (-66.9 to -41.2)	0.65 (0.60 to 0.71)	94.5	147 (130 to 166)	-52.2 (-71.2 to -35.7)	0.64 (0.57 to 0.73)
Slovenia	Asthma	Total	17.4	30.0 (24.7 to 36.4)	-12.6 (-19.0 to -7.32)	0.58 (0.48 to 0.70)	15.2	30.0 (22.8 to 39.5)	-14.8 (-24.3 to -7.67)	0.51 (0.38 to 0.66)
Slovenia	Asthma	Female	19.8	33.9 (27.6 to 41.6)	-14.1 (-21.7 to -7.81)	0.58 (0.48 to 0.72)	14.6	33.9 (25.3 to 45.3)	-19.3 (-30.5 to -10.8)	0.43 (0.32 to 0.58)
Slovenia	Asthma	Male	14.4	25.8 (20.3 to 32.8)	-11.4 (-18.2 to -5.91)	0.56 (0.44 to 0.71)	16	25.8 (18.4 to 36.2)	-9.80 (-20.3 to -2.45)	0.62 (0.44 to 0.87)
Slovenia	COPD	Total	57.6	94.7 (91.0 to 98.6)	-37.1 (-41.0 to -33.4)	0.61 (0.58 to 0.63)	60.6	92.6 (87.5 to 98.1)	-32.0 (-37.4 to -26.9)	0.65 (0.62 to 0.69)
Slovenia	COPD	Female	37.7	70.2 (65.6 to 75.2)	-32.5 (-37.5 to -27.8)	0.54 (0.50 to 0.58)	47.5	70.2 (63.7 to 77.3)	-22.7 (-29.8 to -16.3)	0.68 (0.61 to 0.74)
Slovenia	COPD	Male	84.7	128 (121 to 136)	-43.3 (-51.1 to -35.9)	0.66 (0.62 to 0.70)	78.5	123 (113 to 134)	-44.5 (-55.2 to -34.9)	0.64 (0.59 to 0.69)
Slovenia	Congestive heart failure and hypertension	Total	227	275 (252 to 301)	-48.7 (-74.5 to -25.7)	0.82 (0.75 to 0.90)	229	267 (236 to 302)	-38.1 (-72.6 to -7.21)	0.86 (0.76 to 0.97)
Slovenia	Congestive heart failure and hypertension	Female	186	238 (209 to 272)	-52.0 (-86.3 to -22.8)	0.78 (0.68 to 0.89)	195	238 (198 to 287)	-43.7 (-92.5 to -2.78)	0.82 (0.68 to 0.99)
Slovenia	Congestive heart failure and hypertension	Male	276	339 (302 to 382)	-63.0 (-106 to -25.4)	0.81 (0.72 to 0.92)	267	339 (287 to 401)	-72.0 (-134 to -18.8)	0.79 (0.67 to 0.93)
Slovenia	Congestive heart failure	Total	197	239 (220 to 261)	-42.0 (-64.1 to -22.4)	0.82 (0.75 to 0.90)	202	239 (212 to 270)	-36.9 (-68.3 to -10.1)	0.85 (0.75 to 0.95)
Slovenia	Congestive heart failure	Female	158	194 (172 to 219)	-35.7 (-60.0 to -14.0)	0.82 (0.73 to 0.92)	170	194 (164 to 230)	-23.8 (-58.9 to 6.00)	0.88 (0.74 to 1.04)
Slovenia	Congestive heart failure	Male	247	295 (266 to 328)	-48.3 (-81.1 to -18.7)	0.84 (0.75 to 0.93)	240	295 (255 to 342)	-55.5 (-101 to -15.4)	0.81 (0.70 to 0.94)
Slovenia	Diabetes	Total	90.7	108 (94.7 to 122)	-17.0 (-31.6 to -4.21)	0.84 (0.74 to 0.96)	88.4	108 (94.7 to 122)	-19.3 (-34.2 to -6.53)	0.82 (0.72 to 0.93)
Slovenia	Diabetes	Female	56.4	75.0 (62.6 to 89.8)	-18.6 (-33.3 to -5.99)	0.75 (0.63 to 0.90)	53.8	75.0 (58.1 to 96.8)	-21.2 (-43.1 to -4.52)	0.72 (0.56 to 0.92)
Slovenia	Diabetes	Male	131	137 (115 to 163)	-5.52 (-31.4 to 15.9)	0.96 (0.81 to 1.14)	129	137 (115 to 163)	-7.42 (-33.7 to 14.3)	0.95 (0.79 to 1.12)
Slovenia	Hypertension	Total	29.3	41.2 (36.1 to 47.2)	-11.9 (-17.8 to -6.86)	0.71 (0.62 to 0.81)	26.3	37.9 (31.4 to 45.8)	-11.6 (-19.3 to -4.98)	0.69 (0.58 to 0.84)
Slovenia	Hypertension	Female	27.9	40.5 (34.4 to 47.6)	-12.6 (-19.6 to -6.54)	0.69 (0.59 to 0.81)	24.2	37.0 (29.5 to 46.5)	-12.8 (-22.5 to -5.13)	0.65 (0.52 to 0.83)
Slovenia	Hypertension	Male	29.3	40.5 (34.6 to 47.3)	-11.2 (-18.1 to -5.38)	0.72 (0.62 to 0.84)	27.5	37.3 (29.9 to 46.5)	-9.82 (-18.7 to -2.57)	0.74 (0.59 to 0.91)

Sweden	Asthma	Total	11.6	13.7 (12.7 to 14.8)	-2.12 (-3.19 to -1.12)	0.85 (0.78 to 0.91)	10.9	12.9 (11.6 to 14.3)	-2.00 (-3.47 to -0.711)	0.85 (0.76 to 0.94)
Sweden	Asthma	Female	14.6	18.5 (17.0 to 20.1)	-3.89 (-5.46 to -2.43)	0.79 (0.73 to 0.86)	13.8	17.4 (15.5 to 19.6)	-3.63 (-5.69 to -1.73)	0.79 (0.71 to 0.89)
Sweden	Asthma	Male	8	8.30 (7.28 to 9.47)	-0.304 (-1.48 to 0.728)	0.96 (0.84 to 1.10)	7.5	7.75 (6.43 to 9.33)	-0.249 (-1.84 to 1.08)	0.97 (0.80 to 1.17)
Sweden	COPD	Total	91.2	104 (91.4 to 119)	-13.0 (-27.4 to 0.077)	0.88 (0.77 to 1.00)	94.6	86.1 (64.2 to 115)	8.49 (-21.2 to 30.3)	1.10 (0.82 to 1.47)
Sweden	COPD	Female	100	114 (101 to 128)	-13.4 (-27.8 to -0.556)	0.88 (0.78 to 0.99)	107	92.5 (70.8 to 121)	14.6 (-13.6 to 36.0)	1.16 (0.89 to 1.51)
Sweden	COPD	Male	82	105 (91.8 to 119)	-22.7 (-37.9 to -9.72)	0.78 (0.68 to 0.89)	80.1	98.6 (81.9 to 119)	-18.5 (-38.6 to -1.75)	0.81 (0.68 to 0.98)
Sweden	Congestive heart failure	Total	178	200 (184 to 218)	-21.6 (-39.3 to -5.65)	0.89 (0.82 to 0.97)	196	192 (170 to 217)	4.08 (-20.7 to 26.1)	1.02 (0.90 to 1.15)
Sweden	Congestive heart failure	Female	136	152 (138 to 168)	-17.0 (-32.6 to -3.16)	0.89 (0.81 to 0.98)	154	147 (128 to 168)	7.46 (-14.1 to 26.3)	1.05 (0.92 to 1.21)
Sweden	Congestive heart failure	Male	241	241 (224 to 260)	0.280 (-19.0 to 17.9)	1.00 (0.93 to 1.08)	258	208 (176 to 247)	49.3 (10.8 to 81.8)	1.24 (1.04 to 1.47)
Sweden	Diabetes	Total	57.4	59.5 (53.2 to 66.5)	-2.07 (-9.21 to 4.18)	0.97 (0.86 to 1.08)	59.1	54.5 (46.5 to 63.8)	4.61 (-4.61 to 12.5)	1.08 (0.93 to 1.27)
Sweden	Diabetes	Female	43.1	44.4 (39.1 to 50.3)	-1.26 (-7.06 to 3.89)	0.97 (0.86 to 1.10)	44	40.5 (33.9 to 48.3)	3.52 (-4.37 to 10.0)	1.09 (0.91 to 1.30)
Sweden	Diabetes	Male	73.8	77.6 (68.7 to 87.7)	-3.77 (-13.7 to 4.80)	0.95 (0.84 to 1.07)	76.5	71.3 (60.0 to 84.7)	5.20 (-8.27 to 16.6)	1.07 (0.90 to 1.28)
Sweden	Hypertension	Total	22.9	20.6 (19.0 to 22.3)	2.32 (0.609 to 3.96)	1.11 (1.03 to 1.21)	20.8	18.0 (16.1 to 20.2)	2.78 (0.637 to 4.74)	1.15 (1.03 to 1.29)
Sweden	Hypertension	Female	26.2	21.6 (20.0 to 23.2)	4.61 (2.95 to 6.15)	1.21 (1.13 to 1.31)	21.8	18.9 (17.1 to 21.0)	2.86 (0.789 to 4.75)	1.15 (1.04 to 1.28)
Sweden	Hypertension	Male	17.5	18.0 (15.7 to 20.7)	-0.517 (-3.21 to 1.74)	0.97 (0.85 to 1.11)	18.4	15.7 (12.9 to 19.1)	2.72 (-0.675 to 5.49)	1.17 (0.96 to 1.43)

Table S7. Expected and observed surgical procedures by country, 2020-2021

Location	Year	Surgery	Observed surgeries	Observed rate	Expected surgeries	Expected rate	Difference surgeries	Difference rate	Observed-to-expected ratio
OECD	2020	All surgeries	13,300,000	23.3	16,100,000 (15,800,000 to 16,400,000)	28.2 (27.6 to 28.7)	-2,780,000 (-3,080,000 to -2,490,000)	-4.87 (-5.39 to -4.36)	0.83 (0.81 to 0.84)
OECD	2021	All surgeries	14,800,000	26	16,100,000 (15,600,000 to 16,500,000)	28.3 (27.6 to 29.1)	-1,310,000 (-1,760,000 to -884,000)	-2.32 (-3.10 to -1.56)	0.92 (0.89 to 0.94)
OECD	2020	Non-elective surgeries	2,100,000	3.68	2,380,000 (2,330,000 to 2,440,000)	4.17 (4.07 to 4.26)	-279,000 (-333,000 to -227,000)	-0.489 (-0.583 to -0.398)	0.88 (0.86 to 0.90)
OECD	2021	Non-elective surgeries	2,270,000	4.01	2,370,000 (2,310,000 to 2,420,000)	4.17 (4.08 to 4.27)	-91,700 (-146,000 to -39,900)	-0.162 (-0.257 to -0.070)	0.96 (0.94 to 0.98)
OECD	2020	Appendectomy	598,000	1.06	627,000 (620,000 to 634,000)	1.11 (1.10 to 1.12)	-29,600 (-36,800 to -22,600)	-0.052 (-0.065 to -0.040)	0.95 (0.94 to 0.96)
OECD	2021	Appendectomy	616,000	1.1	613,000 (603,000 to 622,000)	1.09 (1.07 to 1.11)	3,140 (-6,500 to 12,800)	0.006 (-0.012 to 0.023)	1.01 (0.99 to 1.02)
OECD	2020	Cholecystectomy	907,000	1.59	1,060,000 (1,040,000 to 1,090,000)	1.86 (1.82 to 1.91)	-157,000 (-182,000 to -133,000)	-0.275 (-0.319 to -0.232)	0.85 (0.83 to 0.87)
OECD	2021	Cholecystectomy	992,000	1.75	1,060,000 (1,030,000 to 1,080,000)	1.87 (1.82 to 1.91)	-67,100 (-92,500 to -42,600)	-0.118 (-0.163 to -0.075)	0.94 (0.91 to 0.96)
OECD	2020	Total mastectomy	170,000	0.302	188,000 (180,000 to 198,000)	0.336 (0.320 to 0.353)	-19,000 (-28,500 to -9,990)	-0.034 (-0.051 to -0.018)	0.90 (0.86 to 0.94)
OECD	2021	Total mastectomy	183,000	0.329	187,000 (175,000 to 201,000)	0.336 (0.314 to 0.360)	-4,230 (-17,400 to 8,460)	-0.008 (-0.031 to 0.015)	0.98 (0.91 to 1.05)
OECD	2020	Partial excision of mammary gland	427,000	0.769	470,000 (444,000 to 497,000)	0.846 (0.800 to 0.896)	-43,000 (-70,200 to -17,300)	-0.077 (-0.126 to -0.031)	0.91 (0.86 to 0.96)
OECD	2021	Partial excision of mammary gland	483,000	0.877	468,000 (442,000 to 495,000)	0.849 (0.802 to 0.899)	15,500 (-11,900 to 41,700)	0.028 (-0.022 to 0.076)	1.03 (0.98 to 1.09)
OECD	2020	Both	3,610,000	6.32	4,030,000 (3,950,000 to 4,120,000)	7.06 (6.91 to 7.20)	-422,000 (-507,000 to -339,000)	-0.739 (-0.887 to -0.594)	0.90 (0.88 to 0.91)
OECD	2021	Both	3,570,000	6.3	3,790,000 (3,680,000 to 3,920,000)	6.69 (6.48 to 6.91)	-222,000 (-343,000 to -103,000)	-0.391 (-0.604 to -0.181)	0.94 (0.91 to 0.97)
OECD	2020	Caesarean section	1,480,000	2.64	1,500,000 (1,450,000 to 1,560,000)	2.68 (2.59 to 2.77)	-25,100 (-76,800 to 25,600)	-0.045 (-0.137 to 0.046)	0.98 (0.95 to 1.02)
OECD	2021	Caesarean section	1,270,000	2.6	1,120,000 (1,060,000 to 1,190,000)	2.29 (2.17 to 2.43)	149,000 (82,800 to 211,000)	0.305 (0.169 to 0.432)	1.13 (1.07 to 1.20)

OECD	2020	Repair of inguinal hernia	783,000	1.37	1,020,000 (999,000 to 1,040,000)	1.78 (1.75 to 1.82)	-235,000 (-255,000 to -216,000)	-0.412 (-0.446 to - 0.378)	0.77 (0.75 to 0.78)
OECD	2021	Repair of inguinal hernia	869,000	1.53	1,010,000 (988,000 to 1,040,000)	1.79 (1.74 to 1.84)	-146,000 (-173,000 to -119,000)	-0.257 (-0.306 to - 0.211)	0.86 (0.83 to 0.88)
OECD	2020	Transluminal coronary angioplasty	1,190,000	2.1	1,350,000 (1,300,000 to 1,400,000)	2.38 (2.30 to 2.47)	-158,000 (-205,000 to -113,000)	-0.280 (-0.362 to - 0.200)	0.88 (0.85 to 0.91)
OECD	2021	Transluminal coronary angioplasty	1,270,000	2.26	1,380,000 (1,310,000 to 1,450,000)	2.45 (2.33 to 2.57)	-106,000 (-176,000 to -41,000)	-0.189 (-0.314 to - 0.073)	0.92 (0.88 to 0.97)
OECD	2020	Coronary artery bypass graft	157,000	0.275	189,000 (183,000 to 195,000)	0.331 (0.321 to 0.341)	-31,600 (-37,400 to -25,900)	-0.055 (-0.066 to - 0.045)	0.83 (0.81 to 0.86)
OECD	2021	Coronary artery bypass graft	164,000	0.289	185,000 (177,000 to 193,000)	0.326 (0.312 to 0.340)	-20,800 (-28,900 to -13,000)	-0.037 (-0.051 to - 0.023)	0.89 (0.85 to 0.93)
OECD	2020	Elective surgeries	7,600,000	13.3	9,670,000 (9,430,000 to 9,920,000)	16.9 (16.5 to 17.4)	-2,070,000 (- 2,330,000 to - 1,830,000)	-3.63 (-4.07 to - 3.20)	0.79 (0.77 to 0.81)
OECD	2021	Elective surgeries	8,910,000	15.7	9,880,000 (9,530,000 to 10,200,000)	17.4 (16.8 to 18.1)	-975,000 (- 1,330,000 to - 613,000)	-1.72 (-2.34 to - 1.08)	0.90 (0.87 to 0.94)
OECD	2020	Hip replacement	957,000	1.67	1,160,000 (1,130,000 to 1,190,000)	2.02 (1.97 to 2.08)	-200,000 (-231,000 to -170,000)	-0.350 (-0.405 to - 0.297)	0.83 (0.81 to 0.85)
OECD	2021	Hip replacement	1,090,000	1.93	1,180,000 (1,130,000 to 1,220,000)	2.08 (2.00 to 2.15)	-84,900 (-129,000 to -41,300)	-0.150 (-0.227 to - 0.073)	0.93 (0.89 to 0.96)
OECD	2020	Total knee replacement	692,000	1.21	938,000 (896,000 to 981,000)	1.64 (1.57 to 1.72)	-246,000 (-289,000 to -205,000)	-0.430 (-0.506 to - 0.359)	0.74 (0.71 to 0.77)
OECD	2021	Total knee replacement	789,000	1.39	961,000 (901,000 to 1,020,000)	1.70 (1.59 to 1.81)	-172,000 (-236,000 to -112,000)	-0.304 (-0.417 to - 0.198)	0.82 (0.77 to 0.88)
OECD	2020	Cataract surgery	4,790,000	8.38	6,100,000 (5,820,000 to 6,390,000)	10.7 (10.2 to 11.2)	-1,310,000 (- 1,600,000 to - 1,040,000)	-2.30 (-2.80 to - 1.82)	0.78 (0.75 to 0.82)
OECD	2021	Cataract surgery	5,790,000	10.2	6,310,000 (5,920,000 to 6,740,000)	11.1 (10.4 to 11.9)	-520,000 (-943,000 to -128,000)	-0.917 (-1.66 to - 0.226)	0.92 (0.86 to 0.98)
OECD	2020	Tonsillectomy	298,000	0.526	458,000 (435,000 to 483,000)	0.810 (0.769 to 0.854)	-161,000 (-185,000 to -138,000)	-0.284 (-0.328 to - 0.244)	0.65 (0.62 to 0.68)
OECD	2021	Tonsillectomy	282,000	0.503	440,000 (409,000 to 475,000)	0.785 (0.728 to 0.846)	-158,000 (-193,000 to -126,000)	-0.281 (-0.343 to - 0.225)	0.64 (0.59 to 0.69)
OECD	2020	Hysterectomy	438,000	0.774	517,000 (509,000 to 525,000)	0.914 (0.900 to 0.928)	-79,300 (-87,400 to -71,200)	-0.140 (-0.154 to - 0.126)	0.85 (0.83 to 0.86)
OECD	2021	Hysterectomy	476,000	0.849	503,000 (495,000 to 511,000)	0.896 (0.882 to 0.910)	-26,400 (-34,600 to -18,400)	-0.047 (-0.062 to - 0.033)	0.95 (0.93 to 0.96)

OECD	2020	Open prostatectomy	141,000	0.246	158,000 (140,000 to 178,000)	0.276 (0.245 to 0.311)	-17,100 (-36,600 to 615)	-0.030 (-0.064 to 0.001)	0.89 (0.79 to 1.00)
OECD	2021	Open prostatectomy	149,000	0.262	156,000 (132,000 to 183,000)	0.274 (0.233 to 0.323)	-6,750 (-34,100 to 16,400)	-0.012 (-0.060 to 0.029)	0.96 (0.81 to 1.12)
OECD	2020	Transurethral prostatectomy	285,000	0.504	355,000 (345,000 to 365,000)	0.628 (0.611 to 0.645)	-70,200 (-79,700 to -60,600)	-0.124 (-0.141 to -0.107)	0.80 (0.78 to 0.82)
OECD	2021	Transurethral prostatectomy	324,000	0.572	360,000 (346,000 to 374,000)	0.635 (0.611 to 0.660)	-35,900 (-50,200 to -22,300)	-0.063 (-0.089 to -0.039)	0.90 (0.87 to 0.94)
Austria	2020	All surgeries	281,000	31.5	329,000 (323,000 to 335,000)	36.9 (36.2 to 37.6)	-47,500 (-53,700 to -41,300)	-5.32 (-6.02 to -4.64)	0.86 (0.84 to 0.87)
Austria	2021	All surgeries	309,000	34.5	335,000 (326,000 to 344,000)	37.4 (36.4 to 38.4)	-25,700 (-34,400 to -17,000)	-2.87 (-3.84 to -1.90)	0.92 (0.90 to 0.95)
Austria	2020	Non-elective surgeries	39,100	4.38	43,600 (42,100 to 45,200)	4.89 (4.72 to 5.06)	-4,500 (-6,050 to -3,020)	-0.504 (-0.678 to -0.338)	0.90 (0.87 to 0.93)
Austria	2021	Non-elective surgeries	41,200	4.6	43,800 (41,700 to 46,000)	4.89 (4.65 to 5.14)	-2,620 (-4,780 to -486)	-0.292 (-0.534 to -0.054)	0.94 (0.90 to 0.99)
Austria	2020	Appendectomy	11,700	1.31	13,100 (12,300 to 13,900)	1.47 (1.37 to 1.56)	-1,360 (-2,250 to -527)	-0.152 (-0.252 to -0.059)	0.90 (0.84 to 0.96)
Austria	2021	Appendectomy	12,200	1.36	13,100 (12,000 to 14,400)	1.47 (1.34 to 1.61)	-951 (-2,200 to 187)	-0.106 (-0.245 to 0.021)	0.93 (0.85 to 1.02)
Austria	2020	Cholecystectomy	16,500	1.85	18,600 (17,900 to 19,300)	2.09 (2.01 to 2.16)	-2,090 (-2,780 to -1,420)	-0.235 (-0.312 to -0.159)	0.89 (0.86 to 0.92)
Austria	2021	Cholecystectomy	17,200	1.92	18,700 (17,700 to 19,700)	2.09 (1.98 to 2.20)	-1,520 (-2,520 to -570)	-0.170 (-0.281 to -0.064)	0.92 (0.87 to 0.97)
Austria	2020	Total mastectomy	2,680	0.3	3,030 (2,840 to 3,230)	0.340 (0.319 to 0.362)	-353 (-549 to -166)	-0.040 (-0.062 to -0.019)	0.88 (0.83 to 0.94)
Austria	2021	Total mastectomy	2,880	0.321	3,140 (2,870 to 3,430)	0.350 (0.320 to 0.383)	-259 (-554 to 17)	-0.029 (-0.062 to 0.002)	0.92 (0.84 to 1.01)
Austria	2020	Partial excision of mammary gland	8,200	0.919	8,980 (8,420 to 9,580)	1.01 (0.945 to 1.07)	-786 (-1,380 to -252)	-0.088 (-0.155 to -0.028)	0.91 (0.86 to 0.97)
Austria	2021	Partial excision of mammary gland	8,940	0.999	9,020 (8,240 to 9,880)	1.01 (0.920 to 1.10)	-77 (-932 to 710)	-0.009 (-0.104 to 0.079)	0.99 (0.91 to 1.09)
Austria	2020	Both	76,200	8.54	84,500 (82,100 to 86,900)	9.48 (9.21 to 9.75)	-8,300 (-10,700 to -5,980)	-0.931 (-1.20 to -0.670)	0.90 (0.88 to 0.93)
Austria	2021	Both	81,000	9.04	86,100 (82,700 to 89,600)	9.61 (9.24 to 10.0)	-5,140 (-8,560 to -1,700)	-0.574 (-0.955 to -0.190)	0.94 (0.90 to 0.98)
Austria	2020	Caesarean section	24,800	2.78	25,300 (24,100 to 26,600)	2.84 (2.70 to 2.99)	-509 (-1,810 to 705)	-0.057 (-0.203 to 0.079)	0.98 (0.93 to 1.03)
Austria	2021	Caesarean section	26,200	2.92	25,400 (23,700 to 27,300)	2.84 (2.65 to 3.05)	734 (-1,110 to 2,470)	0.082 (-0.124 to 0.276)	1.03 (0.96 to 1.10)
Austria	2020	Repair of inguinal hernia	21,200	2.38	25,700 (24,700 to 26,700)	2.88 (2.77 to 3.00)	-4,460 (-5,480 to -3,480)	-0.500 (-0.614 to -0.390)	0.83 (0.79 to 0.86)
Austria	2021	Repair of inguinal hernia	22,700	2.53	25,800 (24,400 to 27,300)	2.88 (2.72 to 3.05)	-3,090 (-4,580 to -1,660)	-0.345 (-0.511 to -0.185)	0.88 (0.83 to 0.93)

Austria	2020	Transluminal coronary angioplasty	27,300	3.06	30,000 (28,100 to 32,100)	3.37 (3.15 to 3.60)	-2,740 (-4,790 to -783)	-0.307 (-0.537 to -0.088)	0.91 (0.85 to 0.97)
Austria	2021	Transluminal coronary angioplasty	29,000	3.24	31,200 (28,400 to 34,300)	3.48 (3.17 to 3.83)	-2,180 (-5,240 to 654)	-0.243 (-0.585 to 0.073)	0.93 (0.85 to 1.02)
Austria	2020	Coronary artery bypass graft	2,870	0.322	3,240 (2,900 to 3,620)	0.363 (0.325 to 0.405)	-366 (-745 to -29)	-0.041 (-0.083 to -0.003)	0.89 (0.79 to 0.99)
Austria	2021	Coronary artery bypass graft	3,080	0.344	3,250 (2,780 to 3,800)	0.363 (0.311 to 0.424)	-173 (-717 to 299)	-0.019 (-0.080 to 0.033)	0.95 (0.81 to 1.11)
Austria	2020	Elective surgeries	166,000	18.6	201,000 (196,000 to 206,000)	22.6 (22.0 to 23.2)	-35,300 (-40,500 to -30,200)	-3.95 (-4.55 to -3.39)	0.82 (0.80 to 0.85)
Austria	2021	Elective surgeries	187,000	20.9	206,000 (199,000 to 214,000)	23.0 (22.2 to 23.9)	-19,200 (-26,900 to -11,800)	-2.14 (-3.00 to -1.32)	0.91 (0.87 to 0.94)
Austria	2020	Hip replacement	23,500	2.63	26,300 (25,000 to 27,600)	2.95 (2.81 to 3.10)	-2,820 (-4,120 to -1,580)	-0.316 (-0.462 to -0.177)	0.89 (0.85 to 0.94)
Austria	2021	Hip replacement	25,700	2.87	26,400 (24,600 to 28,300)	2.95 (2.75 to 3.16)	-679 (-2,570 to 1,060)	-0.076 (-0.287 to 0.118)	0.97 (0.91 to 1.04)
Austria	2020	Total knee replacement	16,100	1.81	20,400 (19,200 to 21,700)	2.29 (2.15 to 2.43)	-4,270 (-5,540 to -3,080)	-0.479 (-0.622 to -0.345)	0.79 (0.74 to 0.84)
Austria	2021	Total knee replacement	17,500	1.95	20,500 (18,800 to 22,300)	2.29 (2.10 to 2.49)	-2,980 (-4,790 to -1,260)	-0.332 (-0.535 to -0.140)	0.85 (0.78 to 0.93)
Austria	2020	Cataract surgery	104,000	11.6	128,000 (124,000 to 132,000)	14.3 (13.9 to 14.8)	-24,000 (-28,300 to -19,800)	-2.69 (-3.17 to -2.22)	0.81 (0.79 to 0.84)
Austria	2021	Cataract surgery	120,000	13.4	133,000 (127,000 to 139,000)	14.8 (14.2 to 15.5)	-12,900 (-19,100 to -6,780)	-1.44 (-2.13 to -0.757)	0.90 (0.86 to 0.95)
Austria	2020	Tonsillectomy	4,540	0.509	6,530 (6,110 to 6,980)	0.732 (0.685 to 0.783)	-1,990 (-2,460 to -1,570)	-0.224 (-0.276 to -0.176)	0.69 (0.65 to 0.74)
Austria	2021	Tonsillectomy	4,080	0.456	6,230 (5,660 to 6,850)	0.695 (0.633 to 0.765)	-2,140 (-2,760 to -1,580)	-0.239 (-0.308 to -0.177)	0.66 (0.60 to 0.72)
Austria	2020	Hysterectomy	8,330	0.934	9,710 (9,090 to 10,400)	1.09 (1.02 to 1.16)	-1,380 (-2,040 to -772)	-0.155 (-0.228 to -0.087)	0.86 (0.80 to 0.92)
Austria	2021	Hysterectomy	9,240	1.03	9,750 (8,890 to 10,700)	1.09 (0.992 to 1.19)	-512 (-1,440 to 360)	-0.057 (-0.161 to 0.040)	0.95 (0.86 to 1.04)
Austria	2020	Open prostatectomy	3,480	0.39	3,360 (3,060 to 3,690)	0.377 (0.343 to 0.414)	121 (-210 to 423)	0.014 (-0.024 to 0.047)	1.04 (0.94 to 1.14)
Austria	2021	Open prostatectomy	3,650	0.407	3,320 (2,950 to 3,730)	0.371 (0.330 to 0.417)	324 (-80 to 690)	0.036 (-0.009 to 0.077)	1.10 (0.98 to 1.23)
Austria	2020	Transurethral prostatectomy	6,270	0.703	6,930 (6,430 to 7,470)	0.777 (0.721 to 0.838)	-661 (-1,190 to -159)	-0.074 (-0.133 to -0.018)	0.90 (0.84 to 0.98)
Austria	2021	Transurethral prostatectomy	6,770	0.756	6,960 (6,460 to 7,500)	0.777 (0.721 to 0.838)	-193 (-743 to 310)	-0.022 (-0.083 to 0.035)	0.97 (0.90 to 1.05)
Belgium	2020	All surgeries	326,000	28.3	408,000 (399,000 to 418,000)	35.4 (34.5 to 36.2)	-81,900 (-91,700 to -72,300)	-7.10 (-7.94 to -6.27)	0.80 (0.78 to 0.82)
Belgium	2021	All surgeries	395,000	34.1	414,000 (400,000 to 428,000)	35.7 (34.5 to 36.9)	-18,700 (-32,700 to -5,140)	-1.62 (-2.82 to -0.444)	0.95 (0.92 to 0.99)
Belgium	2020	Non-elective surgeries	60,000	5.2	67,100 (61,200 to 73,600)	5.82 (5.30 to 6.38)	-7,120 (-13,600 to -1,260)	-0.617 (-1.18 to -0.109)	0.89 (0.82 to 0.98)

Belgium	2021	Non-elective surgeries	68,500	5.91	67,400 (59,100 to 76,800)	5.82 (5.10 to 6.63)	1,130 (-8,330 to 9,380)	0.097 (-0.719 to 0.810)	1.02 (0.89 to 1.16)
Belgium	2020	Appendectomy	16,100	1.4	16,100 (15,500 to 16,700)	1.40 (1.35 to 1.45)	-2 (-611 to 603)	-0.000 (-0.053 to 0.052)	1.00 (0.96 to 1.04)
Belgium	2021	Appendectomy	16,500	1.43	16,200 (15,400 to 17,100)	1.40 (1.32 to 1.47)	333 (-545 to 1,180)	0.029 (-0.047 to 0.101)	1.02 (0.97 to 1.08)
Belgium	2020	Cholecystectomy	22,900	1.98	26,300 (25,000 to 27,700)	2.28 (2.16 to 2.40)	-3,400 (-4,810 to -2,090)	-0.295 (-0.417 to -0.181)	0.87 (0.83 to 0.92)
Belgium	2021	Cholecystectomy	26,400	2.28	26,400 (24,500 to 28,400)	2.28 (2.12 to 2.45)	44 (-1,930 to 1,880)	0.004 (-0.167 to 0.162)	1.00 (0.93 to 1.08)
Belgium	2020	Total mastectomy	6,830	0.592	7,790 (6,190 to 9,790)	0.675 (0.537 to 0.849)	-959 (-3,000 to 651)	-0.083 (-0.260 to 0.056)	0.88 (0.69 to 1.11)
Belgium	2021	Total mastectomy	7,900	0.682	7,820 (5,660 to 10,800)	0.675 (0.488 to 0.933)	82 (-2,930 to 2,220)	0.007 (-0.253 to 0.192)	1.01 (0.73 to 1.39)
Belgium	2020	Partial excision of mammary gland	14,200	1.23	16,900 (13,500 to 21,100)	1.47 (1.17 to 1.83)	-2,750 (-6,930 to 608)	-0.238 (-0.601 to 0.053)	0.84 (0.67 to 1.04)
Belgium	2021	Partial excision of mammary gland	17,700	1.52	17,000 (12,400 to 23,300)	1.47 (1.07 to 2.01)	666 (-5,760 to 5,200)	0.058 (-0.497 to 0.449)	1.04 (0.75 to 1.42)
Belgium	2020	Both	80,300	6.96	87,300 (84,600 to 90,000)	7.56 (7.33 to 7.80)	-6,970 (-9,690 to -4,370)	-0.604 (-0.840 to -0.379)	0.92 (0.89 to 0.95)
Belgium	2021	Both	88,800	7.67	87,600 (85,000 to 90,300)	7.56 (7.33 to 7.80)	1,200 (-1,600 to 3,820)	0.104 (-0.138 to 0.330)	1.01 (0.98 to 1.04)
Belgium	2020	Caesarean section	24,500	2.13	24,600 (24,200 to 25,100)	2.14 (2.10 to 2.17)	-112 (-567 to 320)	-0.010 (-0.049 to 0.028)	1.00 (0.98 to 1.01)
Belgium	2021	Caesarean section	25,900	2.23	24,500 (23,900 to 25,100)	2.11 (2.06 to 2.17)	1,420 (782 to 2,040)	0.122 (0.067 to 0.176)	1.06 (1.03 to 1.09)
Belgium	2020	Repair of inguinal hernia	20,200	1.75	25,600 (25,000 to 26,200)	2.22 (2.16 to 2.27)	-5,340 (-5,980 to -4,710)	-0.463 (-0.518 to -0.408)	0.79 (0.77 to 0.81)
Belgium	2021	Repair of inguinal hernia	23,900	2.07	25,700 (24,800 to 26,600)	2.22 (2.14 to 2.30)	-1,750 (-2,650 to -872)	-0.151 (-0.228 to -0.075)	0.93 (0.90 to 0.96)
Belgium	2020	Transluminal coronary angioplasty	29,700	2.57	32,700 (29,800 to 35,800)	2.83 (2.58 to 3.10)	-3,020 (-6,230 to -163)	-0.262 (-0.540 to -0.014)	0.91 (0.83 to 0.99)
Belgium	2021	Transluminal coronary angioplasty	32,500	2.8	32,800 (28,800 to 37,300)	2.83 (2.49 to 3.22)	-326 (-4,780 to 3,630)	-0.028 (-0.412 to 0.313)	0.99 (0.87 to 1.13)
Belgium	2020	Coronary artery bypass graft	5,860	0.508	6,910 (6,440 to 7,430)	0.599 (0.558 to 0.644)	-1,050 (-1,580 to -577)	-0.091 (-0.137 to -0.050)	0.85 (0.79 to 0.91)
Belgium	2021	Coronary artery bypass graft	6,510	0.562	6,940 (6,270 to 7,680)	0.599 (0.541 to 0.663)	-431 (-1,170 to 241)	-0.037 (-0.101 to 0.021)	0.94 (0.85 to 1.04)
Belgium	2020	Elective surgeries	186,000	16.1	246,000 (242,000 to 249,000)	21.3 (21.0 to 21.6)	-59,900 (-63,500 to -56,300)	-5.19 (-5.50 to -4.88)	0.76 (0.75 to 0.77)
Belgium	2021	Elective surgeries	238,000	20.5	252,000 (248,000 to 256,000)	21.8 (21.4 to 22.1)	-14,600 (-18,300 to -10,900)	-1.26 (-1.58 to -0.942)	0.94 (0.93 to 0.96)
Belgium	2020	Hip replacement	26,500	2.29	33,400 (32,400 to 34,600)	2.90 (2.80 to 3.00)	-6,980 (-8,090 to -5,870)	-0.605 (-0.701 to -0.509)	0.79 (0.77 to 0.82)
Belgium	2021	Hip replacement	31,400	2.71	34,400 (32,800 to 36,000)	2.97 (2.83 to 3.11)	-2,920 (-4,550 to -1,380)	-0.252 (-0.393 to -0.119)	0.92 (0.87 to 0.96)
Belgium	2020	Total knee replacement	15,800	1.37	24,500 (22,600 to 26,500)	2.12 (1.96 to 2.30)	-8,660 (-10,700 to -6,780)	-0.751 (-0.926 to -0.588)	0.65 (0.60 to 0.70)

Belgium	2021	Total knee replacement	19,000	1.64	24,600 (22,000 to 27,500)	2.12 (1.90 to 2.37)	-5,620 (-8,630 to -2,980)	-0.485 (-0.745 to -0.257)	0.77 (0.69 to 0.86)
Belgium	2020	Cataract surgery	109,000	9.43	142,000 (137,000 to 148,000)	12.3 (11.8 to 12.8)	-33,400 (-39,000 to -28,000)	-2.89 (-3.38 to -2.43)	0.77 (0.74 to 0.80)
Belgium	2021	Cataract surgery	143,000	12.4	145,000 (137,000 to 153,000)	12.5 (11.8 to 13.2)	-1,800 (-9,900 to 6,230)	-0.155 (-0.855 to 0.538)	0.99 (0.94 to 1.05)
Belgium	2020	Tonsillectomy	13,400	1.16	23,400 (21,700 to 25,400)	2.03 (1.88 to 2.20)	-10,000 (-11,900 to -8,250)	-0.871 (-1.03 to -0.715)	0.57 (0.53 to 0.62)
Belgium	2021	Tonsillectomy	18,500	1.6	23,500 (21,100 to 26,300)	2.03 (1.82 to 2.27)	-5,030 (-7,780 to -2,590)	-0.434 (-0.671 to -0.224)	0.79 (0.70 to 0.88)
Belgium	2020	Hysterectomy	9,610	0.833	12,300 (12,000 to 12,600)	1.07 (1.04 to 1.10)	-2,730 (-3,040 to -2,420)	-0.237 (-0.263 to -0.209)	0.78 (0.76 to 0.80)
Belgium	2021	Hysterectomy	11,300	0.975	12,100 (11,600 to 12,500)	1.04 (1.01 to 1.08)	-768 (-1,200 to -360)	-0.066 (-0.104 to -0.031)	0.94 (0.90 to 0.97)
Belgium	2020	Open prostatectomy	4,880	0.423	5,160 (4,760 to 5,580)	0.447 (0.413 to 0.484)	-279 (-714 to 119)	-0.024 (-0.062 to 0.010)	0.95 (0.87 to 1.03)
Belgium	2021	Open prostatectomy	5,530	0.477	5,180 (4,620 to 5,790)	0.447 (0.399 to 0.500)	350 (-266 to 915)	0.030 (-0.023 to 0.079)	1.07 (0.95 to 1.20)
Belgium	2020	Transurethral prostatectomy	6,990	0.605	8,780 (8,370 to 9,200)	0.760 (0.725 to 0.797)	-1,790 (-2,210 to -1,380)	-0.155 (-0.192 to -0.120)	0.80 (0.76 to 0.84)
Belgium	2021	Transurethral prostatectomy	8,520	0.735	8,650 (8,090 to 9,250)	0.747 (0.698 to 0.798)	-135 (-739 to 416)	-0.012 (-0.064 to 0.036)	0.98 (0.92 to 1.05)
Canada	2020	All surgeries	903,000	23.7	1,080,000 (1,050,000 to 1,100,000)	28.3 (27.7 to 29.0)	-175,000 (-199,000 to -151,000)	-4.60 (-5.23 to -3.97)	0.84 (0.82 to 0.86)
Canada	2021	All surgeries	980,000	25.6	1,080,000 (1,060,000 to 1,110,000)	28.3 (27.7 to 29.0)	-104,000 (-128,000 to -79,600)	-2.71 (-3.35 to -2.08)	0.90 (0.88 to 0.92)
Canada	2020	Non-elective surgeries	152,000	3.99	167,000 (163,000 to 170,000)	4.38 (4.29 to 4.47)	-14,600 (-18,300 to -11,200)	-0.384 (-0.481 to -0.294)	0.91 (0.89 to 0.93)
Canada	2021	Non-elective surgeries	159,000	4.15	166,000 (162,000 to 170,000)	4.34 (4.24 to 4.43)	-6,910 (-10,800 to -3,320)	-0.181 (-0.281 to -0.087)	0.96 (0.94 to 0.98)
Canada	2020	Appendectomy	41,300	1.09	42,300 (40,700 to 43,800)	1.11 (1.07 to 1.15)	-936 (-2,480 to 570)	-0.025 (-0.065 to 0.015)	0.98 (0.94 to 1.01)
Canada	2021	Appendectomy	41,900	1.09	42,500 (40,300 to 44,800)	1.11 (1.05 to 1.17)	-631 (-2,840 to 1,480)	-0.016 (-0.074 to 0.039)	0.99 (0.94 to 1.04)
Canada	2020	Cholecystectomy	71,000	1.87	75,300 (72,500 to 78,300)	1.98 (1.91 to 2.06)	-4,300 (-7,160 to -1,540)	-0.113 (-0.188 to -0.040)	0.94 (0.91 to 0.98)
Canada	2021	Cholecystectomy	72,200	1.89	75,800 (71,800 to 80,000)	1.98 (1.88 to 2.09)	-3,600 (-7,850 to 340)	-0.094 (-0.205 to 0.009)	0.95 (0.90 to 1.00)
Canada	2020	Total mastectomy	9,440	0.248	10,700 (9,840 to 11,700)	0.282 (0.259 to 0.307)	-1,280 (-2,240 to -411)	-0.034 (-0.059 to -0.011)	0.88 (0.81 to 0.96)
Canada	2021	Total mastectomy	9,960	0.26	10,800 (9,550 to 12,200)	0.282 (0.250 to 0.318)	-821 (-2,200 to 389)	-0.021 (-0.058 to 0.010)	0.92 (0.82 to 1.04)
Canada	2020	Partial excision of mammary gland	30,100	0.792	35,800 (34,600 to 37,100)	0.942 (0.908 to 0.976)	-5,680 (-7,030 to -4,420)	-0.149 (-0.185 to -0.116)	0.84 (0.81 to 0.87)
Canada	2021	Partial excision of mammary gland	34,900	0.913	36,000 (34,200 to 37,900)	0.942 (0.895 to 0.991)	-1,090 (-3,000 to 682)	-0.028 (-0.078 to 0.018)	0.97 (0.92 to 1.02)

Canada	2020	Both	232,000	6.1	253,000 (248,000 to 258,000)	6.66 (6.52 to 6.79)	-21,000 (-26,400 to -15,900)	-0.553 (-0.693 to -0.419)	0.92 (0.90 to 0.94)
Canada	2021	Both	240,000	6.28	255,000 (247,000 to 262,000)	6.66 (6.47 to 6.85)	-14,200 (-21,900 to -6,940)	-0.372 (-0.572 to -0.181)	0.94 (0.92 to 0.97)
Canada	2020	Caesarean section	107,000	2.81	109,000 (107,000 to 110,000)	2.86 (2.82 to 2.90)	-1,990 (-3,470 to -575)	-0.052 (-0.091 to -0.015)	0.98 (0.97 to 0.99)
Canada	2021	Caesarean section	113,000	2.94	109,000 (107,000 to 111,000)	2.86 (2.80 to 2.91)	3,240 (1,200 to 5,240)	0.085 (0.031 to 0.137)	1.03 (1.01 to 1.05)
Canada	2020	Repair of inguinal hernia	54,600	1.43	64,100 (61,800 to 66,500)	1.69 (1.62 to 1.75)	-9,560 (-12,000 to -7,200)	-0.251 (-0.315 to -0.189)	0.85 (0.82 to 0.88)
Canada	2021	Repair of inguinal hernia	55,700	1.46	62,600 (60,300 to 65,100)	1.64 (1.58 to 1.70)	-6,910 (-9,410 to -4,530)	-0.181 (-0.246 to -0.118)	0.89 (0.86 to 0.92)
Canada	2020	Transluminal coronary angioplasty	53,700	1.41	60,000 (57,900 to 62,100)	1.58 (1.52 to 1.63)	-6,260 (-8,350 to -4,180)	-0.165 (-0.220 to -0.110)	0.90 (0.87 to 0.93)
Canada	2021	Transluminal coronary angioplasty	54,400	1.42	60,300 (57,400 to 63,300)	1.58 (1.50 to 1.66)	-5,900 (-8,960 to -3,000)	-0.154 (-0.234 to -0.079)	0.90 (0.86 to 0.95)
Canada	2020	Coronary artery bypass graft	17,100	0.451	20,500 (19,800 to 21,300)	0.540 (0.521 to 0.560)	-3,400 (-4,180 to -2,660)	-0.089 (-0.110 to -0.070)	0.83 (0.80 to 0.87)
Canada	2021	Coronary artery bypass graft	17,700	0.462	20,700 (19,600 to 21,800)	0.540 (0.513 to 0.569)	-3,000 (-4,100 to -1,980)	-0.078 (-0.107 to -0.052)	0.85 (0.81 to 0.90)
Canada	2020	Elective surgeries	519,000	13.6	660,000 (633,000 to 688,000)	17.3 (16.6 to 18.1)	-140,000 (-169,000 to -114,000)	-3.69 (-4.43 to -3.00)	0.79 (0.75 to 0.82)
Canada	2021	Elective surgeries	581,000	15.2	663,000 (625,000 to 704,000)	17.3 (16.3 to 18.4)	-82,100 (-121,000 to -43,200)	-2.15 (-3.17 to -1.13)	0.88 (0.83 to 0.93)
Canada	2020	Hip replacement	55,000	1.45	66,000 (64,600 to 67,400)	1.74 (1.70 to 1.77)	-11,000 (-12,400 to -9,620)	-0.290 (-0.326 to -0.253)	0.83 (0.82 to 0.85)
Canada	2021	Hip replacement	58,400	1.53	68,500 (66,500 to 70,600)	1.79 (1.74 to 1.85)	-10,100 (-12,200 to -8,150)	-0.264 (-0.319 to -0.213)	0.85 (0.83 to 0.88)
Canada	2020	Total knee replacement	54,900	1.44	77,800 (74,100 to 81,700)	2.05 (1.95 to 2.15)	-22,900 (-26,700 to -19,200)	-0.602 (-0.703 to -0.504)	0.71 (0.67 to 0.74)
Canada	2021	Total knee replacement	58,100	1.52	80,700 (75,400 to 86,500)	2.11 (1.97 to 2.26)	-22,600 (-28,500 to -17,400)	-0.592 (-0.745 to -0.454)	0.72 (0.67 to 0.77)
Canada	2020	Cataract surgery	326,000	8.58	412,000 (396,000 to 428,000)	10.8 (10.4 to 11.2)	-85,100 (-102,000 to -69,400)	-2.24 (-2.68 to -1.82)	0.79 (0.76 to 0.82)
Canada	2021	Cataract surgery	384,000	10	414,000 (398,000 to 430,000)	10.8 (10.4 to 11.2)	-30,100 (-46,300 to -14,400)	-0.786 (-1.21 to -0.375)	0.93 (0.89 to 0.96)
Canada	2020	Tonsillectomy	18,800	0.494	28,100 (25,700 to 30,600)	0.738 (0.677 to 0.805)	-9,260 (-11,800 to -6,930)	-0.243 (-0.309 to -0.182)	0.67 (0.62 to 0.73)
Canada	2021	Tonsillectomy	15,100	0.394	27,300 (24,200 to 30,900)	0.715 (0.632 to 0.808)	-12,300 (-15,800 to -9,100)	-0.321 (-0.412 to -0.238)	0.55 (0.49 to 0.62)
Canada	2020	Hysterectomy	35,400	0.931	41,500 (40,100 to 42,900)	1.09 (1.05 to 1.13)	-6,080 (-7,520 to -4,680)	-0.160 (-0.198 to -0.123)	0.85 (0.82 to 0.88)

Canada	2021	Hysterectomy	35,900	0.94	41,000 (39,100 to 43,000)	1.07 (1.02 to 1.12)	-5,070 (-7,140 to -3,140)	-0.133 (-0.187 to -0.082)	0.88 (0.83 to 0.92)
Canada	2020	Open prostatectomy	10,400	0.274	11,100 (10,300 to 11,800)	0.291 (0.271 to 0.311)	-630 (-1,400 to 102)	-0.017 (-0.037 to 0.003)	0.94 (0.88 to 1.01)
Canada	2021	Open prostatectomy	10,600	0.278	11,200 (10,300 to 12,200)	0.292 (0.269 to 0.318)	-570 (-1,560 to 341)	-0.015 (-0.041 to 0.009)	0.95 (0.87 to 1.03)
Canada	2020	Transurethral prostatectomy	18,200	0.479	22,200 (21,300 to 23,200)	0.584 (0.561 to 0.609)	-4,000 (-4,900 to -3,110)	-0.105 (-0.129 to -0.082)	0.82 (0.79 to 0.85)
Canada	2021	Transurethral prostatectomy	19,300	0.504	22,400 (21,500 to 23,300)	0.584 (0.561 to 0.609)	-3,060 (-3,980 to -2,180)	-0.080 (-0.104 to -0.057)	0.86 (0.83 to 0.90)
Switzerland	2020	All surgeries	232,000	26.8	243,000 (233,000 to 254,000)	28.1 (27.0 to 29.4)	-11,300 (-22,000 to -1,060)	-1.30 (-2.55 to -0.123)	0.95 (0.91 to 1.00)
Switzerland	2021	All surgeries	251,000	28.8	245,000 (235,000 to 256,000)	28.1 (27.0 to 29.4)	5,840 (-4,870 to 15,900)	0.671 (-0.559 to 1.83)	1.02 (0.98 to 1.07)
Switzerland	2020	Non-elective surgeries	41,800	4.84	43,400 (42,300 to 44,500)	5.02 (4.90 to 5.15)	-1,520 (-2,630 to -473)	-0.176 (-0.304 to -0.055)	0.96 (0.94 to 0.99)
Switzerland	2021	Non-elective surgeries	45,100	5.18	43,700 (42,600 to 44,800)	5.02 (4.90 to 5.15)	1,380 (261 to 2,460)	0.159 (0.030 to 0.282)	1.03 (1.01 to 1.06)
Switzerland	2020	Appendectomy	14,500	1.68	14,300 (13,700 to 14,800)	1.65 (1.59 to 1.71)	225 (-317 to 745)	0.026 (-0.037 to 0.086)	1.02 (0.98 to 1.05)
Switzerland	2021	Appendectomy	14,600	1.68	14,400 (13,600 to 15,200)	1.65 (1.57 to 1.74)	223 (-538 to 964)	0.026 (-0.062 to 0.111)	1.02 (0.96 to 1.07)
Switzerland	2020	Cholecystectomy	16,700	1.94	17,700 (17,200 to 18,200)	2.05 (1.99 to 2.11)	-970 (-1,460 to -495)	-0.112 (-0.169 to -0.057)	0.95 (0.92 to 0.97)
Switzerland	2021	Cholecystectomy	18,900	2.17	17,800 (17,400 to 18,300)	2.05 (1.99 to 2.11)	1,020 (520 to 1,510)	0.117 (0.060 to 0.174)	1.06 (1.03 to 1.09)
Switzerland	2020	Total mastectomy	2,880	0.333	3,030 (2,820 to 3,260)	0.351 (0.327 to 0.377)	-154 (-381 to 58)	-0.018 (-0.044 to 0.007)	0.95 (0.88 to 1.02)
Switzerland	2021	Total mastectomy	3,240	0.373	3,060 (2,840 to 3,290)	0.351 (0.327 to 0.377)	187 (-47 to 398)	0.021 (-0.005 to 0.046)	1.06 (0.99 to 1.14)
Switzerland	2020	Partial excision of mammary gland	7,740	0.896	8,240 (7,830 to 8,660)	0.953 (0.906 to 1.00)	-499 (-937 to -86)	-0.058 (-0.108 to -0.010)	0.94 (0.89 to 0.99)
Switzerland	2021	Partial excision of mammary gland	8,380	0.963	8,300 (7,890 to 8,730)	0.953 (0.906 to 1.00)	79 (-354 to 491)	0.009 (-0.041 to 0.056)	1.01 (0.96 to 1.06)
Switzerland	2020	Both	76,700	8.88	81,600 (79,200 to 84,100)	9.45 (9.17 to 9.74)	-4,900 (-7,390 to -2,490)	-0.568 (-0.855 to -0.288)	0.94 (0.91 to 0.97)
Switzerland	2021	Both	81,800	9.4	82,200 (78,800 to 85,800)	9.45 (9.06 to 9.86)	-394 (-4,000 to 3,050)	-0.045 (-0.459 to 0.350)	1.00 (0.95 to 1.04)
Switzerland	2020	Caesarean section	27,400	3.17	27,500 (26,300 to 28,800)	3.18 (3.04 to 3.33)	-87 (-1,390 to 1,140)	-0.010 (-0.161 to 0.132)	1.00 (0.95 to 1.04)
Switzerland	2021	Caesarean section	29,200	3.36	27,700 (26,000 to 29,500)	3.18 (2.98 to 3.39)	1,520 (-319 to 3,220)	0.175 (-0.037 to 0.370)	1.06 (0.99 to 1.12)
Switzerland	2020	Repair of inguinal hernia	20,000	2.31	22,400 (21,700 to 23,100)	2.59 (2.51 to 2.68)	-2,430 (-3,200 to -1,710)	-0.281 (-0.370 to -0.198)	0.89 (0.86 to 0.92)
Switzerland	2021	Repair of inguinal hernia	21,600	2.49	22,600 (21,800 to 23,300)	2.59 (2.51 to 2.68)	-917 (-1,670 to -199)	-0.105 (-0.192 to -0.023)	0.96 (0.93 to 0.99)
Switzerland	2020	Transluminal coronary angioplasty	26,400	3.05	29,700 (27,800 to 31,700)	3.44 (3.22 to 3.67)	-3,340 (-5,370 to -1,460)	-0.387 (-0.622 to -0.170)	0.89 (0.83 to 0.95)

Switzerland	2021	Transluminal coronary angioplasty	27,600	3.18	31,500 (28,700 to 34,600)	3.62 (3.30 to 3.98)	-3,900 (-7,080 to -1,080)	-0.447 (-0.813 to -0.124)	0.88 (0.80 to 0.96)
Switzerland	2020	Coronary artery bypass graft	3,000	0.348	3,390 (3,100 to 3,720)	0.393 (0.359 to 0.430)	-391 (-715 to -96)	-0.045 (-0.083 to -0.011)	0.88 (0.81 to 0.97)
Switzerland	2021	Coronary artery bypass graft	3,340	0.384	3,420 (3,000 to 3,890)	0.393 (0.345 to 0.447)	-79 (-549 to 337)	-0.009 (-0.063 to 0.039)	0.98 (0.86 to 1.11)
Switzerland	2020	Elective surgeries	113,000	13.1	120,000 (112,000 to 128,000)	13.8 (13.0 to 14.8)	-6,250 (-14,300 to 1,400)	-0.723 (-1.65 to 0.162)	0.95 (0.89 to 1.01)
Switzerland	2021	Elective surgeries	124,000	14.2	121,000 (113,000 to 129,000)	13.8 (13.0 to 14.8)	3,430 (-4,780 to 11,200)	0.394 (-0.549 to 1.29)	1.03 (0.96 to 1.10)
Switzerland	2020	Hip replacement	26,400	3.06	26,600 (25,700 to 27,500)	3.08 (2.98 to 3.18)	-162 (-1,040 to 699)	-0.019 (-0.121 to 0.081)	0.99 (0.96 to 1.03)
Switzerland	2021	Hip replacement	28,100	3.23	26,800 (25,900 to 27,700)	3.08 (2.98 to 3.18)	1,280 (376 to 2,130)	0.148 (0.043 to 0.245)	1.05 (1.01 to 1.08)
Switzerland	2020	Total knee replacement	22,100	2.56	21,200 (18,400 to 24,400)	2.46 (2.13 to 2.83)	873 (-2,320 to 3,680)	0.101 (-0.268 to 0.426)	1.04 (0.91 to 1.20)
Switzerland	2021	Total knee replacement	23,800	2.73	21,400 (18,600 to 24,600)	2.46 (2.13 to 2.83)	2,400 (-761 to 5,190)	0.276 (-0.087 to 0.596)	1.11 (0.97 to 1.28)
Switzerland	2020	Cataract surgery	34,300	3.97	36,800 (31,700 to 42,700)	4.26 (3.67 to 4.94)	-2,470 (-8,490 to 2,640)	-0.286 (-0.983 to 0.306)	0.93 (0.80 to 1.08)
Switzerland	2021	Cataract surgery	39,300	4.52	37,100 (31,900 to 43,000)	4.26 (3.67 to 4.94)	2,250 (-3,710 to 7,300)	0.259 (-0.426 to 0.839)	1.06 (0.91 to 1.23)
Switzerland	2020	Tonsillectomy	6,470	0.749	9,020 (8,300 to 9,810)	1.04 (0.961 to 1.14)	-2,560 (-3,330 to -1,840)	-0.296 (-0.385 to -0.213)	0.72 (0.66 to 0.78)
Switzerland	2021	Tonsillectomy	6,120	0.703	9,090 (8,080 to 10,200)	1.04 (0.929 to 1.17)	-2,970 (-4,110 to -1,950)	-0.341 (-0.472 to -0.224)	0.67 (0.60 to 0.76)
Switzerland	2020	Hysterectomy	10,400	1.2	11,900 (11,400 to 12,400)	1.38 (1.32 to 1.43)	-1,540 (-2,010 to -1,070)	-0.178 (-0.233 to -0.124)	0.87 (0.84 to 0.91)
Switzerland	2021	Hysterectomy	11,500	1.33	12,000 (11,300 to 12,700)	1.38 (1.30 to 1.46)	-444 (-1,150 to 229)	-0.051 (-0.132 to 0.026)	0.96 (0.91 to 1.02)
Switzerland	2020	Open prostatectomy	3,700	0.429	3,600 (3,120 to 4,150)	0.416 (0.361 to 0.480)	108 (-444 to 581)	0.012 (-0.051 to 0.067)	1.03 (0.89 to 1.19)
Switzerland	2021	Open prostatectomy	4,010	0.461	3,620 (2,960 to 4,430)	0.416 (0.340 to 0.509)	387 (-415 to 1,050)	0.044 (-0.048 to 0.120)	1.11 (0.91 to 1.35)
Switzerland	2020	Transurethral prostatectomy	9,990	1.16	9,940 (9,360 to 10,600)	1.15 (1.08 to 1.22)	46 (-574 to 629)	0.005 (-0.066 to 0.073)	1.00 (0.95 to 1.07)
Switzerland	2021	Transurethral prostatectomy	11,100	1.27	10,000 (9,430 to 10,700)	1.15 (1.08 to 1.22)	1,070 (418 to 1,660)	0.123 (0.048 to 0.191)	1.11 (1.04 to 1.18)
Costa Rica	2020	All surgeries	44,000	8.61	70,100 (63,900 to 76,900)	13.7 (12.5 to 15.1)	-26,100 (-32,900 to -20,000)	-5.10 (-6.44 to -3.92)	0.63 (0.57 to 0.69)
Costa Rica	2021	All surgeries	50,700	9.83	70,800 (62,100 to 80,800)	13.7 (12.0 to 15.6)	-20,100 (-30,100 to -11,500)	-3.89 (-5.84 to -2.23)	0.72 (0.63 to 0.81)
Costa Rica	2020	Non-elective surgeries	14,100	2.75	19,200 (17,600 to 21,000)	3.76 (3.44 to 4.11)	-5,170 (-6,980 to -3,540)	-1.01 (-1.37 to -0.692)	0.73 (0.67 to 0.80)
Costa Rica	2021	Non-elective surgeries	15,600	3.02	19,400 (17,800 to 21,200)	3.76 (3.44 to 4.11)	-3,850 (-5,680 to -2,220)	-0.746 (-1.10 to -0.430)	0.80 (0.73 to 0.88)
Costa Rica	2020	Appendectomy	6,650	1.3	6,940 (6,490 to 7,420)	1.36 (1.27 to 1.45)	-289 (-764 to 156)	-0.057 (-0.150 to 0.030)	0.96 (0.90 to 1.02)
Costa Rica	2021	Appendectomy	6,640	1.29	7,010 (6,380 to 7,700)	1.36 (1.24 to 1.49)	-370 (-1,060 to 271)	-0.072 (-0.206 to 0.053)	0.95 (0.86 to 1.04)
Costa Rica	2020	Cholecystectomy	5,470	1.07	10,500 (8,490 to 13,100)	2.06 (1.66 to 2.56)	-5,060 (-7,680 to -2,970)	-0.990 (-1.50 to -0.581)	0.52 (0.42 to 0.65)

Costa Rica	2021	Cholecystectomy	6,830	1.32	10,700 (8,510 to 13,500)	2.08 (1.65 to 2.62)	-3,910 (-6,720 to -1,650)	-0.757 (-1.30 to -0.320)	0.64 (0.50 to 0.80)
Costa Rica	2020	Total mastectomy	637	0.125	827 (692 to 988)	0.162 (0.135 to 0.193)	-190 (-348 to -53)	-0.037 (-0.068 to -0.010)	0.77 (0.65 to 0.92)
Costa Rica	2021	Total mastectomy	686	0.133	835 (649 to 1,070)	0.162 (0.126 to 0.208)	-149 (-387 to 39)	-0.029 (-0.075 to 0.007)	0.82 (0.64 to 1.06)
Costa Rica	2020	Partial excision of mammary gland	1,300	0.254	1,780 (1,510 to 2,090)	0.348 (0.296 to 0.408)	-481 (-795 to -218)	-0.094 (-0.156 to -0.043)	0.73 (0.62 to 0.86)
Costa Rica	2021	Partial excision of mammary gland	1,420	0.275	1,800 (1,430 to 2,250)	0.348 (0.277 to 0.437)	-374 (-829 to -10)	-0.072 (-0.161 to -0.002)	0.79 (0.63 to 0.99)
Costa Rica	2020	Both	16,400	3.2	22,000 (20,200 to 23,900)	4.30 (3.94 to 4.68)	-5,600 (-7,590 to -3,780)	-1.10 (-1.49 to -0.740)	0.74 (0.68 to 0.81)
Costa Rica	2021	Both	16,700	3.23	22,200 (20,400 to 24,200)	4.30 (3.94 to 4.68)	-5,520 (-7,480 to -3,650)	-1.07 (-1.45 to -0.707)	0.75 (0.69 to 0.82)
Costa Rica	2020	Caesarean section	13,800	2.7	14,900 (13,900 to 15,900)	2.91 (2.72 to 3.11)	-1,040 (-2,070 to -87)	-0.204 (-0.405 to -0.017)	0.93 (0.87 to 0.99)
Costa Rica	2021	Caesarean section	12,800	2.48	15,000 (13,700 to 16,500)	2.91 (2.65 to 3.19)	-2,180 (-3,670 to -851)	-0.423 (-0.710 to -0.165)	0.85 (0.78 to 0.94)
Costa Rica	2020	Repair of inguinal hernia	1,750	0.343	5,110 (4,140 to 6,310)	1.00 (0.810 to 1.23)	-3,360 (-4,580 to -2,370)	-0.657 (-0.896 to -0.465)	0.34 (0.28 to 0.42)
Costa Rica	2021	Repair of inguinal hernia	2,500	0.483	5,160 (4,180 to 6,380)	1.00 (0.810 to 1.23)	-2,670 (-3,880 to -1,670)	-0.517 (-0.752 to -0.323)	0.48 (0.39 to 0.60)
Costa Rica	2020	Transluminal coronary angioplasty	701	0.137	738 (424 to 1,280)	0.144 (0.083 to 0.251)	-37 (-581 to 270)	-0.007 (-0.114 to 0.053)	0.95 (0.55 to 1.63)
Costa Rica	2021	Transluminal coronary angioplasty	1,200	0.232	745 (340 to 1,630)	0.144 (0.066 to 0.316)	454 (-437 to 863)	0.088 (-0.085 to 0.167)	1.61 (0.73 to 3.57)
Costa Rica	2020	Coronary artery bypass graft	96	0.019	139 (81 to 241)	0.027 (0.016 to 0.047)	-43 (-146 to 16)	-0.009 (-0.029 to 0.003)	0.69 (0.40 to 1.21)
Costa Rica	2021	Coronary artery bypass graft	145	0.028	141 (65 to 305)	0.027 (0.013 to 0.059)	4 (-159 to 79)	0.001 (-0.031 to 0.015)	1.03 (0.48 to 2.21)
Costa Rica	2020	Elective surgeries	13,600	2.66	26,500 (23,400 to 30,000)	5.18 (4.58 to 5.86)	-12,900 (-16,400 to -9,780)	-2.52 (-3.20 to -1.91)	0.51 (0.45 to 0.58)
Costa Rica	2021	Elective surgeries	18,500	3.58	31,200 (27,500 to 35,400)	6.04 (5.33 to 6.85)	-12,700 (-16,900 to -9,120)	-2.46 (-3.27 to -1.77)	0.59 (0.52 to 0.67)
Costa Rica	2020	Hip replacement	722	0.141	1,190 (950 to 1,490)	0.233 (0.186 to 0.291)	-467 (-764 to -225)	-0.091 (-0.149 to -0.044)	0.61 (0.49 to 0.76)
Costa Rica	2021	Hip replacement	922	0.179	1,200 (874 to 1,650)	0.233 (0.169 to 0.320)	-279 (-731 to 55)	-0.054 (-0.142 to 0.011)	0.77 (0.56 to 1.06)
Costa Rica	2020	Total knee replacement	507	0.099	1,660 (1,060 to 2,600)	0.325 (0.207 to 0.509)	-1,150 (-2,120 to -547)	-0.226 (-0.415 to -0.107)	0.31 (0.19 to 0.48)
Costa Rica	2021	Total knee replacement	498	0.096	1,960 (1,040 to 3,700)	0.379 (0.201 to 0.716)	-1,460 (-3,190 to -539)	-0.283 (-0.617 to -0.104)	0.25 (0.14 to 0.48)
Costa Rica	2020	Cataract surgery	7,580	1.48	17,800 (14,400 to 21,900)	3.48 (2.82 to 4.29)	-10,200 (-14,300 to -6,920)	-2.00 (-2.79 to -1.35)	0.43 (0.35 to 0.52)
Costa Rica	2021	Cataract surgery	11,700	2.26	19,700 (14,700 to 26,500)	3.82 (2.84 to 5.14)	-8,030 (-14,900 to -3,050)	-1.56 (-2.88 to -0.590)	0.59 (0.44 to 0.79)
Costa Rica	2020	Tonsillectomy	691	0.135	2,770 (2,360 to 3,260)	0.542 (0.461 to 0.638)	-2,080 (-2,570 to -1,660)	-0.407 (-0.503 to -0.325)	0.25 (0.21 to 0.29)
Costa Rica	2021	Tonsillectomy	906	0.175	2,780 (2,290 to 3,370)	0.538 (0.443 to 0.653)	-1,870 (-2,460 to -1,380)	-0.362 (-0.476 to -0.267)	0.33 (0.27 to 0.40)

Costa Rica	2020	Hysterectomy	2,890	0.566	4,040 (3,710 to 4,400)	0.791 (0.726 to 0.861)	-1,150 (-1,500 to -821)	-0.225 (-0.293 to -0.161)	0.72 (0.66 to 0.78)
Costa Rica	2021	Hysterectomy	3,330	0.645	4,080 (3,620 to 4,600)	0.791 (0.701 to 0.892)	-752 (-1,280 to -285)	-0.146 (-0.247 to -0.055)	0.82 (0.72 to 0.92)
Costa Rica	2020	Open prostatectomy	494	0.097	963 (841 to 1,100)	0.188 (0.164 to 0.216)	-469 (-608 to -346)	-0.092 (-0.119 to -0.068)	0.51 (0.45 to 0.59)
Costa Rica	2021	Open prostatectomy	503	0.097	1,040 (855 to 1,260)	0.201 (0.166 to 0.243)	-533 (-759 to -350)	-0.103 (-0.147 to -0.068)	0.49 (0.40 to 0.59)
Costa Rica	2020	Transurethral prostatectomy	710	0.139	1,930 (1,440 to 2,600)	0.378 (0.281 to 0.508)	-1,220 (-1,880 to -724)	-0.239 (-0.367 to -0.142)	0.37 (0.27 to 0.50)
Costa Rica	2021	Transurethral prostatectomy	636	0.123	1,950 (1,280 to 2,970)	0.378 (0.249 to 0.574)	-1,320 (-2,300 to -655)	-0.255 (-0.446 to -0.127)	0.33 (0.22 to 0.49)
Czechia	2020	All surgeries	274,000	25.6	313,000 (306,000 to 321,000)	29.3 (28.6 to 30.0)	-39,200 (-46,900 to -31,700)	-3.66 (-4.38 to -2.96)	0.88 (0.85 to 0.90)
Czechia	2021	All surgeries	282,000	26.9	311,000 (303,000 to 318,000)	29.6 (28.8 to 30.3)	-28,200 (-36,200 to -20,700)	-2.69 (-3.45 to -1.97)	0.91 (0.89 to 0.93)
Czechia	2020	Non-elective surgeries	31,300	2.92	37,500 (35,800 to 39,300)	3.50 (3.34 to 3.67)	-6,210 (-8,020 to -4,500)	-0.580 (-0.749 to -0.420)	0.83 (0.80 to 0.87)
Czechia	2021	Non-elective surgeries	31,900	3.03	36,800 (35,100 to 38,600)	3.50 (3.34 to 3.67)	-4,940 (-6,710 to -3,300)	-0.470 (-0.639 to -0.314)	0.87 (0.83 to 0.91)
Czechia	2020	Appendectomy	13,400	1.26	14,400 (13,700 to 15,100)	1.34 (1.28 to 1.41)	-938 (-1,670 to -234)	-0.088 (-0.156 to -0.022)	0.93 (0.89 to 0.98)
Czechia	2021	Appendectomy	14,200	1.35	14,100 (13,200 to 15,100)	1.34 (1.25 to 1.44)	115 (-897 to 1,090)	0.011 (-0.085 to 0.104)	1.01 (0.94 to 1.08)
Czechia	2020	Cholecystectomy	17,800	1.67	22,400 (21,400 to 23,500)	2.10 (2.00 to 2.20)	-4,580 (-5,650 to -3,540)	-0.428 (-0.528 to -0.331)	0.80 (0.76 to 0.83)
Czechia	2021	Cholecystectomy	17,600	1.68	22,000 (21,000 to 23,100)	2.10 (2.00 to 2.20)	-4,380 (-5,450 to -3,330)	-0.416 (-0.519 to -0.317)	0.80 (0.76 to 0.84)
Czechia	2020	Both	65,000	6.07	73,400 (70,900 to 76,000)	6.86 (6.63 to 7.10)	-8,430 (-10,900 to -6,000)	-0.788 (-1.02 to -0.561)	0.89 (0.86 to 0.92)
Czechia	2021	Both	66,700	6.35	72,100 (69,700 to 74,600)	6.86 (6.63 to 7.10)	-5,380 (-7,870 to -2,990)	-0.512 (-0.749 to -0.284)	0.93 (0.89 to 0.96)
Czechia	2020	Caesarean section	26,200	2.45	27,000 (25,400 to 28,800)	2.53 (2.38 to 2.69)	-806 (-2,530 to 815)	-0.075 (-0.236 to 0.076)	0.97 (0.91 to 1.03)
Czechia	2021	Caesarean section	27,700	2.64	26,600 (25,000 to 28,200)	2.53 (2.38 to 2.69)	1,160 (-535 to 2,750)	0.110 (-0.051 to 0.262)	1.04 (0.98 to 1.11)
Czechia	2020	Repair of inguinal hernia	13,800	1.29	18,600 (17,900 to 19,200)	1.74 (1.68 to 1.80)	-4,770 (-5,420 to -4,130)	-0.446 (-0.506 to -0.386)	0.74 (0.72 to 0.77)
Czechia	2021	Repair of inguinal hernia	14,000	1.33	18,200 (17,600 to 18,900)	1.74 (1.68 to 1.80)	-4,270 (-4,910 to -3,640)	-0.406 (-0.468 to -0.347)	0.77 (0.74 to 0.79)
Czechia	2020	Transluminal coronary angioplasty	21,000	1.96	23,100 (22,200 to 24,000)	2.16 (2.07 to 2.24)	-2,080 (-2,970 to -1,190)	-0.194 (-0.278 to -0.111)	0.91 (0.88 to 0.95)
Czechia	2021	Transluminal coronary angioplasty	21,200	2.02	23,000 (22,100 to 23,900)	2.19 (2.10 to 2.27)	-1,730 (-2,660 to -818)	-0.165 (-0.253 to -0.078)	0.92 (0.89 to 0.96)
Czechia	2020	Coronary artery bypass graft	3,920	0.366	4,540 (4,270 to 4,830)	0.424 (0.399 to 0.452)	-621 (-914 to -347)	-0.058 (-0.085 to -0.032)	0.86 (0.81 to 0.92)
Czechia	2021	Coronary artery bypass graft	3,760	0.358	4,350 (3,990 to 4,750)	0.414 (0.379 to 0.452)	-588 (-992 to -213)	-0.056 (-0.094 to -0.020)	0.86 (0.79 to 0.95)

Czechia	2020	Elective surgeries	178,000	16.6	205,000 (199,000 to 211,000)	19.2 (18.6 to 19.7)	-27,100 (-32,800 to -21,600)	-2.53 (-3.07 to -2.01)	0.87 (0.84 to 0.89)
Czechia	2021	Elective surgeries	184,000	17.5	204,000 (199,000 to 211,000)	19.5 (18.9 to 20.0)	-20,700 (-26,800 to -14,900)	-1.97 (-2.55 to -1.42)	0.90 (0.87 to 0.93)
Czechia	2020	Hip replacement	19,500	1.82	18,500 (16,600 to 20,700)	1.73 (1.55 to 1.93)	1,010 (-1,160 to 2,950)	0.095 (-0.109 to 0.275)	1.05 (0.94 to 1.18)
Czechia	2021	Hip replacement	20,800	1.98	18,200 (15,500 to 21,200)	1.73 (1.48 to 2.02)	2,630 (-409 to 5,260)	0.250 (-0.039 to 0.501)	1.14 (0.98 to 1.34)
Czechia	2020	Total knee replacement	12,300	1.15	13,000 (11,200 to 15,000)	1.21 (1.05 to 1.40)	-616 (-2,620 to 1,120)	-0.058 (-0.245 to 0.104)	0.95 (0.82 to 1.10)
Czechia	2021	Total knee replacement	11,400	1.08	12,700 (10,400 to 15,600)	1.21 (0.989 to 1.48)	-1,360 (-4,210 to 980)	-0.129 (-0.401 to 0.093)	0.89 (0.73 to 1.09)
Czechia	2020	Cataract surgery	126,000	11.7	146,000 (137,000 to 155,000)	13.6 (12.8 to 14.5)	-20,100 (-29,500 to -11,200)	-1.88 (-2.76 to -1.04)	0.86 (0.81 to 0.92)
Czechia	2021	Cataract surgery	132,000	12.5	148,000 (135,000 to 162,000)	14.1 (12.9 to 15.4)	-16,300 (-30,200 to -3,790)	-1.55 (-2.87 to -0.361)	0.89 (0.81 to 0.97)
Czechia	2020	Tonsillectomy	3,850	0.36	5,580 (5,240 to 5,950)	0.522 (0.489 to 0.556)	-1,740 (-2,100 to -1,390)	-0.162 (-0.196 to -0.130)	0.69 (0.65 to 0.73)
Czechia	2021	Tonsillectomy	3,000	0.285	5,300 (4,840 to 5,810)	0.505 (0.461 to 0.553)	-2,310 (-2,800 to -1,840)	-0.219 (-0.266 to -0.176)	0.57 (0.52 to 0.62)
Czechia	2020	Hysterectomy	12,700	1.19	15,600 (14,600 to 16,700)	1.46 (1.36 to 1.56)	-2,890 (-3,980 to -1,850)	-0.270 (-0.372 to -0.173)	0.81 (0.76 to 0.87)
Czechia	2021	Hysterectomy	13,000	1.24	15,300 (13,900 to 16,900)	1.46 (1.32 to 1.61)	-2,310 (-3,880 to -909)	-0.220 (-0.369 to -0.087)	0.85 (0.77 to 0.93)
Czechia	2020	Open prostatectomy	942	0.088	1,360 (1,180 to 1,580)	0.128 (0.110 to 0.148)	-423 (-635 to -237)	-0.039 (-0.059 to -0.022)	0.69 (0.60 to 0.80)
Czechia	2021	Open prostatectomy	1,030	0.098	1,250 (1,020 to 1,540)	0.119 (0.097 to 0.147)	-218 (-507 to 17)	-0.021 (-0.048 to 0.002)	0.83 (0.67 to 1.02)
Czechia	2020	Transurethral prostatectomy	3,190	0.299	4,150 (3,760 to 4,580)	0.388 (0.351 to 0.428)	-952 (-1,390 to -561)	-0.089 (-0.130 to -0.052)	0.77 (0.70 to 0.85)
Czechia	2021	Transurethral prostatectomy	2,960	0.282	4,070 (3,690 to 4,500)	0.388 (0.351 to 0.428)	-1,110 (-1,530 to -721)	-0.106 (-0.146 to -0.069)	0.73 (0.66 to 0.80)
Germany	2020	All surgeries	2,670,000	32.1	2,870,000 (2,800,000 to 2,940,000)	34.5 (33.6 to 35.3)	-197,000 (-268,000 to -130,000)	-2.36 (-3.22 to -1.57)	0.93 (0.91 to 0.95)
Germany	2021	All surgeries	2,760,000	33.2	2,870,000 (2,770,000 to 2,970,000)	34.5 (33.3 to 35.7)	-108,000 (-207,000 to -10,300)	-1.30 (-2.49 to -0.123)	0.96 (0.93 to 1.00)
Germany	2020	Non-elective surgeries	408,000	4.91	429,000 (421,000 to 436,000)	5.15 (5.07 to 5.24)	-20,500 (-27,800 to -13,200)	-0.246 (-0.335 to -0.158)	0.95 (0.94 to 0.97)
Germany	2021	Non-elective surgeries	408,000	4.91	425,000 (415,000 to 435,000)	5.10 (4.98 to 5.23)	-16,400 (-26,800 to -6,340)	-0.198 (-0.322 to -0.076)	0.96 (0.94 to 0.98)
Germany	2020	Appendectomy	114,000	1.37	117,000 (113,000 to 122,000)	1.41 (1.36 to 1.46)	-3,600 (-7,760 to 503)	-0.043 (-0.093 to 0.006)	0.97 (0.94 to 1.00)
Germany	2021	Appendectomy	112,000	1.34	115,000 (110,000 to 121,000)	1.38 (1.32 to 1.46)	-3,440 (-9,220 to 2,160)	-0.041 (-0.111 to 0.026)	0.97 (0.92 to 1.02)

Germany	2020	Cholecystectomy	191,000	2.3	202,000 (196,000 to 208,000)	2.43 (2.36 to 2.50)	-11,200 (-17,200 to -5,420)	-0.135 (-0.207 to -0.065)	0.94 (0.92 to 0.97)
Germany	2021	Cholecystectomy	191,000	2.29	202,000 (196,000 to 208,000)	2.43 (2.36 to 2.50)	-11,400 (-17,300 to -5,620)	-0.137 (-0.207 to -0.068)	0.94 (0.92 to 0.97)
Germany	2020	Total mastectomy	26,800	0.323	27,600 (26,500 to 28,800)	0.332 (0.319 to 0.346)	-762 (-1,910 to 360)	-0.009 (-0.023 to 0.004)	0.97 (0.93 to 1.01)
Germany	2021	Total mastectomy	27,700	0.333	27,600 (26,100 to 29,300)	0.332 (0.313 to 0.352)	96 (-1,570 to 1,650)	0.001 (-0.019 to 0.020)	1.00 (0.95 to 1.06)
Germany	2020	Partial excision of mammary gland	76,500	0.92	81,700 (78,000 to 85,500)	0.982 (0.938 to 1.03)	-5,160 (-8,980 to -1,420)	-0.062 (-0.108 to -0.017)	0.94 (0.89 to 0.98)
Germany	2021	Partial excision of mammary gland	78,000	0.937	81,700 (76,600 to 87,200)	0.982 (0.920 to 1.05)	-3,740 (-9,230 to 1,480)	-0.045 (-0.111 to 0.018)	0.95 (0.89 to 1.02)
Germany	2020	Both	742,000	8.92	811,000 (788,000 to 835,000)	9.75 (9.47 to 10.0)	-69,300 (-93,200 to -46,100)	-0.834 (-1.12 to -0.555)	0.91 (0.89 to 0.94)
Germany	2021	Both	754,000	9.06	821,000 (787,000 to 855,000)	9.86 (9.46 to 10.3)	-66,800 (-101,000 to -33,600)	-0.804 (-1.22 to -0.404)	0.92 (0.88 to 0.96)
Germany	2020	Caesarean section	234,000	2.81	231,000 (219,000 to 242,000)	2.77 (2.64 to 2.92)	3,420 (-8,590 to 14,900)	0.041 (-0.103 to 0.179)	1.01 (0.96 to 1.07)
Germany	2021	Caesarean section	244,000	2.93	231,000 (215,000 to 248,000)	2.77 (2.58 to 2.98)	13,500 (-3,110 to 29,700)	0.162 (-0.037 to 0.356)	1.06 (0.99 to 1.14)
Germany	2020	Repair of inguinal hernia	149,000	1.79	177,000 (172,000 to 183,000)	2.13 (2.06 to 2.20)	-28,000 (-34,000 to -22,300)	-0.336 (-0.409 to -0.268)	0.84 (0.81 to 0.87)
Germany	2021	Repair of inguinal hernia	148,000	1.78	177,000 (169,000 to 186,000)	2.13 (2.03 to 2.23)	-29,400 (-37,700 to -21,300)	-0.353 (-0.453 to -0.256)	0.83 (0.80 to 0.87)
Germany	2020	Transluminal coronary angioplasty	320,000	3.84	357,000 (345,000 to 370,000)	4.30 (4.15 to 4.45)	-37,600 (-50,600 to -25,400)	-0.452 (-0.608 to -0.305)	0.89 (0.86 to 0.93)
Germany	2021	Transluminal coronary angioplasty	324,000	3.9	366,000 (348,000 to 385,000)	4.40 (4.19 to 4.62)	-41,600 (-60,300 to -23,100)	-0.500 (-0.725 to -0.278)	0.89 (0.84 to 0.93)
Germany	2020	Coronary artery bypass graft	38,900	0.467	44,300 (42,200 to 46,500)	0.533 (0.507 to 0.560)	-5,450 (-7,680 to -3,340)	-0.066 (-0.092 to -0.040)	0.88 (0.84 to 0.92)
Germany	2021	Coronary artery bypass graft	37,200	0.447	43,200 (40,300 to 46,300)	0.520 (0.485 to 0.557)	-6,050 (-9,220 to -3,170)	-0.073 (-0.111 to -0.038)	0.86 (0.80 to 0.92)
Germany	2020	Elective surgeries	1,520,000	18.3	1,610,000 (1,580,000 to 1,650,000)	19.4 (19.0 to 19.8)	-92,100 (-127,000 to -57,600)	-1.11 (-1.53 to -0.693)	0.94 (0.92 to 0.96)
Germany	2021	Elective surgeries	1,600,000	19.2	1,610,000 (1,580,000 to 1,650,000)	19.4 (19.0 to 19.8)	-15,200 (-48,400 to 18,700)	-0.183 (-0.582 to 0.224)	0.99 (0.97 to 1.01)
Germany	2020	Hip replacement	245,000	2.94	265,000 (257,000 to 273,000)	3.19 (3.09 to 3.28)	-20,300 (-28,600 to -12,400)	-0.245 (-0.344 to -0.149)	0.92 (0.90 to 0.95)

Germany	2021	Hip replacement	250,000	3.01	268,000 (257,000 to 280,000)	3.22 (3.09 to 3.37)	-17,800 (-29,600 to -6,420)	-0.214 (-0.356 to -0.077)	0.93 (0.89 to 0.97)
Germany	2020	Total knee replacement	169,000	2.03	189,000 (175,000 to 205,000)	2.27 (2.10 to 2.46)	-20,000 (-35,600 to -5,350)	-0.241 (-0.428 to -0.064)	0.89 (0.83 to 0.97)
Germany	2021	Total knee replacement	167,000	2.01	189,000 (169,000 to 212,000)	2.27 (2.03 to 2.54)	-21,900 (-44,300 to -2,030)	-0.263 (-0.533 to -0.024)	0.88 (0.79 to 0.99)
Germany	2020	Cataract surgery	859,000	10.3	898,000 (873,000 to 924,000)	10.8 (10.5 to 11.1)	-39,700 (-65,000 to -14,800)	-0.478 (-0.781 to -0.178)	0.96 (0.93 to 0.98)
Germany	2021	Cataract surgery	946,000	11.4	914,000 (879,000 to 951,000)	11.0 (10.6 to 11.4)	31,500 (-5,330 to 67,600)	0.379 (-0.064 to 0.812)	1.03 (0.99 to 1.08)
Germany	2020	Tonsillectomy	64,000	0.77	83,900 (76,700 to 91,800)	1.01 (0.922 to 1.10)	-19,900 (-27,900 to -12,800)	-0.239 (-0.335 to -0.154)	0.76 (0.70 to 0.83)
Germany	2021	Tonsillectomy	48,100	0.578	78,000 (68,700 to 88,600)	0.937 (0.825 to 1.06)	-29,900 (-40,500 to -20,500)	-0.360 (-0.487 to -0.246)	0.62 (0.54 to 0.70)
Germany	2020	Hysterectomy	87,700	1.05	97,000 (92,200 to 102,000)	1.17 (1.11 to 1.23)	-9,350 (-14,300 to -4,680)	-0.112 (-0.172 to -0.056)	0.90 (0.86 to 0.95)
Germany	2021	Hysterectomy	87,200	1.05	93,200 (86,800 to 100,000)	1.12 (1.04 to 1.20)	-5,970 (-12,900 to 492)	-0.072 (-0.155 to 0.006)	0.94 (0.87 to 1.01)
Germany	2020	Open prostatectomy	30,300	0.365	33,000 (30,800 to 35,200)	0.396 (0.371 to 0.424)	-2,620 (-4,840 to -529)	-0.032 (-0.058 to -0.006)	0.92 (0.86 to 0.98)
Germany	2021	Open prostatectomy	31,100	0.374	32,200 (28,700 to 36,100)	0.387 (0.344 to 0.434)	-1,040 (-4,950 to 2,480)	-0.013 (-0.059 to 0.030)	0.97 (0.86 to 1.09)
Germany	2020	Transurethral prostatectomy	66,100	0.795	74,900 (71,500 to 78,400)	0.901 (0.860 to 0.943)	-8,790 (-12,400 to -5,340)	-0.106 (-0.149 to -0.064)	0.88 (0.84 to 0.93)
Germany	2021	Transurethral prostatectomy	68,200	0.82	75,100 (70,400 to 80,100)	0.903 (0.846 to 0.963)	-6,860 (-11,800 to -2,190)	-0.082 (-0.142 to -0.026)	0.91 (0.85 to 0.97)
Denmark	2020	All surgeries	148,000	25.5	157,000 (154,000 to 161,000)	27.0 (26.4 to 27.6)	-9,020 (-12,700 to -5,490)	-1.55 (-2.17 to -0.941)	0.94 (0.92 to 0.96)
Denmark	2021	All surgeries	139,000	23.7	158,000 (153,000 to 163,000)	27.0 (26.1 to 27.9)	-19,100 (-24,300 to -14,000)	-3.26 (-4.15 to -2.39)	0.88 (0.85 to 0.91)
Denmark	2020	Non-elective surgeries	23,800	4.08	24,700 (23,700 to 25,700)	4.24 (4.07 to 4.41)	-930 (-1,940 to 55)	-0.159 (-0.333 to 0.009)	0.96 (0.92 to 1.00)
Denmark	2021	Non-elective surgeries	23,600	4.02	24,800 (23,500 to 26,200)	4.24 (4.00 to 4.48)	-1,250 (-2,670 to 112)	-0.213 (-0.456 to 0.019)	0.95 (0.90 to 1.00)
Denmark	2020	Appendectomy	7,540	1.29	7,530 (6,950 to 8,170)	1.29 (1.19 to 1.40)	10 (-633 to 597)	0.002 (-0.109 to 0.102)	1.00 (0.92 to 1.09)
Denmark	2021	Appendectomy	7,550	1.29	7,570 (6,750 to 8,480)	1.29 (1.15 to 1.45)	-20 (-937 to 772)	-0.003 (-0.160 to 0.132)	1.00 (0.89 to 1.11)
Denmark	2020	Cholecystectomy	8,220	1.41	8,720 (8,190 to 9,290)	1.50 (1.40 to 1.59)	-497 (-1,060 to 39)	-0.085 (-0.182 to 0.007)	0.94 (0.89 to 1.00)
Denmark	2021	Cholecystectomy	8,130	1.39	8,760 (8,010 to 9,580)	1.50 (1.37 to 1.64)	-628 (-1,450 to 132)	-0.107 (-0.247 to 0.022)	0.93 (0.85 to 1.02)
Denmark	2020	Total mastectomy	2,560	0.439	2,720 (2,520 to 2,930)	0.466 (0.432 to 0.502)	-153 (-360 to 41)	-0.026 (-0.062 to 0.007)	0.94 (0.88 to 1.02)

Denmark	2021	Total mastectomy	2,300	0.392	2,730 (2,530 to 2,940)	0.466 (0.432 to 0.502)	-430 (-641 to -239)	-0.073 (-0.110 to -0.041)	0.84 (0.78 to 0.91)
Denmark	2020	Partial excision of mammary gland	5,440	0.934	5,830 (5,390 to 6,300)	0.999 (0.924 to 1.08)	-383 (-855 to 58)	-0.066 (-0.147 to 0.010)	0.93 (0.86 to 1.01)
Denmark	2021	Partial excision of mammary gland	5,590	0.954	5,850 (5,240 to 6,540)	0.999 (0.894 to 1.12)	-265 (-945 to 350)	-0.045 (-0.161 to 0.060)	0.95 (0.86 to 1.07)
Denmark	2020	Both	35,600	6.1	36,500 (34,400 to 38,800)	6.26 (5.90 to 6.66)	-954 (-3,180 to 1,190)	-0.164 (-0.544 to 0.203)	0.97 (0.92 to 1.03)
Denmark	2021	Both	34,300	5.85	36,700 (33,700 to 40,000)	6.26 (5.75 to 6.82)	-2,390 (-5,700 to 632)	-0.409 (-0.973 to 0.108)	0.93 (0.86 to 1.02)
Denmark	2020	Caesarean section	12,400	2.12	12,300 (11,500 to 13,200)	2.11 (1.97 to 2.26)	73 (-800 to 896)	0.013 (-0.137 to 0.154)	1.01 (0.94 to 1.08)
Denmark	2021	Caesarean section	12,500	2.13	12,400 (11,200 to 13,600)	2.11 (1.92 to 2.33)	97 (-1,130 to 1,210)	0.017 (-0.193 to 0.207)	1.01 (0.92 to 1.11)
Denmark	2020	Repair of inguinal hernia	9,570	1.64	10,200 (9,340 to 11,100)	1.74 (1.60 to 1.90)	-603 (-1,500 to 230)	-0.103 (-0.258 to 0.039)	0.94 (0.86 to 1.02)
Denmark	2021	Repair of inguinal hernia	9,010	1.54	10,200 (9,050 to 11,500)	1.74 (1.55 to 1.97)	-1,210 (-2,520 to -43)	-0.207 (-0.430 to -0.007)	0.88 (0.78 to 1.00)
Denmark	2020	Transluminal coronary angioplasty	10,400	1.77	11,000 (10,300 to 11,900)	1.89 (1.76 to 2.03)	-681 (-1,530 to 82)	-0.117 (-0.262 to 0.014)	0.94 (0.87 to 1.01)
Denmark	2021	Transluminal coronary angioplasty	9,890	1.69	11,100 (10,000 to 12,300)	1.89 (1.71 to 2.09)	-1,190 (-2,340 to -132)	-0.204 (-0.399 to -0.023)	0.89 (0.81 to 0.99)
Denmark	2020	Coronary artery bypass graft	3,260	0.559	2,890 (2,560 to 3,260)	0.495 (0.438 to 0.559)	376 (3 to 698)	0.065 (0.001 to 0.120)	1.13 (1.00 to 1.27)
Denmark	2021	Coronary artery bypass graft	2,930	0.501	2,780 (2,340 to 3,300)	0.475 (0.400 to 0.564)	151 (-389 to 585)	0.026 (-0.066 to 0.100)	1.05 (0.88 to 1.25)
Denmark	2020	Elective surgeries	89,100	15.3	96,200 (92,400 to 100,000)	16.5 (15.8 to 17.2)	-7,140 (-11,100 to -3,200)	-1.22 (-1.90 to -0.549)	0.93 (0.89 to 0.97)
Denmark	2021	Elective surgeries	81,200	13.9	96,600 (91,300 to 102,000)	16.5 (15.6 to 17.5)	-15,400 (-21,200 to -10,000)	-2.63 (-3.62 to -1.71)	0.84 (0.79 to 0.89)
Denmark	2020	Hip replacement	14,600	2.5	15,300 (14,700 to 15,900)	2.62 (2.53 to 2.72)	-689 (-1,250 to -142)	-0.118 (-0.214 to -0.024)	0.95 (0.92 to 0.99)
Denmark	2021	Hip replacement	13,800	2.36	15,600 (14,800 to 16,400)	2.66 (2.53 to 2.80)	-1,760 (-2,580 to -978)	-0.301 (-0.441 to -0.167)	0.89 (0.84 to 0.93)
Denmark	2020	Total knee replacement	11,400	1.95	10,100 (8,910 to 11,500)	1.74 (1.53 to 1.98)	1,220 (-162 to 2,450)	0.210 (-0.028 to 0.419)	1.12 (0.99 to 1.27)
Denmark	2021	Total knee replacement	10,200	1.73	10,200 (8,950 to 11,600)	1.74 (1.53 to 1.98)	-19 (-1,410 to 1,200)	-0.003 (-0.241 to 0.205)	1.00 (0.88 to 1.13)
Denmark	2020	Cataract surgery	55,100	9.45	60,600 (56,700 to 64,800)	10.4 (9.73 to 11.1)	-5,550 (-9,660 to -1,700)	-0.952 (-1.66 to -0.291)	0.91 (0.85 to 0.97)
Denmark	2021	Cataract surgery	49,300	8.42	60,900 (55,400 to 66,900)	10.4 (9.47 to 11.4)	-11,600 (-17,600 to -6,110)	-1.98 (-3.01 to -1.04)	0.81 (0.74 to 0.89)
Denmark	2020	Tonsillectomy	3,610	0.62	3,860 (3,490 to 4,270)	0.662 (0.599 to 0.732)	-246 (-651 to 122)	-0.042 (-0.112 to 0.021)	0.94 (0.85 to 1.03)
Denmark	2021	Tonsillectomy	3,360	0.574	3,660 (3,180 to 4,220)	0.626 (0.543 to 0.721)	-301 (-860 to 185)	-0.051 (-0.147 to 0.032)	0.92 (0.80 to 1.06)
Denmark	2020	Open prostatectomy	1,200	0.205	1,160 (1,010 to 1,340)	0.200 (0.174 to 0.229)	32 (-142 to 181)	0.005 (-0.024 to 0.031)	1.03 (0.89 to 1.18)
Denmark	2021	Open prostatectomy	1,320	0.225	1,170 (1,020 to 1,340)	0.200 (0.174 to 0.229)	151 (-19 to 301)	0.026 (-0.003 to 0.051)	1.13 (0.99 to 1.30)

Denmark	2020	Transurethral prostatectomy	3,230	0.554	3,500 (3,000 to 4,090)	0.600 (0.514 to 0.702)	-269 (-868 to 238)	-0.046 (-0.149 to 0.041)	0.92 (0.79 to 1.08)
Denmark	2021	Transurethral prostatectomy	3,250	0.554	3,520 (3,010 to 4,110)	0.600 (0.514 to 0.702)	-271 (-851 to 243)	-0.046 (-0.145 to 0.041)	0.92 (0.79 to 1.08)
Spain	2020	All surgeries	934,000	19.7	1,150,000 (1,070,000 to 1,230,000)	24.2 (22.5 to 26.1)	-214,000 (-301,000 to -133,000)	-4.52 (-6.37 to -2.81)	0.81 (0.76 to 0.88)
Spain	2021	All surgeries	1,060,000	22.3	1,150,000 (1,040,000 to 1,270,000)	24.2 (21.9 to 26.9)	-91,700 (-219,000 to 19,100)	-1.93 (-4.61 to 0.403)	0.92 (0.83 to 1.02)
Spain	2020	Non-elective surgeries	170,000	3.58	205,000 (193,000 to 218,000)	4.33 (4.07 to 4.60)	-35,500 (-48,400 to -23,400)	-0.749 (-1.02 to -0.495)	0.83 (0.78 to 0.88)
Spain	2021	Non-elective surgeries	201,000	4.23	205,000 (188,000 to 224,000)	4.33 (3.97 to 4.72)	-4,540 (-23,300 to 12,700)	-0.096 (-0.492 to 0.268)	0.98 (0.90 to 1.07)
Spain	2020	Appendectomy	49,900	1.05	51,700 (49,000 to 54,500)	1.09 (1.04 to 1.15)	-1,830 (-4,640 to 785)	-0.039 (-0.098 to 0.017)	0.96 (0.91 to 1.02)
Spain	2021	Appendectomy	54,200	1.14	51,800 (49,100 to 54,600)	1.09 (1.04 to 1.15)	2,470 (-344 to 5,210)	0.052 (-0.007 to 0.110)	1.05 (0.99 to 1.11)
Spain	2020	Cholecystectomy	67,500	1.42	80,600 (75,400 to 86,100)	1.70 (1.59 to 1.82)	-13,100 (-18,500 to -7,740)	-0.276 (-0.391 to -0.164)	0.84 (0.78 to 0.90)
Spain	2021	Cholecystectomy	83,400	1.76	80,600 (75,500 to 86,200)	1.70 (1.59 to 1.82)	2,710 (-2,880 to 7,940)	0.057 (-0.061 to 0.168)	1.03 (0.97 to 1.11)
Spain	2020	Total mastectomy	11,500	0.242	21,400 (17,500 to 26,100)	0.451 (0.370 to 0.550)	-9,920 (-14,700 to -6,000)	-0.209 (-0.310 to -0.127)	0.54 (0.44 to 0.66)
Spain	2021	Total mastectomy	13,200	0.279	21,400 (16,200 to 28,300)	0.451 (0.341 to 0.598)	-8,180 (-15,200 to -2,810)	-0.172 (-0.320 to -0.059)	0.62 (0.47 to 0.82)
Spain	2020	Partial excision of mammary gland	40,800	0.861	50,000 (45,500 to 55,000)	1.06 (0.961 to 1.16)	-9,250 (-14,100 to -4,690)	-0.195 (-0.299 to -0.099)	0.82 (0.74 to 0.90)
Spain	2021	Partial excision of mammary gland	50,000	1.05	52,000 (45,500 to 59,400)	1.10 (0.959 to 1.25)	-2,020 (-9,350 to 4,410)	-0.043 (-0.197 to 0.093)	0.96 (0.84 to 1.10)
Spain	2020	Both	223,000	4.72	260,000 (249,000 to 271,000)	5.49 (5.26 to 5.73)	-36,400 (-48,000 to -25,500)	-0.768 (-1.01 to -0.539)	0.86 (0.82 to 0.90)
Spain	2021	Both	240,000	5.05	260,000 (245,000 to 276,000)	5.49 (5.16 to 5.83)	-20,600 (-36,800 to -5,650)	-0.434 (-0.776 to -0.119)	0.92 (0.87 to 0.98)
Spain	2020	Caesarean section	82,400	1.74	84,500 (81,300 to 87,800)	1.78 (1.72 to 1.85)	-2,110 (-5,420 to 1,190)	-0.045 (-0.115 to 0.025)	0.98 (0.94 to 1.01)
Spain	2021	Caesarean section	81,600	1.72	81,400 (77,100 to 86,000)	1.72 (1.63 to 1.81)	122 (-4,560 to 4,480)	0.003 (-0.096 to 0.095)	1.00 (0.95 to 1.06)
Spain	2020	Repair of inguinal hernia	80,600	1.7	103,000 (95,100 to 113,000)	2.18 (2.01 to 2.38)	-22,900 (-32,100 to -14,400)	-0.483 (-0.677 to -0.304)	0.78 (0.72 to 0.85)
Spain	2021	Repair of inguinal hernia	92,400	1.95	104,000 (91,900 to 117,000)	2.18 (1.94 to 2.46)	-11,200 (-24,300 to 390)	-0.235 (-0.513 to 0.008)	0.89 (0.79 to 1.00)
Spain	2020	Transluminal coronary angioplasty	54,000	1.14	57,400 (52,400 to 62,800)	1.21 (1.11 to 1.33)	-3,340 (-8,520 to 1,580)	-0.071 (-0.180 to 0.033)	0.94 (0.86 to 1.03)

Spain	2021	Transluminal coronary angioplasty	58,700	1.24	57,400 (52,500 to 62,800)	1.21 (1.11 to 1.33)	1,270 (-3,980 to 6,160)	0.027 (-0.084 to 0.130)	1.02 (0.94 to 1.12)
Spain	2020	Coronary artery bypass graft	6,480	0.137	7,740 (7,210 to 8,320)	0.164 (0.152 to 0.176)	-1,270 (-1,860 to -732)	-0.027 (-0.039 to -0.015)	0.84 (0.78 to 0.90)
Spain	2021	Coronary artery bypass graft	6,860	0.145	7,750 (7,000 to 8,580)	0.164 (0.148 to 0.181)	-889 (-1,720 to -133)	-0.019 (-0.036 to -0.003)	0.89 (0.80 to 0.98)
Spain	2020	Elective surgeries	541,000	11.4	703,000 (644,000 to 767,000)	14.8 (13.6 to 16.2)	-162,000 (-227,000 to -103,000)	-3.43 (-4.79 to -2.18)	0.77 (0.70 to 0.84)
Spain	2021	Elective surgeries	617,000	13	724,000 (640,000 to 819,000)	15.3 (13.5 to 17.3)	-107,000 (-201,000 to -21,400)	-2.26 (-4.24 to -0.452)	0.85 (0.75 to 0.97)
Spain	2020	Hip replacement	51,400	1.08	62,600 (59,900 to 65,500)	1.32 (1.26 to 1.38)	-11,300 (-14,100 to -8,470)	-0.238 (-0.298 to -0.179)	0.82 (0.78 to 0.86)
Spain	2021	Hip replacement	61,200	1.29	64,600 (60,600 to 68,800)	1.36 (1.28 to 1.45)	-3,360 (-7,600 to 597)	-0.071 (-0.160 to 0.013)	0.95 (0.89 to 1.01)
Spain	2020	Total knee replacement	45,800	0.966	71,200 (69,300 to 73,200)	1.50 (1.46 to 1.54)	-25,500 (-27,400 to -23,600)	-0.538 (-0.579 to -0.498)	0.64 (0.63 to 0.66)
Spain	2021	Total knee replacement	57,300	1.21	74,200 (72,100 to 76,400)	1.56 (1.52 to 1.61)	-16,900 (-19,000 to -14,800)	-0.356 (-0.401 to -0.312)	0.77 (0.75 to 0.79)
Spain	2020	Cataract surgery	361,000	7.62	452,000 (401,000 to 510,000)	9.55 (8.47 to 10.8)	-91,600 (-150,000 to -40,000)	-1.93 (-3.16 to -0.845)	0.80 (0.71 to 0.90)
Spain	2021	Cataract surgery	405,000	8.54	453,000 (382,000 to 537,000)	9.55 (8.06 to 11.3)	-48,200 (-132,000 to 23,100)	-1.02 (-2.79 to 0.487)	0.89 (0.75 to 1.06)
Spain	2020	Tonsillectomy	17,500	0.369	29,400 (26,700 to 32,300)	0.620 (0.563 to 0.683)	-11,900 (-14,800 to -9,210)	-0.251 (-0.313 to -0.194)	0.59 (0.54 to 0.65)
Spain	2021	Tonsillectomy	16,500	0.348	29,400 (26,700 to 32,400)	0.620 (0.563 to 0.683)	-12,900 (-15,900 to -10,200)	-0.272 (-0.335 to -0.215)	0.56 (0.51 to 0.62)
Spain	2020	Hysterectomy	29,300	0.619	34,500 (31,500 to 37,900)	0.729 (0.665 to 0.800)	-5,220 (-8,600 to -2,130)	-0.110 (-0.181 to -0.045)	0.85 (0.77 to 0.93)
Spain	2021	Hysterectomy	34,100	0.72	34,600 (30,300 to 39,400)	0.729 (0.640 to 0.831)	-437 (-5,320 to 3,860)	-0.009 (-0.112 to 0.081)	0.99 (0.87 to 1.13)
Spain	2020	Open prostatectomy	15,900	0.336	17,600 (16,000 to 19,400)	0.372 (0.337 to 0.410)	-1,700 (-3,510 to -56)	-0.036 (-0.074 to -0.001)	0.90 (0.82 to 1.00)
Spain	2021	Open prostatectomy	17,600	0.372	17,300 (15,400 to 19,400)	0.364 (0.324 to 0.410)	332 (-1,790 to 2,240)	0.007 (-0.038 to 0.047)	1.02 (0.91 to 1.15)
Spain	2020	Transurethral prostatectomy	19,800	0.418	24,700 (22,700 to 26,800)	0.521 (0.480 to 0.565)	-4,870 (-6,920 to -2,970)	-0.103 (-0.146 to -0.063)	0.80 (0.74 to 0.87)
Spain	2021	Transurethral prostatectomy	25,400	0.537	25,500 (22,700 to 28,600)	0.538 (0.480 to 0.604)	-75 (-3,190 to 2,730)	-0.002 (-0.067 to 0.058)	1.00 (0.89 to 1.12)
Estonia	2020	All surgeries	40,100	30.2	43,700 (38,000 to 50,200)	32.9 (28.6 to 37.7)	-3,550 (-10,100 to 2,070)	-2.67 (-7.56 to 1.56)	0.92 (0.80 to 1.05)
Estonia	2021	All surgeries	39,200	29.5	43,700 (36,000 to 53,200)	32.9 (27.0 to 39.9)	-4,510 (-14,100 to 3,360)	-3.39 (-10.6 to 2.53)	0.90 (0.74 to 1.09)
Estonia	2020	Non-elective surgeries	5,410	4.07	5,960 (5,610 to 6,340)	4.49 (4.22 to 4.77)	-551 (-918 to -206)	-0.414 (-0.690 to -0.155)	0.91 (0.86 to 0.96)
Estonia	2021	Non-elective surgeries	5,390	4.05	5,970 (5,480 to 6,500)	4.49 (4.12 to 4.89)	-582 (-1,120 to -87)	-0.437 (-0.841 to -0.065)	0.90 (0.83 to 0.98)
Estonia	2020	Appendectomy	2,030	1.52	2,080 (1,960 to 2,200)	1.56 (1.48 to 1.66)	-52 (-175 to 68)	-0.039 (-0.132 to 0.051)	0.97 (0.92 to 1.03)

Estonia	2021	Appendectomy	2,040	1.53	2,080 (1,920 to 2,260)	1.56 (1.44 to 1.70)	-39 (-217 to 125)	-0.029 (-0.163 to 0.094)	0.98 (0.90 to 1.07)
Estonia	2020	Cholecystectomy	2,470	1.86	2,850 (2,540 to 3,200)	2.14 (1.91 to 2.41)	-376 (-725 to -56)	-0.282 (-0.546 to -0.042)	0.87 (0.77 to 0.98)
Estonia	2021	Cholecystectomy	2,350	1.77	2,850 (2,420 to 3,360)	2.14 (1.82 to 2.52)	-499 (-1,020 to -71)	-0.375 (-0.764 to -0.053)	0.83 (0.70 to 0.97)
Estonia	2020	Total mastectomy	272	0.205	349 (312 to 391)	0.263 (0.235 to 0.294)	-77 (-120 to -40)	-0.058 (-0.090 to -0.030)	0.78 (0.69 to 0.87)
Estonia	2021	Total mastectomy	299	0.225	349 (312 to 391)	0.263 (0.235 to 0.294)	-50 (-92 to -13)	-0.038 (-0.069 to -0.010)	0.86 (0.76 to 0.96)
Estonia	2020	Partial excision of mammary gland	641	0.482	734 (663 to 813)	0.552 (0.499 to 0.612)	-93 (-174 to -21)	-0.070 (-0.131 to -0.016)	0.87 (0.79 to 0.97)
Estonia	2021	Partial excision of mammary gland	694	0.521	765 (662 to 884)	0.575 (0.497 to 0.664)	-71 (-189 to 32)	-0.053 (-0.142 to 0.024)	0.91 (0.79 to 1.05)
Estonia	2020	Both	7,590	5.71	7,900 (7,550 to 8,270)	5.94 (5.68 to 6.22)	-316 (-689 to 32)	-0.238 (-0.518 to 0.024)	0.96 (0.92 to 1.00)
Estonia	2021	Both	7,600	5.71	7,910 (7,420 to 8,440)	5.94 (5.57 to 6.34)	-313 (-836 to 183)	-0.235 (-0.628 to 0.137)	0.96 (0.90 to 1.02)
Estonia	2020	Caesarean section	2,580	1.94	2,590 (2,350 to 2,850)	1.95 (1.76 to 2.14)	-1 (-267 to 240)	-0.001 (-0.201 to 0.180)	1.00 (0.91 to 1.10)
Estonia	2021	Caesarean section	2,660	1.99	2,590 (2,260 to 2,970)	1.95 (1.70 to 2.23)	66 (-318 to 400)	0.050 (-0.239 to 0.300)	1.03 (0.89 to 1.18)
Estonia	2020	Repair of inguinal hernia	1,820	1.37	1,910 (1,730 to 2,110)	1.44 (1.30 to 1.59)	-93 (-296 to 87)	-0.070 (-0.223 to 0.066)	0.95 (0.86 to 1.05)
Estonia	2021	Repair of inguinal hernia	1,770	1.33	1,910 (1,660 to 2,200)	1.44 (1.25 to 1.66)	-147 (-433 to 104)	-0.110 (-0.326 to 0.078)	0.92 (0.80 to 1.06)
Estonia	2020	Transluminal coronary angioplasty	2,770	2.08	2,890 (2,500 to 3,330)	2.17 (1.88 to 2.50)	-122 (-571 to 261)	-0.092 (-0.429 to 0.196)	0.96 (0.83 to 1.10)
Estonia	2021	Transluminal coronary angioplasty	2,840	2.14	2,890 (2,510 to 3,330)	2.17 (1.88 to 2.50)	-47 (-490 to 330)	-0.036 (-0.368 to 0.248)	0.98 (0.85 to 1.13)
Estonia	2020	Coronary artery bypass graft	416	0.313	480 (368 to 626)	0.361 (0.277 to 0.471)	-64 (-211 to 49)	-0.048 (-0.159 to 0.037)	0.87 (0.66 to 1.13)
Estonia	2021	Coronary artery bypass graft	332	0.249	480 (330 to 699)	0.361 (0.248 to 0.525)	-148 (-364 to 5)	-0.112 (-0.274 to 0.003)	0.69 (0.48 to 1.01)
Estonia	2020	Elective surgeries	27,100	20.4	29,800 (24,100 to 36,900)	22.4 (18.1 to 27.7)	-2,680 (-9,640 to 3,010)	-2.02 (-7.25 to 2.26)	0.91 (0.74 to 1.12)
Estonia	2021	Elective surgeries	26,200	19.7	29,800 (22,100 to 40,300)	22.4 (16.6 to 30.3)	-3,610 (-14,000 to 4,300)	-2.71 (-10.6 to 3.23)	0.88 (0.65 to 1.20)
Estonia	2020	Hip replacement	2,290	1.72	2,320 (2,100 to 2,570)	1.75 (1.58 to 1.93)	-33 (-282 to 190)	-0.025 (-0.212 to 0.143)	0.99 (0.89 to 1.09)
Estonia	2021	Hip replacement	2,280	1.72	2,410 (2,080 to 2,780)	1.81 (1.57 to 2.09)	-121 (-492 to 198)	-0.091 (-0.370 to 0.149)	0.95 (0.82 to 1.09)
Estonia	2020	Total knee replacement	1,380	1.04	1,330 (1,110 to 1,600)	1.00 (0.834 to 1.20)	47 (-218 to 272)	0.036 (-0.164 to 0.204)	1.04 (0.86 to 1.25)
Estonia	2021	Total knee replacement	1,250	0.938	1,330 (1,110 to 1,600)	1.00 (0.834 to 1.20)	-83 (-345 to 145)	-0.062 (-0.259 to 0.109)	0.94 (0.78 to 1.13)
Estonia	2020	Cataract surgery	17,700	13.3	19,000 (15,900 to 22,900)	14.3 (11.9 to 17.2)	-1,390 (-5,230 to 1,720)	-1.05 (-3.93 to 1.29)	0.93 (0.77 to 1.11)
Estonia	2021	Cataract surgery	17,400	13.1	19,100 (14,700 to 24,700)	14.3 (11.1 to 18.5)	-1,630 (-7,250 to 2,610)	-1.22 (-5.45 to 1.96)	0.91 (0.71 to 1.18)

Estonia	2020	Tonsillectomy	3,450	2.6	4,240 (3,980 to 4,520)	3.19 (3.00 to 3.40)	-793 (-1,060 to -531)	-0.596 (-0.800 to -0.399)	0.81 (0.76 to 0.87)
Estonia	2021	Tonsillectomy	2,950	2.22	4,010 (3,670 to 4,390)	3.02 (2.76 to 3.30)	-1,060 (-1,430 to -717)	-0.800 (-1.07 to -0.539)	0.73 (0.67 to 0.80)
Estonia	2020	Hysterectomy	1,470	1.1	1,620 (1,460 to 1,790)	1.21 (1.10 to 1.35)	-146 (-316 to 8)	-0.110 (-0.238 to 0.006)	0.91 (0.82 to 1.01)
Estonia	2021	Hysterectomy	1,470	1.1	1,620 (1,400 to 1,870)	1.21 (1.05 to 1.40)	-147 (-407 to 71)	-0.110 (-0.306 to 0.053)	0.91 (0.78 to 1.05)
Estonia	2020	Open prostatectomy	471	0.354	479 (382 to 600)	0.360 (0.288 to 0.451)	-8 (-128 to 89)	-0.006 (-0.096 to 0.067)	0.98 (0.79 to 1.23)
Estonia	2021	Open prostatectomy	430	0.323	479 (349 to 659)	0.360 (0.262 to 0.495)	-49 (-231 to 81)	-0.037 (-0.173 to 0.061)	0.90 (0.65 to 1.23)
Estonia	2020	Transurethral prostatectomy	418	0.314	495 (414 to 592)	0.372 (0.311 to 0.446)	-77 (-175 to 5)	-0.058 (-0.131 to 0.004)	0.84 (0.71 to 1.01)
Estonia	2021	Transurethral prostatectomy	414	0.311	496 (414 to 593)	0.372 (0.311 to 0.446)	-82 (-178 to 0)	-0.061 (-0.134 to 0.000)	0.84 (0.70 to 1.00)
Finland	2020	All surgeries	164,000	29.6	176,000 (166,000 to 187,000)	31.9 (30.1 to 33.8)	-12,800 (-23,400 to -2,660)	-2.31 (-4.24 to -0.481)	0.93 (0.87 to 0.98)
Finland	2021	All surgeries	176,000	31.7	177,000 (163,000 to 192,000)	31.9 (29.3 to 34.6)	-825 (-16,000 to 13,200)	-0.149 (-2.89 to 2.39)	1.00 (0.92 to 1.08)
Finland	2020	Non-elective surgeries	24,500	4.43	25,700 (24,200 to 27,300)	4.65 (4.38 to 4.93)	-1,210 (-2,810 to 261)	-0.219 (-0.508 to 0.047)	0.95 (0.90 to 1.01)
Finland	2021	Non-elective surgeries	25,800	4.66	26,200 (24,100 to 28,500)	4.74 (4.36 to 5.15)	-445 (-2,770 to 1,650)	-0.080 (-0.500 to 0.299)	0.98 (0.90 to 1.07)
Finland	2020	Appendectomy	7,780	1.41	7,520 (6,880 to 8,220)	1.36 (1.25 to 1.49)	254 (-443 to 886)	0.046 (-0.080 to 0.160)	1.03 (0.95 to 1.13)
Finland	2021	Appendectomy	8,200	1.48	7,540 (6,650 to 8,550)	1.36 (1.20 to 1.54)	656 (-351 to 1,540)	0.118 (-0.063 to 0.277)	1.09 (0.96 to 1.23)
Finland	2020	Cholecystectomy	8,950	1.62	9,490 (8,780 to 10,300)	1.72 (1.59 to 1.85)	-538 (-1,300 to 185)	-0.097 (-0.234 to 0.033)	0.94 (0.87 to 1.02)
Finland	2021	Cholecystectomy	9,670	1.74	9,510 (8,530 to 10,600)	1.72 (1.54 to 1.91)	159 (-940 to 1,110)	0.029 (-0.170 to 0.200)	1.02 (0.91 to 1.13)
Finland	2020	Total mastectomy	2,350	0.426	2,500 (2,280 to 2,730)	0.452 (0.413 to 0.494)	-144 (-375 to 71)	-0.026 (-0.068 to 0.013)	0.94 (0.86 to 1.03)
Finland	2021	Total mastectomy	2,360	0.425	2,500 (2,200 to 2,840)	0.452 (0.398 to 0.513)	-146 (-492 to 163)	-0.026 (-0.089 to 0.029)	0.94 (0.83 to 1.07)
Finland	2020	Partial excision of mammary gland	5,390	0.975	5,950 (5,320 to 6,650)	1.08 (0.963 to 1.20)	-560 (-1,260 to 71)	-0.101 (-0.229 to 0.013)	0.91 (0.81 to 1.01)
Finland	2021	Partial excision of mammary gland	5,580	1.01	6,240 (5,330 to 7,310)	1.13 (0.962 to 1.32)	-663 (-1,740 to 224)	-0.120 (-0.314 to 0.041)	0.89 (0.76 to 1.04)
Finland	2020	Both	32,300	5.85	32,700 (30,200 to 35,300)	5.91 (5.47 to 6.39)	-360 (-3,020 to 2,110)	-0.065 (-0.546 to 0.382)	0.99 (0.91 to 1.07)
Finland	2021	Both	35,500	6.41	32,800 (30,300 to 35,400)	5.91 (5.47 to 6.39)	2,780 (166 to 5,200)	0.501 (0.030 to 0.939)	1.08 (1.00 to 1.17)
Finland	2020	Caesarean section	8,680	1.57	7,720 (7,410 to 8,050)	1.40 (1.34 to 1.46)	960 (638 to 1,260)	0.174 (0.115 to 0.228)	1.12 (1.08 to 1.17)
Finland	2021	Caesarean section	10,300	1.86	7,530 (7,100 to 7,980)	1.36 (1.28 to 1.44)	2,750 (2,300 to 3,200)	0.497 (0.416 to 0.577)	1.37 (1.29 to 1.45)
Finland	2020	Repair of inguinal hernia	9,520	1.72	11,100 (9,960 to 12,300)	2.00 (1.80 to 2.22)	-1,540 (-2,740 to -447)	-0.278 (-0.495 to -0.081)	0.86 (0.78 to 0.96)

Finland	2021	Repair of inguinal hernia	9,870	1.78	11,100 (9,980 to 12,300)	2.00 (1.80 to 2.22)	-1,220 (-2,440 to -106)	-0.220 (-0.440 to -0.019)	0.89 (0.80 to 0.99)
Finland	2020	Transluminal coronary angioplasty	12,600	2.28	13,000 (10,400 to 16,300)	2.36 (1.89 to 2.94)	-423 (-3,770 to 2,240)	-0.076 (-0.682 to 0.405)	0.97 (0.77 to 1.22)
Finland	2021	Transluminal coronary angioplasty	13,900	2.5	13,100 (9,540 to 17,900)	2.36 (1.72 to 3.23)	794 (-4,060 to 4,380)	0.143 (-0.732 to 0.791)	1.06 (0.77 to 1.46)
Finland	2020	Coronary artery bypass graft	1,520	0.276	1,590 (1,410 to 1,790)	0.287 (0.255 to 0.323)	-64 (-262 to 110)	-0.012 (-0.047 to 0.020)	0.96 (0.85 to 1.08)
Finland	2021	Coronary artery bypass graft	1,540	0.278	1,480 (1,250 to 1,750)	0.267 (0.226 to 0.316)	61 (-211 to 288)	0.011 (-0.038 to 0.052)	1.04 (0.88 to 1.23)
Finland	2020	Elective surgeries	107,000	19.3	125,000 (117,000 to 133,000)	22.6 (21.2 to 24.0)	-18,000 (-25,900 to -10,800)	-3.25 (-4.68 to -1.95)	0.86 (0.80 to 0.91)
Finland	2021	Elective surgeries	115,000	20.7	133,000 (116,000 to 152,000)	24.0 (20.9 to 27.5)	-18,300 (-37,400 to -1,690)	-3.30 (-6.75 to -0.305)	0.86 (0.75 to 0.99)
Finland	2020	Hip replacement	14,100	2.55	15,900 (15,200 to 16,500)	2.87 (2.76 to 2.98)	-1,730 (-2,370 to -1,120)	-0.313 (-0.429 to -0.202)	0.89 (0.86 to 0.93)
Finland	2021	Hip replacement	15,700	2.83	16,300 (15,400 to 17,200)	2.94 (2.77 to 3.11)	-563 (-1,510 to 347)	-0.102 (-0.273 to 0.063)	0.97 (0.91 to 1.02)
Finland	2020	Total knee replacement	12,500	2.26	13,400 (11,800 to 15,200)	2.42 (2.13 to 2.75)	-914 (-2,700 to 669)	-0.165 (-0.487 to 0.121)	0.93 (0.82 to 1.06)
Finland	2021	Total knee replacement	14,400	2.6	13,400 (11,200 to 16,000)	2.42 (2.03 to 2.89)	1,010 (-1,610 to 3,170)	0.182 (-0.290 to 0.571)	1.08 (0.90 to 1.28)
Finland	2020	Cataract surgery	64,100	11.6	74,800 (67,200 to 83,300)	13.5 (12.2 to 15.1)	-10,700 (-19,300 to -3,000)	-1.93 (-3.49 to -0.543)	0.86 (0.77 to 0.96)
Finland	2021	Cataract surgery	69,300	12.5	81,800 (64,300 to 104,000)	14.8 (11.6 to 18.8)	-12,500 (-35,200 to 4,990)	-2.25 (-6.36 to 0.900)	0.85 (0.66 to 1.08)
Finland	2020	Tonsillectomy	6,920	1.25	9,920 (8,640 to 11,400)	1.79 (1.56 to 2.06)	-3,000 (-4,490 to -1,740)	-0.543 (-0.813 to -0.316)	0.70 (0.61 to 0.80)
Finland	2021	Tonsillectomy	5,830	1.05	9,940 (8,170 to 12,100)	1.79 (1.47 to 2.18)	-4,110 (-6,180 to -2,380)	-0.742 (-1.12 to -0.429)	0.59 (0.49 to 0.71)
Finland	2020	Hysterectomy	4,400	0.795	4,770 (4,300 to 5,280)	0.862 (0.778 to 0.954)	-368 (-883 to 105)	-0.067 (-0.160 to 0.019)	0.92 (0.83 to 1.02)
Finland	2021	Hysterectomy	4,490	0.81	4,780 (4,140 to 5,520)	0.862 (0.746 to 0.996)	-288 (-1,030 to 348)	-0.052 (-0.186 to 0.063)	0.94 (0.81 to 1.08)
Finland	2020	Open prostatectomy	78	0.014	92 (71 to 120)	0.017 (0.013 to 0.022)	-14 (-42 to 7)	-0.003 (-0.008 to 0.001)	0.85 (0.65 to 1.09)
Finland	2021	Open prostatectomy	68	0.012	75 (52 to 110)	0.014 (0.009 to 0.020)	-7 (-42 to 16)	-0.001 (-0.008 to 0.003)	0.90 (0.62 to 1.30)
Finland	2020	Transurethral prostatectomy	4,620	0.835	5,090 (4,140 to 6,260)	0.921 (0.749 to 1.13)	-476 (-1,630 to 475)	-0.086 (-0.294 to 0.086)	0.91 (0.74 to 1.11)
Finland	2021	Transurethral prostatectomy	4,690	0.846	5,100 (3,810 to 6,830)	0.921 (0.688 to 1.23)	-415 (-2,120 to 855)	-0.075 (-0.383 to 0.154)	0.92 (0.69 to 1.22)
France	2020	All surgeries	1,930,000	28.5	2,280,000 (2,260,000 to 2,310,000)	33.8 (33.4 to 34.2)	-358,000 (-386,000 to -331,000)	-5.29 (-5.71 to -4.89)	0.84 (0.83 to 0.85)
France	2021	All surgeries	2,250,000	33.1	2,320,000 (2,280,000 to 2,360,000)	34.3 (33.7 to 34.9)	-78,300 (-119,000 to -37,500)	-1.16 (-1.76 to -0.554)	0.97 (0.95 to 0.98)

France	2020	Non-elective surgeries	276,000	4.08	290,000 (285,000 to 295,000)	4.29 (4.22 to 4.37)	-14,200 (-19,300 to -9,180)	-0.210 (-0.286 to -0.136)	0.95 (0.93 to 0.97)
France	2021	Non-elective surgeries	297,000	4.38	289,000 (282,000 to 296,000)	4.26 (4.16 to 4.37)	8,120 (880 to 15,200)	0.120 (0.013 to 0.224)	1.03 (1.00 to 1.05)
France	2020	Appendectomy	70,400	1.04	69,900 (66,900 to 72,900)	1.03 (0.991 to 1.08)	556 (-2,490 to 3,410)	0.008 (-0.037 to 0.050)	1.01 (0.97 to 1.05)
France	2021	Appendectomy	71,200	1.05	68,200 (64,200 to 72,400)	1.01 (0.948 to 1.07)	2,970 (-1,250 to 7,010)	0.044 (-0.018 to 0.103)	1.04 (0.98 to 1.11)
France	2020	Cholecystectomy	119,000	1.76	131,000 (127,000 to 135,000)	1.94 (1.89 to 2.00)	-12,000 (-15,900 to -8,320)	-0.177 (-0.235 to -0.123)	0.91 (0.88 to 0.93)
France	2021	Cholecystectomy	129,000	1.91	132,000 (128,000 to 135,000)	1.94 (1.89 to 2.00)	-2,380 (-6,280 to 1,350)	-0.035 (-0.093 to 0.020)	0.98 (0.95 to 1.01)
France	2020	Total mastectomy	20,800	0.307	22,000 (21,600 to 22,400)	0.326 (0.320 to 0.332)	-1,250 (-1,690 to -839)	-0.019 (-0.025 to -0.012)	0.94 (0.92 to 0.96)
France	2021	Total mastectomy	22,100	0.326	22,100 (21,700 to 22,500)	0.326 (0.320 to 0.332)	38 (-382 to 456)	0.001 (-0.006 to 0.007)	1.00 (0.98 to 1.02)
France	2020	Partial excision of mammary gland	65,700	0.972	70,500 (68,500 to 72,600)	1.04 (1.01 to 1.07)	-4,840 (-6,900 to -2,820)	-0.072 (-0.102 to -0.042)	0.93 (0.90 to 0.96)
France	2021	Partial excision of mammary gland	74,600	1.1	70,700 (67,900 to 73,700)	1.04 (1.00 to 1.09)	3,850 (954 to 6,670)	0.057 (0.014 to 0.098)	1.05 (1.01 to 1.10)
France	2020	Both	480,000	7.1	528,000 (522,000 to 534,000)	7.82 (7.73 to 7.90)	-48,500 (-54,400 to -42,500)	-0.718 (-0.805 to -0.629)	0.91 (0.90 to 0.92)
France	2021	Both	512,000	7.56	537,000 (524,000 to 551,000)	7.93 (7.73 to 8.13)	-25,200 (-38,700 to -12,100)	-0.372 (-0.571 to -0.179)	0.95 (0.93 to 0.98)
France	2020	Caesarean section	148,000	2.18	147,000 (144,000 to 150,000)	2.18 (2.13 to 2.22)	580 (-2,200 to 3,250)	0.009 (-0.032 to 0.048)	1.00 (0.99 to 1.02)
France	2021	Caesarean section	152,000	2.24	145,000 (141,000 to 149,000)	2.14 (2.08 to 2.19)	7,100 (3,240 to 11,000)	0.105 (0.048 to 0.162)	1.05 (1.02 to 1.08)
France	2020	Repair of inguinal hernia	133,000	1.96	158,000 (154,000 to 161,000)	2.33 (2.28 to 2.38)	-24,900 (-28,300 to -21,400)	-0.368 (-0.419 to -0.317)	0.84 (0.82 to 0.86)
France	2021	Repair of inguinal hernia	145,000	2.15	158,000 (154,000 to 162,000)	2.33 (2.28 to 2.38)	-12,500 (-16,000 to -9,050)	-0.184 (-0.236 to -0.133)	0.92 (0.90 to 0.94)
France	2020	Transluminal coronary angioplasty	183,000	2.71	203,000 (195,000 to 211,000)	3.00 (2.88 to 3.13)	-19,800 (-28,200 to -11,800)	-0.293 (-0.418 to -0.175)	0.90 (0.87 to 0.94)
France	2021	Transluminal coronary angioplasty	197,000	2.91	212,000 (200,000 to 224,000)	3.13 (2.96 to 3.31)	-14,500 (-27,200 to -2,850)	-0.215 (-0.402 to -0.042)	0.93 (0.88 to 0.99)
France	2020	Coronary artery bypass graft	16,400	0.243	19,400 (18,800 to 20,000)	0.287 (0.278 to 0.296)	-2,980 (-3,580 to -2,380)	-0.044 (-0.053 to -0.035)	0.85 (0.82 to 0.87)
France	2021	Coronary artery bypass graft	17,400	0.257	19,600 (18,800 to 20,400)	0.289 (0.278 to 0.301)	-2,160 (-2,990 to -1,390)	-0.032 (-0.044 to -0.021)	0.89 (0.85 to 0.93)
France	2020	Elective surgeries	1,170,000	17.3	1,470,000 (1,450,000 to 1,490,000)	21.8 (21.4 to 22.1)	-300,000 (-325,000 to -277,000)	-4.44 (-4.80 to -4.09)	0.80 (0.78 to 0.81)

France	2021	Elective surgeries	1,440,000	21.2	1,510,000 (1,470,000 to 1,540,000)	22.3 (21.8 to 22.8)	-72,400 (-107,000 to -38,100)	-1.07 (-1.58 to -0.563)	0.95 (0.93 to 0.97)
France	2020	Hip replacement	149,000	2.21	172,000 (170,000 to 174,000)	2.55 (2.51 to 2.58)	-22,900 (-25,100 to -20,700)	-0.338 (-0.371 to -0.306)	0.87 (0.86 to 0.88)
France	2021	Hip replacement	167,000	2.46	175,000 (172,000 to 178,000)	2.58 (2.53 to 2.62)	-8,120 (-11,300 to -4,980)	-0.120 (-0.167 to -0.073)	0.95 (0.94 to 0.97)
France	2020	Total knee replacement	97,800	1.45	131,000 (126,000 to 135,000)	1.93 (1.87 to 2.00)	-32,900 (-37,200 to -28,600)	-0.486 (-0.551 to -0.424)	0.75 (0.72 to 0.77)
France	2021	Total knee replacement	114,000	1.68	137,000 (131,000 to 144,000)	2.02 (1.93 to 2.12)	-23,300 (-29,900 to -17,100)	-0.344 (-0.442 to -0.253)	0.83 (0.79 to 0.87)
France	2020	Cataract surgery	749,000	11.1	955,000 (940,000 to 970,000)	14.1 (13.9 to 14.4)	-206,000 (-221,000 to -190,000)	-3.05 (-3.27 to -2.81)	0.78 (0.77 to 0.80)
France	2021	Cataract surgery	960,000	14.2	987,000 (965,000 to 1,010,000)	14.6 (14.2 to 14.9)	-27,400 (-50,000 to -5,670)	-0.404 (-0.738 to -0.084)	0.97 (0.95 to 0.99)
France	2020	Tonsillectomy	38,800	0.574	58,500 (51,600 to 66,400)	0.866 (0.764 to 0.982)	-19,700 (-27,600 to -12,900)	-0.292 (-0.409 to -0.191)	0.66 (0.58 to 0.75)
France	2021	Tonsillectomy	44,600	0.658	58,700 (49,100 to 70,100)	0.866 (0.725 to 1.03)	-14,100 (-25,500 to -4,680)	-0.208 (-0.376 to -0.069)	0.76 (0.64 to 0.90)
France	2020	Hysterectomy	51,000	0.754	58,600 (57,000 to 60,300)	0.868 (0.843 to 0.893)	-7,670 (-9,300 to -6,010)	-0.114 (-0.138 to -0.089)	0.87 (0.85 to 0.89)
France	2021	Hysterectomy	56,000	0.826	57,600 (55,300 to 60,000)	0.850 (0.817 to 0.885)	-1,610 (-3,960 to 684)	-0.024 (-0.058 to 0.010)	0.97 (0.93 to 1.01)
France	2020	Open prostatectomy	23,200	0.343	24,900 (21,900 to 28,300)	0.368 (0.324 to 0.419)	-1,700 (-5,100 to 1,300)	-0.025 (-0.075 to 0.019)	0.93 (0.82 to 1.06)
France	2021	Open prostatectomy	25,200	0.371	25,000 (20,800 to 30,000)	0.368 (0.307 to 0.442)	203 (-4,830 to 4,390)	0.003 (-0.071 to 0.065)	1.01 (0.84 to 1.21)
France	2020	Transurethral prostatectomy	61,400	0.908	75,300 (71,400 to 79,300)	1.11 (1.06 to 1.17)	-13,900 (-17,900 to -10,100)	-0.205 (-0.265 to -0.149)	0.82 (0.77 to 0.86)
France	2021	Transurethral prostatectomy	70,200	1.04	77,100 (71,600 to 83,100)	1.14 (1.06 to 1.23)	-6,950 (-12,900 to -1,370)	-0.103 (-0.190 to -0.020)	0.91 (0.84 to 0.98)
United Kingdom	2020	All surgeries	941,000	14	1,490,000 (1,430,000 to 1,550,000)	22.2 (21.3 to 23.1)	-547,000 (-611,000 to -486,000)	-8.16 (-9.11 to -7.24)	0.63 (0.61 to 0.66)
United Kingdom	2021	All surgeries	1,230,000	18.3	1,280,000 (1,190,000 to 1,380,000)	19.0 (17.7 to 20.4)	-52,300 (-146,000 to 34,500)	-0.776 (-2.17 to 0.512)	0.96 (0.89 to 1.03)
United Kingdom	2020	Non-elective surgeries	159,000	2.37	224,000 (212,000 to 237,000)	3.34 (3.16 to 3.53)	-65,600 (-78,200 to -53,500)	-0.978 (-1.17 to -0.797)	0.71 (0.67 to 0.75)
United Kingdom	2021	Non-elective surgeries	208,000	3.09	225,000 (213,000 to 238,000)	3.34 (3.16 to 3.53)	-17,300 (-30,200 to -5,400)	-0.257 (-0.448 to -0.080)	0.92 (0.87 to 0.97)
United Kingdom	2020	Appendectomy	48,500	0.723	55,500 (54,100 to 56,900)	0.827 (0.806 to 0.848)	-7,020 (-8,440 to -5,630)	-0.105 (-0.126 to -0.084)	0.87 (0.85 to 0.90)
United Kingdom	2021	Appendectomy	57,300	0.851	54,700 (52,800 to 56,700)	0.812 (0.784 to 0.842)	2,600 (569 to 4,540)	0.039 (0.008 to 0.067)	1.05 (1.01 to 1.09)

United Kingdom	2020	Cholecystectomy	54,100	0.807	90,300 (83,200 to 98,000)	1.35 (1.24 to 1.46)	-36,200 (-43,900 to -29,000)	-0.539 (-0.654 to -0.433)	0.60 (0.55 to 0.65)
United Kingdom	2021	Cholecystectomy	78,000	1.16	90,700 (83,500 to 98,400)	1.35 (1.24 to 1.46)	-12,600 (-20,400 to -5,400)	-0.187 (-0.303 to -0.080)	0.86 (0.79 to 0.94)
United Kingdom	2020	Total mastectomy	17,400	0.259	20,600 (19,400 to 21,800)	0.307 (0.290 to 0.325)	-3,200 (-4,400 to -2,080)	-0.048 (-0.066 to -0.031)	0.84 (0.80 to 0.89)
United Kingdom	2021	Total mastectomy	20,900	0.31	20,700 (19,000 to 22,400)	0.307 (0.283 to 0.332)	200 (-1,530 to 1,810)	0.003 (-0.023 to 0.027)	1.01 (0.93 to 1.09)
United Kingdom	2020	Partial excision of mammary gland	38,700	0.577	52,000 (50,200 to 53,800)	0.775 (0.749 to 0.802)	-13,300 (-15,100 to -11,500)	-0.198 (-0.225 to -0.172)	0.74 (0.72 to 0.77)
United Kingdom	2021	Partial excision of mammary gland	51,600	0.766	52,200 (50,400 to 54,000)	0.775 (0.749 to 0.802)	-602 (-2,420 to 1,150)	-0.009 (-0.036 to 0.017)	0.99 (0.96 to 1.02)
United Kingdom	2020	Both	331,000	4.94	386,000 (379,000 to 393,000)	5.75 (5.65 to 5.86)	-54,600 (-61,700 to -47,400)	-0.814 (-0.920 to -0.707)	0.86 (0.84 to 0.87)
United Kingdom	2021	Both	165,000	2.44	175,000 (164,000 to 188,000)	2.60 (2.43 to 2.79)	-10,600 (-23,100 to 1,190)	-0.157 (-0.344 to 0.018)	0.94 (0.88 to 1.01)
United Kingdom	2020	Caesarean section	214,000	3.18	211,000 (206,000 to 216,000)	3.15 (3.08 to 3.23)	2,170 (-2,910 to 7,160)	0.032 (-0.043 to 0.107)	1.01 (0.99 to 1.03)
United Kingdom	2020	Repair of inguinal hernia	35,500	0.529	76,000 (69,400 to 83,300)	1.13 (1.03 to 1.24)	-40,600 (-47,800 to -34,000)	-0.605 (-0.713 to -0.507)	0.47 (0.43 to 0.51)
United Kingdom	2021	Repair of inguinal hernia	62,300	0.925	76,300 (67,100 to 86,900)	1.13 (0.996 to 1.29)	-14,100 (-24,800 to -4,950)	-0.209 (-0.368 to -0.074)	0.82 (0.72 to 0.93)
United Kingdom	2020	Transluminal coronary angioplasty	72,900	1.09	83,300 (81,300 to 85,400)	1.24 (1.21 to 1.27)	-10,400 (-12,500 to -8,390)	-0.156 (-0.186 to -0.125)	0.87 (0.85 to 0.90)
United Kingdom	2021	Transluminal coronary angioplasty	88,800	1.32	83,400 (79,000 to 88,000)	1.24 (1.17 to 1.31)	5,450 (833 to 9,810)	0.081 (0.012 to 0.146)	1.07 (1.01 to 1.12)
United Kingdom	2020	Coronary artery bypass graft	9,450	0.141	14,400 (13,700 to 15,000)	0.214 (0.205 to 0.224)	-4,920 (-5,590 to -4,290)	-0.073 (-0.083 to -0.064)	0.66 (0.63 to 0.69)
United Kingdom	2021	Coronary artery bypass graft	13,600	0.201	13,900 (13,000 to 14,800)	0.207 (0.194 to 0.220)	-361 (-1,290 to 488)	-0.005 (-0.019 to 0.007)	0.97 (0.91 to 1.04)
United Kingdom	2020	Elective surgeries	451,000	6.72	890,000 (837,000 to 946,000)	13.3 (12.5 to 14.1)	-439,000 (-494,000 to -385,000)	-6.55 (-7.37 to -5.74)	0.51 (0.48 to 0.54)
United Kingdom	2021	Elective surgeries	857,000	12.7	894,000 (820,000 to 974,000)	13.3 (12.2 to 14.5)	-36,500 (-118,000 to 38,500)	-0.543 (-1.75 to 0.572)	0.96 (0.88 to 1.05)
United Kingdom	2020	Hip replacement	65,500	0.976	122,000 (116,000 to 129,000)	1.82 (1.73 to 1.92)	-56,900 (-63,400 to -50,600)	-0.848 (-0.946 to -0.755)	0.54 (0.51 to 0.56)
United Kingdom	2021	Hip replacement	121,000	1.8	123,000 (114,000 to 132,000)	1.82 (1.70 to 1.96)	-1,420 (-10,800 to 7,310)	-0.021 (-0.160 to 0.109)	0.99 (0.92 to 1.06)
United Kingdom	2020	Total knee replacement	30,800	0.459	95,400 (88,900 to 102,000)	1.42 (1.33 to 1.53)	-64,600 (-71,700 to -58,200)	-0.963 (-1.07 to -0.867)	0.32 (0.30 to 0.35)

United Kingdom	2021	Total knee replacement	73,000	1.08	95,800 (86,700 to 106,000)	1.42 (1.29 to 1.57)	-22,800 (-32,800 to -13,600)	-0.338 (-0.487 to -0.202)	0.76 (0.69 to 0.84)
United Kingdom	2020	Cataract surgery	288,000	4.29	558,000 (516,000 to 603,000)	8.32 (7.70 to 8.99)	-270,000 (-315,000 to -229,000)	-4.03 (-4.69 to -3.41)	0.52 (0.48 to 0.56)
United Kingdom	2021	Cataract surgery	562,000	8.34	575,000 (516,000 to 642,000)	8.54 (7.66 to 9.53)	-13,500 (-80,100 to 45,000)	-0.201 (-1.19 to 0.668)	0.98 (0.88 to 1.09)
United Kingdom	2020	Tonsillectomy	18,200	0.271	49,900 (44,700 to 55,800)	0.744 (0.666 to 0.832)	-31,700 (-37,600 to -26,400)	-0.473 (-0.561 to -0.393)	0.36 (0.33 to 0.41)
United Kingdom	2021	Tonsillectomy	31,100	0.462	50,100 (42,800 to 58,700)	0.744 (0.636 to 0.871)	-19,000 (-27,400 to -11,800)	-0.282 (-0.407 to -0.175)	0.62 (0.53 to 0.73)
United Kingdom	2020	Hysterectomy	28,400	0.423	44,800 (43,200 to 46,500)	0.668 (0.643 to 0.694)	-16,400 (-18,200 to -14,800)	-0.245 (-0.271 to -0.221)	0.63 (0.61 to 0.66)
United Kingdom	2021	Hysterectomy	40,300	0.598	42,400 (40,800 to 44,100)	0.630 (0.606 to 0.654)	-2,120 (-3,760 to -540)	-0.032 (-0.056 to -0.008)	0.95 (0.91 to 0.99)
United Kingdom	2020	Open prostatectomy	6,990	0.104	9,440 (7,620 to 11,700)	0.141 (0.114 to 0.174)	-2,450 (-4,700 to -632)	-0.036 (-0.070 to -0.009)	0.74 (0.60 to 0.92)
United Kingdom	2021	Open prostatectomy	8,470	0.126	9,480 (7,010 to 12,800)	0.141 (0.104 to 0.190)	-1,010 (-4,280 to 1,460)	-0.015 (-0.063 to 0.022)	0.89 (0.66 to 1.21)
United Kingdom	2020	Transurethral prostatectomy	13,300	0.198	24,200 (23,500 to 24,900)	0.361 (0.350 to 0.372)	-10,900 (-11,600 to -10,200)	-0.162 (-0.173 to -0.152)	0.55 (0.53 to 0.57)
United Kingdom	2021	Transurethral prostatectomy	21,100	0.313	23,700 (22,700 to 24,700)	0.351 (0.337 to 0.367)	-2,590 (-3,590 to -1,620)	-0.039 (-0.053 to -0.024)	0.89 (0.85 to 0.93)
Hungary	2020	All surgeries	194,000	19.9	269,000 (255,000 to 284,000)	27.6 (26.2 to 29.2)	-75,300 (-90,500 to -61,100)	-7.72 (-9.28 to -6.27)	0.72 (0.68 to 0.76)
Hungary	2021	All surgeries	155,000	16	275,000 (255,000 to 297,000)	28.3 (26.2 to 30.6)	-120,000 (-142,000 to -99,400)	-12.3 (-14.7 to -10.2)	0.56 (0.52 to 0.61)
Hungary	2020	Non-elective surgeries	31,700	3.26	42,500 (41,400 to 43,700)	4.36 (4.25 to 4.48)	-10,800 (-11,900 to -9,670)	-1.11 (-1.22 to -0.992)	0.75 (0.73 to 0.77)
Hungary	2021	Non-elective surgeries	25,500	2.63	42,300 (41,200 to 43,500)	4.36 (4.25 to 4.48)	-16,800 (-17,900 to -15,700)	-1.73 (-1.85 to -1.62)	0.60 (0.59 to 0.62)
Hungary	2020	Appendectomy	8,310	0.853	9,460 (8,980 to 9,980)	0.971 (0.921 to 1.02)	-1,150 (-1,670 to -671)	-0.118 (-0.171 to -0.069)	0.88 (0.83 to 0.93)
Hungary	2021	Appendectomy	8,000	0.824	9,430 (8,940 to 9,940)	0.971 (0.921 to 1.02)	-1,420 (-1,930 to -945)	-0.147 (-0.199 to -0.097)	0.85 (0.81 to 0.89)
Hungary	2020	Cholecystectomy	15,600	1.6	23,400 (22,800 to 23,900)	2.40 (2.34 to 2.45)	-7,820 (-8,370 to -7,280)	-0.802 (-0.858 to -0.746)	0.67 (0.65 to 0.68)
Hungary	2021	Cholecystectomy	11,100	1.14	23,300 (22,700 to 23,800)	2.40 (2.34 to 2.45)	-12,200 (-12,800 to -11,600)	-1.26 (-1.31 to -1.20)	0.48 (0.46 to 0.49)
Hungary	2020	Total mastectomy	2,190	0.225	2,430 (2,280 to 2,580)	0.249 (0.234 to 0.265)	-233 (-388 to -87)	-0.024 (-0.040 to -0.009)	0.90 (0.85 to 0.96)
Hungary	2021	Total mastectomy	1,770	0.183	2,420 (2,210 to 2,640)	0.249 (0.228 to 0.272)	-642 (-865 to -438)	-0.066 (-0.089 to -0.045)	0.73 (0.67 to 0.80)
Hungary	2020	Partial excision of mammary gland	5,680	0.582	6,530 (6,310 to 6,760)	0.670 (0.647 to 0.693)	-851 (-1,080 to -632)	-0.087 (-0.110 to -0.065)	0.87 (0.84 to 0.90)

Hungary	2021	Partial excision of mammary gland	4,680	0.482	6,430 (6,120 to 6,750)	0.662 (0.631 to 0.695)	-1,750 (-2,070 to -1,450)	-0.180 (-0.213 to -0.149)	0.73 (0.69 to 0.76)
Hungary	2020	Both	71,800	7.36	81,200 (79,100 to 83,500)	8.33 (8.11 to 8.56)	-9,460 (-11,600 to -7,340)	-0.971 (-1.19 to -0.752)	0.88 (0.86 to 0.91)
Hungary	2021	Both	62,800	6.47	81,500 (76,700 to 86,500)	8.39 (7.90 to 8.91)	-18,700 (-23,600 to -14,000)	-1.92 (-2.44 to -1.44)	0.77 (0.73 to 0.82)
Hungary	2020	Caesarean section	35,900	3.68	36,400 (34,800 to 38,200)	3.74 (3.57 to 3.92)	-539 (-2,280 to 1,120)	-0.055 (-0.234 to 0.115)	0.99 (0.94 to 1.03)
Hungary	2021	Caesarean section	32,500	3.35	37,200 (34,800 to 39,700)	3.83 (3.58 to 4.09)	-4,650 (-7,210 to -2,280)	-0.479 (-0.742 to -0.235)	0.87 (0.82 to 0.93)
Hungary	2020	Repair of inguinal hernia	12,600	1.3	18,700 (17,800 to 19,700)	1.92 (1.82 to 2.02)	-6,070 (-7,030 to -5,160)	-0.623 (-0.721 to -0.529)	0.68 (0.64 to 0.71)
Hungary	2021	Repair of inguinal hernia	9,340	0.962	18,600 (16,600 to 20,800)	1.91 (1.71 to 2.14)	-9,240 (-11,500 to -7,250)	-0.951 (-1.18 to -0.746)	0.50 (0.45 to 0.56)
Hungary	2020	Transluminal coronary angioplasty	20,900	2.14	24,600 (23,700 to 25,600)	2.52 (2.43 to 2.62)	-3,730 (-4,710 to -2,780)	-0.382 (-0.483 to -0.285)	0.85 (0.82 to 0.88)
Hungary	2021	Transluminal coronary angioplasty	19,200	1.97	25,500 (24,200 to 27,000)	2.63 (2.49 to 2.78)	-6,370 (-7,800 to -5,000)	-0.656 (-0.803 to -0.515)	0.75 (0.71 to 0.79)
Hungary	2020	Coronary artery bypass graft	2,360	0.243	2,790 (2,510 to 3,100)	0.286 (0.257 to 0.318)	-422 (-741 to -139)	-0.043 (-0.076 to -0.014)	0.85 (0.76 to 0.94)
Hungary	2021	Coronary artery bypass graft	1,770	0.182	2,780 (2,500 to 3,090)	0.286 (0.257 to 0.318)	-1,010 (-1,320 to -729)	-0.104 (-0.136 to -0.075)	0.64 (0.57 to 0.71)
Hungary	2020	Elective surgeries	90,600	9.29	146,000 (133,000 to 159,000)	14.9 (13.7 to 16.3)	-55,000 (-68,200 to -42,700)	-5.64 (-6.99 to -4.38)	0.62 (0.57 to 0.68)
Hungary	2021	Elective surgeries	66,900	6.89	150,000 (133,000 to 169,000)	15.4 (13.7 to 17.4)	-83,000 (-103,000 to -66,000)	-8.55 (-10.6 to -6.80)	0.45 (0.39 to 0.50)
Hungary	2020	Hip replacement	10,500	1.07	15,100 (13,200 to 17,200)	1.55 (1.36 to 1.76)	-4,610 (-6,710 to -2,760)	-0.473 (-0.688 to -0.283)	0.69 (0.61 to 0.79)
Hungary	2021	Hip replacement	7,630	0.786	15,800 (13,100 to 19,000)	1.63 (1.35 to 1.96)	-8,190 (-11,500 to -5,500)	-0.844 (-1.18 to -0.567)	0.48 (0.40 to 0.58)
Hungary	2020	Total knee replacement	5,580	0.572	10,200 (7,610 to 13,600)	1.04 (0.781 to 1.39)	-4,580 (-8,040 to -2,010)	-0.470 (-0.824 to -0.206)	0.55 (0.41 to 0.73)
Hungary	2021	Total knee replacement	2,720	0.28	11,200 (7,470 to 16,900)	1.16 (0.769 to 1.74)	-8,510 (-14,100 to -4,710)	-0.877 (-1.45 to -0.485)	0.24 (0.16 to 0.37)
Hungary	2020	Cataract surgery	60,400	6.19	98,700 (89,300 to 109,000)	10.1 (9.16 to 11.2)	-38,300 (-48,900 to -29,000)	-3.93 (-5.02 to -2.98)	0.61 (0.55 to 0.68)
Hungary	2021	Cataract surgery	46,600	4.8	103,000 (89,100 to 118,000)	10.6 (9.18 to 12.2)	-56,100 (-72,000 to -42,500)	-5.77 (-7.42 to -4.38)	0.45 (0.39 to 0.52)
Hungary	2020	Tonsillectomy	4,570	0.469	9,650 (8,990 to 10,400)	0.990 (0.922 to 1.06)	-5,080 (-5,800 to -4,420)	-0.521 (-0.595 to -0.453)	0.47 (0.44 to 0.51)
Hungary	2021	Tonsillectomy	2,650	0.273	9,300 (8,410 to 10,300)	0.958 (0.866 to 1.06)	-6,660 (-7,660 to -5,770)	-0.685 (-0.789 to -0.594)	0.28 (0.26 to 0.31)
Hungary	2020	Hysterectomy	6,510	0.667	8,630 (8,150 to 9,140)	0.885 (0.836 to 0.938)	-2,120 (-2,620 to -1,650)	-0.218 (-0.269 to -0.169)	0.75 (0.71 to 0.80)
Hungary	2021	Hysterectomy	5,070	0.522	8,600 (8,120 to 9,100)	0.885 (0.836 to 0.938)	-3,530 (-4,030 to -3,040)	-0.363 (-0.415 to -0.313)	0.59 (0.56 to 0.62)

Hungary	2020	Open prostatectomy	684	0.07	938 (808 to 1,090)	0.096 (0.083 to 0.112)	-254 (-407 to -125)	-0.026 (-0.042 to -0.013)	0.73 (0.63 to 0.85)
Hungary	2021	Open prostatectomy	470	0.048	877 (751 to 1,020)	0.090 (0.077 to 0.106)	-407 (-551 to -281)	-0.042 (-0.057 to -0.029)	0.54 (0.46 to 0.63)
Hungary	2020	Transurethral prostatectomy	2,370	0.243	3,420 (3,200 to 3,660)	0.351 (0.328 to 0.375)	-1,060 (-1,300 to -831)	-0.108 (-0.133 to -0.085)	0.69 (0.65 to 0.74)
Hungary	2021	Transurethral prostatectomy	1,770	0.183	3,410 (3,190 to 3,650)	0.351 (0.328 to 0.375)	-1,640 (-1,870 to -1,420)	-0.168 (-0.192 to -0.146)	0.52 (0.49 to 0.56)
Iceland	2020	All surgeries	7,330	20	8,030 (4,380 to 14,700)	21.9 (11.9 to 40.2)	-704 (-7,420 to 2,940)	-1.92 (-20.2 to 8.02)	0.91 (0.50 to 1.67)
Iceland	2021	All surgeries	7,420	19.9	8,160 (3,460 to 19,200)	21.9 (9.29 to 51.7)	-747 (-11,700 to 3,940)	-2.00 (-31.4 to 10.6)	0.91 (0.39 to 2.13)
Iceland	2020	Non-elective surgeries	1,600	4.36	1,760 (1,630 to 1,900)	4.81 (4.45 to 5.20)	-166 (-307 to -33)	-0.452 (-0.839 to -0.090)	0.91 (0.84 to 0.98)
Iceland	2021	Non-elective surgeries	1,680	4.5	1,790 (1,660 to 1,940)	4.81 (4.45 to 5.20)	-115 (-262 to 20)	-0.308 (-0.702 to 0.055)	0.94 (0.87 to 1.01)
Iceland	2020	Appendectomy	563	1.54	576 (520 to 638)	1.57 (1.42 to 1.74)	-13 (-75 to 43)	-0.036 (-0.204 to 0.118)	0.98 (0.88 to 1.08)
Iceland	2021	Appendectomy	541	1.45	586 (529 to 648)	1.57 (1.42 to 1.74)	-45 (-110 to 11)	-0.120 (-0.294 to 0.030)	0.92 (0.83 to 1.02)
Iceland	2020	Cholecystectomy	707	1.93	867 (798 to 942)	2.37 (2.18 to 2.57)	-160 (-235 to -90)	-0.436 (-0.641 to -0.246)	0.82 (0.75 to 0.89)
Iceland	2021	Cholecystectomy	795	2.13	881 (811 to 957)	2.37 (2.18 to 2.57)	-86 (-163 to -16)	-0.231 (-0.438 to -0.042)	0.90 (0.83 to 0.98)
Iceland	2020	Total mastectomy	176	0.48	193 (154 to 243)	0.527 (0.419 to 0.663)	-17 (-66 to 23)	-0.047 (-0.180 to 0.062)	0.91 (0.73 to 1.15)
Iceland	2021	Total mastectomy	148	0.397	196 (142 to 272)	0.527 (0.381 to 0.729)	-48 (-125 to 6)	-0.130 (-0.334 to 0.017)	0.75 (0.54 to 1.04)
Iceland	2020	Partial excision of mammary gland	151	0.412	149 (114 to 195)	0.408 (0.312 to 0.533)	2 (-45 to 37)	0.004 (-0.122 to 0.100)	1.01 (0.77 to 1.32)
Iceland	2021	Partial excision of mammary gland	193	0.518	152 (116 to 199)	0.408 (0.312 to 0.533)	41 (-5 to 77)	0.110 (-0.013 to 0.206)	1.27 (0.98 to 1.66)
Iceland	2020	Both	1,360	3.72	1,420 (1,290 to 1,560)	3.88 (3.53 to 4.26)	-59 (-199 to 71)	-0.161 (-0.543 to 0.194)	0.96 (0.87 to 1.06)
Iceland	2021	Both	1,430	3.84	1,440 (1,260 to 1,650)	3.88 (3.39 to 4.44)	-13 (-217 to 168)	-0.035 (-0.582 to 0.451)	0.99 (0.87 to 1.13)
Iceland	2020	Caesarean section	720	1.96	722 (674 to 772)	1.97 (1.84 to 2.11)	-2 (-53 to 45)	-0.004 (-0.145 to 0.124)	1.00 (0.93 to 1.07)
Iceland	2021	Caesarean section	701	1.88	733 (666 to 807)	1.97 (1.79 to 2.17)	-32 (-106 to 34)	-0.087 (-0.284 to 0.092)	0.96 (0.87 to 1.05)
Iceland	2020	Transluminal coronary angioplasty	572	1.56	746 (622 to 896)	2.04 (1.70 to 2.44)	-174 (-327 to -50)	-0.475 (-0.893 to -0.137)	0.77 (0.64 to 0.92)
Iceland	2021	Transluminal coronary angioplasty	664	1.78	758 (632 to 910)	2.04 (1.70 to 2.44)	-94 (-244 to 31)	-0.253 (-0.656 to 0.082)	0.88 (0.73 to 1.05)
Iceland	2020	Coronary artery bypass graft	70	0.191	74 (50 to 110)	0.202 (0.136 to 0.301)	-4 (-40 to 20)	-0.011 (-0.110 to 0.055)	0.94 (0.63 to 1.40)
Iceland	2021	Coronary artery bypass graft	66	0.177	75 (43 to 132)	0.202 (0.116 to 0.354)	-9 (-67 to 23)	-0.025 (-0.179 to 0.061)	0.88 (0.50 to 1.52)
Iceland	2020	Elective surgeries	4,370	11.9	4,820 (2,900 to 8,010)	13.1 (7.90 to 21.8)	-447 (-3,600 to 1,460)	-1.22 (-9.83 to 3.99)	0.91 (0.55 to 1.50)

Iceland	2021	Elective surgeries	4,310	11.6	4,890 (2,380 to 10,000)	13.1 (6.40 to 27.0)	-585 (-5,710 to 1,900)	-1.57 (-15.3 to 5.10)	0.88 (0.43 to 1.79)
Iceland	2020	Hip replacement	626	1.71	716 (543 to 943)	1.95 (1.48 to 2.57)	-90 (-319 to 83)	-0.245 (-0.869 to 0.226)	0.87 (0.66 to 1.15)
Iceland	2021	Hip replacement	831	2.23	727 (552 to 959)	1.95 (1.48 to 2.57)	104 (-132 to 282)	0.278 (-0.354 to 0.757)	1.14 (0.86 to 1.51)
Iceland	2020	Cataract surgery	3,340	9.1	3,530 (1,790 to 6,950)	9.63 (4.89 to 19.0)	-192 (-3,560 to 1,540)	-0.523 (-9.72 to 4.20)	0.95 (0.48 to 1.86)
Iceland	2021	Cataract surgery	2,980	7.99	3,590 (1,370 to 9,350)	9.63 (3.69 to 25.1)	-608 (-6,370 to 1,590)	-1.63 (-17.1 to 4.27)	0.83 (0.32 to 2.14)
Iceland	2020	Hysterectomy	335	0.914	426 (358 to 508)	1.16 (0.975 to 1.39)	-91 (-172 to -23)	-0.249 (-0.469 to -0.062)	0.79 (0.66 to 0.94)
Iceland	2021	Hysterectomy	390	1.05	433 (363 to 516)	1.16 (0.975 to 1.39)	-43 (-125 to 26)	-0.116 (-0.337 to 0.069)	0.90 (0.76 to 1.07)
Iceland	2020	Open prostatectomy	19	0.052	55 (39 to 78)	0.149 (0.105 to 0.212)	-36 (-59 to -20)	-0.097 (-0.162 to -0.053)	0.35 (0.24 to 0.49)
Iceland	2021	Open prostatectomy	24	0.064	56 (39 to 79)	0.149 (0.105 to 0.212)	-32 (-55 to -15)	-0.085 (-0.148 to -0.041)	0.43 (0.30 to 0.61)
Iceland	2020	Transurethral prostatectomy	53	0.145	68 (40 to 117)	0.186 (0.108 to 0.320)	-15 (-63 to 13)	-0.041 (-0.172 to 0.036)	0.78 (0.46 to 1.33)
Iceland	2021	Transurethral prostatectomy	86	0.231	69 (32 to 149)	0.186 (0.086 to 0.401)	17 (-63 to 54)	0.045 (-0.169 to 0.145)	1.24 (0.58 to 2.68)
Israel	2020	All surgeries	202,000	21.9	207,000 (198,000 to 215,000)	22.4 (21.5 to 23.3)	-4,600 (-12,800 to 3,560)	-0.499 (-1.39 to 0.386)	0.98 (0.94 to 1.02)
Israel	2021	All surgeries	210,000	22.4	210,000 (199,000 to 222,000)	22.4 (21.2 to 23.7)	255 (-11,900 to 11,900)	0.027 (-1.27 to 1.27)	1.00 (0.95 to 1.06)
Israel	2020	Non-elective surgeries	29,700	3.23	29,400 (27,900 to 30,900)	3.19 (3.03 to 3.36)	370 (-1,210 to 1,810)	0.040 (-0.131 to 0.196)	1.01 (0.96 to 1.06)
Israel	2021	Non-elective surgeries	31,300	3.34	29,900 (27,800 to 32,100)	3.19 (2.96 to 3.43)	1,420 (-833 to 3,490)	0.151 (-0.089 to 0.373)	1.05 (0.97 to 1.13)
Israel	2020	Appendectomy	9,540	1.04	9,070 (8,550 to 9,610)	0.984 (0.928 to 1.04)	473 (-75 to 986)	0.051 (-0.008 to 0.107)	1.05 (0.99 to 1.12)
Israel	2021	Appendectomy	9,880	1.05	8,990 (8,270 to 9,770)	0.959 (0.883 to 1.04)	895 (134 to 1,610)	0.096 (0.014 to 0.172)	1.10 (1.01 to 1.19)
Israel	2020	Cholecystectomy	12,100	1.31	11,900 (11,200 to 12,700)	1.29 (1.22 to 1.37)	162 (-557 to 837)	0.018 (-0.060 to 0.091)	1.01 (0.96 to 1.07)
Israel	2021	Cholecystectomy	12,800	1.37	12,100 (11,400 to 12,900)	1.29 (1.22 to 1.37)	689 (-53 to 1,370)	0.074 (-0.006 to 0.146)	1.06 (1.00 to 1.12)
Israel	2020	Total mastectomy	1,490	0.162	1,540 (1,330 to 1,780)	0.167 (0.144 to 0.193)	-44 (-288 to 168)	-0.005 (-0.031 to 0.018)	0.97 (0.84 to 1.13)
Israel	2021	Total mastectomy	1,630	0.174	1,560 (1,350 to 1,810)	0.167 (0.144 to 0.193)	63 (-179 to 280)	0.007 (-0.019 to 0.030)	1.04 (0.90 to 1.21)
Israel	2020	Partial excision of mammary gland	6,620	0.718	6,610 (6,360 to 6,860)	0.717 (0.690 to 0.745)	10 (-249 to 256)	0.001 (-0.027 to 0.028)	1.00 (0.96 to 1.04)
Israel	2021	Partial excision of mammary gland	6,960	0.742	6,720 (6,470 to 6,980)	0.717 (0.690 to 0.745)	237 (-25 to 489)	0.025 (-0.003 to 0.052)	1.04 (1.00 to 1.08)
Israel	2020	Both	70,500	7.65	73,900 (71,100 to 76,900)	8.02 (7.71 to 8.34)	-3,460 (-6,420 to -585)	-0.375 (-0.697 to -0.063)	0.95 (0.92 to 0.99)
Israel	2021	Both	70,600	7.53	75,200 (71,100 to 79,500)	8.02 (7.59 to 8.48)	-4,590 (-8,820 to -453)	-0.490 (-0.942 to -0.048)	0.94 (0.89 to 0.99)

Israel	2020	Caesarean section	28,100	3.05	28,400 (27,200 to 29,700)	3.08 (2.95 to 3.22)	-278 (-1,540 to 951)	-0.030 (-0.167 to 0.103)	0.99 (0.95 to 1.03)
Israel	2021	Caesarean section	26,500	2.83	28,300 (26,600 to 30,100)	3.02 (2.84 to 3.21)	-1,780 (-3,580 to -104)	-0.190 (-0.382 to -0.011)	0.94 (0.88 to 1.00)
Israel	2020	Repair of inguinal hernia	15,700	1.71	17,500 (16,800 to 18,200)	1.89 (1.82 to 1.97)	-1,720 (-2,420 to -1,040)	-0.186 (-0.263 to -0.113)	0.90 (0.87 to 0.94)
Israel	2021	Repair of inguinal hernia	16,700	1.79	17,700 (17,100 to 18,500)	1.89 (1.82 to 1.97)	-1,020 (-1,730 to -323)	-0.109 (-0.185 to -0.034)	0.94 (0.91 to 0.98)
Israel	2020	Transluminal coronary angioplasty	23,600	2.56	24,300 (23,200 to 25,400)	2.63 (2.52 to 2.76)	-688 (-1,800 to 381)	-0.075 (-0.195 to 0.041)	0.97 (0.93 to 1.02)
Israel	2021	Transluminal coronary angioplasty	24,300	2.59	24,700 (23,200 to 26,300)	2.63 (2.47 to 2.81)	-392 (-2,030 to 1,120)	-0.042 (-0.217 to 0.120)	0.98 (0.92 to 1.05)
Israel	2020	Coronary artery bypass graft	3,010	0.327	3,340 (2,970 to 3,750)	0.362 (0.322 to 0.407)	-323 (-737 to 38)	-0.035 (-0.080 to 0.004)	0.90 (0.80 to 1.01)
Israel	2021	Coronary artery bypass graft	3,030	0.323	3,390 (2,880 to 4,000)	0.362 (0.307 to 0.427)	-364 (-967 to 147)	-0.039 (-0.103 to 0.016)	0.89 (0.76 to 1.05)
Israel	2020	Elective surgeries	102,000	11	103,000 (96,400 to 111,000)	11.2 (10.5 to 12.0)	-1,510 (-8,830 to 5,360)	-0.164 (-0.958 to 0.581)	0.99 (0.92 to 1.06)
Israel	2021	Elective surgeries	108,000	11.6	105,000 (95,300 to 116,000)	11.2 (10.2 to 12.4)	3,430 (-7,350 to 13,100)	0.366 (-0.784 to 1.40)	1.03 (0.94 to 1.14)
Israel	2020	Hip replacement	6,130	0.665	6,630 (6,270 to 7,020)	0.720 (0.680 to 0.761)	-500 (-887 to -134)	-0.054 (-0.096 to -0.014)	0.92 (0.87 to 0.98)
Israel	2021	Hip replacement	6,840	0.73	6,910 (6,380 to 7,480)	0.737 (0.681 to 0.798)	-69 (-631 to 461)	-0.007 (-0.067 to 0.049)	0.99 (0.92 to 1.07)
Israel	2020	Total knee replacement	5,920	0.643	7,000 (6,190 to 7,910)	0.759 (0.672 to 0.858)	-1,080 (-1,970 to -284)	-0.117 (-0.214 to -0.031)	0.85 (0.75 to 0.95)
Israel	2021	Total knee replacement	6,780	0.724	7,460 (6,280 to 8,870)	0.796 (0.670 to 0.946)	-679 (-2,060 to 479)	-0.072 (-0.220 to 0.051)	0.91 (0.77 to 1.08)
Israel	2020	Cataract surgery	71,900	7.8	71,100 (65,400 to 77,500)	7.72 (7.09 to 8.41)	774 (-5,650 to 6,650)	0.084 (-0.613 to 0.722)	1.01 (0.93 to 1.10)
Israel	2021	Cataract surgery	75,200	8.02	72,400 (64,200 to 81,600)	7.72 (6.85 to 8.71)	2,820 (-6,570 to 11,000)	0.301 (-0.701 to 1.17)	1.04 (0.92 to 1.17)
Israel	2020	Tonsillectomy	7,280	0.791	8,600 (7,700 to 9,600)	0.933 (0.836 to 1.04)	-1,320 (-2,310 to -404)	-0.143 (-0.250 to -0.044)	0.85 (0.76 to 0.95)
Israel	2021	Tonsillectomy	8,340	0.89	8,750 (7,830 to 9,770)	0.933 (0.836 to 1.04)	-410 (-1,440 to 522)	-0.044 (-0.153 to 0.056)	0.95 (0.85 to 1.07)
Israel	2020	Hysterectomy	5,390	0.585	5,460 (5,210 to 5,720)	0.593 (0.565 to 0.621)	-69 (-335 to 184)	-0.008 (-0.036 to 0.020)	0.99 (0.94 to 1.04)
Israel	2021	Hysterectomy	5,670	0.605	5,720 (5,450 to 6,000)	0.610 (0.582 to 0.640)	-54 (-338 to 208)	-0.006 (-0.036 to 0.022)	0.99 (0.94 to 1.04)
Israel	2020	Open prostatectomy	1,360	0.147	1,310 (1,130 to 1,520)	0.143 (0.123 to 0.165)	42 (-167 to 228)	0.005 (-0.018 to 0.025)	1.03 (0.89 to 1.20)
Israel	2021	Open prostatectomy	1,390	0.148	1,270 (1,030 to 1,560)	0.135 (0.110 to 0.167)	123 (-172 to 362)	0.013 (-0.018 to 0.039)	1.10 (0.89 to 1.35)
Israel	2020	Transurethral prostatectomy	3,740	0.405	3,510 (3,260 to 3,780)	0.381 (0.353 to 0.410)	228 (-43 to 481)	0.025 (-0.005 to 0.052)	1.06 (0.99 to 1.15)
Israel	2021	Transurethral prostatectomy	4,240	0.452	3,570 (3,310 to 3,840)	0.381 (0.353 to 0.410)	672 (397 to 926)	0.072 (0.042 to 0.099)	1.19 (1.10 to 1.28)
Italy	2020	All surgeries	1,180,000	19.9	1,580,000 (1,430,000 to 1,750,000)	26.6 (24.0 to 29.4)	-396,000 (-564,000 to -241,000)	-6.66 (-9.49 to -4.05)	0.75 (0.68 to 0.83)

Italy	2021	All surgeries	1,420,000	24.1	1,570,000 (1,360,000 to 1,820,000)	26.6 (23.0 to 30.7)	-149,000 (-393,000 to 65,600)	-2.52 (-6.64 to 1.11)	0.91 (0.78 to 1.05)
Italy	2020	Non-elective surgeries	188,000	3.17	235,000 (230,000 to 240,000)	3.95 (3.87 to 4.03)	-46,500 (-51,200 to -41,800)	-0.782 (-0.861 to -0.703)	0.80 (0.79 to 0.82)
Italy	2021	Non-elective surgeries	213,000	3.6	231,000 (224,000 to 237,000)	3.90 (3.79 to 4.01)	-17,700 (-24,100 to -11,300)	-0.299 (-0.408 to -0.191)	0.92 (0.90 to 0.95)
Italy	2020	Appendectomy	32,800	0.551	38,200 (37,100 to 39,300)	0.643 (0.624 to 0.662)	-5,450 (-6,540 to -4,390)	-0.092 (-0.110 to -0.074)	0.86 (0.83 to 0.88)
Italy	2021	Appendectomy	34,500	0.584	36,800 (35,300 to 38,300)	0.622 (0.597 to 0.647)	-2,230 (-3,760 to -760)	-0.038 (-0.064 to -0.013)	0.94 (0.90 to 0.98)
Italy	2020	Cholecystectomy	80,000	1.35	109,000 (106,000 to 112,000)	1.84 (1.79 to 1.89)	-29,200 (-32,500 to -26,300)	-0.492 (-0.547 to -0.442)	0.73 (0.71 to 0.75)
Italy	2021	Cholecystectomy	93,200	1.58	108,000 (103,000 to 112,000)	1.82 (1.75 to 1.89)	-14,400 (-18,800 to -10,200)	-0.243 (-0.317 to -0.172)	0.87 (0.83 to 0.90)
Italy	2020	Total mastectomy	21,300	0.358	23,500 (23,000 to 23,900)	0.395 (0.387 to 0.402)	-2,200 (-2,670 to -1,750)	-0.037 (-0.045 to -0.029)	0.91 (0.89 to 0.92)
Italy	2021	Total mastectomy	23,000	0.388	23,700 (23,100 to 24,400)	0.401 (0.390 to 0.412)	-734 (-1,400 to -110)	-0.012 (-0.024 to -0.002)	0.97 (0.94 to 1.00)
Italy	2020	Partial excision of mammary gland	54,300	0.913	64,700 (62,500 to 66,900)	1.09 (1.05 to 1.13)	-10,400 (-12,700 to -8,210)	-0.175 (-0.213 to -0.138)	0.84 (0.81 to 0.87)
Italy	2021	Partial excision of mammary gland	62,400	1.05	64,400 (61,300 to 67,600)	1.09 (1.04 to 1.14)	-1,990 (-5,150 to 1,040)	-0.034 (-0.087 to 0.018)	0.97 (0.92 to 1.02)
Italy	2020	Both	342,000	5.75	415,000 (404,000 to 426,000)	6.99 (6.80 to 7.17)	-73,500 (-84,500 to -62,900)	-1.24 (-1.42 to -1.06)	0.82 (0.80 to 0.84)
Italy	2021	Both	372,000	6.29	411,000 (388,000 to 436,000)	6.95 (6.55 to 7.37)	-39,100 (-63,400 to -15,800)	-0.661 (-1.07 to -0.266)	0.90 (0.85 to 0.96)
Italy	2020	Caesarean section	131,000	2.21	130,000 (127,000 to 132,000)	2.18 (2.14 to 2.23)	1,700 (-1,070 to 4,390)	0.029 (-0.018 to 0.074)	1.01 (0.99 to 1.03)
Italy	2021	Caesarean section	129,000	2.19	123,000 (119,000 to 126,000)	2.07 (2.01 to 2.13)	6,830 (3,280 to 10,400)	0.115 (0.056 to 0.175)	1.06 (1.03 to 1.09)
Italy	2020	Repair of inguinal hernia	78,500	1.32	124,000 (121,000 to 128,000)	2.09 (2.03 to 2.15)	-45,600 (-49,100 to -42,200)	-0.767 (-0.827 to -0.710)	0.63 (0.61 to 0.65)
Italy	2021	Repair of inguinal hernia	96,000	1.62	123,000 (116,000 to 131,000)	2.08 (1.96 to 2.22)	-27,200 (-35,200 to -19,600)	-0.459 (-0.596 to -0.332)	0.78 (0.73 to 0.83)
Italy	2020	Transluminal coronary angioplasty	116,000	1.96	138,000 (134,000 to 143,000)	2.33 (2.25 to 2.41)	-21,900 (-26,700 to -17,000)	-0.368 (-0.449 to -0.287)	0.84 (0.81 to 0.87)
Italy	2021	Transluminal coronary angioplasty	130,000	2.19	140,000 (133,000 to 147,000)	2.36 (2.25 to 2.48)	-10,100 (-17,300 to -3,210)	-0.171 (-0.292 to -0.054)	0.93 (0.88 to 0.98)
Italy	2020	Coronary artery bypass graft	15,400	0.259	20,500 (20,000 to 21,000)	0.345 (0.336 to 0.354)	-5,100 (-5,620 to -4,580)	-0.086 (-0.094 to -0.077)	0.75 (0.73 to 0.77)

Italy	2021	Coronary artery bypass graft	16,900	0.286	20,600 (19,500 to 21,800)	0.349 (0.329 to 0.369)	-3,720 (-4,950 to -2,580)	-0.063 (-0.084 to -0.044)	0.82 (0.77 to 0.87)
Italy	2020	Elective surgeries	654,000	11	924,000 (715,000 to 1,190,000)	15.6 (12.0 to 20.1)	-271,000 (-536,000 to -64,700)	-4.56 (-9.01 to -1.09)	0.71 (0.55 to 0.91)
Italy	2021	Elective surgeries	837,000	14.2	920,000 (640,000 to 1,320,000)	15.6 (10.8 to 22.3)	-82,300 (-484,000 to 199,000)	-1.39 (-8.19 to 3.36)	0.91 (0.63 to 1.31)
Italy	2020	Hip replacement	96,600	1.63	118,000 (115,000 to 121,000)	1.98 (1.93 to 2.04)	-21,200 (-24,400 to -18,100)	-0.357 (-0.411 to -0.305)	0.82 (0.80 to 0.84)
Italy	2021	Hip replacement	115,000	1.94	120,000 (115,000 to 125,000)	2.03 (1.95 to 2.11)	-5,020 (-9,630 to -641)	-0.085 (-0.163 to -0.011)	0.96 (0.92 to 0.99)
Italy	2020	Total knee replacement	61,900	1.04	87,100 (82,700 to 91,700)	1.46 (1.39 to 1.54)	-25,200 (-29,800 to -20,700)	-0.423 (-0.502 to -0.349)	0.71 (0.67 to 0.75)
Italy	2021	Total knee replacement	75,000	1.27	92,300 (82,300 to 104,000)	1.56 (1.39 to 1.75)	-17,300 (-28,700 to -7,380)	-0.293 (-0.486 to -0.125)	0.81 (0.72 to 0.91)
Italy	2020	Cataract surgery	386,000	6.5	576,000 (330,000 to 1,000,000)	9.69 (5.56 to 16.9)	-190,000 (-627,000 to 53,200)	-3.19 (-10.6 to 0.894)	0.67 (0.38 to 1.16)
Italy	2021	Cataract surgery	524,000	8.87	573,000 (261,000 to 1,260,000)	9.69 (4.42 to 21.3)	-48,600 (-728,000 to 262,000)	-0.822 (-12.3 to 4.43)	0.92 (0.42 to 2.00)
Italy	2020	Tonsillectomy	13,800	0.231	26,300 (25,700 to 26,900)	0.443 (0.433 to 0.453)	-12,600 (-13,100 to -12,000)	-0.211 (-0.221 to -0.201)	0.52 (0.51 to 0.53)
Italy	2021	Tonsillectomy	12,900	0.218	24,900 (24,100 to 25,700)	0.420 (0.407 to 0.434)	-12,000 (-12,800 to -11,200)	-0.203 (-0.216 to -0.190)	0.52 (0.50 to 0.53)
Italy	2020	Hysterectomy	44,200	0.744	55,800 (54,100 to 57,500)	0.938 (0.911 to 0.967)	-11,500 (-13,200 to -9,950)	-0.194 (-0.222 to -0.167)	0.79 (0.77 to 0.82)
Italy	2021	Hysterectomy	51,300	0.867	54,800 (52,500 to 57,100)	0.926 (0.888 to 0.966)	-3,520 (-5,940 to -1,260)	-0.060 (-0.100 to -0.021)	0.94 (0.90 to 0.98)
Italy	2020	Open prostatectomy	19,200	0.323	24,100 (22,500 to 25,700)	0.405 (0.379 to 0.432)	-4,860 (-6,490 to -3,350)	-0.082 (-0.109 to -0.056)	0.80 (0.75 to 0.85)
Italy	2021	Open prostatectomy	21,200	0.358	24,500 (21,200 to 28,400)	0.415 (0.359 to 0.480)	-3,400 (-7,150 to -82)	-0.057 (-0.121 to -0.001)	0.86 (0.75 to 1.00)
Italy	2020	Transurethral prostatectomy	31,700	0.533	44,500 (42,500 to 46,700)	0.749 (0.714 to 0.785)	-12,900 (-15,000 to -10,800)	-0.216 (-0.253 to -0.182)	0.71 (0.68 to 0.75)
Italy	2021	Transurethral prostatectomy	37,700	0.637	45,100 (42,200 to 48,200)	0.763 (0.713 to 0.816)	-7,400 (-10,600 to -4,430)	-0.125 (-0.179 to -0.075)	0.84 (0.78 to 0.89)
Korea	2020	All surgeries	1,370,000	26.4	1,460,000 (1,310,000 to 1,630,000)	28.2 (25.3 to 31.4)	-94,600 (-268,000 to 54,300)	-1.82 (-5.16 to 1.05)	0.94 (0.84 to 1.04)
Korea	2021	All surgeries	1,470,000	28.5	1,540,000 (1,320,000 to 1,790,000)	29.8 (25.6 to 34.7)	-65,200 (-323,000 to 154,000)	-1.26 (-6.25 to 2.98)	0.96 (0.82 to 1.12)
Korea	2020	Non-elective surgeries	220,000	4.25	220,000 (175,000 to 277,000)	4.25 (3.37 to 5.35)	369 (-56,600 to 45,800)	0.007 (-1.09 to 0.884)	1.00 (0.80 to 1.26)
Korea	2021	Non-elective surgeries	236,000	4.57	220,000 (159,000 to 304,000)	4.25 (3.07 to 5.88)	16,600 (-68,800 to 76,900)	0.321 (-1.33 to 1.49)	1.08 (0.77 to 1.48)

Korea	2020	Appendectomy	78,600	1.52	79,400 (73,400 to 85,900)	1.53 (1.42 to 1.66)	-785 (-7,210 to 5,230)	-0.015 (-0.139 to 0.101)	0.99 (0.92 to 1.07)
Korea	2021	Appendectomy	81,200	1.57	77,000 (68,900 to 86,000)	1.49 (1.33 to 1.66)	4,220 (-4,780 to 12,300)	0.082 (-0.092 to 0.238)	1.05 (0.94 to 1.18)
Korea	2020	Cholecystectomy	86,300	1.66	89,500 (82,700 to 96,800)	1.73 (1.60 to 1.87)	-3,220 (-10,300 to 3,450)	-0.062 (-0.199 to 0.066)	0.96 (0.89 to 1.04)
Korea	2021	Cholecystectomy	92,400	1.79	94,500 (84,600 to 106,000)	1.83 (1.63 to 2.04)	-2,080 (-13,100 to 7,920)	-0.040 (-0.253 to 0.153)	0.98 (0.88 to 1.09)
Korea	2020	Total mastectomy	18,900	0.364	18,000 (12,600 to 25,800)	0.347 (0.242 to 0.498)	861 (-6,900 to 6,250)	0.017 (-0.133 to 0.121)	1.05 (0.73 to 1.50)
Korea	2021	Total mastectomy	20,800	0.402	18,000 (12,500 to 25,800)	0.347 (0.242 to 0.498)	2,820 (-4,830 to 8,240)	0.054 (-0.093 to 0.159)	1.16 (0.81 to 1.66)
Korea	2020	Partial excision of mammary gland	36,700	0.709	39,000 (31,500 to 48,400)	0.753 (0.608 to 0.934)	-2,310 (-11,500 to 5,110)	-0.045 (-0.222 to 0.099)	0.94 (0.76 to 1.16)
Korea	2021	Partial excision of mammary gland	41,900	0.809	40,100 (26,700 to 60,400)	0.775 (0.515 to 1.17)	1,780 (-18,200 to 15,300)	0.034 (-0.351 to 0.296)	1.04 (0.70 to 1.58)
Korea	2020	Both	258,000	4.98	270,000 (222,000 to 330,000)	5.21 (4.27 to 6.36)	-12,400 (-72,200 to 35,200)	-0.239 (-1.39 to 0.680)	0.95 (0.78 to 1.16)
Korea	2021	Both	270,000	5.21	270,000 (204,000 to 357,000)	5.21 (3.94 to 6.91)	18 (-86,300 to 65,500)	0.000 (-1.67 to 1.27)	1.00 (0.76 to 1.32)
Korea	2020	Caesarean section	146,000	2.82	153,000 (140,000 to 168,000)	2.96 (2.70 to 3.24)	-6,840 (-21,900 to 6,440)	-0.132 (-0.423 to 0.124)	0.96 (0.87 to 1.05)
Korea	2021	Caesarean section	148,000	2.86	153,000 (134,000 to 174,000)	2.96 (2.60 to 3.37)	-4,950 (-25,900 to 13,600)	-0.096 (-0.501 to 0.262)	0.97 (0.85 to 1.10)
Korea	2020	Repair of inguinal hernia	31,700	0.612	36,700 (34,100 to 39,500)	0.708 (0.657 to 0.762)	-4,940 (-7,800 to -2,380)	-0.095 (-0.151 to -0.046)	0.87 (0.80 to 0.93)
Korea	2021	Repair of inguinal hernia	33,700	0.652	36,600 (34,000 to 39,400)	0.708 (0.657 to 0.762)	-2,910 (-5,730 to -266)	-0.056 (-0.111 to -0.005)	0.92 (0.85 to 0.99)
Korea	2020	Transluminal coronary angioplasty	75,400	1.45	80,600 (75,800 to 85,700)	1.55 (1.46 to 1.65)	-5,180 (-10,300 to -493)	-0.100 (-0.198 to -0.010)	0.94 (0.88 to 0.99)
Korea	2021	Transluminal coronary angioplasty	83,500	1.61	83,500 (76,500 to 91,100)	1.61 (1.48 to 1.76)	-35 (-7,680 to 7,060)	-0.001 (-0.148 to 0.137)	1.00 (0.92 to 1.09)
Korea	2020	Coronary artery bypass graft	4,310	0.083	4,310 (3,770 to 4,930)	0.083 (0.073 to 0.095)	-4 (-630 to 527)	-0.000 (-0.012 to 0.010)	1.00 (0.87 to 1.14)
Korea	2021	Coronary artery bypass graft	4,620	0.089	4,300 (3,560 to 5,210)	0.083 (0.069 to 0.101)	322 (-578 to 1,070)	0.006 (-0.011 to 0.021)	1.07 (0.89 to 1.30)
Korea	2020	Elective surgeries	890,000	17.2	953,000 (891,000 to 1,020,000)	18.4 (17.2 to 19.7)	-62,900 (-130,000 to -2,460)	-1.21 (-2.50 to -0.047)	0.93 (0.87 to 1.00)
Korea	2021	Elective surgeries	968,000	18.7	1,010,000 (919,000 to 1,110,000)	19.5 (17.8 to 21.5)	-41,900 (-143,000 to 49,900)	-0.809 (-2.77 to 0.964)	0.96 (0.87 to 1.05)
Korea	2020	Hip replacement	31,300	0.604	30,500 (16,400 to 56,500)	0.588 (0.317 to 1.09)	823 (-25,200 to 14,900)	0.016 (-0.486 to 0.288)	1.03 (0.55 to 1.91)
Korea	2021	Hip replacement	33,300	0.643	30,400 (12,700 to 72,900)	0.588 (0.246 to 1.41)	2,860 (-39,900 to 20,400)	0.055 (-0.771 to 0.394)	1.09 (0.45 to 2.58)

Korea	2020	Total knee replacement	72,400	1.4	83,000 (75,700 to 91,000)	1.60 (1.46 to 1.76)	-10,700 (-18,600 to -3,320)	-0.206 (-0.359 to -0.064)	0.87 (0.80 to 0.96)
Korea	2021	Total knee replacement	77,600	1.5	86,600 (76,100 to 98,700)	1.67 (1.47 to 1.91)	-9,030 (-21,300 to 1,600)	-0.174 (-0.412 to 0.031)	0.90 (0.78 to 1.02)
Korea	2020	Cataract surgery	703,000	13.6	731,000 (670,000 to 797,000)	14.1 (12.9 to 15.4)	-28,200 (-94,600 to 32,700)	-0.544 (-1.83 to 0.630)	0.96 (0.88 to 1.05)
Korea	2021	Cataract surgery	781,000	15.1	772,000 (682,000 to 873,000)	14.9 (13.2 to 16.9)	9,550 (-93,300 to 101,000)	0.185 (-1.80 to 1.95)	1.01 (0.89 to 1.15)
Korea	2020	Tonsillectomy	31,000	0.598	41,900 (37,700 to 46,700)	0.809 (0.727 to 0.900)	-10,900 (-15,700 to -6,610)	-0.211 (-0.303 to -0.128)	0.74 (0.66 to 0.82)
Korea	2021	Tonsillectomy	21,300	0.411	41,900 (37,600 to 46,600)	0.809 (0.727 to 0.900)	-20,600 (-25,300 to -16,400)	-0.398 (-0.488 to -0.316)	0.51 (0.46 to 0.56)
Korea	2020	Hysterectomy	39,700	0.766	40,800 (38,100 to 43,800)	0.788 (0.735 to 0.844)	-1,150 (-4,110 to 1,540)	-0.022 (-0.079 to 0.030)	0.97 (0.91 to 1.04)
Korea	2021	Hysterectomy	41,200	0.797	39,700 (36,000 to 43,800)	0.767 (0.696 to 0.846)	1,550 (-2,630 to 5,200)	0.030 (-0.051 to 0.101)	1.04 (0.94 to 1.14)
Korea	2020	Open prostatectomy	1,850	0.036	2,230 (1,920 to 2,590)	0.043 (0.037 to 0.050)	-377 (-732 to -61)	-0.007 (-0.014 to -0.001)	0.83 (0.72 to 0.97)
Korea	2021	Open prostatectomy	1,540	0.03	2,230 (1,800 to 2,750)	0.043 (0.035 to 0.053)	-681 (-1,210 to -250)	-0.013 (-0.023 to -0.005)	0.69 (0.56 to 0.86)
Korea	2020	Transurethral prostatectomy	11,600	0.223	12,200 (11,100 to 13,500)	0.236 (0.213 to 0.260)	-650 (-1,930 to 522)	-0.013 (-0.037 to 0.010)	0.95 (0.86 to 1.05)
Korea	2021	Transurethral prostatectomy	12,200	0.237	12,200 (10,600 to 14,000)	0.236 (0.205 to 0.271)	53 (-1,830 to 1,630)	0.001 (-0.035 to 0.031)	1.00 (0.87 to 1.15)
Lithuania	2020	All surgeries	62,600	22.4	86,000 (80,000 to 92,400)	30.8 (28.6 to 33.0)	-23,400 (-29,700 to -17,400)	-8.37 (-10.6 to -6.24)	0.73 (0.68 to 0.78)
Lithuania	2021	All surgeries	71,300	25.5	89,200 (80,600 to 98,700)	31.8 (28.8 to 35.3)	-17,900 (-27,300 to -9,180)	-6.38 (-9.74 to -3.28)	0.80 (0.72 to 0.89)
Lithuania	2020	Non-elective surgeries	11,800	4.21	15,600 (14,900 to 16,400)	5.60 (5.32 to 5.89)	-3,860 (-4,680 to -3,110)	-1.38 (-1.67 to -1.11)	0.75 (0.72 to 0.79)
Lithuania	2021	Non-elective surgeries	13,100	4.67	15,700 (14,900 to 16,500)	5.60 (5.32 to 5.89)	-2,600 (-3,430 to -1,830)	-0.928 (-1.22 to -0.652)	0.83 (0.79 to 0.88)
Lithuania	2020	Appendectomy	3,940	1.41	4,610 (4,420 to 4,810)	1.65 (1.58 to 1.72)	-668 (-862 to -477)	-0.239 (-0.309 to -0.171)	0.86 (0.82 to 0.89)
Lithuania	2021	Appendectomy	4,260	1.52	4,560 (4,290 to 4,840)	1.63 (1.53 to 1.73)	-301 (-578 to -38)	-0.108 (-0.206 to -0.014)	0.93 (0.88 to 0.99)
Lithuania	2020	Cholecystectomy	5,550	1.99	7,800 (7,020 to 8,660)	2.79 (2.51 to 3.10)	-2,240 (-3,120 to -1,470)	-0.803 (-1.11 to -0.527)	0.71 (0.64 to 0.79)
Lithuania	2021	Cholecystectomy	6,230	2.22	7,810 (7,030 to 8,680)	2.79 (2.51 to 3.10)	-1,580 (-2,450 to -812)	-0.566 (-0.873 to -0.290)	0.80 (0.72 to 0.88)
Lithuania	2020	Total mastectomy	715	0.256	758 (618 to 930)	0.271 (0.221 to 0.333)	-43 (-216 to 95)	-0.015 (-0.077 to 0.034)	0.94 (0.77 to 1.15)
Lithuania	2021	Total mastectomy	770	0.275	760 (570 to 1,010)	0.271 (0.203 to 0.362)	10 (-247 to 203)	0.004 (-0.088 to 0.073)	1.01 (0.76 to 1.36)
Lithuania	2020	Partial excision of mammary gland	1,570	0.561	2,160 (2,020 to 2,320)	0.775 (0.724 to 0.829)	-597 (-750 to -454)	-0.214 (-0.268 to -0.163)	0.72 (0.68 to 0.78)

Lithuania	2021	Partial excision of mammary gland	1,820	0.65	2,170 (2,030 to 2,320)	0.775 (0.724 to 0.829)	-349 (-501 to -204)	-0.125 (-0.179 to -0.073)	0.84 (0.78 to 0.90)
Lithuania	2020	Both	17,000	6.07	21,700 (19,800 to 23,600)	7.75 (7.10 to 8.46)	-4,680 (-6,660 to -2,860)	-1.67 (-2.38 to -1.02)	0.78 (0.72 to 0.86)
Lithuania	2021	Both	18,500	6.62	22,400 (19,800 to 25,300)	7.98 (7.05 to 9.04)	-3,810 (-6,750 to -1,180)	-1.36 (-2.41 to -0.421)	0.83 (0.73 to 0.94)
Lithuania	2020	Caesarean section	4,710	1.68	4,810 (4,360 to 5,300)	1.72 (1.56 to 1.90)	-104 (-596 to 345)	-0.037 (-0.213 to 0.123)	0.98 (0.89 to 1.08)
Lithuania	2021	Caesarean section	4,920	1.76	4,650 (4,050 to 5,340)	1.66 (1.45 to 1.91)	269 (-433 to 864)	0.096 (-0.154 to 0.309)	1.06 (0.92 to 1.21)
Lithuania	2020	Repair of inguinal hernia	3,740	1.34	5,540 (5,080 to 6,030)	1.98 (1.82 to 2.16)	-1,800 (-2,290 to -1,340)	-0.643 (-0.819 to -0.480)	0.68 (0.62 to 0.74)
Lithuania	2021	Repair of inguinal hernia	4,390	1.57	5,710 (5,060 to 6,440)	2.04 (1.81 to 2.30)	-1,320 (-2,060 to -683)	-0.473 (-0.734 to -0.244)	0.77 (0.68 to 0.87)
Lithuania	2020	Transluminal coronary angioplasty	7,380	2.64	9,430 (7,910 to 11,200)	3.37 (2.83 to 4.02)	-2,050 (-3,880 to -525)	-0.732 (-1.39 to -0.188)	0.78 (0.66 to 0.93)
Lithuania	2021	Transluminal coronary angioplasty	8,130	2.9	9,820 (6,640 to 14,500)	3.51 (2.37 to 5.19)	-1,690 (-6,510 to 1,430)	-0.604 (-2.32 to 0.512)	0.83 (0.56 to 1.21)
Lithuania	2020	Coronary artery bypass graft	1,150	0.41	1,670 (1,460 to 1,920)	0.599 (0.521 to 0.688)	-527 (-777 to -309)	-0.189 (-0.278 to -0.111)	0.69 (0.60 to 0.79)
Lithuania	2021	Coronary artery bypass graft	1,110	0.396	1,680 (1,460 to 1,930)	0.599 (0.521 to 0.688)	-568 (-823 to -349)	-0.203 (-0.294 to -0.125)	0.66 (0.57 to 0.76)
Lithuania	2020	Elective surgeries	33,800	12.1	48,700 (44,300 to 53,500)	17.4 (15.8 to 19.1)	-14,900 (-19,800 to -10,400)	-5.32 (-7.07 to -3.73)	0.69 (0.63 to 0.76)
Lithuania	2021	Elective surgeries	39,700	14.2	51,200 (44,800 to 58,600)	18.3 (16.0 to 20.9)	-11,500 (-18,800 to -5,240)	-4.11 (-6.72 to -1.87)	0.78 (0.68 to 0.88)
Lithuania	2020	Hip replacement	4,700	1.68	5,480 (3,570 to 8,400)	1.96 (1.28 to 3.01)	-779 (-3,720 to 1,120)	-0.279 (-1.33 to 0.401)	0.86 (0.56 to 1.31)
Lithuania	2021	Hip replacement	5,260	1.88	5,490 (3,000 to 10,100)	1.96 (1.07 to 3.59)	-222 (-4,710 to 2,280)	-0.079 (-1.68 to 0.814)	0.96 (0.53 to 1.76)
Lithuania	2020	Total knee replacement	1,890	0.675	3,650 (2,760 to 4,840)	1.31 (0.987 to 1.73)	-1,770 (-2,940 to -884)	-0.632 (-1.05 to -0.316)	0.52 (0.39 to 0.68)
Lithuania	2021	Total knee replacement	2,060	0.737	3,660 (2,460 to 5,440)	1.31 (0.879 to 1.94)	-1,600 (-3,330 to -374)	-0.570 (-1.19 to -0.134)	0.56 (0.38 to 0.85)
Lithuania	2020	Cataract surgery	20,300	7.27	28,000 (26,300 to 29,800)	10.0 (9.40 to 10.7)	-7,670 (-9,480 to -5,960)	-2.75 (-3.39 to -2.13)	0.73 (0.68 to 0.77)
Lithuania	2021	Cataract surgery	25,300	9.05	29,800 (27,200 to 32,500)	10.6 (9.72 to 11.6)	-4,430 (-7,210 to -1,910)	-1.58 (-2.57 to -0.681)	0.85 (0.78 to 0.93)
Lithuania	2020	Tonsillectomy	2,250	0.805	4,700 (4,240 to 5,200)	1.68 (1.52 to 1.86)	-2,450 (-2,950 to -1,980)	-0.876 (-1.06 to -0.710)	0.48 (0.43 to 0.53)
Lithuania	2021	Tonsillectomy	1,870	0.668	4,710 (4,070 to 5,440)	1.68 (1.45 to 1.94)	-2,840 (-3,570 to -2,220)	-1.01 (-1.27 to -0.791)	0.40 (0.34 to 0.46)
Lithuania	2020	Hysterectomy	3,010	1.08	4,020 (3,590 to 4,510)	1.44 (1.28 to 1.61)	-1,020 (-1,510 to -580)	-0.363 (-0.540 to -0.207)	0.75 (0.67 to 0.84)
Lithuania	2021	Hysterectomy	3,350	1.2	4,030 (3,600 to 4,520)	1.44 (1.28 to 1.61)	-682 (-1,170 to -240)	-0.244 (-0.417 to -0.086)	0.83 (0.74 to 0.93)
Lithuania	2020	Open prostatectomy	691	0.247	1,020 (796 to 1,310)	0.365 (0.285 to 0.469)	-330 (-605 to -101)	-0.118 (-0.217 to -0.036)	0.68 (0.53 to 0.87)
Lithuania	2021	Open prostatectomy	714	0.255	1,020 (798 to 1,310)	0.365 (0.285 to 0.469)	-309 (-596 to -81)	-0.110 (-0.213 to -0.029)	0.70 (0.54 to 0.90)

Lithuania	2020	Transurethral prostatectomy	985	0.352	1,450 (1,210 to 1,730)	0.518 (0.434 to 0.619)	-463 (-740 to -230)	-0.166 (-0.265 to -0.082)	0.68 (0.57 to 0.81)
Lithuania	2021	Transurethral prostatectomy	1,110	0.397	1,450 (1,130 to 1,870)	0.518 (0.403 to 0.667)	-338 (-757 to -19)	-0.121 (-0.270 to -0.007)	0.77 (0.60 to 0.98)
Luxembourg	2020	All surgeries	15,100	24	17,000 (16,000 to 18,000)	26.9 (25.4 to 28.5)	-1,830 (-2,850 to -871)	-2.90 (-4.52 to -1.38)	0.89 (0.84 to 0.95)
Luxembourg	2021	All surgeries	17,600	27.6	17,200 (16,200 to 18,300)	26.9 (25.4 to 28.5)	428 (-645 to 1,410)	0.669 (-1.01 to 2.20)	1.02 (0.96 to 1.09)
Luxembourg	2020	Non-elective surgeries	2,100	3.33	2,240 (2,090 to 2,400)	3.55 (3.31 to 3.81)	-138 (-297 to 17)	-0.218 (-0.472 to 0.026)	0.94 (0.88 to 1.01)
Luxembourg	2021	Non-elective surgeries	2,200	3.44	2,270 (2,120 to 2,440)	3.55 (3.31 to 3.81)	-72 (-237 to 84)	-0.113 (-0.371 to 0.132)	0.97 (0.90 to 1.04)
Luxembourg	2020	Appendectomy	606	0.961	630 (523 to 759)	1.00 (0.830 to 1.20)	-24 (-157 to 81)	-0.039 (-0.249 to 0.129)	0.96 (0.79 to 1.16)
Luxembourg	2021	Appendectomy	594	0.928	640 (531 to 771)	1.00 (0.830 to 1.20)	-46 (-176 to 64)	-0.072 (-0.275 to 0.100)	0.93 (0.77 to 1.12)
Luxembourg	2020	Cholecystectomy	865	1.37	959 (917 to 1,000)	1.52 (1.45 to 1.59)	-94 (-138 to -52)	-0.149 (-0.219 to -0.083)	0.90 (0.86 to 0.94)
Luxembourg	2021	Cholecystectomy	940	1.47	974 (931 to 1,020)	1.52 (1.45 to 1.59)	-34 (-79 to 9)	-0.053 (-0.123 to 0.013)	0.97 (0.92 to 1.01)
Luxembourg	2020	Total mastectomy	203	0.322	208 (186 to 233)	0.330 (0.295 to 0.370)	-5 (-30 to 17)	-0.008 (-0.048 to 0.028)	0.97 (0.87 to 1.09)
Luxembourg	2021	Total mastectomy	203	0.317	202 (176 to 231)	0.315 (0.274 to 0.362)	1 (-28 to 27)	0.002 (-0.044 to 0.042)	1.01 (0.88 to 1.15)
Luxembourg	2020	Partial excision of mammary gland	427	0.677	442 (388 to 505)	0.701 (0.615 to 0.800)	-15 (-79 to 40)	-0.024 (-0.125 to 0.063)	0.97 (0.84 to 1.10)
Luxembourg	2021	Partial excision of mammary gland	464	0.725	449 (394 to 512)	0.701 (0.615 to 0.800)	15 (-50 to 71)	0.023 (-0.078 to 0.111)	1.03 (0.90 to 1.18)
Luxembourg	2020	Both	3,630	5.75	3,970 (3,610 to 4,360)	6.30 (5.73 to 6.91)	-341 (-732 to 20)	-0.542 (-1.16 to 0.031)	0.91 (0.83 to 1.01)
Luxembourg	2021	Both	3,860	6.02	4,030 (3,530 to 4,600)	6.30 (5.51 to 7.19)	-175 (-740 to 321)	-0.273 (-1.16 to 0.502)	0.96 (0.84 to 1.09)
Luxembourg	2020	Caesarean section	1,820	2.89	1,840 (1,690 to 2,010)	2.92 (2.67 to 3.19)	-19 (-187 to 133)	-0.031 (-0.297 to 0.211)	0.99 (0.91 to 1.08)
Luxembourg	2021	Caesarean section	1,880	2.93	1,870 (1,650 to 2,120)	2.92 (2.58 to 3.31)	9 (-231 to 230)	0.015 (-0.361 to 0.359)	1.00 (0.89 to 1.14)
Luxembourg	2020	Repair of inguinal hernia	1,060	1.68	1,110 (926 to 1,340)	1.77 (1.47 to 2.12)	-56 (-289 to 133)	-0.089 (-0.458 to 0.211)	0.95 (0.79 to 1.14)
Luxembourg	2021	Repair of inguinal hernia	1,150	1.8	1,130 (940 to 1,360)	1.77 (1.47 to 2.12)	22 (-207 to 213)	0.034 (-0.324 to 0.332)	1.02 (0.85 to 1.23)
Luxembourg	2020	Transluminal coronary angioplasty	647	1.03	762 (651 to 892)	1.21 (1.03 to 1.41)	-115 (-242 to -6)	-0.182 (-0.384 to -0.010)	0.85 (0.73 to 0.99)
Luxembourg	2021	Transluminal coronary angioplasty	724	1.13	773 (619 to 966)	1.21 (0.967 to 1.51)	-49 (-240 to 106)	-0.077 (-0.375 to 0.165)	0.94 (0.75 to 1.17)
Luxembourg	2020	Coronary artery bypass graft	102	0.162	90 (68 to 119)	0.143 (0.108 to 0.189)	12 (-17 to 33)	0.019 (-0.027 to 0.053)	1.13 (0.86 to 1.49)
Luxembourg	2021	Coronary artery bypass graft	101	0.158	81 (55 to 120)	0.127 (0.086 to 0.187)	20 (-19 to 47)	0.031 (-0.030 to 0.073)	1.25 (0.84 to 1.86)
Luxembourg	2020	Elective surgeries	9,400	14.9	11,300 (10,500 to 12,200)	18.0 (16.7 to 19.4)	-1,940 (-2,830 to -1,110)	-3.08 (-4.48 to -1.76)	0.83 (0.77 to 0.89)

Luxembourg	2021	Elective surgeries	11,600	18.1	11,500 (10,300 to 12,800)	18.0 (16.1 to 20.0)	71 (-1,250 to 1,260)	0.112 (-1.95 to 1.97)	1.01 (0.90 to 1.12)
Luxembourg	2020	Hip replacement	1,010	1.6	1,150 (1,040 to 1,260)	1.82 (1.66 to 1.99)	-139 (-250 to -40)	-0.221 (-0.396 to -0.063)	0.88 (0.80 to 0.96)
Luxembourg	2021	Hip replacement	1,190	1.86	1,160 (1,060 to 1,280)	1.82 (1.66 to 1.99)	25 (-88 to 127)	0.039 (-0.137 to 0.198)	1.02 (0.93 to 1.12)
Luxembourg	2020	Total knee replacement	812	1.29	1,060 (935 to 1,200)	1.68 (1.48 to 1.90)	-246 (-388 to -126)	-0.391 (-0.615 to -0.200)	0.77 (0.68 to 0.87)
Luxembourg	2021	Total knee replacement	958	1.5	1,080 (902 to 1,280)	1.68 (1.41 to 2.00)	-117 (-327 to 59)	-0.182 (-0.512 to 0.092)	0.89 (0.75 to 1.07)
Luxembourg	2020	Cataract surgery	6,480	10.3	7,760 (7,140 to 8,430)	12.3 (11.3 to 13.4)	-1,280 (-1,940 to -660)	-2.03 (-3.08 to -1.05)	0.84 (0.77 to 0.91)
Luxembourg	2021	Cataract surgery	8,230	12.9	8,160 (7,260 to 9,180)	12.8 (11.3 to 14.3)	65 (-968 to 967)	0.102 (-1.51 to 1.51)	1.01 (0.89 to 1.13)
Luxembourg	2020	Tonsillectomy	409	0.649	635 (526 to 765)	1.01 (0.835 to 1.21)	-226 (-357 to -118)	-0.358 (-0.566 to -0.187)	0.64 (0.53 to 0.78)
Luxembourg	2021	Tonsillectomy	449	0.701	596 (457 to 776)	0.931 (0.714 to 1.21)	-147 (-324 to -7)	-0.229 (-0.507 to -0.011)	0.75 (0.58 to 0.98)
Luxembourg	2020	Hysterectomy	543	0.861	712 (612 to 828)	1.13 (0.971 to 1.31)	-169 (-287 to -70)	-0.268 (-0.455 to -0.111)	0.76 (0.65 to 0.89)
Luxembourg	2021	Hysterectomy	619	0.967	723 (584 to 895)	1.13 (0.912 to 1.40)	-104 (-276 to 36)	-0.162 (-0.431 to 0.056)	0.86 (0.69 to 1.06)
Luxembourg	2020	Open prostatectomy	147	0.233	163 (128 to 206)	0.258 (0.204 to 0.327)	-16 (-59 to 19)	-0.025 (-0.094 to 0.030)	0.90 (0.71 to 1.15)
Luxembourg	2021	Open prostatectomy	141	0.22	165 (118 to 231)	0.258 (0.185 to 0.361)	-24 (-88 to 22)	-0.038 (-0.138 to 0.035)	0.85 (0.61 to 1.19)
Norway	2020	All surgeries	98,700	18.3	107,000 (64,000 to 178,000)	19.8 (11.9 to 33.1)	-8,090 (-77,900 to 34,300)	-1.50 (-14.5 to 6.38)	0.92 (0.56 to 1.53)
Norway	2021	All surgeries	104,000	19.2	107,000 (52,100 to 221,000)	19.8 (9.63 to 40.9)	-3,370 (-116,000 to 52,200)	-0.623 (-21.5 to 9.65)	0.97 (0.47 to 2.01)
Norway	2020	Non-elective surgeries	20,400	3.79	16,300 (6,560 to 40,500)	3.03 (1.22 to 7.52)	4,100 (-19,600 to 13,900)	0.762 (-3.64 to 2.58)	1.25 (0.51 to 3.14)
Norway	2021	Non-elective surgeries	21,800	4.03	16,400 (6,600 to 40,700)	3.03 (1.22 to 7.52)	5,430 (-19,300 to 15,100)	1.00 (-3.57 to 2.79)	1.33 (0.53 to 3.24)
Norway	2020	Appendectomy	7,730	1.44	7,290 (6,850 to 7,760)	1.36 (1.27 to 1.44)	438 (-38 to 878)	0.081 (-0.007 to 0.163)	1.06 (1.00 to 1.13)
Norway	2021	Appendectomy	7,980	1.47	7,330 (6,720 to 8,010)	1.36 (1.24 to 1.48)	642 (-25 to 1,250)	0.119 (-0.005 to 0.231)	1.09 (1.00 to 1.19)
Norway	2020	Cholecystectomy	6,620	1.23	6,970 (6,540 to 7,430)	1.30 (1.22 to 1.38)	-348 (-819 to 72)	-0.065 (-0.152 to 0.013)	0.95 (0.89 to 1.01)
Norway	2021	Cholecystectomy	7,190	1.33	7,260 (6,640 to 7,950)	1.34 (1.23 to 1.47)	-74 (-762 to 555)	-0.014 (-0.141 to 0.103)	0.99 (0.90 to 1.08)
Norway	2020	Total mastectomy	1,760	0.327	1,860 (1,720 to 2,000)	0.345 (0.319 to 0.373)	-99 (-244 to 40)	-0.018 (-0.045 to 0.008)	0.95 (0.88 to 1.02)
Norway	2021	Total mastectomy	1,790	0.33	1,870 (1,670 to 2,080)	0.345 (0.309 to 0.385)	-79 (-297 to 115)	-0.015 (-0.055 to 0.021)	0.96 (0.86 to 1.07)
Norway	2020	Partial excision of mammary gland	4,290	0.797	4,630 (4,280 to 5,000)	0.861 (0.796 to 0.930)	-343 (-728 to 2)	-0.064 (-0.135 to 0.000)	0.93 (0.85 to 1.00)
Norway	2021	Partial excision of mammary gland	4,860	0.898	4,780 (4,020 to 5,690)	0.884 (0.743 to 1.05)	74 (-835 to 838)	0.014 (-0.154 to 0.155)	1.02 (0.85 to 1.21)
Norway	2020	Both	26,300	4.89	24,700 (9,490 to 64,200)	4.59 (1.76 to 11.9)	1,600 (-37,900 to 16,500)	0.298 (-7.05 to 3.07)	1.07 (0.41 to 2.70)

Norway	2021	Both	26,800	4.96	24,800 (9,540 to 64,500)	4.59 (1.76 to 11.9)	2,020 (-35,600 to 17,300)	0.373 (-6.58 to 3.19)	1.08 (0.43 to 2.81)
Norway	2020	Caesarean section	8,350	1.55	8,490 (8,100 to 8,890)	1.58 (1.51 to 1.65)	-132 (-532 to 264)	-0.025 (-0.099 to 0.049)	0.98 (0.94 to 1.03)
Norway	2021	Caesarean section	8,730	1.61	8,300 (7,780 to 8,870)	1.54 (1.44 to 1.64)	425 (-133 to 935)	0.079 (-0.025 to 0.173)	1.05 (0.98 to 1.12)
Norway	2020	Repair of inguinal hernia	5,830	1.08	6,460 (5,780 to 7,220)	1.20 (1.07 to 1.34)	-635 (-1,400 to 50)	-0.118 (-0.261 to 0.009)	0.90 (0.81 to 1.01)
Norway	2021	Repair of inguinal hernia	5,860	1.08	6,500 (5,810 to 7,260)	1.20 (1.07 to 1.34)	-639 (-1,420 to 44)	-0.118 (-0.262 to 0.008)	0.90 (0.81 to 1.01)
Norway	2020	Transluminal coronary angioplasty	10,700	2	11,700 (11,300 to 12,100)	2.18 (2.10 to 2.25)	-968 (-1,390 to -566)	-0.180 (-0.258 to -0.105)	0.92 (0.89 to 0.95)
Norway	2021	Transluminal coronary angioplasty	11,000	2.04	11,800 (11,400 to 12,200)	2.18 (2.10 to 2.25)	-738 (-1,160 to -328)	-0.136 (-0.214 to -0.061)	0.94 (0.91 to 0.97)
Norway	2020	Coronary artery bypass graft	1,370	0.255	1,440 (1,160 to 1,800)	0.269 (0.215 to 0.335)	-74 (-425 to 210)	-0.014 (-0.079 to 0.039)	0.95 (0.76 to 1.18)
Norway	2021	Coronary artery bypass graft	1,220	0.225	1,450 (1,060 to 1,990)	0.269 (0.196 to 0.367)	-235 (-769 to 162)	-0.043 (-0.142 to 0.030)	0.84 (0.61 to 1.15)
Norway	2020	Elective surgeries	52,000	9.66	58,200 (41,300 to 82,000)	10.8 (7.67 to 15.3)	-6,220 (-29,900 to 10,600)	-1.16 (-5.56 to 1.98)	0.89 (0.63 to 1.26)
Norway	2021	Elective surgeries	55,300	10.2	58,500 (36,000 to 95,100)	10.8 (6.66 to 17.6)	-3,190 (-40,400 to 19,500)	-0.590 (-7.48 to 3.61)	0.95 (0.58 to 1.55)
Norway	2020	Hip replacement	12,900	2.4	14,400 (13,800 to 15,000)	2.67 (2.56 to 2.79)	-1,500 (-2,120 to -892)	-0.278 (-0.394 to -0.166)	0.90 (0.86 to 0.94)
Norway	2021	Hip replacement	13,800	2.55	14,500 (13,600 to 15,400)	2.67 (2.52 to 2.84)	-658 (-1,550 to 190)	-0.122 (-0.286 to 0.035)	0.95 (0.90 to 1.01)
Norway	2020	Total knee replacement	5,800	1.08	6,320 (5,260 to 7,580)	1.17 (0.978 to 1.41)	-516 (-1,770 to 535)	-0.096 (-0.329 to 0.099)	0.92 (0.77 to 1.10)
Norway	2021	Total knee replacement	6,570	1.22	6,350 (4,900 to 8,220)	1.17 (0.907 to 1.52)	222 (-1,620 to 1,650)	0.041 (-0.300 to 0.305)	1.03 (0.80 to 1.34)
Norway	2020	Cataract surgery	19,200	3.57	21,200 (19,000 to 23,600)	3.94 (3.54 to 4.39)	-2,010 (-4,390 to 153)	-0.374 (-0.815 to 0.028)	0.91 (0.81 to 1.01)
Norway	2021	Cataract surgery	21,500	3.98	21,300 (19,100 to 23,800)	3.94 (3.54 to 4.39)	177 (-2,220 to 2,410)	0.033 (-0.411 to 0.446)	1.01 (0.91 to 1.13)
Norway	2020	Tonsillectomy	6,590	1.22	8,290 (6,300 to 10,900)	1.54 (1.17 to 2.02)	-1,700 (-4,200 to 283)	-0.315 (-0.781 to 0.053)	0.80 (0.61 to 1.04)
Norway	2021	Tonsillectomy	5,820	1.08	8,330 (5,660 to 12,300)	1.54 (1.05 to 2.27)	-2,510 (-6,510 to 174)	-0.464 (-1.20 to 0.032)	0.70 (0.47 to 1.03)
Norway	2020	Hysterectomy	4,610	0.856	4,870 (4,190 to 5,670)	0.906 (0.778 to 1.05)	-268 (-1,050 to 416)	-0.050 (-0.196 to 0.077)	0.95 (0.81 to 1.10)
Norway	2021	Hysterectomy	4,660	0.861	4,900 (3,950 to 6,070)	0.906 (0.731 to 1.12)	-243 (-1,410 to 715)	-0.045 (-0.261 to 0.132)	0.95 (0.77 to 1.18)
Norway	2020	Open prostatectomy	110	0.02	148 (32 to 684)	0.027 (0.006 to 0.127)	-38 (-574 to 78)	-0.007 (-0.107 to 0.015)	0.74 (0.16 to 3.49)
Norway	2021	Open prostatectomy	121	0.022	149 (17 to 1,300)	0.027 (0.003 to 0.240)	-28 (-1,140 to 104)	-0.005 (-0.210 to 0.019)	0.81 (0.10 to 7.20)
Norway	2020	Transurethral prostatectomy	2,780	0.517	2,750 (2,560 to 2,950)	0.511 (0.476 to 0.548)	34 (-165 to 221)	0.006 (-0.031 to 0.041)	1.01 (0.94 to 1.09)
Norway	2021	Transurethral prostatectomy	2,840	0.525	2,660 (2,410 to 2,940)	0.492 (0.445 to 0.543)	178 (-97 to 428)	0.033 (-0.018 to 0.079)	1.07 (0.97 to 1.18)
New Zealand	2020	All surgeries	88,400	17.4	91,500 (88,200 to 95,000)	18.0 (17.3 to 18.7)	-3,060 (-6,490 to 191)	-0.600 (-1.27 to 0.037)	0.97 (0.93 to 1.00)

New Zealand	2020	Non-elective surgeries	17,700	3.47	17,700 (17,000 to 18,600)	3.48 (3.33 to 3.65)	-59 (-876 to 720)	-0.012 (-0.172 to 0.141)	1.00 (0.95 to 1.04)
New Zealand	2020	Appendectomy	6,410	1.26	6,450 (6,130 to 6,780)	1.27 (1.20 to 1.33)	-39 (-370 to 283)	-0.008 (-0.073 to 0.056)	0.99 (0.95 to 1.05)
New Zealand	2020	Cholecystectomy	6,760	1.33	6,560 (6,230 to 6,910)	1.29 (1.22 to 1.36)	200 (-150 to 533)	0.039 (-0.029 to 0.105)	1.03 (0.98 to 1.09)
New Zealand	2020	Total mastectomy	1,620	0.319	1,700 (1,560 to 1,860)	0.335 (0.306 to 0.366)	-81 (-239 to 63)	-0.016 (-0.047 to 0.012)	0.95 (0.87 to 1.04)
New Zealand	2020	Partial excision of mammary gland	2,890	0.568	2,980 (2,730 to 3,250)	0.585 (0.536 to 0.639)	-91 (-371 to 159)	-0.018 (-0.073 to 0.031)	0.97 (0.89 to 1.06)
New Zealand	2020	Both	29,100	5.72	30,000 (28,800 to 31,300)	5.90 (5.65 to 6.15)	-897 (-2,220 to 329)	-0.176 (-0.436 to 0.065)	0.97 (0.93 to 1.01)
New Zealand	2020	Caesarean section	17,000	3.35	17,200 (16,300 to 18,100)	3.38 (3.20 to 3.56)	-147 (-1,120 to 777)	-0.029 (-0.219 to 0.153)	0.99 (0.94 to 1.05)
New Zealand	2020	Repair of inguinal hernia	4,480	0.881	5,090 (4,390 to 5,900)	1.00 (0.862 to 1.16)	-608 (-1,430 to 100)	-0.119 (-0.280 to 0.020)	0.88 (0.76 to 1.02)
New Zealand	2020	Transluminal coronary angioplasty	6,100	1.2	6,540 (6,080 to 7,020)	1.28 (1.20 to 1.38)	-433 (-924 to 27)	-0.085 (-0.182 to 0.005)	0.93 (0.87 to 1.00)
New Zealand	2020	Coronary artery bypass graft	1,500	0.295	1,560 (1,440 to 1,700)	0.307 (0.283 to 0.334)	-62 (-199 to 63)	-0.012 (-0.039 to 0.012)	0.96 (0.88 to 1.04)
New Zealand	2020	Elective surgeries	41,600	8.18	44,600 (42,800 to 46,600)	8.77 (8.40 to 9.16)	-3,000 (-4,970 to -1,130)	-0.588 (-0.977 to -0.222)	0.93 (0.89 to 0.97)
New Zealand	2020	Hip replacement	7,480	1.47	8,120 (7,540 to 8,760)	1.60 (1.48 to 1.72)	-648 (-1,280 to -59)	-0.127 (-0.252 to -0.012)	0.92 (0.85 to 0.99)
New Zealand	2020	Total knee replacement	5,220	1.02	5,560 (4,860 to 6,360)	1.09 (0.954 to 1.25)	-339 (-1,150 to 360)	-0.067 (-0.225 to 0.071)	0.94 (0.82 to 1.07)
New Zealand	2020	Cataract surgery	19,000	3.73	20,900 (19,800 to 22,100)	4.11 (3.90 to 4.34)	-1,970 (-3,140 to -849)	-0.387 (-0.616 to -0.167)	0.91 (0.86 to 0.96)
New Zealand	2020	Tonsillectomy	4,080	0.802	5,620 (5,000 to 6,320)	1.10 (0.982 to 1.24)	-1,540 (-2,260 to -936)	-0.303 (-0.443 to -0.184)	0.73 (0.64 to 0.81)
New Zealand	2020	Hysterectomy	3,250	0.639	3,220 (3,020 to 3,440)	0.633 (0.592 to 0.676)	30 (-195 to 235)	0.006 (-0.038 to 0.046)	1.01 (0.94 to 1.08)
New Zealand	2020	Open prostatectomy	2,660	0.522	1,680 (1,090 to 2,580)	0.329 (0.214 to 0.507)	979 (62 to 1,580)	0.192 (0.012 to 0.310)	1.58 (1.02 to 2.47)
Poland	2020	All surgeries	658,000	17.4	926,000 (848,000 to 1,010,000)	24.4 (22.4 to 26.7)	-268,000 (-355,000 to -190,000)	-7.08 (-9.38 to -5.02)	0.71 (0.65 to 0.78)
Poland	2021	All surgeries	774,000	20.5	978,000 (862,000 to 1,110,000)	25.9 (22.8 to 29.4)	-204,000 (-333,000 to -89,200)	-5.40 (-8.82 to -2.36)	0.79 (0.70 to 0.90)
Poland	2020	Non-elective surgeries	86,800	2.29	115,000 (106,000 to 125,000)	3.03 (2.79 to 3.29)	-28,000 (-37,900 to -18,900)	-0.738 (-0.999 to -0.498)	0.76 (0.70 to 0.82)
Poland	2021	Non-elective surgeries	107,000	2.84	114,000 (102,000 to 129,000)	3.03 (2.69 to 3.41)	-7,110 (-21,900 to 5,580)	-0.188 (-0.580 to 0.148)	0.94 (0.83 to 1.05)
Poland	2020	Appendectomy	22,300	0.589	28,200 (26,600 to 29,800)	0.743 (0.701 to 0.787)	-5,820 (-7,510 to -4,250)	-0.154 (-0.198 to -0.112)	0.79 (0.75 to 0.84)
Poland	2021	Appendectomy	26,800	0.711	28,000 (26,500 to 29,700)	0.743 (0.701 to 0.787)	-1,210 (-2,860 to 348)	-0.032 (-0.076 to 0.009)	0.96 (0.90 to 1.01)
Poland	2020	Cholecystectomy	51,600	1.36	72,500 (65,500 to 80,300)	1.91 (1.73 to 2.12)	-21,000 (-28,600 to -13,800)	-0.553 (-0.755 to -0.365)	0.71 (0.64 to 0.79)

Poland	2021	Cholecystectomy	64,600	1.71	72,200 (62,500 to 83,400)	1.91 (1.66 to 2.21)	-7,630 (-18,800 to 1,840)	-0.202 (-0.498 to 0.049)	0.89 (0.77 to 1.03)
Poland	2020	Total mastectomy	8,430	0.222	8,890 (8,320 to 9,500)	0.235 (0.220 to 0.251)	-464 (-1,070 to 107)	-0.012 (-0.028 to 0.003)	0.95 (0.89 to 1.01)
Poland	2021	Total mastectomy	10,400	0.275	9,180 (8,570 to 9,820)	0.243 (0.227 to 0.260)	1,190 (539 to 1,800)	0.032 (0.014 to 0.048)	1.13 (1.05 to 1.21)
Poland	2020	Partial excision of mammary gland	4,490	0.118	6,230 (5,270 to 7,370)	0.164 (0.139 to 0.195)	-1,750 (-2,900 to -785)	-0.046 (-0.076 to -0.021)	0.72 (0.61 to 0.85)
Poland	2021	Partial excision of mammary gland	5,410	0.143	6,210 (4,900 to 7,870)	0.164 (0.130 to 0.208)	-799 (-2,470 to 517)	-0.021 (-0.065 to 0.014)	0.87 (0.69 to 1.11)
Poland	2020	Both	273,000	7.21	312,000 (281,000 to 347,000)	8.24 (7.42 to 9.16)	-39,300 (-74,500 to -8,530)	-1.04 (-1.97 to -0.225)	0.87 (0.79 to 0.97)
Poland	2021	Both	300,000	7.96	304,000 (240,000 to 384,000)	8.05 (6.37 to 10.2)	-3,360 (-84,000 to 60,800)	-0.089 (-2.22 to 1.61)	0.99 (0.78 to 1.25)
Poland	2020	Caesarean section	139,000	3.68	151,000 (137,000 to 166,000)	3.98 (3.61 to 4.39)	-11,500 (-26,900 to 2,400)	-0.304 (-0.709 to 0.063)	0.92 (0.84 to 1.02)
Poland	2021	Caesarean section	140,000	3.71	156,000 (136,000 to 178,000)	4.12 (3.59 to 4.73)	-15,700 (-38,200 to 4,350)	-0.415 (-1.01 to 0.115)	0.90 (0.79 to 1.03)
Poland	2020	Repair of inguinal hernia	41,300	1.09	58,600 (54,000 to 63,500)	1.54 (1.42 to 1.68)	-17,200 (-22,200 to -12,700)	-0.455 (-0.585 to -0.335)	0.71 (0.65 to 0.77)
Poland	2021	Repair of inguinal hernia	50,900	1.35	58,300 (52,000 to 65,400)	1.54 (1.38 to 1.73)	-7,370 (-14,300 to -988)	-0.195 (-0.379 to -0.026)	0.87 (0.78 to 0.98)
Poland	2020	Transluminal coronary angioplasty	80,600	2.13	97,400 (82,600 to 115,000)	2.57 (2.18 to 3.03)	-16,800 (-34,400 to -1,880)	-0.443 (-0.908 to -0.050)	0.83 (0.70 to 0.98)
Poland	2021	Transluminal coronary angioplasty	96,500	2.56	96,300 (66,600 to 139,000)	2.55 (1.76 to 3.69)	177 (-43,600 to 30,200)	0.005 (-1.16 to 0.801)	1.00 (0.69 to 1.46)
Poland	2020	Coronary artery bypass graft	11,800	0.311	19,300 (16,300 to 22,800)	0.509 (0.431 to 0.601)	-7,480 (-10,900 to -4,490)	-0.197 (-0.289 to -0.119)	0.61 (0.52 to 0.72)
Poland	2021	Coronary artery bypass graft	13,100	0.348	19,200 (16,300 to 22,700)	0.509 (0.431 to 0.601)	-6,090 (-9,690 to -3,200)	-0.161 (-0.257 to -0.085)	0.68 (0.58 to 0.80)
Poland	2020	Elective surgeries	298,000	7.87	478,000 (433,000 to 528,000)	12.6 (11.4 to 13.9)	-180,000 (-230,000 to -135,000)	-4.75 (-6.06 to -3.56)	0.62 (0.57 to 0.69)
Poland	2021	Elective surgeries	366,000	9.7	518,000 (451,000 to 596,000)	13.7 (11.9 to 15.8)	-152,000 (-228,000 to -84,900)	-4.04 (-6.04 to -2.25)	0.71 (0.62 to 0.81)
Poland	2020	Hip replacement	53,200	1.4	76,200 (66,900 to 86,800)	2.01 (1.77 to 2.29)	-23,000 (-33,700 to -13,800)	-0.608 (-0.890 to -0.365)	0.70 (0.61 to 0.79)
Poland	2021	Hip replacement	69,100	1.83	85,200 (70,800 to 102,000)	2.26 (1.88 to 2.71)	-16,100 (-33,000 to -1,760)	-0.425 (-0.873 to -0.047)	0.81 (0.68 to 0.98)
Poland	2020	Total knee replacement	20,800	0.548	33,600 (28,300 to 39,800)	0.885 (0.746 to 1.05)	-12,800 (-19,100 to -7,540)	-0.338 (-0.505 to -0.199)	0.62 (0.52 to 0.73)
Poland	2021	Total knee replacement	26,400	0.698	39,400 (30,900 to 50,200)	1.04 (0.819 to 1.33)	-13,100 (-24,000 to -4,340)	-0.346 (-0.635 to -0.115)	0.67 (0.52 to 0.86)

Poland	2020	Cataract surgery	168,000	4.43	289,000 (252,000 to 331,000)	7.62 (6.65 to 8.74)	-121,000 (-164,000 to -84,000)	-3.20 (-4.32 to -2.22)	0.58 (0.51 to 0.67)
Poland	2021	Cataract surgery	199,000	5.28	318,000 (262,000 to 386,000)	8.43 (6.95 to 10.2)	-119,000 (-186,000 to -61,700)	-3.14 (-4.94 to -1.64)	0.63 (0.52 to 0.76)
Poland	2020	Tonsillectomy	12,100	0.32	24,300 (22,400 to 26,400)	0.642 (0.592 to 0.696)	-12,200 (-14,200 to -10,300)	-0.322 (-0.375 to -0.272)	0.50 (0.46 to 0.54)
Poland	2021	Tonsillectomy	14,900	0.394	24,500 (22,100 to 27,100)	0.648 (0.586 to 0.717)	-9,590 (-12,200 to -7,270)	-0.254 (-0.323 to -0.193)	0.61 (0.55 to 0.67)
Poland	2020	Hysterectomy	28,700	0.757	37,400 (34,000 to 41,100)	0.986 (0.897 to 1.08)	-8,660 (-12,300 to -5,230)	-0.228 (-0.324 to -0.138)	0.77 (0.70 to 0.85)
Poland	2021	Hysterectomy	36,000	0.954	37,300 (33,200 to 42,000)	0.989 (0.880 to 1.11)	-1,350 (-6,000 to 2,750)	-0.036 (-0.159 to 0.073)	0.96 (0.86 to 1.08)
Poland	2020	Open prostatectomy	6,490	0.171	8,240 (7,060 to 9,610)	0.217 (0.186 to 0.253)	-1,740 (-3,120 to -595)	-0.046 (-0.082 to -0.016)	0.79 (0.68 to 0.92)
Poland	2021	Open prostatectomy	8,070	0.214	8,880 (7,140 to 11,000)	0.235 (0.189 to 0.293)	-811 (-2,980 to 918)	-0.021 (-0.079 to 0.024)	0.91 (0.73 to 1.13)
Poland	2020	Transurethral prostatectomy	9,260	0.244	13,800 (12,300 to 15,500)	0.364 (0.324 to 0.409)	-4,540 (-6,220 to -3,040)	-0.120 (-0.164 to -0.080)	0.67 (0.60 to 0.75)
Poland	2021	Transurethral prostatectomy	12,100	0.321	14,400 (11,100 to 18,700)	0.382 (0.295 to 0.496)	-2,340 (-6,530 to 923)	-0.062 (-0.173 to 0.024)	0.84 (0.65 to 1.08)
Portugal	2020	All surgeries	155,000	15.1	192,000 (180,000 to 205,000)	18.6 (17.5 to 19.9)	-36,700 (-49,700 to -24,700)	-3.57 (-4.82 to -2.40)	0.81 (0.76 to 0.86)
Portugal	2021	All surgeries	190,000	18.3	193,000 (176,000 to 212,000)	18.6 (17.0 to 20.4)	-3,090 (-21,200 to 13,600)	-0.299 (-2.05 to 1.31)	0.98 (0.90 to 1.08)
Portugal	2020	Non-elective surgeries	30,000	2.92	35,900 (33,700 to 38,300)	3.49 (3.27 to 3.72)	-5,900 (-8,310 to -3,590)	-0.573 (-0.807 to -0.349)	0.84 (0.78 to 0.89)
Portugal	2021	Non-elective surgeries	33,300	3.22	36,200 (33,000 to 39,600)	3.49 (3.18 to 3.82)	-2,830 (-6,300 to 287)	-0.273 (-0.608 to 0.028)	0.92 (0.84 to 1.01)
Portugal	2020	Appendectomy	7,540	0.732	8,560 (8,140 to 9,010)	0.832 (0.790 to 0.875)	-1,030 (-1,480 to -605)	-0.100 (-0.144 to -0.059)	0.88 (0.84 to 0.93)
Portugal	2021	Appendectomy	7,820	0.754	8,430 (7,850 to 9,060)	0.814 (0.757 to 0.875)	-616 (-1,240 to -24)	-0.059 (-0.119 to -0.002)	0.93 (0.86 to 1.00)
Portugal	2020	Cholecystectomy	12,900	1.26	16,300 (14,800 to 18,000)	1.58 (1.44 to 1.75)	-3,390 (-5,000 to -1,900)	-0.329 (-0.485 to -0.185)	0.79 (0.72 to 0.87)
Portugal	2021	Cholecystectomy	14,700	1.42	16,400 (14,900 to 18,100)	1.58 (1.44 to 1.75)	-1,700 (-3,370 to -153)	-0.164 (-0.325 to -0.015)	0.90 (0.81 to 0.99)
Portugal	2020	Total mastectomy	3,320	0.322	3,620 (3,210 to 4,080)	0.351 (0.311 to 0.396)	-301 (-755 to 111)	-0.029 (-0.073 to 0.011)	0.92 (0.81 to 1.03)
Portugal	2021	Total mastectomy	3,620	0.349	3,640 (3,230 to 4,110)	0.351 (0.311 to 0.396)	-21 (-485 to 390)	-0.002 (-0.047 to 0.038)	0.99 (0.88 to 1.12)
Portugal	2020	Partial excision of mammary gland	6,260	0.608	6,860 (6,400 to 7,350)	0.666 (0.622 to 0.714)	-603 (-1,100 to -147)	-0.059 (-0.106 to -0.014)	0.91 (0.85 to 0.98)
Portugal	2021	Partial excision of mammary gland	7,170	0.692	6,900 (6,440 to 7,400)	0.666 (0.622 to 0.714)	269 (-230 to 732)	0.026 (-0.022 to 0.071)	1.04 (0.97 to 1.11)
Portugal	2020	Both	25,500	2.48	31,200 (29,500 to 32,900)	3.03 (2.87 to 3.20)	-5,650 (-7,370 to -4,000)	-0.548 (-0.716 to -0.388)	0.82 (0.78 to 0.86)
Portugal	2021	Both	26,800	2.59	31,400 (29,700 to 33,100)	3.03 (2.87 to 3.20)	-4,560 (-6,310 to -2,900)	-0.440 (-0.609 to -0.280)	0.85 (0.81 to 0.90)

Portugal	2020	Repair of inguinal hernia	12,700	1.23	16,200 (14,700 to 17,800)	1.57 (1.43 to 1.73)	-3,530 (-5,160 to -2,030)	-0.343 (-0.501 to -0.198)	0.78 (0.71 to 0.86)
Portugal	2021	Repair of inguinal hernia	14,200	1.37	16,300 (14,200 to 18,700)	1.57 (1.38 to 1.80)	-2,140 (-4,440 to -65)	-0.207 (-0.429 to -0.006)	0.87 (0.76 to 1.00)
Portugal	2020	Transluminal coronary angioplasty	10,800	1.05	12,600 (11,700 to 13,600)	1.22 (1.13 to 1.32)	-1,780 (-2,780 to -865)	-0.172 (-0.270 to -0.084)	0.86 (0.80 to 0.93)
Portugal	2021	Transluminal coronary angioplasty	10,300	0.997	12,700 (11,700 to 13,800)	1.23 (1.13 to 1.33)	-2,390 (-3,460 to -1,400)	-0.230 (-0.334 to -0.135)	0.81 (0.75 to 0.88)
Portugal	2020	Coronary artery bypass graft	2,010	0.195	2,170 (2,110 to 2,230)	0.210 (0.205 to 0.216)	-157 (-216 to -97)	-0.015 (-0.021 to -0.009)	0.93 (0.90 to 0.95)
Portugal	2021	Coronary artery bypass graft	2,310	0.223	2,140 (2,060 to 2,220)	0.206 (0.198 to 0.215)	168 (85 to 250)	0.016 (0.008 to 0.024)	1.08 (1.04 to 1.12)
Portugal	2020	Elective surgeries	99,700	9.68	125,000 (114,000 to 137,000)	12.1 (11.0 to 13.3)	-25,300 (-37,400 to -13,900)	-2.45 (-3.63 to -1.35)	0.80 (0.73 to 0.88)
Portugal	2021	Elective surgeries	130,000	12.5	126,000 (110,000 to 144,000)	12.1 (10.6 to 13.9)	4,200 (-14,000 to 20,000)	0.406 (-1.35 to 1.93)	1.03 (0.90 to 1.18)
Portugal	2020	Hip replacement	8,920	0.867	10,600 (9,870 to 11,400)	1.03 (0.958 to 1.10)	-1,660 (-2,420 to -956)	-0.161 (-0.235 to -0.093)	0.84 (0.79 to 0.90)
Portugal	2021	Hip replacement	11,200	1.08	10,700 (9,650 to 11,800)	1.03 (0.931 to 1.13)	567 (-544 to 1,560)	0.055 (-0.052 to 0.151)	1.05 (0.95 to 1.16)
Portugal	2020	Total knee replacement	5,080	0.493	6,610 (5,650 to 7,730)	0.642 (0.549 to 0.751)	-1,530 (-2,670 to -553)	-0.148 (-0.259 to -0.054)	0.77 (0.66 to 0.90)
Portugal	2021	Total knee replacement	7,810	0.754	6,650 (5,680 to 7,780)	0.642 (0.549 to 0.751)	1,160 (23 to 2,120)	0.112 (0.002 to 0.205)	1.17 (1.00 to 1.37)
Portugal	2020	Cataract surgery	71,000	6.9	88,300 (76,400 to 102,000)	8.58 (7.42 to 9.93)	-17,300 (-31,000 to -5,170)	-1.68 (-3.01 to -0.502)	0.80 (0.70 to 0.93)
Portugal	2021	Cataract surgery	94,900	9.16	88,900 (72,300 to 109,000)	8.58 (6.98 to 10.5)	5,980 (-14,800 to 22,700)	0.577 (-1.43 to 2.19)	1.07 (0.86 to 1.32)
Portugal	2020	Tonsillectomy	5,130	0.499	7,020 (6,450 to 7,630)	0.681 (0.626 to 0.741)	-1,880 (-2,500 to -1,330)	-0.183 (-0.243 to -0.129)	0.73 (0.67 to 0.79)
Portugal	2021	Tonsillectomy	5,380	0.52	6,800 (6,040 to 7,660)	0.656 (0.583 to 0.739)	-1,410 (-2,270 to -654)	-0.136 (-0.219 to -0.063)	0.79 (0.70 to 0.89)
Portugal	2020	Hysterectomy	5,770	0.56	7,170 (6,700 to 7,660)	0.696 (0.651 to 0.744)	-1,400 (-1,890 to -939)	-0.136 (-0.184 to -0.091)	0.80 (0.75 to 0.86)
Portugal	2021	Hysterectomy	6,270	0.605	6,940 (6,320 to 7,630)	0.670 (0.609 to 0.737)	-670 (-1,360 to -49)	-0.065 (-0.131 to -0.005)	0.90 (0.82 to 0.99)
Portugal	2020	Open prostatectomy	1,930	0.188	2,200 (1,950 to 2,470)	0.213 (0.190 to 0.240)	-262 (-537 to -17)	-0.025 (-0.052 to -0.002)	0.88 (0.78 to 0.99)
Portugal	2021	Open prostatectomy	2,050	0.198	2,210 (1,870 to 2,610)	0.213 (0.181 to 0.252)	-157 (-560 to 181)	-0.015 (-0.054 to 0.017)	0.93 (0.79 to 1.10)
Portugal	2020	Transurethral prostatectomy	1,790	0.174	2,140 (1,920 to 2,400)	0.208 (0.186 to 0.233)	-351 (-604 to -131)	-0.034 (-0.059 to -0.013)	0.84 (0.75 to 0.93)
Portugal	2021	Transurethral prostatectomy	2,320	0.224	2,160 (1,840 to 2,520)	0.208 (0.178 to 0.244)	167 (-198 to 485)	0.016 (-0.019 to 0.047)	1.08 (0.92 to 1.26)
Slovak Republic	2020	All surgeries	89,700	16.4	116,000 (108,000 to 125,000)	21.3 (19.7 to 23.0)	-26,500 (-35,700 to -17,800)	-4.86 (-6.55 to -3.26)	0.77 (0.72 to 0.83)
Slovak Republic	2021	All surgeries	88,900	16.3	120,000 (107,000 to 133,000)	22.0 (19.7 to 24.5)	-30,800 (-44,700 to -18,400)	-5.65 (-8.20 to -3.38)	0.74 (0.67 to 0.83)

Slovak Republic	2020	Non-elective surgeries	10,500	1.92	14,200 (12,600 to 15,900)	2.60 (2.32 to 2.92)	-3,700 (-5,430 to -2,130)	-0.677 (-0.994 to -0.390)	0.74 (0.66 to 0.83)
Slovak Republic	2021	Non-elective surgeries	9,910	1.82	14,200 (12,600 to 15,900)	2.60 (2.32 to 2.92)	-4,260 (-6,000 to -2,700)	-0.781 (-1.10 to -0.496)	0.70 (0.62 to 0.79)
Slovak Republic	2020	Cholecystectomy	9,350	1.71	11,700 (10,400 to 13,200)	2.14 (1.90 to 2.41)	-2,330 (-3,820 to -1,010)	-0.428 (-0.699 to -0.185)	0.80 (0.71 to 0.90)
Slovak Republic	2021	Cholecystectomy	9,060	1.66	11,700 (9,840 to 13,800)	2.14 (1.81 to 2.53)	-2,600 (-4,760 to -790)	-0.477 (-0.875 to -0.145)	0.78 (0.66 to 0.92)
Slovak Republic	2020	Total mastectomy	1,150	0.211	1,160 (885 to 1,510)	0.212 (0.162 to 0.277)	-2 (-356 to 273)	-0.000 (-0.065 to 0.050)	1.00 (0.76 to 1.31)
Slovak Republic	2021	Total mastectomy	854	0.157	1,150 (883 to 1,510)	0.212 (0.162 to 0.277)	-299 (-661 to -33)	-0.055 (-0.121 to -0.006)	0.74 (0.56 to 0.96)
Slovak Republic	2020	Both	27,400	5.03	29,600 (28,400 to 30,800)	5.42 (5.20 to 5.65)	-2,150 (-3,400 to -944)	-0.393 (-0.623 to -0.173)	0.93 (0.89 to 0.97)
Slovak Republic	2021	Both	27,800	5.1	28,500 (26,000 to 31,200)	5.23 (4.77 to 5.72)	-669 (-3,350 to 1,800)	-0.123 (-0.615 to 0.330)	0.98 (0.89 to 1.07)
Slovak Republic	2020	Caesarean section	16,700	3.05	16,700 (16,100 to 17,300)	3.06 (2.96 to 3.18)	-66 (-679 to 521)	-0.012 (-0.124 to 0.095)	1.00 (0.96 to 1.03)
Slovak Republic	2021	Caesarean section	16,700	3.07	16,800 (16,000 to 17,600)	3.08 (2.94 to 3.23)	-36 (-832 to 723)	-0.007 (-0.153 to 0.133)	1.00 (0.95 to 1.05)
Slovak Republic	2020	Repair of inguinal hernia	8,920	1.63	11,900 (10,500 to 13,400)	2.18 (1.93 to 2.46)	-2,980 (-4,500 to -1,640)	-0.545 (-0.825 to -0.300)	0.75 (0.66 to 0.84)
Slovak Republic	2021	Repair of inguinal hernia	9,120	1.68	11,900 (10,200 to 13,800)	2.18 (1.87 to 2.54)	-2,750 (-4,740 to -1,070)	-0.505 (-0.870 to -0.196)	0.77 (0.66 to 0.90)
Slovak Republic	2020	Coronary artery bypass graft	1,870	0.342	2,050 (1,420 to 2,960)	0.376 (0.261 to 0.542)	-185 (-1,110 to 445)	-0.034 (-0.204 to 0.082)	0.91 (0.63 to 1.31)
Slovak Republic	2021	Coronary artery bypass graft	1,940	0.357	2,050 (1,220 to 3,430)	0.376 (0.224 to 0.630)	-104 (-1,460 to 732)	-0.019 (-0.268 to 0.134)	0.95 (0.57 to 1.61)
Slovak Republic	2020	Elective surgeries	51,700	9.47	72,700 (65,400 to 80,700)	13.3 (12.0 to 14.8)	-20,900 (-29,000 to -13,700)	-3.84 (-5.32 to -2.50)	0.71 (0.64 to 0.79)
Slovak Republic	2021	Elective surgeries	51,200	9.4	76,300 (65,800 to 88,500)	14.0 (12.1 to 16.2)	-25,100 (-37,500 to -14,500)	-4.61 (-6.89 to -2.66)	0.67 (0.58 to 0.78)
Slovak Republic	2020	Hip replacement	5,710	1.05	6,880 (5,990 to 7,920)	1.26 (1.10 to 1.45)	-1,180 (-2,200 to -292)	-0.216 (-0.404 to -0.053)	0.83 (0.72 to 0.95)
Slovak Republic	2021	Hip replacement	5,380	0.987	6,790 (4,970 to 9,280)	1.25 (0.913 to 1.70)	-1,420 (-3,860 to 388)	-0.261 (-0.708 to 0.071)	0.79 (0.58 to 1.08)
Slovak Republic	2020	Total knee replacement	3,930	0.72	5,180 (4,390 to 6,130)	0.950 (0.804 to 1.12)	-1,260 (-2,220 to -467)	-0.230 (-0.406 to -0.086)	0.76 (0.64 to 0.89)
Slovak Republic	2021	Total knee replacement	2,680	0.492	4,900 (3,370 to 7,120)	0.900 (0.619 to 1.31)	-2,220 (-4,460 to -689)	-0.408 (-0.818 to -0.126)	0.55 (0.38 to 0.80)
Slovak Republic	2020	Cataract surgery	34,800	6.38	51,100 (43,900 to 59,500)	9.36 (8.04 to 10.9)	-16,300 (-24,700 to -8,900)	-2.98 (-4.52 to -1.63)	0.68 (0.59 to 0.80)
Slovak Republic	2021	Cataract surgery	36,800	6.75	54,400 (43,800 to 67,400)	9.98 (8.05 to 12.4)	-17,600 (-30,300 to -7,060)	-3.23 (-5.56 to -1.30)	0.68 (0.55 to 0.84)
Slovak Republic	2020	Hysterectomy	5,280	0.967	5,980 (5,580 to 6,420)	1.10 (1.02 to 1.18)	-704 (-1,130 to -301)	-0.129 (-0.208 to -0.055)	0.88 (0.82 to 0.95)
Slovak Republic	2021	Hysterectomy	4,640	0.851	5,780 (5,240 to 6,370)	1.06 (0.961 to 1.17)	-1,140 (-1,720 to -598)	-0.209 (-0.317 to -0.110)	0.80 (0.73 to 0.89)
Slovak Republic	2020	Open prostatectomy	339	0.062	457 (344 to 607)	0.084 (0.063 to 0.111)	-118 (-268 to -5)	-0.022 (-0.049 to -0.001)	0.74 (0.56 to 0.98)

Slovak Republic	2021	Open prostatectomy	332	0.061	456 (344 to 606)	0.084 (0.063 to 0.111)	-124 (-272 to -12)	-0.023 (-0.050 to -0.002)	0.73 (0.55 to 0.97)
Slovak Republic	2020	Transurethral prostatectomy	1,630	0.299	2,100 (1,780 to 2,470)	0.385 (0.327 to 0.453)	-467 (-840 to -150)	-0.085 (-0.154 to -0.028)	0.78 (0.66 to 0.92)
Slovak Republic	2021	Transurethral prostatectomy	1,380	0.254	2,100 (1,780 to 2,470)	0.385 (0.327 to 0.453)	-713 (-1,080 to -404)	-0.131 (-0.198 to -0.074)	0.66 (0.56 to 0.77)
Slovenia	2020	All surgeries	47,700	22.7	55,200 (51,200 to 59,600)	26.3 (24.4 to 28.3)	-7,510 (-11,700 to -3,380)	-3.57 (-5.59 to -1.61)	0.86 (0.80 to 0.93)
Slovenia	2021	All surgeries	49,600	23.5	55,400 (49,800 to 61,700)	26.3 (23.6 to 29.2)	-5,780 (-11,900 to -169)	-2.74 (-5.66 to -0.080)	0.90 (0.81 to 1.00)
Slovenia	2020	Non-elective surgeries	8,300	3.95	9,530 (8,840 to 10,300)	4.53 (4.20 to 4.89)	-1,240 (-1,970 to -526)	-0.588 (-0.936 to -0.250)	0.87 (0.81 to 0.94)
Slovenia	2021	Non-elective surgeries	8,550	4.06	9,560 (8,590 to 10,600)	4.53 (4.07 to 5.05)	-1,000 (-2,090 to -29)	-0.477 (-0.993 to -0.014)	0.89 (0.80 to 1.00)
Slovenia	2020	Appendectomy	2,380	1.13	2,530 (2,390 to 2,680)	1.20 (1.14 to 1.27)	-147 (-293 to -9)	-0.070 (-0.139 to -0.004)	0.94 (0.89 to 1.00)
Slovenia	2021	Appendectomy	2,490	1.18	2,540 (2,400 to 2,680)	1.20 (1.14 to 1.27)	-46 (-195 to 93)	-0.022 (-0.093 to 0.044)	0.98 (0.93 to 1.04)
Slovenia	2020	Cholecystectomy	3,910	1.86	4,860 (4,440 to 5,330)	2.31 (2.11 to 2.53)	-949 (-1,410 to -520)	-0.451 (-0.670 to -0.247)	0.80 (0.74 to 0.88)
Slovenia	2021	Cholecystectomy	4,000	1.9	4,880 (4,450 to 5,340)	2.31 (2.11 to 2.53)	-879 (-1,350 to -460)	-0.417 (-0.642 to -0.218)	0.82 (0.75 to 0.90)
Slovenia	2020	Total mastectomy	614	0.292	629 (551 to 719)	0.299 (0.262 to 0.342)	-15 (-104 to 64)	-0.007 (-0.049 to 0.030)	0.98 (0.86 to 1.12)
Slovenia	2021	Total mastectomy	636	0.302	631 (522 to 762)	0.299 (0.248 to 0.361)	5 (-126 to 116)	0.002 (-0.060 to 0.055)	1.01 (0.83 to 1.22)
Slovenia	2020	Partial excision of mammary gland	1,390	0.66	1,560 (1,220 to 2,000)	0.744 (0.581 to 0.953)	-177 (-615 to 165)	-0.084 (-0.293 to 0.078)	0.89 (0.69 to 1.13)
Slovenia	2021	Partial excision of mammary gland	1,430	0.679	1,570 (1,110 to 2,220)	0.744 (0.525 to 1.06)	-138 (-780 to 325)	-0.065 (-0.370 to 0.154)	0.91 (0.65 to 1.29)
Slovenia	2020	Both	12,300	5.83	13,800 (12,700 to 15,100)	6.59 (6.03 to 7.19)	-1,590 (-2,850 to -446)	-0.755 (-1.35 to -0.212)	0.89 (0.81 to 0.96)
Slovenia	2021	Both	12,400	5.86	13,900 (12,300 to 15,700)	6.59 (5.82 to 7.46)	-1,520 (-3,420 to 92)	-0.722 (-1.62 to 0.044)	0.89 (0.78 to 1.01)
Slovenia	2020	Caesarean section	4,080	1.94	4,360 (4,210 to 4,520)	2.08 (2.00 to 2.15)	-290 (-446 to -140)	-0.138 (-0.212 to -0.067)	0.93 (0.90 to 0.97)
Slovenia	2021	Caesarean section	4,070	1.93	4,380 (4,220 to 4,540)	2.08 (2.00 to 2.15)	-308 (-467 to -153)	-0.146 (-0.221 to -0.072)	0.93 (0.90 to 0.96)
Slovenia	2020	Repair of inguinal hernia	3,280	1.56	4,210 (3,840 to 4,630)	2.00 (1.82 to 2.20)	-934 (-1,340 to -559)	-0.444 (-0.638 to -0.266)	0.78 (0.71 to 0.85)
Slovenia	2021	Repair of inguinal hernia	3,230	1.53	4,220 (3,850 to 4,640)	2.00 (1.82 to 2.20)	-993 (-1,410 to -618)	-0.471 (-0.668 to -0.293)	0.77 (0.70 to 0.84)
Slovenia	2020	Transluminal coronary angioplasty	4,230	2.01	4,720 (3,990 to 5,580)	2.24 (1.90 to 2.65)	-490 (-1,360 to 244)	-0.233 (-0.647 to 0.116)	0.90 (0.76 to 1.06)
Slovenia	2021	Transluminal coronary angioplasty	4,360	2.07	4,730 (3,730 to 6,000)	2.24 (1.77 to 2.85)	-365 (-1,640 to 639)	-0.173 (-0.780 to 0.303)	0.92 (0.73 to 1.17)
Slovenia	2020	Coronary artery bypass graft	681	0.324	808 (716 to 911)	0.384 (0.340 to 0.433)	-127 (-229 to -33)	-0.060 (-0.109 to -0.016)	0.84 (0.75 to 0.95)
Slovenia	2021	Coronary artery bypass graft	698	0.331	810 (718 to 914)	0.384 (0.340 to 0.433)	-112 (-214 to -21)	-0.053 (-0.102 to -0.010)	0.86 (0.77 to 0.97)

Slovenia	2020	Elective surgeries	27,200	12.9	31,900 (28,900 to 35,100)	15.2 (13.7 to 16.7)	-4,690 (-7,980 to -1,720)	-2.23 (-3.79 to -0.819)	0.85 (0.77 to 0.94)
Slovenia	2021	Elective surgeries	28,700	13.6	31,900 (27,800 to 36,700)	15.2 (13.2 to 17.4)	-3,250 (-7,990 to 843)	-1.54 (-3.79 to 0.400)	0.90 (0.78 to 1.03)
Slovenia	2020	Hip replacement	3,470	1.65	3,750 (3,160 to 4,450)	1.78 (1.50 to 2.12)	-280 (-997 to 315)	-0.133 (-0.474 to 0.150)	0.93 (0.78 to 1.10)
Slovenia	2021	Hip replacement	3,690	1.75	3,760 (3,170 to 4,460)	1.78 (1.50 to 2.12)	-68 (-770 to 522)	-0.032 (-0.365 to 0.248)	0.98 (0.83 to 1.16)
Slovenia	2020	Total knee replacement	2,200	1.05	2,880 (2,300 to 3,610)	1.37 (1.09 to 1.72)	-685 (-1,390 to -102)	-0.326 (-0.663 to -0.048)	0.76 (0.61 to 0.96)
Slovenia	2021	Total knee replacement	2,360	1.12	2,890 (2,100 to 3,980)	1.37 (0.997 to 1.89)	-536 (-1,600 to 262)	-0.254 (-0.761 to 0.124)	0.81 (0.59 to 1.13)
Slovenia	2020	Cataract surgery	17,600	8.36	19,700 (17,500 to 22,200)	9.37 (8.33 to 10.5)	-2,130 (-4,570 to 10)	-1.01 (-2.17 to 0.005)	0.89 (0.79 to 1.00)
Slovenia	2021	Cataract surgery	18,800	8.94	19,800 (16,700 to 23,300)	9.37 (7.94 to 11.1)	-921 (-4,500 to 2,220)	-0.437 (-2.14 to 1.05)	0.95 (0.81 to 1.13)
Slovenia	2020	Tonsillectomy	682	0.324	1,180 (841 to 1,660)	0.561 (0.400 to 0.788)	-498 (-971 to -155)	-0.237 (-0.462 to -0.074)	0.58 (0.41 to 0.81)
Slovenia	2021	Tonsillectomy	547	0.259	1,180 (843 to 1,660)	0.561 (0.400 to 0.788)	-636 (-1,110 to -294)	-0.302 (-0.527 to -0.140)	0.46 (0.33 to 0.65)
Slovenia	2020	Hysterectomy	1,840	0.876	2,200 (2,030 to 2,380)	1.04 (0.966 to 1.13)	-354 (-532 to -188)	-0.168 (-0.253 to -0.089)	0.84 (0.78 to 0.91)
Slovenia	2021	Hysterectomy	1,920	0.913	2,200 (2,040 to 2,380)	1.04 (0.966 to 1.13)	-277 (-452 to -110)	-0.131 (-0.214 to -0.052)	0.87 (0.81 to 0.95)
Slovenia	2020	Open prostatectomy	281	0.134	498 (400 to 621)	0.237 (0.190 to 0.295)	-217 (-340 to -120)	-0.103 (-0.162 to -0.057)	0.56 (0.45 to 0.70)
Slovenia	2021	Open prostatectomy	237	0.112	500 (366 to 682)	0.237 (0.174 to 0.324)	-263 (-447 to -129)	-0.125 (-0.212 to -0.061)	0.47 (0.35 to 0.65)
Slovenia	2020	Transurethral prostatectomy	1,120	0.534	1,270 (1,100 to 1,470)	0.605 (0.524 to 0.698)	-149 (-343 to 20)	-0.071 (-0.163 to 0.009)	0.88 (0.77 to 1.02)
Slovenia	2021	Transurethral prostatectomy	1,100	0.52	1,360 (1,110 to 1,660)	0.644 (0.526 to 0.789)	-262 (-563 to -9)	-0.124 (-0.267 to -0.004)	0.81 (0.66 to 0.99)
Sweden	2020	All surgeries	252,000	24.4	290,000 (268,000 to 312,000)	28.0 (25.9 to 30.2)	-37,300 (-60,500 to -16,200)	-3.60 (-5.84 to -1.57)	0.87 (0.81 to 0.94)
Sweden	2021	All surgeries	273,000	26.2	291,000 (262,000 to 324,000)	28.0 (25.1 to 31.1)	-17,900 (-50,700 to 11,400)	-1.72 (-4.87 to 1.09)	0.94 (0.84 to 1.04)
Sweden	2020	Non-elective surgeries	37,900	3.66	40,800 (39,400 to 42,200)	3.94 (3.80 to 4.08)	-2,920 (-4,380 to -1,480)	-0.282 (-0.424 to -0.143)	0.93 (0.90 to 0.96)
Sweden	2021	Non-elective surgeries	39,900	3.83	40,600 (38,900 to 42,400)	3.90 (3.73 to 4.07)	-707 (-2,490 to 1,010)	-0.068 (-0.239 to 0.097)	0.98 (0.94 to 1.03)
Sweden	2020	Appendectomy	13,100	1.27	12,700 (12,300 to 13,100)	1.22 (1.18 to 1.27)	467 (31 to 883)	0.045 (0.003 to 0.085)	1.04 (1.00 to 1.07)
Sweden	2021	Appendectomy	13,200	1.27	12,400 (11,600 to 13,400)	1.19 (1.11 to 1.28)	789 (-126 to 1,640)	0.076 (-0.012 to 0.157)	1.06 (0.99 to 1.14)
Sweden	2020	Cholecystectomy	13,500	1.31	14,700 (14,000 to 15,400)	1.42 (1.36 to 1.48)	-1,170 (-1,840 to -510)	-0.113 (-0.178 to -0.049)	0.92 (0.88 to 0.96)
Sweden	2021	Cholecystectomy	14,300	1.37	14,800 (14,100 to 15,500)	1.42 (1.36 to 1.48)	-515 (-1,200 to 127)	-0.049 (-0.115 to 0.012)	0.97 (0.92 to 1.01)
Sweden	2020	Total mastectomy	3,660	0.353	3,980 (3,690 to 4,290)	0.384 (0.356 to 0.415)	-322 (-640 to -31)	-0.031 (-0.062 to -0.003)	0.92 (0.85 to 0.99)
Sweden	2021	Total mastectomy	3,800	0.365	4,000 (3,590 to 4,460)	0.384 (0.345 to 0.428)	-198 (-659 to 221)	-0.019 (-0.063 to 0.021)	0.95 (0.85 to 1.06)

Sweden	2020	Partial excision of mammary gland	7,530	0.728	7,930 (7,160 to 8,780)	0.766 (0.692 to 0.848)	-398 (-1,240 to 374)	-0.038 (-0.120 to 0.036)	0.95 (0.86 to 1.05)
Sweden	2021	Partial excision of mammary gland	8,560	0.822	7,980 (7,210 to 8,840)	0.766 (0.692 to 0.848)	585 (-270 to 1,360)	0.056 (-0.026 to 0.130)	1.07 (0.97 to 1.19)
Sweden	2020	Both	53,900	5.21	61,900 (59,100 to 64,800)	5.98 (5.71 to 6.26)	-7,990 (-10,800 to -5,140)	-0.772 (-1.05 to -0.497)	0.87 (0.83 to 0.91)
Sweden	2021	Both	57,000	5.47	62,300 (59,500 to 65,200)	5.98 (5.71 to 6.26)	-5,280 (-8,120 to -2,490)	-0.507 (-0.780 to -0.239)	0.92 (0.88 to 0.96)
Sweden	2020	Caesarean section	20,000	1.93	20,600 (19,300 to 21,900)	1.99 (1.87 to 2.11)	-593 (-1,880 to 627)	-0.057 (-0.181 to 0.061)	0.97 (0.91 to 1.03)
Sweden	2021	Caesarean section	20,700	1.99	20,700 (19,500 to 22,000)	1.99 (1.87 to 2.11)	58 (-1,260 to 1,280)	0.006 (-0.121 to 0.123)	1.00 (0.94 to 1.07)
Sweden	2020	Repair of inguinal hernia	12,800	1.24	16,200 (14,900 to 17,600)	1.56 (1.44 to 1.70)	-3,390 (-4,840 to -2,080)	-0.327 (-0.467 to -0.201)	0.79 (0.73 to 0.86)
Sweden	2021	Repair of inguinal hernia	14,800	1.42	16,300 (14,400 to 18,400)	1.56 (1.39 to 1.77)	-1,500 (-3,560 to 324)	-0.144 (-0.342 to 0.031)	0.91 (0.81 to 1.02)
Sweden	2020	Transluminal coronary angioplasty	18,600	1.8	21,100 (19,700 to 22,700)	2.04 (1.90 to 2.19)	-2,490 (-4,030 to -1,070)	-0.241 (-0.390 to -0.103)	0.88 (0.82 to 0.95)
Sweden	2021	Transluminal coronary angioplasty	18,900	1.81	21,300 (19,300 to 23,500)	2.04 (1.85 to 2.25)	-2,380 (-4,550 to -397)	-0.228 (-0.437 to -0.038)	0.89 (0.81 to 0.98)
Sweden	2020	Coronary artery bypass graft	2,480	0.24	2,750 (2,550 to 2,960)	0.265 (0.246 to 0.286)	-264 (-482 to -65)	-0.026 (-0.047 to -0.006)	0.90 (0.84 to 0.97)
Sweden	2021	Coronary artery bypass graft	2,570	0.247	2,650 (2,380 to 2,950)	0.254 (0.229 to 0.283)	-81 (-376 to 186)	-0.008 (-0.036 to 0.018)	0.97 (0.87 to 1.08)
Sweden	2020	Elective surgeries	160,000	15.5	189,000 (168,000 to 213,000)	18.3 (16.2 to 20.6)	-28,600 (-52,300 to -7,190)	-2.76 (-5.05 to -0.695)	0.85 (0.75 to 0.96)
Sweden	2021	Elective surgeries	176,000	16.9	190,000 (161,000 to 225,000)	18.3 (15.4 to 21.6)	-13,700 (-48,400 to 15,600)	-1.31 (-4.65 to 1.50)	0.93 (0.78 to 1.10)
Sweden	2020	Hip replacement	21,000	2.03	25,600 (24,700 to 26,400)	2.47 (2.39 to 2.55)	-4,500 (-5,360 to -3,650)	-0.435 (-0.518 to -0.352)	0.82 (0.80 to 0.85)
Sweden	2021	Hip replacement	22,400	2.15	25,500 (24,500 to 26,600)	2.45 (2.36 to 2.55)	-3,120 (-4,160 to -2,120)	-0.300 (-0.399 to -0.203)	0.88 (0.84 to 0.91)
Sweden	2020	Total knee replacement	10,100	0.979	13,800 (12,700 to 15,000)	1.33 (1.23 to 1.45)	-3,680 (-4,860 to -2,580)	-0.355 (-0.470 to -0.250)	0.73 (0.68 to 0.80)
Sweden	2021	Total knee replacement	10,400	0.997	13,900 (12,800 to 15,100)	1.33 (1.23 to 1.45)	-3,510 (-4,750 to -2,400)	-0.337 (-0.456 to -0.230)	0.75 (0.69 to 0.81)
Sweden	2020	Cataract surgery	106,000	10.3	119,000 (99,400 to 142,000)	11.5 (9.60 to 13.8)	-12,600 (-36,800 to 7,340)	-1.22 (-3.55 to 0.709)	0.89 (0.74 to 1.07)
Sweden	2021	Cataract surgery	121,000	11.6	120,000 (92,800 to 154,000)	11.5 (8.91 to 14.8)	1,370 (-34,100 to 28,600)	0.132 (-3.27 to 2.74)	1.01 (0.78 to 1.31)
Sweden	2020	Tonsillectomy	9,450	0.913	13,600 (10,400 to 17,700)	1.31 (1.01 to 1.71)	-4,120 (-8,320 to -990)	-0.398 (-0.804 to -0.096)	0.70 (0.53 to 0.91)
Sweden	2021	Tonsillectomy	8,260	0.793	13,700 (9,420 to 19,800)	1.31 (0.904 to 1.90)	-5,400 (-11,500 to -1,150)	-0.519 (-1.10 to -0.111)	0.60 (0.42 to 0.88)
Sweden	2020	Hysterectomy	6,900	0.667	7,920 (7,200 to 8,700)	0.765 (0.696 to 0.841)	-1,020 (-1,780 to -304)	-0.098 (-0.172 to -0.029)	0.87 (0.79 to 0.96)
Sweden	2021	Hysterectomy	7,090	0.68	7,960 (6,970 to 9,110)	0.765 (0.669 to 0.874)	-878 (-2,000 to 151)	-0.084 (-0.192 to 0.014)	0.89 (0.78 to 1.02)

Sweden	2020	Open prostatectomy	2,930	0.283	3,560 (3,020 to 4,190)	0.344 (0.292 to 0.405)	-635 (-1,260 to -93)	-0.061 (-0.122 to -0.009)	0.82 (0.70 to 0.97)
Sweden	2021	Open prostatectomy	3,010	0.289	3,580 (3,040 to 4,220)	0.344 (0.292 to 0.405)	-568 (-1,210 to -24)	-0.055 (-0.116 to -0.002)	0.84 (0.71 to 0.99)
Sweden	2020	Transurethral prostatectomy	3,640	0.352	4,600 (4,200 to 5,050)	0.445 (0.405 to 0.488)	-962 (-1,420 to -555)	-0.093 (-0.137 to -0.054)	0.79 (0.72 to 0.87)
Sweden	2021	Transurethral prostatectomy	4,290	0.412	4,450 (3,910 to 5,080)	0.428 (0.375 to 0.487)	-166 (-777 to 379)	-0.016 (-0.075 to 0.036)	0.96 (0.85 to 1.10)

Table S8. Unadjusted and adjusted coefficients from mixed linear models quantifying the associations between health system characteristics, socio-demographic factors, public health and social measures, and COVID-19 severity on observed-to-expected (OE) ratios of inpatient hospitalizations

Parameter	Category	Standardized unadjusted coefficient	Standardized adjusted coefficient	Adjusted exponentiated coefficient
Total healthcare workers per capita (per 10 per 1,000 population)	Health system factors (2019)	0.078 (0.047, 0.108)	0.048 (0.005, 0.091)	1.020 (1.002, 1.039)
Doctors per capita (per 5 per 1,000 population)	Health system factors (2019)	0.027 (-0.023, 0.073)	-0.001 (-0.030, 0.027)	0.996 (0.825, 1.213)
Nurses per capita (per 5 per 1,000 population)	Health system factors (2019)	0.063 (0.025, 0.106)	0.009 (-0.043, 0.061)	1.011 (0.946, 1.087)
Care personnel per capita (per 5 per 1,000 population)	Health system factors (2019)	0.054 (0.015, 0.095)	0.003 (-0.037, 0.044)	1.003 (0.960, 1.051)
Hospital beds per capita (per 5 per 1,000 population)	Health system factors (2019)	-0.002 (-0.045, 0.041)	0.000 (-0.022, 0.022)	1.000 (0.956, 1.051)
Insurance coverage (per 5%)	Health system factors (2019)	0.083 (0.053, 0.112)	0.044 (0.017, 0.070)	1.052 (1.018, 1.088)
Share of GDP for health care (per 5%)	Health system factors (2019)	0.067 (0.033, 0.099)	0.008 (-0.024, 0.039)	1.021 (0.934, 1.112)
Share for inpatient care (per 5%)	Health system factors (2019)	-0.054 (-0.095, -0.016)	-0.020 (-0.043, 0.005)	0.980 (0.955, 1.005)
Share for outpatient care (per 5%)	Health system factors (2019)	-0.006 (-0.050, 0.040)	0.014 (-0.012, 0.039)	1.012 (0.991, 1.034)
Share for preventive care (per 5%)	Health system factors (2019)	0.011 (-0.031, 0.055)	-0.003 (-0.027, 0.021)	0.986 (0.899, 1.081)
Doctor consultations per capita (per 5 per 1,000 population)	Health system factors (2019)	-0.011 (-0.056, 0.034)	-0.004 (-0.029, 0.019)	0.987 (0.919, 1.060)
Log gross domestic product (GDP) per capita (per 10% change)	Socio-demographic factors (2019)	0.091 (0.065, 0.115)	0.048 (0.020, 0.075)	1.008 (1.003, 1.013)
Share of GDP for social spending (per 5%)	Socio-demographic factors (2019)	0.058 (0.022, 0.095)	0.014 (-0.029, 0.059)	1.013 (0.967, 1.060)
Income inequality	Socio-demographic factors (2019)	-0.038 (-0.078, 0.002)	-0.011 (-0.039, 0.016)	0.833 (0.551, 1.299)
Poverty rate	Socio-demographic factors (2019)	-0.023 (-0.064, 0.025)	0.007 (-0.018, 0.034)	1.200 (0.597, 2.456)
% population > 65 years and greater	Socio-demographic factors (2019)	0.033 (-0.006, 0.073)	0.019 (-0.007, 0.044)	1.026 (0.992, 1.060)

Pandemic preparedness (per 5 points)	Public health and social measures	0.049 (0.010, 0.093)	0.009 (-0.019, 0.038)	1.007 (0.983, 1.031)
Stringency index (per 10 points)	Public health and social measures	-0.016 (-0.026, -0.005)	-0.025 (-0.035, -0.014)	0.984 (0.977, 0.991)
Stay-at-home orders (per 10 days)	Public health and social measures	-0.034 (-0.055, -0.011)	-0.022 (-0.043, -0.003)	0.998 (0.996, 1.000)
School closures (per 10 days)	Public health and social measures	-0.027 (-0.041, -0.012)	-0.021 (-0.035, -0.006)	0.997 (0.995, 0.999)
Workplace closures (per 10 days)	Public health and social measures	-0.018 (-0.035, -0.001)	-0.016 (-0.031, 0.000)	0.998 (0.996, 1.000)
Mobility (per 10% change)	Public health and social measures	0.023 (0.007, 0.037)	0.006 (-0.015, 0.027)	1.005 (0.988, 1.025)
COVID-19 case rate (per 1 per 1000 population)	COVID-19 severity	0.011 (-0.001, 0.022)	-0.004 (-0.022, 0.011)	0.999 (0.996, 1.003)
COVID-19 death rate (per 1 per 1000 population)	COVID-19 severity	-0.020 (-0.034, -0.008)	-0.029 (-0.042, -0.015)	0.957 (0.937, 0.977)
COVID-19 hospitalization rate (per 1 per 1000 population)	COVID-19 severity	-0.004 (-0.022, 0.014)	-0.012 (-0.031, 0.007)	1.000 (0.999, 1.000)
Excess all-cause death rate (per 1 per 1000 population)	COVID-19 severity	-0.022 (-0.039, -0.006)	-0.023 (-0.038, -0.007)	0.978 (0.963, 0.993)
Excess all-cause death rate (without COVID) (per 1 per 1000 population)	COVID-19 severity	-0.008 (-0.034, 0.015)	0.005 (-0.016, 0.025)	1.008 (0.975, 1.041)
Excess avoidable death rate (per 10 per 1000 population)	COVID-19 severity	-0.089 (-0.123, -0.053)	-0.046 (-0.075, -0.014)	0.993 (0.988, 0.998)
Excess avoidable death rate (without COVID) (per 1 per 1000 population)	COVID-19 severity	-0.063 (-0.110, -0.011)	-0.044 (-0.079, -0.009)	0.908 (0.838, 0.983)

Caption: Coefficients are from mixed linear regressions with random intercept terms for country. Separate models were conducted for each variable of interest. In adjusted analyses, log-transformed income (pre-pandemic gross domestic product per capita), pre-pandemic health expenditure, the stringency index, and COVID-19 death rate were covariates. Owing to high correlation between variables, there some differences in included covariates in each regression. In the healthcare workers model, gross domestic product (GDP) per capita was removed due to very high correlation between GDP and healthcare workers (r=0.88). In COVID-19 severity models, COVID-19 deaths covariate was removed. In individual public health and social measure models (stay-at-home, school closures, workplace closures), the stringency index covariate was removed.

Table S9. Unadjusted and adjusted coefficients from mixed linear models quantifying the associations between health system characteristics, socio-demographic factors, public health and social measures, and COVID-19 severity on observed-to-expected (OE) ratios of surgical procedures

Parameter	Category	Standardized unadjusted coefficient	Standardized adjusted coefficient	Adjusted exponentiated coefficient
Total healthcare workers per capita (per 10 per 1,000 population)	Health system factors (2019)	0.072 (0.031, 0.111)	0.026 (-0.024, 0.071)	1.011 (0.990, 1.031)
Doctors per capita (per 5 per 1,000 population)	Health system factors (2019)	0.007 (-0.038, 0.053)	0.001 (-0.029, 0.032)	1.007 (0.816, 1.251)
Nurses per capita (per 5 per 1,000 population)	Health system factors (2019)	0.044 (-0.000, 0.082)	-0.002 (-0.041, 0.033)	0.997 (0.949, 1.043)
Care personnel per capita (per 5 per 1,000 population)	Health system factors (2019)	0.034 (-0.012, 0.077)	-0.030 (-0.070, 0.007)	0.973 (0.939, 1.006)
Hospital beds per capita (per 5 per 1,000 population)	Health system factors (2019)	0.009 (-0.038, 0.059)	0.034 (0.003, 0.069)	1.074 (1.006, 1.157)
Insurance coverage (per 5%)	Health system factors (2019)	0.088 (0.054, 0.121)	0.067 (0.023, 0.115)	1.142 (1.047, 1.255)
Share of GDP for health care (per 5%)	Health system factors (2019)	0.052 (0.007, 0.097)	0.017 (-0.019, 0.058)	1.049 (0.948, 1.180)
Share for inpatient care (per 5%)	Health system factors (2019)	-0.062 (-0.104, -0.021)	-0.030 (-0.068, 0.008)	0.970 (0.931, 1.008)
Share for outpatient care (per 5%)	Health system factors (2019)	0.014 (-0.030, 0.061)	0.011 (-0.025, 0.048)	1.009 (0.980, 1.039)
Share for preventive care (per 5%)	Health system factors (2019)	0.000 (-0.048, 0.049)	-0.007 (-0.043, 0.031)	0.974 (0.847, 1.129)
Doctor consultations per capita (per 5 per 1,000 population)	Health system factors (2019)	-0.014 (-0.066, 0.035)	0.027 (-0.008, 0.060)	1.087 (0.975, 1.204)
Log gross domestic product (GDP) per capita (per 10% change)	Socio-demographic factors (2019)	0.085 (0.050, 0.117)	0.051 (0.011, 0.091)	1.009 (1.002, 1.016)
Share of GDP for social spending (per 5%)	Socio-demographic factors (2019)	0.033 (-0.006, 0.071)	-0.005 (-0.063, 0.050)	0.994 (0.924, 1.065)
Income inequality (per 0.05 in Gini Index)	Socio-demographic factors (2019)	-0.049 (-0.094, -0.007)	-0.043 (-0.076, -0.012)	0.958 (0.926, 0.988)
Poverty rate	Socio-demographic factors (2019)	-0.030 (-0.075, 0.016)	-0.012 (-0.050, 0.024)	0.718 (0.261, 1.906)
% population > 65 years and greater	Socio-demographic factors (2019)	0.018 (-0.026, 0.065)	0.037 (0.001, 0.075)	1.061 (1.002, 1.128)

Pandemic preparedness (per 5 points)	Public health and social measures	0.048 (0.004, 0.096)	0.033 (-0.011, 0.077)	1.022 (0.993, 1.052)
Stringency index (per 10 points)	Public health and social measures	-0.031 (-0.046, -0.015)	-0.041 (-0.056, -0.024)	0.973 (0.963, 0.984)
Stay-at-home orders (per 10 days)	Public health and social measures	-0.001 (-0.032, 0.031)	0.015 (-0.013, 0.044)	1.002 (0.998, 1.005)
School closures (per 10 days)	Public health and social measures	-0.056 (-0.078, -0.034)	-0.048 (-0.069, -0.026)	0.994 (0.991, 0.997)
Workplace closures (per 10 days)	Public health and social measures	-0.016 (-0.040, 0.008)	-0.008 (-0.033, 0.017)	0.999 (0.996, 1.002)
Mobility (per 10% change)	Public health and social measures	0.060 (0.037, 0.083)	0.070 (0.038, 0.102)	1.075 (1.040, 1.112)
COVID-19 case rate (per 1 per 1000 population)	COVID-19 severity	0.029 (0.012, 0.045)	0.012 (-0.010, 0.036)	1.003 (0.998, 1.009)
COVID-19 death rate (per 1 per 1000 population)	COVID-19 severity	-0.034 (-0.053, -0.014)	-0.044 (-0.065, -0.023)	0.934 (0.904, 0.965)
COVID-19 hospitalization rate (per 10 per 1000 population)	COVID-19 severity	-0.021 (-0.050, 0.011)	-0.037 (-0.071, -0.005)	0.999 (0.997, 1.000)
Excess all-cause death rate (per 1 per 1000 population)	COVID-19 severity	-0.043 (-0.066, -0.021)	-0.038 (-0.061, -0.013)	0.960 (0.936, 0.986)
Excess all-cause death rate (without COVID) (per 1 per 1000 population)	COVID-19 severity	-0.031 (-0.060, 0.000)	0.006 (-0.021, 0.037)	1.010 (0.962, 1.069)
Excess avoidable death rate (per 10 per 1000 population)	COVID-19 severity	-0.081 (-0.150, -0.020)	-0.050 (-0.159, 0.051)	0.974 (0.920, 1.027)
Excess avoidable death rate (without COVID) (per 1 per 1000 population)	COVID-19 severity	0.021 (-0.062, 0.101)	0.059 (-0.027, 0.142)	1.159 (0.934, 1.427)

Caption: Coefficients are from mixed linear regressions with random intercept terms for country. Separate models were conducted for each variable of interest. In adjusted analyses, log-transformed income (pre-pandemic gross domestic product per capita), pre-pandemic health expenditure, the stringency index, and COVID-19 death rate were covariates. Owing to high correlation between variables, there some differences in included covariates in each regression. In the healthcare workers model, gross domestic product (GDP) per capita was removed due to very high correlation between GDP and healthcare workers (r=0.87). In COVID-19 severity models, COVID-19 deaths covariate was removed. In individual public health and social measure models (stay-at-home, school closures, workplace closures), the stringency index covariate was removed.
